# Genomics and evolutionary aspect of calcium signaling event in calmodulin and calmodulin-like proteins in plants

**DOI:** 10.1186/s12870-017-0989-3

**Published:** 2017-02-03

**Authors:** Tapan Kumar Mohanta, Pradeep Kumar, Hanhong Bae

**Affiliations:** 1 Free Major of Natural Science, College of Basic Studies, Yeungnam University, Gyeongsan, Gyeongsangbuk-do 38541 Republic of Korea; 2 School of Biotechnology, Yeungnam University, Gyeongsan, Gyeongsangbuk-do 38541 Republic of Korea

**Keywords:** Calmodulin, Calmodulin-like, Calcium signaling, EF-hands, Evolution

## Abstract

**Background:**

Ca^2+^ ion is a versatile second messenger that operate in a wide ranges of cellular processes that impact nearly every aspect of life. Ca^2+^ regulates gene expression and biotic and abiotic stress responses in organisms ranging from unicellular algae to multi-cellular higher plants through the cascades of calcium signaling processes.

**Results:**

In this study, we deciphered the genomics and evolutionary aspects of calcium signaling event of calmodulin (*CaM*) and calmodulin like- (*CML*) proteins. We studied the *CaM* and *CML* gene family of 41 different species across the plant lineages. Genomic analysis showed that plant encodes more calmodulin like-protein than calmodulins. Further analyses showed, the majority of *CMLs* were intronless, while *CaMs* were intron rich. Multiple sequence alignment showed, the EF-hand domain of CaM contains four conserved D-x-D motifs, one in each EF-hand while CMLs contain only one D-x-D-x-D motif in the fourth EF-hand. Phylogenetic analysis revealed that, the CMLs were evolved earlier than CaM and later diversified. Gene expression analysis demonstrated that different *CaM* and *CMLs* genes were express differentially in different tissues in a spatio-temporal manner.

**Conclusion:**

In this study we provided in detailed genome-wide identifications and characterization of CaM and CML protein family, phylogenetic relationships, and domain structure. Expression study of *CaM* and *CML* genes were conducted in *Glycine max* and *Phaseolus vulgaris*. Our study provides a strong foundation for future functional research in *CaM* and *CML* gene family in plant kingdom.

**Electronic supplementary material:**

The online version of this article (doi:10.1186/s12870-017-0989-3) contains supplementary material, which is available to authorized users.

## Background

In the nuclear fusion of stars and sun, the elements were evolved from hydrogen [1]. During the process of evolution, the element calcium (Ca) was born by successive capture of α particle by oxygen and neon in the process of nuclear fusion [1, 2]. After about 10 billion years, the cell membrane most likely shown its charged activity locally with relentless entropy [1]. To adapt to changing environment, cell must respond to changing environmental signals, and cellular signaling requires an efficient messenger that can move through all parts of the cell to decipher the message. Calcium ion commonly fulfills this signaling role. The concentrations of signaling molecules vary in the cell with time and environmental conditions. The speed and effectiveness of the Ca^2+^ ion is 20,000 fold higher in the intracellular (~100 nM) compartment than the extracellular (~2 mM) compartment [1]. Cells use a great deal of energy to induce changes in Ca^2+^ concentration and stabilize the cell. The concentration of Mg^2+^, which is popularly known as a cousin of Ca^2+^ doesn’t differ greatly across the cellular compartments. Then question arises, why the concentration of Ca^2+^ is very less in the cytosol? This is because Mg^2+^ binds the cytosolic water molecules less efficiently than phosphates. Therefore, if there will be higher Ca^2+^ concentrations in the cytosol, Ca^2+^ will bind with phosphate and thus turning the cell into a bone like structure. Unlike other complex molecules, Ca^2+^ cannot be altered chemically. Therefore, it is necessary to control the cytosolic Ca^2+^ concentration to avoid any precipitation with the phosphate in the cytosol. Hence, cells have developed necessary cellular mechanisms to control the cytosolic Ca^2+^ concentration by chelating, compartmentalizing or extruding the ion from the cell. Hence hundreds of proteins have evolved to bind the Ca^2+^ ion over a million-fold range of affinities (nM to mM) to buffer or lower Ca^2+^ level in the cell. One of the most important protein chelators of Ca^2+^ ion is the EF-hand domain containing proteins. There are hundreds of EF-hand containing proteins present in the plants. These proteins are found as family proteins. Some of the important EF-hand domain containing families of proteins are calcium dependent protein kinase (CDPK) [3, 4], calcium dependent protein kinase related kinase (CRK) [4], calcineurin-B like (CBL) [5], calmodulin (CaM) and calmodulin like (CML) protein [6]. The CDPK contains the kinase domain, auto-inhibitory domain and a regulatory domain that contains four calcium binding EF-hands while CRK contains kinase domain, auto-inhibitory domain and a regulatory domain that contain only three calcium binding EF-hands. Additionally, the CBL contains only three EF-hands and no kinase domain while CaM and CML contain only four EF-hands and lack a kinase domain [3, 6, 7]. The calcium ion binds to the Asp (D) or Glu (E) amino acids of the EF-hands. The D and E amino acids in the EF-hands are reported to be conserved and present as D-x-D or D/E-E-L motif [8, 9]. The D-x-D motifs are conserved at 14, 15 and 16^th^ position of the EF-hands [8, 9]. Detailed investigations of different genomics and evolutionary aspects of the CDPK and CBL protein family have been discussed recently [8, 9]. However, there have been only little information is available regarding the detail study of *CaM* and *CML* gene family in the plants. Therefore in this study, we conducted genome-wide identification of *CaM* and *CML* gene family members in plants and analyzed their genomic and evolutionary aspects. Along with the reports of CDPK and CBL protein family, this study completely unveils the genomic aspects of calcium signaling events in plants and calcium signature motifs in EF-hand domains.

## Results and discussion

### Genomics of CaMs and CMLs

Genome-wide identification of calmodulin (*CaM*) and calmodulin-like (*CML*) gene family members from plant shows, plant encodes more *CMLs* than *CaMs* (Table 1). The genome size of the green algae *Ostreococcus lucimarinus* was found to be 13.2 Mb and it encoded only two *CaMs*. The *Coccomyxa subellipsoidea* and *Chlamydomonas reinhardtii* encoded three and six *CaMs* respectively. The genome size of *Brassica rapa* and *Mimulus guttatus* was 283.8 and 321.7 Mb, respectively and both of them were found to encode 13 *CaMs* in their genome. The genomes of *M. guttatus* and *B. rapa* are diploid and both were found to encode 13 CaMs each. The genome size of *E. grandis* was found to be 691 Mb and contains only one *CaM* gene.

**Table 1:** The CaM and CML protein family members of different plant species. A particular protein was considered as either CaM or CML that contained only four calcium binding EF-hands. In total, 41 species were studied to identify CaM and CML protein family. In the table CaM stands for calmodulin and CML stands for calmodulin-like

Sl. no	Specie name	Gene abbreviation (CaM/CML)	Ploidy level	Genome size (Mbs)	Total No. of protein coding genes	No. of CaMs	No. of CMLs	% of CMLs compared to CaMs	Database version
1	*Aquilegia coerulea*	AcCaM/AcCML	Diploid	302	24823	5	21	420	Phytozome V10
2	*Arabidopsis thaliana*	AtCaM/AtCML	Diploid	135	27416	9	47	522.22	TAIR, 2015
3	*Brachypodium distachyon*	BdCaM/BdCML	Diploid	272	34310	5	23	460	Phytozome V10
4	*Brassica rapa*	BrCaM/BrCML	Diploid	283.8	40492	13	36	276.92	Phytozome V10
5	*Capsella rubella*	CrCaM/CrCML	Diploid	134.8	26521	10	29	290	Phytozome V10
6	*Carica papaya*	CpCaM/CpCML	Diploid	135	27332	5	15	300	Phytozome V10
7	*Chlamydomonas reinhardtii*	CreinCaM/CreinCML	Haploid	111.1	17741	6	3	50	Phytozome V10
8	*Citrus clementina*	CcCaM/CcCML	Diploid	301.4	24533	8	19	237.5	Phytozome V10
9	*Citrus sinensis*	CsCaM/CsCML	Diploid	319	25376	6	20	333.33	Phytozome V10
10	*Coccomyxa subellipsoidea*	CsubCaM/CsubCML	Haploid	49	9629	3	2	66.66	Phytozome V10
11	*Cucumis sativus*	CsatCaM/CsatCML	Diploid	203	21491	6	21	350	Phytozome V10
12	*Eucalyptus grandis*	EgCaM/EgCML	Diploid	691	36349	1	25	2500	Phytozome V10
13	*Fragaria vesca*	FvCaM/FvCML	Diploid	240	32831	5	19	380	Phytozome V10
14	*Glycine max*	GmCaM/GmCML	Tetraploid	975	56044	8	27	337.5	Phytozome V10
15	*Gossypium raimondii*	GrCaM/GrCML	Diploid	761.4	55294	6	30	500	Phytozome V10
16	*Linum usitatissimum*	LuCaM/LuCML	Diploid	318.3	43471	11	21	190.90	Phytozome V10
17	*Malus domestica*	MdCaM/MdCML	Diploid	881.3	63514	9	32	355.55	Phytozome V10
18	*Manihot esculenta*	MeCaM/MeCML	Diploid	532.5	33033	9	22	244.44	Phytozome V10
19	*Medicago truncatula*	MtCaM/MtCML	Diploid	241	50894	4	24	600	Phytozome V10
20	*Micromonas pusilla*	MpCaM/MpCML	Haploid	22	10660	5	8	160	Phytozome V10
21	*Mimulus guttatus*	MgCaM/MgCML	Diploid	321.7	26718	13	19	146.15	Phytozome V10
22	*Oryza sativa*	OsCaM/OsCML	Diploid	372	42189	5	33	660	RGAP 7
23	*Ostreococcus lucimarinus*	OlCaM/OlCML	Haploid	13.2	7796	2	2	100	Phytozome V10
24	*Panicum hallii*	PhCaM/PhCML	Diploid	554	37232	5	17	340	Phytozome V10
25	*Panicum virgatum*	PvCaM/PvCML	Tetraploid	1358	102065	9	20	222.22	Phytozome V10
26	*Phaseolus vulgaris*	PvulCaM/PvulCML	Diploid	521.1	27197	9	26	288.88	Phytozome V10
27	*Physcomitrella patens*	PpCaM/PpCML	Haploid	480	32926	7	17	242.85	Phytozome V10
28	*Picea abies*	PaCaM/PaCML	Diploid	1960	28354	9	15	166.66	Congenie V1
29	*Populus trichocarpa*	PtCaM/PtCML	Diploid	422.9	41335	8	26	325	Phytozome V10
30	*Prunus persica*	PperCaM/PperCML	Diploid	227.3	26873	4	21	525	Phytozome V10
31	*Ricinus communis*	RcCaM/RcCML	Diploid	400	31221	4	8	200	Phytozome V10
32	*Selaginella moellendorffii*	SmCaM/SmCML	Haploid	212.5	22273	6	11	183.33	Phytozome V10
33	*Setaria italica*	SiCaM/SiCML	Diploid	405.7	34584	5	17	340	Phytozome V10
34	*Solanum lycopersicum*	SlCaM/SlCML	Diploid	900	34727	9	27	300	Phytozome V10
35	*Solanum tuberosum*	StCaM/StCML	Diploid	800	35119	5	27	540	Phytozome V10
36	*Sorghum bicolor*	SbCaM/SbCML	Diploid	697.5	34211	8	22	275	Phytozome V10
37	*Thellungiella halophila*	ThCaM/ThCML	Diploid	238.5	26351	10	27	270	Phytozome V10
38	*Theobroma cacao*	TcCaM/TcCML	Diploid	346	29452	2	14	700	Phytozome V10
39	*Vitis vinifera*	VvCaM/VvCML	Diploid	748	26346	5	13	260	Phytozome V10
40	*Volvox carteri*	VcCaM/VcCML	Haploid	125.4	14247	4	4	100	Phytozome V10
41	*Zea mays*	ZmCaM/ZmCML	Diploid	2500	63540	8	21	262.5	Phytozome V10

**Fig. 1:**
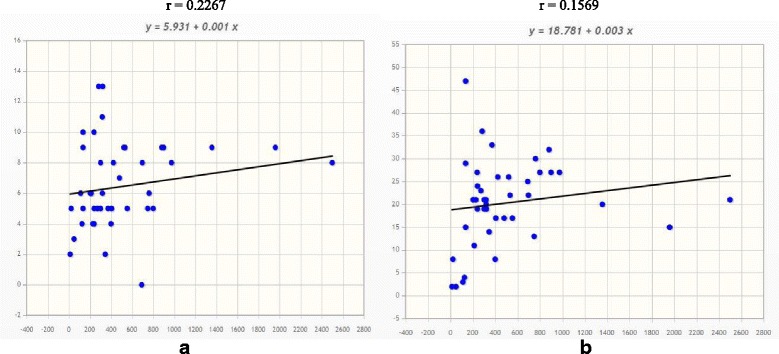
Regression analyses of number of *CaM* and *CML* genes in different genomes. **a** Correlation regression of *CaM* gene number in different genomes. Most of the genome encodes four to ten *CaM* genes. The increase in the genome size is not related to the increase in number of *CaM* genes in the genome. The correlation coefficient of the analysis was *r* = 0.2267. **b** Correlation regression analysis of *CML* genes. Similar to the *CaM* gene family, the number of *CML* genes in a genome does not increase with increase with genome size. The correlation coefficient of *CML* was *r* = 0.1569

The average number of *CaMs* in plant was found to be 6.60 per genome and the majority of the plants encode less than 10 *CaMs* in its genome. The size of plant genome vary from species to species, and these variations are completely depends on the ploidy and duplication events of the genome. However, the variations in the number of genes in a gene family were not directly correlated with the genome size, ploidy or genome duplication events of an organism. The correlation regression analysis of *CaMs* and *CMLs* with respect to genome size has shown that they are not correlated (Fig. 1). The correlation coefficient of *CaM* was *r* = 0.2267 and that of *CML* was *r* = 0.1569. The tetraploid species *Glycine max* and *Panicum virgatum* encoded eight and nine *CaMs* respectively which is less than the *CaMs* of the diploid species *B. rapa* and *M. guttatus* (Table 1, Additional file 1: Table S1). The normal distribution analysis shows, the probability of genome that can encode CaMs more than once was 0.9767 (97.67%) (Table 2). Similarly, the probability to encode more than 13 *CaM* in a genome was only 0.0113 (1.13%). The details regarding the probability of distribution of *CaM* among different groups of organisms in plant lineage are mentioned in Table 2. These findings show that the presence and distribution of varied gene number and type of gene in a genome is dependent on the evolutionary pressure, it functional requirements and complexities of the plant. Two sample t-tests between CML and CaM were conducted and the mean of CML and CaM was found to be 20.26 and 6.60 respectively (Table 3). The t-value of unpaired and paired *t*-test was found to be 8.91 and 10.43, respectively.

**Table 2:** Normal distribution of CaMs and CMLs in plant lineage. In the table, P = probability, X = CaMs/CMLs, * = lowest number of CaMs/CMLs in the specified group, ** = highest number of CaMs/CMLs in the specified group

Species group	No. of species (N)	Mean	Standard deviation	Normal Distribution		
CaMs						
Haploid	7	4.7143	1.7995	0.9345 P (X > 2*)	0.2389 P (X > 6**)	0.7611 P (X < 6**)
Diploid	32	6.7879	2.9343	0.9756 P (X > 1*)	0.017 P (X > 13**)	0.983 P (X < 13**)
Tetraploid	2	8.5	0.7071	0.7611 P (X > 8*)	0.2389 P (X > 9**)	0.7611 P (X < 9**)
Monocot	6	6.4286	1.8127	0.7852 P (X > 5*)	0.0778 P (X > 9*)	0.8222 P (X < 9**)
Dicot	27	7.1429	3.0026	0.9798 P (X > 1*)	0.0256 P (X > 13**)	0.9744 P (X < 13**)
All	41	6.60	2.8096	0.9767 P (X > 1*)	0.0113 P (X > 13**)	0.9887 P (X < 13**)
CMLs						
Haploid	7	6.7143	5.6484	0.7967 P (X > 2*)	0.0344 P (X > 17**)	0.9656 P (X < 17**)
Diploid	32	22.3939	8.2838	0.9931 P (X > 2*)	0.0015 P (X > 47**)	0.9985 P (X < 47**)
Tetraploid	2	23.5	4.9497	0.7611 P (X > 20*)	0.2389 P (X > 27**)	0.7611 P (X < 27**)
Monocot	6	21.85	5.4292	0.8133 P (X > 17*)	0.0202 P (X > 33**)	0.9798 P (X < 33**)
Dicot	27	23.3704	7.8503	0.975 P (X > 8*)	0.0013 P (X > 47**)	0.9987 P (X < 47**)
All	41	20.26	9.3995	0.9738 P (X > 2*)	0.0022 P (X > 47**)	0.9978 P (X < 47**)

**Table 3:** Two sample *t*-test between CMLs and CaMs

Statistical parameters	Group 1	Group 2
Mean	20.26	6.6098
Variance	88.3512	7.8939
Stand. Deviation	9.3995	2.8096
n	41	41
Unpaired test		
t	8.91	
Degree of freedom	47	
Critical value	2.013	
Paired test		
t	10.4323	
Degree of freedom	41	
Critical value	2.021	

Group 1 indicates CMLs and group 2 indicates CaMs. Different parameters used to run the statistical analysis was as follows: group description, groups have unequal variance; number of tails, two tailed test; significance level (*P*), 0.05; test, unpaired and paired *t*-test. In the table n signifies number of samples. In both the paired and unpaired test, group 1 and 2 are significantly different at *p* < 0.05

Genome-wide analysis of the *CML* gene family in plants showed that the green algae *C. subellipsoidea*, *O. lucimarinus*, and *C. reinhardtii* encoded lower numbers of *CMLs* than the higher plants (Table 1). The genome of *C. subellipsoidea* and *O. lucimarinus* encoded only three and two CaMs, respectively. These two species encoded the same number of *CMLs*, whereas *C. reinhardtii* encoded six *CaMs* and three *CMLs* respectively. *C. reinhardtii* encoded more *CaMs* (six) than *CMLs* (three). Conversely, *O. lucimarinus* encoded equal numbers of C*aMs* and *CMLs* (two). When compared with *O. lucimarinus*, *V. carteri* was also found to contain similar numbers of *CaMs* and *CMLs* (four) (Table 1). *A. thaliana* encoded maximum of 47 *CML* genes while *B. rapa* encoded 36 *CMLs*. The tetraploid species *G. max* and *P. virgatum* encoded 26 and 20 *CMLs* respectively. The monocot plant *O. sativa* encoded 33, while *S. bicolor* and *P. hallii* encoded 27 *CMLs*. On the other hand, *P. patens*, and *S. italica* encoded 17 *CMLs* each. *C. clementina*, *F. vesca*, and *M. guttatus* encoded 19; *A. coerulea*, *C. sativus*, *L. usitatissimum*, *P. persica*, and *Z. mays* encoded 21 *CMLs* each. *G. max*, *S. lycopersicum*, *S. tuberosum*, and *T. halophila* encoded 27 *CMLs* each. This distribution of the *CML* gene family shows, several plant species has encoded the same numbers of *CML* genes while other do not. The percentage analysis comparison between *CaM* and *CML* shows, *T. cacao* encoded 700% and *O. sativa* 660% more *CMLs* compared to their counterpart *CaMs* (Table 1). The normal distribution study shows, the probability of occurrence of more than two *CMLs* in a plant genome was 0.9706 (97.06%) while the probability of occurrence of more than 47 *CMLs* was 0.0024 (0.24%) only (Table 2). The details about the probability of distribution of the *CMLs* among different groups are mentioned in Table 2. The student’s *t*-test was conducted to understand the significance of differences between gene numbers present between *CaM* and *CML* gene family. Both unpaired and paired *t*-test analysis shows *CaM* and *CML* gene family group members were significantly different from each other (Table 3). These changes in gene family size and unequal distribution of *CaMs* and *CMLs* may be attributed to their ploidy level and different cellular processes require for different plants [10], but they were not related to the size of the genome (Fig. 1). Because in principle, addition or evolution of more genes or genomic content within the genome will lead to increase in the genome size, but vice versa (increase in genome size will lead to more number of genes in a genome) is not true. This might have occurred because of the different cellular and ecological strategies associated with adaptation and expansion of the gene family [10–12]. The variations in the gene family size were largely attributed to the important mechanisms that shape natural variation and adaptation in different species [13].

### CMLs and CaMs Contain varied numbers of introns

Genome-wide analysis of the *CML* gene family in plants revealed that larger parts of the *CMLs* were intronless. Among the studied 831 *CMLs* of 41 species, 596 genes (71.72%) were identified to be intronless (Additional file 2: Table S2) whereas 79 had one intron (9.5%), 24 had two introns (2.88%), 44 had three introns (5.29%), 29 had four introns (3.48%), and 15 had five introns (1.8%). Only a few *CMLs* contained six, seven, eight or nine introns, and none of them were found to contain ten or more than ten introns (Additional file 2: Table S2). In opposite to *CMLs*, the majorities of *CaMs* were contained introns. Among the studied 271 *CaMs* of 41 species, 14 (5.16%) were found to be intronless, 113 (41.69%) contained one, 35 (12.91%) contained two, 86 (31.73%) contained three, six (2.21%) contained 4, five (1.84%) contained five, and seven (2.58%) contained six introns respectively. The evolutionary perspectives regarding the presence of introns in eukaryotic protein coding genes are not yet clear. However, Mattic [14] reported that introns can function as a transposable element and nuclear introns has originated from the self splicing group II introns, which later evolved in conjunction with the spliceosome. It assumed that these introns were evolve after divergence from the prokaryotes and later established in the eukaryotic genome with new genetic space and function, which provided a positive pressure for their expansion [14]. According to this concept, it can be speculated that the majority of *CMLs* were intronless and can therefore be considered older than *CaMs*. A few *CMLs* contains introns in their genes, and it is believed that these introns were evolved recently with *CaMs*. This explains why the intron containing *CMLs* contains only one (9.5%) intron in their gene. Similarly, a few *CaMs* were also intronless (5.5%), which indicates that the genome has yet to incorporate the introns into the *CaMs*. Some other *CaMs* contains either one (42.44%), two (34.81%) or three (32.22%) introns. This could be possible because these introns were might have added recently and the genome did not got ample time to add more introns into the *CaMs*. Similarly, the introns present in *CMLs* are assumed to have been added recently. It requires sufficient time to carry out a major evolutionary event and the addition of more introns into a gene.

According to the intron late hypothesis, introns are the eukaryotic novelty and new introns are emerging continuously during the evolution of eukaryotic genome [15]. Different genes in eukaryotic organisms differ dramatically in terms of density and size distribution. In some cases, zero to six introns per kilobase were observed in the eukaryotic genome [15, 16]. Comparative analysis of exon-intron structures of orthologous genes in higher eukaryotic organisms revealed that they share approximately 25% to 30% of the introns [15]. The presence of 71.72% intronless genes in *CMLs* shows that the *CMLs* of plants are highly orthologous and conserved genes in the plant kingdom that evolved from a common ancestor. Similarly, the presence of 42.44%, 34.81% and 32.22% similarity for one, two and three introns containing genes, respectively, shows their close homology with orthologous genes. Intron loss events dominate the short evolutionary distances, whereas intron gain accompanies important evolutionary transitions. Intron gain is an ongoing process, and a high rate of intron gain has been reported for paralogous genes in the model plant *Arabidopsis thaliana* and *Oryza sativa* [17–19]. The shared introns were likely derived from a common ancestor of the corresponding species, while the lineage-specific introns were introduced into the genes at the subsequent stages of evolution.

### CaM contains four D-x-D motifs and CML contains One D-x-D-x-D motif in their EF-hands

**Fig. 2:**
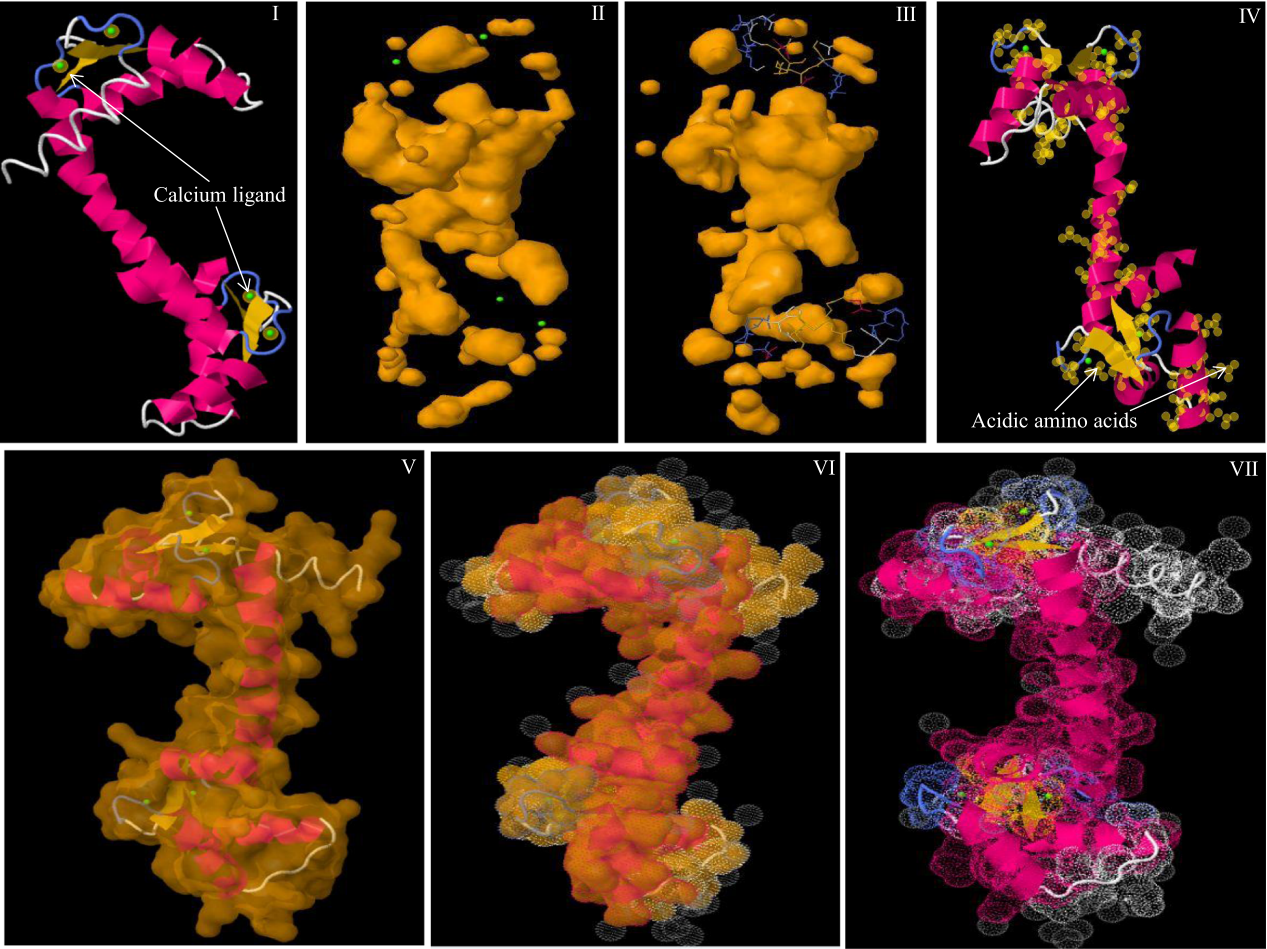
Three dimensional structure of CaM protein. (I) Secondary structure of the CaM protein where calcium ligand was found to bind the N- and C-terminal end of the EF-hands. Four calcium ligands bind to the four EF-hands. (II) The cavity model of CaM and calcium ligands shown in green, (III) ligands and pockets in cavity model, (IV) distribution of acidic amino acids in CaM, (V) molecular structure (VI) van der Waals surface, and (VII) dot surface. The protein sequence of *Arabidopsis thaliana* AtCaM1 was used as a query sequence to model the figure. The protein model was created using Geno3D software

**Fig. 3:**
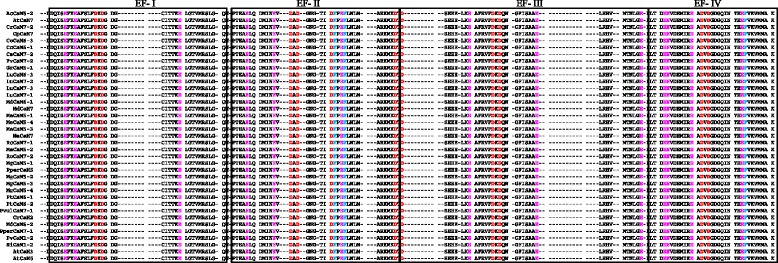
Multiple sequence alignment of CaM protein sequences. The sequence alignment shows presence of four conserved D-x-D motifs, one in each EF hand. The D-x-D motif was conserved at positions 15, and 17 in each EF-hand. In addition to the presence of conserved D-x-D motifs in the EF-hands, CaMs were also contained several conserved D (aspartate) and E (glutamate) amino acids in each EF-hand. The D and E amino acids are prominent calcium sensors that bind calcium ions in the EF-hands. Therefore, CaM contains several conserved D and E amino acids in the EF-hands to increase the calcium binding affinity. Multiple sequence alignment was conducted using Multalin software

CaMs and CMLs are evolutionarily conserved gene families of plants, therefore it was very important to understand their conserved domains and motifs. Hence, we conducted multiple sequence alignment of CaMs and CMLs protein sequences separately to identify the conserved domains and motifs. Multiple sequence alignment has revealed the presence of several conserved domains and motifs. The CaM protein contains four calcium binding EF-hands (Fig. 2). Multiple sequence alignment of CaMs revealed the presence of four D-x-D motifs in four EF-hand domains (Fig. 3, Additional file 3: Figure S1). Each EF-hand domain contains one D-x-D motif and the motif was conserved at position 14^th^, and 16^th^ in all of the EF-hands. In addition to the presence of a D-x-D motif in the EF-hands, the first EF-hand contains a conserved E-x_2_-E motif that conserved at 5^th^, and 8^th^ position. Besides this it was found to contain a conserved E amino acid at position 25^th^ of the 1^st^ EF-hand (Fig. 3). The second EF-hand contained a conserved E amino acids at positions 5^th^ and 12^th^, respectively; a conserved D-F-x-E-F domain at the position 22^nd^, 23^rd^, 25^th^ and 26^th^, respectively and a conserved D amino acid at position 36^th^ (Fig. 3). The third EF-hand contained conserved D and E amino acids at the 1^st^, and 8^th^ position respectively, and conserved E amino acids at position 25^th^ and 36^th^. The fourth EF-hand contained conserved E amino acids at the 4^th^, 5^th^, 12^th^ and 25^th^ position. A conserved E amino acid was found to present at 5^th^ position in the first, second and fourth EF-hand. Similarly, a conserved E amino acid was also found to present at position 25^th^ in all four EF-hands (Fig. 3). The first and fourth EF-hands contain no conserved amino acids at the 36^th^ position, while the second and third EF-hands contained a conserved D and E amino acid respectively the 36^th^ position respectively.

**Fig. 4:**
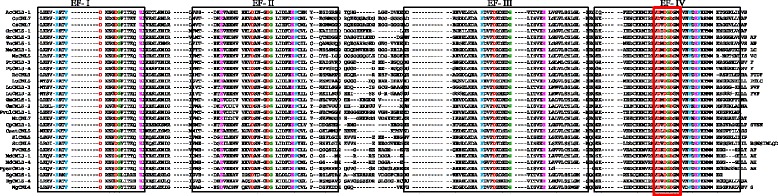
Multiple sequence alignment of CML protein sequences. The sequence alignment shows presence of the D-x-D-x-D motif in the fourth EF-hand. The D-x-D-x-D motif was conserved at 14^th^, 16^th^, and 18^th^ position. In addition to the presence of a conserved D-x-D-x-D motif, CML also found to contain several conserved D and E amino acids in other EF-hands. The multiple sequence alignment was conducted using the Multalin software

**Fig. 5:**
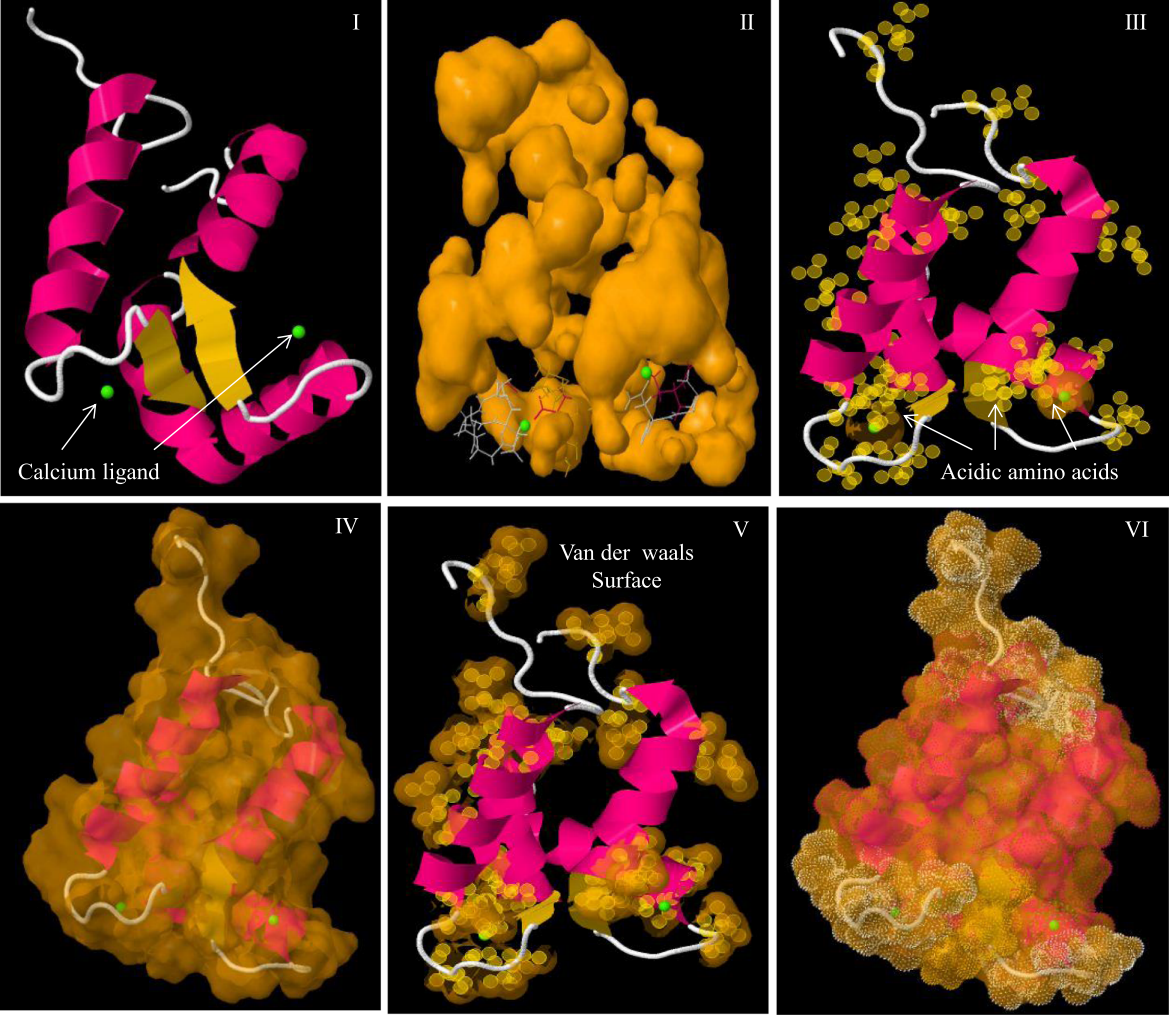
Three dimensional structure of CML protein. (I) Secondary structure of CML showing the binding site of calcium ligand in the C-terminal region, (II) cavity model of CML showing ligands and pockets, (III) distribution of acidic amino acids in CML, (IV) molecular structure of CML (V) van der Waals surface of CML, and (VI) dot surface of CML. The protein sequence of *Arabidopsis thaliana* AtCML1 was used as a query sequence to model this ure. The protein model was created using the Geno3D software

Unlike the CaMs, CMLs were also found to contain four calcium binding EF-hand domains (Fig. 4). Each EF-hand is around 36 amino acids long and contains a conserved aspartate (D) and glutamate (E) amino acid in the EF-hands. The EF-hand has a helix-loop-helix structure that coordinates the calcium ion. Multiple sequence alignment of CMLs shows the presence of a conserved D-x-D-x-D motif in the fourth EF-hand (Fig. 4, Additional file 4: Figure S2) that is conserved at 14^th^, 16^th^, and 18^th^ position. No other EF-hands were found to contain conserved D-x-D motifs. Instead, they contain some other conserved amino acid at different positions. The first EF-hand contained conserved F-x_2_-F motif at the 5^th^ and 8^th^ position and a calcium binding D-x_3_-D motif at 9^th^ and 13^th^ position. Glycine (G) was found to conserve at position 14^th^ and glutamate (E) was conserved at position 20^th^ in the first EF-hand. Unlike the first EF-hand, the second EF-hand was also contained a conserved D-x_3_-D motif at the 13^th^ and 17^th^ position. Glycine was found to conserve at position 18^th^ and E and F were conserved at position 24^th^ and 25^th^, respectively, in the second EF-hand. In the third EF-hand, F was conserved at position 10^th^, while D and E were conserved at positions 14^th^ and 25^th^ respectively. In addition to the presence of a D-x-D-x-D motif in the fourth EF-hand, it was also found to contain a conserved F-x-E-F domain. The calcium sensor protein, calcium dependent protein kinase (CPK) contains a kinase domain and four calcium binding EF-hands. The EF-hand domain of CPK contains conserved D-x-D motifs in each EF-hand. The D-x-D motifs in the EF-hands of CPKs are conserved at positions 14^th^, 15^th^ and 16^th^ similar to the D-x-D motifs of CaMs. The D-x-D-x-D motif of CML was conserved at 14^th^, 16^th^, and 18^th^ position of the EF-hand. The molecular structure of CML also revealed about the presence of only two calcium binding ligand pockets in the C-terminal region of the EF-hand (Fig. 5). These finding indicated that, the fourth EF-hand of CML present in the C-terminal region is more functional than the other three EF-hands. The two EF-hands of the N-terminal region and the first EF-hand of the C-terminal region (third EF-hand) don’t have any calcium binding ligand pockets. This may be the reason that CMLs might have undergone evolutionary changes to modified to CaMs and to add four calcium sensing D-x-D motif in it and hence they contain introns in the *CaM* gene. Although the D-x-D motifs were conserved at similar positions in the CaM and CPKs, only the CML contains the D-x-D-x-D motif in the fourth EF-hand while CaM contains the D-x-D motif in all four EF-hands. These findings show that the EF-hands present in CPKs are much similar to CaMs than that of CMLs. The presence of four EF-hand domains in CMLs, similar to that of CaMs and CPKs as well as the absence of a conserved D-x-D motif from all EF-hand domains of CML shows that they have developed recently and have yet to gain complete structural conservation unlike CaM and CPK.

### CML contain signal sequences while CaM do not

**Fig. 6:**
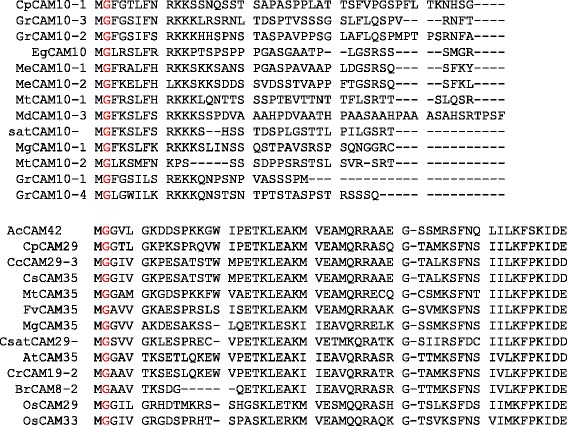
Myristoylation site of CMLs. The CMLs were found to contain putative myristoylation sites in the N-terminal end. Glycine amino acid, which undergoes myristoylation, was conserved at 2^nd^ position in the N-terminal end. Approximately 7.58% of the CMLs were found to contain putative myristoylation sites

Proteomes are larger and more dynamic than genomes because of the presence of abundant alternative splicing of genes and expanded functional and chemical complexities at the protein level due to post-translational modifications. This explains to some extent why larger genomes do not automatically translate into more complex organisms. Post-translational modifications lead to incorporation of new chemistry and molecular functions that cannot be precisely encoded by gene sequences. The posttranslational modifications event including myristoylation and palmitoylation have incredibly diverse biological functions in signaling, protein trafficking, localization, extracellular communication, protein regulation and metabolism. Co-translational and irreversible addition of myristic acid to N-terminal glycine residues are known as myristoylation. The N-terminal glycine residues that undergo protein myristoylation are usually conserved at the second position in the N-terminal region. Therefore, we analyzed about the presence of putative myristoylation and palmitoylation sites in CaMs and CMLs using CSS Palm software version 4.0. Our analysis revealed that the CaMs do not contain any palmitoylation or myristoylation sites. However, myristoylation sites were predicted in few CMLs (CML10, CML21, CML25, CML29, CML33, and CML34) (Additional file 4: Figure S2, highlighted in yellow). Approximately 63 (7.58%) of the 831 studied CMLs were found to contain glycine (G) amino acid residue at the second position of N-terminal end. The myristoylation motif found in CMLs were M-G-F, M-G-G and M-G-x (Fig. 6) where G amino acid was found at the 2^nd^ position of the N-terminal end. The CPKs were also reported to contain conserved myristoylation motif including M-G-C, and M-G-N at the N-terminal end [8]. Although the G amino acid was conserved at the second position in CML and CaM, the third position was dynamic. The palmitoylation and myristoylation events are sometimes correlated, and the absence of myristoylation may abolish the palmitoylation. When myristoylation of OsCPK2 was abolished by removing the N-terminal G amino acid, the protein could no longer be palmitoylated [20]. These finding indicated that the myristoylation event is pre-requisite to palmitoylation. The absence of a myristoylation and palmitoylation site in CaM likely forced it to merged with the kinase domain resulting in evolution of CPK that contains palmitoylation and myristoylation site in the N-terminal region. Similarly, the presence of myristoylation sites in a few CMLs shows that the palmitoylation site has evolved recently in these proteins. Although the myristoylation has been shown to be pre-requisite to palmitoylation the same is not true for myristoylation. The myristoylation event might have occur independently without the requirement of a palmitoylation site. This is because neither CaM nor CML were found to contain any palmitoylation sites.

### CMLs were evolved earlier than the CaMs

**Fig. 7:**
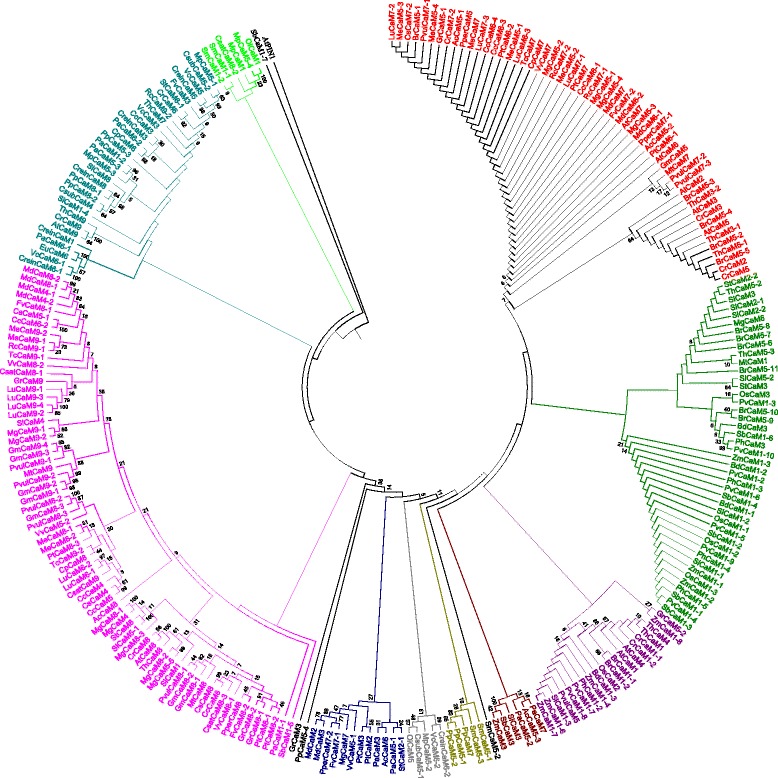
Phylogenetic tree of CaMs. The phylogenetic tree shows presence of ten monophyletic groups of CaM. The CaMs of the lower eukaryotic plants were found at the base of the phylogenetic tree, indicating that the CaMs of higher plants were evolved from the common ancestor of basal lower eukaryotic lineage. The phylogenetic tree was constructed from the protein sequences of CaMs using the MEGA6 software

**Fig. 8:**
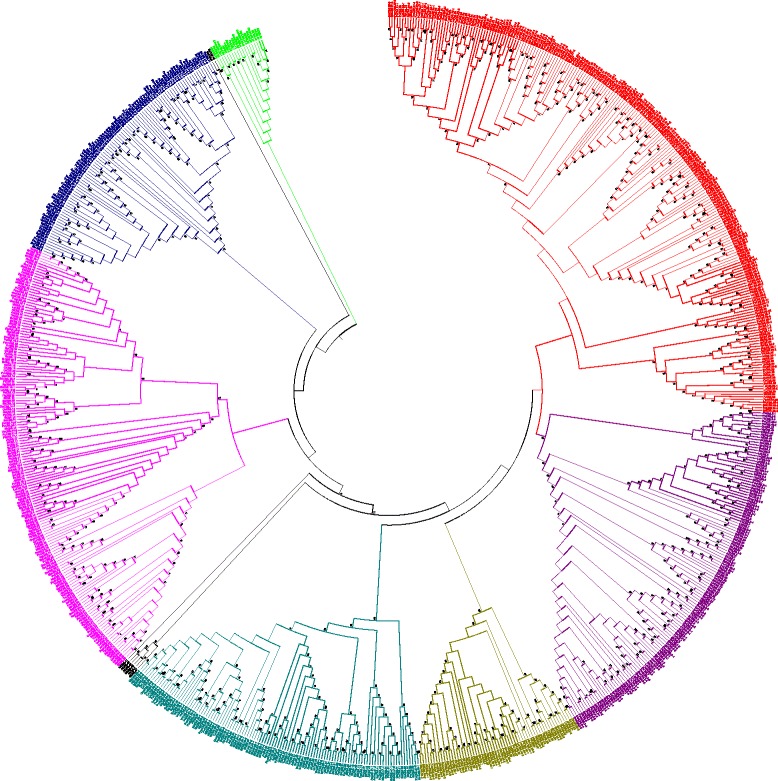
Phylogenetic tree of CMLs. The phylogenetic tree shows presence of seven monophyletic groups of CMLs. The CMLs of lower eukaryotic plants were located at the base of the phylogenetic tree, indicating CMLs of higher plants have evolved from the common ancestor of basal lower eukaryotic lineage. The phylogenetic tree was constructed from the protein sequences of CMLs using the MEGA6 software

**Fig. 9:**
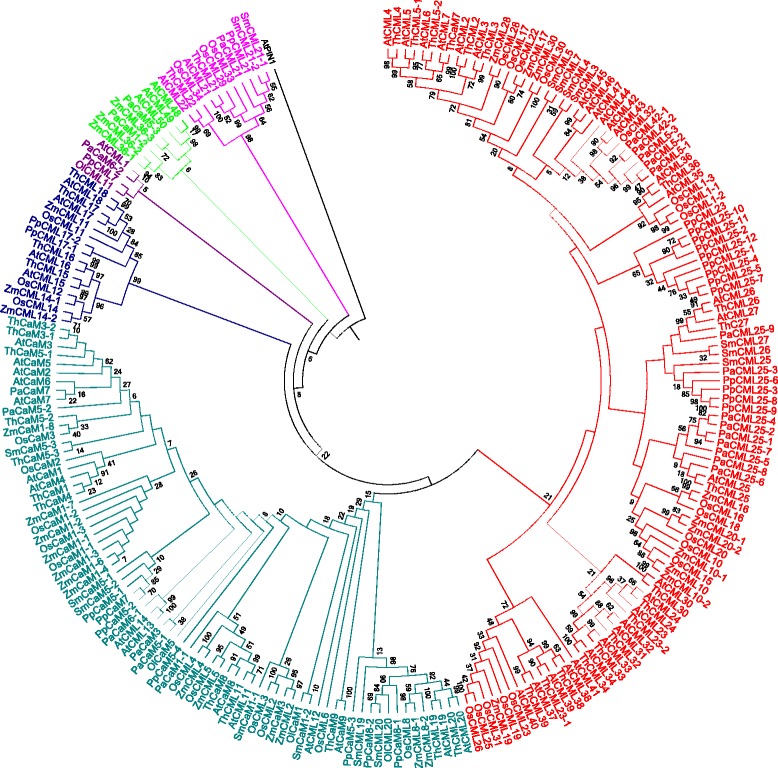
Phylogenetic tree of CaMs and CMLs. The phylogenetic tree shows presence of six monophyletic groups. The monophyletic groups were shared by CaM and CMLs. The phylogenetic analysis shows that CMLs were evolved earlier than CaMs and diversified which led to generation of CaMs. The phylogenetic tree was built using the protein sequences of CaMs and CMLs of *A. thaliana, O. lucimarinus, O. sativa, P. abies, P. patens* and *Z. mays*

**Fig. 10:**
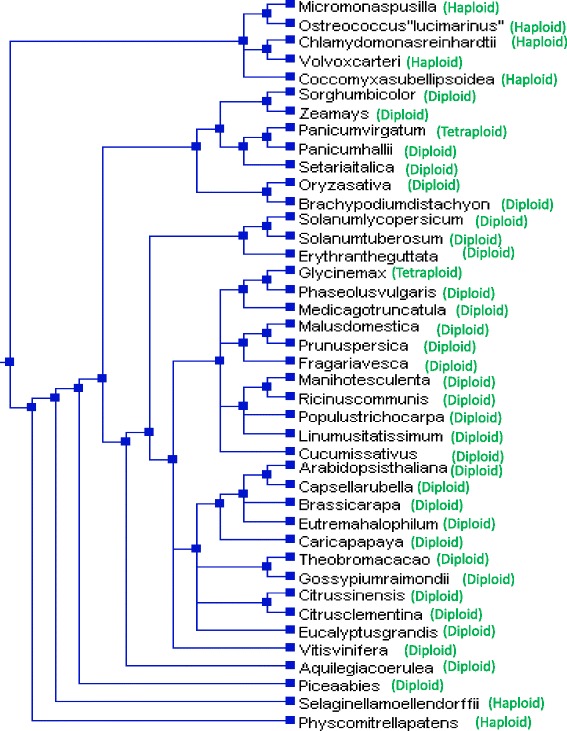
Species tree of studied plants. Species tree shows, higher plants were evolved from their basal lower eukaryotic lineages

Phylogenetic trees were constructed to understand the evolution of CaMs and CMLs. The phylogenetic tree was constructed by taking the protein sequences of CaMs resulted in a single monophyletic clades with different groups and shows that they have evolved from a common ancestor of lower eukaryotic plants lineages (Fig. 7). We named them as group I (red), II (green), III (purple), IV (marron), V (olive), VI (silver), VII (blue), VIII (fuschia), IX (teal) and X (lime). The majorities of CaMs were clustered in group I (red), II (green), III (purple), VIII (fuschia) and IX (teal). The CaM group of *Ostreococus, Micromonas, Selaginella, Volvox, Chlamydomonas* and *Picea* forms the basal root of the phylogenetic tree. These finding show the that plant CaMs were evolved from their common ancestor of basal lower eukaryotic lineages. Construction of phylogenetic trees by taking protein sequences of CMLs revealed presence of eight monophyletic groups (Fig. 8). We named them as group I (red), II (purple), III (olive), IV (green), V (black), VI (fuschia), VII (blue) and VIII (lime) (Fig. 8). The CMLs of lower eukaryotes forms the basal root of the phylogenetic tree. These finding show that CMLs were also evolved from common ancestors of basal lower eukaryotic lineages. Both the results show that, CaMs and CMLs were evolved from their common ancestor. As both the CaMs and CMLs were evolved from a common ancestor and contain four calcium binding EF-hands, it was very important to determine if CaMs and CMLs were coevolved. Therefore, we took the protein sequences of CaMs and CMLs together and constructed a phylogenetic tree (Fig. 9). The phylogenetic tree revealed the presence of six monophyletic groups with CaMs and CMLs analysis (Fig. 9). We named them as group I (red), II (green), III (blue), IV (purple), V (lime) and VI (fuschia) (Fig. 9). The CaMs and CMLs of lower eukaryotic plants forms the basal root of the phylogenetic tree which reflects that both CaM and CMLs were evolved from basal lower eukaryotes together. The monophyletic clades of CaMs and CMLs were shared by each other. In the phylogenetic tree, part of the CML group is dominated (red) (Fig. 9). These finding indicate that these CMLs were evolved recently by duplication and got diversified. The CaMs and CMLs show that they have evolved together from their common ancestors and CMLs were found to be older than CaMs. This is why, during the evolution process, *Eucalyptus grandis* did able to acquire only one CaMs in its genome. The species tree of studied species shows that, the higher plants were evolved from their basal ancestors of lower eukaryotic lineage (Fig. 10). To understand the rate of evolution of CaM and CML, evolutionary rate was studied by estimating gamma parameters for site rates (ML). For CaMs, substitution pattern and rates were estimated under the Jones-Taylor-Thornton model (+G) [1]. A discrete Gamma distribution was used to model evolutionary rate among differences sites (5 categories, [+*G*]). Mean evolutionary rates for CaM in these categories were 0.21, 0.50, 0.81, 1.24, 2.25 substitutions per site. The amino acid frequencies were 7.69% (A), 5.11% (R), 4.25% (N), 5.13% (D), 2.03% (C), 4.11% (Q), 6.18% (E), 7.47% (G), 2.30% (H), 5.26% (I), 9.11% (L), 5.95% (K), 2.34% (M), 4.05% (F), 5.05% (P), 6.82% (S), 5.85% (T), 1.43% (W), 3.23% (Y), and 6.64% (V). The maximum Log likelihood for this computation was −15907.460 and the analysis involved 262 amino acid sequences. There were a total of 139 positions in the final dataset. The mean evolutionary rates for CMLs were 0.18, 0.47, 0.79, 1.24, 2.32 substitutions per site. The amino acid frequencies were 7.69% (A), 5.11% (R), 4.25% (N), 5.13% (D), 2.03% (C), 4.11% (Q), 6.18% (E), 7.47% (G), 2.30% (H), 5.26% (I), 9.11% (L), 5.95% (K), 2.34% (M), 4.05% (F), 5.05% (P), 6.82% (S), 5.85% (T), 1.43% (W), 3.23% (Y), and 6.64% (V). The maximum Log likelihood for this computation was −63710.347. The analysis involved 824 amino acid sequences. There were a total of 116 positions in the final dataset. In both the cases, all positions with less than 95% site coverage were eliminated. That is, fewer than 5% alignment gaps, and missing data, and ambiguous bases were allowed at any position. Result shows that the substitution rates of CaMs are higher than those of CMLs.

### CaM and CMLs are differentially expressed in different tissues

*CaM* and *CMLs* were reportedly involved in diverse cellular process including signaling and different biotic and abiotic stress responses. Different stress responses have varying effects on different parts of the plant. Therefore, tissue specific expressions of the genes also have a large impact in regulating stress conditions. The presence of tissue specific expression data in the phytozome database led us to analyze the expression data of *CaMs* and *CMLs* of *G. max* and *P. vulgaris*. The data revealed that the relative abundance of *GmCaM5* and *GmCaM8-2* were higher in all the studied tissues (pod, nodule, flower, stem, leaves and roots) compared to the rest of *GmCaMs* (Table 4). The maximum abundance of *GmCaM5* was found to be 20.16 in nodules and 18.85 in stems respectibely. Similarly, the maximum abundance of *GmCaM8-2* was found to be 19.62 in leaves and 80.77 in roots respectively followed by *GmCaM9-4* which was also relatively highly expressed in roots (Table 4). The expression of *GmCaM9-1* and *GmCaM9-2* was not found in other tissues except the roots. The expressions of maximum *PvulCaMs* genes of *P. vulgaris* were observed in all tissues except the *PvulCaM9-2*. The abundance of *PvulCaM5* was higher than others in all the tissues. The *PvulCaM7-2* and *PvulCaM7-3* were highly expressed in all tissues, but to a lesser extent than the *PvulCaM5* (Table 4). Although other *PvulCaMs* were expressed in all tissues, their expression level was comparatively lower than that of *PvulCaM5, PvulCaM7-2,* and *PvulCaM7-3*. Overall the result shows that, *CaM5* was highly expressed in all tissues in *G. max* and *P. vulgaris,* while *GmCaM8-2* was highly expressed in roots. Similarly, the *PvulCaM7-2* and *PvulCaM7-3* were ubiquitously expressed in all tissues.

**Table 4:** Tissue specific expression of CaM gene family of *Glycine max* and *Phaseolus vulgaris*. All the expression data are presented as FPKM (Fragments per kilobase of exon per million reads mapped)

Gene	Locus ID	Pods	Nodules	flowers	Stems	leaves	Roots
*Glycine max*							
GmCaM5	Glyma.19G121900	9.41	20.16	11.56	18.85	11.79	15.06
GmCaM8-1	Glyma.02G002100	0	0.641	0.174	0.021	0.873	5.854
GmCaM8-2	Glyma.10G002200	0.09	6.81	3.27	1.53	19.62	85.77
GmCaM8-3	Glyma.10G161900	0.19	0.10	2.35	1.02	0.20	1.58
GmCaM9-1	Glyma.02G143800	0	0	0	0	0	0.292
GmCaM9-2	Glyma.10G030500	0	0	0	0	0	0
GmCaM9-3	Glyma.19G160100	0.21	0.23	0.26	0.46	3.39	3.63
GmCaM9-4	Glyma.03G157800	0.17	0.97	0.13	0.09	5.76	27.48
*Phaseolus vulgaris*							
PvulCaM5	Phvul.006G021800	533.129	266.96	472.30	584.83	259.96	360.75
PvulCaM7-1	Phvul.001G102700	72.09	28.52	79.06	76.92	53.12	50.31
PvulCaM7-2	Phvul.004G076400	196.48	61.87	247.58	163.76	42.72	92.18
PvulCaM7-3	Phvul.008G206000	226.47	268.64	336.004	486.32	295.89	351.73
PvulCaM8-1	Phvul.007G278900	1.26	3.85	4.70	2.44	0.15	4.18
PvulCaM8-2	Phvul.007G187200	2.18	0.17	2.86	7.75	1.16	1.16
PvulCaM8-3	Phvul.006G101200	10.06	2.73	8.80	6.81	2.90	1.39
PvulCaM9-1	Phvul.001G155400	0.69	7.36	2.83	0.88	0.29	16.15
PvulCaM9-2	Phvul.007G175400	0	0	0	0	0	0

Compared to the *CaM, CMLs* were expressed relatively at lower levels in different tissues (Table 5). The *GmCML20* was found to be highly expressed in nodules (51.11) and flowers (54.53), while *GmCML3-3* was highly expressed in pods (34.97), nodules (40.57), stems (157.21), and roots (124.09) (Table 5). The *GmCML5-3* was highly expressed in stems and flowers while *GmCML3-4* and *GmCML3-5* were not expressed in pods, nodules, flowers, or leaves. Similarly, *GmCML15-2* and *GmCML15-3* were not expressed in pods, nodules, flowers, stems, leaves or roots (Table 5). The *GmCML15-1* was found to be slightly expressed in nodules, flowers, stems, leaves and roots whereas *GmCML16* was not expressed in leaves, while it was slightly expressed in other tissues. Additionally, *GmCML25* was also found to be not expressed in flowers while slightly expressed in other tissues. Similarly, the *GmCML20* was not expressed in pods and slightly expressed in other tissues.

**Table 5:** Tissue specific expression of *CML* gene family of *Glycine max* and *Phaseolus vulgaris*. All the expression data are presented as FPKM (Fragments per kilobase of exon per million reads mapped)

Gene	Locus ID	Pods	Nodules	Flowers	Stems	Leaves	Roots
*Glycine max*							
GmCML3-1	Glyma.12G052100	0.63	7.23	0.45	5.81	0.64	6.12
GmCML3-3	Glyma.13G344200	34.97	40.57	26.26	157.21	18.38	124.09
GmCML3-4	Glyma.19G129800	0	0	0	0	0	0.16
GmCML3-5	Glyma.03G127000	0	0	0	0.05	0	0
GmCML5-1	Glyma.17G112000	0.05	0.54	0.07	0.14	6.64	1.10
GmCML5-2	Glyma.13G159600	3.16	1.62	9.69	9.52	11.40	0.61
GmCML5-3	Glyma.15G030100	4.06	37.14	38.28	73.43	2.83	5.86
GmCML11-1	Glyma.19G244300	1.03	0.80	0.50	0.38	0.57	3.83
GmCML11-2	Glyma.03G246800	1.49	0.14	1.57	0.42	0.80	0.26
GmCML11-3	Glyma.20G211700	2.45	17.43	4.65	12.08	4.16	20.00
GmCML11-4	Glyma.10G178400	0.28	4.53	5.63	2.94	10.25	78.66
GmCML15-1	Glyma.05G015500	0	2.93	0.03	0.05	0.05	3.43
GmCML15-2	Glyma.06G208800	0	0	0	0	0	0
GmCML15-3	Glyma.04G144800	0	0	0	0	0	0
GmCML16	Glyma.16G099600	12.54	2.99	16.07	5.37	0	17.45
GmCML17	Glyma.12G089800	0.19	2.08	0.04	2.06	0.99	7.15
GmCML18	Glyma.11G182700	0.21	3.67	0.44	3.66	0.66	0.46
GmCML20	Glyma.15G055100	0	51.11	54.53	60.75	38.74	67.36
GmCML25	Glyma.13G083700	4.05	5.00	0	6.22	26.49	0.64
GmCML27-1	Glyma.14G215800	1.62	5.08	13.66	7.74	9.95	25.79
GmCML27-2	Glyma.02G245700	0.64	3.47	16.90	2.12	16.64	12.21
GmCML27-3	Glyma.07G101100	8.56	8.92	12.50	5.41	3.67	5.42
GmCML27-4	Glyma.18G039500	4.17	8.34	34.59	12.07	8.00	33.48
GmCML27-5	Glyma.08G053500	8.33	4.55	48.03	18.00	51.54	15.43
GmCML30-1	Glyma.02G133000	6.60	5.52	25.35	4.70	3.49	2.81
GmCML30-2	Glyma.17G175400	0.20	1.25	2.17	19.08	13.22	0.83
*Phaseolus vulgaris*							
PvulCML3-1	Phvul.003G168200	1.92	8.23	45.92	12.65	4.09	12.48
PvulCML3-2	Phvul.005G152900	5.06	72.86	30.79	36.36	7.38	38.76
PvulCML3-3	Phvul.011G054100	31.86	39.28	53.71	87.46	8.00	73.65
PvulCML3-4	Phvul.001G122800	0	0	3.58	0	0	0
PvulCML11	Phvul.006G101200	10.06	2.73	8.80	6.81	2.90	1.39
PvulCML15	Phvul.009G201700	0	0	4.71	0	0	0
PvulCML16	Phvul.003G281700	3.13	7.03	2.25	3.21	2.00	4.92
PvulCML18	Phvul.003G283800	0.69	2.62	2.56	3.90	1.88	3.85
PvulCML20	Phvul.006G204800	33.01	35.89	73.85	48.62	27.89	37.04
PvulCML25-1	Phvul.010G085100	7.59	0.11	6.77	0.03	0	0.26
PvulCML25-2	Phvul.003G019600	0.03	0.72	12.63	0.07	0	0.20
PvulCML25-3	Phvul.008G167700	34.20	1.76	96.27	38.21	21.46	7.84
PvulCML25-4	Phvul.002G320800	0.85	0.25	8.92	0.68	0	0.35
PvulCML27-1	Phvul.008G235100	16.14	17.75	9.71	7.49	5.36	31.28
PvulCML27-2	Phvul.001G231000	21.50	6.81	47.32	67.12	49.45	16.92
PvulCML27-3	Phvul.L002000	8.52	65.15	12.28	37.28	15.86	21.65
PvulCML27-4	Phvul.002G329300	30.35	4.86	65.89	17.17	85.82	9.74
PvulCML30-1	Phvul.003G292700	6.15	2.62	41.58	8.96	5.67	6.08
PvulCML30-2	Phvul.008G031800	20.44	2.81	77.19	16.57	6.71	8.61
PvulCML30-3	Phvul.002G019300	14.53	4.23	189.8	7.90	33.04	9.00
PvulCML38-1	Phvul.005G026100	8.82	15.51	2.06	0.21	0	34.39
PvulCML38-2	Phvul.005G026000	4.67	6.16	1.73	0.24	0	9.40
PvulCML38-3	Phvul.003G210000	74.90	16.56	2.41	0.96	0.37	38.55
PvulCML38-4	Phvul.004G055200	0.02	2.30	0.22	0.47	0	3.15
PvulCML38-5	Phvul.001G095600	0.20	3.99	4.01	0	0.19	3.86
PvulCML41	Phvul.003G251000	13.23	183.22	42.30	32.82	3.14	24.24

When compared to *PvulCaMs, PvulCMLs* were also expressed at relatively lower levels. The *PvulCML3-3* was ubiquitously expressed in pods (31.86), nodules (39.28), flowers (53.71), stems (87.46), leaves (8), and roots (73.65) (Table 5) while *PvulCML3-2* was found to be expressed significantly higher in nodules (72.86), flowers (30.79), stems (36.36), leaves (7.38) and roots (38.76), but expressed to a lesser extent than that of *PvulCML3-1*. The *PvulCML38-3* was highly expressed in pods (74.9) and roots (38.55) followed by expression of *PvulCML25-3* in pods (34.2), flowers (96.27), stems (38.21), and roots (38.55) (Table 5). The *PvulCML20* was highly expressed in pods (33.01), nodules (35.89), flowers (73.85), stems (48.62), leaves (27.89) and roots (37.04) while *PvulCML3-4* was not expressed in pods, nodules, stems, leaves and roots but it was relatively highly expressed in flowers (3.58) (Table 5). Similarly, *PvulCML15* was not expressed in pods, nodules, stems, leaves and roots while relatively highly expressed in flowers (4.71). Investigations of the expression of *G. max* and *P. vulgaris CMLs* revealed that, *CML3* and *CML 20* were expressed in all tissues in both the plants, while *CML3-4*, and *CML15* (*CML15-2* and *CML15-3* in the case of *G. max*) were not expressed in any of the plants.

## Conclusion

The *CaM* and *CML* gene family from 41 plant species were studied. Study shows the presence of four calcium binding D-x-D motifs in CaM and one D-x-D-x-D motif in CMLs. The number of family members of *CaM* and *CMLs* gene family vary significantly and do not correlate to the genome size of the organism. The evolutionary study shows, CMLs were evolved earlier than CaMs and diversified later. Tissue specific expression of *CaM* and *CML* shows, these genes plays important role in development of different tissues in *G. max* and *P. vulgaris*.

## Methods

### Identification of CaM and CML gene family

The calmodulin and calmodulin-like genes of *Arabidopsis thaliana* and *Oryza sativa* were downloaded from the “Arabidopsis Information Resource” database [21] and “Rice Genome Annotation Project” respectively [22]. The protein sequences of CaM and CMLs of *A. thaliana* and *O. sativa* were used as the query sequences in the publicly available phytozome databases to identify the protein sequences of CaM and CMLs of other plant species using BLASTP program [23]. The *CaM* and *CML* genes of *Picea abies* were downloaded from the spruce genome project [24]. The protein sequences of CaM and CML were used to identify the *CaM* and *CML* gene family in other plant species. Overall, 41 plant species were considered during the study (Table 1). The statistical parameters used during BLASTP searches were target type, proteome; expect (E) threshold, (−1); and comparison matrix, BLOSUM62. Sequences recovered from the BLASTP searches were collected for further analysis. Later, all the collected sequences of BLAST results were evaluated using the scanprosite software to confirm the presence of the prosite calcium binding EF-hands domain. The sequences those showed the presence of four calcium binding EF-hands domains were considered as CaM or CML proteins. Later, all sequences were subjected to the BLASTP analysis in the *A. thaliana* (TAIR) and *O. sativa proteome* (rice genome annotation project) database. The sequences that resulted in BLASTP hits of the *CaM* gene in both the database were considered as CaM protein while that resulted in BLASTP hits to the CMLs were considered as CML proteins.

Subsequently we named all the CaM and CML proteins of the studied plant species. Nomenclature was conducted according to the orthologous based nomenclature system as proposed earlier [8, 25]. Name were given by taking the first letter of the genus name in upper case and the first letter of the species (sometimes 2 to3 letters were used when redundancy was observed) name in the lower case followed by the number corresponding to the orthologs genes of *A. thaliana* or *O. sativa*. Monocot plant species were named according to the orthologous genes of *O. sativa* while dicot and other species were named according to the orthologous genes of *A. thaliana* as proposed earlier [8, 25, 26].

### Molecular modeling of CaM and CML

Molecular modeling was conducted to evaluate the molecular details of CaM and CML proteins. The Geno3d software [27] was used to construct the molecular structure of CaM and CMLs. The protein sequence of AtCaM1 and AtCML1 was utilized as the query sequence to search the model. Following statistical parameters were used to run the analysis: database, non-redundant protein sequences; filter query sequence (−F), true; expectation value (−e, real), 10.0; number of on-line descriptions (−v, int), 500; number of alignments to show (−b, int), 500; matrix (−M), BLOSUM62; expectation value threshold for inclusion in multipass model (−h, real), 0.002; maximum number of passes to use in multipass version (−j, int), 3.

### Multiple sequence alignment

Multiple sequence alignment of CaM and CML proteins was conducted separately to investigate the presence of conserved domains and motifs. Multalin software was used to run the multiple sequence alignment. The statistical parameters used during multiple sequence alignments were, sequence input format, Multalin-fasta; protein weight matrix, BLSOUM62-12-2; gap penalty at opening, default; gap penalty at extension, default; gap penalties at extremities, none; one iteration only, no; high consensus level, 90%; low consensus level 50%.

### Palmitoylation site prediction

The palmitoylation sites of CaMs and CMLs protein were predicted using the CSS palm software version 2.0 [28]. During the prediction, input sequences were submitted in FASTA format and the threshold was set to higher or medium.

### Phylogenetic tree

The phylogenetic trees were constructed to understand the evolution of CaM and CMLs. To construct the phylogenetic tree of CaMs and CMLs, protein sequences were subjected to clustalW or clustal omega software to generate a clustal file [29]. The generated clustal files were then converted to MEGA file format using the MEGA6 software [30]. The generated MEGA files of CaMs and CMLs were used to construct the phylogenetic trees. Different statistical parameters used to construct the phylogenetic tree were as follows: analysis, phylogeny reconstruction; statistical method, maximum likelihood; test of phylogeny, bootstrap method; no. of bootstrap replicates, 1000; substitution type, amino acid; model/method, Jones-Taylor-Thornton (JTT) model; gaps/missing data treatment, partial deletion; site coverage cutoff (%), 95; ML heuristic method, nearest-neighbor-interchange (NNI); and branch swap filter, very strong. The gamma parameter for site rates was estimated using MEGA 6 software. Following parameters were used to study the site rate: analysis, estimate rate variation among sites (ML); statistical method, maximum likelihood; substitution type, amino acid; model/method, Jones-Taylor-Thornton (JTT); rates among sites, gamma distributed (G); number of discrete gamma categories, 5; gaps/missing data treatment, partial deletion; site coverage cutoff (%), very strong. The species tree was built using NCBI taxonomy browser (http://www.ncbi.nlm.nih.gov/Taxonomy/CommonTree/wwwcmt.cgi).

### Tissue specific expression of CaMs and CMLs

Understanding the tissue specific expression of any particular gene is important to elucidating its role in growth, development and stress responses. Therefore, we studied the tissue specific expression of *CaM* and *CML* genes of *G. max* and *P. vulgaris*. The expression profiles of *CaMs* and *CMLs* were searched in the phytomine database of phytozome. The expression profiles of all of the genes are represented as FPK (fragments per kilo base of exon per million reads mapped).

### Statistical analysis

Regression analysis was conducted to evaluate the correlation of *CaM* and *CML* gene family size with regard to the genome size. Mathportal (http://www.mathportal.org/calculators/statistics-calculator/correlation-and-regression-calculator.php) was used for the correlation regression analyses.

## Additional files

Additional file 1: Table S1. Calmodulin (*CaM*) gene family members of monocot, dicot and lower eukaryotic plant lineages. Table shows gene name, locus ID, open reading frame (ORF), number of introns and 5'-3' coordinates of *CaM* genes. (DOC 428 kb)
Additional file 2: Table S2. Calmodulin-like (*CML*) gene family members of monocot, dicot and lower eukaryotic plant lineages. Table shows gene name, locus ID, open reading frame (ORF), number of introns and 5'-3' coordinates of *CML* genes. (DOC 1223 kb)
Additional file 3: Figure S1. Multiple sequence alignment of CaM protein of plant. Alignment shows presence of conserved motifs in EF-hand domain. (PDF 817 kb)
Additional file 4: Figure S2. Multiple sequence alignment of CML protein of plant. Alignment shows presence of conserved motifs in EF-hand domain. (PDF 441 kb)

## Abbreviations

CaM: Calmodulin
CBL: Calcineurin B-like
CDPK: Calcium dependent protein kinase
CML: Calmodulin-like
CRK: CDPK Related kinase
EF: Elongation factor
