# Supplementary material for: Genomics and evolutionary aspect of calcium signaling event in calmodulin and calmodulin-like proteins in plants
Source: BMC Plant Biol. 2017 Feb 3;17:38. doi: 10.1186/s12870-017-0989-3 (PMC5291997; doi:10.1186/s12870-017-0989-3)
Supplement: Additional file 1: Table S1. — Calmodulin (CaM) gene family members of monocot, dicot and lower eukaryotic plant lineages. Table shows gene name, locus ID, open reading frame (ORF), number of introns and 5'-3' coordinates of CaM genes. (DOC 428 kb) [file 12870_2017_989_MOESM1_ESM.doc]

Additional file 1: Table S1: Calmodulin (*CaM*) gene family members of monocot, dicot and lower eukaryotic plant lineages. Table shows gene name, locus ID, open reading frame (ORF), number of introns and 5'-3' coordinates of *CaM* genes.

|  |  | | | |  | |  |  |  | |
| --- | --- | --- | --- | --- | --- | --- | --- | --- | --- | --- |
| **Gene Name** | **Locus ID** | | | | **ORF** | | **No. of amino acids** | **No. of Introns** | **5'-3' Coordinate** | |
| *Aguilegia coerulea* | | | | | | | | | | |
| AcCaM5-1 | Aquca_009_00099 | | | | 555 | | 184 | 2 | | scaffold_9: 586745 - 590996 |
| AcCaM5-2 | Aquca_014_00917 | | | | 450 | | 149 | 1 | | scaffold_14: 5630453 - 5632701 |
| AcCaM5-3 | Aquca_014_00916 | | | | 402 | | 133 | 1 | | scaffold_14: 5619649 - 5620407 |
| AcCaM6 | Aquca_002_01201 | | | | 450 | | 149 | 3 | | scaffold_2: 8885647 - 8889386 |
| AcCaM8 | Aquca_049_00129 | | | | 453 | | 150 | 3 | | scaffold_49: 1099172 - 1100620 |
| *Arabidopsis thaliana* | | | | | | | | | | |
| AtCaM1 | [At5g37780](http://www.arabidopsis.org/servlets/TairObject?type=locus&name=AT5G37780) | | | | 528 | | 175 | 1 | Chr5: 15004527 - 15006205 | |
| AtCaM2 | [At2g41110](http://www.arabidopsis.org/servlets/TairObject?type=locus&name=AT2G41110) | | | | 486 | | 161 | 1 | Chr2: 17140229 - 17141393 | |
| AtCaM3 | [At3g56800](http://www.arabidopsis.org/servlets/TairObject?type=locus&name=AT3G56800) | | | | 450 | | 149 | 1 | Chr3: 21034537 - 21036077 | |
| AtCaM4 | [At1g66410](http://www.arabidopsis.org/servlets/TairObject?type=locus&name=AT1G66410) | | | | 480 | | 159 | 1 | Chr1: 24774260 - 24775984 | |
| AtCaM5 | [At2g27030](http://www.arabidopsis.org/servlets/TairObject?type=locus&name=AT2G27030) | | | | 546 | | 181 | 1 | Chr2: 11532004 - 11534333 | |
| AtCaM6 | [At5g21274](http://www.arabidopsis.org/servlets/TairObject?type=locus&name=AT5G21274) | | | | 450 | | 149 | 1 | Chr5: 7214500 - 7216019 | |
| AtCaM7 | [At3g43810](http://www.arabidopsis.org/servlets/TairObject?type=locus&name=AT3G43810) | | | | 450 | | 149 | 1 | Chr3: 15664371 - 15666460 | |
| AtCaM8 | [At4g14640](http://www.arabidopsis.org/servlets/TairObject?type=locus&name=AT4G14640) | | | | 456 | | 151 | 3 | Chr4: 8397712 - 8400076 | |
| AtCaM9 | [At3g51920](http://www.arabidopsis.org/servlets/TairObject?type=locus&name=AT3G51920) | | | | 456 | | 151 | 3 | Chr3: 19268043 - 19269479 | |
| *Brachypodium distachyon* | | | | | | | | | | |
| BdCaM1-1 | Bradi1g17236 | | | | 450 | | 149 | 1 | Bd1: 13811143 - 13812767 | |
| BdCaM1-2 | Bradi1g64020 | | | | 450 | | 149 | 1 | Bd1: 63222811 - 63225165 | |
| BdCaM1-3 | Bradi2g21460 | | | | 450 | | 149 | 1 | Bd2: 18961624 - 18964400 | |
| BdCaM2 | Bradi2g10010 | | | | 450 | | 149 | 1 | Bd2: 8192560 - 8196245 | |
| BdCaM3 | Bradi2g10790 | | | | 450 | | 149 | 0 | Bd2: 9010480 - 9013295 | |
| *Brassica rapa* | | | | | | | | | | |
| BrCaM1-1 | Brara.G02648 | | | | 450 | | 149 | 1 | A07:21702931..21704112 | |
| BrCaM1-2 | Brara.D01190 | | | | 450 | | 149 | 1 | A04:10969850..10970993 | |
| BrCaM5-1 | Brara.D01606 | | | | 543 | | 180 | 2 | A04:14181424..14183197 | |
| BrCaM5-2 | Brara.C02103 | | | | 450 | | 149 | 1 | A03:10491309..10492159 | |
| BrCaM5-3 | Brara.G01708 | | | | 450 | | 149 | 1 | A07:16492622..16494092 | |
| BrCaM5-4 | Brara.I03906 | | | | 450 | | 149 | 1 | A09:35644800..35645914 | |
| BrCaM5-5 | Brara.D00328 | | | | 450 | | 149 | 1 | A04:2322900..2324478 | |
| BrCaM5-6 | Brara.J01511 | | | | 450 | | 149 | 1 | A10:13219180..13220322 | |
| BrCaM5-7 | Brara.C00973 | | | | 450 | | 149 | 1 | A03:4570068..4571241 | |
| BrCaM5-8 | Brara.B00936 | | | | 450 | | 149 | 1 | A02:4431851..4432908 | |
| BrCaM5-9 | Brara.F01927 | | | | 630 | | 209 | 2 | A06:14143466..14145950 | |
| BrCaM5-10 | Brara.G01295 | | | | 450 | | 149 | 1 | A07:13645782..13646627 | |
| BrCaM5-11 | Brara.F01882 | | | | 444 | | 147 | 2 | A06:12950836..12951842 | |
| *Capsella rubella* | | | | | | | | | | |
| CrCaM1-1 | Carubv10021106m.g | | | | 450 | | 149 | 1 | scaffold_2: 7946754 - 7948512 | |
| CrCaM1-2 | Carubv10005958m.g | | | | 450 | | 149 | 1 | scaffold_7: 14968510 - 14970352 | |
| CrCaM2 | Carubv10024180m.g | | | | 516 | | 171 | 2 | scaffold_4: 12286815 - 12289482 | |
| CrCaM3 | Carubv10024013m.g | | | | 675 | | 224 | 2 | scaffold_4: 5681316 - 5684055 | |
| CrCaM5 | Carubv10018192m.g | | | | 450 | | 149 | 1 | scaffold_5: 11275198 - 11276747 | |
| CrCaM6 | Carubv10010474m.g | | | | 513 | | 170 | 0 | scaffold_1: 6285614 - 6286433 | |
| CrCaM7-1 | Carubv10019058m.g | | | | 648 | | 215 | 2 | scaffold_5: 5733314 - 5735877 | |
| CrCaM7-2 | Carubv10002202m.g | | | | 450 | | 149 | 11 | scaffold_6: 7234584 - 7241598 | |
| CrCaM8 | Carubv10006432m.g | | | | 456 | | 151 | 3 | scaffold_7: 10514644 - 10516346 | |
| CrCaM9 | Carubv10018188m.g | | | | 456 | | 151 | 3 | scaffold_5: 9360180 - 9361769 | |
| *Carica papaya* | | | | | | | | | | |
| CpCaM5-1 | | evm.TU.supercontig_33.178 | | | 450 | | 149 | 1 | supercontig_33:1798360..1800175 | |
| CpCaM5-2 | | evm.TU.supercontig_33.179 | | | 450 | | 149 | 1 | supercontig_33:1802299..1803640 | |
| CpCaM6 | | evm.TU.supercontig_1473.1 | | | 543 | | 180 | 4 | supercontig_1473: 4003 - 17910 | |
| CpCaM7 | | evm.TU.supercontig_34.217 | | | 450 | | 149 | 1 | supercontig_34: 2026376 - 2027875 | |
| CpCaM8 | | evm.TU.supercontig_117.85 | | | 453 | | 150 | 3 | supercontig_117: 869363 - 870353 | |
| *Chlamydomonas reinhardtii* | | | | | | | | | | |
| CreinCaM1 | | Cre03.g178350 | | | 474 | | 157 | 6 | Chr3: 4780759 - 4783824 | |
| CreinCaM3 | | Cre12.g527450 | | | 597 | | 198 | 8 | Chr12: 5169560 - 5172352 | |
| CreinCaM5 | | Cre14.g617550 | | | 555 | | 184 | 0 | Chr14: 1403594 - 1405257 | |
| CreinCaM6-1 | | Cre03.g150300 | | | 438 | | 145 | 3 | Chr3: 1290492 - 1292539 | |
| CreinCaM6-2 | | Cre03.g178150 | | | 492 | | 163 | 5 | Chr3: 4764485 - 4766557 | |
| CreinCaM8 | | Cre11.g468450 | | | 510 | | 169 | 6 | Chr11: 2036432 - 2038737 | |
| *Citrus clementina* | | | | | | | | | | |
| CcCaM3 | Ciclev10005743m.g | | | | 726 | | 241 | 4 | scaffold_9: 15114173 - 15116741 | |
| CcCaM4 | Ciclev10002774m.g | | | | 453 | | 150 | 3 | scaffold_5: 29580438 - 29581464 | |
| CcCaM5 | Ciclev10003695m.g | | | | 453 | | 150 | 3 | scaffold_5: 29574924 - 29576567 | |
| CcCaM6-1 | Ciclev10013030m.g | | | | 450 | | 149 | 1 | scaffold_6: 21563536 - 21565310 | |
| CcCaM6-2 | Ciclev10022698m.g | | | | 450 | | 149 | 3 | scaffold_3: 44031714 - 44032693 | |
| CcCaM6-3 | Ciclev10013029m.g | | | | 450 | | 149 | 1 | scaffold_6: 21559346 - 21562036 | |
| CcCaM6-4 | Ciclev10016866m.g | | | | 552 | | 183 | 3 | scaffold_2: 8494369 - 8498965 | |
| CcCaM8 | Ciclev10006151m.g | | | | 453 | | 150 | 3 | scaffold_9: 4266408 - 4267837 | |
| *Citrus sinensis* | | | | | | | | | | |
| CsCaM4 | orange1.1g040628m.g | | | | 375 | | 124 | 2 | scaffold00255: 218481 - 219136 | |
| CsCaM5-1 | orange1.1g032008m.g | | | | 450 | | 149 | 3 | scaffold00022: 563932 - 565053 | |
| CsCaM5-2 | orange1.1g032375m.g | | | | 429 | | 142 | 0 | scaffold00158:134776..135605 | |
| CsCaM7-1 | orange1.1g044644m.g | | | | 552 | | 183 | 2 | scaffold00028: 876163 - 879487 | |
| CsCaM7-2 | orange1.1g031534m.g | | | | 477 | | 158 | 2 | scaffold00091: 230103 - 236254 | |
| CsCaM8 | orange1.1g031903m.g | | | | 453 | | 150 | 3 | scaffold00132: 352651 - 354037 | |
| *Coccomyxa subellipsoidea* | | | | | | | | | | |
| CsubCaM4 | | estExt_fgenesh1_pg.C_200007 | | | 513 | | 170 | 5 | scaffold_20: 54335 - 56861 | |
| CsubCaM5-1 | | estExt_Genewise1.C_70390 | | | 456 | | 151 | 3 | scaffold_7: 2177784 - 2179198 | |
| CsubCaM5-2 | | fgenesh1_pm.1_#_105 | | | 555 | | 184 | 2 | scaffold_1: 1511852 - 1512625 | |
| *Cuccumis sativus* | | | | | | | | | | |
| CsatCaM5-1 | | | | Cucsa.142500 | 450 | | 149 | 1 | scaffold01079:765938..768048 | |
| CsatCaM5-2 | | | | Cucsa.018090 | 450 | | 149 | 1 | scaffold00252:367512..369370 | |
| CsatCaM8-1 | | | | Cucsa.016450 | 450 | | 149 | 3 | scaffold00227: 145820 - 147841 | |
| CsatCaM8-2 | | | | Cucsa.386760 | 489 | | 162 | 3 | scaffold03918: 303881 - 304986 | |
| CsatCaM8-3 | | | | Cucsa.254730 | 453 | | 150 | 3 | scaffold02229: 2966599 - 2968198 | |
| CsatCaM9 | | | | Cucsa.133320 | 441 | | 146 | 3 | scaffold01037: 1041161 - 1043802 | |
| *Fragaria vesca* | | | | | | | | | | |
| FvCaM3 | | | gene09328-v1.0-hybrid | | 450 | | 149 | 3 | LG5: 9755368 - 9757357 | |
| FvCaM7-1 | | | gene29325-v1.0-hybrid | | 537 | | 178 | 0 | LG5: 19283420 - 19283956 | |
| FvCaM7-2 | | | gene30517-v1.0-hybrid | | 450 | | 149 | 1 | LG1: 9651053 - 9652367 | |
| FvCaM8-1 | | | gene20270-v1.0-hybrid | | 1008 | | 335 | 4 | LG2: 14569384 - 14571586 | |
| FvCaM8-2 | | | gene29038-v1.0-hybrid | | 780 | | 259 | 6 | LG4: 920961 - 924227 | |
| *Glycine max* | | | | | | | | | | |
| GmCaM5 | | | Glyma.19G121900 | | 450 | | 149 | 1 | Gm19: 37805394 - 37807412 | |
| GmCaM8-1 | | | Glyma.02G002100 | | 453 | | 150 | 3 | Gm02: 247329 - 250238 | |
| GmCaM8-2 | | | Glyma.10G002200 | | 453 | | 150 | 3 | Gm10: 248104 - 250721 | |
| GmCaM8-3 | | | Glyma.10G161900 | | 450 | | 149 | 3 | Gm10: 39055435 - 39056840 | |
| GmCaM9-1 | | | Glyma.02G143800 | | 450 | | 149 | 3 | Gm02: 14630203 - 14631393 | |
| GmCaM9-2 | | | Glyma.10G030500 | | 450 | | 149 | 3 | Gm10: 2644392 - 2645552 | |
| GmCaM9-3 | | | Glyma.19G160100 | | 447 | | 148 | 3 | Gm19: 41900372 - 41901513 | |
| GmCaM9-4 | | | Glyma.03G157800 | | 447 | | 148 | 3 | Gm03: 39344097 - 39346448 | |
| *Gossypium raimondii* | | | | | | | | | | |
| GrCaM3 | | | Gorai.008G292900 | | 453 | | 150 | 3 | Chr08: 56668980 - 56671010 | |
| GrCaM5-1 | | | Gorai.001G128100 | | 450 | | 149 | 1 | Chr06: 36827583 - 36830275 | |
| GrCaM5-2 | | | Gorai.013G256400 | | 450 | | 149 | 1 | Chr13:57253722..57256035 | |
| GrCaM8-1 | | | Gorai.005G046000 | | 453 | | 150 | 3 | Chr05: 4365518 - 4367929 | |
| GrCaM8-2 | | | Gorai.011G166700 | | 453 | | 150 | 3 | Chr11: 33253294 - 33255566 | |
| GrCaM9 | | | Gorai.004G076400 | | 465 | | 154 | 3 | Chr04: 8741315 - 8742521 | |
| *Linum usitatissimum* | | | | | | | | | | |
| LuCaM6-1 | | | Lus10009176.g | | 486 | | 161 | 1 | scaffold471: 33573 - 34149 | |
| LuCaM6-2 | | | Lus10001775.g | | 465 | | 154 | 2 | scaffold1230: 20022 - 21929 | |
| LuCaM6-3 | | | Lus10027283.g | | 450 | | 149 | 1 | scaffold472: 624425 - 626113 | |
| LuCaM7-1 | | | Lus10041288.g | | 450 | | 149 | 1 | scaffold280: 1791274 - 1792598 | |
| LuCaM7-2 | | | Lus10038981.g | | 450 | | 149 | 1 | scaffold34: 1114621 - 1116298 | |
| LuCaM7-3 | | | Lus10037423.g | | 450 | | 149 | 1 | scaffold462: 1766342 - 1767682 | |
| LuCaM7-4 | | | Lus10022589.g | | 498 | | 165 | 1 | scaffold59:60279..60982 | |
| LuCaM9-1 | | | Lus10024830.g | | 468 | | 155 | 3 | scaffold473: 271596 - 272738 | |
| LuCaM9-2 | | | Lus10014708.g | | 444 | | 147 | 2 | scaffold584: 306810 - 307443 | |
| LuCaM9-3 | | | Lus10004088.g | | 441 | | 146 | 2 | scaffold115: 174350 - 174987 | |
| LuCaM9-4 | | | Lus10014709.g | | 444 | | 147 | 2 | scaffold584: 308949 - 309582 | |
| *Malus domestica* | | | | | | | | | | |
| MdCaM2 | | | MDP0000277474 | | 450 | | 149 | 3 | MDC003936.406: 14490 - 15933 | |
| MdCaM3 | | | MDP0000208420 | | 498 | | 165 | 3 | MDC017021.252: 21324 - 22777 | |
| MdCaM4-1 | | | MDP0000234624 | | 447 | | 148 | 3 | MDC005635.301: 619 - 1589 | |
| MdCaM4-2 | | | MDP0000295068 | | 447 | | 148 | 3 | MDC011897.309: 6835 - 7805 | |
| MdCaM6-1 | | | MDP0000865414 | | 450 | | 149 | 1 | MDC003294.519:8465..9901 | |
| MdCaM6-2 | | | MDP0000361567 | | 534 | | 179 | 1 | MDC021801.378:725..2119 | |
| MdCaM7 | | | MDP0000183898 | | 450 | | 149 | 1 | MDC006442.550:9959..11358 | |
| MdCaM8-1 | | | MDP0000203128 | | 447 | | 148 | 3 | MDC013321.85: 5797 - 6750 | |
| MdCaM8-2 | | | MDP0000214380 | | 447 | | 148 | 3 | MDC018173.205: 4840 - 5805 | |
| *Manihot esculenta* | | | | | | | | | | |
| MeCaM5-1 | | | cassava4.1_018395m.g | | 450 | | 149 | 1 | scaffold09761: 823493 - 824967 | |
| MeCaM5-2 | | | cassava4.1_018409m.g | | 450 | | 149 | 1 | scaffold01045: 162759 - 164667 | |
| MeCaM5-3 | | | cassava4.1_018417m.g | | 450 | | 149 | 1 | scaffold04443: 214307 - 216710 | |
| MeCaM5-4 | | | cassava4.1_017016m.g | | 570 | | 189 | 2 | scaffold04209: 689232 - 693591 | |
| MeCaM7 | | | cassava4.1_018413m.g | | 450 | | 149 | 1 | scaffold05703: 100680 - 102495 | |
| MeCaM8-1 | | | cassava4.1_030730m.g | | 453 | | 150 | 3 | scaffold03802: 637490 - 638502 | |
| MeCaM8-2 | | | cassava4.1_020944m.g | | 450 | | 149 | 3 | scaffold03614: 2900593 - 2901271 | |
| MeCaM9-1 | | | cassava4.1_024201m.g | | 447 | | 148 | 3 | scaffold11998: 644356 - 645044 | |
| MeCaM9-2 | | | cassava4.1_029517m.g | | 447 | | 148 | 3 | scaffold09347: 42192 - 42887 | |
| *Medicago truncatula* | | | | | | | | | | |
| MtCaM1 | | | Medtr7g087610 | | 450 | | 149 | 1 | chr7: 26854798 - 26856288 | |
| MtCaM7 | | | Medtr5g088320 | | 450 | | 149 | 1 | chr5: 37277966 - 37281091 | |
| MtCaM8 | | | Medtr7g034850 | | 453 | | 150 | 3 | chr7: 10020030 - 10021112 | |
| MtCaM9 | | | Medtr1g071150 | | 486 | | 161 | 3 | chr1: 17460161 - 17461255 | |
| *Micromonas pusila* | | | | | | | | | | |
| MpCaM1 | | | fgenesh1_pm.14_#_119 | | | 486 | 161 | 0 | scaffold_14: 673066 - 673551 | |
| MpCaM5-1 | | | MicpuC2.estExt_fgenesh1_kg.C_20005 | | | 528 | 175 | 5 | scaffold_2: 1323508 - 1324870 | |
| MpCaM5-2 | | | MicpuC2.estExt_fgenesh1_kg.C_20061 | | | 450 | 149 | 0 | scaffold_2: 499927 - 500722 | |
| MpCaM5-3 | | | estExt_Genemark1.C_9_t10062 | | | 594 | 197 | 1 | scaffold_9: 117466 - 118503 | |
| MpCaM5-4 | | | MicpuC2.EuGene.0000170071 | | | 714 | 237 | 1 | scaffold_17: 134057 - 134843 | |
| *Mimulus guttatus* | | | | | | | | | | |
| MgCaM4 | | | mgv1a023810m.g | | 441 | | 146 | 3 | scaffold_6: 3937391 - 3941030 | |
| MgCaM5-1 | | | mgv1a015696m.g | | 450 | | 149 | 1 | scaffold_2: 291350 - 293379 | |
| MgCaM5-2 | | | mgv1a015681m.g | | 450 | | 149 | 1 | scaffold_1: 1528596 - 1530423 | |
| MgCaM5-3 | | | mgv1a015694m.g | | 450 | | 149 | 1 | scaffold_75: 790785 - 792231 | |
| MgCaM5-4 | | | mgv1a015677m.g | | 450 | | 149 | 1 | scaffold_204: 232084 - 234046 | |
| MgCaM5-5 | | | mgv1a019086m.g | | 456 | | 151 | 3 | scaffold_416: 40972 - 42462 | |
| MgCaM6 | | | mgv1a015693m.g | | 450 | | 149 | 1 | scaffold_204: 228246 - 230591 | |
| MgCaM7 | | | mgv1a015687m.g | | 450 | | 149 | 3 | scaffold_54: 198674 - 201734 | |
| MgCaM8-1 | | | mgv1a019170m.g | | 426 | | 141 | 3 | scaffold_6: 3928488 - 3930058 | |
| MgCaM8-2 | | | mgv1a021942m.g | | 456 | | 151 | 3 | scaffold_130: 106392 - 107611 | |
| MgCaM8-3 | | | mgv1a023732m.g | | 453 | | 150 | 3 | scaffold_6: 3921568 - 3922617 | |
| MgCaM9-1 | | | mgv1a021796m.g | | 453 | | 150 | 3 | scaffold_1: 3314404 - 3315197 | |
| MgCaM9-2 | | | mgv1a018394m.g | | 381 | | 126 | 2 | scaffold_140: 411088 - 412644 | |
| *Oryza sativa* | | | | | | | | | | |
| OsCaM1-1 | | | LOC_Os03g20370 | | 450 | | 149 | 1 | Chr3: 11522674 - 11524473 | |
| OsCaM1-2 | | | LOC_Os07g48780 | | 450 | | 149 | 1 | Chr7: 29200392 - 29197727 | |
| OsCaM1-3 | | | LOC_Os01g16240 | | 450 | | 149 | 1 | Chr1: 9198962 - 9195527 | |
| OsCaM2 | | | LOC_Os05g41210 | | 450 | | 149 | 1 | Chr5: 24134899 - 24132441 | |
| OsCaM3 | | | LOC_Os01g17190 | | 450 | | 149 | 1 | Chr1: 9887626 - 9889328 | |
| *Ostreococcus lucimarinus* | | | | | | | | | | |
| OlCaM1 | | | e_gwEuk.17.206.1 | | 492 | | 163 | 0 | Chr_17:148977..149468 | |
| OlCaM5 | | | e_gwEuk.1.768.1 | | 450 | | 149 | 0 | Chr_1:699084..699533 | |
| *Panicum hallii* | | | | | | | | | | |
| PhCaM1-2 | | | Pahal.C02522 | | 450 | | 149 | 1 | Chr_03:16239413..16241769 | |
| PhCaM1-3 | | | Pahal.E03401 | | 450 | | 149 | 1 | Chr_05:41051661..41055572 | |
| PhCaM1-4 | | | Pahal.D02442 | | 450 | | 149 | 1 | Chr_04:45637714..45640023 | |
| PhCaM1-5 | | | Pahal.B05011 | | 450 | | 149 | 1 | Chr_02:68790341..68792678 | |
| PhCaM3 | | | Pahal.E01222 | | 549 | | 182 | 2 | Chr_05:7203464..7206238 | |
| *Panicum virgatum* | | | | | | | | | | |
| PvCaM1-2 | | | Pavir.Ea01136 | | 681 | | 226 | 1 | Chr05a:15392368..15396578 | |
| PvCaM1-3 | | | Pavir.Ia03465 | | 450 | | 149 | 1 | Chr09a:68904334..68906701 | |
| PvCaM1-4 | | | Pavir.Ba00296 | | 450 | | 149 | 1 | Chr02a:3730111..3732012 | |
| PvCaM1-5 | | | Pavir.Eb01106 | | 450 | | 149 | 1 | Chr05b:16763152..16766850 | |
| PvCaM1-6 | | | Pavir.Ib01490 | | 450 | | 149 | 1 | Chr09b:16406808..16409188 | |
| PvCaM1-7 | | | Pavir.J04355 | | 450 | | 149 | 1 | contig06024:6388..8835 | |
| PvCaM1-8 | | | Pavir.J05308 | | 450 | | 149 | 1 | contig08045:8611..11040 | |
| PvCaM1-9 | | | Pavir.Bb03712 | | 1317 | | 438 | 7 | Chr02b:73860296..73864809 | |
| PvCaM1-10 | | | Pavir.Eb03514 | | 549 | | 182 | 2 | Chr05b:70268479..70271053 | |
| *Phaseolus vulgaris* | | | | | | | | | | |
| PvulCaM5 | | | Phvul.006G021800 | | 450 | | 149 | 1 | Chr06:10187482..10189726 | |
| PvulCaM7-1 | | | Phvul.001G102700 | | 450 | | 149 | 1 | Chr01:23671381..23672973 | |
| PvulCaM7-2 | | | Phvul.004G076400 | | 450 | | 149 | 1 | Chr04:12769531..12771291 | |
| PvulCaM7-3 | | | Phvul.008G206000 | | 450 | | 149 | 1 | Chr08:51723327..51725429 | |
| PvulCaM8-1 | | | Phvul.007G278900 | | 453 | | 150 | 3 | Chr07:51560570..51562809 | |
| PvulCaM8-2 | | | Phvul.007G187200 | | 450 | | 149 | 3 | Chr07:42358585..42359713 | |
| PvulCaM8-3 | | | Phvul.006G101200 | | 450 | | 149 | 3 | Chr06:21833976..21835032 | |
| PvulCaM9-1 | | | Phvul.001G155400 | | 447 | | 148 | 3 | Chr01:41292521..41293336 | |
| PvulCaM9-2 | | | Phvul.007G175400 | | 447 | | 148 | 3 | Chr07:41073402..41074222 | |
| *Physcomitrella patens* | | | | | | | | | | |
| PpCaM5-1 | | | Phpat.024G006800 | | 450 | | 149 | 2 | Chr24:1487455..1489394 | |
| PpCaM5-2 | | | Phpat.010G082200 | | 450 | | 149 | 2 | Chr10:14787471..14789302 | |
| PpCaM5-3 | | | Phpat.025G006800 | | 579 | | 192 | 0 | Chr25:935523..937062 | |
| PpCaM5-4 | | | Phpat.014G030000 | | 450 | | 149 | 3 | Chr14:5508383..5510531 | |
| PpCaM7 | | | Phpat.020G076400 | | 450 | | 149 | 1 | Chr20:13936769..13938528 | |
| PpCaM8-1 | | | Phpat.003G135400 | | 519 | | 172 | 6 | Chr03:23089095..23092119 | |
| PpCaM8-2 | | | Phpat.004G006200 | | 510 | | 169 | 6 | Chr04:1066886..1070483 | |
| *Picea abies* | | | | | | | | | | |
| PaCaM1-1 | | | MA_52889g0010 | | 450 | | 149 | 3 | MA_52889:3092...4552 | |
| PaCaM1-2 | | | MA_48600g0010 | | 552 | | 183 | 0 | MA_48600:4939...5530 | |
| PaCaM3 | | | MA_52449g0010 | | 514 | | 170 | 4 | MA_52449:-19...4596 | |
| PaCaM5-1 | | | MA_66144g0010 | | 450 | | 149 | 3 | MA_66144:12374...16500 | |
| PaCaM5-2 | | | MA_10429336g0010 | | 450 | | 149 | 1 | MA_10429336:8732...12457 | |
| PaCaM5-3 | | | MA_10428197g0010 | | 591 | | 196 | 3 | MA_10428197:8520...40021 | |
| PaCaM6-1 | | | MA_335961g0010 | | 444 | | 147 | 0 | MA_335961:3475...3958 | |
| PaCaM6-2 | | | MA_91036g0010 | | 582 | | 193 | 5 | MA_91036:15904...19116 | |
| PaCaM7 | | | MA_15211g0010 | | 465 | | 154 | 1 | MA_15211:2205...9723 | |
| *Populous trichocarpa* | | | | | | | | | | |
| PtCaM2 | | | Potri.015G032600 | | 450 | | 149 | 3 | Chr15:2629536..2632931 | |
| PtCaM3 | | | Potri.012G041000 | | 450 | | 149 | 3 | Chr12:3682715..3685215 | |
| PtCaM6-1 | | | Potri.016G024700 | | 450 | | 149 | 2 | Chr16:1392584..1394934 | |
| PtCaM6-2 | | | Potri.006G026700 | | 450 | | 149 | 1 | Chr06:1807595..1808904 | |
| PtCaM7 | | | Potri.009G021500 | | 540 | | 179 | 2 | Chr09:3333550..3337206 | |
| PtCaM8-1 | | | Potri.008G159300 | | 453 | | 150 | 3 | Chr08:10815709..10817972 | |
| PtCaM8-2 | | | Potri.010G080900 | | 453 | | 150 | 3 | Chr10:10696668..10698894 | |
| PtCaM8-3 | | | Potri.005G052800 | | 450 | | 149 | 3 | Chr05:3770064..3771477 | |
| *Prunus persica* | | | | | | | | | | |
| PperCaM5 | | | ppa012912m.g | | 450 | | 149 | 1 | scaffold_4:25011636..25014071 | |
| PperCaM7-1 | | | ppa012070m.g | | 555 | | 184 | 2 | scaffold_7:1025641..1028056 | |
| PperCaM7-2 | | | ppa012922m.g | | 450 | | 149 | 3 | scaffold_5:18042237..18044427 | |
| PperCaM8 | | | ppa012907m.g | | 453 | | 150 | 3 | scaffold_1:1660475..1662387 | |
| *Ricinus communis* | | | | | | | | | | |
| RcCaM7-1 | | | 30089.t000002 | | 450 | | 149 | 1 | 30089:87408..89201 | |
| RcCaM7-2 | | | 29828.t000012 | | 450 | | 149 | 1 | 29828:299975..301772 | |
| RcCaM9-1 | | | 29883.t000021 | | 447 | | 148 | 3 | 29883:168978..169684 | |
| RcCaM9-2 | | | 30174.t000299 | | 564 | | 187 | 0 | 30174:889340..889903 | |
| *Selaginella moellendorffii* | | | | | | | | | | |
| SmCaM1-1 | | | 159356 | | 450 | | 149 | 5 | scaffold_87:588438..589993 | |
| SmCaM1-2 | | | 413463 | | 492 | | 163 | 3 | scaffold_20:1893859..1894579 | |
| SmCaM5-1 | | | 141966 | | 450 | | 149 | 1 | scaffold_3:3786316..3787319 | |
| SmCaM5-2 | | | 181303 | | 459 | | 152 | 2 | scaffold_64:818481..819445 | |
| SmCaM5-3 | | | 107557 | | 450 | | 149 | 1 | scaffold_35:356987..357725 | |
| SmCaM5-6 | | | 83841 | | 459 | | 152 | 3 | scaffold_6:477157..477858 | |
| *Setaria italica* | | | | | | | | | | |
| SiCaM1-1 | | | Si037909m.g | | 450 | | 149 | 1 | scaffold_9:47944522..47946788 | |
| SiCaM1-2 | | | Si002816m.g | | 678 | | 225 | 2 | scaffold_5:4377776..4382844 | |
| SiCaM1-3 | | | Si023544m.g | | 450 | | 149 | 1 | scaffold_3:14923845..14926348 | |
| SiCaM1-4 | | | Si032548m.g | | 435 | | 144 | 3 | scaffold_2:214813..216216 | |
| SiCaM3 | | | Si027608m.g | | 543 | | 180 | 2 | scaffold_8:215604..216668 | |
| *Solanum lycopersicum* | | | | | | | | | | |
| SlCaM1 | | | Solyc06g053930.2 | | 450 | | 149 | 3 | ch06:33328449..33330360 | |
| SlCaM2-1 | | | Solyc11g072240.1 | | 450 | | 149 | 1 | ch11:52538666..52540732 | |
| SlCaM2-2 | | | Solyc10g077010.1 | | 450 | | 149 | 1 | ch10:59260504..59262472 | |
| SlCaM5 | | | Solyc10g081170.1 | | 450 | | 149 | 1 | ch10:61626964..61628347 | |
| SlCaM3 | | | Solyc12g099990.1 | | 450 | | 149 | 1 | ch12:65249443..65252546 | |
| SlCaM4 | | | Solyc01g105630.2 | | 444 | | 147 | 3 | ch01:85497235..85498101 | |
| SlCaM5-1 | | | Solyc03g005240.1 | | 462 | | 153 | 3 | ch03:139692..142348 | |
| SlCaM5-2 | | | Solyc01g008950.2 | | 450 | | 149 | 1 | ch01:2960056..2963201 | |
| SlCaM8 | | | Solyc02g090810.2 | | 507 | | 168 | 6 | ch02:46876372..46878474 | |
| *Solanum tuberosum* | | | | | | | | | | |
| StCaM2-1 | | | PGSC0003DMG400033685 | | 450 | | 149 | 3 | chr03:35492665..35496314 | |
| StCaM2-2 | | | PGSC0003DMG400007205 | | 450 | | 149 | 1 | chr10:43298120..43300593 | |
| StCaM3 | | | PGSC0003DMG400022693 | | 450 | | 149 | 1 | chr01:4726488..4729652 | |
| StCaM5 | | | PGSC0003DMG400027384 | | 450 | | 149 | 1 | chr11:41005290..41008897 | |
| StCaM8 | | | PGSC0003DMG400013390 | | 462 | | 153 | 3 | chr03:532882..535539 | |
| *Sorghum bicolor* | | | | | | | | | | |
| SbCaM1-1 | | | Sobic.009G180000 | | 450 | | 149 | 1 | Chr09:53450704..53453625 | |
| SbCaM1-2 | | | Sobic.002G424300 | | 450 | | 149 | 1 | Chr02:77077866..77079686 | |
| SbCaM1-3 | | | Sobic.009G180000 | | 450 | | 149 | 1 | Chr09:53450704..53453625 | |
| SbCaM1-4 | | | Sobic.001G390300 | | 450 | | 149 | 1 | Chr01:60476874..60480792 | |
| SbCaM1-5 | | | Sobic.001G112300 | | 450 | | 149 | 3 | Chr01:8791765..8795468 | |
| SbCaM1-6 | | | Sobic.003G330300 | | 555 | | 184 | 2 | Chr03:65550004..65552602 | |
| SbCaM1-7 | | | Sobic.001G094400 | | 465 | | 154 | 4 | Chr01:7240544..7243147 | |
| SbCaM3 | | | Sobic.005G024300 | | 543 | | 180 | 2 | Chr05:2206074..2207485 | |
| *Thelluginella halophila* (*Eutrema salsugineum*) | | | | | | | | | | |
| ThCaM1 | | | Thhalv10019293m.g | | 450 | | 149 | 1 | scaffold_9:8857233..8859113 | |
| ThCaM3-1 | | | Thhalv10002103m.g | | 543 | | 180 | 2 | scaffold_12:102059..104400 | |
| ThCaM3-2 | | | Thhalv10017373m.g | | 450 | | 149 | 1 | scaffold_10:11067197..11068631 | |
| ThCaM4 | | | Thhalv10027976m.g | | 450 | | 149 | 1 | scaffold_14:6501357..6502940 | |
| ThCaM5-1 | | | Thhalv10006295m.g | | 450 | | 149 | 1 | scaffold_19:2432165..2433642 | |
| ThCaM5-2 | | | Thhalv10002700m.g | | 450 | | 149 | 1 | scaffold_4:14098174..14100181 | |
| ThCaM5-3 | | | Thhalv10014926m.g | | 453 | | 150 | 1 | scaffold_2:7374742..7375966 | |
| ThCaM7 | | | Thhalv10009546m.g | | 459 | | 152 | 1 | scaffold_5:13794658..13795208 | |
| ThCaM8 | | | Thhalv10026490m.g | | 456 | | 151 | 3 | scaffold_1:13504298..13506986 | |
| ThCaM9 | | | Thhalv10010796m.g | | 456 | | 151 | 3 | scaffold_16:1740724..1741817 | |
| *Theobroma cacao* | | | | | | | | | | |
| TcCaM5 | | | Thecc1EG041153 | | 555 | | 184 | 2 | scaffold_9:35793140..35797044 | |
| ThCaM8 | | | Thecc1EG020731 | | 459 | | 152 | 3 | scaffold_4:29953718..29955413 | |
| *Vitis vinifera* | | | | | | | | | | |
| VvCaM5-1 | | | GSVIVG01000380001 | | 450 | | 149 | 3 | chr17_random:371956..379989 | |
| VvCaM5-2 | | | GSVIVG01021797001 | | 462 | | 153 | 3 | chr14:7260625..7262658 | |
| VvCaM7 | | | GSVIVG01037418001 | | 462 | | 153 | 2 | chr6:14126556..14129950 | |
| VvCaM8-1 | | | GSVIVG01018070001 | | 450 | | 149 | 3 | chr5:6213952..6215356 | |
| VvCaM8-2 | | | GSVIVG01003132001 | | 450 | | 149 | 3 | chrUn:7523488..7524903 | |
| *Volvox carteri* | | | | | | | | | | |
| VcCaM3 | | | Vocar20008528m.g | | 486 | | 161 | 4 | scaffold_1:3220773..3224615 | |
| VcCaM5 | | | Vocar20001229m.g | | 555 | | 184 | 0 | scaffold_24:385125..386465 | |
| VcCaM 6-1 | | | Vocar20000711m.g | | 438 | | 145 | 3 | scaffold_19:1827851..1830073 | |
| VcCaM6-2 | | | Vocar20007941m.g | | 498 | | 165 | 6 | scaffold_15:2460407..2462641 | |
| *Zea mays* | | | | | | | | | | |
| ZmCaM1-1 | | | GRMZM2G044963 | | 450 | | 149 | 1 | 8:3573239..3577147 | |
| ZmCaM1-2 | | | GRMZM2G395244 | | 546 | | 182 | 2 | 2:218313414..218316109 | |
| ZmCaM1-3 | | | GRMZM2G152891 | | 474 | | 158 | 2 | 1:52092838..52096639 | |
| ZmCaM1-4 | | | GRMZM2G117582 | | 597 | | 198 | 2 | 6:155708781..155712626 | |
| ZmCaM1-6 | | | GRMZM2G004703 | | 450 | | 149 | 1 | 8:119432505..119435152 | |
| ZmCaM1-7 | | | GRMZM2G029107 | | 450 | | 149 | 1 | 9:134490909..134494910 | |
| ZmCaM1-8 | | | GRMZM2G067511 | | 450 | | 149 | 1 | 3:38428315..38432905 | |
| ZmCaM3 | | | GRMZM2G146720 | | 543 | | 180 | 2 | 4:186214191..186216020 | |
