# Supplementary material for: Genomics and evolutionary aspect of calcium signaling event in calmodulin and calmodulin-like proteins in plants
Source: BMC Plant Biol. 2017 Feb 3;17:38. doi: 10.1186/s12870-017-0989-3 (PMC5291997; doi:10.1186/s12870-017-0989-3)
Supplement: Additional file 2: Table S2. — Calmodulin-like (CML) gene family members of monocot, dicot and lower eukaryotic plant lineages. Table shows gene name, locus ID, open reading frame (ORF), number of introns and 5'-3' coordinates of CML genes. (DOC 1223 kb) [file 12870_2017_989_MOESM2_ESM.doc]

Additional file 2: Table S2: Calmodulin-like (*CML*) gene family members of monocot, dicot and lower eukaryotic plant lineages. Table shows gene name, locus ID, open reading frame (ORF), number of introns and 5'-3' coordinates of *CML* genes.

| **Gene Name** | **Locus ID** | | | | | **ORF** | | **No. of amino acids** | **No. of Introns** | **5'-3' Coordinate** | |
| --- | --- | --- | --- | --- | --- | --- | --- | --- | --- | --- | --- |
| *Aguilegia coerulea* | | | | | | | | | | | |
| AcCML3-1 | Aquca_020_00064 | | | | | 693 | | 230 | 0 | | scaffold_20: 545888 - 546580 |
| AcCML3-2 | Aquca_058_00098 | | | | | 699 | | 232 | 0 | | scaffold_58: 613323 - 614021 |
| AcCML11 | Aquca_017_00557 | | | | | 450 | | 149 | 3 | | scaffold_17: 3367082 - 3368432 |
| AcCML16 | Aquca_002_01045 | | | | | 675 | | 224 | 1 | | scaffold_2: 7844672 - 7846020 |
| AcCML18 | Aquca_045_00104 | | | | | 489 | | 162 | 0 | | scaffold_45: 767863 - 768351 |
| AcCML20 | Aquca_007_00652 | | | | | 510 | | 169 | 6 | | scaffold_7: 5163490 - 5168130 |
| AcCML21 | Aquca_005_00289 | | | | | 696 | | 231 | 4 | | scaffold_5: 2884962 - 2889071 |
| AcCML22 | Aquca_034_00136 | | | | | 747 | | 248 | 4 | | scaffold_34: 1054056 - 1056165 |
| AcCML23 | Aquca_142_00012 | | | | | 501 | | 166 | 1 | | scaffold_142: 66644 - 67573 |
| AcCML25-1 | Aquca_024_00114 | | | | | 561 | | 186 | 0 | | scaffold_24: 3010645 - 3011205 |
| AcCML25-2 | Aquca_024_00161 | | | | | 651 | | 216 | 0 | | scaffold_24: 3747556 - 3748508 |
| AcCML25-3 | Aquca_024_00164 | | | | | 348 | | 115 | 0 | | scaffold_24: 3801349 - 3801696 |
| AcCML25-4 | Aquca_013_00571 | | | | | 792 | | 263 | 0 | | scaffold_13: 3761066 - 3762544 |
| AcCML25-5 | Aquca_014_00163 | | | | | 474 | | 157 | 0 | | scaffold_14: 924515 - 924988 |
| AcCML27 | Aquca_002_01215 | | | | | 489 | | 162 | 0 | | scaffold_2: 8949868 - 8950356 |
| AcCML30-1 | Aquca_007_00642 | | | | | 552 | | 183 | 0 | | scaffold_7: 5117609 - 5118160 |
| AcCML30-2 | Aquca_007_00644 | | | | | 552 | | 183 | 0 | | scaffold_7: 5122270 - 5122821 |
| AcCML38 | Aquca_038_00011 | | | | | 651 | | 216 | 0 | | scaffold_38: 98013 - 98663 |
| AcCML39 | Aquca_011_00211 | | | | | 438 | | 145 | 0 | | scaffold_11: 3268177 - 3268614 |
| AcCML41-1 | Aquca_007_00126 | | | | | 603 | | 200 | 0 | | scaffold_7: 1172262 - 1173088 |
| AcCML41-2 | Aquca_007_00128 | | | | | 603 | | 200 | 0 | | scaffold_7: 1193328 - 1193930 |
| *Arabidopsis thaliana* | | | | | | | | | | | |
| AtCML1 | [At3g59450](http://signal.salk.edu/cgi-bin/tdnaexpress?GENE=At3g59450&FUNCTION=&TDNA=) | | | | | 447 | | 148 | 4 | Chr3: 21972840-21974923 | |
| AtCML2 | [At4g12860](http://signal.salk.edu/cgi-bin/tdnaexpress?GENE=At4g12860&FUNCTION=&TDNA=) | | | | | 447 | | 148 | 4 | Chr3: 21972840-21974923 | |
| AtCML3 | [At3g07490](http://signal.salk.edu/cgi-bin/tdnaexpress?GENE=At3g07490&FUNCTION=&TDNA=) | | | | | 462 | | 153 | 0 | Chr3: 2391189...2391650 | |
| AtCML4 | [At3g59440](http://signal.salk.edu/cgi-bin/tdnaexpress?GENE=At3g59440&FUNCTION=&TDNA=) | | | | | 588 | | 195 | 0 | Chr3: 21970355 - 21971122 | |
| AtCML5 | [At2g43290](http://signal.salk.edu/cgi-bin/tdnaexpress?GENE=At2g43290&FUNCTION=&TDNA=) | | | | | 648 | | 215 | 0 | Chr2: 17991052 - 17992047 | |
| AtCML6 | [At4g03290](http://signal.salk.edu/cgi-bin/tdnaexpress?GENE=At4g03290&FUNCTION=&TDNA=) | | | | | 465 | | 154 | 0 | Chr4: 1442634 - 1443499 | |
| AtCML7 | [At1g05990](http://signal.salk.edu/cgi-bin/tdnaexpress?GENE=At1g05990&FUNCTION=&TDNA=) | | | | | 453 | | 150 | 0 | Chr1: 1818447 - 1819040 | |
| AtCML11 | [At3g22930](http://signal.salk.edu/cgi-bin/tdnaexpress?GENE=At3g22930&FUNCTION=&TDNA=) | | | | | 522 | | 173 | 2 | Chr3: 8124083 - 8125931 | |
| AtCML12 | [At2g41100](http://signal.salk.edu/cgi-bin/tdnaexpress?GENE=At2g41100&FUNCTION=&TDNA=) | | | | | 975 | | 324 | 4 | Chr2: 17137829...17139612 | |
| AtCML13 | [At1g12310](http://signal.salk.edu/cgi-bin/tdnaexpress?GENE=At1g12310&FUNCTION=&TDNA=) | | | | | 447 | | 148 | 0 | Chr1: 4187165...4188056 | |
| AtCML14 | [At1g62820](http://signal.salk.edu/cgi-bin/tdnaexpress?GENE=At1g62820&FUNCTION=&TDNA=) | | | | | 447 | | 148 | 0 | Chr1: 23263671...23264349 | |
| AtCML15 | [At1g18530](http://signal.salk.edu/cgi-bin/tdnaexpress?GENE=At1g18530&FUNCTION=&TDNA=) | | | | | 474 | | 157 | 0 | Chr1: 6376783 - 6377256 | |
| AtCML16 | [At3g25600](http://signal.salk.edu/cgi-bin/tdnaexpress?GENE=At3g25600&FUNCTION=&TDNA=) | | | | | 486 | | 161 | 0 | Chr3: 9307254 - 9307967 | |
| AtCML17 | [At1g32250](http://signal.salk.edu/cgi-bin/tdnaexpress?GENE=At1g32250&FUNCTION=&TDNA=) | | | | | 501 | | 166 | 0 | Chr1: 11639843 - 11640343 | |
| AtCML18 | [At3g03000](http://signal.salk.edu/cgi-bin/tdnaexpress?GENE=At3g03000&FUNCTION=&TDNA=) | | | | | 498 | | 165 | 0 | Chr3: 677240 - 678084 | |
| AtCML19 | [At4g37010](http://signal.salk.edu/cgi-bin/tdnaexpress?GENE=At4g37010&FUNCTION=&TDNA=) | | | | | 516 | | 171 | 5 | Chr4: 17444309 - 17445615 | |
| AtCML20 | [At3g50360](http://signal.salk.edu/cgi-bin/tdnaexpress?GENE=At3g50360&FUNCTION=&TDNA=) | | | | | 510 | | 169 | 5 | Chr3: 18674337 - 18675712 | |
| AtCML21 | [At4g26470](http://signal.salk.edu/cgi-bin/tdnaexpress?GENE=At4g26470&FUNCTION=&TDNA=) | | | | | 696 | | 231 | 4 | Chr4: 13371066 - 13372392 | |
| AtCML22 | [At3g24110](http://signal.salk.edu/cgi-bin/tdnaexpress?GENE=At3g24110&FUNCTION=&TDNA=) | | | | | 900 | | 299 | 3 | Chr3: 8704429 - 8705413 | |
| AtCML23 | [At1g66400](http://signal.salk.edu/cgi-bin/tdnaexpress?GENE=At1g66400&FUNCTION=&TDNA=) | | | | | 474 | | 157 | 0 | Chr1: 24770575 - 24771371 | |
| AtCML24 | [At5g37770](http://signal.salk.edu/cgi-bin/tdnaexpress?GENE=At5g37770&FUNCTION=&TDNA=) | | | | | 486 | | 161 | 0 | Chr5: 14998854 - 14999619 | |
| AtCML25 | [At1g24620](http://signal.salk.edu/cgi-bin/tdnaexpress?GENE=At1g24620&FUNCTION=&TDNA=) | | | | | 561 | | 186 | 0 | Chr1: 8723712 - 8724459 | |
| AtCML26 | [At1g73630](http://signal.salk.edu/cgi-bin/tdnaexpress?GENE=At1g73630&FUNCTION=&TDNA=) | | | | | 492 | | 163 | 0 | Chr1: 27684736 - 27685453 | |
| AtCML27 | [At1g18210](http://signal.salk.edu/cgi-bin/tdnaexpress?GENE=At1g18210&FUNCTION=&TDNA=) | | | | | 513 | | 170 | 1 | Chr1: 6267969 - 6268828 | |
| AtCML28 | [At3g03430](http://signal.salk.edu/cgi-bin/tdnaexpress?GENE=At3g03430&FUNCTION=&TDNA=) | | | | | 252 | | 83 | 0 | Chr3: 814473...814732 | |
| AtCML29 | [At5g17480](http://signal.salk.edu/cgi-bin/tdnaexpress?GENE=At5g17480&FUNCTION=&TDNA=) | | | | | 252 | | 83 | 0 | Chr5: 5762689...5762940 | |
| AtCML30 | [At2g15680](http://signal.salk.edu/cgi-bin/tdnaexpress?GENE=At2g15680&FUNCTION=&TDNA=) | | | | | 564 | | 187 | 0 | Chr2: 6830995 - 6831641 | |
| AtCML31 | [At2g36180](http://signal.salk.edu/cgi-bin/tdnaexpress?GENE=At2g36180&FUNCTION=&TDNA=) | | | | | 435 | | 144 | 0 | Chr2: 15173782 - 15174216 | |
| AtCML32 | [At5g17470](http://signal.salk.edu/cgi-bin/tdnaexpress?GENE=At5g17470&FUNCTION=&TDNA=) | | | | | 441 | | 146 | 0 | Chr5: 5760966 - 5761406 | |
| AtCML33 | [At3g03400](http://signal.salk.edu/cgi-bin/tdnaexpress?GENE=At3g03400&FUNCTION=&TDNA=) | | | | | 414 | | 137 | 0 | Chr3: 808745 - 809158 | |
| AtCML34 | [At3g03410](http://signal.salk.edu/cgi-bin/tdnaexpress?GENE=At3g03410&FUNCTION=&TDNA=) | | | | | 396 | | 131 | 0 | Chr3: 811324 - 811719 | |
| AtCML35 | [At2g41410](http://signal.salk.edu/cgi-bin/tdnaexpress?GENE=At2g41410&FUNCTION=&TDNA=) | | | | | 651 | | 216 | 0 | Chr2: 17261728 - 17262884 | |
| AtCML36 | [At3g10190](http://signal.salk.edu/cgi-bin/tdnaexpress?GENE=At3g10190&FUNCTION=&TDNA=) | | | | | 630 | | 209 | 0 | Chr3: 3155298 - 3156143 | |
| AtCML37 | [At5g42380](http://signal.salk.edu/cgi-bin/tdnaexpress?GENE=At5g42380&FUNCTION=&TDNA=) | | | | | 558 | | 185 | 0 | Chr5: 16942576 - 16943366 | |
| AtCML38 | [At1g76650](http://signal.salk.edu/cgi-bin/tdnaexpress?GENE=At1g76650&FUNCTION=&TDNA=) | | | | | 534 | | 177 | 0 | Chr1: 28766750 - 28767517 | |
| AtCML39 | [At1g76640](http://signal.salk.edu/cgi-bin/tdnaexpress?GENE=At1g76640&FUNCTION=&TDNA=) | | | | | 480 | | 159 | 0 | Chr1: 28765324 - 28765803 | |
| AtCML40 | [At3g01830](http://signal.salk.edu/cgi-bin/tdnaexpress?GENE=At3g01830&FUNCTION=&TDNA=) | | | | | 441 | | 146 | 0 | Chr3: 296054...299263 | |
| AtCML41 | [At3g50770](http://signal.salk.edu/cgi-bin/tdnaexpress?GENE=At3g50770&FUNCTION=&TDNA=) | | | | | 618 | | 205 | 0 | Chr3: 18873958 - 18874780 | |
| AtCML42 | [At4g20780](http://signal.salk.edu/cgi-bin/tdnaexpress?GENE=At4g20780&FUNCTION=&TDNA=) | | | | | 576 | | 191 | 0 | Chr4: 11133186 - 11133970 | |
| AtCML43 | [At5g44460](http://signal.salk.edu/cgi-bin/tdnaexpress?GENE=At5g44460&FUNCTION=&TDNA=) | | | | | 546 | | 181 | 0 | Chr5: 17917226 - 17917983 | |
| AtCML44 | [At1g21550](http://signal.salk.edu/cgi-bin/tdnaexpress?GENE=At1g21550&FUNCTION=&TDNA=) | | | | | 468 | | 155 | 0 | Chr1: 7553101...7553876 | |
| AtCML45 | [At3g29000](http://signal.salk.edu/cgi-bin/tdnaexpress?GENE=At3g29000&FUNCTION=&TDNA=) | | | | | 585 | | 194 | 0 | Chr3: 11005779...11006414 | |
| AtCML46 | [At5g39670](http://signal.salk.edu/cgi-bin/tdnaexpress?GENE=At5g39670&FUNCTION=&TDNA=) | | | | | 615 | | 204 | 0 | Chr5: 15883179...15884067 | |
| AtCML47 | [At3g47480](http://signal.salk.edu/cgi-bin/tdnaexpress?GENE=At3g47480&FUNCTION=&TDNA=) | | | | | 552 | | 183 | 0 | Chr3: 17496354...17496947 | |
| AtCML48 | [At2g27480](http://signal.salk.edu/cgi-bin/tdnaexpress?GENE=At2g27480&FUNCTION=&TDNA=) | | | | | 687 | | 228 | 4 | Chr2: 11746720...11748051 | |
| AtCML49 | [At3g10300](http://signal.salk.edu/cgi-bin/tdnaexpress?GENE=At3g10300&FUNCTION=&TDNA=) | | | | | 1008 | | 335 | 0 | Chr3: 3186314...3188205 | |
| AtCML50 | [At5g04170](http://signal.salk.edu/cgi-bin/tdnaexpress?GENE=At5g04170&FUNCTION=&TDNA=) | | | | | 1065 | | 354 | 4 | Chr5: 1145431...1147781 | |
| *Brachypodium distachyon* | | | | | | | | | | | |
| BdCML1 | Bradi2g52940 | | | | | 552 | | 183 | 2 | Bd2:52219384..52224070 | |
| BdCML2 | Bradi4g26400 | | | | | 768 | | 255 | 2 | Bd4:31680296..31681638 | |
| BdCML3 | Bradi4g43630 | | | | | 546 | | 181 | 2 | Bd4: 47270297 - 47271341 | |
| BdCML4 | Bradi1g09040 | | | | | 465 | | 154 | 3 | Bd1: 6389708 - 6391954 | |
| BdCML5 | Bradi4g02480 | | | | | 477 | | 158 | 0 | Bd4: 1649307 - 1650167 | |
| BdCML8 | Bradi1g21070 | | | | | 507 | | 168 | 6 | Bd1: 16961980 - 16964156 | |
| BdCML10 | Bradi2g60660 | | | | | 588 | | 195 | 0 | Bd2: 57963624 - 57964518 | |
| BdCML11 | Bradi3g34380 | | | | | 591 | | 196 | 0 | Bd3: 36781438 - 36782028 | |
| BdCML12 | Bradi2g42920 | | | | | 696 | | 231 | 0 | Bd2: 43394651 - 43395638 | |
| BdCML13 | Bradi2g10790 | | | | | 450 | | 149 | 0 | Bd2: 9010480 - 9013295 | |
| BdCML14 | Bradi2g15570 | | | | | 546 | | 181 | 0 | Bd2: 13844626 - 13845408 | |
| BdCML15 | Bradi2g27050 | | | | | 576 | | 191 | 0 | Bd2: 25782009 - 25782941 | |
| BdCML16 | Bradi2g02340 | | | | | 570 | | 189 | 0 | Bd2: 1594720 - 1595741 | |
| BdCML20 | Bradi3g56850 | | | | | 477 | | 158 | 0 | Bd3: 56593654 - 56594130 | |
| BdCML21 | Bradi2g10010 | | | | | 450 | | 149 | 1 | Bd2: 8192560 - 8196245 | |
| BdCML22 | Bradi5g14297 | | | | | 738 | | 245 | 0 | Bd5: 17719555 - 17720619 | |
| BdCML23 | Bradi2g51090 | | | | | 456 | | 151 | 0 | Bd2: 50744967 - 50745634 | |
| BdCML24 | Bradi1g17236 | | | | | 450 | | 149 | 1 | Bd1: 13811143 - 13812767 | |
| BdCML28 | Bradi4g39900 | | | | | 474 | | 157 | 0 | Bd4: 44460140 - 44460613 | |
| BdCML29 | Bradi4g32200 | | | | | 552 | | 183 | 0 | Bd4: 37908745 - 37909689 | |
| BdCML30 | Bradi1g48070 | | | | | 720 | | 239 | 0 | Bd1: 46684770 - 46686046 | |
| BdCML31 | Bradi2g61150 | | | | | 462 | | 153 | 0 | Bd2: 58291086 - 58291547 | |
| BdCML32 | Bradi2g36187 | | | | | 735 | | 244 | 4 | Bd2: 36629461 - 36630598 | |
| *Brassica rapa* | | | | | | | | | | | |
| BrCML2-1 | Brara.D00748 | | | | | 459 | | 152 | 0 | A04:6379069..6379527 | |
| BrCML2-2 | Brara.H00492 | | | | | 459 | | 152 | 0 | A08:5340208..5340666 | |
| BrCML3-1 | Brara.A03606 | | | | | 450 | | 149 | 0 | A01:29133332..29133781 | |
| BrCML3-2 | Brara.C03212 | | | | | 462 | | 153 | 0 | A03:16295886..16296475 | |
| BrCML4 | Brara.G01846 | | | | | 612 | | 203 | 0 | A07:17258798..17259409 | |
| BrCML5-1 | Brara.D02625 | | | | | 633 | | 210 | 0 | A04:20352759..20353391 | |
| BrCML5-2 | Brara.C02195 | | | | | 645 | | 214 | 0 | A03:11005711..11006355 | |
| BrCML5-3 | Brara.C02762 | | | | | 465 | | 154 | 0 | A03:14152700..14153164 | |
| BrCML11-1 | Brara.C03895 | | | | | 453 | | 150 | 3 | A03:20204995..20207038 | |
| BrCML11-2 | Brara.E01967 | | | | | 468 | | 155 | 6 | A05:17819866..17824221 | |
| BrCML11-3 | Brara.A02692 | | | | | 471 | | 156 | 2 | A01:22695440..22697258 | |
| BrCML15 | Brara.I04783 | | | | | 471 | | 156 | 0 | A09:40620975..40621445 | |
| BrCML16-1 | Brara.F03338 | | | | | 489 | | 162 | 0 | A06:26135973..26136681 | |
| BrCML16-2 | Brara.I02151 | | | | | 486 | | 161 | 0 | A09:16249841..16250506 | |
| BrCML17 | Brara.E01900 | | | | | 501 | | 166 | 0 | A05:16287292..16287792 | |
| BrCML18 | Brara.A03850 | | | | | 510 | | 169 | 0 | A01:30456651..30457160 | |
| BrCML23 | Brara.H02431 | | | | | 525 | | 174 | 0 | A08:20439871..20440644 | |
| BrCML24 | Brara.D01191 | | | | | 483 | | 160 | 0 | A04:10972109..10973187 | |
| BrCML25-1 | Brara.H02086 | | | | | 564 | | 187 | 0 | A08:18691713..18692276 | |
| BrCML25-2 | Brara.I01613 | | | | | 990 | | 329 | 2 | A09:10692687..10694531 | |
| BrCML25-3 | Brara.K01135 | | | | | 555 | | 184 | 0 | Scaffold5066:896..1450 | |
| BrCML26 | Brara.G03253 | | | | | 480 | | 159 | 0 | A07:25022673..25023152 | |
| BrCML27-1 | Brara.H02431 | | | | | 525 | | 174 | 0 | A08:20439871..20440644 | |
| BrCML27-2 | Brara.F01281 | | | | | 507 | | 168 | 0 | A06:7153824..7155019 | |
| BrCML30 | Brara.I00982 | | | | | 570 | | 189 | 0 | A09:5524678..5525312 | |
| BrCML32-1 | Brara.A00300 | | | | | 435 | | 144 | 0 | A03:590962..591396 | |
| BrCML32-2 | Brara.C00136 | | | | | 435 | | 144 | 0 | A03:590962..591396 | |
| BrCML34-1 | Brara.F00375 | | | | | 402 | | 133 | 0 | A06:2175601..2176002 | |
| BrCML34-2 | Brara.E01705 | | | | | 396 | | 131 | 0 | A05:12152201..12152596 | |
| BrCML35 | Brara.E00219 | | | | | 642 | | 213 | 0 | A05:1249556..1250197 | |
| BrCML36 | Brara.E03036 | | | | | 627 | | 208 | 0 | A05:25320430..25321056 | |
| BrCML37 | Brara.I01729 | | | | | 555 | | 184 | 0 | A09:11854417..11854971 | |
| BrCML38-1 | Brara.G03461 | | | | | 543 | | 180 | 0 | A07:26012062..26012604 | |
| BrCML38-2 | Brara.G02177 | | | | | 540 | | 179 | 0 | A07:19052948..19053696 | |
| BrCML39-1 | Brara.B02195 | | | | | 510 | | 169 | 0 | A02:14001851..14002360 | |
| BrCML39-2 | Brara.G03460 | | | | | 510 | | 169 | 0 | A07:26010146..26010655 | |
| *Capsella rubella* | | | | | | | | | | | |
| CrCML2 | | Carubv10007052m.g | | | | 459 | | 152 | 0 | scaffold_7: 11801984 - 11802442 | |
| CrCML3 | | Carubv10015864m.g | | | | 462 | | 153 | 0 | scaffold_3: 2423429 - 2423890 | |
| CrCML4 | | Carubv10018003m.g | | | | 651 | | 216 | 0 | scaffold_5: 12267204 - 12267855 | |
| CrCML5-1 | | Carubv10025336m.g | | | | 651 | | 216 | 0 | scaffold_4: 13183034 - 13183684 | |
| CrCML5-2 | | Carubv10003659m.g | | | | 465 | | 154 | 0 | scaffold_6: 15251569 - 15252033 | |
| CrCML36-1 | | Carubv10014618m.g | | | | 639 | | 212 | 0 | scaffold_3: 3208216 - 3209301 | |
| CrCML36-2 | | Carubv10002001m.g | | | | 600 | | 199 | 0 | scaffold_6: 1083951 - 1084622 | |
| CrCML7 | | Carubv10010897m.g | | | | 456 | | 151 | 0 | scaffold_1: 1895241 - 1895696 | |
| CrCML11 | | Carubv10014817m.g | | | | 474 | | 157 | 2 | scaffold_3: 8350150 - 8352023 | |
| CrCML12 | | Carubv10023991m.g | | | | 693 | | 230 | 2 | scaffold_4: 12281826 - 12283116 | |
| CrCML15 | | Carubv10011170m.g | | | | 474 | | 157 | 0 | scaffold_1: 6405303 - 6405776 | |
| CrCML16 | | Carubv10018156m.g | | | | 483 | | 160 | 0 | scaffold_5: 1352683 - 1353326 | |
| CrCML17 | | Carubv10012615m.g | | | | 501 | | 166 | 0 | scaffold_1: 11324749 - 11325249 | |
| CrCML18 | | Carubv10014793m.g | | | | 498 | | 165 | 0 | scaffold_3: 697214 - 697849 | |
| CrCML20 | | Carubv10018135m.g | | | | 510 | | 169 | 5 | scaffold_5: 8761467 - 8765501 | |
| CrCML21 | | Carubv10007314m.g | | | | 702 | | 233 | 3 | scaffold_7: 5416247 - 5417285 | |
| CrCML23 | | Carubv10022253m.g | | | | 462 | | 153 | 0 | scaffold_2: 7943885 - 7944346 | |
| CrCML24 | | Carubv10007462m.g | | | | 492 | | 163 | 0 | scaffold_7: 14966161 - 14966652 | |
| CrCML25 | | Carubv10011672m.g | | | | 555 | | 184 | 0 | scaffold_1: 9191775 - 9192329 | |
| CrCML26 | | Carubv10021066m.g | | | | 489 | | 162 | 0 | scaffold_2: 11251776 - 11252599 | |
| CrCML30 | | Carubv10015796m.g | | | | 636 | | 211 | 0 | scaffold_3: 12448102 - 12448737 | |
| CrCML32-1 | | Carubv10025116m.g | | | | 441 | | 146 | 0 | scaffold_4: 2222918 - 2223358 | |
| CrCML32-2 | | Carubv10003528m.g | | | | 441 | | 146 | 0 | scaffold_6: 5813038 - 5813478 | |
| CrCML32-3 | | Carubv10024624m.g | | | | 435 | | 144 | 0 | scaffold_4: 10133442 - 10133876 | |
| CrCML34 | | Carubv10015681m.g | | | | 396 | | 131 | 0 | scaffold_3: 826011 - 826406 | |
| CrCML35 | | Carubv10024577m.g | | | | 648 | | 215 | 0 | scaffold_4: 12412540 - 12413187 | |
| CrCML37 | | Carubv10028385m.g | | | | 540 | | 179 | 0 | scaffold_8: 3440500 - 3441039 | |
| CrCML38 | | Carubv10022145m.g | | | | 558 | | 185 | 0 | scaffold_2: 12425355 - 12425912 | |
| CrCML39 | | Carubv10021626m.g | | | | 480 | | 159 | 0 | scaffold_2: 12423538 - 12424017 | |
| *Carica papaya* | | | | | | | | | | | |
| CpCML3-1 | | evm.TU.supercontig_46.38 | | | | 678 | | 225 | 0 | supercontig_46: 307994 - 308671 | |
| CpCML3-2 | | evm.TU.supercontig_8.56 | | | | 459 | | 152 | 0 | supercontig_8: 380979 - 381437 | |
| CpCML5 | | evm.TU.supercontig_9.284 | | | | 678 | | 225 | 0 | supercontig_9: 1966321 - 1966998 | |
| CpCML15 | | evm.TU.supercontig_13.81 | | | | 486 | | 161 | 0 | supercontig_13: 835798 - 836283 | |
| CpCML16 | | evm.TU.supercontig_1.247 | | | | 483 | | 160 | 0 | supercontig_1: 3525379 - 3525861 | |
| CpCML18 | | evm.TU.supercontig_90.10 | | | | 495 | | 164 | 0 | supercontig_90: 291521 - 292015 | |
| CpCML20 | | evm.TU.supercontig_55.154 | | | | 510 | | 169 | 6 | supercontig_55: 964104 - 968079 | |
| CpCML21 | | evm.TU.supercontig_12.76 | | | | 744 | | 247 | 3 | supercontig_12: 611806 - 614372 | |
| CpCML24 | | evm.TU.supercontig_116.35 | | | | 495 | | 164 | 0 | supercontig_116: 278964 - 279458 | |
| CpCML25-1 | | evm.TU.supercontig_2.199 | | | | 621 | | 206 | 0 | supercontig_2: 2741382 - 2742002 | |
| CpCML25-2 | | evm.TU.supercontig_18.122 | | | | 615 | | 204 | 0 | supercontig_18: 1093477 - 1094091 | |
| CpCML27 | | evm.TU.supercontig_3.406 | | | | 525 | | 174 | 0 | supercontig_3: 2860348 - 2860872 | |
| CpCML35 | | evm.TU.supercontig_5.191 | | | | 672 | | 223 | 0 | supercontig_5: 1658921 - 1659592 | |
| CpCML37 | | evm.TU.supercontig_36.91 | | | | 573 | | 190 | 0 | supercontig_36: 734034 - 734606 | |
| CpCML40 | | evm.TU.supercontig_171.33 | | | | 438 | | 145 | 0 | supercontig_171: 467549 - 467986 | |
| *Chlamydomonas reinhardtii* | | | | | | | | | | | |
| CreinCML20-1 | | | Cre06.g289800 | | | 1107 | | 368 | 8 | Chr6: 6083476 - 6089657 | |
| CreinCML20-2 | | | g18380 | | | 1065 | | 354 | 9 | scaffold_40: 36 - 2671 | |
| CreinCAM32 | | | Cre03.g197500 | | | 1371 | | 456 | 8 | Chr3: 6695119 - 6699170 | |
| *Citrus clementina* | | | | | | | | | | | |
| CcCML3 | | | Ciclev10022299m.g | | | 612 | | 203 | 0 | scaffold_3: 38345735 - 38347292 | |
| CcCML7 | | | Ciclev10009394m.g | | | 681 | | 226 | 0 | scaffold_1: 25765317 - 25766713 | |
| CcCML15 | | | Ciclev10023347m.g | | | 483 | | 160 | 0 | scaffold_3: 9012256 - 9012738 | |
| CcCML16 | | | Ciclev10032937m.g | | | 492 | | 163 | 0 | scaffold_4: 19956092 - 19956932 | |
| CcCML18 | | | Ciclev10002732m.g | | | 492 | | 163 | 0 | scaffold_5: 27141622 - 27142692 | |
| CcCML20 | | | Ciclev10026635m.g | | | 510 | | 169 | 6 | scaffold_7: 3159264 - 3162290 | |
| CcCML21 | | | Ciclev10009385m.g | | | 690 | | 229 | 4 | scaffold_1: 24224244 - 24227187 | |
| CcCML23-1 | | | Ciclev10013012m.g | | | 462 | | 153 | 0 | scaffold_6: 22520370 - 22521865 | |
| CcCML23-2 | | | Ciclev10022392m.g | | | 579 | | 192 | 0 | scaffold_3: 49674215 - 49674873 | |
| CcCML25 | | | Ciclev10002752m.g | | | 474 | | 157 | 0 | scaffold_5: 36974513 - 36975459 | |
| CcCML27 | | | Ciclev10009740m.g | | | 492 | | 163 | 0 | scaffold_1: 14026557 - 14027565 | |
| CcCML30-1 | | | Ciclev10029994m.g | | | 564 | | 187 | 0 | scaffold_8: 4178524 - 4179087 | |
| CcCML30-2 | | | Ciclev10029360m.g | | | 558 | | 185 | 0 | scaffold_8: 3134318 - 3135122 | |
| CcCML36 | | | Ciclev10005842m.g | | | 666 | | 221 | 0 | scaffold_9: 22394207 - 22395728 | |
| CcCML37 | | | Ciclev10002523m.g | | | 612 | | 203 | 0 | scaffold_5: 43189010 - 43190241 | |
| CcCML38-1 | | | Ciclev10022753m.g | | | 429 | | 142 | 0 | scaffold_3: 4311923 - 4312682 | |
| CcCML38-2 | | | Ciclev10018267m.g | | | 426 | | 141 | 0 | scaffold_2: 13869717 - 13870142 | |
| CcCML41 | | | Ciclev10026510m.g | | | 615 | | 204 | 0 | scaffold_7: 2461706 - 2462587 | |
| CcCML42 | | | Ciclev10012776m.g | | | 624 | | 207 | 0 | scaffold_6: 17943468 - 17944691 | |
| *Citrus sinensis* | | | | | | | | | | | |
| CsCML3-1 | orange1.1g028812m.g | | | | | 612 | | 203 | 0 | scaffold00348: 1901 - 2538 | |
| CsCML3-2 | orange1.1g037200m.g | | | | | 456 | | 151 | 0 | scaffold00075: 114974 - 115429 | |
| CsCML7 | orange1.1g027734m.g | | | | | 660 | | 219 | 1 | scaffold00013: 483839 - 485426 | |
| CsCML15 | orange1.1g042615m.g | | | | | 483 | | 160 | 0 | scaffold00030: 202109 - 202591 | |
| CsCML16 | orange1.1g031260m.g | | | | | 492 | | 163 | 0 | scaffold00019: 892109 - 892801 | |
| CsCML17 | orange1.1g037787m.g | | | | | 492 | | 163 | 0 | scaffold00212: 259108 - 259599 | |
| CsCML20 | orange1.1g038366m.g | | | | | 510 | | 169 | 6 | scaffold00006: 1723370 - 1726360 | |
| CsCML21 | orange1.1g026998m.g | | | | | 690 | | 229 | 4 | scaffold00013: 1949436 - 1951795 | |
| CsCML22 | orange1.1g026268m.g | | | | | 726 | | 241 | 4 | scaffold00055: 482078 - 485090 | |
| CsCML23-1 | orange1.1g031791m.g | | | | | 462 | | 153 | 0 | scaffold00042: 5847 - 6787 | |
| CsCML23-2 | orange1.1g047502m.g | | | | | 438 | | 145 | 0 | scaffold00001: 1312141 - 1312578 | |
| CsCML25-1 | orange1.1g029304m.g | | | | | 588 | | 195 | 0 | scaffold00058: 87051 - 87797 | |
| CsCML25-2 | orange1.1g031582m.g | | | | | 474 | | 157 | 0 | scaffold00004: 968352 - 969105 | |
| CsCML27 | orange1.1g031226m.g | | | | | 492 | | 163 | 0 | scaffold00039: 203343 - 204301 | |
| CsCML30-1 | orange1.1g041237m.g | | | | | 558 | | 185 | 0 | scaffold00065: 53841 - 54398 | |
| CsCML30-2 | orange1.1g030421m.g | | | | | 534 | | 177 | 0 | scaffold00184: 43430 - 44268 | |
| CsCML36 | orange1.1g027592m.g | | | | | 666 | | 221 | 0 | scaffold04569: 1 - 1091 | |
| CsCML38-1 | orange1.1g032375m.g | | | | | 429 | | 142 | 0 | scaffold00158: 134776 - 135605 | |
| CsCML38-2 | orange1.1g037913m.g | | | | | 426 | | 141 | 0 | scaffold00110: 150690 - 151115 | |
| CsCML41 | orange1.1g028784m.g | | | | | 615 | | 204 | 0 | scaffold00006: 2365623 - 2366361 | |
| *Coccomyxa subellipsoidea* | | | | | | | | | | | |
| CsubCML20 | | estExt_Genewise1.C_100087 | | | | 507 | | 168 | 3 | scaffold_10: 723794 - 725618 | |
| CsubCML24 | | e_gw1.25.78.1 | | | | 861 | | 286 | 0 | scaffold_25: 569136 - 569996 | |
| *Cuccumis sativus* | | | | | | | | | | | |
| CsatCML3 | | | | | Cucsa.094740 | 453 | | 150 | 0 | scaffold00919: 861278 - 861730 | |
| CsatCML5 | | | | | Cucsa.132360 | 684 | | 227 | 0 | scaffold01037: 334615 - 335298 | |
| CsatCML15 | | | | | Cucsa.198740 | 483 | | 160 | 0 | scaffold01357: 1685210 - 1685692 | |
| CsatCML16 | | | | | Cucsa.284110 | 459 | | 152 | 0 | scaffold02653: 989079 - 989537 | |
| CsatCML18 | | | | | Cucsa.322020 | 468 | | 155 | 0 | scaffold03080: 1826577 - 1827044 | |
| CsatCML20 | | | | | Cucsa.366810 | 507 | | 168 | 6 | scaffold03625: 310020 - 315027 | |
| CsatCML21 | | | | | Cucsa.042260 | 690 | | 229 | 4 | scaffold00540: 726680 - 729074 | |
| CsatCML22 | | | | | Cucsa.256460 | 684 | | 227 | 4 | scaffold02229: 4085250 - 4087621 | |
| CsatCML23 | | | | | Cucsa.375430 | 414 | | 137 | 0 | scaffold03746: 518464 - 518877 | |
| CsatCML25-1 | | | | | Cucsa.012210 | 549 | | 182 | 0 | scaffold00154: 1686534 - 1687082 | |
| CsatCML25-2 | | | | | Cucsa.029820 | 449 | | 148 | 0 | scaffold00399: 60136 - 60584 | |
| CsatCML25-3 | | | | | Cucsa.274740 | 510 | | 169 | 1 | scaffold02541: 86255 - 86948 | |
| CsatCML27 | | | | | Cucsa.111510 | 567 | | 188 | 0 | scaffold00953: 276714 - 277600 | |
| CsatCML30-1 | | | | | Cucsa.219910 | 555 | | 184 | 0 | scaffold01658: 1503454 - 1504197 | |
| CsatCML30-2 | | | | | Cucsa.366800 | 573 | | 190 | 1 | scaffold03625: 301697 - 303052 | |
| CsatCML36 | | | | | Cucsa.362850 | 537 | | 178 | 1 | scaffold03611: 562441 - 563369 | |
| CsatCML38-1 | | | | | Cucsa.157410 | 429 | | 142 | 0 | scaffold01141: 352261 - 353151 | |
| CsatCML38-2 | | | | | Cucsa.087640 | 423 | | 140 | 0 | scaffold00888: 244246 - 244789 | |
| CsatCML38-3 | | | | | Cucsa.280520 | 438 | | 145 | 0 | scaffold02633: 415774 - 416211 | |
| CsatCML41 | | | | | Cucsa.054260 | 426 | | 141 | 0 | scaffold00582: 576087 - 576905 | |
| CsatCML50 | | | | | Cucsa.153010 | 516 | | 171 | 1 | scaffold01124: 269561 - 271134 | |
| *Eucalyptus grandis* | | | | | | | | | | | |
| EgCML3 | | | | | Eucgr.H00711 | 459 | | 152 | 0 | scaffold_8: 9401894 - 9402765 | |
| EgCML5-1 | | | | | Eucgr.H00167 | 654 | | 218 | 0 | scaffold_8: 1640438 - 1641091 | |
| EgCML5-2 | | | | | Eucgr.H00370 | 624 | | 207 | 0 | scaffold_8: 4937553 - 4939002 | |
| EgCML11 | | | | | Eucgr.H03405 | 459 | | 152 | 3 | scaffold_8: 49844491 - 49845529 | |
| EgCML15-1 | | | | | Eucgr.B00667 | 471 | | 156 | 0 | scaffold_2: 8440756 - 8441338 | |
| EgCML15-2 | | | | | Eucgr.G02382 | 489 | | 162 | 0 | scaffold_7: 42302750 - 42303438 | |
| EgCML17 | | | | | Eucgr.D02220 | 492 | | 163 | 0 | scaffold_4: 36358285 - 36359280 | |
| EgCML18 | | | | | Eucgr.A02973 | 426 | | 141 | 0 | scaffold_1: 40064515 - 40064940 | |
| EgCML20 | | | | | Eucgr.H01660 | 507 | | 168 | 6 | scaffold_8: 20296363 - 20300919 | |
| EgCML23 | | | | | Eucgr.F02840 | 516 | | 171 | 1 | scaffold_6: 38073103 - 38075502 | |
| EgCML24-1 | | | | | Eucgr.J01551 | 501 | | 166 | 0 | scaffold_10: 19970194 - 19970877 | |
| EgCML24-2 | | | | | Eucgr.H00832 | 468 | | 155 | 1 | scaffold_8: 10400011 - 10401175 | |
| EgCML25-1 | | | | | Eucgr.B02672 | 585 | | 194 | 0 | scaffold_2: 49308646 - 49309547 | |
| EgCML25-2 | | | | | Eucgr.D02448 | 429 | | 142 | 0 | scaffold_4: 38796776 - 38797204 | |
| EgCML26 | | | | | Eucgr.B00943 | 516 | | 171 | 0 | scaffold_2: 13312558 - 13313352 | |
| EgCML30-1 | | | | | Eucgr.I00935 | 549 | | 182 | 0 | scaffold_9: 19136881 - 19138097 | |
| EgCML30-2 | | | | | Eucgr.G01446 | 420 | | 139 | 0 | scaffold_7: 24989271 - 24990065 | |
| EgCML37 | | | | | Eucgr.F03632 | 606 | | 201 | 0 | scaffold_6: 44706754 - 44707694 | |
| EgCML38 | | | | | Eucgr.B00125 | 432 | | 143 | 0 | scaffold_2: 2142809 - 2143311 | |
| EgCML39-1 | | | | | Eucgr.B00124 | 432 | | 143 | 0 | scaffold_2: 2125068 - 2125557 | |
| EgCML39-2 | | | | | Eucgr.B00127 | 432 | | 143 | 0 | scaffold_2: 2157685 - 2158177 | |
| EgCML40-1 | | | | | Eucgr.B00128 | 426 | | 141 | 0 | scaffold_2: 2169290 - 2169738 | |
| EgCML40-2 | | | | | Eucgr.J02318 | 426 | | 141 | 0 | scaffold_10: 29151678 - 29152411 | |
| EgCML40-3 | | | | | Eucgr.B00123 | 432 | | 143 | 0 | scaffold_2: 2113375 - 2113890 | |
| EgCML41 | | | | | Eucgr.H01924 | 588 | | 195 | 0 | scaffold_8: 24861031 - 24861990 | |
| *Fragaria vesca* | | | | | | | | | | | |
| FvCML3 | | | | gene17503-v1.0-hybrid | | 471 | | 156 | 0 | LG2: 10650190 - 10650660 | |
| FvCML5 | | | | gene31102-v1.0-hybrid | | 660 | | 219 | 0 | LG1: 2535418 - 2536077 | |
| FvCML7 | | | | gene21676-v1.0-hybrid | | 606 | | 201 | 0 | LG4: 7529136 - 7529741 | |
| FvCML11 | | | | gene16557-v1.0-hybrid | | 453 | | 150 | 3 | LG1: 19846947 - 19848350 | |
| FvCML15 | | | | gene26183-v1.0-hybrid | | 1158 | | 385 | 2 | LG5: 8226995 - 8228728 | |
| FvCML16 | | | | gene04478-v1.0-hybrid | | 459 | | 152 | 0 | LG4: 25952767 - 25953225 | |
| FvCML18-1 | | | | gene12572-v1.0-hybrid | | 513 | | 170 | 0 | LG7: 19428403 - 19428915 | |
| FvCML18-2 | | | | gene08252-v1.0-hybrid | | 492 | | 163 | 0 | LG2: 12607829 - 12608320 | |
| FvCML20 | | | | gene24041-v1.0-hybrid | | 801 | | 266 | 7 | LG6: 35505246 - 35508650 | |
| FvCML21 | | | | gene01447-v1.0-hybrid | | 687 | | 228 | 3 | LG3: 29041572 - 29042752 | |
| FvCML22 | | | | gene09782-v1.0-hybrid | | 666 | | 221 | 3 | LG4: 12653889 - 12654848 | |
| FvCML23 | | | | gene25352-v1.0-hybrid | | 447 | | 148 | 0 | LG1: 11314707 - 11315153 | |
| FvCML25 | | | | gene19352-v1.0-hybrid | | 588 | | 195 | 0 | LG7: 4924975 - 4925562 | |
| FvCML27-1 | | | | gene09237-v1.0-hybrid | | 465 | | 154 | 0 | LG7: 9196149 - 9196613 | |
| FvCML27-2 | | | | gene09392-v1.0-hybrid | | 513 | | 170 | 0 | LG5: 10104046 - 10104558 | |
| FvCML36 | | | | gene21916-v1.0-hybrid | | 633 | | 210 | 0 | LG6: 10494040 - 10494672 | |
| FvCML38-1 | | | | gene25910-v1.0-hybrid | | 423 | | 140 | 0 | LG6: 38426998 - 38427420 | |
| FvCML38-2 | | | | gene29325-v1.0-hybrid | | 537 | | 178 | 0 | LG5: 19283420 - 19283956 | |
| FvCML38-3 | | | | gene06350-v1.0-hybrid | | 423 | | 140 | 0 | LG5: 4843910 - 4844332 | |
| *Glycine max* | | | | | | | | | | | |
| GmCML3-1 | | | | Glyma.12G052100 | | 666 | | 221 | 1 | Chr12:3728258..3730493 | |
| GmCML3-2 | | | | Glyma.12G052100 | | 666 | | 221 | 1 | Chr15:2420032..2426219 | |
| GmCML3-3 | | | | Glyma.13G344200 | | 555 | | 184 | 0 | Chr13:43525753..43527011 | |
| GmCML3-4 | | | | Glyma.19G129800 | | 459 | | 152 | 0 | Chr19:38934366..38935155 | |
| GmCML3-5 | | | | Glyma.03G127000 | | 459 | | 152 | 0 | Chr03:34108226..34108684 | |
| GmCML5-1 | | | | Glyma.17G112000 | | 690 | | 229 | 0 | Chr17:8807715..8808902 | |
| GmCML5-2 | | | | Glyma.13G159600 | | 690 | | 229 | 0 | Chr13:27482929..27484304 | |
| GmCML5-3 | | | | Glyma.15G030100 | | 636 | | 211 | 1 | Chr15:2420032..2426219 | |
| GmCML11-1 | | | | Glyma.19G244300 | | 450 | | 149 | 3 | Chr19:49159509..49160879 | |
| GmCML11-2 | | | | Glyma.03G246800 | | 450 | | 149 | 3 | Chr03:44366628..44368101 | |
| GmCML11-3 | | | | Glyma.20G211700 | | 453 | | 150 | 3 | Chr20:44839406..44841287 | |
| GmCML11-4 | | | | Glyma.10G178400 | | 453 | | 150 | 3 | Chr10:41169297..41171844 | |
| GmCML15-1 | | | | Glyma.05G015500 | | 486 | | 161 | 0 | Chr05:1422180..1422665 | |
| GmCML15-2 | | | | Glyma.06G208800 | | 483 | | 160 | 0 | Chr06:20458731..20459213 | |
| GmCML15-3 | | | | Glyma.04G144800 | | 483 | | 160 | 0 | Chr04:26497656..26498138 | |
| GmCML16 | | | | Glyma.16G099600 | | 483 | | 160 | 1 | Chr16:19187944..19189460 | |
| GmCML17 | | | | Glyma.12G089800 | | 492 | | 163 | 0 | Chr12:7377582..7378561 | |
| GmCML18 | | | | Glyma.11G182700 | | 480 | | 159 | 1 | Chr11:25028353..25031056 | |
| GmCML20 | | | | Glyma.15G055100 | | 513 | | 170 | 6 | Chr15:4326815..4329488 | |
| GmCML25 | | | | Glyma.13G083700 | | 474 | | 157 | 0 | Chr13:19442707..19443539 | |
| GmCML27-1 | | | | Glyma.14G215800 | | 426 | | 141 | 0 | Chr14:48030837..48031262 | |
| GmCML27-2 | | | | Glyma.02G245700 | | 426 | | 141 | 0 | Chr02:43391804..43393333 | |
| GmCML27-3 | | | | Glyma.07G101100 | | 540 | | 179 | 0 | Chr07:9597834..9598744 | |
| GmCML27-4 | | | | Glyma.18G039500 | | 420 | | 139 | 0 | Chr18:3218263..3219353 | |
| GmCML27-5 | | | | Glyma.08G053500 | | 543 | | 180 | 0 | Chr08:4137943..4139273 | |
| GmCML30-1 | | | | Glyma.02G133000 | | 558 | | 185 | 0 | Chr02:13760608..13761881 | |
| GmCML30-2 | | | | Glyma.17G175400 | | 573 | | 190 | 0 | Chr17:18331356..18332518 | |
| *Gossypium raimondii* | | | | | | | | | | | |
| GrCML3-1 | | | | Gorai.N002400 | | 459 | | 152 | 0 | scaffold_14: 835625 - 836178 | |
| GrCML3-2 | | | | Gorai.011G142200 | | 651 | | 216 | 0 | Chr11: 22459517 - 22460167 | |
| GrCML3-3 | | | | Gorai.001G227900 | | 459 | | 152 | 0 | Chr01: 46410198 - 46410656 | |
| GrCML5-1 | | | | Gorai.004G276300 | | 681 | | 226 | 0 | Chr04: 60973110 - 60974117 | |
| GrCML5-2 | | | | Gorai.005G128600 | | 453 | | 150 | 0 | Chr05: 30300114 - 30301158 | |
| GrCML5-3 | | | | Gorai.001G044300 | | 792 | | 263 | 0 | Chr01: 4216940 - 4217731 | |
| GrCML7 | | | | Gorai.005G016900 | | 465 | | 154 | 0 | Chr05: 1237021 - 1237485 | |
| GrCML11-1 | | | | Gorai.007G140300 | | 450 | | 149 | 3 | Chr07: 11600617 - 11601607 | |
| GrCML11-2 | | | | Gorai.013G201100 | | 456 | | 151 | 3 | Chr13: 51205771 - 51207197 | |
| GrCML11-3 | | | | Gorai.006G243600 | | 453 | | 150 | 3 | Chr06: 48929839 - 48931576 | |
| GrCML15-1 | | | | Gorai.008G201600 | | 483 | | 160 | 0 | Chr08: 48714982 - 48715464 | |
| GrCML15-2 | | | | Gorai.007G041100 | | 489 | | 162 | 0 | Chr07: 2882173 - 2882987 | |
| GrCML15-3 | | | | Gorai.001G065000 | | 486 | | 161 | 0 | Chr01: 6497767 - 6498252 | |
| GrCML16 | | | | Gorai.008G188600 | | 522 | | 173 | 0 | Chr08: 47059862 - 47061001 | |
| GrCML18-1 | | | | Gorai.004G182700 | | 501 | | 166 | 1 | Chr04: 49598794 - 49599975 | |
| GrCML18-2 | | | | Gorai.003G184700 | | 495 | | 164 | 1 | Chr03: 45546774 - 45547633 | |
| GrCML20-1 | | | | Gorai.003G023300 | | 513 | | 170 | 6 | Chr03: 1797043 - 1799498 | |
| GrCML20-2 | | | | Gorai.011G105500 | | 510 | | 169 | 6 | Chr11: 12249228 - 12251664 | |
| GrCML23-1 | | | | Gorai.007G178400 | | 459 | | 152 | 0 | Chr07: 16599474 - 16599932 | |
| GrCML23-2 | | | | Gorai.001G087100 | | 465 | | 154 | 1 | Chr01: 9301813 - 9305285 | |
| GrCML25-1 | | | | Gorai.009G330300 | | 558 | | 185 | 0 | Chr09: 33933650 - 33934527 | |
| GrCML25-2 | | | | Gorai.004G160800 | | 615 | | 204 | 0 | Chr04: 45084796 - 45085709 | |
| GrCML25-3 | | | | Gorai.002G172400 | | 594 | | 197 | 0 | Chr02: 43781026 - 43782184 | |
| GrCML25-4 | | | | Gorai.005G156600 | | 516 | | 171 | 0 | Chr05: 44335884 - 44336399 | |
| GrCML25-5 | | | | Gorai.008G168100 | | 588 | | 195 | 0 | Chr08: 43928819 - 43929691 | |
| GrCML27-1 | | | | Gorai.007G004000 | | 441 | | 146 | 0 | Chr07: 331306 - 332566 | |
| GrCML27-2 | | | | Gorai.005G131800 | | 492 | | 163 | 0 | Chr05: 32636614 - 32637450 | |
| GrCML27-3 | | | | Gorai.008G289600 | | 465 | | 154 | 0 | Chr08: 56439989 - 56441038 | |
| GrCML30-1 | | | | Gorai.007G212400 | | 573 | | 190 | 0 | Chr07: 23266238 - 23267054 | |
| GrCML30-2 | | | | Gorai.003G023100 | | 555 | | 184 | 0 | Chr03: 1763560 - 1764114 | |
| *Linum usitatissimum* | | | | | | | | | | | |
| LuCML3-1 | | | | Lus10009564.g | | 459 | | 152 | 0 | scaffold1331: 243581 - 244039 | |
| LuCML3-2 | | | | Lus10038088.g | | 726 | | 241 | 0 | scaffold475: 1459819 - 1460544 | |
| LuCML3-3 | | | | Lus10006644.g | | 732 | | 243 | 0 | scaffold345: 27232 - 27963 | |
| LuCML5 | | | | Lus10027701.g | | 690 | | 229 | 0 | scaffold2: 966605 - 967294 | |
| LuCML15-1 | | | | Lus10027243.g | | 477 | | 158 | 0 | scaffold472: 460667 - 461143 | |
| LuCML15-2 | | | | Lus10017900.g | | 504 | | 167 | 0 | scaffold35: 648932 - 649435 | |
| LuCML18-1 | | | | Lus10004610.g | | 516 | | 171 | 0 | scaffold1170: 164698 - 165213 | |
| LuCML18-2 | | | | Lus10030986.g | | 489 | | 162 | 0 | scaffold261: 395977 - 396465 | |
| LuCML24 | | | | Lus10009127.g | | 831 | | 276 | 1 | scaffold1536: 122694 - 124193 | |
| LuCML25-1 | | | | Lus10024574.g | | 441 | | 146 | 1 | scaffold349: 102732 - 103261 | |
| LuCML25-2 | | | | Lus10004330.g | | 459 | | 152 | 0 | scaffold1134: 74662 - 75120 | |
| LuCML25-3 | | | | Lus10028913.g | | 474 | | 157 | 0 | scaffold540: 844170 - 844643 | |
| LuCML27-1 | | | | Lus10018012.g | | 528 | | 175 | 0 | scaffold687: 347946 - 348473 | |
| LuCML27-2 | | | | Lus10031345.g | | 528 | | 175 | 0 | scaffold977: 1271703 - 1272230 | |
| LuCML27-3 | | | | Lus10009059.g | | 528 | | 175 | 0 | scaffold883: 326499 - 327026 | |
| LuCML30-1 | | | | Lus10014050.g | | 573 | | 190 | 0 | scaffold1247: 180894 - 181466 | |
| LuCML30-2 | | | | Lus10019863.g | | 573 | | 190 | 0 | scaffold1491: 401512 - 402084 | |
| LuCML30-3 | | | | Lus10039986.g | | 609 | | 202 | 1 | scaffold12: 983225 - 983915 | |
| LuCML36-1 | | | | Lus10027261.g | | 648 | | 215 | 0 | scaffold472: 537579 - 538226 | |
| LuCML36-2 | | | | Lus10038970.g | | 642 | | 213 | 1 | scaffold34: 1067722 - 1070731 | |
| LuCML38 | | | | Lus10022343.g | | 444 | | 147 | 0 | scaffold225: 800816 - 801259 | |
| *Malus domestica* | | | | | | | | | | | |
| MdCML3-1 | | | | MDP0000535637 | | 660 | | 219 | 0 | MDC003523.387: 404 - 1063 | |
| MdCML3-2 | | | | MDP0000817646 | | 459 | | 152 | 0 | MDC000962.743: 16567 - 17025 | |
| MdCML3-4 | | | | MDP0000418827 | | 660 | | 219 | 0 | MDC016069.206: 6127 - 6786 | |
| MdCML11 | | | | MDP0000403476 | | 567 | | 188 | 4 | MDC003816.1052: 13834 - 16842 | |
| MdCML15-1 | | | | MDP0000602146 | | 483 | | 160 | 0 | MDC013824.133: 14803 - 15285 | |
| MdCML15-2 | | | | MDP0000311043 | | 483 | | 160 | 0 | MDC020136.407: 68287 - 68769 | |
| MdCML17-1 | | | | MDP0000328568 | | 597 | | 198 | 6 | MDC022192.328: 15369 - 16973 | |
| MdCML17-2 | | | | MDP0000859609 | | 489 | | 162 | 0 | MDC020006.134: 6614 - 7102 | |
| MdCML17-3 | | | | MDP0000190637 | | 468 | | 155 | 0 | MDC010403.411: 34209 - 34676 | |
| MdCML18 | | | | MDP0000864163 | | 492 | | 163 | 0 | MDC000741.237: 14130 - 14621 | |
| MdCML20 | | | | MDP0000216112 | | 510 | | 169 | 6 | MDC022192.320: 3519 - 5796 | |
| MdCML25-1 | | | | MDP0000250594 | | 561 | | 186 | 1 | MDC010996.317: 1321 - 1930 | |
| MdCML25-2 | | | | MDP0000240032 | | 474 | | 157 | 0 | MDC009965.40: 4854 - 5327 | |
| MdCML25-3 | | | | MDP0000587060 | | 750 | | 249 | 1 | MDC002552.263: 15892 - 16969 | |
| MdCML25-4 | | | | MDP0000140151 | | 609 | | 202 | 0 | MDC014172.222: 603 - 1211 | |
| MdCML27-1 | | | | MDP0000143036 | | 480 | | 159 | 0 | MDC020883.179: 905 - 1384 | |
| MdCML27-2 | | | | MDP0000330029 | | 579 | | 192 | 0 | MDC013646.294: 34425 - 34997 | |
| MdCML27-3 | | | | MDP0000780674 | | 528 | | 175 | 0 | MDC013646.298: 8417 - 8944 | |
| MdCML27-5 | | | | MDP0000586415 | | 465 | | 154 | 0 | MDC004754.345: 5223 - 5687 | |
| MdCML27-6 | | | | MDP0000834180 | | 465 | | 154 | 0 | MDC005169.268: 14011 - 14475 | |
| MdCML30-1 | | | | MDP0000606583 | | 555 | | 184 | 0 | MDC011373.250: 26343 - 26897 | |
| MdCML30-2 | | | | MDP0000187969 | | 558 | | 185 | 0 | MDC009254.293: 5644 - 6201 | |
| MdCML30-3 | | | | MDP0000215758 | | 573 | | 190 | 0 | MDC022191.207: 14489 - 15061 | |
| MdCML36-1 | | | | MDP0000859814 | | 672 | | 223 | 0 | MDC014107.433: 489 - 1160 | |
| MdCML36-2 | | | | MDP0000294840 | | 660 | | 219 | 0 | MDC015029.291: 961 - 1620 | |
| MdCML36-3 | | | | MDP0000870230 | | 867 | | 288 | 1 | MDC021135.127: 2058 - 2998 | |
| MdCML36-4 | | | | MDP0000146799 | | 846 | | 281 | 0 | MDC001754.725: 14232 - 15077 | |
| MdCML38 | | | | MDP0000545337 | | 423 | | 140 | 0 | MDC007389.248: 38446 - 38868 | |
| MdCLM39-1 | | | | MDP0000853812 | | 423 | | 140 | 0 | MDC007389.240: 8978 - 9400 | |
| MdCML39-2 | | | | MDP0000222664 | | 423 | | 140 | 0 | MDC019739.241: 20935 - 21357 | |
| MdCML41-1 | | | | MDP0000157748 | | 582 | | 193 | 0 | MDC001065.206: 2242 - 2823 | |
| MdCML41-2 | | | | MDP0000157748 | |  | |  |  |  | |
| *Manihot esculenta* | | | | | | | | | | | |
| MeCML3-1 | | | | cassava4.1_015318m.g | | 699 | | 232 | 0 | scaffold05297: 129328 - 130026 | |
| MeCML3-2 | | | | cassava4.1_018286m.g | | 459 | | 152 | 0 | scaffold08314: 92626 - 93499 | |
| MeCML3-3 | | | | cassava4.1_018287m.g | | 459 | | 152 | 0 | scaffold06158: 57869 - 58492 | |
| MeCML5 | | | | cassava4.1_015160m.g | | 711 | | 236 | 0 | scaffold05760: 60466 - 61530 | |
| MeCML11-1 | | | | cassava4.1_018359m.g | | 453 | | 150 | 3 | scaffold10563: 306849 - 308082 | |
| MeCML11-2 | | | | cassava4.1_018372m.g | | 453 | | 150 | 3 | scaffold01551: 1152248 - 1153179 | |
| MeCML15 | | | | cassava4.1_025234m.g | | 510 | | 169 | 0 | scaffold00889: 248519 - 249028 | |
| MeCML16-1 | | | | cassava4.1_034063m.g | | 489 | | 162 | 0 | scaffold06656: 323419 - 323907 | |
| MeCML16-2 | | | | cassava4.1_032271m.g | | 486 | | 161 | 0 | scaffold08359: 683678 - 684163 | |
| MeCML18 | | | | cassava4.1_017935m.g | | 492 | | 163 | 1 | scaffold03614: 695832 - 696791 | |
| MeCML20-1 | | | | cassava4.1_017704m.g | | 510 | | 169 | 6 | scaffold08639: 42455 - 45008 | |
| MeCML20-2 | | | | cassava4.1_032745m.g | | 528 | | 176 | 5 | scaffold03060: 168474 - 170042 | |
| MeCML23 | | | | cassava4.1_017956m.g | | 492 | | 163 | 0 | scaffold06871: 43403 - 44191 | |
| MeCML25-1 | | | | cassava4.1_016892m.g | | 579 | | 192 | 0 | scaffold10173: 950512 - 951336 | |
| MeCML25-2 | | | | cassava4.1_033144m.g | | 579 | | 192 | 0 | scaffold08847: 278865 - 279443 | |
| MeCML27-1 | | | | cassava4.1_017854m.g | | 498 | | 165 | 0 | scaffold06631: 376824 - 377565 | |
| MeCML27-2 | | | | cassava4.1_017617m.g | | 519 | | 172 | 0 | scaffold02572: 297776 - 298485 | |
| MeCML30-1 | | | | cassava4.1_031346m.g | | 558 | | 185 | 0 | scaffold11242: 132964 - 133521 | |
| MeCML30-2 | | | | cassava4.1_017145m.g | | 558 | | 185 | 0 | scaffold03175: 27880 - 28623 | |
| MeCML30-3 | | | | cassava4.1_017248m.g | | 549 | | 182 |  | scaffold07300: 12461 - 13009 | |
| MeCML32 | | | | cassava4.1_016872m.g | | 582 | | 193 | 3 | scaffold03481: 216136 - 218632 | |
| MeCML36 | | | | cassava4.1_015467m.g | | 690 | | 229 | 0 | scaffold06890: 195129 - 196210 | |
| *Medicago truncatula* | | | | | | | | | | | |
| MtCML3 | | | | Medtr7g089760 | | 459 | | 152 | 0 | chr7: 27952223 - 27952681 | |
| MtCML5 | | | | Medtr2g098890 | | 636 | | 211 | 0 | chr2: 31087335 - 31088709 | |
| MtCML7 | | | | Medtr4g115170 | | 681 | | 226 | 0 | chr4: 39747067 - 39748090 | |
| MtCML11 | | | | Medtr4g122260 | | 492 | | 163 | 0 | chr4: 42264661 - 42265421 | |
| MtCML15 | | | | Medtr4g112460 | | 510 | | 169 | 0 | chr4: 38142255 - 38142764 | |
| MtCML18 | | | | Medtr8g066630 | | 495 | | 164 | 1 | chr8: 16717505 - 16719185 | |
| MtCML20 | | | | Medtr7g074020 | | 516 | | 171 | 6 | chr7: 19737118 - 19739928 | |
| MtCML21 | | | | Medtr8g070510 | | 684 | | 227 | 3 | chr8: 18299008 - 18302072 | |
| MtCML24 | | | | Medtr5g079340 | | 420 | | 139 | 0 | chr5: 32913864 - 32914810 | |
| MtCML25-1 | | | | Medtr7g075040 | | 588 | | 195 | 0 | chr7: 20253312 - 20253899 | |
| MtCML25-2 | | | | Medtr5g027000 | | 477 | | 158 | 0 | chr5: 10940339 - 10940815 | |
| MtCML25-3 | | | | Medtr8g105230 | | 567 | | 188 | 0 | chr8: 31193866 - 31194432 | |
| MtCML25-4 | | | | Medtr1g032070 | | 597 | | 198 | 0 | chr1:11350123..11350719 | |
| MtCML27 | | | | Medtr8g078270 | | 567 | | 188 | 0 | chr8: 21867420 - 21868178 | |
| MtCML30-1 | | | | Medtr5g017550 | | 567 | | 188 | 0 | chr5: 6152691 - 6153402 | |
| MtCML30-2 | | | | Medtr5g017510 | | 570 | | 189 | 0 | chr5: 6142322 - 6142891 | |
| MtCML30-3 | | | | Medtr5g017510 | | 570 | | 189 | 0 | chr5:6399203..6400045 | |
| MtCML36 | | | | Medtr7g090450 | | 651 | | 216 | 0 | chr7: 28335054 - 28336139 | |
| MtCML38-1 | | | | Medtr3g089090 | | 420 | | 139 | 0 | chr3: 29778940 - 29780054 | |
| MtCML38-2 | | | | Medtr3g089070 | | 423 | | 140 | 0 | chr3: 29772174 - 29772974 | |
| MtCML38-3 | | | | Medtr4g103630 | | 423 | | 140 | 0 | chr4: 35873386 - 35873808 | |
| MtCML41-1 | | | | Medtr8g078270 | | 567 | | 188 | 0 | chr8:33366028..33366970 | |
| MtCML41-2 | | | | Medtr6g023460 | | 429 | | 142 | 0 | chr6: 5307128 - 5307556 | |
| MtCML46 | | | | Medtr4g127560 | | 759 | | 252 | 2 | chr4: 44366324 - 44370817 | |
| *Micromonas pusilla* | | | | | | | | | | | |
| MpCML4 | | | | MicpuC2.e_gw1.6.402.1 | | | 708 | 235 | 1 | scaffold_6: 120523 - 121430 | |
| MpCML11 | | | | estExt_Genewise1Plus.C_15_t10399 | | | 501 | 166 | 0 | scaffold_15: 506208 - 506801 | |
| MpCML12 | | | | MicpuC2.fgenesh1_pg.C_scaffold_12000259 | | | 615 | 204 | 1 | scaffold_12: 818673 - 819433 | |
| MpCML15 | | | | MicpuC2.gw1.12.373.1 | | | 459 | 153 | 1 | scaffold_12: 63234 - 63734 | |
| MpCML20-1 | | | | estExt_Genewise1Plus.C_5_t30101 | | | 525 | 174 | 1 | scaffold_5: 1154962 - 1155823 | |
| MpCML20-2 | | | | MicpuC2.estExt_fgenesh1_kg.C_30020 | | | 498 | 165 | 1 | scaffold_3: 943822 - 945420 | |
| MpCML21 | | | | estExt_fgenesh2_kg.C_90079 | | | 636 | 211 | 0 | scaffold_9: 771281 - 772035 | |
| MpCML30 | | | | e_gw1.15.619.1 | | | 504 | 168 | 1 | scaffold_15: 199751 - 200305 | |
| *Mimulus guttatus* | | | | | | | | | | | |
| MgCML3-1 | | | | Migut.N00671 | | | 468 | 155 | 0 | scaffold_14:3526345..3526812 | |
| MgCML3-2 | | | | Migut.G01083 | | | 687 | 28 | 0 | scaffold_7:13103207..13104419 | |
| MgCML4 | | | | Migut.K00428 | | | 627 | 208 | 0 | scaffold_11:2079258..2080012 | |
| MgCML7 | | | | Migut.D00745 | | | 465 | 154 | 0 | scaffold_4:3620650..3622059 | |
| MgMCL16 | | | | Migut.E00277 | | | 486 | 161 | 0 | scaffold_5:1442414..1443400 | |
| MgCML18 | | | | Migut.E00757 | | | 498 | 165 | 1 | scaffold_5:4981174..4982943 | |
| MgCML21 | | | | Migut.H01719 | | | 690 | 229 | 4 | scaffold_8:17556793..17558890 | |
| MgCML22 | | | | Migut.N00380 | | | 735 | 244 | 4 | scaffold_14:1820686..1822752 | |
| MgCML25-1 | | | | Migut.E00473 | | | 2055 | 684 | 5 | scaffold_5:2611944..2617375 | |
| MgCML25-2 | | | | Migut.B01645 | | | 468 | 155 | 0 | scaffold_2:17684703..17685436 | |
| MgCML27-1 | | | | Migut.I00446 | | | 507 | 168 | 0 | scaffold_9:9772532..9773557 | |
| MgCML27-2 | | | | Migut.H00277 | | | 459 | 152 | 0 | scaffold_8:1555070..1556006 | |
| MgCML30 | | | | Migut.B00343 | | | 552 | 183 | 0 | scaffold_2:1595685..1596498 | |
| MgCML36-1 | | | | Migut.K00111 | | | 630 | 209 | 1 | scaffold_11:524513..525602 | |
| MgCML36-2 | | | | Migut.J01687 | | | 675 | 224 | 0 | scaffold_10:18189272..18189946 | |
| MgCML38-1 | | | | Migut.K01451 | | | 429 | 142 | 0 | scaffold_11:24010938..24011734 | |
| MgCML38-2 | | | | Migut.K01450 | | | 429 | 142 | 0 | scaffold_11:24006305..24006840 | |
| MgCML38-3 | | | | Migut.F00079 | | | 417 | 138 | 0 | scaffold_6:408896..409312 | |
| MgCML42 | | | | Migut.J00006 | | | 597 | 198 | 0 | scaffold_10:32465..33533 | |
| *Oryza sativa* | | | | | | | | | | | |
| OsCML1 | | | | LOC_Os01g59530 | | | 564 | 188 | 2 | Chr1: 34420990 - 34423678 | |
| OsCML2 | | | | LOC_Os11g03980 | | | 552 | 184 | 2 | Chr11: 1610131 - 1609280 | |
| OsCML3 | | | | LOC_Os12g03816 | | | 552 | 184 | 2 | Chr12: 1561568 - 1559859 | |
| OsCML4 | | | | LOC_Os03g53200 | | | 465 | 155 | 3 | Chr3: 30517536 - 30520559 | |
| OsCML5 | | | | LOC_Os12g41110 | | | 501 | 167 | 0 | Chr12: 25462589 - 25463518 | |
| OsCML6 | | | | LOC_Os11g37550 | | | 513 | 171 | 4 | Chr11: 22174756 - 22173476 | |
| OsCML7 | | | | [LOC_Os08g](http://rice.plantbiology.msu.edu/cgi-bin/ORF_infopage.cgi?orf=LOC_Os05g05460.1)02420 | | | 447 | 149 | 0 | Chr8: 970545 - 969281 | |
| OsCML8 | | | | LOC_Os10g25010 | | | 576 | 192 | 6 | Chr10: 12874087 - 12870885 | |
| OsCML9 | | | | LOC_Os05g41200 | | | 468 | 156 | 1 | Chr5: 24131120 - 24130113 | |
| OsCML10 | | | | LOC_Os01g72100 | | | 558 | 186 | 0 | Chr1: 41827156 - 41825882 | |
| OsCML11 | | | | LOC_Os01g32120 | | | 636 | 212 | 0 | Chr1: 17581113 - 17582133 | |
| OsCML12 | | | | LOC_Os01g41990 | | | 750 | 250 | 0 | Chr1: 23811557 - 23810148 | |
| OsCML14 | | | | LOC_Os05g50180 | | | 522 | 174 | 0 | Chr5: 28758694 - 28757904 | |
| OsCML15 | | | | LOC_Os05g31620 | | | 606 | 202 | 0 | Chr5: 18407458 - 18406480 | |
| OsCML16 | | | | LOC_Os01g04330 | | | 546 | 182 | 0 | Chr1: 1931904 - 1930994 | |
| OsCML17 | | | | LOC_Os02g39380 | | | 495 | 165 | 0 | Chr2: 23767449 - 23766605 | |
| OsCML18 | | | | LOC_Os05g13580 | | | 477 | 159 | 0 | Chr5: 7534664 - 7533633 | |
| OsCML19 | | | | LOC_Os01g72550 | | | 441 | 147 | 0 | Chr1: 42080116 - 42080556 | |
| OsCML20 | | | | LOC_Os02g50060 | | | 525 | 175 | 0 | Chr2: 30580869 - 30579808 | |
| OsCML21 | | | | LOC_Os05g24780 | | | 594 | 198 | 0 | Chr5: 14361588 - 14360734 | |
| OsCML22 | | | | LOC_Os04g41540 | | | 753 | 251 | 0 | Chr4: 24640382 - 24641575 | |
| OsCML23 | | | | LOC_Os01g72540 | | | 456 | 152 | 0 | Chr1: 42079137 - 42078682 | |
| OsCML24 | | | | LOC_Os07g48340 | | | 594 | 198 | 1 | Chr7: 28889240 - 28892030 | |
| OsCML25 | | | | LOC_Os11g01390 | | | 450 | 150 | 0 | Chr11: 245637 - 245188 | |
| OsCML26 | | | | LOC_Os12g01400 | | | 450 | 150 | 0 | Chr12: 261030 - 260311 | |
| OsCML27 | | | | LOC_Os03g21380 | | | 573 | 191 | 0 | Chr3: 12226100 - 12223886 | |
| OsCML28 | | | | LOC_Os12g12730 | | | 519 | 173 | 0 | Chr12: 7014840 - 7014179 | |
| OsCML29 | | | | [LOC_Os06g40720](http://rice.plantbiology.msu.edu/cgi-bin/ORF_infopage.cgi?orf=LOC_Os06g40720.1) | | | 675 | 225 | 4 | Chr6: 24275956 - 24273302 | |
| OsCML30 | | | | LOC_Os06g07560 | | | 711 | 237 | 0 | Chr6: 3649517 - 3650806 | |
| OsCML31 | | | | LOC_Os01g72530 | | | 456 | 152 | 0 | Chr1: 42074982 - 42073984 | |
| OsCML32 | | | | LOC_Os08g04890 | | | 591 | 197 | 0 | Chr8: 2495070 - 2496026 | |
| OsCML33 | | | | [LOC_Os02g10470](http://rice.plantbiology.msu.edu/cgi-bin/ORF_infopage.cgi?orf=LOC_Os02g10470.1) | | | 687 | 229 | 4 | Chr2: 5501165 - 5504381 | |
| OsCML34 | | | | [LOC_Os05g05460](http://rice.plantbiology.msu.edu/cgi-bin/ORF_infopage.cgi?orf=LOC_Os05g05460.1) | | | 750 | 250 | 3 | Chr5: 2718310 - 2716143 | |
| *Ostreococcus lucimarinus* | | | | | | | | | | | |
| OlCML11 | | | | gwEuk.7.585.1 | | | 393 | 131 | 0 | Chr_7:597092..597484 | |
| OlCML20 | | | | eugene.0500010299 | | | 492 | 163 | 1 | Chr_5:496282..497461 | |
| *Panicum hallii* | | | | | | | | | | | |
| PhCML4 | | | | Pahal.I01776 | | | 465 | 154 | 3 | Chr_09:13638058..13640581 | |
| PhCML5 | | | | Pahal.C04675 | | | 492 | 163 | 0 | Chr_03:62943188..62944041 | |
| PhCML6 | | | | Pahal.B03080 | | | 564 | 187 | 0 | Chr_02:54094796..54095882 | |
| PhCML8-1 | | | | Pahal.B04677 | | | 513 | 170 | 6 | Chr_02:66681056..66683249 | |
| PhCML8-2 | | | | Pahal.I03392 | | | 534 | 177 | 6 | Chr_09:31221526..31224066 | |
| PhCML10 | | | | Pahal.C00880 | | | 528 | 175 | 1 | Chr_03:5433427..5435201 | |
| PhCML11 | | | | Pahal.C01834 | | | 540 | 179 | 0 | Chr_03:11570911..11571736 | |
| PhCML12 | | | | Pahal.E02472 | | | 675 | 224 | 0 | Chr_05:14963475..14964149 | |
| PhCML14 | | | | Pahal.C01834 | | | 540 | 179 | 0 | Chr_03:11570911..11571736 | |
| PhCML16 | | | | Pahal.E03871 | | | 570 | 189 | 0 | Chr_05:44424425..44424994 | |
| PhCML17 | | | | Pahal.G00725 | | | 732 | 243 | 0 | Chr_07:4642780..4643784 | |
| PhCML18 | | | | Pahal.C00880 | | | 528 | 175 | 1 | Chr_03:5433427..5435201 | |
| PhCML19 | | | | Pahal.E00233 | | | 435 | 144 | 0 | Chr_05:1361336..1361770 | |
| PhCML20 | | | | Pahal.A03341 | | | 543 | 180 | 0 | Chr_01:46394705..46395247 | |
| PhCML28 | | | | Pahal.C00744 | | | 480 | 159 | 0 | Chr_03:4648287..4649471 | |
| PhCML30 | | | | Pahal.F02277 | | | 738 | 245 | 1 | Chr_06:25918360..25919725 | |
| PhCML31 | | | | Pahal.I04118 | | | 459 | 152 | 1 | Chr_09:45257991..45259292 | |
| *Panicum virgatum* | | | | | | | | | | | |
| PvCML4 | | | | Pavir.J36101 | | | 465 | 154 | 3 | contig57712:1..2057 | |
| PvCML5-1 | | | | Pavir.Cb00254 | | | 483 | 160 | 1 | Chr03b:4171870..4172933 | |
| PvCML5-2 | | | | Pavir.J21570 | | | 495 | 164 | 1 | contig234992:508..1443 | |
| PvCML8-1 | | | | Pavir.Ia02639 | | | 534 | 177 | 6 | Chr09a:51126390..51130606 | |
| PvCML8-2 | | | | Pavir.J35616 | | | 513 | 170 | 6 | contig54316:2400..4345 | |
| PvCML8-3 | | | | Pavir.Ib02905 | | | 534 | 177 | 6 | Chr09b:43227213..43229227 | |
| PvCML10-1 | | | | Pavir.Eb03899 | | | 564 | 187 | 0 | Chr05b:74918951..74920565 | |
| PvCML10-2 | | | | Pavir.J31351 | | | 489 | 162 | 0 | contig370980:664..1152 | |
| PvCML10-3 | | | | Pavir.J18221 | | | 576 | 191 | 0 | contig19998:14..589 | |
| PvCML12 | | | | Pavir.Eb02092 | | | 663 | 220 | 1 | Chr05b:40582743..40594254 | |
| PvCML14-1 | | | | Pavir.Ca00981 | | | 546 | 181 | 0 | Chr03a:10777705..10778662 | |
| PvCML14-2 | | | | Pavir.J02745 | | | 549 | 182 | 0 | contig03283:17956..18504 | |
| PvCML15 | | | | Pavir.Cb01421 | | | 609 | 202 | 0 | Chr03b:35320202..35321193 | |
| PvCML16 | | | | Pavir.Ea00612 | | | 579 | 192 | 0 | Chr05a:8023870..8025674 | |
| PvCML18-1 | | | | Pavir.Cb01740 | | | 522 | 173 | 1 | Chr03b:43109293..43110537 | |
| PvCML18-2 | | | | Pavir.J35809 | | | 525 | 174 | 0 | contig55596:2931..4139 | |
| PvCML28-1 | | | | Pavir.Ca00383 | | | 480 | 159 | 0 | Chr03a:4841258..4841737 | |
| PvCML28-2 | | | | Pavir.Cb01831 | | | 480 | 159 | 0 | Chr03b:43732220..43732699 | |
| PvCML29 | | | | Pavir.J23152 | | | 522 | 173 | 2 | contig252786:893..1789 | |
| PvCML36 | | | | Pavir.J19247 | | | 759 | 252 | 5 | contig209819:168..2172 | |
| *Phaseolus vulgaris* | | | | | | | | | | | |
| PvulCML3-1 | | | | Phvul.003G168200 | | 684 | | 227 | 0 | Chr03:37785829..37786512 | |
| PvulCML3-2 | | | | Phvul.005G152900 | | 630 | | 209 | 0 | Chr05:37989265..37990592 | |
| PvulCML3-3 | | | | Phvul.011G054100 | | 453 | | 150 | 0 | Chr11:4583824..4584276 | |
| PvulCML3-4 | | | | Phvul.001G122800 | | 453 | | 150 | 0 | Chr01:34529753..34530716 | |
| PvulCML11 | | | | Phvul.006G101200 | | 450 | | 149 | 3 | Chr06:21833976..21835032 | |
| PvulCML15 | | | | Phvul.009G201700 | | 537 | | 178 | 1 | Chr09:29861786..29863978 | |
| PvulCML16 | | | | Phvul.003G281700 | | 483 | | 160 | 0 | Chr03:50747207..50747689 | |
| PvulCML18 | | | | Phvul.003G283800 | | 492 | | 163 | 0 | Chr03:50989628..50990460 | |
| PvulCML20 | | | | Phvul.006G204800 | | 513 | | 170 | 6 | Chr06:30785818..30787886 | |
| PvulCML25-1 | | | | Phvul.010G085100 | | 639 | | 212 | 0 | Chr10:31707286..31708492 | |
| PvulCML25-2 | | | | Phvul.003G019600 | | 579 | | 192 | 0 | Chr03:1738789..1739771 | |
| PvulCML25-3 | | | | Phvul.008G167700 | | 474 | | 157 | 0 | Chr08:43401090..43401951 | |
| PvulCML25-4 | | | | Phvul.002G320800 | | 567 | | 188 | 0 | Chr02:48035445..48036011 | |
| PvulCML27-1 | | | | Phvul.008G235100 | | 423 | | 140 | 1 | Chr08:54898350..54899305 | |
| PvulCML27-2 | | | | Phvul.001G231000 | | 420 | | 139 | 1 | Chr01:49218669..49221075 | |
| PvulCML27-3 | | | | Phvul.L002000 | | 534 | | 177 | 0 | scaffold_24:9100..9633 | |
| PvulCML27-4 | | | | Phvul.002G329300 | | 519 | | 172 | 0 | Chr02:48782930..48784258 | |
| PvulCML30-1 | | | | Phvul.003G292700 | | 558 | | 185 | 0 | Chr03:51850091..51850794 | |
| PvulCML30-2 | | | | Phvul.008G031800 | | 558 | | 185 | 2 | Chr08:2633636..2635298 | |
| PvulCML30-3 | | | | Phvul.002G019300 | | 567 | | 188 | 0 | Chr02:2129877..2130681 | |
| PvulCML38-1 | | | | Phvul.005G026100 | | 426 | | 141 | 0 | Chr05:2361402..2362044 | |
| PvulCML38-2 | | | | Phvul.005G026000 | | 432 | | 143 | 0 | Chr05:2358886..2359691 | |
| PvulCML38-3 | | | | Phvul.003G210000 | | 423 | | 140 | 0 | Chr03:42489216..42489941 | |
| PvulCML38-4 | | | | Phvul.004G055200 | | 426 | | 141 | 0 | Chr04:7183009..7183700 | |
| PvulCML38-5 | | | | Phvul.001G095600 | | 423 | | 140 | 0 | Chr01:20789706..20790128 | |
| PvulCML41 | | | | Phvul.003G251000 | | 585 | | 194 | 0 | Chr03:47811418..47812002 | |
| *Physcomitrella patens* | | | | | | | | | | | |
| PpCML7 | | | | Phpat.016G094500 | | 642 | | 213 | 0 | Chr16:15273996..15275887 | |
| PpCML17-1 | | | | Phpat.007G067000 | | 603 | | 200 | 0 | Chr07:11521169..11523008 | |
| PpCML17-2 | | | | Phpat.011G033000 | | 531 | | 176 | 0 | Chr11:5795609..5796783 | |
| PpCML21 | | | | Phpat.006G081800 | | 750 | | 249 | 4 | Chr06:14193480..14196246 | |
| PpCML23 | | | | Phpat.006G086400 | | 756 | | 251 | 0 | Chr06:15482698..15484434 | |
| PpCML25-1 | | | | Phpat.025G037100 | | 573 | | 190 | 0 | Chr25:7433357..7434698 | |
| PpCML25-2 | | | | Phpat.012G020900 | | 585 | | 194 | 0 | Chr12:4525799..4528325 | |
| PpCML25-3 | | | | Phpat.006G086200 | | 735 | | 244 | 0 | Chr06:15468571..15470020 | |
| PpCML25-4 | | | | Phpat.009G095400 | | 588 | | 195 | 0 | Chr09:17036015..17036602 | |
| PpCML25-5 | | | | Phpat.016G092800 | | 537 | | 178 | 0 | Chr16:14548749..14550004 | |
| PpCML25-6 | | | | Phpat.005G042100 | | 711 | | 236 | 0 | Chr05:7765920..7767755 | |
| PpCML25-7 | | | | Phpat.006G006800 | | 564 | | 187 | 0 | Chr06:899902..901231 | |
| PpCML25-8 | | | | Phpat.005G044100 | | 435 | | 144 | 0 | Chr05:8200428..8200862 | |
| PpCML25-9 | | | | Phpat.005G044200 | | 435 | | 144 | 0 | Chr05:8238109..8238543 | |
| PpCML25-10 | | | | Phpat.003G023000 | | 513 | | 170 | 0 | Chr03:3756818..3757896 | |
| PpCML25-11 | | | | Phpat.013G007900 | | 558 | | 185 | 0 | Chr13:1471564..1473257 | |
| PpCML25-12 | | | | Phpat.013G007600 | | 624 | | 207 | 0 | Chr13:1463522..1465071 | |
| *Picea abies* | | | | | | | | | | | |
| PaCML5-1 | | | | MA_10431273g0010 | | 459 | | 153 | 0 | MA_10431273:1585..2083 | |
| PaCML5-2 | | | | MA_10289213g0010 | | 504 | | 167 | 0 | MA_10289213:3277...3820 | |
| PaCML5-3 | | | | MA_393053g0010 | | 456 | | 151 | 0 | MA_393053:2759...3254 | |
| PaCAM19 | | | | MA_10207337g0010 | | 489 | | 162 | 0 | MA_10207337:1300...1828 | |
| PaCML21 | | | | MA_10436115g0010 | | 606 | | 201 | 3 | MA_10436115:11447...20432 | |
| PaCML25-1 | | | | MA_51891g0010 | | 738 | | 245 | 0 | MA_51891:5922...6699 | |
| PaCML25-2 | | | | MA_194997g0010 | | 636 | | 211 | 0 | MA_194997:3716...4391 | |
| PaCML25-3 | | | | MA_12364g0010 | | 657 | | 218 | 0 | MA_12364:53475...54171 | |
| PaCML25-4 | | | | MA_8414519g0010 | | 672 | | 223 | 0 | MA_8414519:1043...1754 | |
| PaCML25-5 | | | | MA_52212g0010 | | 675 | | 224 | 0 | MA_52212:8168...8882 | |
| PaCML25-6 | | | | MA_41536g0010 | | 549 | | 182 | 1 | MA_41536:14136...16238 | |
| PaCML25-7 | | | | MA_76703g0020 | | 549 | | 182 | 1 | MA_76703:10514...12429 | |
| PaCML25-8 | | | | MA_456498g0010 | | 585 | | 194 | 0 | MA_456498:1644...2268 | |
| PaCML42-1 | | | | MA_13199g0010 | | 582 | | 193 | 0 | MA_13199:24483...25104 | |
| PaCML42-2 | | | | MA_103558g0010 | | 594 | | 197 | 0 | MA_103558:728...1361 | |
| *Populus trichocarpa* | | | | | | | | | | | |
| PtCML3-1 | | | | Potri.017G029700 | | 672 | | 223 | 0 | Chr17:2602274..2603659 | |
| PtCML3-2 | | | | Potri.T124900 | | 672 | | 223 | 0 | scaffold_219:63391..64187 | |
| PtCML3-3 | | | | Potri.006G065900 | | 711 | | 236 | 0 | Chr06:4883386..4884654 | |
| PtCML3-4 | | | | Potri.018G127100 | | 708 | | 235 | 1 | Chr18:14930455..14931868 | |
| PtCML3-5 | | | | Potri.002G239100 | | 459 | | 152 | 0 | Chr02:23213475..23213933 | |
| PtCML5 | | | | Potri.007G128600 | | 888 | | 295 | 1 | Chr07:14456363..14458527 | |
| PtCML11-1 | | | | Potri.013G040300 | | 450 | | 149 | 4 | Chr13:2800317..2810665 | |
| PtCML11-2 | | | | Potri.002G047300 | | 462 | | 153 | 3 | Chr02:3034969..3036079 | |
| PtCML11-3 | | | | Potri.005G215700 | | 450 | | 149 | 3 | Chr05:22883521..22884841 | |
| PtCML15 | | | | Potri.015G052600 | | 483 | | 160 | 0 | Chr15:6778068..6778550 | |
| PtCML16 | | | | Potri.010G132800 | | 483 | | 160 | 0 | Chr10:14753229..14753711 | |
| PtCML18-1 | | | | Potri.003G095700 | | 495 | | 164 | 2 | Chr03:12235645..12237925 | |
| PtCML18-2 | | | | Potri.001G138000 | | 495 | | 164 | 0 | Chr01:11110532..11111182 | |
| PtCML20 | | | | Potri.005G138000 | | 510 | | 169 | 6 | Chr05:11428638..11431930 | |
| PtCML23-1 | | | | Potri.017G126200 | | 474 | | 157 | 0 | Chr17:13823215..13824126 | |
| PtCML23-2 | | | | Potri.004G089400 | | 627 | | 208 | 1 | Chr04:7589229..7591084 | |
| PtCML25-1 | | | | Potri.010G107100 | | 594 | | 197 | 0 | Chr10:12692251..12693383 | |
| PtCML25-2 | | | | Potri.008G134300 | | 588 | | 195 | 0 | Chr08:8927495..8928413 | |
| PtCML25-3 | | | | Potri.002G088500 | | 498 | | 165 | 0 | Chr02:6282533..6283783 | |
| PtCML27-1 | | | | Potri.014G070700 | | 627 | | 208 | 0 | Chr14:5734197..5735847 | |
| PtCML27-2 | | | | Potri.015G039500 | | 525 | | 174 | 0 | Chr15:3611887..3612791 | |
| PtCML27-3 | | | | Potri.012G048200 | | 492 | | 163 | 0 | Chr12:4492175..4493247 | |
| PtCML30-1 | | | | Potri.009G102500 | | 558 | | 185 | 1 | Chr09:9027354..9028186 | |
| PtCML30-2 | | | | Potri.007G042900 | | 615 | | 204 | 0 | Chr07:3673748..3674736 | |
| PtCML37 | | | | Potri.002G132500 | | 423 | | 140 | 0 | Chr02:9879443..9879948 | |
| PtCML41 | | | | Potri.007G031900 | | 567 | | 188 | 0 | Chr07:2426677..2428139 | |
| *Prunus persica* | | | | | | | | | | | |
| PperCML5-1 | | | | Prupe.1G194800 | | 681 | | 226 | 1 | Pp01:18428468..18429235 | |
| PperCML5-2 | | | | Prupe.7G248200 | | 693 | | 230 | 0 | Pp07:21063801..21064493 | |
| PperCML11-1 | | | | Prupe.1G023600 | | 486 | | 161 | 3 | Pp01:1659923..1662734 | |
| PperCML11-2 | | | | Prupe.6G342600 | | 465 | | 154 | 3 | Pp06:29522683..29524333 | |
| PperCML11-3 | | | | Prupe.8G234000 | | 447 | | 148 | 3 | Pp08:20741871..20743397 | |
| PperCML12 | | | | Prupe.1G007100 | | 756 | | 251 | 4 | Pp01:594535..597085 | |
| PperCML15 | | | | Prupe.5G214100 | | 483 | | 160 | 0 | Pp05:16882765..16883858 | |
| PperCML16 | | | | Prupe.1G301400 | | 477 | | 158 | 0 | Pp01:29633634..29634840 | |
| PperCML18 | | | | Prupe.5G029500 | | 492 | | 163 | 2 | Pp05:3459746..3462801 | |
| PperCML20 | | | | Prupe.7G133900 | | 510 | | 169 | 6 | Pp07:15206597..15208484 | |
| PperCML21 | | | | Prupe.6G033600 | | 684 | | 227 | 4 | Pp06:2550741..2554143 | |
| PperCML25-1 | | | | Prupe.5G232300 | | 513 | | 170 | 0 | Pp05:17686526..17688673 | |
| PperCML25-2 | | | | Prupe.2G146900 | | 618 | | 205 | 0 | Pp02:20317958..20319271 | |
| PperCML25-3 | | | | Prupe.1G505300 | | 474 | | 157 | 0 | Pp01:41737613..41738432 | |
| PperCML27-2 | | | | Prupe.3G160600 | | 486 | | 161 | 1 | Pp03:17937811..17939413 | |
| PperCML27-1 | | | | Prupe.5G232300 | | 513 | | 170 | 0 | Pp05:17686526..17688673 | |
| PperCML30-1 | | | | Prupe.8G123900 | | 567 | | 188 | 0 | Pp08:14961350..14962069 | |
| PperCML30-2 | | | | Prupe.7G134500 | | 570 | | 189 | 0 | Pp07:15239842..15240732 | |
| PperCML36 | | | | Prupe.7G047300 | | 690 | | 229 | 0 | Pp07:8360632..8361519 | |
| PperCML38-1 | | | | Prupe.5G152700 | | 423 | | 140 | 0 | Pp05:13769041..13769469 | |
| PperCML38-2 | | | | Prupe.5G000600 | | 558 | | 185 | 0 | Pp05:202891..203448 | |
| *Ricinus communis* | | | | | | | | | | | |
| RcCML3 | | | | 29927.t000001 | | 720 | | 239 | 0 | 29927:35621..36340 | |
| RcCML15 | | | | 30174.t000558 | | 492 | | 163 | 0 | 30174:2649853..2650344 | |
| RcCML16 | | | | 30138.t000007 | | 477 | | 158 | 0 | 30138:63953..64429 | |
| RcCML18 | | | | 30073.t000080 | | 540 | | 179 | 0 | 30073:569393..569932 | |
| RcCML23 | | | | 29900.t000034 | | 2112 | | 703 | 8 | 29900:227407..230555 | |
| RcCML25 | | | | 30174.t000299 | | 564 | | 187 | 0 | 30174:889340..889903 | |
| RcCML39 | | | | 30170.t000141 | | 573 | | 190 | 0 | 30170:4592799..4593371 | |
| RcCML41 | | | | 29250.t000006 | | 597 | | 198 | 0 | 29250:43521..44117 | |
| *Selaginella moellendorffii* | | | | | | | | | | | |
| SmCML3 | | | | 18113 | | 459 | | 153 | 0 | scaffold_38:681358..681816 | |
| SmCML4-1 | | | | 16319 | | 405 | | 135 | 1 | scaffold_63:626944..627477 | |
| SmCML5 | | | | 233632 | | 447 | | 148 | 0 | scaffold_47:140236..140682 | |
| SmCML7 | | | | 96597 | | 480 | | 159 | 0 | scaffold_18:1506156..1506635 | |
| SmCML19 | | | | 106606 | | 462 | | 153 | 5 | scaffold_33:1264038..1264749 | |
| SmCML20 | | | | 413855 | | 510 | | 169 | 6 | scaffold_21:2000016..2000945 | |
| SmCML21-1 | | | | 425580 | | 702 | | 233 | 3 | scaffold_75:428075..428926 | |
| SmCML21-2 | | | | 422194 | | 732 | | 243 | 3 | scaffold_55:515895..516823 | |
| SmCML25 | | | | 17991 | | 405 | | 135 | 0 | scaffold_11:1783215..1783619 | |
| SmCML26 | | | | 91501 | | 489 | | 162 | 0 | scaffold_12:2476213..2476701 | |
| SmCML27 | | | | 69135 | | 420 | | 140 | 0 | scaffold_2:3139681..3140100 | |
| *Setaria italica* | | | | | | | | | | | |
| SiCML2 | | | | Si011894m.g | | 459 | | 153 | 1 | scaffold_7:34719678..34720231 | |
| SiCML5 | | | | Si023155m.g | | 684 | | 227 | 2 | scaffold_3:48325368..48327322 | |
| SiCML10 | | | | Si024895m.g | | 765 | | 254 | 0 | scaffold_3:22798129..22798893 | |
| SiCML11 | | | | Si005109m.g | | 579 | | 192 | 0 | scaffold_5:46242983..46243561 | |
| SiCML12 | | | | Si002788m.g | | 693 | | 230 | 0 | scaffold_5:27773054..27773761 | |
| SiCML14 | | | | Si023383m.g | | 552 | | 183 | 0 | scaffold_3:9416288..9417081 | |
| SiCML16 | | | | Si002992m.g | | 591 | | 196 | 0 | scaffold_5:7896331..7897204 | |
| SiCML18 | | | | Si023408m.g | | 540 | | 179 | 0 | scaffold_3:5063311..5064272 | |
| SiCML19 | | | | Si003742m.g | | 429 | | 142 | 0 | scaffold_5:46562666..46563094 | |
| SiCML20 | | | | Si019543m.g | | 528 | | 175 | 0 | scaffold_1:37253371..37253898 | |
| SiCML22 | | | | Si010908m.g | | 756 | | 251 | 0 | scaffold_7:23704027..23705050 | |
| SiCML23 | | | | Si004282m.g | | 459 | | 152 | 0 | scaffold_5:46561042..46561500 | |
| SiCML28 | | | | Si024979m.g | | 480 | | 159 | 0 | scaffold_3:6138239..6138718 | |
| SiCML29 | | | | Si007236m.g | | 675 | | 224 | 4 | scaffold_4:33958455..33961568 | |
| SiCML30 | | | | Si007171m.g | | 735 | | 244 | 2 | scaffold_4:4242973..4244379 | |
| SiCML33 | | | | Si018277m.g | | 681 | | 226 | 3 | scaffold_1:4684943..4687441 | |
| SiCML36 | | | | Si037792m.g | | 510 | | 169 | 9 | scaffold_9:57000402..57003586 | |
| *Solanum lycopersicum* | | | | | | | | | | | |
| SlCML3-1 | | | | Solyc05g050750.1 | | 453 | | 150 | 0 | SL2.40ch05:60029606..60030058 | |
| SlCML3-2 | | | | Solyc09g055880.1 | | 453 | | 150 | 0 | SL2.40ch09:41606762..41607214 | |
| SlCML5 | | | | Solyc07g007950.1 | | 669 | | 222 | 0 | SL2.40ch07:2646086..2646754 | |
| SlCML7 | | | | Solyc06g083000.1 | | 450 | | 149 | 0 | SL2.40ch06:44867806..44868255 | |
| SlCML15 | | | | Solyc03g114420.1 | | 516 | | 171 | 0 | SL2.40ch03:58474677..58475192 | |
| SlCML16 | | | | Solyc01g010020.2 | | 486 | | 161 | 0 | SL2.40ch01:4657174..4658216 | |
| SlCML17 | | | | Solyc00g120930.1 | | 486 | | 161 | 1 | SL2.40ch00:16469782..16470453 | |
| SlCML18 | | | | Solyc03g097100.1 | | 492 | | 163 | 0 | SL2.40ch03:52891513..52892004 | |
| SlCML20 | | | | Solyc04g026350.2 | | 522 | | 173 | 6 | SL2.40ch04:26595840..26604419 | |
| SlCML21 | | | | Solyc10g005750.2 | | 693 | | 230 | 3 | SL2.40ch10:609439..611689 | |
| SlCML23 | | | | Solyc02g063350.1 | | 438 | | 145 | 0 | SL2.40ch02:29992547..29992984 | |
| SlCML24 | | | | Solyc02g091500.1 | | 471 | | 156 | 0 | SL2.40ch02:47358016..47358486 | |
| SlCML25-1 | | | | Solyc04g008000.2 | | 576 | | 191 | 1 | SL2.40ch04:1652161..1653061 | |
| SlCML25-2 | | | | Solyc01g005370.2 | | 519 | | 172 | 0 | SL2.40ch01:259638..260564 | |
| SlCML27-1 | | | | Solyc06g068960.1 | | 465 | | 154 | 0 | SL2.40ch06:39171990..39172454 | |
| SlCML27-2 | | | | Solyc03g118810.1 | | 495 | | 164 | 0 | SL2.40ch03:61676281..61676775 | |
| SlCML30-1 | | | | Solyc02g065000.1 | | 522 | | 173 | 0 | SL2.40ch02:30706851..30707372 | |
| SlCML30-2 | | | | Solyc01g108190.2 | | 558 | | 185 | 0 | SL2.40ch01:87315276..87316549 | |
| SlCML35 | | | | Solyc04g018110.1 | | 729 | | 242 | 0 | SL2.40ch04:9603164..9603892 | |
| SlCML36 | | | | Solyc10g079420.1 | | 618 | | 205 | 0 | SL2.40ch10:60282482..60283099 | |
| SlCML37-1 | | | | Solyc02g094000.1 | | 426 | | 141 | 0 | SL2.40ch02:49243310..49243735 | |
| SlCML37-2 | | | | Solyc03g005040.1 | | 426 | | 141 | 0 | SL2.40ch03:42015..42440 | |
| SlCML38-1 | | | | Solyc11g071740.1 | | 606 | | 201 | 0 | SL2.40ch11:52221262..52221867 | |
| SlCML38-2 | | | | Solyc06g073830.1 | | 555 | | 184 | 0 | SL2.40ch06:41978680..41979234 | |
| SlCML39-1 | | | | Solyc11g071750.1 | | 351 | | 116 | 0 | SL2.40ch11:52223163..52223513 | |
| SlCML39-2 | | | | Solyc11g071760.1 | | 597 | | 198 | 0 | SL2.40ch11:52225377..52225973 | |
| SlCML41 | | | | Solyc03g044900.2 | | 582 | | 193 | 1 | SL2.40ch03:14746177..14747185 | |
| *Solanum tuberosum* | | | | | | | | | | | |
| StCML3-1 | | | | PGSC0003DMG400035905 | | 534 | | 177 | 0 | chr12:2752501..2753034 | |
| StCML3-2 | | | | PGSC0003DMG400006998 | | 453 | | 150 | 0 | chr05:54497807..54498934 | |
| StCML3-3 | | | | PGSC0003DMG400026914 | | 660 | | 219 | 0 | chr06:47490420..47491377 | |
| StCML5 | | | | PGSC0003DMG400030892 | | 705 | | 234 | 0 | chr07:4121617..4123207 | |
| StCML7 | | | | PGSC0003DMG402027685 | | 450 | | 149 | 0 | chr06:54937634..54938275 | |
| StCML15 | | | | PGSC0003DMG400024604 | | 486 | | 161 | 0 | chr03:40200290..40201001 | |
| StCML18-1 | | | | PGSC0003DMG400003146 | | 492 | | 163 | 0 | chr03:22982025..22984113 | |
| StCML18-2 | | | | PGSC0003DMG400014797 | | 492 | | 163 | 0 | chr08:35893722..35895820 | |
| StCML20 | | | | PGSC0003DMG402004023 | | 507 | | 168 | 6 | chr02:65148009..65151274 | |
| StCML21 | | | | PGSC0003DMG400008692 | | 693 | | 230 | 3 | chr10:613197..617523 | |
| StCML22 | | | | PGSC0003DMG400031286 | | 714 | | 237 | 3 | chr09:50533331..50534540 | |
| StCML23-1 | | | | PGSC0003DMG400013427 | | 423 | | 140 | 0 | chr03:428262..428941 | |
| StCML23-2 | | | | PGSC0003DMG400030608 | | 471 | | 156 | 0 | chr02:63154779..63156028 | |
| StCML23-3 | | | | PGSC0003DMG400010410 | | 438 | | 145 | 0 | chr02:44338956..44339881 | |
| StCML25-1 | | | | PGSC0003DMG402029470 | | 585 | | 194 | 1 | chr04:2600053..2601193 | |
| StCML25-2 | | | | PGSC0003DMG400012770 | | 579 | | 192 | 0 | chr01:76984188..76985491 | |
| StCML30-1 | | | | PGSC0003DMG402025892 | | 558 | | 185 | 0 | chr01:90660990..90661547 | |
| StCML30-2 | | | | PGSC0003DMG400039532 | | 522 | | 173 | 0 | chr02:45391739..45392260 | |
| StCML36-1 | | | | PGSC0003DMG400010631 | | 651 | | 216 | 0 | chr04:38052353..38053345 | |
| StCML36-2 | | | | PGSC0003DMG400008163 | | 618 | | 205 | 0 | chr10:44530069..44531020 | |
| StCML36-3 | | | | PGSC0003DMG400029833 | | 636 | | 211 | 0 | chr10:44877807..44878919 | |
| StCML37 | | | | PGSC0003DMG400045603 | | 552 | | 183 | 0 | chr11:40408684..40409235 | |
| StCML38-1 | | | | PGSC0003DMG400005909 | | 576 | | 191 | 0 | chr06:49508947..49509951 | |
| StCML38-2 | | | | PGSC0003DMG400040538 | | 486 | | 161 | 0 | chr11:40405106..40405591 | |
| StCML38-3 | | | | PGSC0003DMG400002993 | | 588 | | 195 | 0 | chr11:40414040..40414886 | |
| StCML38-4 | | | | PGSC0003DMG400020261 | | 426 | | 141 | 0 | chr02:69135730..69136633 | |
| StCML41 | | | | PGSC0003DMG401022924 | | 585 | | 194 | 1 | chr03:25522674..25523377 | |
| *Sorghum bicolor* | | | | | | | | | | | |
| SbCML1 | | | | Sobic.002G226300 | | 555 | | 184 | 0 | Chr02:61776875..61777948 | |
| SbCML5 | | | | Sobic.008G159100 | | 531 | | 176 | 0 | Chr08:51837600..51838779 | |
| SbCML6 | | | | Sobic.004G095400 | | 1140 | | 379 | 7 | Chr04:8320056..8325469 | |
| SbCML8 | | | | Sobic.002G376500 | | 519 | | 172 | 5 | Chr02:73341044..73343871 | |
| SbCML10-1 | | | | Sobic.003G425500 | | 570 | | 189 | 0 | Chr03:73005444..73006013 | |
| SbCML10-2 | | | | Sobic.009G120800 | | 600 | | 199 | 0 | Chr09:47159076..47160774 | |
| SbCML11 | | | | Sobic.004G024000 | | 759 | | 252 | 0 | Chr04:1968141..1969211 | |
| SbCML12 | | | | Sobic.003G212000 | | 711 | | 236 | 0 | Chr03:54678909..54679619 | |
| SbCML14 | | | | Sobic.009G242700 | | 561 | | 186 | 0 | Chr09:57924084..57925104 | |
| SbCML15 | | | | Sobic.006G270800 | | 1089 | | 362 | 5 | Chr06:61218699..61220906 | |
| SbCML16 | | | | Sobic.003G082600 | | 621 | | 206 | 1 | Chr03:7084351..7087691 | |
| SbCML18 | | | | Sobic.009G083600 | | 540 | | 179 | 0 | Chr09:12793788..12795037 | |
| SbCML19 | | | | Sobic.003G430600 | | 465 | | 154 | 0 | Chr03:73342009..73342730 | |
| SbCML20 | | | | Sobic.004G247900 | | 543 | | 180 | 0 | Chr04:58844433..58844975 | |
| SbCML22 | | | | Sobic.006G131600 | | 717 | | 238 | 0 | Chr06:50500756..50502394 | |
| SbCML28 | | | | Sobic.008G079200 | | 498 | | 165 | 0 | Chr08:12521944..12523256 | |
| SbCML29 | | | | Sobic.010G188800 | | 675 | | 224 | 4 | Chr10:52654747..52658262 | |
| SbCML30 | | | | Sobic.010G057000 | | 738 | | 245 | 0 | Chr10:4454188..4456436 | |
| SbCML31 | | | | Sobic.003G430400 | | 507 | | 168 | 0 | Chr03:73337673..73338179 | |
| SbCML33 | | | | Sobic.004G080800 | | 687 | | 228 | 4 | Chr04:6655302..6659511 | |
| SbCML34 | | | | Sobic.006G010800 | | 693 | | 230 | 3 | Chr06:1533006..1537355 | |
| SbCML50 | | | | Sobic.001G517100 | | 510 | | 169 | 9 | Chr01:71155794..71160067 | |
| *Thelluginella halophila (Eutrema salsugineum)* | | | | | | | | | | | |
| ThCML2 | | | | Thhalv10027394m.g | | 459 | | 152 | 0 | scaffold_1:20034269..20034727 | |
| ThCML3 | | | | Thhalv10021694m.g | | 462 | | 153 | 0 | scaffold_13:7004492..7004984 | |
| ThCML4 | | | | Thhalv10006367m.g | | 606 | | 201 | 0 | scaffold_19:1415640..1416245 | |
| ThCML5-1 | | | | Thhalv10001753m.g | | 804 | | 267 | 1 | scaffold_22:44360..46624 | |
| ThCML5-2 | | | | Thhalv10029037m.g | | 465 | | 154 | 0 | scaffold_3:1659514..1660302 | |
| ThCML11 | | | | Thhalv10021692m.g | | 462 | | 153 | 2 | scaffold_13:820045..822186 | |
| ThCML15 | | | | Thhalv10008984m.g | | 474 | | 157 | 0 | scaffold_5:9132877..9133367 | |
| ThCML16 | | | | Thhalv10005049m.g | | 489 | | 162 | 0 | scaffold_6:6737640..6738308 | |
| ThCML17 | | | | Thhalv10009363m.g | | 501 | | 166 | 0 | scaffold_5:2778319..2778819 | |
| ThCML18 | | | | Thhalv10021666m.g | | 504 | | 167 | 0 | scaffold_13:8800440..8801302 | |
| ThCML19 | | | | Thhalv10026403m.g | | 513 | | 170 | 5 | scaffold_1:1249730..1251030 | |
| ThCML20 | | | | Thhalv10008929m.g | | 510 | | 169 | 5 | scaffold_5:904135..905783 | |
| ThCML21 | | | | Thhalv10026168m.g | | 681 | | 226 | 4 | scaffold_1:5776670..5778104 | |
| ThCML22 | | | | Thhalv10002654m.g | | 681 | | 226 | 3 | scaffold_4:7480936..7481918 | |
| ThCML23-1 | | | | Thhalv10019832m.g | | 507 | | 168 | 0 | scaffold_9:1737046..1737552 | |
| ThCML23-2 | | | | Thhalv10019556m.g | | 774 | | 258 | 2 | scaffold_9:8823862..8826275 | |
| ThCML24 | | | | Thhalv10027972m.g | | 480 | | 159 | 0 | scaffold_14:6508167..6508983 | |
| ThCML25 | | | | Thhalv10008816m.g | | 597 | | 198 | 0 | scaffold_5:6022730..6023563 | |
| ThCML26 | | | | Thhalv10019258m.g | | 483 | | 160 | 0 | scaffold_9:3100055..3101077 | |
| ThCML27 | | | | Thhalv10008945m.g | | 501 | | 166 | 1 | scaffold_5:9262473..9263747 | |
| ThCML30 | | | | Thhalv10022869m.g | | 570 | | 189 | 0 | scaffold_11:5720319..5720985 | |
| ThCML32 | | | | Thhalv10015440m.g | | 435 | | 144 | 0 | scaffold_2:1018462..1018896 | |
| ThCML33 | | | | Thhalv10022417m.g | | 462 | | 153 | 0 | scaffold_13:8668506..8668967 | |
| ThCML34 | | | | Thhalv10022182m.g | | 396 | | 131 | 0 | scaffold_13:8658723..8659118 | |
| ThCML36 | | | | Thhalv10021512m.g | | 636 | | 211 | 0 | scaffold_13:6258662..6259615 | |
| ThCML38 | | | | Thhalv10019204m.g | | 537 | | 178 | 0 | scaffold_9:1734774..1735500 | |
| ThCML39 | | | | Thhalv10003430m.g | | 555 | | 184 | 0 | scaffold_17:5169510..5170064 | |
| *Theobroma cacao* | | | | | | | | | | | |
| TcCML5-1 | | | | Thecc1EG038140 | | 702 | | 233 | 1 | scaffold_9:7238830..7240674 | |
| TcCML5-2 | | | | Thecc1EG020039 | | 663 | | 220 | 0 | scaffold_4:26291299..26292543 | |
| TcCML15 | | | | Thecc1EG014474 | | 486 | | 161 | 0 | scaffold_3:22621157..22621938 | |
| TcCML18 | | | | Thecc1EG016579 | | 492 | | 163 | 1 | scaffold_3:33486411..33487370 | |
| TcCML21 | | | | Thecc1EG030523 | | 693 | | 230 | 4 | scaffold_7:122970..125479 | |
| TcCML22 | | | | Thecc1EG020331 | | 735 | | 244 | 5 | scaffold_4:27959257..27964633 | |
| TcCML23 | | | | Thecc1EG019204 | | 459 | | 152 | 1 | scaffold_4:20966233..20971155 | |
| TcCML25-1 | | | | Thecc1EG005493 | | 591 | | 196 | 0 | scaffold_1:36922990..36924420 | |
| TcCML25-2 | | | | Thecc1EG035023 | | 474 | | 157 | 0 | scaffold_8:6054339..6055227 | |
| TcCML25-3 | | | | Thecc1EG011044 | | 600 | | 199 | 2 | scaffold_2:37573844..37585702 | |
| TcCML27 | | | | Thecc1EG012743 | | 510 | | 169 | 0 | scaffold_3:5654309..5655459 | |
| TcCML30 | | | | Thecc1EG000601 | | 567 | | 188 | 0 | scaffold_1:2544437..2545479 | |
| TcCML37 | | | | Thecc1EG034792 | | 915 | | 304 | 1 | scaffold_8:4826061..4827605 | |
| TcCML38 | | | | Thecc1EG014613 | | 423 | | 140 | 0 | scaffold_3:23494069..23494838 | |
| *Vitis vinifera* | | | | | | | | | | | |
| VvCML16-1 | | | | GSVIVG01008204001 | | 483 | | 160 | 0 | chr17:4709595..4710077 | |
| VvCML16-2 | | | | GSVIVG01012006001 | | 489 | | 162 | 0 | chr1:2251732..2252693 | |
| VvCML20-1 | | | | GSVIVG01022137001 | | 516 | | 171 | 7 | chr7:16861134..16865806 | |
| VvCML20-2 | | | | GSVIVG01038234001 | | 498 | | 165 | 5 | chr5:24529157..24533756 | |
| VvCML21 | | | | GSVIVG01014761001 | | 699 | | 232 | 3 | chr19:9373956..9376573 | |
| VvCML30 | | | | GSVIVG01031752001 | | 876 | | 291 | 2 | chr3:4054284..4059659 | |
| VvCML36 | | | | GSVIVG01033567001 | | 528 | | 175 | 1 | chr8:19704974..19705910 | |
| VvCML38 | | | | GSVIVG01010383001 | | 447 | | 148 | 1 | chr1:20027402..20029452 | |
| VvCML39-1 | | | | GSVIVG01008516001 | | 432 | | 143 | 0 | chr17:1200420..1200851 | |
| VvCML39-2 | | | | GSVIVG01013467001 | | 618 | | 205 | 1 | chr18:227240..228213 | |
| VvCML41-1 | | | | GSVIVG01010378001 | | 465 | | 154 | 1 | chr1:19997292..20000950 | |
| VvCML41-2 | | | | GSVIVG01010382001 | | 474 | | 157 | 1 | chr1:20019343..20022287 | |
| VvCML41-3 | | | | GSVIVG01010385001 | | 471 | | 156 | 1 | chr1:20036450..20040327 | |
| *Volvox carteri* | | | | | | | | | | | |
| VcCML4 | | | | Vocar20008810m.g | | 687 | | 228 | 5 | scaffold_1:12719166..12722029 | |
| VcCML5 | | | | Vocar20012315m.g | | 891 | | 296 | 8 | scaffold_32:582482..588177 | |
| VcCML20-1 | | | | Vocar20011868m.g | | 507 | | 168 | 6 | scaffold_50:197326..199345 | |
| VcCML20-2 | | | | Vocar20006166m.g | | 513 | | 170 | 8 | scaffold_12:1933884..1935845 | |
| *Zea mays* | | | | | | | | | | | |
| ZmCML2 | | | | GRMZM2G115628 | | 483 | | 160 | 2 | 2:149302366..149303449 | |
| ZmCML4 | | | | GRMZM2G149923 | | 465 | | 154 | 3 | 1:275154534..275157007 | |
| ZmCML5 | | | | GRMZM2G444621 | | 510 | | 169 | 0 | 3:118981074..118981990 | |
| ZmCML8-1 | | | | GRMZM2G096228 | | 540 | | 179 | 5 | 5:37396826..37398843 | |
| ZmCML8-2 | | | | GRMZM2G048846 | | 519 | | 172 | 6 | 7:165886707..165889308 | |
| ZmCML10-1 | | | | GRMZM2G106945 | | 585 | | 194 | 0 | 3:149591737..149593176 | |
| ZmCML10-2 | | | | GRMZM2G474755 | | 570 | | 189 | 0 | 8:91126489..91127653 | |
| ZmCML10-3 | | | | GRMZM2G340807 | | 615 | | 204 | 0 | 6:142752579..142753820 | |
| ZmCML11 | | | | GRMZM2G152432 | | 636 | | 211 | 0 | 5:43954103..43955065 | |
| ZmCML14-1 | | | | GRMZM2G072052 | | 705 | | 234 | 0 | 3:223776106..223777096 | |
| ZmCML14-2 | | | | GRMZM5G847466 | | 519 | | 172 | 0 | 6:166188864..166189789 | |
| ZmCLM16 | | | | GRMZM2G097900 | | 618 | | 205 | 0 | 8:12992175..12993622 | |
| ZmCML17 | | | | GRMZM2G062673 | | 669 | | 222 | 0 | 2:34358963..34360177 | |
| ZmCML19 | | | | GRMZM2G340313 | | 456 | | 151 | 0 | 8:157227643..157228098 | |
| ZmCML20-1 | | | | GRMZM2G362857 | | 534 | | 177 | 0 | 4:167002557..167003289 | |
| ZmCML20-2 | | | | GRMZM2G375782 | | 540 | | 179 | 0 | 5:205125425..205126244 | |
| ZmCML28 | | | | GRMZM2G133588 | | 480 | | 159 | 0 | 10:34007307..34007944 | |
| ZmCML30 | | | | GRMZM2G071100 | | 732 | | 243 | 0 | 6:122846832..122848227 | |
| ZmCML36-1 | | | | GRMZM2G081310 | | 1689 | | 562 | 6 | 4:157020786..157026774 | |
| ZmCML36-2 | | | | GRMZM2G040743 | | 1623 | | 540 | 6 | 1:84303766..84308704 | |
| ZmCML36-3 | | | | GRMZM2G003059 | | 618 | | 205 | 3 | 10:34934947..34936354 | |
