# Supplementary material for: Genomics and evolutionary aspect of calcium signaling event in calmodulin and calmodulin-like proteins in plants
Source: BMC Plant Biol. 2017 Feb 3;17:38. doi: 10.1186/s12870-017-0989-3 (PMC5291997; doi:10.1186/s12870-017-0989-3)
Supplement: Additional file 3: Figure S1. — Multiple sequence alignment of CaM protein of plant. Alignment shows presence of conserved motifs in EF-hand domain. (PDF 817 kb) [file 12870_2017_989_MOESM3_ESM.pdf]

```
PvCaM1-2     MLSVPSFPRG ERLARPARAG ALAASVAWVR SVSATVSRLL FSSLVGWAPL SHFLHS---- ---RFASLSL PVSTPSVRSL APAAMADQLT
SiCaM1-2      MEDAQLPNI APQLEHERQV SRGTHEAGRS VRLGLLSSRL PRGRGILSPL PPFALR---- ---LSAPRLP PSLPPSVRSL ARVAMADQLT
AtCaM3       MADQLT
AtCaM5       MADQLT
ThCaM3-2     MADQLT
BrCaM5-5     MADQLT
BrCaM5-4     MADQLT
BrCaM5-3     MADQLT
BrCaM5-2     MADQLT
CrCaM5       MADQLT
ThCaM5-1     MADQLT
BdCaM1-2     MADQLT
BdCaM1-1     MADQLT
ZmCaM1-1     MADQLT
SbCaM1-4     MADQLT
SbCaM1-2     MADQLT
SbCaM1-3     MADQLT
SbCaM1-1     MADQLT
SiCaM1-1     MADQLT
OsCaM1-2     MADQLT
OsCaM1-3     MADQLT
OsCaM1-1     MADQLT
PhCaM1-3     MADQLT
PhCaM1-4     MADQLT
PhCaM1-5     MADQLT
PvCaM1-4     MADQLT
PvCaM1-5     MADQLT
PvCaM1-6     MADQLT
BdCaM3       MADQLT
PvCaM1-3     MADQLT
BrCaM5-6     MADQLT
BrCaM5-7     MADQLT
BrCaM5-8     MADQLT
MgCaM6       MADQLT
SlCaM2-2     MADQLT
ThCaM5-2     MADQLT
SlCaM2-1     MADQLT
SlCaM3       MADQLT
StCaM2-2     MADQLT
BrCaM5-10    MADQLT
SlCaM5-2     MADQLT
StCaM3       MADQLT
AtCaM6       MADQLT
BdCaM1-3     MADQLT
ZmCaM1-6     MADQLT
PvulCaM5     MADQLT
SiCaM1-3     MADQLT
ZmCaM1-7     MADQLT
PhCaM1-2     MADQLT
PvCaM1-7     MADQLT
PvCaM1-8     MADQLT
MtCaM7       MADQLT
PvulCaM7-3   MADQLT
PvulCaM7-2   MADQLT
GrCaM5-2     MADQLT
ZmCaM1-8     MADQLT
OsCaM2       MADQLT
OsCaM3       MADQLT
GmCaM5       MANQLT
AtCaM1       MADQLT
AtCaM4       MADQLT
ThCaM1       MADQLT
ThCaM4       MADQLT
CrCaM1-2     MADQLT
CrCaM1-1     MADQLT
BrCaM1-1     MADQLT
BrCaM1-2     MADQLT
MtCaM1       MADQLT
SmCaM5-1     MADQLT
PaCaM7       M EASRIVEQLT
AcCaM5-1     MADQLT
TcCaM7       MADQLT
CcCaM6-4     MADQLT
BrCaM5-1     MADQLT
ThCaM3-1     MADQLT
CrCaM3       M GLSPPFSSAL YNSRTTPPPS SLNHTN---- ---VDSSSSS SSSL-----F LILKMADQLT
PtCaM7       MADQLT
BrCaM5-9     MADQLT
ZmCaM1-4     MADQLT
PpCaM5-1     MVEQLS
ZmCaM1-2     MADQLT
PhCaM3       MADQLS
PvCaM1-10    MADQLS
SbCaM1-6     MADQLS
VvCaM7       MADQLT
ZmCaM1-3     MADQLT
PaCaM5-2     MMDKLT
AcCaM6       MAEQLT
VvCaM5-1     MAEQLT
StCaM2-1     MAEQLT
PtCaM2       MTEQLT
PtCaM3       MSEQLT
MgCaM7       MAEQLT
PaCaM5-1     MAEQLT
PpCaM5-4     MAEHLT
PpCaM5-2     MVEQLS
PpCaM7       MVEQLT
AtCaM2       MADQLT
SmCaM5-2     MAAAAEQLT
SmCaM5-3     MVEELT
CsubCaM5-1   MTEAAQLT
MpCaM5-2     MADTLT
OlCaM5       MAADLT
FvCaM7-1     MTEQLT
PperCaM7-2   MAEQLT
MdCaM2       MAEQLT
MdCaM3       MAEQLT
PaCaM3       NRKQIEL SRSFSHLGTV QSNTMAVKLT
GrCaM3       MVDQLT
CreinCaM6-   MAANTEQLT
VcCaM6-2     MAQTTEQLT
PaCaM1-1     MANPLT
BrCaM5-11    MADQLT
AtCaM8       MEETALT
CrCaM8       MEETALT
ThCaM8       MEETALT
MgCaM5-5     MEELVLN
MgCaM8-2     MEELVLN
CcCaM8       MSEVLT
CsCaM8       MSEVLT
PtCaM8-2     MAEALT
PtCaM8-1     MAEVLT
CsatCaM8-3   MTEVLS
GrCaM8-1     MGDILS
PperCaM8     MADVLS
VvCaM8-1     MADVLS
GmCaM8-1     MADVLS
GmCaM8-2     MADVLS
PvulCaM8-1   MADVLS
MtCaM8       MAHVLS
SlCaM1       MGDVLN
MgCaM8-3     MADALN
GrCaM8-2     MRDIIS
FvCaM8-2     MAGVET LFGGVESGFF WWSRDSGPNA DTSGGGHVER GAEVYVPDVG GGVESLTEAC SLRSPV-GPF SSSSKGQNML QLPYKRVENT KYYLNKIKDE EEDTRGIFI QTFTMAEVLS
SbCaM1-7     MAGKGGKGLL AAKTTAAKSA EKDKGKKAPV SRSSRAGLQL KQRTQANGRV GATAAVYSAA ILEYLTAEVL ELAGNASKDL KVKRITPRHL QLAIRGDEEL DTLIKGTIAG GGPEHTAILP PVCCVVQGGL VFRAAGCGVI KEPCYTI--- ---LRLLKSN SNTKEQLFLS GIGSCMDGLT
ThCaM5-3     MADELF
FvCaM8-1     MAEALT
PvCaM1-9     MADQLT
SiCaM3       MDEVEQQLS
SbCaM3       MEEVEQTLT
ZmCaM3       MEEVEQQLT
SbCaM1-5     MAEHFN
AcCaM5-3
AcCaM8       MADLLT
CpCaM8       MGDVLT
TcCaM9-2     MGEVLT
CcCaM5       MGDVLP
MeCaM8-2     MGDVLT
MeCaM8-1     MGDVLS
PtCaM8-3     MVDVLT
VvCaM5-2     MRPNNGDVLT
CcCaM4       MGDVLP
PvulCaM8-3   MKETLR
GmCaM8-3     MKEVLS
PvulCaM8-2   MKEVLS
SmCaM1-2     MEASETSTA SQDKIIEGLT
SlCaM5-1     MADKIE
StCaM8       MADKIV
SmCaM1-1     MAELAAdditional
```

```
CcCaM6-2     MADALT
CsCaM5-1     MADALT
CsatCaM8-1   MAAEALT
MdCaM4-1     MADALT
MdCaM4-2     MADALT
MdCaM8-1     MAEALT
MdCaM8-2     MAEALT
MeCaM9-2     MTDALT
RcCaM9-1     MADALT
MeCaM9-1     MADALT
VvCaM8-2     MANALT
GmCaM9-3     MAGGLT
GmCaM9-4     MAGALT
PvulCaM9-1   MEGALT
MgCaM9-1     MTDSLS
SlCaM4       MADALT
MgCaM4       NLYV
MgCaM8-1
LuCaM6-1     MEV LTAAGNGTEV
LuCaM6-2     MEV LQG-----EI
CsatCaM9     M
CsatCaM8-2   MGEALT
CreinCaM1    M PGEKYNVEVD
MpCaM1       MVL TEKVAGAG--
MpCaM5-4     MQARKIA LRGRVRHDSH PFVAEPPHSR GAPAGSARGR STFTALRDRR VSSHPAQHLA RGAEEIER-- ---RTRGGRM SREGKKKYLT DEELSAEWPY
OlCaM1       MSRKHLT DEELASAWPY
GmCaM9-1     MANAWT
GmCaM9-2     MANAWT
PvulCaM9-2   MANAWT
MtCaM9       MSHKLT
AtCaM9       MADAFT
ThCaM9       MADGFT
CrCaM9       MADAFT
CreinCaM8    MSYKA KTVVSARRDQ KK-GRV-GLT
CsubCaM4     MSSYR KAVTAARRDK KN-GKHQGLT
PpCaM8-2     MGTARRDK NK-GRSQGLT
PpCaM8-1     MSFRK PGMGPARRDK AKPGRFQGLT
SlCaM8       M ASVSRKMSRK ETRNRHPGLT
LuCaM9-2     MNTAAGAILE
LuCaM9-4     MNTAAGAILE
LuCaM9-3     MNNA-GAILE
LuCaM9-1     MAAGAVEAAA
CsCaM4
MgCaM9-2
CreinCaM6-
VcCaM6-1
EuCaM6       MGKDLT
PaCaM6-1     MGKDLS
GrCaM9       MAHTLT
SiCaM1-4
TcCaM9-1     MPDTLT
ThCaM7
CrCaM6       MASAN PETAKLTPAS
StCaM6-1     MAATDN VNNNSVSKPS
RcCaM9-2     MDNANK SKKNEGKRLS STFELLTPSF
FvCaM3       MQIIA IGSKAYKWFF TTKNLML--- ---KLNLSFL GSKPKNHSPS LSASLPTESS
MpCaM5-3     M GLFGKSKSKR AQELKKLYTG YKVDPKVAEL
PaCaM5-3     M TNKFKKR--A LRV---IADH
PaCaM1-2     MKQFSA INKLKKKKVA LKV---IVES
CcCaM3       M GTSHPCPPLK YLSNKVRGML CHCGSQNKYA RLDAKLE--- ---KKMIEVK KSSSGHSNFK SIDSVIVRFP
PpCaM5-3     MCYEGV PGSNDHRRSL ERSEGARGAV
VcCaM3       MPNKSK DGPYSTNLPP
CpCaM6
PaCaM6-2
CreinCaM3
CreinCaM5
VcCaM5
CsubCaM5-2
MpCaM5-1
Consensus    ... ....madqlt

             181                                                                                                                                                                                 360
AcCaM5-2     --DDQISEFK EAFSLFDKDG DG-------- ----CITTKE LGTVMRSLG- QN-PTEAELQ DMINEV---D AD--GNG-TI DFPEFLNLM- ---ARKMKDT D--------- --SEEE-LKE AFRVFDKDQN -GFISAAE-- ---------- ----LRHV-- MTNLGE-KLT DEEVDEMIRE
AtCaM7       --DDQISEFK EAFSLFDKDG DG-------- ----CITTKE LGTVMRSLG- QN-PTEAELQ DMINEV---D AD--GNG-TI DFPEFLNLM- ---ARKMKDT D--------- --SEEE-LKE AFRVFDKDQN -GFISAAE-- ---------- ----LRHV-- MTNLGE-KLT DEEVDEMIRE
CrCaM7-2     --DDQISEFK EAFSLFDKDG DG-------- ----CITTKE LGTVMRSLG- QN-PTEAELQ DMINEV---D AD--GNG-TI DFPEFLNLM- ---ARKMKDT D--------- --SEEE-LKE AFRVFDKDQN -GFISAAE-- ---------- ----LRHV-- MTNLGE-KLT DEEVDEMIRE
CpCaM7       --DDQISEFK EAFSLFDKDG DG-------- ----CITTKE LGTVMRSLG- QN-PTEAELQ DMINEV---D AD--GNG-TI DFPEFLNLM- ---ARKMKDT D--------- --SEEE-LKE AFRVFDKDQN -GFISAAE-- ---------- ----LRHV-- MTNLGE-KLT DEEVDEMIRE
CcCaM6-3     --DDQISEFK EAFSLFDKDG DG-------- ----CITTKE LGTVMRSLG- QN-PTEAELQ DMINEV---D AD--GNG-TI DFPEFLNLM- ---ARKMKDT D--------- --SEEE-LKE AFRVFDKDQN -GFISAAE-- ---------- ----LRHV-- MTNLGE-KLT DEEVDEMIRE
CcCaM6-1     --DDQISEFK EAFSLFDKDG DG-------- ----CITTKE LGTVMRSLG- QN-PTEAELQ DMINEV---D AD--GNG-TI DFPEFLNLM- ---ARKMKDT D--------- --SEEE-LKE AFRVFDKDQN -GFISAAE-- ---------- ----LRHV-- MTNLGE-KLT DEEVDEMIRE
CsCaM7-2     --DDQISEFK EAFSLFDKDG DG-------- ----CITTKE LGTVMRSLG- QN-PTEAELQ DMINEV---D AD--GNG-TI DFPEFLNLM- ---ARKMKDT D--------- --SEEE-LKE AFRVFDKDQN -GFISAAE-- ---------- ----LRHV-- MTNLGE-KLT DEEVDEMIRE
FvCaM7-2     --DDQISEFK EAFSLFDKDG DG-------- ----CITTKE LGTVMRSLG- QN-PTEAELQ DMINEV---D AD--GNG-TI DFPEFLNLM- ---ARKMKDT D--------- --SEEE-LKE AFRVFDKDQN -GFISAAE-- ---------- ----LRHV-- MTNLGE-KLT DEEVDEMIRE
GrCaM5-1     --DDQISEFK EAFSLFDKDG DG-------- ----CITTKE LGTVMRSLG- QN-PTEAELQ DMINEV---D AD--GNG-TI DFPEFLNLM- ---ARKMKDT D--------- --SEEE-LKE AFRVFDKDQN -GFISAAE-- ---------- ----LRHV-- MTNLGE-KLT DEEVDEMIRE
LuCaM6-3     --DDQISEFK EAFSLFDKDG DG-------- ----CITTKE LGTVMRSLG- QN-PTEAELQ DMINEV---D AD--GNG-TI DFPEFLNLM- ---ARKMKDT D--------- --SEEE-LKE AFRVFDKDQN -GFISAAE-- ---------- ----LRHV-- MTNLGE-KLT DEEVDEMIRE
LuCaM7-2     --DDQISEFK EAFSLFDKDG DG-------- ----CITTKE LGTVMRSLG- QN-PTEAELQ DMINEV---D AD--GNG-TI DFPEFLNLM- ---ARKMKDT D--------- --SEEE-LKE AFRVFDKDQN -GFISAAE-- ---------- ----LRHV-- MTNLGE-KLT DEEVDEMIRE
LuCaM7-3     --DDQISEFK EAFSLFDKDG DG-------- ----CITTKE LGTVMRSLG- QN-PTEAELQ DMINEV---D AD--GNG-TI DFPEFLNLM- ---ARKMKDT D--------- --SEEE-LKE AFRVFDKDQN -GFISAAE-- ---------- ----LRHV-- MTNLGE-KLT DEEVDEMIRE
LuCaM7-1     --DDQISEFK EAFSLFDKDG DG-------- ----CITTKE LGTVMRSLG- QN-PTEAELQ DMINEV---D AD--GNG-TI DFPEFLNLM- ---ARKMKDT D--------- --SEEE-LKE AFRVFDKDQN -GFISAAE-- ---------- ----LRHV-- MTNLGE-KLT DEEVDEMIRE
MdCaM6-1     --DDQISEFK EAFSLFDKDG DG-------- ----CITTKE LGTVMRSLG- QN-PTEAELQ DMINEV---D AD--GNG-TI DFPEFLNLM- ---ARKMKDT D--------- --SEEE-LKE AFRVFDKDQN -GFISAAE-- ---------- ----LRHV-- MTNLGE-KLT DEEVDEMIRE
MdCaM7       --DDQISEFK EAFSLFDKDG DG-------- ----CITTKE LGTVMRSLG- QN-PTEAELQ DMINEV---D AD--GNG-TI DFPEFLNLM- ---ARKMKDT D--------- --SEEE-LKE AFRVFDKDQN -GFISAAE-- ---------- ----LRHV-- MTNLGE-KLT DEEVDEMIRE
MeCaM5-1     --DDQISEFK EAFSLFDKDG DG-------- ----CITTKE LGTVMRSLG- QN-PTEAELQ DMINEV---D AD--GNG-TI DFPEFLNLM- ---ARKMKDT D--------- --SEEE-LKE AFRVFDKDQN -GFISAAE-- ---------- ----LRHV-- MTNLGE-KLT DEEVDEMIRE
MeCaM5-4     --DDQISEFK EAFSLFDKDG DG-------- ----CITTKE LGTVMRSLG- QN-PTEAELQ DMINEV---D AD--GNG-TI DFPEFLNLM- ---ARKMKDT D--------- --SEEE-LKE AFRVFDKDQN -GFISAAE-- ---------- ----LRHV-- MTNLGE-KLT DEEVDEMIRE
MeCaM5-3     --DDQISEFK EAFSLFDKDG DG-------- ----CITTKE LGTVMRSLG- QN-PTEAELQ DMINEV---D AD--GNG-TI DFPEFLNLM- ---ARKMKDT D--------- --SEEE-LKE AFRVFDKDQN -GFISAAE-- ---------- ----LRHV-- MTNLGE-KLT DEEVDEMIRE
MeCaM7       --DDQISEFK EAFSLFDKDG DG-------- ----CITTKE LGTVMRSLG- QN-PTEAELQ DMINEV---D AD--GNG-TI DFPEFLNLM- ---ARKMKDT D--------- --SEEE-LKE AFRVFDKDQN -GFISAAE-- ---------- ----LRHV-- MTNLGE-KLT DEEVDEMIRE
RcCaM7-1     --DDQISEFK EAFSLFDKDG DG-------- ----CITTKE LGTVMRSLG- QN-PTEAELQ DMINEV---D AD--GNG-TI DFPEFLNLM- ---ARKMKDT D--------- --SEEE-LKE AFRVFDKDQN -GFISAAE-- ---------- ----LRHV-- MTNLGE-KLT DEEVDEMIRE
MeCaM5-2     --DDQISEFK EAFSLFDKDG DG-------- ----CITTKE LGTVMRSLG- QN-PTEAELQ DMINEV---D AD--GNG-TI DFPEFLNLM- ---ARKMKDT D--------- --SEEE-LKE AFRVFDKDQN -GFISAAE-- ---------- ----LRHV-- MTNLGE-KLT DEEVDEMIRE
RcCaM7-2     --DDQISEFK EAFSLFDKDG DG-------- ----CITTKE LGTVMRSLG- QN-PTEAELQ DMINEV---D AD--GNG-TI DFPEFLNLM- ---ARKMKDT D--------- --SEEE-LKE AFRVFDKDQN -GFISAAE-- ---------- ----LRHV-- MTNLGE-KLT DEEVDEMIRE
MgCaM5-1     --DDQISEFK EAFSLFDKDG DG-------- ----CITTKE LGTVMRSLG- QN-PTEAELQ DMINEV---D AD--GNG-TI DFPEFLNLM- ---ARKMKDT D--------- --SEEE-LKE AFRVFDKDQN -GFISAAE-- ---------- ----LRHV-- MTNLGE-KLT DEEVDEMIRE
PperCaM5     --DDQISEFK EAFSLFDKDG DG-------- ----CITTKE LGTVMRSLG- QN-PTEAELQ DMINEV---D AD--GNG-TI DFPEFLNLM- ---ARKMKDT D--------- --SEEE-LKE AFRVFDKDQN -GFISAAE-- ---------- ----LRHV-- MTNLGE-KLT DEEVDEMIRE
MgCaM5-2     --DDQISEFK EAFSLFDKDG DG-------- ----CITTKE LGTVMRSLG- QN-PTEAELQ DMINEV---D AD--GNG-TI DFPEFLNLM- ---ARKMKDT D--------- --SEEE-LKE AFRVFDKDQN -GFISAAE-- ---------- ----LRHV-- MTNLGE-KLT DEEVDEMIRE
MgCaM5-3     --DDQISEFK EAFSLFDKDG DG-------- ----CITTKE LGTVMRSLG- QN-PTEAELQ DMINEV---D AD--GNG-TI DFPEFLNLM- ---ARKMKDT D--------- --SEEE-LKE AFRVFDKDQN -GFISAAE-- ---------- ----LRHV-- MTNLGE-KLT DEEVDEMIRE
MgCaM5-4     --DDQISEFK EAFSLFDKDG DG-------- ----CITTKE LGTVMRSLG- QN-PTEAELQ DMINEV---D AD--GNG-TI DFPEFLNLM- ---ARKMKDT D--------- --SEEE-LKE AFRVFDKDQN -GFISAAE-- ---------- ----LRHV-- MTNLGE-KLT DEEVDEMIRE
PtCaM6-1     --DDQISEFK EAFSLFDKDG DG-------- ----CITTKE LGTVMRSLG- QN-PTEAELQ DMINEV---D AD--GNG-TI DFPEFLNLM- ---ARKMKDT D--------- --SEEE-LKE AFRVFDKDQN -GFISAAE-- ---------- ----LRHV-- MTNLGE-KLT DEEVDEMIRE
PtCaM6-2     --DDQISEFK EAFSLFDKDG DG-------- ----CITTKE LGTVMRSLG- QN-PTEAELQ DMINEV---D AD--GNG-TI DFPEFLNLM- ---ARKMKDT D--------- --SEEE-LKE AFRVFDKDQN -GFISAAE-- ---------- ----LRHV-- MTNLGE-KLT DEEVDEMIRE
PvulCaM7-1   --DDQISEFK EAFSLFDKDG DG-------- ----CITTKE LGTVMRSLG- QN-PTEAELQ DMINEV---D AD--GNG-TI DFPEFLNLM- ---ARKMKDT D--------- --SEEE-LKE AFRVFDKDQN -GFISAAE-- ---------- ----LRHV-- MTNLGE-KLT DEEVDEMIRE
CrCaM2       --DDQISEFK EAFSLFDKDG DG-------- ----CITTKE LGTVMRSLG- QN-PTEAELQ DMINEV---D AD--GNG-TI DFPEFLNLM- ---ARKMKDT D--------- --SEEE-LKE AFRVFDKDQN -GFISAAE-- ---------- ----LRHV-- MTNLGE-KLT DEEVDEMIKE
MdCaM6-2     --DDQISEFK EAFSLFDKDG DG-------- ----CITTKE LGTVMRSLG- QN-PTEAELQ DMINEV---D AD--GNG-TI DFPEFLNLM- ---ARKMKDT D--------- --SEEE-LKE AFRVFDKDQN -GFISAAE-- ---------- ----LRHV-- MTNLGE-KLT DEEVDEMIRE
PperCaM7-1   --DDQISEFK EAFSLFDKDG DG-------- ----CITTKE LGTVMRSLG- QN-PTEAELQ DMINEV---D AD--GNG-TI DFPEFLNLM- ---ARKMKDT D--------- --SEEE-LKE AFRVFDKDQN -GFISAAE-- ---------- ----LRHV-- MTNLGE-KLT DEEVDEMIRE
PvCaM1-2     --DDQIAEFK EAFSLFDKDG DG-------- ----CITTKE LGTVMRSLG- QN-PTEAELQ DMINEV---D AD--GNG-TI DFPEFLNLM- ---ARKMKDT D--------- --SEEE-LKE AFRVFDKDQN -GFISAAE-- ---------- ----LRHV-- MTNLGE-KLT DEEVDEMIRE
SiCaM1-2     --DDQIAEFK EAFSLFDKDG DG-------- ----CITTKE LGTVMRSLG- QN-PTEAELQ DMINEV---D AD--GNG-TI DFPEFLNLM- ---ARKMKDT D--------- --SEEE-LKE AFRVFDKDQN -GFISAAE-- ---------- ----LRHV-- MTNLGE-KLT DEEVDEMIRE
AtCaM3       --DDQISEFK EAFSLFDKDG DG-------- ----CITTKE LGTVMRSLG- QN-PTEAELQ DMINEV---D AD--GNG-TI DFPEFLNLM- ---ARKMKDT D--------- --SEEE-LKE AFRVFDKDQN -GFISAAE-- ---------- ----LRHV-- MTNLGE-KLT DEEVDEMIKE
AtCaM5       --DDQISEFK EAFSLFDKDG DG-------- ----CITTKE LGTVMRSLG- QN-PTEAELQ DMINEV---D AD--GNG-TI DFPEFLNLM- ---ARKMKDT D--------- --SEEE-LKE AFRVFDKDQN -GFISAAE-- ---------- ----LRHV-- MTNLGE-KLT DEEVDEMIKE
ThCaM3-2     --DDQISEFK EAFSLFDKDG DG-------- ----CITTKE LGTVMRSLG- QN-PTEAELQ DMINEV---D AD--GNG-TI DFPEFLNLM- ---ARKMKDT D--------- --SEEE-LKE AFRVFDKDQN -GFISAAE-- ---------- ----LRHV-- MTNLGE-KLT DEEVDEMIKE
BrCaM5-5     --DDQISEFK EAFSLFDKDG DG-------- ----CITTKE LGTVMRSLG- QN-PTEAELQ DMINEV---D AD--GNG-TI DFPEFLNLM- ---ARKMKDT D--------- --SEEE-LKE AFRVFDKDQN -GFISAAE-- ---------- ----LRHV-- MTNLGE-KLT DEEVDEMIKE
BrCaM5-4     --DDQISEFK EAFSLFDKDG DG-------- ----CITTKE LGTVMRSLG- QN-PTEAELQ DMINEV---D AD--GNG-TI DFPEFLNLM- ---ARKMKDT D--------- --SEEE-LKE AFRVFDKDQN -GFISAAE-- ---------- ----LRHV-- MTNLGE-KLT DEEVDEMIKE
BrCaM5-3     --DDQISEFK EAFSLFDKDG DG-------- ----CITTKE LGTVMRSLG- QN-PTEAELQ DMINEV---D AD--GNG-TI DFPEFLNLM- ---ARKMKDT D--------- --SEEE-LKE AFRVFDKDQN -GFISAAE-- ---------- ----LRHV-- MTNLGE-KLT DEEVDEMIKE
BrCaM5-2     --DDQISEFK EAFSLFDKDG DG-------- ----CITTKE LGTVMRSLG- QN-PTEAELQ DMINEV---D AD--GNG-TI DFPEFLNLM- ---ARKMKDT D--------- --SEEE-LKE AFRVFDKDQN -GFISAAE-- ---------- ----LRHV-- MTNLGE-KLT DEEVDEMIKE
CrCaM5       --DDQISEFK EAFSLFDKDG DG-------- ----CITTKE LGTVMRSLG- QN-PTEAELQ DMINEV---D AD--GNG-TI DFPEFLNLM- ---ARKMKDT D--------- --SEEE-LKE AFRVFDKDQN -GFISAAE-- ---------- ----LRHV-- MTNLGE-KLT DEEVDEMIKE
ThCaM5-1     --DDQISEFK EAFSLFDKDG DG-------- ----CITTKE LGTVMRSLG- QN-PTEAELQ DMINEV---D AD--GNG-TI DFPEFLNLM- ---ARKMKDT D--------- --SEEE-LKE AFRVFDKDQN -GFISAAE-- ---------- ----LRHV-- MTNLGE-KLT DEEVDEMIKE
BdCaM1-2     --DDQIAEFK EAFSLFDKDG DG-------- ----CITTKE LGTVMRSLG- QN-PTEAELQ DMINEV---D AD--GNG-TI DFPEFLNLM- ---ARKMKDT D--------- --SEEE-LKE AFRVFDKDQN -GFISAAE-- ---------- ----LRHV-- MTNLGE-KLT DEEVDEMIRE
BdCaM1-1     --DDQIAEFK EAFSLFDKDG DG-------- ----CITTKE LGTVMRSLG- QN-PTEAELQ DMINEV---D AD--GNG-TI DFPEFLNLM- ---ARKMKDT D--------- --SEEE-LKE AFRVFDKDQN -GFISAAE-- ---------- ----LRHV-- MTNLGE-KLT DEEVDEMIRE
ZmCaM1-1     --DDQIAEFK EAFSLFDKDG DG-------- ----CITTKE LGTVMRSLG- QN-PTEAELQ DMINEV---D AD--GNG-TI DFPEFLNLM- ---ARKMKDT D--------- --SEEE-LKE AFRVFDKDQN -GFISAAE-- ---------- ----LRHV-- MTNLGE-KLT DEEVDEMIRE
SbCaM1-4     --DDQIAEFK EAFSLFDKDG DG-------- ----CITTKE LGTVMRSLG- QN-PTEAELQ DMINEV---D AD--GNG-TI DFPEFLNLM- ---ARKMKDT D--------- --SEEE-LKE AFRVFDKDQN -GFISAAE-- ---------- ----LRHV-- MTNLGE-KLT DEEVDEMIRE
SbCaM1-2     --DDQIAEFK EAFSLFDKDG DG-------- ----CITTKE LGTVMRSLG- QN-PTEAELQ DMINEV---D AD--GNG-TI DFPEFLNLM- ---ARKMKDT D--------- --SEEE-LKE AFRVFDKDQN -GFISAAE-- ---------- ----LRHV-- MTNLGE-KLT DEEVDEMIRE
SbCaM1-3     --DDQIAEFK EAFSLFDKDG DG-------- ----CITTKE LGTVMRSLG- QN-PTEAELQ DMINEV---D AD--GNG-TI DFPEFLNLM- ---ARKMKDT D--------- --SEEE-LKE AFRVFDKDQN -GFISAAE-- ---------- ----LRHV-- MTNLGE-KLT DEEVDEMIRE
SbCaM1-1     --DDQIAEFK EAFSLFDKDG DG-------- ----CITTKE LGTVMRSLG- QN-PTEAELQ DMINEV---D AD--GNG-TI DFPEFLNLM- ---ARKMKDT D--------- --SEEE-LKE AFRVFDKDQN -GFISAAE-- ---------- ----LRHV-- MTNLGE-KLT DEEVDEMIRE
SiCaM1-1     --DDQIAEFK EAFSLFDKDG DG-------- ----CITTKE LGTVMRSLG- QN-PTEAELQ DMINEV---D AD--GNG-TI DFPEFLNLM- ---ARKMKDT D--------- --SEEE-LKE AFRVFDKDQN -GFISAAE-- ---------- ----LRHV-- MTNLGE-KLT DEEVDEMIRE
OsCaM1-2     --DDQIAEFK EAFSLFDKDG DG-------- ----CITTKE LGTVMRSLG- QN-PTEAELQ DMINEV---D AD--GNG-TI DFPEFLNLM- ---ARKMKDT D--------- --SEEE-LKE AFRVFDKDQN -GFISAAE-- ---------- ----LRHV-- MTNLGE-KLT DEEVDEMIRE
OsCaM1-3     --DDQIAEFK EAFSLFDKDG DG-------- ----CITTKE LGTVMRSLG- QN-PTEAELQ DMINEV---D AD--GNG-TI DFPEFLNLM- ---ARKMKDT D--------- --SEEE-LKE AFRVFDKDQN -GFISAAE-- ---------- ----LRHV-- MTNLGE-KLT DEEVDEMIRE
OsCaM1-1     --DDQIAEFK EAFSLFDKDG DG-------- ----CITTKE LGTVMRSLG- QN-PTEAELQ DMINEV---D AD--GNG-TI DFPEFLNLM- ---ARKMKDT D--------- --SEEE-LKE AFRVFDKDQN -GFISAAE-- ---------- ----LRHV-- MTNLGE-KLT DEEVDEMIRE
PhCaM1-3     --DDQIAEFK EAFSLFDKDG DG-------- ----CITTKE LGTVMRSLG- QN-PTEAELQ DMINEV---D AD--GNG-TI DFPEFLNLM- ---ARKMKDT D--------- --SEEE-LKE AFRVFDKDQN -GFISAAE-- ---------- ----LRHV-- MTNLGE-KLT DEEVDEMIRE
PhCaM1-4     --DDQIAEFK EAFSLFDKDG DG-------- ----CITTKE LGTVMRSLG- QN-PTEAELQ DMINEV---D AD--GNG-TI DFPEFLNLM- ---ARKMKDT D--------- --SEEE-LKE AFRVFDKDQN -GFISAAE-- ---------- ----LRHV-- MTNLGE-KLT DEEVDEMIRE
PhCaM1-5     --DDQIAEFK EAFSLFDKDG DG-------- ----CITTKE LGTVMRSLG- QN-PTEAELQ DMINEV---D AD--GNG-TI DFPEFLNLM- ---ARKMKDT D--------- --SEEE-LKE AFRVFDKDQN -GFISAAE-- ---------- ----LRHV-- MTNLGE-KLT DEEVDEMIRE
PvCaM1-4     --DDQIAEFK EAFSLFDKDG DG-------- ----CITTKE LGTVMRSLG- QN-PTEAELQ DMINEV---D AD--GNG-TI DFPEFLNLM- ---ARKMKDT D--------- --SEEE-LKE AFRVFDKDQN -GFISAAE-- ---------- ----LRHV-- MTNLGE-KLT DEEVDEMIRE
PvCaM1-5     --DDQIAEFK EAFSLFDKDG DG-------- ----CITTKE LGTVMRSLG- QN-PTEAELQ DMINEV---D AD--GNG-TI DFPEFLNLM- ---ARKMKDT D--------- --SEEE-LKE AFRVFDKDQN -GFISAAE-- ---------- ----LRHV-- MTNLGE-KLT DEEVDEMIRE
PvCaM1-6     --DDQIAEFK EAFSLFDKDG DG-------- ----CITTKE LGTVMRSLG- QN-PTEAELQ DMINEV---D AD--GNG-TI DFPEFLNLM- ---ARKMKDT D--------- --SEEE-LKE AFRVFDKDQN -GFISAAE-- ---------- ----LRHV-- MTNLGE-KLT DEEVDEMIRE
BdCaM3       --DDQIAEFK EAFSLFDKDG DG-------- ----CITTKE LGTVMRSLG- QN-PTEAELQ DMINEV---D AD--GNG-TI DFPEFLNLM- ---ARKMKDT D--------- --SEEE-LKE AFRVFDKDQN -GFISAAE-- ---------- ----LRHV-- MTNLGE-KLT DEEVDEMVRE
PvCaM1-3     --DDQIAEFK EAFSLFDKDG DG-------- ----CITTKE LGTVMRSLG- QN-PTEAELQ DMINEV---D AD--GNG-TI DFPEFLNLM- ---ARKMKDT D--------- --SEEE-LKE AFRVFDKDQN -GFISAAE-- ---------- ----LRHV-- MTNLGE-KLT DEEVDEMIRE
BrCaM5-6     --DDQISEFK EAFSLFDKDG DG-------- ----CITTKE LGTVMRSLG- QN-PTEAELQ DMINEV---D AD--GNG-TI DFPEFLNLM- ---ARKMKDT D--------- --SEEE-LKE AFRVFDKDQN -GFISAAE-- ---------- ----LRHV-- MTNLGE-KLT DEEVDEMIRE
BrCaM5-7     --DDQISEFK EAFSLFDKDG DG-------- ----CITTKE LGTVMRSLG- QN-PTEAELQ DMINEV---D AD--GNG-TI DFPEFLNLM- ---ARKMKDT D--------- --SEEE-LKE AFRVFDKDQN -GFISAAE-- ---------- ----LRHV-- MTNLGE-KLT DEEVDEMIRE
BrCaM5-8     --DDQISEFK EAFSLFDKDG DG-------- ----CITTKE LGTVMRSLG- QN-PTEAELQ DMINEV---D AD--GNG-TI DFPEFLNLM- ---ARKMKDT D--------- --SEEE-LKE AFRVFDKDQN -GFISAAE-- ---------- ----LRHV-- MTNLGE-KLT DEEVDEMIRE
MgCaM6       --DDQISEFK EAFSLFDKDG DG-------- ----CITTKE LGTVMRSLG- QN-PTEAELQ DMINEV---D AD--GNG-TI DFPEFLNLM- ---ARKMKDT D--------- --SEEE-LKE AFRVFDKDQN -GFISAAE-- ---------- ----LRHV-- MTNLGE-KLT DEEVDEMIRE
SlCaM2-2     --DDQISEFK EAFSLFDKDG DG-------- ----CITTKE LGTVMRSLG- QN-PTEAELQ DMINEV---D AD--GNG-TI DFPEFLNLM- ---ARKMKDT D--------- --SEEE-LKE AFRVFDKDQN -GFISAAE-- ---------- ----LRHV-- MTNLGE-KLT DEEVDEMIRE
ThCaM5-2     --DDQISEFK EAFSLFDKDG DG-------- ----CITTKE LGTVMRSLG- QN-PTEAELQ DMINEV---D AD--GNG-TI DFPEFLNLM- ---ARKMKDT D--------- --SEEE-LKE AFRVFDKDQN -GFISAAE-- ---------- ----LRHV-- MTNLGE-KLT DEEVDEMIRE
SlCaM2-1     --DDQISEFK EAFSLFDKDG DG-------- ----CITTKE LGTVMRSLG- QN-PTEAELQ DMINEV---D AD--GNG-TI DFPEFLNLM- ---ARKMKDT D--------- --SEEE-LKE AFRVFDKDQN -GFISAAE-- ---------- ----LRHV-- MTNLGE-KLT DEEVDEMIRE
SlCaM3       --DDQISEFK EAFSLFDKDG DG-------- ----CITTKE LGTVMRSLG- QN-PTEAELQ DMINEV---D AD--GNG-TI DFPEFLNLM- ---ARKMKDT D--------- --SEEE-LKE AFRVFDKDQN -GFISAAE-- ---------- ----LRHV-- MTNLGE-KLT DEEVDEMIRE
StCaM2-2     --DDQISEFK EAFSLFDKDG DG-------- ----CITTKE LGTVMRSLG- QN-PTEAELQ DMINEV---D AD--GNG-TI DFPEFLNLM- ---ARKMKDT D--------- --SEEE-LKE AFRVFDKDQN -GFISAAE-- ---------- ----LRHV-- MTNLGE-KLT DEEVDEMIRE
BrCaM5-10    --DDQISEFK EAFSLFDKDG DG-------- ----CITTKE LGTVMRSLG- QN-PTEAELQ DMINEV---D AD--GNG-TI DFPEFLNLM- ---ARKMKDT D--------- --SEEE-LKE AFRVFDKDQN -GFISAAE-- ---------- ----LRHV-- MTNLGE-KLT DEEVDEMVRE
SlCaM5-2     --EDQISEFK EAFSLFDKDG DG-------- ----CITTKE LGTVMRSLG- QN-PTEAELQ DMINEV---D AD--GNG-TI DFPEFLNLM- ---ARKMKDT D--------- --SEEE-LKE AFRVFDKDQN -GFISAAE-- ---------- ----LRHV-- MTNLGE-KLT DEEVDEMIRE
StCaM3       --EDQISEFK EAFSLFDKDG DG-------- ----CITTKE LGTVMRSLG- QN-PTEAELQ DMINEV---D AD--GNG-TI DFPEFLNLM- ---ARKMKDT D--------- --SEEE-LKE AFRVFDKDQN -GFISAAE-- ---------- ----LRHV-- MTNLGE-KLT DEEVDEMIRE
AtCaM6       --DDQISEFK EAFSLFDKDG DG-------- ----CITTKE LGTVMRSLG- QN-PTEAELQ DMINEV---D AD--GNG-TI DFPEFLNLM- ---ARKMKDT D--------- --SEEE-LKE AFRVFDKDQN -GFISAAE-- ---------- ----LRHV-- MTNLGE-KLS DEEVDEMIRE
BdCaM1-3     --DEQIAEFK EAFSLFDKDG DG-------- ----CITTKE LGTVMRSLG- QN-PTEAELQ DMINEV---D AD--GNG-TI DFPEFLNLM- ---ARKMKDT D--------- --SEEE-LKE AFRVFDKDQN -GFISAAE-- ---------- ----LRHV-- MTNLGE-KLT DEEVDEMIRE
ZmCaM1-6     --DEQIAEFK EAFSLFDKDG DG-------- ----CITTKE LGTVMRSLG- QN-PTEAELQ DMINEV---D AD--GNG-TI DFPEFLNLM- ---ARKMKDT D--------- --SEEE-LKE AFRVFDKDQN -GFISAAE-- ---------- ----LRHV-- MTNLGE-KLT DEEVDEMIRE
PvulCaM5     --DEQIAEFK EAFSLFDKDG DG-------- ----CITTKE LGTVMRSLG- QN-PTEAELQ DMINEV---D AD--GNG-TI DFPEFLNLM- ---ARKMKDT D--------- --SEEE-LKE AFRVFDKDQN -GFISAAE-- ---------- ----LRHV-- MTNLGE-KLT DEEVDEMIRE
SiCaM1-3     --DEQIAEFK EAFSLFDKDG DG-------- ----CITTKE LGTVMRSLG- QN-PTEAELQ DMINEV---D AD--GNG-TI DFPEFLNLM- ---ARKMKDT D--------- --SEEE-LKE AFRVFDKDQN -GFISAAE-- ---------- ----LRHV-- MTNLGE-KLT DEEVDEMIRE
ZmCaM1-7     --DEQIAEFK EAFSLFDKDG DG-------- ----CITTKE LGTVMRSLG- QN-PTEAELQ DMINEV---D AD--GNG-TI DFPEFLNLM- ---ARKMKDT D--------- --SEEE-LKE AFRVFDKDQN -GFISAAE-- ---------- ----LRHV-- MTNLGE-KLT DEEVDEMIRE
PhCaM1-2     --DEQIAEFK EAFSLFDKDG DG-------- ----CITTKE LGTVMRSLG- QN-PTEAELQ DMINEV---D AD--GNG-TI DFPEFLNLM- ---ARKMKDT D--------- --SEEE-LKE AFRVFDKDQN -GFISAAE-- ---------- ----LRHV-- MTNLGE-KLT DEEVDEMIRE
PvCaM1-7     --DEQIAEFK EAFSLFDKDG DG-------- ----CITTKE LGTVMRSLG- QN-PTEAELQ DMINEV---D AD--GNG-TI DFPEFLNLM- ---ARKMKDT D--------- --SEEE-LKE AFRVFDKDQN -GFISAAE-- ---------- ----LRHV-- MTNLGE-KLT DEEVDEMIRE
PvCaM1-8     --DEQIAEFK EAFSLFDKDG DG-------- ----CITTKE LGTVMRSLG- QN-PTEAELQ DMINEV---D AD--GNG-TI DFPEFLNLM- ---ARKMKDT D--------- --SEEE-LKE AFRVFDKDQN -GFISAAE-- ---------- ----LRHV-- MTNLGE-KLT DEEVDEMIRE
MtCaM7       --DEQISEFK EAFSLFDKDG DG-------- ----CITTKE LGTVMRSLG- QN-PTEAELQ DMINEV---D AD--GNG-TI DFPEFLNLM- ---ARKMKDT D--------- --SEEE-LKE AFRVFDKDQN -GFISAAE-- ---------- ----LRHV-- MTNLGE-KLT DEEVDEMIRE
PvulCaM7-3   --DEQISEFK EAFSLFDKDG DG-------- ----CITTKE LGTVMRSLG- QN-PTEAELQ DMINEV---D AD--GNG-TI DFPEFLNLM- ---ARKMKDT D--------- --SEEE-LKE AFRVFDKDQN -GFISAAE-- ---------- ----LRHV-- MTNLGE-KLT DEEVDEMIRE
PvulCaM7-2   --DEQISEFK EAFSLFDKDG DG-------- ----CITTKE LGTVMRSLG- QN-PTEAELQ DMINEV---D AD--GNG-TI DFPEFLNLM- ---ARKMKDT D--------- --SEEE-LKE AFRVFDKDQN -GFISAAE-- ---------- ----LRHV-- MTNLGE-KLT DEEVDEMIRE
```

```
  GrCaM5-2  --DEQISEFK EAFSLFDKDG DG-------- ----CITTKE LGTVMRSLG- QN-PTEAELQ DMINEV---D AD--GNG-TI DFPEFLNLM- ---ARKMKDT D--------- --SEEE-LKE AFRVFDKDQN -GFISAAE-- ---------- ----LRHV-- MTNLGE-KLT DEEVDEMIRE
  ZmCaM1-8  --DEQIAEFK EAFSLFDKDG DG-------- ----CITTKE LGTVMRSLG- QN-PTEAELQ DMINEV---D AD--GNG-TI DFPEFLNLM- ---ARKMKDT D--------- --SEEE-LKE AFRVFDKDQN -GFISAAE-- ---------- ----LRHV-- MTNLGE-KLT DEEVDEMIRE
    OsCaM2  --DEQIAEFK EAFSLFDKDG DG-------- ----CITTKE LGTVMRSLG- QN-PTEAELQ DMINEV---D AD--GNG-TI DFPEFLNLM- ---AKKMKDT D--------- --SEEE-LKE AFRVFDKDQN -GFISAAE-- ---------- ----LRHV-- MTNLGE-KLT DEEVDEMIRE
    OsCaM3  --DDQIAEFK EAFSLFDKDG DG-------- ----CITTKE LGTVMRSLG- QN-PTEAELQ DMINEV---D AD--GNG-TI DFPEFLNLM- ---ARKMKDT D--------- --SEEE-LKE AFRVFDKDQN -GFISAAE-- ---------- ----LRHV-- MTNLGE-KLT DEEVEEMIRE
    GmCaM5  --DEQISEFK EAFSLFDKDG DG-------- ----CITTKE LGTVMRSLG- QN-PTEAELQ DMINEV---D AD--GNG-TI DFPEFLNLM- ---ARKMKDT D--------- --SEEE-LKE AFRVFDKDQN -GFISAAE-- ---------- ----LRHV-- MTNLGE-KLT DEEVDEMIRE
    AtCaM1  --DEQISEFK EAFSLFDKDG DG-------- ----CITTKE LGTVMRSLG- QN-PTEAELQ DMINEV---D AD--GNG-TI DFPEFLNLM- ---AKKMKDT D--------- --SEEE-LKE AFRVFDKDQN -GFISAAE-- ---------- ----LRHV-- MTNLGE-KLT DEEVEEMIRE
    AtCaM4  --DEQISEFK EAFSLFDKDG DG-------- ----CITTKE LGTVMRSLG- QN-PTEAELQ DMINEV---D AD--GNG-TI DFPEFLNLM- ---AKKMKDT D--------- --SEEE-LKE AFRVFDKDQN -GFISAAE-- ---------- ----LRHV-- MTNLGE-KLT DEEVEEMIRE
    ThCaM1  --DEQISEFK EAFSLFDKDG DG-------- ----CITTKE LGTVMRSLG- QN-PTEAELQ DMINEV---D AD--GNG-TI DFPEFLNLM- ---AKKMKDT D--------- --SEEE-LKE AFRVFDKDQN -GFISAAE-- ---------- ----LRHV-- MTNLGE-KLT DEEVEEMIRE
    ThCaM4  --DEQISEFK EAFSLFDKDG DG-------- ----CITTKE LGTVMRSLG- QN-PTEAELQ DMINEV---D AD--GNG-TI DFPEFLNLM- ---AKKMKDT D--------- --SEEE-LKE AFRVFDKDQN -GFISAAE-- ---------- ----LRHV-- MTNLGE-KLT DEEVEEMIRE
  CrCaM1-2  --DEQISEFK EAFSLFDKDG DG-------- ----CITTKE LGTVMRSLG- QN-PTEAELQ DMINEV---D AD--GNG-TI DFPEFLNLM- ---AKKMKDT D--------- --SEEE-LKE AFRVFDKDQN -GFISAAE-- ---------- ----LRHV-- MTNLGE-KLT DEEVEEMIRE
  CrCaM1-1  --DEQISEFK EAFSLFDKDG DG-------- ----CITTKE LGTVMRSLG- QN-PTEAELQ DMINEV---D AD--GNG-TI DFPEFLNLM- ---AKKMKDT D--------- --SEEE-LKE AFRVFDKDQN -GFISAAE-- ---------- ----LRHV-- MTNLGE-KLT DEEVEEMIRE
  BrCaM1-1  --DEQISEFK EAFSLFDKDG DG-------- ----CITTKE LGTVMRSLG- QN-PTEAELQ DMINEV---D AD--GNG-TI DFPEFLNLM- ---AKKMKDT D--------- --SEEE-LKE AFRVFDKDQN -GFISAAE-- ---------- ----LRHV-- MTNLGE-KLT DEEVEEMVRE
  BrCaM1-2  --DEQISEFK EAFSLFDKDG DG-------- ----CITTKE LGTVMRSLG- QN-PTEAELQ DMINEV---D AD--GNG-TI DFPEFLNLM- ---AKKMKDT D--------- --SEEE-LKE AFRVFDKDQN -GFISAAE-- ---------- ----LRHV-- MTNLGE-KLT DEEVEEMVRE
    MtCaM1  --DDQISEFK EAFSLFDKDG DG-------- ----SITTKE LGTVMRSLG- QN-PTEAELQ DMINEV---D AD--GNG-TI DFPEFLNLM- ---ARKMKDT D--------- --SEEE-LKE AFRVFDKDQN -GFISAAE-- ---------- ----LRHV-- MTNLGE-KLT DEEVDEMIRE
  SmCaM5-1  --EDQIAEFK EAFSLFDKDG DG-------- ----CITTKE LGTVMRSLG- QN-PTEAELQ DMINEV---D AD--GNG-TI DFPEFLNLM- ---ARKMKDT D--------- --SEEE-LKE AFRVFDKDQN -GFISAAE-- ---------- ----LRHV-- MTNLGE-KLT DDEVDEMIRE
    PaCaM7  --EEQISEFK EAFSLFDKDG DG-------- ----CITTKE LGTVMRSLG- QN-PTEAELQ DMINEV---D AD--GNG-TI DFPEFLNLM- ---ARKMKDT D--------- --SEEE-LKE AFRVFDKDQN -GFISAAE-- ---------- ----LRHV-- MTNLGE-KLT DEEVDEMIRE
  AcCaM5-1  --DDQISEFK EAFSLFDKDG DG-------- ----CITTKE LGTVMRSLG- QN-PTEAELQ DMINEV---D AD--GNG-TI DFPEFLNLM- ---ARKMKDT D--------- --SEEE-LKE AFRVFDKDQN -GFISAAE-- ---------- ----LRHV-- MTNLGE-KLT DEEVDEMIRE
    TcCaM7  --DDQISEFK EAFSLFDKDG DG-------- ----CITTKE LGTVMRSLG- QN-PTEAELQ DMINEV---D AD--GNG-TI DFPEFLNLM- ---ARKMKDT D--------- --SEEE-LKE AFRVFDKDQN -GFISAAE-- ---------- ----LRHV-- MTNLGE-KLT DEEVDEMIRE
  CcCaM6-4  --DDQISEFK EAFSLFDKDG DG-------- ----CITTKE LGTVMRSLG- QN-PTEAELQ DMINEV---D AD--GNG-TI DFPEFLNLM- ---ARKMKDT D--------- --SEEE-LKE AFRVFDKDQN -GFISAAE-- ---------- ----LRHV-- MTNLGE-KLT DEEVDEMIRE
  BrCaM5-1  --DDQISEFK EAFSLFDKDG DG-------- ----CITTKE LGTVMRSLG- QN-PTEAELQ DMINEV---D AD--GNG-TI DFPEFLNLM- ---ARKMKDT D--------- --SEEE-LKE AFRVFDKDQN -GFISAAE-- ---------- ----LRHV-- MTNLGE-KLT DEEVDEMIRE
  ThCaM3-1  --DDQISEFK EAFSLFDKDG DG-------- ----CITTKE LGTVMRSLG- QN-PTEAELQ DMINEV---D AD--GNG-TI DFPEFLNLM- ---ARKMKDT D--------- --SEEE-LKE AFRVFDKDQN -GFISAAE-- ---------- ----LRHV-- MTNLGE-KLT DEEVDEMIKE
    CrCaM3  --DDQISEFK EAFSLFDKDG DG-------- ----CITTKE LGTVMRSLG- QN-PTEAELQ DMINEV---D AD--GNG-TI DFPEFLNLM- ---ARKMKDT D--------- --SEEE-LKE AFRVFDKDQN -GFISAAE-- ---------- ----LRHV-- MTNLGE-KLT DEEVDEMIKE
    PtCaM7  --DDQISEFK EAFSLFDKDG DG-------- ----CITTKE LGTVMRSLG- QN-PTEAELQ DMINEV---D AD--GNG-TI DFPEFLNLM- ---ARKMKDT D--------- --SEEE-LKE AFRVFDKDQN -GFISAAE-- ---------- ----LRHV-- MTNLGE-KLT DEEVDEMIRE
  BrCaM5-9  --DDQISEFK EAFSLFDKDG DG-------- ----CITTKE LGTVMRSLG- QN-PTEAELQ DMINEV---D AD--GNG-TI DFPEFLNLM- ---ARKMKDT D--------- --SEEE-LKE AFRVFDKDQN -GFISAAE-- ---------- ----LRHV-- MTNLGE-KLT DEEVDEMVRE
  ZmCaM1-4  --DEQIAEFK EAFSLFDKDG DG-------- ----CITTKE LGTVMRSLG- QN-PTEAELQ DMINEV---D AD--GNG-TI DFPEFLNLM- ---ARKMKDT D--------- --SEEE-LKE AFRVFDKDQN -GFISAAE-- ---------- ----LRHV-- MTNLGE-KLT DEEVDEMIRE
  PpCaM5-1  --EDQIAEFK EAFSLFDKDG DG-------- ----SITTKE LGTVMRSLG- QN-PTEAELQ DMISEV---D AD--GNG-TI DFAEFLNLM- ---ARKMKDS D--------- --SEEE-LKE AFRVFDKDQN -GFISAAE-- ---------- ----LRHV-- MTNLGE-KLT DDEVDEMIRE
  ZmCaM1-2  --DDQIAEFK EAFSLFDKDG DG-------- ----CITTKE LGTVMRSLG- QN-PTEAELQ DMINEV---D AD--GNG-TI DFPEFLNLM- ---ARKMKDT D--------- --SEEE-LKE AFRVFDKDQN -GFISAAE-- ---------- ----LRHV-- MTNLGE-KLT DEEVDEMIRE
    PhCaM3  --DDQIAEFK EAFTLFDKDG DG-------- ----CITTKE LGTVMRSLG- QN-PTEAELQ DMIAEV---D AD--SNG-TV DFPEFLNLM- ---ARRMKDS D--------- --SEEE-LRE AFRVFDKDQN -GFISAAE-- ---------- ----LRHV-- MANLGE-RLT DAEVDEMVRE
 PvCaM1-10  --DDQIAEFK EAFSLFDKDG DG-------- ----CITTKE LGTVMRSLG- QN-PTEAELQ DMIAEV---D AD--GNG-TV DFPEFLSLM- ---ARRMKDS D--------- --SEEE-LKE AFRVFDKDQN -GFISAAE-- ---------- ----LRHV-- MANLGE-RLT DAEVDEMVRE
  SbCaM1-6  --EDQIAEFK EAFSLFDKDD DG-------- ----CITTKE LGTVMRSLG- QN-PTEAELQ DMIAEV---D SN--NSG-TI DLQEFLGLM- ---ARKMKDT E--------- --SEEELRE AFRVFDKDQD -GFISAAE-- ---------- ----LRHV-- MTNLGE-KLS NEEVGEMIRE
    VvCaM7  --DDQISEFK EAFSLFDKDG DG-------- ----CITTKE LGTVMRSLG- QN-PTEAELQ DMINEV---D AD--GNG-TI DFPEFLNLM- ---ARKMKDT D--------- --SEEE-LKE AFRVFDKDQN -GFISAAE-- ---------- ----LRHV-- MTNLGE-KLT DEEVDEMIRE
  ZmCaM1-3  --DDQIAEFK EAFSLFDKDG DG-------- ----CITTKE LGTVMRSLG- QN-PTEAELQ DMINEV---D AD--GNG-TI DFPEFLNLM- ---ARKMKDT D--------- --SEEE-LKE AFRVFDKDQN -GFISAAE-- ---------- ----LRHV-- MTNLGE-KLT DEEVDEMIRE
  PaCaM5-2  --EEQISEFK EAFSLFDKDG DG-------- ----CITTKE LGTVMRSLG- QN-PTEAELQ DMINEV---D AD--GNG-TI DFAEFLNLM- ---ARKVKDT D--------- --SEEE-LKE AFRVFDKDQN -GFISAAE-- ---------- ----LRHV-- MTNLGE-KLT DEEVDEMIRE
    AcCaM6  --EEQISEFK EAFSLFDKDG DG-------- ----CITTKE LGTVMRSLG- QN-PTEAELQ DMINEV---D AD--QNG-TI DFPEFLNLM- ---ARKMKDT D--------- --SEEE-LKE AFKVFDKDQN -GFISAAE-- ---------- ----LRHV-- MTNLGE-KLT DEEVDEMIRE
  VvCaM5-1  --EEQIAEFK EAFSLFDKDG DG-------- ----CITTKE LGTVMRSLG- QN-PTEAELQ DMINEV---D AD--QNG-TI DFPEFLNLM- ---ARKMKDT D--------- --SEEE-LKE AFKVFDKDQN -GFISAAE-- ---------- ----LRHV-- MTNLGE-KLT DEEVDEMIRE
  StCaM2-1  --EEQIAEFK EAFSLFDKDG DG-------- ----CITTKE LGTVMRSLG- QN-PTEAELQ DMISEV---D AD--QNG-TI DFPEFLNLM- ---ARKMKDT D--------- --SEEE-LKE AFKVFDKDQN -GFISAAE-- ---------- ----LRHV-- MTNLGE-KLT DEEVDEMIRE
    PtCaM2  --EEQIAEFK EAFSLFDKDG DG-------- ----CITTKE LGTVMRSLG- QN-PTEAELQ DMINEV---D AD--QNG-TI DFPEFLNLM- ---ARKMKDT D--------- --SEEE-LKE AFKVFDKDQN -GFISAAE-- ---------- ----LRHV-- MTNLGE-KLT DEEVEEMIRE
    PtCaM3  --EEQIAEFK EAFSLFDKDG DG-------- ----CITTKE LGTVMRSLG- QN-PTEAELQ DMISEV---D AD--QNG-TI DFPEFLNLM- ---ARKMKDT D--------- --SEEE-LKE AFKVFDKDQN -GFISAAE-- ---------- ----LRHV-- MTNLGE-KLT DEEVEEMIRE
    MgCaM7  --EEQIAEFK EAFSLFDKDG DG-------- ----CITSKE LGTVMRSLG- QN-PTEAELQ DMINEV---D AD--QNG-TI DFPEFLNLM- ---ARKMKDT D--------- --SEEE-LKE AFKVFDKDQN -GFISAAE-- ---------- ----LRHV-- MANLGE-KLT DEEVDEMIRE
  PaCaM5-1  --EDQIAEFK EAFSLFDKDG DG-------- ----CITTKE LGTVMRSLG- QN-PTEAELQ DMISEV---D AD--GNG-TI DFPEFLNLM- ---ARKMKDT D--------- --SEEE-LKE AFKVFDKDQN -GFISAAE-- ---------- ----LRHV-- MTNLGE-KLT DEEVDEMIRE
  PpCaM5-4  --EEQIAEFK EAFSLFDKDG DG-------- ----SITTKE LGTVMRSLG- QN-PTEAELQ DMINEV---D AD--GNG-TI DFPEFLNLM- ---ARKMKDT D--------- --SEEE-LKE AFKVFDKDQN -GFISAAE-- ---------- ----LRHV-- MTNLGE-KLT DEEVDEMIRE
  PpCaM5-2  --EEQIAEFK EAFSLFDKDG DG-------- ----SITTKE LGTVMRSLG- QN-PTEAELQ DMINEV---D AD--GNG-TI DFAEFLNLM- ---ARKMKDT D--------- --SEEE-LKE AFRVFDKDQN -GFISAAE-- ---------- ----LRHV-- MTNLGE-KLT DDEVDEMIRE
    PpCaM7  --EDQIAEFK EAFSLFDKDG DG-------- ----SITTKE LGTVMRSLG- QN-PTEAELQ DMINEV---D AD--GNG-TI DFAEFLNLM- ---ARKMKDT D--------- --SEEE-LKE AFRVFDKDQN -GFISAAE-- ---------- ----LRHV-- MTNLGE-KLT DDEVDEMIRE
    AtCaM2  --DDQISEFK EAFSLFDKDG DGMLHPPFPS IIVGCITTKE LGTVMRSLG- QN-PTEAELQ DMINEV---D AD--GNG-TI DFPEFLNLM- ---ARKMKDT D--------- --SEEE-LKE AFRVFDKDQN -GFISAAE-- ---------- ----LRHV-- MTNLGE-KLT DEEVDEMIKE
  SmCaM5-2  --QEQLAEFK EAFSLFDKDG DG-------- ----CITTKE LGTVMRSLG- QN-PTEAELQ DMINEV---D AD--GNG-TI DFAEFLSLM- ---ARKMKDT D--------- --SEEE-LKE AFRVFDKDQN -GFISAVE-- ---------- ----LRHV-- MTNLGE-KLT DEEVDEMIRE
  SmCaM5-3  --EEQISEFK EAFSLFDKDG DG-------- ----CITTKE LGTVMRSLG- QN-PTEAELQ DMINEI---D AD--GSG-TV DFPEFLNLM- ---ARKMKDT D--------- --SEEE-LKE AFRVFDKEQN -GFISAAE-- ---------- ----LRHV-- MTNLGE-KLT DDEVDEMIRE
 CsubCaM5-1 --DEQVAEFK EAFALFDKDG DG-------- ----TITTKE LGTVMRSLG- QN-PTEAELQ DMINEV---D AD--GNG-TI DFPEFLNLM- ---ARKMKDS D--------- --SEEE-LRE AFKVFDKDGN -GFISAAE-- ---------- ----LRHV-- MTNLGE-KLT DEEVDEMIRE
  MpCaM5-2  --DEQIAEFK EAFALFDKDG DG-------- ----TITTKE LGTVMRSLG- QN-PTEAELQ DMINEV---D AD--GNG-TI DFPEFLNLM- ---ARKMKDT D--------- --SEEE-LKE AFKVFDKDGN -GFISAAE-- ---------- ----LRHV-- MTNLGE-KLT DEEVDEMIRE
    OlCaM5  --DEQIAEFK EAFALFDKDG DG-------- ----TITTKE LGTVMRSLG- QN-PTEAELQ DMINEV---D AD--GNG-TI DFPEFLNLM- ---ARKMKDT D--------- --SEEE-LQE AFKVFDKDGN -GTISAAE-- ---------- ----LRHV-- MTNLGE-KLT DEEVDEMIRE
  FvCaM7-1  --EEQIAEFK EAFCLFDKDG DG-------- ----CITTKE LGTVMRSLG- QN-PTEAELQ DMINEV---D AD--QNG-TI DFSEFLNLM- ---ARKMKDT D--------- --SEEE-LRE AFKVFDKDQN -GYISAAE-- ---------- ----LRHV-- MANLGE-KLT DEEVDEMIRE
 PperCaM7-2 --EEQIAEFK EAFCLFDKDG DG-------- ----CITTKE LGTVMRSLG- QN-PTEAELQ DMINEV---D AD--QNG-TI DFSEFLNLM- ---ARKMKDT D--------- --SEEE-LRE AFKVFDKDQN -GFISAAE-- ---------- ----LRHV-- MANLGE-KLT DEEVDEMIRE
    MdCaM2  --EEQIAEFK EAFCLFDKDG DG-------- ----CITTKE LGTVMRSLG- QN-PTEAELQ DMISEV---D AD--QNG-TI DFSEFLNLM- ---ARKMKDT D--------- --SEEE-LKE AFKVFDKDQN -GFISAAE-- ---------- ----LRHV-- MANLGE-KLT DEEVNEMIRE
    MdCaM3  --EEQIAEFK EAFCLFDKDG DG-------- ----CITTKE LGTVMRSLG- QN-PTEAELQ DMISEV---D AD--QNG-TI DFSEFLNLM- ---ARKMKDT D--------- --SEEE-LKE AFKVFDKDQN -GFISAAENV RSGKLIGTIC LIVQLRHV-- MANLGE-KLT DEEVSEMIRE
    PaCaM3  --EEQISEFK EAFSLFDKDA DG-------- ----CITTKE LGTVMRSLG- QN-PTEAEVQ DMINEV---D AD--GNG-TV DFPEFLNLM- ---GRKMKYT D--------- --SEDE-LKE AFKVFDKDQD -GLISAAE-- ---------- ----LRHV-- MTSLGE-KLT DEEVDEMIKE
    GrCaM3  --DEQIAEFK EAFSLFDKDG DG-------- ----CITTKE LGTVMRSLG- QN-PTEAELQ GMINEV---G AD--QNG-TI DFPEFLNLI- ---ARKMKDT E--------- --SEEE-LKE AFKVFDKDQN -GFISATD-- ---------- ----LRHA-- MTNLGE-ILT DEEVDEMIHE
 CreinCaM6- --EEQIAEFK EAFALFDKDG DG-------- ----TITTKE LGTVMRSLG- QN-PTEAELQ DMISEV---D AD--GNG-TI DFPEFLMLM- ---ARKMKET D--------- --HEDE-LRE AFKVFDKDGN -GFISAAE-- ---------- ----LRHV-- MTNLGE-KLS EEEVDEMIRE
  VcCaM6-2  --EEQIAEFK EAFALFDKDG DG-------- ----TITTKE LGTVMRSLG- QN-PTEAELQ DMISEV---D AD--GNG-TI DFPEFLMLM- ---ARKMKDH D--------- --HEDE-LRE AFKVFDKDGN -GFISAAE-- ---------- ----LRHV-- MTNLGE-KLS DEEVDEMIRE
  PaCaM1-1  --EEQIAEFR EAFSLFDRDG DG-------- ----SITTKE LATVIRSLG- QN-PTEAEIQ DMINEV---D TD--GNG-TI DFREFLDLM- ---AHKIKDL D--------- --SDEE-LRE AFKVFDKDQN -GYISAAE-- ---------- ----LRHV-- MINLGE-KLT EEEVELMIKE
 BrCaM5-11  --DDQISEFK EAFSLFDKDG DG-------- ----CITTKE LGTVMRSLG- QN-PTEAELQ DLMNEA---D AD--GNG-TI DFPEFLNLM- ---AH---EG L--------- --IQWKSLKK PSGFFDKDQN -GFISAAE-- ---------- ----LRHV-- MKNLGE-KLT DEEVDEMIRE
    AtCaM8  --KDQITEFK EAFCLFDKDG DG-------- ----CITVEE LATVIRSLD- QN-PTEQELH DIITEI---D SD--SNG-TI EFAEFLNLM- ---AKKLQES D--------- --AEEE-LKE AFKVFDKDQN -GYISASE-- ---------- ----LSHV-- MINLGE-KLT DEEVEQMIKE
    CrCaM8  --KDQITEFK EAFCLFDKDG DG-------- ----CITVEE LATVIRSLD- QN-PTEQELQ EIITEI---D SD--GNG-TI EFAEFLNLI- ---AKKLQEN D--------- --AEEE-LKE AFKVFDKDQN -GYISASE-- ---------- ----LSHV-- MINLGE-KLT DEEVEQMIKE
    ThCaM8  --KDQIIEFK EAFCLFDKDG DG-------- ----CITVDE LATVIRSLD- QN-PTEQELQ DIITEI---D SD--GNG-TI EFAEFLNLM- ---AKKLQEN D--------- --VEEE-LKE AFKVFDKDQN -GYISASE-- ---------- ----LSHV-- MINLGE-KLT DEEVEQMIKE
  MgCaM5-5  --KDQIIEFQ EAFSLFDKNG DG-------- ----CITIEE LATVIRSLD- QN-PTVEKLR DMINEV---D SD--GNG-TI EFEEFMTLM- ---AKKMKES D--------- --AEEE-LKE AFKVFDKDQN -GYISADE-- ---------- ----LRQV-- MINLGE-KLT DEEVKQMIRE
  MgCaM8-2  --KDQIIEFQ EAFSLFDKNG DG-------- ----CITIEE LATVIRSLD- QN-PTVEELR DMINEV---D SD--GNG-TI EFEEFMTLM- ---AKKMKES D--------- --AEEE-LKE AFKVFDKDQN -GYISADE-- ---------- ----LRHV-- MINLGE-KLT DEEVKQMIKE
    CcCaM8  --NEQIVEFK EAFCLFDKDG DG-------- ----CITVEE LATVIRSLD- QN-PTEEELQ DMINEV---D SD--RNG-TI EFGEFLNLM- ---AKKMKET D--------- --AEEE-LKE AFKVFDKDQN -GYISATE-- ---------- ----LRHV-- MINLGE-KLT DDEVEQMINE
    CsCaM8  --NEQIVEFK EAFCLFDKDG DG-------- ----CITVEE LATVIRSLD- QN-PTEEELQ DMINEV---D SD--RNG-TI EFGEFLNLM- ---AKKMKET D--------- --AEEE-LKE AFKVFDKDQN -GYISATE-- ---------- ----LRHV-- MINLGE-KLT DDEVEQMINE
  PtCaM8-2  --EEQIVEFR EAFCLFDKDG DG-------- ----CITVEE LATVIRSLD- QN-PTEEELH DMISEV---D SD--RNG-TI EFAEFLSLM- ---AKKMKET D--------- --AEEE-LKE AFKVFDKDQN -GYISANE-- ---------- ----LRHV-- MINLGE-KLT DEEVEQMIKE
  PtCaM8-1  --EEQMVEFK EAFCLFDKDG DG-------- ----CITIDE LATVIRSLD- QN-PTEEELQ DMISEV---D SD--GNG-TI EFAEFLTLM- ---AKKTKET D--------- --AEEE-LKE AFKVFDKDQN -GYISANE-- ---------- ----LRHV-- MINLGE-KLT DEEVDQMIKE
 CsatCaM8-3 --EEQIVEFK EAFCLFDKDG DG-------- ----CITIEE LATVIRSLD- QN-PTEEELQ DMIKEV---D VD--GNG-TI EFAEFLNLM- ---AKKIKET D--------- --AEEE-LKE AFKVFDKDQN -GYISATE-- ---------- ----LRHV-- MINLGE-KLT DDEVEQMIKE
  GrCaM8-1  --EEQIVEFK EAFCLFDKDG DG-------- ----CITVEE LATVIRSLD- QN-PTEEELQ DMITEV---D AD--GNG-TI EFAEFLNLM- ---AKKMKET D--------- --AEEE-LKE AFKVFDKDQN -GYISANE-- ---------- ----LRHV-- MINLGE-KLT DEEVEQMIKE
  PperCaM8  --EEQIVEFK EAFCLFDKDG DG-------- ----CITVDE LATVIRSLD- QN-PTEEELQ DMISEV---D VD--GNG-TI EFAEFLSLM- ---ANKMKET D--------- --AEEE-LKE AFKVFDKDQN -GYISATE-- ---------- ----LRHV-- MINLGE-KLT DEEVEQMIKE
  VvCaM8-1  --EEQIVEFK EAFCLFDKDG DG-------- ----CITVEE LATVIRSLD- QN-PTEEELQ DMIREV---D AD--GNG-SI EFAEFLNLM- ---AKKVKET D--------- --AEEE-LKE AFKVFDKDQN -GYISATE-- ---------- ----LRHV-- MINLGE-KLT DEEVEQMIRE
  GmCaM8-1  --EEQISEIK EAFGLFDKDG DG-------- ----CITVEE LATVIRSLD- QN-PTEEELQ DMINEV---D AD--GNG-TI EFVEFLNLM- ---AKKMKET D--------- --EEED-LKE AFKVFDKDQN -GYISASE-- ---------- ----LRHV-- MINLGE-KLT DEEVEQMIEE
  GmCaM8-2  --EEQIGEIK EAFGLFDKDG DG-------- ----CITVEE LATVIRSLD- QN-PTEEELQ DMINEV---D TD--GNG-TI EFVEFLNLM- ---AKKMKET D--------- --AEED-LKE AFKVFDKDQN -GYISASE-- ---------- ----LRHV-- MINLGE-KLT DEEVEQMIKE
 PvulCaM8-1 --AEQIGEIK EAFGLFDKDG DG-------- ----CITVEE LATVIRSLD- QN-PTEEELQ DMINEV---D SD--GNG-TI EFVEFLTLM- ---AKKMKET D--------- --AEED-LKE AFKVFDKDQN -GYISASE-- ---------- ----LRHV-- MINLGE-KLS DAEVEQMIKE
    MtCaM8  --EEQIVDFK EAFSLFDKDG DG-------- ----CITVEE LATVIRSLD- QN-PTEEELQ DMISEV---D AD--GNG-TI EFDEFLNLM- ---ARKMKDT D--------- --AEEE-LRE AFKVFDKDQN -GYISASE-- ---------- ----LRHV-- MINLGE-KLS DEEVEQMIKE
    SlCaM1  --NDQIVELQ EAFSLFDRDG DG-------- ----CITVEE LATVIRSLD- QN-PTEEELQ DMITEV---D SD--GNG-TI EFTEFLNLM- ---AKKMKDT D--------- --AEEE-LKE AFKVFDKDQN -GYISANE-- ---------- ----LRHV-- MINLGE-KLT DEEVEQMIKE
  MgCaM8-3  --EEQIVEFQ EAFSLFDKDG DG-------- ----CITIEE LATVIRSLD- QN-PTEEELH EMMNEV---D SD--RNG-TI EFAEFLNLM- ---SRKMKEI D--------- --AGEE-LKE AFKVFDKDQN -GYISANE-- ---------- ----LRQV-- LMNLGE-KLT DEEVQQMIRE
  GrCaM8-2  --EEQIVEFK EAFCLFDKDG NG-------- ----CVTVEE LAMVIGSLD- RY-PTEEELH DMITEF---D AD--GDG-AV EFAEFFNLM- ---AKKMKET D--------- --AEEE-LQE AFKVFDKDQN -GYISANE-- ---------- ----LRHV-- MINLGE-KLS DKEVEQMIKE
  FvCaM8-2  --EEQIVEFK EAFCLFDKDG DG-------- ----CITIEE LATVIRSLD- QN-PTEEELQ DMISEV---D AD--GNG-TI EFAEFLNLM- ---ANKMKET D--------- --AEEE-LKE AFKVFDKDQN -GYISANE-- ---------- ----LRHV-- MINLGE-KLT DEEVEQMIKE
  SbCaM1-7  --DEQRLAFQ EAFSLFDKNG DG-------- ----CITMEE LAAVTRSLG- LD-PSDQELN DMMSEV---D TD--GNG-II DFQEFLSLI- ---ARKMKDG D--------- --GDEE-LRE AFEVLDKDQN -GFISPIE-- ---------- ----LRTV-- MTNLGE-KMT DEEVEQMIRE
  ThCaM5-3  --DDIISEFK ETFSLFDKDG DG-------- ----FISMKE LGTVMASSG- QN-PTEAELQ DMINEV---D AD--GNG-KI DFPEFLNLM- ---ARKMKDT T--------- --DSEEELKE AFKAFDKDQN -GFISAAD-- ---------- ----LRHV-- MTNLGE-NFT DEVVDEMIRK
  FvCaM8-1  --ETQIAEFQ EAFCLIDKDS DG-------- ----VITLEE LASVIQSLD- ER-PTKEEVQ DMISEI---S AD--VTG-TI DFDEFLSIM- ---ARKMQEN V--------- --AEE--LKE AFKVFDRDQD -GYISASE-- ---------- ----LRQV-- MINLGE-RLS NEEAEQMIRE
  PvCaM1-9  --DDQIAEFK EAFSLFDKDG DG-------- ----CITTKE LGTVMRSLG- QN-PTEAELQ DMINEV---D AD--GNG-TI DFPEFLNLM- ---ARKMKDT D--------- --SEEE-LKE AFRVFDKDQN -GFISAAE-- ---------- ----LRHV-- MTNLGE-KLT DEEVDEMIRE
    SiCaM3  --KQQIEEFR EAFSLFDKDG DG-------- ----TITSKE LGTVMRSLG- QS-PSEEELQ DMVKEV---D AD--GSG-AI DFQEFLTLL- ---ARQMREA NG-------- --ADEDELRE AFRVFDQDQN -GFISRDE-- ---------- ----LGHV-- LQNLGE-RLS DDELAEMLRE
    SbCaM3  --KKQIEEFR EAFSLFDKDG DG-------- ----TITTKE LGTVMRSLG- QS-PTEEELQ GMVDEV---D AD--GSG-AI DFQEFLTLL- ---ARQMQEA SG-------- --ADEDELRE AFRVFDQDQN -GFISRDE-- ---------- ----LRHV-- LQNLGE-KLS DDELAEMLRE
    ZmCaM3  --KKQIEEFR EAFSLFDKDG DG-------- ----TITTKE LGTVMRSLG- QT-PTEEELQ GMVDEV---D AD--GSG-AI DFQEFLTLM- ---ARQMREA SG-------- --ADEEELRE AFRVFDQDQN -GFISREE-- ---------- ----LRHV-- LQNLGE-RLS DDELAEMLRE
  SbCaM1-5  --EEEIAAFT EVFALFDKNS DG-------- ----FITSEE LGTVMKSLG- QN-LTGSELQ DMITEV---D AD--GNG-TI EFPEFLNLM- ---AYNLKDT D--------- --SEEE-VKE AFKMFDKDRD -GYISAAE-- ---------- ----LRDM-- MANLGE-QLT DEEVKDMIRE
  AcCaM5-3            MIVEVDR SPR------- ----CITTKE LGTVMRSLG- QN-PTEADLQ DMINEV---D AD--GNG-TI DFPEFLNLM- ---ARKMKDT D--------- --SEEE-LKE AFRVFDKDQN -GFISAAE-- ---------- ----LRHV-- MTNLGE-KLT DEEVDEMIRE
    AcCaM8  --REQIDEFH EVFCWFDMDG DG-------- ----CITIQE LETVIRSLD- QHPTEEDVLK QMISEV---D VN--RNG-TI EFGEFLSLM- ---ARKMKEV DA-------- --EEE--LTE AFRVFDKDQD -GYISATE-- ---------- ----LRHV-- MLNLGE-TLT DEEVELMIKE
    CpCaM8  --EEQIAEFQ EAFCLFDKDG DG-------- ----CITMEE LEIAMRSVD- HHHPSEEELQ NMMAEV---D AD--GNG-TI EFGEFLSLM- ---ARKMKEA EA-------- --EEE--LRE AFKVFDKDQD -GYISPNE-- ---------- ----LRHV-- MINLGE-RLT DEEAEQMIRE
  TcCaM9-2  --EEQIAEFQ EAFFLFDKDG DG-------- ----CITMEE LAIAIKSLD- EN-PTEEELQ NMINEV---D TD--GNG-TI EFGEFLNLM- ---ARKMKEA EA-------- --EEE--LKE AFRVFDKDQD -GYISPFE-- ---------- ----LRQV-- MINLGE-KLT DEEIEQMIRE
    CcCaM5  --EDQIAEFQ EAFCMLDRDG DG-------- ----CITIEE LAVAIKSLD- QN-PTEEELR NMISEV---D VN--GNG-TI EFGEFLNLM- ---ARKMKEN EA-------- --QEE--LKE AFKVFDKDQD -GYISPNE-- ---------- ----LRHV-- MMNIGE-KVT DEELEQMVRE
  MeCaM8-2  --EEQISEFQ EAFCLLDKDG DG-------- ----RITFEE LATAIKSLD- QN-PTEEELQ SMINEV---D VD--GNG-TI EFGEFLNLK- ---ARKMKET EA-------- --EEE--LKE AFKVFDKDQD -GYISPNE-- ---------- ----LRHV-- MINLGE-KLT DEEVEQMIRE
  MeCaM8-1  --EEQIFEFQ EAFCLLDKDG DG-------- ----RITFDE LATAIKSLE- LN-PTEEELQ RMINEV---D VN--GNG-TI EFGEFLNLM- ---ARKMKES DA-------- --EEE--LKE AFKVFDKDRD -GYISPNE-- ---------- ----LRHV-- MINLGE-KLT DEELEQMIKE
  PtCaM8-3  --EEQTAEFQ EAFCLFDKDG DG-------- ----CITFEE LATVIKSLD- DS-ATDEELH IMISEV---D VD--GNG-TI EFGEFLNLM- ---ARKMREN DA-------- --AEE--LKE AFKVFDKDQD -GYISPNE-- ---------- ----LRHV-- MINLGE-QLT DEELEQMIRE
  VvCaM5-2  --AEQIAEFQ EAFCLFDMDG DG-------- ----CITLDE LATVMKSLE- HS-TTKEELQ TMMDEV---D VD--GNG-TI EFGEFLNLM- ---ARKMKES EA-------- --EEE--LKE AFKVFDKDQD -GYISANE-- ---------- ----LRNV-- MFNLGE-RLT DEEAEQMIRE
    CcCaM4  --QYQIAEFQ KAFYLLDKDG DG-------- ----CITIDE LAAAIRSLD- QN-PTKEELR LMIDEV---D AN--GNG-TI EFEEFLDLM- ---AKKMRET KA-------- --QEE--MKE AFKVFDKNRD -GFISPNE-- ---------- ----LRHV-- MLNLGE-KVT DEELEQMVKD
 PvulCaM8-3 --EEQIGEFL EAFCLFDKDG DG-------- ----CINIEE LGTAIRSLD- EN-PTLEELQ IMMSEV---D TD--CNG-TI EFGEFLNLM- ---ARKMKES EA-------- --EEE--LKE AFRVFDKDQD -GYISASE-- ---------- ----LRSV-- MRTIGE-KVT EEEVAQMVKE
  GmCaM8-3  --EDLIVEFL EAFCLFDRDG DG-------- ----CITMEE LASALRTLN- QNNPRKEELQ IMMNEV---D MD--GSG-TI EFGQFLNLM- ---ARKMKQS EA-------- --EEE--LKE AFKLFDKDQD -GYISPTE-- ---------- ----LLSV-- MRNIGV-KVT EEELEHMIRV
 PvulCaM8-2 --ENLIGDFL EAFCLFDRDG DG-------- ----CITMEE LASAIRVLN- QNNPRKEELQ VMMSEV---D MN--GNG-MI DFGQFLNLM- ---ARKMKQS ET-------- --EEE--LKE AFRLFDQDQD -GYISPTE-- ---------- ----LLSV-- MRNIGE-KIT EKELEEMIRV
  SmCaM1-2  --EEQLREFR DAFSLFDKDG DG-------- ----SITTKE LGIVMRSLG- QN-PSDTELL DMINEV---D VD--GNG-TI DWTEFLVLM- ---ARKMKDA D--------- ---AEEDLKE AFTVLDRNRD -GFITEIE-- ---------- ----LKHV-- MHQLGE-SFT DEEIADMVRE
  SlCaM5-1  KEENQLLQFK EAINLFDKDV HG-------- ----CITIEE LATVIRSLD- EN-PTEEELC HMISEV---D ADHHDNG-TM EFIEFLNLI- ---TKKMKET DL-------- --EEE--LKE VFKVFDKDQN -GFISATD-- ---------- ----LRHV-- MINLGE-KLT EEEAQHMIRE
    StCaM8  KEENQLVQFK EAINLFDKDV HG-------- ----CITIEE LATVIRSLD- EN-PTEEELC HMISEV---D ADHHDNG-TM EFNEFLNLI- ---TKKMKET DL-------- --EEE--LKE VFKVFDKDQN -GFISATD-- ---------- ----LRHV-- MINLGE-KLT EEEAEQMIRE
  SmCaM1-1  --EEQVSELK EAFSLFDKDG DE-------- ----RITTRE LGAVMKSLG- LH-PTEVELQ DMIDEV---D KD--KSG-TV EFPEFVALM- ---ARKIRGA EC-------- --EEE--LKE AFRVFDRDQN -GYISAVE-- ---------- ----LRQV-- MASMGE-KLG QEELEEMMRE
  CcCaM6-2  --EDQIAEFQ EAFCMIDKDS DG-------- ----LITMKD LATVIQSAQ- DEHPRKEEVQ EMISEV---D FD--GNG-SI DFLEFLTIM- ---GRKMKEN VS-------- --EE---LKE AFKVFDRDQD -GFISAAE-- ---------- ----LRNV-- MMNLGE-RLS DEETEQMIRE
  CsCaM5-1  --EDQIAEFQ EAFCMIDKDS DG-------- ----LITMKD LATVIQSAQ- DEHPRKEEVQ EMISEV---D FD--GNG-SI DFLEFLTIM- ---GRKMKEN VS-------- --EE---LKE AFKVFDRDQD -GFISAAE-- ---------- ----LRNV-- MMNLGE-RLS DEETEQMIRE
 CsatCaM8-1 --EDQIADFR EAFCLIDKDA DG-------- ----FITMEE LGAVIQSL-- DVHPTKNEIR DMISEV---D VD--NNG-TI DFDEFLNVM- ---ARKMKDN VT-------- --EE---LKE AFKVFDRDQD -GYISAFE-- ---------- ----LRNV-- MINLGE-RLT DDEAEQMIRE
  MdCaM4-1  --EXQIAEFQ EAFCLIDKDS DG-------- ----LISLEE LATVIQSL-- DQRPTKEEIQ DMISEI---X AD--GNG-TI DFEEFLNIM- ---SRKMKEN VA-------- --EE---LKE AFKVFDRDQD -GYISASE-- ---------- ----LRQV-- MINLGE-RLT DEEAEQMIRE
  MdCaM4-2  --EXQIAEFQ EAFCLIDKDS DG-------- ----LISLEE LATVIQSL-- DQRPTKEEIQ DMISEI---X AD--GNG-TI DFEEFLNIM- ---SRKMKEN VA-------- --EE---LKE AFKVFDRDQD -GYISASE-- ---------- ----LRQV-- MINLGE-RLT DEEAEQMIRE
  MdCaM8-1  --EGQIAEFQ EAFCLIDKDS DG-------- ----LISLEE LATVIQSL-- DQRPSKEEIX DMIXEI---G GD--GNG-TI DFEEFLNIM- ---SRKMKEN AA-------- --EE---LKE AFKVFDRDQD -GYISAFE-- ---------- ----LRQV-- MINLGE-RLS DEEXEQMIRE
  MdCaM8-2  --EGQIAEFQ EAFCLIDKDS DG-------- ----LISLEE LATVIQSL-- DQRPSKEEIX DMIXEI---G GD--GNG-TI DFEEFLNIM- ---SRKMKEN AA-------- --EE---LKE AFKVFDRDQD -GYISAFE-- ---------- ----LRQV-- MINLGE-RLS DEEXEQMIRE
  MeCaM9-2  --EEQIAEFR EAFCLIDKDS DG-------- ----FITMEE LATVIQSL-- DGHPTKEEVG DMISEV---E FD--GNG-TI DFDEFLSII- ---GRKMKEN VT-------- --EE---LKE AFKVFDRNQD -GYISANE-- ---------- ----LRQV-- MINLGE-RLT EEEAEQMIRE
  RcCaM9-1  --EDQIAEFH EAFCLIDKDS DG-------- ----FITMEE LATVIQSL-- DGHPTKEEIR DMISEV---D FD--GNG-TI DFQEFLNIM- ---GRKMKEN VV-------- --EE---LKE AFKVFDRNQD -GFISANE-- ---------- ----LRQV-- MINLGE-RLT EEEAEQMIRE
  MeCaM9-1  --VDQIADFR EAFCLIDKDS DG-------- ----FITVEE LATTIQSL-- DGHPTKEEVQ QMTSEV---E VN--GNG-TI DFQEFLNIM- ---GRKMKEN VA-------- --EE---LKE AFKVFDRNQD -GYISANE-- ---------- ----LRQV-- MINMGE-RLT EEEAEQMIRE
  VvCaM8-2  --EDQIVQFR EAFCLIDKDS DG-------- ----SITVEE LATVIQSL-- DGHPTQEEVQ DMISEV---D AD--GNR-SI DFAEFLNIM- ---ARKMKEN VA-------- --EE---IKE AFKVFDRDQD -GYISAIE-- ---------- ----LRNV-- MINLGE-RLT DEEAEQMIRE
  GmCaM9-3  --DDQIAEFH EAFSLIDKDS DG-------- ----FITVDE LTTIIRSL-- EGNPTKEEIQ NMISEV---D ID--GNG-SI DFEEFLNIM- ---GRKMKET LA-------- --EE---LKE AFKVFDRDQN -GYISATE-- ---------- ----LRHV-- MTNLGE-RLT GEEAEQMIME
  GmCaM9-4  --DDQIAEFH EAFCLIDKDS DG-------- ----FITVDE LATIIRSL-- EGNPTKEEIQ DMISEV---D ID--GNG-SI DFEEFLNIM- ---GRKMKET LA-------- --EE---LRE AFKVFDRDQN -GYISATE-- ---------- ----LRHV-- MMNLGE-RLT DEEAEQMIRE
 PvulCaM9-1 --DDQIAEFQ EAFCLIDKDC DG-------- ----FISVEE LTTTFRSI-- VGNPTKKEIQ DMISEV---D ID--GNG-SI DFEEFLSIM- ---GRKMKET LS-------- --EE---LKE AFKVFDRDED -GYISATE-- ---------- ----LRNV-- MMSLGE-RLT DEEAEQMIRD
  MgCaM9-1  --VEQIAEFR EAFSLIDKDS DG-------- ----VITVEE LALAIQSL-- NEHPTSEEIQ EMVNEA---D SD--GDG-TV DFEEFLIVM- ---SSKMKEN VV-------- --EE---IKD AFKVFDRDQD -GFISAIE-- ---------- ----LRNV-- MINMGE-RLT EEEAEQMINE
    SlCaM4  --EDQISEFR QAFWVIDKDC DG-------- ----VIRIEE LASVIQSL-- HENPTREEMQ EMMNEV---D PD--GDG-TI DFEDFLNIM- ---AKKLKDN VT-------- --EE---LKE AFKVFDRDQD -GFISANE-- ---------- ----LRNV-- MMNLGE-RLT EEEAQQMIRE
    MgCaM4  LNKEQIVEIQ EAFTMFDKDG DG-------- ----CINIEE LGIVMRSLN- QN-PTEKELR DMINEV---D SD--GNG-TI DFDEFLNLM- ---TAK-KMQ EI-------- --DE---AKE TFKVFDIDQN -GYISANE-- ---------- ----IRQ--- --KLGH-KLT DEEVNQMIRE
  MgCaM8-1        ILEFQ TSFNVFDKDG DG-------- ----CINIEE LGIVMRSLD- QN-PSEKELR DMINEV---D SD--GNG-TI DFDEFLNLM- ---TAKEKMQ VY-------- --NE---LKE AFKVFDKDQN -GYISANE-- ---------- ----LRQT-- MINLGE-KLT VEEVEQMVRE
  LuCaM6-1  LGQDQILEFH EAFYLLDKDG DG-------- ----RITMEE LGRAMKSLG- LR-PSEGELQ EMIKEV---D WD--GNG-SI EFEEFLSLM- ---ASKLEEK Q--------- --SDHLQLRE AFIVFDKDQD -GFISPNE-- ---------- ----LRHG-- MVNVGE-RIT DDEVDQMVKE
  LuCaM6-2  LGNDQILEFH EAFCLLDKDG DG-------- ----RISIQE LKTALRSLG- LH-PSERELT AMINQV---D VD--GKG-SV GFEEFLSLM- ---ASKVKEN E--------- --ADQ-ELRD AFIVFDKDQD -GFISPSE-- ---------- ----LRHV-- MMNVGE-RVT DEELEQMVKE
  CsatCaM9  SEEQIISEFQ EAFCLLDKDG DG-------- ----CITINE LATAIRSLH- HN-PTEEELQ IMMNEV---D VN--GNG-YI KFGEFFNLM- ---AKKMKEN EA-------- --EDE--LRE AFKVFDMDDD -GKISPNE-- ---------- ----LKNV-- MIHMVE-KLT DEEIEQMVNE
 CsatCaM8-2 --KEQMDQLK EAFFLFDKNK DG-------- ----RITIDE LRAEIRNLG- HN-PTEEELK EMIREV---D AD--GNG-TI EFGEFQNLM- ---SKIMKE- ---------- --ETEKKLKE AFKVFDKNQD -GYISANE-- ---------- ----LSHVYW MLNSGEEKLT DEEVFHMISE
  CreinCaM1 LPPERLEELK EAFKLFDKDG NG-------- ----HITHRE LGLVMRSLG- QN-PTEAELH QMIREV---D TN--DSG-AV EFPEFVKLM- ----MKQPEN PA-------- --DQEESLRE AFRMFDRDGN -GFINADE-- ---------- ----LKHV-- MCNLGE-ALT EQEVEDMIKE
    MpCaM1  LSEEQCAEFK EAFAIFDKDG DG-------- ----TITIKE LGVVMRSLG- QN-PTESELQ DMINEV---D ES--GDG-EL DFPEFLLLM- ---SNRMKDN GS-------- --EDE--LVE AFKVFDRDGD -GSVSVDE-- ---------- ----LMTI-- MTMLGE-RLT REEVETMIRD
  MpCaM5-4  LTRTQVKEFR DAFAIFDKDG GG-------- ----SITTDE LGDVMKSLG- QK-PSHAELD AMVREI---D AD--GNG-EI DFPEFLTMM- ---LRKMNEG NP-------- --EKE--LMD VFMVFDKDGS -GTISAEE-- ---------- ----LRSA-- MRVIGE-KLT DDEIEDAIKL
    OlCaM1  LTRAQVQEFK EAFDIFDVDG GG-------- ----TITAEE LGEVMKSLG- QK-PTRAQLE AMVREI---D AD--GDG-AI DFPEFLTMM- ---LRKMNEG DP-------- --ERE--LRD VFTVFDKDQS -GTISADE-- ---------- ----LKSV-- MKVIGE-KLT EQEIEDAIRL
  GmCaM9-1  --EDQIAIFH EAFDVVDKDS DG-------- ----FISVDE LLSIVRSLE- GN-STKEEIR EMISEV---D ID--GNGRSV NFENFLKIM- ---GRTMK-- ---------- -ENQTEELKD SFKVFDRDND -GYISATE-- ---------- ----LRQV-- MVKLGE-RLT DEEVEQMIRE
  GmCaM9-2  --EDQIAIFH EAFVVVDKDS DG-------- ----FITVDE LISIVRSLE- GN-ATKEKIQ EMISEV---D IN--GNSLSV NFEDFLKIM- ---GRTIK-- ---------- -ENLTEELKD SFKVFDRDND -GYISATE-- ---------- ----LRQV-- MVKLGE-RLT DEEVEQMIRE
 PvulCaM9-2 --EDQIAIFH EAFDVVDKDS DG-------- ----FITVEE LISIVQSLD- GN-TPKEEVQ EKISKV---D ID--GKG-SV NFEEFLNVI- ---GMTMK-- ---------- -ENLTEELKD AFKVFDRDND -GYISATE-- ---------- ----LRHV-- MMKLGE-RLT EEEVEQMIRE
    MtCaM9  --KDQIAEFW EAFCLIDKDS DG-------- ----FITVDE LITIIKALE- GN-LTKEEIQ EMIRKT---D ID--GNG-RV DFEKFLHII- ---EIKMKVK NCTINLHVSF IEYLTEELKD SFKVFDSNND -GYISATE-- ---------- ----LRHV-- MMKLGE-RLT DEEVEQMIRE
    AtCaM9  --DEQIQEFY EAFCLIDKDS DG-------- ----FITKEK LTKVMKSMG- KN-PKAEQLQ QMMSDV---D IF--GNG-GI TFDDFLYIM- ---AQNTSQE ---------- --SASDELIE VFRVFDRDGD -GLISQLE-- ---------- ----LGEG-- MKDMGM-KIT AEEAEHMVRE
    ThCaM9  --DEQIQEFY EAFCLIDKDS DG-------- ----FITKEK LKKVMKSMG- KN-PKAEQLQ EMMSDV---D IF--GNG-GI TFDDFLYIM- ---AQNTSQE ---------- --SASDELIE VFRVFDRDGD -GFISALE-- ---------- ----LGEG-- MKDMGM-KIT AEEAEHMVRE
    CrCaM9  --DEQIQEFY EAFCLIDKDS DG-------- ----FITKEK LTKVMKSMG- KN-PKAEQLQ QMMSDV---D IF--GNG-GI TFDDFLYIM- ---AQNSSQE ---------- --SVSDELID VFRVFDKDGN -GFISPLE-- ---------- ----LGEG-- MKDMGM-KIT AEEAEHMVRE
 CreinCaM8  --EEQKQEIR EAFDLFDTDG SG-------- ----TIDAKE LKVAMRALG- FE-PKKEEIK KMISEI---D KD--GSG-TI DFEEFLTMM- ---TAKMGER D--------- ---SREEILK AFRLFDDDNS -GTITIKD-- ---------- ----LRRV-- AKELGE-NLT EEELQEMIAE
  CsubCaM4  --EEQKQEIR EAFDLFDTDG SG-------- ----TIDAKE LKVAMRALG- FE-PKKEEVK KMIADI---D KD--GSG-TI DFDEFLTMM- ---TAKMDER D--------- ---PKEEIMK AFRLFDDDET -GKISFKN-- ---------- ----LKRV-- AKELGE-NMT DEELQEMIDE
  PpCaM8-2  --EEQKQEIR EAFDLFDTDG SG-------- ----TIDAKE LKVAMRALG- FE-PKKEEIK KMIADI---D KD--GSG-TI DFEEFLQMM- ---TTKMGER D--------- ---SKEEIMK AFRLFDDDET -GKISFKN-- ---------- ----LKRV-- AKELGE-NMT DEELQEMIDE
  PpCaM8-1  --DDQKQEIR EAFDLFDTDG SG-------- ----TIDAKE LKVAMRALG- FE-PKKEEIK KMIADI---D KD--GSG-TI DFDEFVHMM- ---TAKMGER D--------- ---SREEIMK AFRLFDDDDT -GTITFKD-- ---------- ----LKRV-- ARELGE-NLS DAELQEMIEE
    SlCaM8  --QQKRQEIK EAFELFDTDG SG-------- ----TIDASE LNVAMRALG- FE-MTEEQVE QMIADV---D KD--GSG-AI DFDEFVHMM- ---TAKIGER D--------- ---TKEDLAK AFNILDQDKN -GKISAAD-- ---------- ----IQQI-- AKELGE-NFS AKEIQEMIEA
  LuCaM9-2  LTEDQIDEFR KAFSLIDKNG DG-------- ----LITKEE LAMVVQSPE- IH-------- DLISEV---D LL--GDG-AM DFDEFLTVM- ---SRKMQEN ---------- ---VRDELKE AFKVFDRDQD -GFISPTD-- ---------- ----LRQV-- MINMGE-RLT EEEAEQMIRE
  LuCaM9-4  LTEDQIDEFR KAFSLIDKNG DG-------- ----LITKEE LAMVVQSTE- IH-------- DLISEV---D LL--GDG-AM DFDEFLTVM- ---SRKMQEN ---------- ---VRDELKE AFKVFDRDQD -GFISPTD-- ---------- ----LRQV-- MINMGE-RLT EEEAEQMIRE
  LuCaM9-3  LTEDQIDEFR KAFSLIDKNG DG-------- ----LITKEE LAMVIQSTE- IH-------- DLISEV---D LL--GDG-AM DFDEFLTVM- ---TRKMQEN ---------- ---VRDELKE AFKVFDRDQD -GFISPTD-- ---------- ----LRQV-- MINMGE-RLT EEEAEQMIRE
  LuCaM9-1  MLEDQIQEFR EAFCLIDRNS DG-------- ---GFITVEE LAMLIQTVD- AH-PKKQEVR DMISEV---D FL--GNE-AM SFNDFLNVM- ---ARKMEEN ---------- ---AGDELKE AFKVFDRDQD -GFISPSE-- ---------- ----LREV-- MMDMGE-RLT EEEAEHMIRE
    CsCaM4                                 CITIEE LAVAIKSLD- QN-PTEEELR NMISEV---D VN--GNG-TI EFGEFLNLM- ---ARKMKEN E--------- ---AQEELKE AFKVFDKDQD -GYISPNE-- ---------- ----LRHV-- MMNIGE-KVT DEELEQMVRE
    MgCaM9-2                               LITVDE LAYVIQSLN- GH-PTKEEIQ EMMNEF---D AD--GDG-TV DFEDFLSIM- ---ARKMKEN ---------- ---ITEELKE AFKVFDRDQD -GFISAIE-- ---------- ----LRNV-- MLNLGE-RLS DEEAEQMIRE
 CreinCaM6- MSDDRYAEYK EAFTLFDSDG DG-------- ----FITTKE LGTVLRALG- KS-PTEAEIK AIVKDI---D PD---NRGVV DYKEFEKIM- ---TRDIREY D--------- ---NEQDLKA AWKVFDKAGA -GSIATSE-- ---------- ----LKHV-- LTSVGE-KLS PEEIAEMIAE
  VcCaM6-1  MAEERYAEYK EAFTLFDSDG DG-------- ----FITTKE LGTVLRALG- KS-PTEAEIK TIVKDI---D PD---NRGVV DYKEFEKIM- ---TRDIREY D--------- ---NEQDLKA AWKVFDKAGQ -GFISKSE-- ---------- ----LKHV-- LTSVGE-KLS PEEINEMMAE
    EuCaM6  --DDQISSMK EAFTLFDTDG DG-------- ----RIAPSE LGILMRSLG- GN-PTQAQLK SIVAGE---G LT-----APF DFPRFLDLM- ---SRHLRAE ---------- --PFDRQLRD AFKVLDKEST -GYVSVAD-- ---------- ----LRHI-- LTSIGE-KLT PSEFDEWIRE
  PaCaM6-1  --EEQIAPMR EAFSLFDTDR DG-------- ----YITAAE LGTVMRSLG- EN-PTQAELK EIIKRE---E IS-----GTI DFPHFLDVM- ---KRNLKGG ---------- --SFHVQLRD AFKALDKEGT -GKISASE-- ---------- ----LRHI-- LTSVGE-KLE PSEFDEWIRE
    GrCaM9  --DDQVV--R EVFCLIDKDS DG-------- ----FISMEE LASVIQTLD- DANPIKEDQN MMMMMMVINE ADDIERKGKI DFEDFVNIM- ---ARKMQEN ---------- ---VVDELKE AFKVFDLDQD -GFISANE-- ---------- ----LRQV-- MMNMGE-KLT MEEAEQMIRE
  SiCaM1-4   MAERFTQIK AAFDLFDRNM DG-------- ----FITVED LGAVLESLG- QN-NTAFELQ SMIAMV---D SD--GDG-AI NFEEFSMLM- ---ELKPNGV D--------- ---SDEEMTR AFRMFDMDKD -GFISEAE-- ---------- ----LCHI-- MYNLGS-NLS GDEVKEMMRV
  TcCaM9-1  --EDQIAEFR EAFCLIDKDS DG-------- ----FVTMEE LATVIQTLD- GN-PTKEEVQ DMISEV---D VE--GNR-TI DFEDFLNIM- ---ATKMKEN ---------- ---VADELKE AFKVFDRDQD -GFISANE-- ---------V LSKLARAVFI FLLLFPPTKE CLTTNSSLKY
    ThCaM7     MDPTELK RVFQMFDKNG DG-------- ----TITKKE LSETLRSLG- IYIP-DKELV QMIEKI---D VN--GDG-CV DIDEFGELY- ---KTIIEEE D--------- --EEEEDMKE AFNVFDQNGD -GFITVDE-- ---------- ----LKAVLS SLGLKQGKTL DD-CKKMIKQ
    CrCaM6  VDMANPDELK KVFDQFDSNG DG-------- ----KISVLE LGGVFKAMG- -TSYTETELN RVLEEV---D TD--RDG-FI NLDEFSTLC- ---RSSSSAA ---------- ---E---IRD AFDLYDQDKN -GLISAAE-- ---------- ----LHQV-- LNRLG-MSCS VEDCTRMIGP
   StCaM6-1 LYLQNMDEVQ KVFQRFDTNG DG-------- ----MISGDE LSGVISALG- -SGTSPDEVA RMMDEI---D AD--RDG-FI SLKEFAEFC- ---KGDINSV GDGG------ -VKE---LRE AFDLYDQDSN -GLISAAE-- ---------- ----LHQI-- LNRLG-ESCS VQDCTKMITS
```

```
   RcCaM9-2  AAMEVHNQFK QVFKLIDANG DG-------- ----KISSCE LSELLLCLGF DKSKATSEAE GMVREM---D CN--GDG-FV DMDEFIYTL- ---NDDGKLR GVGG------ -DNKKDYLMD AFLIFDADKN -GLISAEE-- ---------- ----LKKV-- LTNLGCDNCS LKKCRRMIKG
     FvCaM3  SSVASDEKLA KVFDHFDTNK DG-------- ----KISADE LQAYFMSIG- -EPMSINEAQ TVIKEL---D ND--GDN-LL EFEDFVKLM- ---RREDIDA ---------- --DENDDLKN AFEMFQVDKG CGCITPKG-- ---------- ----LQNM-- FNRLG-DAKS YDECVSMIRV
   MpCaM5-3  VGPKVLKELR AKYVLFDADG TG-------- ----LMDLDE LGDLMRATGL N--PVETKVK QLMQRY---D HD--KSG-DL NFSEFVDLF- ---AHELLEA E--------- --SDESMFKR AFQFFDADGS -GDIALSE-- ---------- ----FRKV-- LTELGD-PLS KQELEKFFEL
   PaCaM5-3  LSVEETKGIK DMFKMMDTDN SG-------- ----SISYDK LKAGLHKLGS Q--MDESEVQ ILMDAA---D AD--GNG-ML DYREFVAAS- ---LH--MQS I--------- --DNDDYLRK AFLFFDKDGS -GYIEIEE-- ---------- ----FREA-- LADDFG-PND IDVVNSIFNE
   PaCaM1-2  LSEEEINGLK EMFRSMDIDN SG-------- ----TLTFEE LKNDLARFGS K--ISESEVR QLMDAA---D VD--GDG-TI DYLEFITTI- ---MH--MNR M--------- --DREYHLYT SFQYFDKDSS -GYITKEE-- ---------- ----LEQA-- LKN-YG-MGD QETIKDIIDE
     CcCaM3  QFKDGLKNIK SVFEQYDKDS NG-------- ----TIDREE LKKCLE-ELQ LH-LKEEEIE DLFKSC---D ID--GSE-GI QFNEFIVLLC LIYLLTTPSS SSMSKMGSPQ LKATFDAIVE VFLFLDKNGD -GRLNKKD-- ---------- ----MVKTLN ESYPRE-KSP AHVTRTRFEE
   PpCaM5-3  VWQES--QLK EAFDMMDRDG DG-------- ----TIKAED LSHFLQYSLK SN-LSSEEIE NMISLA---D RD--GNG-AV DFDEF---MS LVSAHVPQAP ESLSSLG--- ----YEALRQ IFRVLDRNGD -DVLCSDD-- ---------- ----LSGVMG SL--GQ-CLS LEDLLAMVET
     VcCaM3  LNEDELEMCR KAFNMFDKES SG-------- ----TIDTKD LRTALS-ALG QN-PSEEEMF VMISQV---D EE--GSR-CI EFTEFVRVIQ FNKALSARDA DELDTL---- -----DAFAA LGGNIDRTG- --KISIDK-- ---------- ----LKSICE EF------EL TINLDRVAKD
     CpCaM6
   PaCaM6-2
  CreinCaM3
  CreinCaM5
     VcCaM5
  CsubCaM5-2
   MpCaM5-1
  Consensus  ...dqi.#fk #aFslfDk#g #G              cIttkE Lgtvmrs$g  qn.pteaElq d$in#!   D a#  gnG t! #FpEFlnl$    arkmk#t d.           seee.Lk# AFrvFDkDq#  GfISaaE                  Lrh!   $tnlG# klt d#EvdeM!r#

             361                                                                                                                                                                                             540
   AcCaM5-2  ADVDGDGQIN YEEFVKVMMA K
     AtCaM7  ADVDGDGQIN YEEFVKVMMA K
   CrCaM7-2  ADVDGDGQIN YEEFVKVMMA K
     CpCaM7  ADVDGDGQIN YEEFVKVMMA K
   CcCaM6-3  ADVDGDGQIN YEEFVKVMMA K
   CcCaM6-1  ADVDGDGQIN YEEFVKVMMA K
   CsCaM7-2  ADVDGDGQIN YEEFVKVMMA K
   FvCaM7-2  ADVDGDGQIN YEEFVKVMMA K
   GrCaM5-1  ADVDGDGQIN YEEFVKVMMA K
   LuCaM6-3  ADVDGDGQIN YEEFVKVMMA K
   LuCaM7-2  ADVDGDGQIN YEEFVKVMMA K
   LuCaM7-3  ADVDGDGQIN YEEFVKVMMA K
   LuCaM7-1  ADVDGDGQIN YEEFVKVMMA K
   MdCaM6-1  ADVDGDGQIN YEEFVKVMMA K
     MdCaM7  ADVDGDGQIN YEEFVKVMMA K
   MeCaM5-1  ADVDGDGQIN YEEFVKVMMA K
   MeCaM5-4  ADVDGDGQIN YEEFVKVMMA K
   MeCaM5-3  ADVDGDGQIN YEEFVKVMMA K
     MeCaM7  ADVDGDGQIN YEEFVKVMMA K
   RcCaM7-1  ADVDGDGQIN YEEFVKVMMA K
   MeCaM5-2  ADVDGDGQIN YEEFVKVMMA K
   RcCaM7-2  ADVDGDGQIN YEEFVKVMMA K
   MgCaM5-1  ADVDGDGQIN YEEFVKVMMA K
   PperCaM5  ADVDGDGQIN YEEFVKVMMA K
   MgCaM5-2  ADVDGDGQIN YEEFVKVMMA K
   MgCaM5-3  ADVDGDGQIN YEEFVKVMMA K
   MgCaM5-4  ADVDGDGQIN YEEFVKVMMA K
   PtCaM6-1  ADVDGDGQIN YEEFVKVMMA K
   PtCaM6-2  ADVDGDGQIN YEEFVKVMMA K
 PvulCaM7-1  ADVDGDGQIN YEEFVKVMMA K
     CrCaM2  ADVDGDGQIN YEEFVKVMMA K
   MdCaM6-2  ADVDGDGQIN YEEFVKVMMA KX
 PperCaM7-1  ADVDGDGQIN YEEFVKVMMA K
   PvCaM1-2  ADVDGDGQIN YEEFVKVMMA K
   SiCaM1-2  ADVDGDGQIN YEEFVKVMMA K
     AtCaM3  ADVDGDGQIN YEEFVKVMMA K
     AtCaM5  ADVDGDGQIN YEEFVKVMMA K
   ThCaM3-2  ADVDGDGQIN YEEFVKVMMA K
   BrCaM5-5  ADVDGDGQIN YEEFVKVMMA K
   BrCaM5-4  ADVDGDGQIN YEEFVKVMMA K
   BrCaM5-3  ADVDGDGQIN YEEFVKVMMA K
   BrCaM5-2  ADVDGDGQIN YEEFVKVMMA K
     CrCaM5  ADVDGDGQIN YEEFVKVMMA K
   ThCaM5-1  ADVDGDGQIN YEEFVKVMMA K
   BdCaM1-2  ADVDGDGQIN YEEFVKVMMA K
   BdCaM1-1  ADVDGDGQIN YEEFVKVMMA K
   ZmCaM1-1  ADVDGDGQIN YEEFVKVMMA K
   SbCaM1-4  ADVDGDGQIN YEEFVKVMMA K
   SbCaM1-2  ADVDGDGQIN YEEFVKVMMA K
   SbCaM1-3  ADVDGDGQIN YEEFVKVMMA K
   SbCaM1-1  ADVDGDGQIN YEEFVKVMMA K
   SiCaM1-1  ADVDGDGQIN YEEFVKVMMA K
   OsCaM1-2  ADVDGDGQIN YEEFVKVMMA K
   OsCaM1-3  ADVDGDGQIN YEEFVKVMMA K
   OsCaM1-1  ADVDGDGQIN YEEFVKVMMA K
   PhCaM1-3  ADVDGDGQIN YEEFVKVMMA K
   PhCaM1-4  ADVDGDGQIN YEEFVKVMMA K
   PhCaM1-5  ADVDGDGQIN YEEFVKVMMA K
   PvCaM1-4  ADVDGDGQIN YEEFVKVMMA K
   PvCaM1-5  ADVDGDGQIN YEEFVKVMMA K
   PvCaM1-6  ADVDGDGQIN YEEFVKVMMA K
     BdCaM3  ADVDGDGQIN YDEFVKVMMA K
   PvCaM1-3  ADVDGDGQIN YDEFVKVMMA K
   BrCaM5-6  ADVDGDGQIN YDEFVKVMMA K
   BrCaM5-7  ADVDGDGQIN YDEFVKVMMA K
   BrCaM5-8  ADVDGDGQIN YDEFVKVMMA K
     MgCaM6  ADVDGDGQIN YDEFVKVMMA K
   SlCaM2-2  ADVDGDGQIN YDEFVKVMMA K
   ThCaM5-2  ADVDGDGQIN YDEFVKVMMA K
   SlCaM2-1  ADVDGDGQIN YDEFVKVMMA K
     SlCaM3  ADVDGDGQIN YDEFVKVMMA K
   StCaM2-2  ADVDGDGQIN YDEFVKVMMA K
  BrCaM5-10  ADVDGDGQIN YDEFVKVMMA K
   SlCaM5-2  ADVDGDGQIN YDEFVKVMMA K
     StCaM3  ADVDGDGQIN YDEFVKVMMA K
     AtCaM6  ADVDGDGQIN YEEFVKVMMA K
   BdCaM1-3  ADVDGDGQIN YEEFVKVMMA K
   ZmCaM1-6  ADVDGDGQIN YEEFVKVMMA K
   PvulCaM5  ADVDGDGQIN YEEFVKVMMA K
   SiCaM1-3  ADVDGDGQIN YEEFVKVMMA K
   ZmCaM1-7  ADVDGDGQIN YEEFVKVMMA K
   PhCaM1-2  ADVDGDGQIN YEEFVKVMMA K
   PvCaM1-7  ADVDGDGQIN YEEFVKVMMA K
   PvCaM1-8  ADVDGDGQIN YEEFVKVMMA K
     MtCaM7  ADVDGDGQIN YEEFVKVMMA K
 PvulCaM7-3  ADVDGDGQIN YEEFVKVMMA K
 PvulCaM7-2  ADVDGDGQIN YEEFVKVMMA K
   GrCaM5-2  ADVDGDGQIN YDEFVKVMMA K
   ZmCaM1-8  ADVDGDGQIN YDEFVKVMMA K
     OsCaM2  ADVDGDGQIN YEEFVKVMMA K
     OsCaM3  ADVDGDGQIN YDEFVKVMMA K
     GmCaM5  ADVDGDGQIN YEEFVKVMMA K
     AtCaM1  ADVDGDGQIN YEEFVKIMMA K
     AtCaM4  ADVDGDGQIN YEEFVKIMMA K
     ThCaM1  ADVDGDGQIN YEEFVKIMMA K
     ThCaM4  ADVDGDGQIN YEEFVKIMMA K
   CrCaM1-2  ADVDGDGQIN YEEFVKIMMA K
   CrCaM1-1  ADVDGDGQIN YEEFVKIMMA K
   BrCaM1-1  ADVDGDGQIN YEEFVKIMMA K
   BrCaM1-2  ADVDGDGQIN YEEFVKIMMA K
     MtCaM1  ADVDGDGQIN YDEFVKVMMA K
   SmCaM5-1  ADVDGDGQIN YEEFVKMMLA K
     PaCaM7  ADVDGDGQIN YEEFVKVMMA K
   AcCaM5-1  ADVDGDGQIN YEEFVKVMMA KRRQKRSEGD SVMNRKKDAV SNGKRRDRIR SKCVVL
     TcCaM7  ADVDGDGQIN YEEFVKVMMA KRTQRTVSSH RSQNEEKPNA GRKRRKRVKI RDCVFL
   CcCaM6-4  ADVDGDGQIN YEEFVKVMMA KRRRKRIE-E RSSAMENEKE GKKVDGERKR RRCAIL
   BrCaM5-1  ADVDGDGQIN YEEFVKVMMA KRRRKKS--- MAGKPSS-ST ERKEERRRRK SHCRIL
   ThCaM3-1  ADVDGDGQIN YEEFVKVMMA KRRRKRN--- MTAKRSS-TT EYKEENRRRK SHCRIL
     CrCaM3  ADVDGDGQIN YEEFVKVMMA KRRGKRV--- MTTRRSSNSV EYKEKNGRRK SHCRIL
     PtCaM7  ADVDGDGQIN YEEFVKVMMA KKRKMR---- -SDSKEGSSI GGENNHKPEK KCCNVL
   BrCaM5-9  ADVDGDGQIN YDEFVKVMMA NKLRKCATNK KKKTKKKKTK NAKQNEKEMI RQKPANSESH CIPVPSCMLR FCYRPSFSSI L
   ZmCaM1-4  ADVDGDGQIN YEEFVKVMMA KSCPRLSPPP RSPLPAPREF KLQQ------ RQDPTPPWSR ERRGRSCSTR RWAWAT
   PpCaM5-1  ADVDGDGQIN YEEFVKMMMA KFASSDTSFK PSAAVQSPCH HFFICGFLAA MPSSTFSLTL
   ZmCaM1-2  ADVDGDGQIN YEEFVKVMMA KWSHLENLNM QQGNVSVIFY LPKVISPLGV LPYL
     PhCaM3  ADVDGDGRIN FDEFVKVMMA KRRSKRTEEK A-ARGKKKLA GPSDAGKRPQ KCVIL
  PvCaM1-10  ADVDGDGRIN FDEFVKVMMA KRRSKRTEEK P-ACGKKKPA GPSDVGKRPQ KCVIL
   SbCaM1-6  ADADGDGDIN YAEFVKVMMA KRRSKRTEEK ATARGKKKAG APSDAGKRGQ KCVIL
     VvCaM7  ADVDGDGQIN YEEFVKVMMA KKRGR
   ZmCaM1-3  ADVDGDGQIN YEEFVKVMMA NRHHASAGRH
   PaCaM5-2  ADVDGDGQIN YEEFVKVMMA K
     AcCaM6  ADVDGDGQVN YEEFVRMMLA K
   VvCaM5-1  ADVDGDGQVN YEEFVRMMLA K
   StCaM2-1  ADIDGDGQVN YEEFVRMMLA K
     PtCaM2  ADVDGDGQVN YEEFVRMMLA K
     PtCaM3  ADVDGDGQVN YEEFVRMMLA K
     MgCaM7  ADVDGDGQVN YEEFVRMMLA K
   PaCaM5-1  ADVDGDGQVN YEEFVRMMLA K
   PpCaM5-4  ADVDGDGQVN YEEFVRMMMA K
   PpCaM5-2  ADVDGDGQIN YEEFVKMMMA K
     PpCaM7  ADVDGDGQIN YEEFVKMMMA K
     AtCaM2  ADVDGDGQIN YEEFVKVMMA K
   SmCaM5-2  ADVDGDGQIN YEEFVKMMMV K
   SmCaM5-3  ANVDGDGQIN YEDFVKMMMS K
 CsubCaM5-1  ADVDGDGQVN YEEFVKMMMA K
   MpCaM5-2  ADVDGDGQVN YDEFVKMMMA K
     OlCaM5  ADVDGDGEVN YEEFVKMMMA K
   FvCaM7-1  ADEDGDGQVN YDEFVRMMLN K
 PperCaM7-2  ADEDGDGQVN YEEFVRMMLN K
     MdCaM2  ADEDGDGQVN YEEFVRMMLN K
     MdCaM3  ADDDGDGQVN YEEFVRMMLN K
     PaCaM3  ADVDGDGQVN YEDFARMMLA K
```

```
     GrCaM3  ADTDGDGQVN YEEFVRMMMV AK
  CreinCaM6- ADVDGDGQVN YEEFVRMMTS GATDDKDKKG HK
   VcCaM6-2  ADCDGDGQVN YEEFVKMMTS SVEKPQEASV GKKP
    PaCaM1-1 ADTDGDGQVN YEEFVRMMMG A
   BrCaM5-11 ADVDGDGQIN YDEFVKVMMA K
      AtCaM8 ADLDGDGQVN YDEFVKMMIN ID
      CrCaM8 ADLDGDGQVN YDEFVKMMIN IG
      ThCaM8 ADLDGDGQVN YDEFVKMMIN IG
    MgCaM5-5 ADLDGDGQVN YDEFVKMMIN IG
    MgCaM8-2 ADLDGDGQVN YDEFVKMMIN IG
      CcCaM8 ADLDGDGQVN YDEFVKMMMT IG
      CsCaM8 ADLDGDGQVN YDEFVKMMMT IG
    PtCaM8-2 ADLDGDGQVN YDEFVKMMMN VG
    PtCaM8-1 ADLDGDGQVN YDEFVKMMMN VG
  CsatCaM8-3 ADLDGDGQVN FEEFVKMMMA VG
    GrCaM8-1 ADLDGDGQVN YDEFVKMMTT VG
    PperCaM8 ADLDGDGQVN YDEFVKMMMT IG
    VvCaM8-1 ADLDGDGQVN YDEFVKMMMT A
    GmCaM8-1 ADLDGDGQVN YDEFVKMMMT IG
    GmCaM8-2 ADLDGDGQVG YDEFVKMMMI IG
  PvulCaM8-1 ADLDGDGQVN YEEFVKMMMT IG
      MtCaM8 ADMDGDGQVD FDEFVKMMMT IR
      SlCaM1 ADLDGDGQVN FDEFVKMMMN V
    MgCaM8-3 ADLDGDGQVN FDEFVKMMTT VG
    GrCaM8-2 ADLDGDGQVN YDDFVKLMTT VG
    FvCaM8-2 ADLDGDGQVN YDEFVKMMMT IG
    SbCaM1-7 ADTDGDGQVN YDEFVLMMKN AERKISG
    ThCaM5-3 AGVDGDGQIK YDDFVKFMNA I
    FvCaM8-1 ADFDGDGLEP -----STTDS DLSASFTTIT FDRPLPLLR- ------GPVR AAPPDDPSSG PY-VLAFRDP KAWARAYSAC EVKIIHQCEG GSRIGCAISA ASKCKPPWWR FLIGGKAPD- ---LKERAEC EERETEGCVV AAKEKCVGFA RDKCV--GPF REARVCGVSK KQAEEMVGW-
    PvCaM1-9 ADVDGDGQIN YEEFVKVMMA KLQPNFCWVM ATFSLGVVGT SYHKPPGYLR FQEVVSICAA PWGVLMEQGK KAKVLCLHGF RTSGNFLKKQ ISKWHPSIFQ QFDMVFPDGI FPAGGKSEID GIFPPPYFEW FQFNKEFTEY TNLDECISYL CDYMVKNGPF EGLLGFSQGA TLSALLIGYQ
      SiCaM3 ADVDGDGQIN YTEFAKVMLA KRRNQELEDH GSDSSHSKSS CPSCTIL
      SbCaM3 ADADGDGQIN YNEFTKVMLA KRRNQELEDH GSESSHSKRL CPSCTIL
      ZmCaM3 ADADGDGQIN YTEFTKVMLA KRRNQELEDH GSESSHSKRL CPSCTIL
    SbCaM1-5 ADTDGDGLVS YDEFKQRMLR K
    AcCaM5-3 ADVDGDGQIN YEEFVKVMMA K
      AcCaM8 ADLDGDGQVN YEEFVRMMLA V
      CpCaM8 ADLDGDGQVN YEEFVRMMLA V
    TcCaM9-2 ADLDGDGQVN YEEFVRMMLA A
      CcCaM5 ADLDGDGQIN YEEFARMMLL PV
    MeCaM8-2 ADLDGDGQVN YEEFVRMMLA I
    MeCaM8-1 ADLDGDGQIN YDEFVRIMLA SS
    PtCaM8-3 ADLDGDGQVN YEEFVRIMLA A
    VvCaM5-2 ADLDGDGQVN YEEFVRMMLA V
      CcCaM4 ADLDGDGQIN YEEFSRMMLA AV
  PvulCaM8-3 ADLDGDGLVD YEEFVRMMLA A
    GmCaM8-3 ADLDGDGRVN YEEFMRMMTI
  PvulCaM8-2 ADLDGDGRLN YEEFVRMMTV
    SmCaM1-2 ADTDKDGKVS YPEFVKIVMP RQ
    SlCaM5-1 ADLDGDGQVN FDEFFKMMIK L
      StCaM8 ADLDGDGQVN FDEFVKMMIK L
    SmCaM1-1 ADIDGDGNVN YVEFVKIMTM CN
    CcCaM6-2 ADLDGDGLVS FEEFARMMMA F
    CsCaM5-1 ADLDGDGLVS FEEFARMMMA F
  CsatCaM8-1 ADLDGDGRVS YEEFARIMTI S
    MdCaM4-1 ADLDGDGLVS YEEFARMMML S
    MdCaM4-2 ADLDGDGLVS YEEFARMMML S
    MdCaM8-1 ADLDGDGLVS YEEFARMMLL C
    MdCaM8-2 ADLDGDGLVS YEEFARMMLL C
    MeCaM9-2 ADLNGDGLVS YEEFARMMMA F
    RcCaM9-1 ADLDGDGLVS YEEFARMMMA F
    MeCaM9-1 ADFDGDGLVS YEEFARIMMT A
    VvCaM8-2 ADMDGDGQVS YEEFAKMMMT FN
    GmCaM9-3 ADLDGDGQVS FEEFARIMML K
    GmCaM9-4 ADLDGDGQVS FEEFSRIMML K
  PvulCaM9-1 ADLDGDGQVS FDEFARIMML K
    MgCaM9-1 VDLDGDGLVS YDDFVKMMMA SYL
      SlCaM4 ADLDGDGLVS YEEFVKIMII
      MgCaM4 ADLDGDGQIS FDEFVKNMAT ASN
    MgCaM8-1 ADLDGDGQIS FDEFVKTMAT ATN
    LuCaM6-1 ADLDGDGLIS YHEFVQMMLA SFS
    LuCaM6-2 ADLDGDGLIN FEEFVKVMLA SL
    CsatCaM9 ADLDGDGLID YEEFVKMMLL I
  CsatCaM8-2 ADLNGDGHVD YHEFVKIMTQ DVSQILPESG FF
   CreinCaM1 ADVNEDKMVN YEEFVRMMTT R
      MpCaM1 ADKDEDGELN FEEFKSIWFN SVALNT
    MpCaM5-4 ADSSGDGEVD YDEFINFVLN D
      OlCaM1 ADTTGDGEVD YDEFIAFVLS SN
    GmCaM9-1 ADLDGDGRVS YEEFVRFMTL N
    GmCaM9-2 ADLDGDGRDS YEEFLRFMTL N
  PvulCaM9-2 ADLDGDGRVS YEEFARFMSL N
      MtCaM9 ADLDGDGRVS YEEFVKFMML N
      AtCaM9 ADLDGDGFLS FHEFSKMMIA ASY
      ThCaM9 ADLDGDGFLS FHEFSKMMIA ASY
      CrCaM9 ADLDGDGFLS LHEFSKMMIA ASY
   CreinCaM8 ADRNDDNEID EDEFIRIMKK TSLF
    CsubCaM4 ADRDGDGEVN EEEFFRIMKK TSLF
    PpCaM8-2 ADRDGDGEIN EEEFYRIMKK TSLF
    PpCaM8-1 ADRDGDNAVS EDEFYRIMKK TSLFN
      SlCaM8 ADRDRDGEVS AEEFMRMMRK TSFAN
    LuCaM9-2 ADFDGDGRVS YHDFTNMMTV C
    LuCaM9-4 ADFDGDGRVS YHDFTNMMTV C
    LuCaM9-3 ADFDGDGRVS YHDFTNKMTV C
    LuCaM9-1 ADFDGDGKVN YDEFARMMSS F
      CsCaM4 ADLDGDGQIN YEEFARMMLL PV
    MgCaM9-2 ADLDGDGLVS YDEFVRMMML MLASS
  CreinCaM6- ADPDKSGKVL YDNFIKMMLA R
    VcCaM6-1 ADPENTGKVI YDNFIRMMLA R
      EuCaM6 VEVGPDGRIR YEDFIARMVA K
    PaCaM6-1 IKVDSDGNIC YEDFIARMMA K
      GrCaM9 ADLDGDGVVS FDEFARMMMV AL
    SiCaM1-4 ADTDGDGRLS YEEFKQIMHR I
    TcCaM9-1 EKISLDPSTN QIPYVIGVQL RQVMMNLGER LTVEEAEQMI KEADLDGDGL VSYEEFARMI MAF
      ThCaM7 VDVDGDGRVN YKEFRQMMKG GMENTKER
      CrCaM6 VDADGDGNVN FEEFQKMMTS SSLINSNGSA NGGSA
    StCaM6-1 VDSDGDGYVS FEEFKKMMTT SK
    RcCaM9-2 VDKDGDGSVN FEEFRSMMTN TSRLANTMK
      FvCaM3 FDLDGNGVLD FHEFLKMMIS T
    MpCaM5-3 VDADGDGHLS YKEFLGFLQN ERQSSGGRLP GPPRPAFNPK G
    PaCaM5-3 VDADKDGRIS YEEFASMMKT GT-DWRKASR HYSRGRFNNL SIKLVRDGSL QMDNNERR
    PaCaM1-2 VDMDSDGRID YGDFVAMMSK GNQDNRKHDI TVGAPRCR
      CcCaM3 MDWDRNGKVS FREFLFALIS WIGIDNDEEI AEME
    PpCaM5-3 ATGSDQRKVN FEDFCQLMSS ALAELRYVAS QSATIRI
      VcCaM3 AEHEINGTLT YEEFKAVLS
      CpCaM6                                                                                 MGWWKEEHFN ASDAD-GDGL LNITEFNDFL HPADTKNPKL LQWLCKEEVR ERDSDGDGKV NFKEFFHGLF DLVRNYDE-- --DSDNSSH-
    PaCaM6-2                                                                      M PGNISAYGIE NGWWMEEHFN ASDED-GDGL LNRTEFNNFL HPADSNNSKL IEWLCKEEVR ERDTDKDGRL NFPEFYHGLY DLIRNYDD-- --NTFNESH-
   CreinCaM3                                           MKG SLARKSVDTP HAHAHSGKKR LGGGAKYSKA DVLYLKELFD KHDQD-HNGK VTIAELIEAL -----SAEKK LTHQELSAFR TIDINQDGVL TFNEYLRRLF PMATDKEFAC MLSWANPHT-
   CreinCaM5                                                                     MASA AARERYARKN EQNELERAFN TIDSK-GDKK IDAEELTELF L---RLGHKP KRGEVEDMIW EVDEDCDGCI SLLEF-QTLY TRCRD----- ----DKTGY-
      VcCaM5                                                                     MATA AARERYARKN EQNELERAFN TIDSK-GDKK IDAEELTELF L---RLGHKP KRGEVEDMIW EVDEDCDGCI SLQEF-QTLY TRCRD----- ----DKTGY-
  CsubCaM5-2                                                                          MIQERLSRKS EAADLRRAFT LLDTK-GDGK VDAVELGLVF Q---KLRHKE KKSEIEGMIW EVDEDCDQCL TWKEF-QDMY HRCRN----- ----DQTGY-
    MpCaM5-1                                                                      MAQ AARERFAKKN EAEDLLRAFR RLDTR-GDGK IDADELSSVF R---ILGHTA KKTEIEDMIW EVDDDCDMCV NWPEF-QTMY NRCRQ----- ----DKTGY-
   Consensus ADvDGDGq!n %eEFvk.Mma k......... ....

             541                                                                                                                                                672
    AcCaM5-2
      AtCaM7
    CrCaM7-2
      CpCaM7
    CcCaM6-3
    CcCaM6-1
    CsCaM7-2
    FvCaM7-2
    GrCaM5-1
    LuCaM6-3
    LuCaM7-2
    LuCaM7-3
    LuCaM7-1
    MdCaM6-1
      MdCaM7
    MeCaM5-1
    MeCaM5-4
    MeCaM5-3
      MeCaM7
    RcCaM7-1
    MeCaM5-2
    RcCaM7-2
    MgCaM5-1
    PperCaM5
    MgCaM5-2
    MgCaM5-3
    MgCaM5-4
    PtCaM6-1
    PtCaM6-2
  PvulCaM7-1
      CrCaM2
    MdCaM6-2
  PperCaM7-1
    PvCaM1-2
    SiCaM1-2
      AtCaM3
```

```
      AtCaM5
    ThCaM3-2
    BrCaM5-5
    BrCaM5-4
    BrCaM5-3
    BrCaM5-2
      CrCaM5
    ThCaM5-1
    BdCaM1-2
    BdCaM1-1
    ZmCaM1-1
    SbCaM1-4
    SbCaM1-2
    SbCaM1-3
    SbCaM1-1
    SiCaM1-1
    OsCaM1-2
    OsCaM1-3
    OsCaM1-1
    PhCaM1-3
    PhCaM1-4
    PhCaM1-5
    PvCaM1-4
    PvCaM1-5
    PvCaM1-6
      BdCaM3
    PvCaM1-3
    BrCaM5-6
    BrCaM5-7
    BrCaM5-8
      MgCaM6
    SlCaM2-2
    ThCaM5-2
    SlCaM2-1
      SlCaM3
    StCaM2-2
   BrCaM5-10
    SlCaM5-2
      StCaM3
      AtCaM6
    BdCaM1-3
    ZmCaM1-6
    PvulCaM5
    SiCaM1-3
    ZmCaM1-7
    PhCaM1-2
    PvCaM1-7
    PvCaM1-8
      MtCaM7
  PvulCaM7-3
  PvulCaM7-2
    GrCaM5-2
    ZmCaM1-8
      OsCaM2
      OsCaM3
      GmCaM5
      AtCaM1
      AtCaM4
      ThCaM1
      ThCaM4
    CrCaM1-2
    CrCaM1-1
    BrCaM1-1
    BrCaM1-2
      MtCaM1
    SmCaM5-1
      PaCaM7
    AcCaM5-1
      TcCaM7
    CcCaM6-4
    BrCaM5-1
    ThCaM3-1
      CrCaM3
      PtCaM7
    BrCaM5-9
    ZmCaM1-4
    PpCaM5-1
    ZmCaM1-2
      PhCaM3
   PvCaM1-10
    SbCaM1-6
      VvCaM7
    ZmCaM1-3
    PaCaM5-2
      AcCaM6
    VvCaM5-1
    StCaM2-1
      PtCaM2
      PtCaM3
      MgCaM7
    PaCaM5-1
    PpCaM5-4
    PpCaM5-2
      PpCaM7
      AtCaM2
    SmCaM5-2
    SmCaM5-3
  CsubCaM5-1
    MpCaM5-2
      OlCaM5
    FvCaM7-1
  PperCaM7-2
      MdCaM2
      MdCaM3
      PaCaM3
      GrCaM3
  CreinCaM6-
    VcCaM6-2
    PaCaM1-1
   BrCaM5-11
      AtCaM8
      CrCaM8
      ThCaM8
    MgCaM5-5
    MgCaM8-2
      CcCaM8
      CsCaM8
    PtCaM8-2
    PtCaM8-1
  CsatCaM8-3
    GrCaM8-1
    PperCaM8
    VvCaM8-1
    GmCaM8-1
    GmCaM8-2
  PvulCaM8-1
      MtCaM8
      SlCaM1
    MgCaM8-3
    GrCaM8-2
    FvCaM8-2
    SbCaM1-7
    ThCaM5-3
    FvCaM8-1  -----VSVMG KSRWVGLIGL EK------CG FGATNCRAGE LVGYSGEVDR VLGGGTETV
    PvCaM1-9  SQGKVLSDHP PIKFMVSISG SKFRDPSICN IAYKDPIKVK SVHFIGEKDW LKIPSEELAS AFVDPLIIRH PQGHTVPRLD DASVKLLSEW SSNILEDLKN EDVNVTEALD SDESADVELA ENNMMEQVAA
      SiCaM3
      SbCaM3
      ZmCaM3
    SbCaM1-5
    AcCaM5-3
      AcCaM8
      CpCaM8
    TcCaM9-2
      CcCaM5
    MeCaM8-2
    MeCaM8-1
    PtCaM8-3
    VvCaM5-2
      CcCaM4
  PvulCaM8-3
    GmCaM8-3
  PvulCaM8-2
    SmCaM1-2
    SlCaM5-1
      StCaM8
    SmCaM1-1
    CcCaM6-2
    CsCaM5-1
  CsatCaM8-1
```

```
    MdCaM4-1
    MdCaM4-2
    MdCaM8-1
    MdCaM8-2
    MeCaM9-2
    RcCaM9-1
    MeCaM9-1
    VvCaM8-2
    GmCaM9-3
    GmCaM9-4
  PvulCaM9-1
    MgCaM9-1
      SlCaM4
      MgCaM4
    MgCaM8-1
    LuCaM6-1
    LuCaM6-2
     CsatCaM9
  CsatCaM8-2
   CreinCaM1
      MpCaM1
    MpCaM5-4
      OlCaM1
    GmCaM9-1
    GmCaM9-2
  PvulCaM9-2
      MtCaM9
      AtCaM9
      ThCaM9
      CrCaM9
   CreinCaM8
    CsubCaM4
    PpCaM8-2
    PpCaM8-1
      SlCaM8
    LuCaM9-2
    LuCaM9-4
    LuCaM9-3
    LuCaM9-1
      CsCaM4
    MgCaM9-2
  CreinCaM6-
    VcCaM6-1
      EuCaM6
    PaCaM6-1
      GrCaM9
    SiCaM1-4
    TcCaM9-1
      ThCaM7
      CrCaM6
    StCaM6-1
    RcCaM9-2
      FvCaM3
    MpCaM5-3
    PaCaM5-3
    PaCaM1-2
      CcCaM3
    PpCaM5-3
      VcCaM3
      CpCaM6  ---------P SG---DLMET PA------KN MFSRLDKDGD GYLSDIELLP IIDKLHPSER YYAKQQA--- --DYIISQAD TDKDRRLT-- ---LIEMIEN PYVFYSAIFN DDDADDYEYH DEFR
    PaCaM6-2  ---------V SQGYESMREA SA------KK LFTELDKNHD GFLMENELQP VINRLHPGEE YYAKQQA--- --DYMIMQAD ADKDGRLS-- ---LKEMLDH PYVFYSAIFT DEEDED-DYH DEFR
   CreinCaM3  ---------A AGAHETLFEP TKEQLEEIKK MFAMFDKNGN GTIEKVELVA VAEKCG---- -YVGSDV--- --EDLFKAHD VDHNGSIS-- ---FDEFVQL MKISYV
   CreinCaM5  ---------E PRGLFNVVE- ---------- -FVMNDKNNQ GFITLEEAMQ IMYLRYG--R AELDMQL--- --EQVFGTAD LNSGKILT-- ---LTEFLHC LHTNQVKQLL NRVTAKTYKA PPPPQKRK
      VcCaM5  ---------E PRGLFNVVE- ---------- -FVMNDKNNQ GFITLEEAMQ IMYLRYG--R AELDMQL--- --EQVFGTAD LNSGKILT-- ---LTEFLHC LHTNQVKQLL NRVTAKTYKA PPPPQKRK
  CsubCaM5-2  ---------E PHRLFNVVQ- ---------- -FLMNDKEGK GTVSLEETMQ IMYLRYG--R QLLDAQL--- --EEIFGTSD LNSGKTLT-- ---LTQFLHS LHVSQIRQLN SRVTAKTYQP PAVILKKQVS SV
    MpCaM5-1  ---------E PRRLFNVVE- ---------- -FLMNDKDDS GTVSIEEAMQ ILYLRFG--K GLLDSHL--- --EDMFGTSD ANSTSDLT-- ---LTEFLNS LHLSQAKHL- -RSTAKSHQQ KR
   Consensus
```
