# Supplementary material for: Genomics and evolutionary aspect of calcium signaling event in calmodulin and calmodulin-like proteins in plants
Source: BMC Plant Biol. 2017 Feb 3;17:38. doi: 10.1186/s12870-017-0989-3 (PMC5291997; doi:10.1186/s12870-017-0989-3)
Supplement: Additional file 4: Figure S2. — Multiple sequence alignment of CML protein of plant. Alignment shows presence of conserved motifs in EF-hand domain. (PDF 441 kb) [file 12870_2017_989_MOESM4_ESM.pdf]

```
 Name: PperCML11-3    Len:   727  Check: 2221  Weight:  1.04
 Name: BdCML5         Len:   727  Check: 2061  Weight:  1.06
 Name: OsCML6         Len:   727  Check: 8671  Weight:  1.06
 Name: MdCML11        Len:   727  Check: 5125  Weight:  1.10
 Name: AtCML13        Len:   727  Check: 8993  Weight:  1.05
 Name: AtCML14        Len:   727  Check: 9664  Weight:  1.05
 Name: BrCML34-2      Len:   727  Check: 6938  Weight:  1.07
 Name: BrCML34-1      Len:   727  Check: 4056  Weight:  1.07
 Name: CrCML34        Len:   727  Check: 5771  Weight:  1.06
 Name: AtCML34        Len:   727  Check: 7873  Weight:  1.06
 Name: ThCML34        Len:   727  Check: 4146  Weight:  1.06
 Name: BrCML32-2      Len:   727  Check: 2471  Weight:  1.06
 Name: CrCML32        Len:   727  Check: 4594  Weight:  1.03
 Name: CrCML32-2      Len:   727  Check: 3471  Weight:  1.03
 Name: CrCML32-1      Len:   727  Check:  517  Weight:  1.04
 Name: AtCML32        Len:   727  Check:  216  Weight:  1.04
 Name: AtCML31        Len:   727  Check: 4281  Weight:  1.04
 Name: ThCML32        Len:   727  Check: 9693  Weight:  1.05
 Name: ThCML33        Len:   727  Check: 4375  Weight:  1.06
 Name: AtCML33        Len:   727  Check: 9063  Weight:  1.06
 Name: EgCML25-2      Len:   727  Check: 4328  Weight:  1.12
 Name: SmCML25        Len:   727  Check: 3096  Weight:  1.12
 Name: LuCML25-1      Len:   727  Check: 8912  Weight:  1.12
 Name: AcCML25-3      Len:   727  Check: 1518  Weight:  1.15
 Name: BdCML2         Len:   727  Check: 2099  Weight:  1.09
 Name: CrCML12        Len:   727  Check: 7445  Weight:  1.06
 Name: AtCML12        Len:   727  Check: 4723  Weight:  1.06
 Name: CsubCML24      Len:   727  Check: 7487  Weight:  1.10
 Name: PpCML7         Len:   727  Check: 7974  Weight:  1.14
 Name: MpCML21-1      Len:   727  Check: 1380  Weight:  1.12
 Name: MpCML4         Len:   727  Check: 3569  Weight:  1.12
 Name: MpCML30        Len:   727  Check: 2177  Weight:  1.13
 Name: MpCML15        Len:   727  Check: 4068  Weight:  1.13
 Name: MtCML46        Len:   727  Check: 3855  Weight:  1.07
 Name: AtCML45        Len:   727  Check: 1602  Weight:  1.06
 Name: AtCML46        Len:   727  Check: 3670  Weight:  1.06
 Name: AtCML47        Len:   727  Check: 5529  Weight:  1.11
 Name: VcCML4         Len:   727  Check: 8787  Weight:  1.12
 Name: OsCML1-3       Len:   727  Check: 3255  Weight:  1.12
 Name: AtCML44        Len:   727  Check: 1498  Weight:  1.14
 Name: OlCML11        Len:   727  Check: 7512  Weight:  1.17
 Name: AtCML1         Len:   727  Check: 2677  Weight:  1.17
 Name: CsubCML20      Len:   727  Check: 3959  Weight:  1.11
 Name: SiCML36        Len:   727  Check: 7987  Weight:  1.05
 Name: SbCML50        Len:   727  Check: 6591  Weight:  1.05
 Name: MtCML11        Len:   727  Check: 3020  Weight:  1.16
 Name: Consensus      Len:   727  Check: 3094  Weight:  0.00

//

               1                                                                                                                                            180
   AcCML3-1                                                                                                MVIAITLLAI LFLVGFINTL F-YIPPNKFL AHLQSFLPYK P------SSI MTNNKSSIST VKEEGKKKNN TKSVHEVM-- --------E-
     CcCML7                                                                                                MVISILLLAV LFIAGFINIF F-YFPTKKFY AWIQSFFSKK A--------- ----TSTSES RVSSPSKETS AYK------- -KA-----E-
     CsCML7                                                                                                MVVSILLLAV LFIAGFINIF V-YFPTKKFY AWIQSFFSKT A--------- ----TTTGES R-------TS AYK------- -KA-----E-
   GrCML5-1                                                                                                MVTTLLLLAI LFILGFIHIY F-SFLPNKFY YWIQSLIRNT S------PSS SSSSAVNYKT AQKISVTERG DPN------- -NL-----E-
   GrCML5-3                                                                LCRPQPN PPFNSNNPSF LLLFYVHLLL LGCISVT-RN MVTSLLLLAL LFIAGFLNVY F-YISRKKFH AWLQSFSPKT C--------S SA-GSVVSSG PKAATEEKRD SNK------- -VV-----E-
   TcCML5-1                                                                                                MVTSILLLAA LFIAGVVNVY F-YFSPKMFY AWLLSFFRNS S--------S SAVSSKTAAA PQVISVKERS DSE------- -KV-----E-
   MeCML3-1                                                                                          MV-LS SPAAMLLLLL LFLAGLLNVY F-SVPSKNLY SWIQSFLFKN ---------P SAKVPPLIK- --STTASCNS TAM------- --G-----E-
     MeCML5                                                                                          MV-LS PAMQLFLLLL LFLAGLLNFY F-YVPSKKLC SWFQSFLFKN N------PAA SSKVSPQIER TCNTSSSCNA KNM------- --G-----E-
   PtCML3-3                                                                                             MM MIITILLLVV LFLAGLINIY F-YLPSKKLY AWLQSFA--S K------NPP TAITPQVSHK ERSITTSSLS ISSSSGSPAY DEA-----E-
   PtCML3-4                                                                                             MM MIITILLLIV LFLAGLINIY F-YLPSRKLY AWLQSLA--S K------TPP RTITLQVSNK GRSVTTTSVS SSSVSG-PAY EKA-----E-
     RcCML3                                                                                                MVIAILLLLL LFLAGFLNVY F-YLPPKKLY AWLRSFSFKN K------NIS SSTPIKVVPT AALDSEKDKA KASSENYDSN KKD-----E-
     LuCML5                                                                                                MITAALLLLL LFIAGVFNLS S-YFPSKKLY ARLHSLFLAA T------MKA ETEEIKSAPV KQVI------ ---------- -KNSAAEDE-
   LuCML3-3                                                                                                MISAALLLLL LFLAGLFNLN S-YLPSKKLY AWLHSLFLPR A------VTA ALEKVKSAPL QAESSKSVTT VSKSDQISNK KKTEDTDDE-
   LuCML3-2                                                                                                MISAALLLLL LFLAGLLNLN S-YLPSKKLY AWLHSLFLPR A------VIT AAEKVKSAPL QAESSKAVTT VAKSDQIS-K KKTEDTDDE-
   GmCML5-1                                                                                                MDLSTLLLAV LFIAGLINIF F-YVPTTKIG AWLQTFLFPN N------NAC NKTKTNLVPS SS-SSPTTKV EST------- -GSQKKKEE-
   GmCML5-2                                                                                                MDLATLLLTV LFIAGLINVF F-YIPTTKIS AWLQTF-FPN N------NSC NKTKTNLAPS PSPSSPTTKM AES------- -GSQKKKEE-
 PvulCML3-1                                                                                                MDLATLLLAV VFIAGLINIF F-HVPNNKIT TCLQNF-LPN N--------V FKTKTTLAPP -----PTTK- EEH------- -GSSRKKKE-
     MtCML7                                                                                                MVLATLLLAV LFIAGLINII F-YIPTNKIR AFLQII-FPT N------NS- NVSKTNLV-- ------TTTK LEK------- -DANYGKEE-
   CpCML3-1                                                                                              M VITSVLLLSL LFALGFFYIF F-NFPST--N KLFIRLRSNL F------SSH -ESPSVSIPI AAGSSSARLQ SGS------- -----RRGE-
   CsatCML5                                                                                             ME PIFNFLLLSV LFVAGFINFL L-YFPSKRFS AWFQSIKPSS Q------ITH FKSTPLQPPP PPSPSPSPSP PPP------- -----SAME-
     SlCML5                                                                                              M LTISLLFLAF LFILGLITTL F-NFPTKKFQ SWIFSLSVKP Q------TLT PTTPFVNKDS IFVEKKSSKV ---------- --------E-
     StCML5                                                                                              M LTISLLFLAF LFILGLITTL F-NFPTKKFQ SWIFSLSVKP Q------TLT PTTPFVNKDS ILVEKKSSKL ---------- --------E-
     FvCML5                                                                                              M VFSTLLLLAL LFFAAF---F I-YFPNRKLL AWFQSF-LPI Q------TVS KSTATKKEAV VRSVMTTKRE QED------- -----P--E-
   MdCML3-1                                                                                              M VLTTVLLLAV LFIAGLVNIF X-LFPTPKLL AWFQSLSIPY S------TPT TTPSKDKNNL VMNSINPQPK VED------- -----RAVE-
   MdCML3-4                                                                                              M VLTTVLLLAV LFIAGLVNIF X-LFPTPKLL AWFQSLSIPY S------TPT TTPSKDKNNL VMNSINPQPK VED------- -----RAVE-
 PperCML5-2                                                                                              M VLITILLLAV LFIAGLINIV F-YLPTNKFL AWFQSLFQAN S------STA ATPVAPPTK- -SKDIISSNR AED------- -----A--E-
   EgCML5-1                                                                                                MLLTALLLSL IFIATLGNIL F-HVPLKKLP VLVKSLFAV- ---------- ----VTAREQ ERLQKRKEQP TMS------- -AKR--REE-
   EgCML5-4                                                                                              M VLLAALFLSL VLLVSLANVL LLRVPPKKLP ALLRTLFAQP P------PPA APGKVDGGPP PPPPPQKQQQ KKK------- -ERRGVSAE-
     MgCML4                                                                                                 MITILLSSL LFIFSLIFIK F--SNTKKYL KNIKTPPQDS P------TQS NPTSTKPKPK PAA------- ---------- --------D-
  LuCML30-3                                                                                                MITAALLLLL LFIAGVFNIS S-YFPSKKFY ARLHSLFLAA T------TKA ETDEIKSAPM KQEIRNSVAE D--------- --------E-
   StCML3-1                                                                                                                                                             MADQKKVKS FNN------- ----YNKVE-
    BdCML30                                                                                                    MAPLLL LFLLGGLCAF FSLASAASRA ASKK------ ---------- CG-AGGRNCE QGKSLVEEEE EER------A KAPERDREDA
    OsCML30                                                                                                    MAPLLL LFLLGGLCAL FSLAS--SSP ATKK------ ---------- CGDAKKRREE EGEEVVVVAK KRP------E EEPRRPDPDA
    PhCML30                                                                                                    MAPLLL LFLLGGLCAL FSLTTSSRAA AAAKNCGDVK S------GAK SGDAGK---- -DGSEGEEAA RRE-----GA RKQARPDPEA
    SbCML30                                                                                                    MAPLLL LFLLGSICAL FSLTTSSRAS G--KKSD--- ---------- ----GKEPRC RDGCVPEETT QQQQEEEEGK KKQARADPEK
    ZmCML30                                                                                                    MAPLLL LFLLGGLCAL LSLATSSRAS A--KKCDDVK S------NGN CGDSGEEARC RDDCAPEETQ QQG------- KMQARPDPEK
    SiCML30                                                                                                    MAPLLL LFILGGLCAL FSLTTSSRST AAKKCVGDVK ---------- CGDAGKGSA- -SEGEEEEAA RRR------- KQARGPDPET
   AcCML3-2                                                                                              MPTILLRI SLLYNLVYSI FLYFLPKKFR SFLP--SSSW LFFNSNVVVE EEEPITSSTT TTTSLPPSSS STPLLLIQDK KMDQQTMDPV
     CpCML5                                                                                              MPTILLRI FLVYNLLNSI LLYLIPKKLR GFLP--PSWY PHPHPHHHHH QQQQQ-PNLV LDSSSKSPS- ----PSPSSV SGL-KRMDSA
   MgCML3-2                                                                                              MPTILLRI FLLYKLLNTI FLYLVPKKLR TFLP--PSWY PYLHQQEQQK QQKHNNTNTI NEPASPSSS- ----PVISPL HKFPRRMDAD
   PtCML3-1                                                                                              MPTILLRI FILYNLLNSF LLSLVPKKLR FLLP--TSWY ---HPHQANT NTSWCHHHQA NTNTKKPSS- ----LLPSPS -FVLTRMDQA
   PtCML3-2                                                                                              MPTILLRI FLLYNLLNSF LLSLVPKKLR FLLP--TSWY ---HPHQANT NTSWCHPHQA NTNTKKPSS- ----LLPSPS -FVLARMDQA
     PtCML5            MSSFHQVVFT RRLIVSFKAV QVQITRPNSI NWAKPASLLN VARCHHPHQS RVNSRLFITV SNGSKTKAKH LPPNIFSTLL LSMRTILLRI FLLYNLLNSF LLSLVPKKLR FLLP--TSWY ---H------ -----HPHQA ITNTKKPSS- ----LLPSSS NFVLKRMDQA
 PperCML5-1                                                                                              MPTIFPRI FLIYNLLNTF LLSLVPKNLR PLLP--SSWF P-CQTNLVAT NTSLPHFPPS SSSSSLPLP- ----LPLPCG APKVIRMDPN
   StCML3-3                                                                                            MQFPAIFFKT RFIYNLFNPI LLSLLPKKLI SFLP--PSWF ---------- HQKHLHSRSP APPQQSPVS- ----VSDAVQ SHIQKRMDSD
   GrCML3-2                                                                                              MPTFLFRI FLLYNLL--- LDYLVPRKLK SFLS--PSCT T-TTPFVSVG GETEKNPS-- --PAVALAS- -------VSP RCPLKRMDAA
   TcCML5-2                                                                                              MPTVLLRI FLLYNLV--- LDYLVPKKLK TFLP--SSWI P-TRTLVSTG SESKTHTSTS TAPESASAP- -------ASS ACCPQRMDGA
     CcCML3                                                                                              MRFILLRI FLLY----TF ILHLLPKKLR RFLP--RSWF P--------- ------APAL GPSLSSQSN- -----TNPT- ---RSTMDQA
   CsCML3-1                                                                                              MRSILLRI FLLY----TF ILHLLPKKLR RFLP--RSWF P--------- ------APAL GPSLSSQSN- -----TNPT- ---RSTMDQA
   EgCML5-3                                                                                              MPAIITRI FLLYHLLHTW FHYLVPKKLR VYLP--PSWS P--------- ------LRLD PTPPPLPRS- -----LSLV- ---KAPMDAA
   GmCML5-3                                                                                              MPTILHRI FLLYNLVHSF LLCLVPKKVR PFLP--PSWF Q--------- ------TKTI TAPSSSSSS- -----SSSAR IIKRTTMDPN
 PvulCML3-2                                                                                              MPTILHRF FLLYNLLHPF LLFLVPKKVR AILS--PSWF R--------- ------STTT PPPPSSSSS- -----RLITT I--SPPMDPH
     MtCML5                                                                                              MPTILLRI FLLYNVVNSF LISLVPKKLI TFFP--HSWF T--------- ------HQTL TTPSSTSKR- -----GLVFT --KTITMDPN
     FvCML7                                                                                              MPTIFFRV FLIYNLVHAF LLSLLPKSLR RPLQ--NQ-- ---------- ------AKVD KRRSSVGAA- ---------- --NKVEMQPT
   BrCML4-1                                                                                                  MVRV FLLYNLINSF LLCLVPKKLR VLFP--PSWY ---------- --------ID D--NIPPPL- S------EPE PKSQTRTDPV
     CrCML4                                                                       YQK L-FPSLFSTK P---TVMVRV FLLYSLFNSF LLSLVPKKLR VLFP--PSWY ---------- --------ID D--KNPPPVP S------QSE TESPGRTDPV
   BrCML4-2                                                                                                  MVRV FLLYNLLNSF LLCLVPKKLR VLFP--PSWY ---------- --------TD D--KITPP-- --------SE SECSLRTDPV
     ThCML4                                                                                                  MVRV FLLYNLFNSI LLCLVPKKLR VLFP--HSWI ---------- --------ID D--KNPPP-- --------SK SESPARTDPV
     AtCML4                                                                                                  MVRV FLLYNLFNSF LLCLVPKKLR VFFP--PSWY ---------- --------ID D--KNPPP-- ---------- PDESETESPV
   CrCML5-1                                                                                                  MVRI FLLYNILNSF LLSLVPKKLR SLFP--LSWF ---------- --------DK TLHMNSPPSP PTMLPSPSSS PLPTKKIDPS
     AtCML5                                                                                                  MVRI FLLYNILNSF LLSLVPKKLR TLFP--LSWF ---------- --------DK TLHKNSPPSP STMLPSPSSS SAPTKRIDPS
   ThCML5-1                                         MVV EQVGPRGRSK PTEWRQPS-- --GDQQFPSF SLSPNLCFQA LCFHQISENK SFDSAVMVRI FLLYNLLNSF LLSLVPKKLR SLFP--LSWF ---------- --------DK TLHKTSPSS- --MLPSPSPS SAPTKRTDPS
   BrCML5-1                                                                                                                                                                         MLPSPSPS SAPSIKTDPT
   BrCML5-2                                                                                                                                                                         MLPSPSPS SAPSIKTDPT
   BrCML3-1                                                                                                                                                                                        MDQA
   BrCML3-2                                                                                                                                                                                        MDQA
     CrCML3                                                                                                                                                                                        MDQA
     ThCML3                                                                                                                                                                                        MDQA
     AtCML3                                                                                                                                                                                        MDQA
   CpCML3-2                                                                                                                                                                                        MDPG
   PtCML3-5                                                                                                                                                                                        MDPA
     EgCML3                                                                                                                                                                                        MDPA
   GrCML3-1                                                                                                                                                                                        MDPT
   CsatCML3                                                                                                                                                                                        MDPT
   LuCML3-1                                                                                                                                                                                        MDAN
     FvCML3                                                                                                                                                                                        MDRA
   MdCML3-2                                                                                                                                                                                        MDRA
   CsCML3-2                                                                                                                                                                                       IIMDT
   SlCML3-1                                                                                                                                                                                        METD
   StCML3-2                                                                                                                                                                                        METD
   MeCML3-2                                                                                                                                                                                        MDSM
     SlCML7                                                                                                                                                                                        MDPV
     StCML7                                                                                                                                                                                        MDPV
   SlCML3-2                                                                                                                                                                                        MDPK
   GmCML3-1                                                                                                                                                                                        MDPM
   GmCML3-2                                                                                                                                                                                        MDPA
   GmCML3-3                                                                                                                                                                 MMR QEVVFWHPRG FKKRISMEAQ
 PvulCML3-3                                                                                                                                                                                        MEAV
   BrCML5-3                                                                                                                                                                                        MNTT
   CrCML5-2                                                                                                                                                                                        MDST
     AtCML6                                                                                                                                                                                        MDST
   ThCML5-2                                                                                                                                                                                        MDST
     CrCML7                                                                                                                                                                                        MDPT
     AtCML7                                                                                                                                                                                        MDPT
   GrCML5-2                                                                                                                                                                                        MDDI
     GrCML7                                                                                                                                                                                    IFVGMDET
   BrCML2-1                                                                                                                                                                                        MDSG
   BrCML2-2                                                                                                                                                                                        MDRA
     CrCML2                                                                                                                                                                                        MDRG
     ThCML2                                                                                                                                                                                        MDRG
     AtCML2                                                                                                                                                                                        MDRG
   GrCML3-3                                                                                                                                                                                        MDLV
   GmCML3-4                                                                                                                                                                                        MDQG
   GmCML3-5                                                                                                                                                                                        MDQG
     MtCML3                                                                                                                                                                                        MDQG
 PvulCML3-4                                                                                                                                                                                        MDQA
   MeCML3-3                                                                                                                                                                                        MDAA
```

```
BdCML28     MDES
PhCML28     MDSS
SiCML28     MDSS
PvCML28-1   MDSS
PvCML28-2   MDSS
SbCML28     MDSS
ZmCML28     MNSIESS
OsCML28     MDST
MgCML3      MMKEMGTQ
MgCML7      MDPA
BdCML22     MR CQAEAAPTAT IPSSMPPSAP LVKPPR--SG LLSYIPTSIS SILP-ARGCV D--------A SSPPPSAPPA PPSPSKMSPP SGTTTTTKGS ------AEAA
PhCML17     MH CRAHTVP--- ---AMPPSSP LVKLPLLPRG LLSYIPT--- SILP-S-GRE SA---ATPTT CSSPSKPQPA PPCPKKMSSS SVQQQTGSGS GK---QADQA
SiCML22     MH CRAHTVS--- ---AMPPSSP LVKLPLLPRG LLSYLPA--- SILP-SSGRE ST---ITPTT SSPSTPPQPA PPSPKKMSSS PGQQQQAGSG GG---KADSA
SbCML22     MLSSSSP LVKLPLLPRG LLSYLPT--- SILP-S-GRE ST---ATPAA ANAPSTP--- PSSPKKMSSS SAQQQQQAGN GGSSKKADSA
OsCML22     MS CQVPTVP--- ---A----SP VVKFPLLPRG LLSYLPANLS SILPVARGAA STCEASSTTT TTMPPEPPAS PPPPKKMSP- PGAGAGAGSK KKQQQQADAA
ZmCML17     MLSSSH LVKLPLLPRG LLSYLPA--- SILP--SGRE ST-------- ---ADAAPST PPSSKKMSSS AQQQQQQQAG SS---KAESA
OsCML17     MACDQQA
SmCML5
SmCML7      MAAPIDQA
CcCML42     ME VAAAAGPGAT PTEKRRWSRR PSSSSS---F RLRCPSLNTL
MgCML42     METAGGSD EMVKRSSISL GGASNS---F RLRSPSLNAV
AtCML42     M ESNNNEKKKV ARQSSS---F RLRSPSLNAL
AtCML43     MEINNEKKKL SRQSSS---F RLRSPSLNAL
OsCML32     MDAKQS VAAAKPSLAK KTASAS---F RLRNGSLNAV
PaCML42-1   MFHGSRNL VCKHSEASSS PSGGLCSDLL AANSHS---- -KLDNPSEPV
PaCML42-2   MSRGSRNF ICKNG--GSS PSGGLCSDLY GLNDHSDIDH SKLQDPSEAV
PaCML5-2    MGFCLDTD
PaCML5-1    MALCIDTH
SmCML4
SmCML3      EER
AcCML25-1   MGLRSFLARR K-----K--- -----SKSNG GENPFRSLSL SSRNIMNTSP QT--ETQIE-
PvulCML25-  MGLRSLFGKK K-----KLPK TTSTLSNTSS SSSPPSSLPS SSLSLSFMHN HS--RAPVTV
BrCML25     MFNSK K-----ESNG TASRT----- ------GADS PFLQKARS-- ----GKTEVR
CrCML25     MFNKG A-----GS-- -SSTNGG--- -----IGADS PYLQKARS-- ----GKTEIR
AtCML25     MFNKN Q-----GSNG GSSSNVG--- -----IGADS PYLQKARS-- ----GKTEIR
BrCML25-1   MSN NKNQTNGSGG GSASRTGADS PFLHKARS-- ----GKTEIR
ThCML25     MGFGTMFNKK K-----ESDG SSSSLPGGGG GTVPRIGADS PFLQKARS-- ----GKTEIR
CpCML25-1   MGFGTLFNRK K-----SSNQ SSTSAPASPP LATTSFVPGS PFLTKNHS-- ----GKTEIR
EgCML25-1   MGLRSLFRRK --------KP --TSPSPPPG ASGAATPLGS RSSSMGR--- -----RSQIE
MeCML25-1   MGFRALFHRK K------SKK --SANSPGAS PAVAAPLDGS RSQSFKY--- -----KPQVE
MeCML25-2   MGFKELFHLK K------SKK --SDDSSVDS STVAPPFTGS RSQSFKL--- -----NTQLE
PtCML25-1   MGFKALFNRK K-----KSKK --SDSSVDSP SAIVASISGS KSPSIRL--- -----RPQVE
PtCML25-2   MGFKCLFNRK -------SKK --SDSSIDSP SGMAASINGS KSPSVRL--- -----RLQVE
GrCML25-3   MGFGSIFNRK K-----KLRS ------RNLT DSPTVSSSGS LFLQSPV--- -RNFTQTQLQ
GrCML25-5   MGFGSIFSRK K-----KHHS ------PNST ASPAVPPSGL AFLQSPMPTP SRNFAQIQPQ
TcCML25-3   MGFGSIFNRK K-----KRHS --LSNASNLT GSPAVGANGS SFLQSPMRTP TRNLTQTQLQ
MdCML25-3   MAY LRESDMGRRR LLSGXRVASV ASVACNESLK THKSESRIL- -----VLPIG FGFRLXSDPD G-----FQIP LLPEEEIQSL LRRRRRRRTP PRLRPTHSRS PSFHSRAQIA
MdCML25-4   MGFKSLFSRK K-----KSSP DVAAAHPDVA ATHPAASAAH PAAASAHSRT PSFHSRAQIA
PperCML25-  MGFKSLFSRK K-----KSIP ---------- ------SATA LPLTTTPSRS PSFSSRAQIA
CsCML25-1   MS PNGSQHSPHP LITATSAACN SNRSNSSSNN NNNVIIPDVH
CsatCML25-  MGFKSLFSRK K-----KS-- HSS------T DSPLGSTTLP ILGSRTQ--- -------IAE
MgCML25-1   MGFKSLFKRK K-----KSLI NSS------Q STPAVSRSPS QNGGRCR--- -------IEE
MtCML25-4   MGFRSLFHRK K-----KLQN TTSSSPTEVT TNTTFLSRTT SLQSRVQ--- -------FTE
SlCML25-1   MGLKSLFKIK K-----KDVP SGNPFVVNDN PMSVNLSRVS SIDSRAR--- -------IEE
StCML25-1   MGLKSLFKIK K-----KGVP SANPFVVNNN PMPVNLSRVS SIDSRAR--- -------IEE
MtCML25-3   MGLKSMFNKP S------S-- ---------S SDPPSRSTSL SVRSRTR--- -------LAG
PvulCML25   MGLKSLFNRE E-----GT-- ---------T TGGASRSTSL SVRSRVR--- -------MAG
GrCML25-4   MGFGSILSRE K-----KQNP ---------- -------SNP VASSSPM--- -----RTQLE
SlCML25-2   MG L-IKSLFNRK K-----KNDP ---------- -----TPANS PPVNTPV--- ------MIEE
GrCML25-1   MGLGWILKRK K-----KQNS ---------T SNTPTSTASP STRSSSQ--- ------TQLQ
LuCML25-2   MNCHAS
LuCML25-3   MNCHAS
CsatCML25-  SQIT
PaCML25-9   MG KNLLSRSSRK K-----SKGG GEDNSSSSSS RSEPSEPSSV SESLAGA--- -----LHNRE
BdCML10     MGKIK M--PSLFRRR S-----R--- ----SKSPPS QRQLEEEGLG QDQQQQCGDG SSSPARTPEE
PvCML10-1   MGKMR MPVPSLFRLG R-----R--- ----SKSPPP PPQQ-----Q AQ-----AAG SPPR--TAEE
SbCML10-1   MGKIR MPVPSLFRRR S-----K--- ----SPPPQQ QPAA-----E GK-----GTG SPPRAATAEE
ZmCML10-1   MGKIR MPVPFLFRRR S-----S--- ----KPPPPQ PQQQAPAAGD GD-----GSG SPERA-TAEE
OsCML10     MVK IKMPALFRRR S-----G--- ----SKSPPL PQAD-----P AS-----GGG SPAP--TPEE
PvCML10-2   QPAAQA AGSPPRTAEE
BdCML15     MGKMR SLFSR----- ------S--- ---GSSRRSS SSSKSPPSST RGAESPARAA TAP-PPRPED
OsCML15     MGKVR AFFSRKGRGN S-----S--- ---GRSRSMR EAAMNVDWSP RPSDLAAAAA AKPRPPAAED
PhCML10     MGKVR SFFSRSGKRG A-----G--- -----SSSP- ASSAAGSAPS SPSPS----- SPAPAA-EKD
PvCML15     MGKVR SFFSRSGKRC A-----G--- -----SSSP- SSSAAGSAPS SPSPRKSSAL APAPAAAEKD
SiCML10     FPSI IGEQQLRSYE CFLASRHRNK QGINRHQRHL SSSFPARATP PGTSAMGKVR SFFSRSGKRA G-----GGGS FRAGTSSSPR SSSAAASAPP SSSPSPR--- NAAGAPREQD
SbCML10-2   MGKVR SFFSRSRSGG S-----KRGA AGAPGASSPF SSAP-----P SPLPRRS--- PSAARAA--D
ZmCML10     MGKVR SFFSRSRSG- ------KRGS ARRAGSSSPH SSAASAPPSP SPLPRRS--- PSSSSTTTRD
ZmCML10-2   MGKVR SFFCRARSGK R-----G--- -----GSRPG LSSSSLS--- SPCPS----- ARRASPPAGD
PvCML10-3   GKRGAGPSSP SSSAAGSAPS SPSPEEVVGV GAGGPGGGDQ
AcCML25-2   MKCCV VFRPHKKIKF KGSRIFSETN IKVDENNL-K P-----AKIY GIVKKFKSWF HICEKSSLKP SGDYEKTLFR SSNSNTTQLR
PaCML25-3   MNLAV EKPQTLGLGT RSAKMRVPKF LRRKIRVLWK P-----RK-- NTVNTHQQEI NTDEKCAGVE ILQELEVVFR HFDANGDGKI
PaCML25-4   MAF RCLFRGQKAK VRPSCRQSRF SLCILPPRSR SSSPASRSPT S-----EHPE RSSRRRRNVS LKREGKDDKD VELFRPRS-- -SDLRSRSAV
PaCML25-2   MMSPLNYALL LLLLLPPCGR VV--LWRSKT S-----QSPK SSSSRSGKRS LRREGKDDEP VELCSPPPPP PSAMRSSSAV
PaCML25-5   MHFPMLV NCINGGQKSA NGSPRRRSKS S-----PTGS SRRPTNESTS S-----LRRR KTSASTKADE VNMEIDSDME RLSPSPSSSP SPPPLPTAEG
PaCML25-1   M GCPSFLNRKK TPADTSRRQS AMSNMFSSKS RPASDLSSRR AGSGSDKSPS N-----RRRK LSLSRRNDVV SDKKLDDVGV DVGVGAGVGA SRSAVVPSAA
PaCML25-7   MSAG FDLGLHALSL CVSVGVGVGA SHSEV-SSVI
PaCML25-6   MVA MHSTSKDRDG GIFKALRQKK KKIGKMGVRT PPLYNSFSIV
PaCML25-8   MSSGQRNMS SEQETVSQIE
AcCML27     MSRSNST KSSLLLENMD
AcCML23     MSSG GKVFSMENME
CcCML23-1   MAANNSN GANY-LGSMD
CsCML23-1   MAANNSN GANY-LGSMD
SlCML24     MAKTPSDSK QPAVSSAEMD
StCML23-2   MDKTPSDSK QPIVSSAEMD
SlCML23     MAKLTPRMK
StCML23-3   MAKLTARMK
MgCML27-2   MEKMEKPE
GmCML27-1   MDD
GmCML27-2   MDV
PvulCML27-  MDD
MtCML24     MDD
GmCML27-4   MDEE
PvulCML27-  MDE
CsatCML23   MD
BrCML23     MSKS G-RYCLGSME
CrCML23     MSKN GSRNCLGSME
AtCML23     MSKN VSRNCLGSME
BrCML24     MSKSNG A-RSCLGSMD
ThCML24     MSK-NG V-RSCLGSME
CrCML24     MSTKNG VVRSCLGSMD
AtCML24     MSSKNG VVRSCLGSMD
LuCLM24     MAKQSEAAA SPPIVTDTTS
ThCML23-2   MSKN GARNCLGSME
MeCML23     MARS GPTNLIGSMD
PtCML23-1   MAKS KNPTTFGSMD
PtCML23     MVDHTLKYGL SLLLYTPRER K-----HSFP PFLLSSYSSC KTKVSVFSLF SFPFCTMAKS RNPTAFGSMD
CpCML24     MARKAN STACCLGSMD
GrCML23-1   MATFLGSLE
GrCML23-2   MASFLGSVE
TcCML23     MASPLGSME
EgCML24-1   M GKSGGSSSIP KPRCSLGTMD
FvCML23     MSSRNL- --------RG GGVGS--SAE
MdCML27-5   MSKSDLV S-------KG SGDGAAGSME
MdCML27-6   MSKNDSI S-------KG SGIGASGSME
PperCML27-  MSKSNLV SSSPNPSWKG SGVGAVGSRE
FvCML27-1   MSSSFNS S-------SS SGSGVVVSME
BrCML27-2   MA SGNPVTA--- -------KPT PTPVDMANPE
AtCML27     MA SANPETA--- -------KPT PATVDMANPE
BrCML27-1   MA STNHETA--- -------KAT PSSVDMANPE
ThC27       MAETA--- -------KST PASVDMANPE
BrCML26     MA SENPETTN-- -------KPS S--TDM----
CrCML26     MA NTTPETTN-- -------KST TPSTDL----
AtCML26     MA NTNLESTN-- -------KST TPSTDM----
ThCML26     MA TANPDSTN-- -------KST T---DL----
CpCML27     MA STNPESISND AVAAVPQENS SSSIYMGNPE
CcCML27     MA ATTEADS--- --------NR KQLI--VSRE
CsCML27     MA ATTEADS--- --------NR KQLI--VSRE
GrCML27-2   MSATPT--- --------QK SSNV---NQG
TcCML27     MS TTTPSPQ--- --------PN TSSVYLENMD
GrCML27-3   MESTD
CsatCML27   M ATDPNPIPTS KSEADQIAKQ NPSVLLQDDE
GmCML27-5   M ATNPIEAGNG DAAPNPNATT KSSVYLQDSE
PvulCML27-  M ATNPIATGNG DAAPNPDAGA KSSVYLQDMD
MtCML27     M ATNP------ --NTETTTTT KSSVYLGDMD
GmCML27-3   M AANPPAPSES D--PNQNPGS ESFPYFEDMN
PvulCML27-  MA TTNPPVPSES D--PKPKSDP ASFPYFEDMN
LuCML27-1   MAEAISAEA SPKPTPTITS PAAG-TYSTD
LuCML27-2   MAESVLADA ATTPTATAAA EAAAYLQNME
LuCML27-3   MAESALANA TTAPTATAAA EAAAYLQNMD
PtCML27-2   MATPISNP SPETTAPASS TTIN-LDDTA
PtCML27-3   MATP NAKTTAPKPT TAVK-LDDTE
MeCML27-1   MASTTS STSLYFHNME
MeCML27-2   MASKPTS SSTLYFQDMD
EgCML26     MA TPPTPTQAPT PAAAAAAAAA PPHLPHMTNE
FvCML27-2   MASN GTPTPPPA-- --DAKPIVTD
PperCML27-  MATN GTPSPAQAPK SVDAKPVVTD
MdCML27-1   MATNGAPT STGPKPVVTD
MdCML27-2   LLYKPIDF L-----LSL- --LNSESSEN TQLPKNQKFP TTMATNGAPT STGPKPVVTD
```

```
MdCML27-3    MATNC APASTDPKPA VTDXKPAVTD
GrCML27-1    MD
MgCML27-1    MATNAA-- -------ANP QASMYLQDMD
SlCML27-2    MA ATDNVNNN-- -------SVS KPSLYLQNMD
SlCML27-1    MAT-- -------EGP KPCVYLQDME
EgCML23      MWKRSSS SSSSSFNSKS SSSSSSSSQA KPADSLGNVD
EgCML24-2    KSASLTTAG KAAGGLGNVD
BdCML16      MSSG KQKMRKGQVP ASLAAGAGAG EDA-------
OsCML16      MSNT TEKKMPQQQQ VERPT-ALAP ADA-------
PhCML16      MSNG -TEKQSHA-A AAAEAQKQRP PAVSA-----
PvCML16      MSNG -TETQAQA-A AAAQ-QQQRP PAVSA-----
SiCML16      MSNA STEKQAQATS AAAAAQSQRP PAVSA-----
SbCML16      MSTT SASENKQAQH QQA-RRPAAV VSSAADDA--
ZmCML16      MAMSSS ASKKQAQAQA QKAGRRPPAV SSAAAADADA
PhCML18      MASVKQ GGDAA-SA-- ---GGGGGPG MPAA------
PvCML18-2    MASVKQ GGDAAKGA-- ---GGGGGLG MPAA------
PvCML18-1    MASVKQ GGDAAKAA-- ---GGGGGLG MPAA------
SbCML18      MASVKG GDSAKAKA-- ---AGRGGRG MPVA------
SiCML18      MASVNK GRDSAKAAAG GAVGGGGGVG MPAA------
OsCML18      MPMA------
AcCML30      MSRLSLLN FQYNLSK-KL P-----SKPV RWGSS----D KQ-------- ------LSGL SVSY-QPNVE
AcCML30-2    MSRLSLLN FQYNLSK-RL P-----SKPV RWGSK----E RQ-------- ------CSGL SPSF-QPNTE
BrCML30      MSNVNFLE LQYKLSKNKM L-----RKPS RLFSRDRQSS GL-------- ------SSPG PVGVPQPTIN
ThCML30      MSKVSFLE LQYKLSKNKM L-----RKPS RLFSRDRKSS GL-------- ------SSPG SGGVSQPTIS
CrCML30      FI LWDRRAFLES VYQEKIGDLI SDMSNVSFLE LQYKLSKNKM L-----RKPS RMFSRDRQSS GL-------- ------SSPG PGGFSQPSVN
AtCML30      MSNVSFLE LQYKLSKNKM L-----RKPS RMFSRDRQSS GL-------- ------SSPG PGGFSQPSVN
LuCML30-1    MSNVSFLE FQYKLSRNKF L-----RKPT RILSSRSTSS RQ-------- ------NSAL PTTVLQPSLA
LuCML30-2    MSNGSFLE FQYKLSRNKF L-----RKPT RILSSRSTSS RQ-------- ------NSAL PTTVMQPSLT
CcCML30-2    MSNLSFLD FQYKISKNKF L-----RKPS RLFSSR---D RQ-------- ------NSSL LPTF-QADRN
CsCML30-1    MSNLSFLD FQYKISKNKF L-----RKPS RLFSSR---D RQ-------- ------NSSL LPTF-QADRN
MeCML30-1    MPNVSFLE FQYKLSRNKL L-----RRPS RLFSSR---D RQ-------- ------FSGA LPPF-QPNLN
PtCML30-1    MANLSFLE FQYKLSRNKF L-----RKPS RLFSSR---D RQ-------- ------SSGM LSTF-QPNMK
MdCML30-3    MANANFLD FQYNLSKRKF L-----RKPS QMFSFR---D RQ-------- ------YSGI APTF-EPNPN
PperCML30-   MSSANFLD FQYNLSKRKF L-----RKPS HVFSFR---D RQ-------- ------NSGV SPSF-EPNPN
MdCML30-4    MDMSNVNSLD FQYSLSKRKF L-----CKPS RIFSFQ---D RK-------- ------NSGL ALTF-EPNHN
VvCM30       MSTPSFLD FQYNISKRKY L-----RKPS RLFSSR---D RQ-------- ------NSDL PPIF-HLNMD
GmCML30-1    MSNTSLLD FQYSLSRKKY L-----RKPS RLFS-W---D RQ-------- ------NSGP KNVFPQPKAD
PvulCML30-   MPGTSFLD FQYNLSRKKY L-----RKPS RLFS-R---D RQ-------- ------NSGL NSVCLQPNAD
MeCML30-3    MSNISFLE FQYKLSRSKL L-----RRPS RLFSSR---S IQ-------- ------SSSK LPAILQPNLS
CsatCML30-   MPINPNFLE LQYNVSKKKF L-----REPS RLFSFK---D RQ-------- ------SSGL RRAL-QPSSE
MgCML30      MLPRFSFLD MQYNLSKKKF L-----KKPS RMFS------ -Q-------- ------NSGT LPMY-LPNIE
SlCML30-2    MSNPSFLD FQYNLSKRSF L-----RKPT RMFTR----E RQ-------- ------NSRS LPVY-QPSVD
StCML30-1    MPNPSFLD FQYNLSKRSF L-----RKPT RMFTR----E RQ-------- ------NSCS LPVY-QTSVD
EgCML30-1    MSRFRLN LQYSLSKIPS F-----KLGK RSLSKD---T SQ-------- ------RPAA APRIYRPTTE
EgCML30-2
CcCML30-1    MSKVSFLN F-YNGLSRKP S-----VKST PKESMPKERQ NP-------- ---------S VPRASRSNVQ
CsCML30-2    MAFQESP Q-----LNQP PKESMPKERQ NP-------- ---------S VPRASRSNVQ
GrCML30-1    MSKLSFIE FKYGAFPRKL S-----RKPT TQLSINKDSL KS-------- --------NV SSRTYQPNVE
TcCML30      MSKLSFIE FKYGGLPRKL S-----RKPT -QLSITKERQ QS-------- --------NV SSRTYQPNAE
GrCML30-2    MSKLSFIE FKYGSLKR-- --------PT DQLNSINKTR QS-------- --------SS NMSSYQPNME
MeCML30-2    MSSKNYSFLS FHGLSRKPSS -------LKP VQSRIHKEKQ TS-------- ---------- -PKSFQPNQE
PtCML30-2    MSHLSFLN FHGLPRNSSS S-----SLTP TESRIYREKR QSCSVSKSMQ QNVILGQSSN ESKSFQPNVE
GmCML30-2    MSNLSFLQ FHSSSPLGHS S-----SQPK SPTPNSNNFK SS-------- -----KHTSN VGCNIQPKSE
PvulCML30-   MSFLK IQNSSSLGHS S------QPK SAVPNSNNFK SN-------- -----Q-TSN MGCNFQPRSE
MtCML30-2    MSKLSFLK LHGSFSAKPI S-------PR KNIAEQNSFK LR-------- -----ETSNA E-MSFQPKKD
MtCML30-3    MSKLSFLK LHGSFSAKPI S-------PR KNIAEQNSFK LR-------- -----ETSNA E-MSFQPKKD
MtCML30-1    MSKLSFLK FHGSFSAKPT S-------PR KNIAQKNSFK LR-------- -----ETSST E-MSFQPKKD
PvulCML30-   MSNLSFLK FNYSFSWKPS S-------SR KHLAQKNSFI SR-------- -----QTSNN EGQSFQPKEE
MdCML30-5    MSNFSFFG FQYGTPRKPP N-----KPAP QMSNISKERS KR-------- -------YSF SRPSSQPKVE
PperCML30-   MYNLPKAE
CsatCML30-   MSSLLK LFRSCSGKRP S-----KNTS FSIPIKERSN SS-------- --------SP SSKHFRPNLE
SlCML30-1    M-----SKSR PSLHLSKVKQ TP-------- -------STT SLVHFSPKLE
StCML30-2    M-----SKSK RSLHLSKVKQ TP-------- -------STT SLVHFSPKLE
BdCML29      MSHLSILT FKYNLAKLQF K-----PA-- --RPTGRQLS SR-------- -----DRQPS DLMMYKPDDE
PhCML6       MSHLSILS FKYNLAKLRS K-----AGSK ASRPVGRPLS AR-------- -----DRQFS DLSTYKPDEE
SbCML1       MSHLSILS FKYNLAKLRS K-----AG-- --RPIGRPLS AR-------- -----DRQYS DLSTYKPDDE
BdCML20      MGLAASSFR RRPEKLLPDS
PhCML20      M-----GLVV SAAASCSDLL GRRRSTPPPP PIPARAASTQ QPDDGTTPEP
SbCML20      M-----GIVV SATASCGDIF GRRRSPAPPA PTATPAASTP PPTD-TTPEP
ZmCML20-2    M-----GLVV SATASCSDIF GRRRSSTLPP ATATPTSSTP PLTD-TTPEP
ZmCML20-1    M-----GLVV SATASCSDIF FSRRRSSSPP PPA--AASTP PPTY-TIPEP
SiCML20      M-----GLVV SATASCGDIL GRGRSPPPPP PTT--AATTQ PPNDGTTPDP
OsCML20      M-----GLVV SAAASCGRL- --RRSRSRSP PPAVLDPSQS PLSLEREAEP
AcCML25-5    MAQSGS-L SQQTEMLSQV
CcCML25      MAQSRP-L SEETETLNHV
CsCML25-2    MAESRP-L SEETETLNHV
GmCML25      MAQVGS-L SSEMETLNHV
PvulCML25-   MAQVGS-L CAEMETLNHV
MtCML25-2    MAQVSSHL SVEMETLNQV
MdCML25-2    MAQPSX-L SAETETLSQV
PperCML25-   MAQISS-L SAETETLSQV
TcCML25-2    MAQPGS-L SAETETLSHV
PtCML25-3    MAQLGS-L SAELESLNQV
CsatCML25    MAQAGS-L LVDTEALSHI
AcCML25-4    MHF SILNTHLKHL TITYLHYSLR FSLLTTSFIY LTFQFIIYIT PHHSVYLELS LPFSPSQVSI MFASCKQLSN S---SRNWVR NSIQKVKAKD TKKGKPKKLL SSGVDWIT-- -SSFAAMEVS
CpCML25-2    MFGC FRP--LSLHR R---VKTLLL RLKTPSRNKT WLSKIKHKRL SSSFDLLT-- -SSFATMEVS
GrCML25-2    MLSS CKTCWISLFK R---VQLFLH -------VDR CNRRSKHRRL PSSCDVIT-- -SSFATMELS
TcCML25-1    MLSS CKACWISLYK R---AKLFLH -------VKR CKRRNL-RRL SSSFDLTT-- -SSFGAMELS
PtCML27-1    MLNS FRTCLSSLHR R---AKNFLQ QPRNTERVSK CNRRKNKRLL SSSFDLLT-- -YSFAAMEMS
MtCML25-1    MLGL FETCFTFLNK K---AKFLFN QPRSMNIIRE PNTR------ -------L-- -SSFVDMEMS
PvulCML25-   MLQP FTTFFTFLHS K---TKSILN QPRIMMSACY KGNR-PKRKF SSGSDLSI-- -SSFLHMELS
RcCML25      MDNAN KSKKNEGKRL SSTFELLT-- -PSFAAMEVH
FvCML25      M S---CFRICL TALRRCTFSG CKSFRKKRRR SSNLDAPATA ASSFAAMEVS
PperCML25-   M S---CIRICL AASLRRITSG CKKTRKQGKL SSSFDVPAT- -SSFAAMEVS
MdCML25-1    M S---CIRIFL ESTIQRMTSG CKK-RKPPKL SSIFDAPAAA TSSFAAMEVS
CcCML23-2    MSGF FKTRLISLRR R---MKLFLR QLI------- KKRTKHKLLL SSIFDSLA-- -SSFAMMEVS
CsCML23-2    MEVS
MgCML25-2    S CCNNNNNNKH ASDLSWLT-- -SSFPSMEIS
StCML25-2    M D---SVTVWF KSIGKFVGQI KKGKKTPKRR ASDLSYL--- -SGFATMEVS
CsatCML36    MFNLTSSL FPTFFHRNIS SKPFFQKSLN NQKLPSFTTF
PpCML23      MKVP FFSKSKKSQS DVHLAISSCS RGSGNVETLC SGSSSFASSS QVSSTEKKLK KMSSFSGPLD K---LRKSSS FSSLTMPVRQ SSGKLFSFNL MRQKS----- -GTQTPTGYC
PpCML25-3    MKGFG SLGKLKK-QG SGTVDLSGIG TGNCNGNYGG NGPSVLVDVA VADSPKVEKA KKAP---RDE S---SKKQGL MSKITSRASL KLKKTKSGTG PPALR----- -VSK----SE
PpCML25-8    MKGFA SLGKSKK-QE SGTLDYSR-- ----NGN--G NGGPVVPAAG TSVSPKVNR- -------KVA I---TSRANL RSKRTNSE-- --KNGGGVTV PPVVS----- -GSK----SD
PpCML25-9    MKGFA SLGKSKK-QE SGTLDYSR-- ----NGN--G NGGPVVPAAG TSVSPKVNR- -------KVA I---TSRANL RSKRTNSE-- --KNGGGVTV PPVVS----- -GSK----SD
PpCML25-6    MMRLV SFGKSKKLQT SRSMDPTYV- ---------T EQSPKAVEKT TGKSNHEMQL -------ERS N---SKGRNF ITRLTSMGSL KVKRTKSGIV SQALN----- -APCTIHCSE
PpCML25-1    MATKTGNRT LESEPLVGLV
PpCML25-2    MGRMAL SKANRPEIEE IGSGRRPQTS ES-SLPPQLV
PpCML25-11   MMPA GWSGQTEIRG VSNLRGSYPG VS-DLPVDVV
PpCML25-10   ME VGNTKMSQ-- ---DLTASAV
PpCML25-4    MDLL MESCWTGAAN FGTTNPSLVS TVFDLPPQLV
PpCML25-5    M AATIRPSRPE IDLNLQPHLL
PpCML25-7    MDSRT GFAMNRKAAG FNLNLSPDLV
PpCML25-12   MK F---ISSLKK LGSKARFGSS SRSSSPNVSR SSSRRMEPAT GADSLAQSLV
SmCML27
SmCML26      MNKQQHPRHI
BrCML36      MKF AKLNPKRLF- RSKDRSTVSK STAS----SF -----SSGAA D---ECNNHS S----AVTGG SVTPTSILPE VSAV--HSPY SYVEI-----
ThCML36      MKF AKLNPKRLF- GSKDRSTVSK PGSSFGSASS -----SSSGA P---DCKNSS V----VVTGG SVTPTSILPE VSGD--PSPY SYVEI-----
CrCML36-1    MKL AKLNPKRFFI RSKDRSTVSK SP-AFSFGSA -----SSSSG Q---DCIKNS SISSDGGGGG SVTPTSILPD VS-----SPY SYVEI-----
AtCML36      MKL AKLIPKRFFI RSKDRSTVSK SPTAFSFGSA -----SSSSG Q---DC-KNS G---GDGGGG SVTPTSILPE VP-----SPY SYVEI-----
CrCML36-2    MMKL IKFNPKRFF- SSKKPSKVSR SGTS-SLGSA -----SSSSS G---ETKNLC ---------- -VTTSSPY-- ---------Y SHLEL-----
BrCML35      MKLAASL -----KRVFR SKSKG---SV SRSEPSSFSS A----ASSSS S---DGGSYG KLKPG----P AATPVSVLPQ SSG-----DF YSEL------
CrCML35      MKLAASL SRISPKRLFR SKSKA---SV SRSEPSSFSS N----ASSSS S---DG-SYG NLKPG----P TATPISVLPH NSG-----DF YTEL------
AtCML35      MKLAASL NRLSPKRLFR TKSKA---SV SRSEPSSFSS N----ASSSS S---DG-SYG NLKQG----P TATPISVLPQ NSG-----DF YTEL------
CpCML35      M KLVKM-NPKY LKFSPKRLFR SK-DRSDVSR --SDPPSFG- -----SSSSS D---ASSSIH KPSSGIGAPG SGTPTSVLPE ISGD--WSDI SPYICFEL--
MeCML36      MK QFIKIINPKR LNLTPKRLFR SKKDRSIVCR --SDPSSYSY GA--TSSSSS D---SFVSNH KGGSRDGVVD VGTPTSVLPE ISSD--WSDI SCDIYSEL--
FvCML36      MKLMKINS --LSPKRLFR SKKDRSAVSR --SDPPSFGS GT-----ASS S---SSDSLH KPG-----GP AATPTSVLP- --GD--WSEF SS---VDM--
MdCML36-1    MKFIKISS KNLSPKRLFR SKKDRSAVSR --SEPPSFGS ETASASSSSS S---YT--IH KPGAGA--GG GTTPTSVLPE RSGE--WSEF SADLQLDM--
PperCML36    MKLIKISS KSLSPKRLFR SKKDRSAVSR RSSDPLSFES GTASSSSSSS S---SSDSLH KPGTGAEAGG IGTPTSVLLE RSGD--WSDF STDLQLDM--
MdCML36-2    MKLIKISS KNLSPKCLFG SKDRS---SV --SRCPSFGS GS-DXSSSFS S---SPDSIH KPGAGA--GG ITTPTSVLPQ KSGV--WSHX SADL--DV--
MdCML36-3    MATSKIN RLKRFTSETF WKLPWQCLLS HHLTPHPFLS ASHLFLSLSS SFIIIILSLS TTSSPIWVFI FKHEVDQISS KNLSPKCLFG XXXRS---SV --SRCPSFGS GS-DFSSSFS S---SPDSIH KPGAGA--GG ITTPTSVLPQ KSGV--WSHF SADL--DV--
MdCML36-4    MVHPRSF LEVAMAVPSL TPYIPHLFLY ASHLFLSFSS FFTNIFSLSL QLLPPFGFSS SIMKFINISS KNLSPKRLFR SKKDRYAVSS --SEPPSFGS GT-DFSSSSS S---SSDSIH KPGAG----G VTTPTSVLAE RSGD--WSEF SADLQLDM--
MtCML36      MKFINP KNLSPKRLFR -KKEKSSVSR --SDPLSFG- -------SSS S---SDESLH KPIT----AG SQTPTSVLPE VSGE--WSDI TVDVQCEL--
CcCML36      MKLI EKLSPKRLFR SKSKKDRTSV SRSDPSSF-- -----SSSST S---DSSTHN KLGSAP--DR VGTPTSVLHE ISGD--WSDM SADISLDMNY
CsCML36      MKLI EKLSPKRLFR SKSKKDRTSV SRSDPSSF-- -----SSSST S---DSSTHN KLGSAP--DR VGTPTSVLHE ISGD--WSDM SADISLDMNY
LuCML36-1    MK VFKIITNPKS LNLSPSRLFR SKKSSRSTVS -RSDPSSFSS -----STSDS S---SSTNLK HPHS------ SATPKSVLPP QSS----GEW LSDLSGDL--
LuCML36-2    MK VFKIITNPKS LNLSPSRLFR SKKST-STVS -RSDPSSFSS -----SISDS S---SS---K HPHS------ SATPKSVLPH QSS----GEW SSDLSENL--
MgCML36-1    MKLTK--- -FI-PGTLFK SKKKSRSVSR SE-ETSSFG- ------SQTT A---SSSSTE N-------GG LSTPTSVLPS AD-------- ------DL-C
MgCML36-2    MKLTK--- -II-PGKLLK SKK-SRSVTR SEAETSSFS- ------SQTT S---SSSASS SEDVFKKPNG LSTPTSVLPT ASSAEISTEE WSEISADV-Y
SlCML36      MKLIT--- -DL-PKKLFK SKK-KRSISR SE--NTSFG- ------SVTT S---SSSSG- ---------S VTTPTSVLPS TN-----TQW PSEISAEV-Y
StCML36-2    MKLIT--- -NL-PKKLFK SKK-KRSISR SE--NPSFG- ------SVTT S---SSSSG- ---------S VTTPTSVLPS TT-----TQW PSEIPAEV-Y
StCML36-3    MKLLK--- -NF-PKKLFN YKK-TRSISR SQ--TSSFG- ------SSSD S---DSHPHP H-------RG FSKPTGSSTP TS-----VLP NEISAAEVGY
SlCML35      ME SYLNLLHFFF LSTSFINSYS FSKFSTM--- -KF-PKNFFK SKK-SRSLSR SD--DPSYS- ------SSSS S---SNKGGS VGG------- ISTPTSVLPT LSNEISADEW SDISTAGV-Y
StCML36-1    M--- -KF-PKNFFK SKK-SRSLSR SD--DPSYS- ------SSSS S---SNKGGG CGG-----GG ISTPTSVLPT LSNEISADDW SEISTAGV-Y
VvCML36      MKLIY--- -KINPKQLFR SKK-SRSVTR TD--PSSFS- ------SGTS S---STSS-- ---------- ---------- ---------- ----------
OsCML1-1     MMKLAHLFG SSSSSSSSKK ENKVSSKKRR SGAKSCSFG- ------STTS S---SS--LA ASSSDD--SA ATTPRSVLPT SA-------- ----------
OsCML1-2     MMKLAHLFG SSSSSSSSSS KKR--RKKRR SGAKSCSFG- ------STTS S---SSSSLA ASSSDD--SA ATTPRSVLPA SA-------- ----------
AcCML38      MKSKTP SPTHSSEGSV GSSGSTSPTQ SVFSKLRRKL SPRKSESQQQ T--LAAEPVV VLPRKIS--I NTH------- ---HITRTLS IDPKAPKCGE
EgCML37      MMKF GPSSRSSSSN SSSSPDRDPG T--KRESAFG RLRRRLS--P RRRSSAA--- DQSAAPVVEI AAGDCGKSGE
TcCML37      MYEIVLK EETRKNSPMS LTLNLEPPGP KEASQTSGSA ASATVYKQLA EANYQNYIQA NPLLLLATTC AFLPFTNSPT IRNKLQSTQT HPHRERERER ERERDQYIDA FRMNSNGDFI ATTSTTASSN S--QPKSVLA SLRRKLS--P RTKSCSE--- D--ARRCSSR SSNISEASSD
VvCML39-2    MEEVSPT RCRHRAEKSL ISSVQSLESV G--EGFIYKQ ITMRRED--T AASPACN--- SSPKSGSGKL SRRKGAACSQ
FvCML38-2    MDKAQQQ H--SPKSTLA KLCRKLS--S RKA------- ----ERRTLS SPTSSSCSSE
PperCML38-   MEITP K--SSSSALG RLCRKLS--P RKTKAGHHDV EGPIENPTTC SSISSNSDLQ
RcCML39      MQSHT LSPSLNHNTH S--SPKSPLS RLRRMLS--P RTP------- DQPSLSPPIN STTATSISSE
CcCML37      MK SEAATAHSHG G--RGQSSFE RLRRKLSFSP KTKKADNKEG SLSLSLVLKD ETESSSGSDE
BrCML37      MTLA N--IKKSPLA RLRSKLS--S RKSESSVD-E EEARSSTSS- NRSSSLNVDE
AtCML37      MTLA K--NQKSSLS RLYKKVS--S KRSESSRNLE DESRTSSNS- SGSSSLNVNE
ThCML39      MTLV D--IQKSPLS RLYKKYF--S RKSQPSGKLE DEEARRSST- S-TSSLNVDE
CrCML37      M--SQKSPLS RLYKKYS--K KRSESSSRTH EDEEARSST- S--SSLNVDE
CpCML37      MNTMMKSE G--DERNSLG RFWRKLL--S PKGQVERKRF LPSAVDSEDV ASSSSSSGSQ
```

```
SlCML38-1    MVSSVSVST AEKESFFSRL R--NMFHFRR NEDEKKTTTT TTTRAAAAAA TTTTNCVPVS DNGGSSNKGE
StCML38-3    MCIASVTVST AEKESFFSRL R--NIFHLKK KEEEKKTTTT TTT---ITTA TTTNSCV--- ---SSSNKGE
SlCML39-2    M SNMVLVTVSM AEKGSFFSRL R--KRFFLKK ANTPKKEHES VTTTTTITST STIDRLSV-- ---SDSNSGE
StCML37      MVLVSVST AEKESFFSRL R--NRFFLKK TNTPKKEHES VTTTTITTTT TTIDRLSV-- ---SDSNSGE
StCML38-2    MTTTNTR ATTTNCIPVS DNSGSSNKGE
SlCML38-2    METTISN TKNKSVYSKL K--NKFSSKK SIAIDASTTS R-------II TTS------- ---ISESSDQ
StCML38-1    METTISN TENKSVLSKL K--NKFSSKK SIAIDDSTIS RNNTLSASII TTS------- ---SSENNDQ
BrCML39-1    MK TSQRQLSSSF TKLCERLSSF RNGEDNIRD-
ThCML23-1    MK YTQRQLFSSF MKLCEQLSSD RNGESKNRD-
BrCML39-2    MKLYERLSSD INREDKNKD-
CrCML39      MK NIQRQLSASF MKFLD----- ----DKNRD-
AtCML39      MK NTQRQLSSSF MKFLE----- ----EKNRD-
BrCML38-2    ME K--IAQPQSS --FMKLCRKL SPKRKDSAA- --E-SQHIKN GDHD-KNRH-
ThCML38      M K--NTQPQSS --FKKLCRKL SPERKDSAA- --E-KRQNNR NGED-KNRD-
BrCML38-1    MMK N--TQEPQSS SSFVKFCRKL SPKRKDSPA- --ESTQHNIN EDQD-KNKD-
AtCML38      MK N--NTQPQSS --FKKLCRKL SPKREDSAG- --EIQQHNSS NGED-KNRE-
CrCML38      MKS N--NTQPQSS --FKKFCRKL SPERKASSAG EGEIQQHISS NAHDDKNRD-
AcCML41-2    MSSNSGSSS SSNTINVSKL S--KPSKWFF NKTFKISLPR SNSNKSSTIT PPPMSP--LQ LVKPHKFIKG
AcCML41-1    MSSNRGSSS SSNSSNVSKL S--KPSKWFF NKTFKLSLPR SNSNKSSTIA TPPMSP--LP SVKPNKFIKG
AtCML41      MATQK EKPSSNSFKW F--STKTLKL NLSFQNRRRS PKSNSSSTLN SPRSNSDDNN NIKSHQASK-
EgCML41      MANDG SAPSPKPFKW L--N-KSLRL TFPRRSSQSK SSSSNLSTPS SPTSTLPAPN LKRTISPNRE
CcCML41      MATDDNKVA GSKPSNSKWF F--SNKGHGL SLHRRRSKSK SSSSSSTTTT TLSSPNSSLS ACEPKKITKE
CsCML41      MATDDNKVA GSKPSNSKWF F--SNKGHGL SLHRRRSKSK SSSSSSTTTT TLSSPNSSLS ACEPKKITKE
MdCML41-2    MATTDRPI- -SKPS--KWF ---SNKTIKL SFPGRRSSK- -SSFSTSPNS PLSPTTARTG IISPRCTSKE
MdCML41-1    MATTDRPI- -SKPS--KWF ---SNKTIKL SFPGRRSSK- -SSFSTSPNS PLSPTTARTG IISPRCTSKE
MtCML41-1    MAR ILKPS--KWF ---ANK---L GLKLIRSR-- -SSNSSSL-C SPRSPISLST TPKTNTNEDE
PvulCML41    MATDR IFKPS--KWF ---SNKTLKL SLHRPTSR-- -SSNSSSH-S SNASPRSPMS TCTTPKKKEV
PtCML41      METER ASKSS--KWF ---SNKGLRL SLHRRRSK-- -SSSTSS--- SPNSPMSPC- TPKKDSTK-E
RcCML41      MAKER ILKPS--KWF ---SNKGLRL SLNRGRSK-- -SSSSSTLSC SPPSPMSPC- TPKNNTLKVE
SlCML41      MELIR SSKSF--SFS S--HYKNLRV ILPRLRSK-- ---SSNSSQS SPRTPKSS-H I-SRRNSSRE
StCML41      MESIR LSKSF--SFS S--HYKNLRV ILPRLRSK-- ---SSNSSQS SPRTPKSSKN I-SRRNSSRE
CpCML40      MNKQ
CcCML38-2    MKEKY
CsCML38-2    MKEKH
FvCML38-1    MDKL
MtCML41-2    MVMDKV
PvulCML38-   MMDKV
PvulCML38-   MDKL
EgCML40-2    MDKR
MgCML38-2    MERY
MgCML38-1    MERY
MgCML38-3    ME
CsatCML38-   MDKI
CsatCML41    MEKP
SlCML37-1    MAEY
StCML38-4    MAEY
SlCML37-2    M
StCML23-1    M
CcCML38-1    MVKG
CsCML38-1    MVKG
FvCML38-3    MENH
MdCML39-2    MESD
MdCML39-1    MESD
MdCML38      MGSH
PperCML38-   MTSN
TcCML38      MMKG
MtCML38-1    MKN
MtCML38-2    MMKN
MtCML38-3    MRSN
PtCML37      MVNA
PvulCML38-   MMKG
CsatCML38-   MEIVKSG
EgCML40      MVRLAEA
EgCML39-1    MVRLAEA
EgCML38      MVRLAEA
EgCML39-2    MVRLAEA
EgCML40-1    MARLAD-
VvCML39-1    MKRD
PvulCML38-   MVN
PvulCML38-   MRMVN
VvCML41-1    MINC
VvCML41-2    MINC
VvCML38      MINC
VvCML41-3    MINC
LuCML38      MV AMKELIMDKQ
CsatCML38-   MDKLS
AtCML40      MKSENVNKR
AcCML39      MFSY
BdCML31      MVVASSSSSW
OsCML31      MVVASAASP-
PhCML31      MVVASSYSAS ISTSTSTSTS
SbCML31      MVVAAPPS-S CSISISIGSR
PhCML19      MAVVT
SiCML19      MA
OsCML19      MVAAT
SbCML19      MAVYAS
ZmCML19      MAPAARAP
BdCML23      MTMVAT
SiCML23      MVAAS
OsCML23      MVAS
OsCML25      MA
OsCML26      MA
SlCML39-1
AcCML21      MGGVLGKDDS PK--KGW-IP ETKLEAKMVE AMQRRAAE-G SSMRSFNQLI LKFSKIDESL
MtCML21      MGGAMGKGDS PK--KFW-VA ETKLEAKMVE AMQRRECQ-G CSMKSFNTII LKFPKIDESF
VvCML21      MGGVVGKGDS PR--KAW-VP ETKLEAKMVE AMQRRASE-G STMKSFNSII LKFPKIDESL
CpCML21      MGGTLGKPKS PR--QVW-IP ETKLEAKMVE AMQRRASQ-G TAMKSFNSII LKFPKIDESL
TcCML21      MGGVAGKLES PE--MVW-VP ETKLEAKMVE AMQRRASK-G TAMKSFNSII LKFPKIDENL
CcCML21      MGGIVGKPES AT--STW-MP ETKLEAKMVE AMQRRAAE-G TALKSFNSII LKFPKIDDSL
CsCML21      MGGIVGKPES AT--STW-MP ETKLEAKMVE AMQRRAAE-G TALKSFNSII LKFPKIDDSL
FvCML21      MGAVVGKAES PR--SLS-IS ETKLEAKMVE AMQRRAAK-G SVMKSFNSII LKFPKIDESL
PperCML21    MGAVVGKGDS PR--QFS-IH ETKLEAKMVE AMKRRAVK-G SVMKSFDSLI LKFRKIDESL
MgCML21      MGGVVAKDES A---KSS-LQ ETKLESKIIE AVQRRELK-G SSMKSFNSII LKFPKIDESF
SlCML21      MGGVLGKNES PSSPKAS-VP ETKLEAKITE AMLRRETE-G SSIRSFDSLV LKFPKIDENL
StCML21      MGGVLGKNES PSSPKAS-VP ETKLEAKITE AMLRRETE-G SSIRSFDSLV LKFPKIDENL
CrCML21      MGAAVTKSES LQ--KEW-VP ETKLEAKIIE AVQRRATR-G TAMKSFNSIV LKFPKIDEGL
AtCML21      MGGAVTKSET LQ--KEW-VP ETKLEAKIIE AVQRRASR-G TTMKSFNSIV LKFPKIDDGL
ThCML21      MGGAVTKSET EQ--KEW-VP ETKLEAKIIE AVRRRASR-G TTTKSFNSIV LKFPKIDDGL
CsatCML21    MGSVVGKLES PR---EC-VP ETKLEAKMVE TMKQRATK-G SIIRSFDCII LKFPKIDDSL
SiCML33      MGGVIG-GDS P---RNS-SP ATKLERKMVE AMQQRALK-G TSVKSFNSVI MKFPKIDESL
SbCML33      MGGVMS-GDS P---RHS-SP ASKLEKKMVE AMKQRALK-G TSMKSFNSVI MKFPKIDENL
OsCML33      MGGIVGRGDS P---RHT-SP ASKLERKMVE AMQQRAQK-G TSVKSFNSVI MKFPKIDESL
SiCML29      MGAVLGRQDT Q---KRS-FH GSKLEAKMVD AMQQRASH-G TSLKSFDGIV MKFPRIDESF
SbCML29      MGAVLGRHDT H---KRS-FH GSKLESKMVD AMQKRALH-G TSLKSFDSII MKFPKIDESF
OsCML29      MGGILGRHDT M---KRS-SH GSKLETKMVE SMQQRASH-G TSLKSFDSII MKFPKIDESL
PaCML21      MVE AMRKKATSGG STLKSFNSII MKFPKIDESF
PpCML21      MGLIQS Q---ERR-GQ LTKIERRMVQ AMAERAQKVN VTLKTFNTFI LKFPKIDASF
AcCML22      M KESPGTSRIC PALKSFFSDK MGGILHHCNG TS--KCR-RL DPKLEKKLVE SIKRRASSGK NTFRSINSII MRFPQFKEGL
CsCML22      MGTSHPC PPLKYL-SNK VRGMLCHCGS QN--KYA-RL DAKLEKKMIE V--KKSSSGH SNFKSIDSVI VRFPQFKDGL
TcCML22      MGKCRPC PSLKSL-SST IGGMLCHCGS PN--KYK-KL DSKLERKMIE M--KRSASGQ SNFKSIDSII VRFPQFRDGL
PperCML12    MKG STSTGTSH-C PYLKSL-SNK VGSMLCCRGS TN--KYS-RL DAKLEKKMVE F--KRGSLGR SNFKSIDSII MRFPQFREEL
FvCML22      MLCCRGS AN--KYS-RL DAKLEKRMAE F--KRVSSGQ SNFRSIDSII LRFPQFREEL
CsatCML22    MSK VGSIFFCCSS SN--KYN-RL DAKLGRKMVE V--KRNSAGH DNFKSLNGII LRFPQFKEGL
MgCML22      M KDSQGTS--- -VLKSLYDKA QDIICCHVLL PN--TYK-RL DTKLERKMME I-KKSTSAHH INFKSIDSII MKFPQFRDGL
StCML22      NA PK--KYR-RL DTKLETKMVE L--KRSSSGQ SKFRSINSII LKFPHFKEGL
ThCML22      MLCCCVKSEN NK--KYA-EL DSKLERKMVE S--RRNYPGH RRLKSMDSII MKFPKLREGL
AtCML22      MLCCCVNSEN NK--KYA-EL DAKLARKMVE S--RSIYPGH RSLKSMDSII MKFPKLREGL
BdCML32      MGGAASRLAT PI--KQR-RV EKDLDNKVVE ALRERARARK KTFKSVNSIT MRLPRFKDGL
SbCML34      MGMVTSICME PI--KRR-RV EKDLDDKVAD ALQERTRSRQ RTFRSVNSIT MRLPRFKEGL
OsCML34      MSISVAKFA LILQNF-IAE MGCVESKLVA TI--KYR-RV EKDLDKKVAE ALKERTKSKK KTFRSVNSIT MGLPRFKEGL
SmCML21-1    MGGSISASR- ----REL-PD GDVVEKRLVK ALQQRAAAAK SSVKSFDAIL MKFSKIDMAF
SmCML21-2    MGIKGSKD- ----VQL-ND VSKIERRMVE AMRKRAKAGP SPFKSFNSIL MKFPKIDKSF
MpCML12      MKFPSMLSSF
BrCML32-1
PvCML36      SGIFTAILRG QLDLASEPWP HISPGAKDLV RKMLNINPKD RLTAFQVLNH PWIKEDGDAP DTPLDNVVLN RLKQFRAMNQ FKKAAL-RII AGCLSEEE-I
PvCML29      VI AENLSEEE-I
ZmCML36-1    MLMHCIRA ALLFFLLIED LYLFYFDQVI AESLSEEE-I
MeCML32      MMNR FKRKAL-RVI ADFLSIEE-V
ZmCML36-3    MNK FKKKAL-LVV AEYLPAEE-L
ZmCML36-2
MpCML11      MD-K
CreinCML20   MGTLN RALKCLFDHA YAG---EKLP PYDQEDAWRH ASVKVFGVVA ASSGADPKVG ITWR--DL-Y TYLSRNIPGG ASAKAVDALF HSLDRSSNGA VTADEFTEVV MKRLGTMREE RELESRLATA VPRIHVVPGS NRLNFLAKTE AFKVQVVTRA ---WVRDLGA
CreinCML20   MAD---VTSS LKSCRTAYKE AAAIVRMTEG GKERPEMAED SIGA--EMAL CRLAEMLVQP GNTTAAMSAF QQYDSSGDGY LDVGELCKAL RSLPGVQLSN HEV--RLLLA YLFHYGDKDK DLRLSVAELQ SSLAPFVPRP ---KEEELQR
AtCML48      MSYSNA
AtCML49      MSGYPP SSQGYGYG-- -GN-PPPPQP PYGS--TGNN PPPYGSSGSN P-PPPYGSSA --SSPYAVPY GAQPAPYGAP PSAPYASLPG DHNKPHKEKP HGASYGSPSP GG------YG AHPS-SGPSD ---YGGYGGA
AtCML50      MSGYPP TSQGYGYGYG GGNQPPPPQP PYSS--GGNN PPYGSSTTSS PYAVPYGASK PQSSSSSAPT YGSSSYGAPP PSAPYAPSPG DYNKPPKEKP YGGGYGAPPP SGSSDYGSYG AGPRPSQPSG ---HGGGYGA
CreinCML32   MS ATNPTRASTR VSQAPGGTSS ISFGDAPPPS
RcCML23      MAHITLLAFI TLLMIANVQS RSIRSSSDEQ LVPSGVEHLE INSSILSLKP LNSTQHTCVH YYSFLPCATN IPGFIFQIVV FEYLLILGDK FLTKGRQQLF SILGVGIYGA TLFRILAVLP TNVLILASGL AQNREDARAR IENGAGLLAG STVFCLTLQW GICVLLGRRK IAQESEPNQE
SbCML6       M AKPAAAATRK PSAASTVALT LALALASAGL -LFLLLRLSP SSPSPTPHPH RRLRLRARAH VHHHQIPFDP VVADIERRIE DREWERLAA- -----AGLHA
SbCML15      MA GSAVAVVFLS AVALLCLYNH -LHLSLS-DP ASVVPVPVPA AAVRSHRRAG GHRTNTTPSG SGNIVLRFGM SGQPLRVYSP APTPTAGGGI
VcCML5
AcCML18
MeCML18
RcCML18
SlCML18
StCML18-1
CpCML18
MgCML18
PtCML18-1
```

PtCML18-2
CcCML18
CsCML17
FvCML18-2
PperCML18
MdCML18
TcCML18
EgCML17
StCML18-2
GrCML18-2
GmCML17
PvulCML18
MtCML18
CsatCML18
GrCML18-1
BdCML11
PhCML11
SbCML11
ZmCML11
OsCML11
GmCML18
BrCML18
ThCML18
CrCML18
AtCML18
LuCML18-1
BrCML17
CrCML17
AtCML17
ThCML17
SlCML17
FvCML18-1
LuCML18-2
EgCML18
MdCML17-3
MdCML17-2
PpCML17-1
PpCML17-2
AcCML16
FvCML15
BdCML12
PhCML12
PvCML12
SiCML12
SbCML12
ZmCML14-1
OsCML12
BdCML14
PhCML14
PvCML14-1
PvCML14-2
SiCML14
SbCML14
ZmCML14-2
OsCML14
BrCML16-1
BrCML16-2
ThCML16
AtCML16
CrCML16
CpCML16
MeCML16-1
PtCML16
RcCML16
MgCML26
PperCML16
VvCML16-2
CcCML16
CsCML16
LuCML15-2
MeCML16-2
SlCML16
GmCML16
PvulCML16
EgCML15-2
CpCML15
CcCML15
CsCML15
CsatCML15
MdCML15-2
PperCML15
MdCML15-1
GrCML15-2
GrCML15-3
TcCML15
MeCML15
RcCML15
PtCML15
VvCML16-1
SlCML15
StCML15
BrCML15
CrCML15
AtCML15
ThCML15
CsatCML16
GrCML16
EgCML15-1
GmCML15-2
GmCML15-3
PvulCML15
GmCML15-1
MtCML15
GrCML15-1
FvCML16
LuCML15
AcCML20
GrCML20-2
FvCML20
MdCML17-1
MdCML20
PperCML20
CcCML20
CsCML20
CpCML20
PtCML20
MeCML20-1
CrCML20
AtCML20
ThCML20
CsatCML20
SlCML20
GrCML20-1
EgCML20
StCML20
GmCML20
PvulCML20
MtCML20
VvCML20-1
VvCML20-2
BdCML8
SbCML8
ZmCML8-2
PhCML8-1
PvCML8-2
ThCML19
AtCML19
MeCML20-2
MpCML20-2
SmCML20
OlCML20
VcCML20-1
VcCML20-2
PhCML8-2
PvCML8-1
PvCML8-3
ZmCML8-1
OsCML8
SmCML19
MpCML20-1
AcCML11
GmCML11-3
GmCML11-4
MeCML11-2
MeCML11-1
PperCML11-
GrCML11-3
BrCML11-2
BrCML11-3

```
    CrCML11
    AtCML11
    ThCML11
  BrCML11-1
    FvCML11
 PperCML11-
  GrCML11-2
  GmCML11-1
  GmCML11-2
  PvulCML11
  PtCML11-1
     BdCML4
     OsCML4
     PhCML4
     PvCML4
     ZmCML4
     PhCML5
   PvCML5-2
   PvCML5-1
     SiCML5
     ZmCML5
     SbCML5
     OsCML5
    EgCML11
     BdCML1
     BdCML3
     OsCML2
     OsCML3
  CsatCML50
     SiCML2
     ZmCML2
  GrCML11-1
  PtCML11-2
  PtCML11-3
 PperCML11-
     BdCML5
     OsCML6
    MdCML11
    AtCML13
    AtCML14
  BrCML34-2
  BrCML34-1
    CrCML34
    AtCML34
    ThCML34
  BrCML32-2
    CrCML32
  CrCML32-2
  CrCML32-1
    AtCML32
    AtCML31
    ThCML32
    ThCML33
    AtCML33
  EgCML25-2
    SmCML25
  LuCML25-1
  AcCML25-3
     BdCML2
    CrCML12
    AtCML12
  CsubCML24
     PpCML7
  MpCML21-1
     MpCML4
    MpCML30
    MpCML15
    MtCML46
    AtCML45
    AtCML46
    AtCML47
     VcCML4
   OsCML1-3
    AtCML44
    OlCML11
     AtCML1
  CsubCML20
    SiCML36
    SbCML50
    MtCML11
  Consensus                                                                                   ........ .......... .......... .......... .   ...... .......... .......... .......... ..........

             181                                                                                                                                                                                      360
   AcCML3-1  -LEKV-FSTF ---------D KNGDGFITRQ ELGDTLRNIG -----LFSS- --DKEVASMI EKLDIN-GDG LIDLEEFCEM Y--ESIGRSS TQHG------ LGE-DVKEDD ---------- --EEVELRDA FDVFDGDKDG ----VITVEE LVSVLSSLGL -KKGIQ---- --FEDCKEMI
     CcCML7  -LKRV-FATF ---------D KDGDGFITKT ELVESLRNLR -----LMVT- --DMEAEEMV AKVDAN-GDG LIEFDEFCML Y--EGMMGGD RQEKGGAGDG EGG-GGGGAD ---------- --EGDDLKDA FDVFDKDKDG ----LISVEE LGLVLSALGL -NEGNK---- --IENCKKMI
     CsCML7  -LKRV-FATF ---------D KDGDGFITKT ELVESLRNLR -----LMVT- --DMEAEEMV AKVDAN-GDG LIEFDEFCML Y--EGMMGGD RQEKGGAGDG EGG-GGGGAD ---------- --EGDDLKDA FDVFDKDKDG ----LISVEE LGLVLSALGL -NEGNK---- --IENCKKMI
   GrCML5-1  -LKRV-FSTF ---------D KNGDGFITKQ ELRESLKNIR -----MFMT- --ESEVEEMV VKLDAN-GDG LIDFDEFCVL C--KAIDDDD DGGGEEFKR- -------LGE ---------- --EGEILKEA FDVFDRDKDG ----LITVEE LGSVLCSLGL -KEGDK---- --IEDCKAMI
   GrCML5-3  -LKRV-FATF ---------D KNGDGFITKQ ELRESLKNIR -----VLMT- --EKEVEDMV VKVDAN-GDG LIDFDEFCIL C--QTMDGGT DASGTILKED ------DGMN ---------- --GEEELKEA FDVFDKDKDG ----LISFEE LGSVLCSLGL -KEGNK---- --MEDCKAMI
   TcCML5-1  -LKRV-FATF ---------D KNGDGFITKQ ELGESLKNMR -----LFIT- --EKEVEEMV VKVDAN-GDG LIDFDEFCIL C--QAMGGQG HQEGARRGED GNG-VYEEGD ---------- --AEGELKEA FDVFDKDKDG ----LISVEE LGSVLSSLGL -KEGNK---- --MEDCKAMI
   MeCML3-1  -LKSV-FATF ---------D KNGDGFITKQ ELRDSLKNIG -----IFTT- --EKEVDEMV VKVDSN-GDG LIDYEEFCLL C--NSSSMGG GDQDMVEEGS GGG-REIAEE ---------- --EEGDLKEA FDVFDRDKDG ----LISVEE LSLVLSCLGL -KEGKR---- --AEDCKEMI
     MeCML5  -LRSV-FATF ---------D KNGDGFITKQ ELGESLKNIR -----IFMT- --EKEVEEMV LKFDSN-GDG LIDYEEFCLL C--DYS-LGG GGSGGGQGLE DDG-ESGKEE ---------- --AEVDLKEA FDVFDRDKDG ----LISVEE LGLVLCSLGL -KEGKR---- --REDCREMI
   PtCML3-3  -LKSV-FATF ---------D KNGDGFITKQ ELRESFKNIR -----IFMT- --EKEVEEMV VKVDTN-GDG LIDFEEFCIL C--KAIGVRD QGGD-----E EK--EGQQDG ---------- --GEGDLKEA FDVFDKDKDG ----LISVEE LGLMLCSLGL -KEGGR---- --VEDCKEMI
   PtCML3-4  -LKSV-FATF ---------D KNGDGFITKQ ELRESFKNIR -----IFMT- --EKEVEEMV VKIDSN-GDG LIDFEEFCIL C--KVVGIQD QGGD-----D EK--EGQGDG ---------- --GEGDLKEA FDVFDRDKDG ----LISVEE LGLVLCSLGL -KEGGR---- --VEDCKEMI
     RcCML3  -LRSV-FATF ---------D KNGDGFITRQ ELRDSLENIR -----IIMT- --EKEVEEMV TKVDSN-GDG LIDFEEFCLL C--ESMAMPS SDQE-----R ESF-EGKDQE ---------- --AGGDLQEA FDVFDRDKDG ----LISVEE LGLVLSSLGL -REGRR---- --VEDCKAMI
     LuCML5  -MTSV-FATF ---------D KNGDGFITKQ ELKESLKNIR -----ISVT- --EKEAEEMV VKVDSN-GDG LIDLEEFKIL Y--RNMTAAG SGGG----DQ EAR-DPAVED ---------- --NGNVLKEA FEVFDRDNDG ----LITVEE LGSVLSSLGM -KEGKR---- --MEDCKEMI
   LuCML3-3  -MTSV-FATF ---------D KNGDGFITKQ ELKESLKNIR -----IIVT- --ETEVEEMV VKVDSN-GDG LIDLEEFKIL C--RKILSGG EDQ-----IS GCG-GAAGEG ---------- --GGGELKKA FDVFDRNKDG ----LITVEE LWWVLSSLGL -KEGKR---- --IEDCKEMI
   LuCML3-2  -MTSV-FATF ---------D KNGDGFITKQ ELKESLKNIR -----IIVT- --ETEVEEMV VKVDSN-GDG LIDLEEFKIL C--RKILSGG EEI-----SS G-R-GAAGEG ---------- --GGGELKKA FDVFDRNKDG ----LITVEE LWWVLSSLGL -KEGKR---- --IEDCKEMI
   GmCML5-1  -LRKV-FSTF ---------D KNGDGFITKQ ELRESLRNIR -----IFMT- --EQEVDDIV VKYDSN-GDG LIDFEEFCLL T--SECVGVD HE---KEGDG V------IEN ---------- --EEVDLKEA FDVFDKDNDG ----LISVEE LALVLTSLGL -REGRK---- --IEECKEMI
   GmCML5-2  -LRKL-FSTF ---------D KNGDGFITKQ ELRESLRNIG -----IFMA- --DKEVDDIV VKYDSN-SDG LIDFEEFCLL T--SECVGGD HH---EKEGG V------MGN ---------- --EEVDLKEA FDVFDKDNDG ----LISVEE LALVLTSLGL -REGRK---- --IEECKEMI
 PvulCML3-1  -LERV-FSTF ---------D KNGDGFITKQ ELRESLRNIG -----IFMT- --EKEVDDIV VKYDSN-GDG LIDLEEFCLL T--SECMGGN KEGGGEKEGG VGM-EGEEED ---------- --EEVNLKEA FDVFDKDNDG ----LISVEE LALVLTSLGL -NEGRK---- --TEECKEMI
     MtCML7  -LKKV-FSTF ---------D KNGDGFITKQ ELKESLRNIR -----IFMD- --EKEIDDIV GKFDSN-GDE LIDFDEFCLL T--SEFMGGG E---GEKEGG VGSKEDELEE ---------- --LEANLKEA FDVFDKDNDG ----LISVEE LALVLCSLGL -SEGNK---- --IEECKEMI
   CpCML3-1  -LKRV-FGTF ---------D KNGDGFITKQ ELRESMKSMR -----MSMT- --ETEVEEMV SKLDSN-GDG LIDFEEFCLV Y--DDGSHG- -EMG------ ----EEGGGE ---------- --ETGELKEA FDVFDKDRDG ----LISVDE LGSVLFALGL -QEGKR---- --VEDCREMI
   CsatCML5  -MKKV-FGTF ---------D KNDDGFITKK ELMESLKSMR -----MMIT- --EKDAEEML KGVDEN-GDG LIDFEEFCVL G--GKLMMGF EENK------ ----KTSVED ---------- --EEDELKDA FGVFDKDSDG ----LISVEE LSLVLCSLGM -NEGKI---- --VENCKEMI
     SlCML5  -LSGV-FATF ---------D KNNDGYITKQ ELKQSLKNIG -----IFME- --DGDILEMV EKVDFN-KDG LIDIDEFYEL C--HSFLGIE KVVS-----E EE---EEGGV ---------- --EEGDLKDA FDVFDYDKDG ----LISEEE LSKVLSSLGL -NQGKR---- --LDDCKEMI
     StCML5  -LRGI-FATF ---------D KNNDGYITKQ ELKQSLKNIG -----IFME- --DGDILEMV EKVDSN-KDG LIDIDEFYEL C--HSFLGIE KVVS-----E EEGGVEKGEE ---------- --EEGDLKDA FDVFDYDKDG ----LISEEE LSKVLSSLGL -NQGKR---- --LDDCKEMI
     FvCML5  -LKRV-FATF ---------D KNSDGFITKQ ELKESLKNIR -----VFMS- --DEEVDEMV KKVDAN-GDG LIDFSEFCLL C--QGQSILS ---G----EE EAGN---EVE ---------- --QEEELKEA FDVFDKDKDG ----LISVEE LGVVLCSLGL -REGNR---- --VEACKEMV
   MdCML3-1  -LKQV-FATF ---------D KNSDGFITTQ ELKESLKNIR -----ICMS- --DAEVEEMV KKVDAN-GDG LIDFDEFCML C--GS---MR ---R----RE DGGA---E-- ---------- ---EEELKEA FGVFDKDKDG ----LISVEE LAVVLCSLGL -REGSK---- --VEDCKEMV
   MdCML3-4  -LKQV-FATF ---------D KNSDGFITTQ ELKESLKNIR -----IXMS- --DAEVEEMV KKVDAN-GDG LIDFDEFCML C--GS---MR ---R----RE DGGA---E-- ---------- ---EEELKEA FGVFDKDKDG ----LISVEE LAVVLCSLGL -REGSK---- --VEDCKEMV
 PperCML5-2  -LKQV-FATF ---------D KNSDGFITKQ ELKESLKNIR -----IFMS- --DAEVEEMV KKVDAN-GDG LIEFDEFRML C--ESMHRRD QGVG----GE DGNGLSREEE ---------- --AEEELKEA FDVFDKDKDG ----LISVEE LG-------- ---GNK---- --VEDCKEMV
   EgCML5-1  -LKSV-FAMF ---------D RNHDGLIARE ELGESLRGLG -----ISVS- --DADLEEAV IGSDTD-GDG RLDLDEFCSL L--ESLSTAN A--NEREARR VDGDFCEGDG ---------- --WEVELKEA FDVFDRDRDG ----VITVEE LGLVLSSLGM -SQGKR---- --REECQEMI
   EgCML5-4  -LRRV-FATF ---------D KNGDGLITRE ELRESLRGLQ -----ISVS- --DEEVEEAV AGSDAN-GDG RIDLDEFCGL L--ESMSAVG SAVGSSSSDR SLGDG-ECGG ---------- --EEAELKEA FDVFDRNGDG ----VITVEE LEGVLSSLGM -SEGKR---- --AKACEEMI
     MgCML4  -LRGV-FATF ---------D KNNDGYITKH ELRDSLEKIG -----IEAG- --ESDVADMV QRVDSN-GDG LIDFDEFCVL F--DSFKDDE QGQ------- ------VKVH ---------- --EDGDLREA FDVFDENRDG ----LISVEE LGLVLSSMGF -NQGNR---- --IDDCKEMI
  LuCML30-3  -MTSV-FATF ---------D KNGDGFITKQ ELKESLKNIR -----ISVT- --EKEAEEMV VKVDSN-GDG LIDLEEFKIL C--R------ ---------- ---------- ---------- ---------- ----NRDNDG ----LITVEE LGSVLSSLGM -NEGKR---- --MEDCKEMI
   StCML3-1  -LRGI-FSTF ---------D KNNDGYITKQ ELKLSLKNIG -----IFME- --DKDIVDMV EKVDSN-KDG LIDLDEFYEL C--HTFLGIE GVEQ-----E GEMNEEEVAN ---------- --REQGLKEA FDVFDHDKDG ----LISEEE LSKILSSLGM -KEGKK---- --LDHCKEMI
    BdCML30  DLGIV-FSTF ---------D HDGDGFITAG ELEESLSRLG -----IAVS- --AAEAALMV ARVDAN-RDG LIDIHEFREL Y--DSIPKKR THNPSLPSSP VDAAAAEGAG DGDEDEEEE- --EEMDLREA FDVFDGNKDG ----LISAEE LGTVLGSLGL -RRPGPGGRR PAAAECRDMI
    OsCML30  DLGIV-FSTF ---------D HDGDGFITAA ELEESLKRLG -----IAVSS --AAEAAALV ARVDAN-SDG LIDIHEFREL Y--DSIPKRR KSHQQHPLPS TAAADEEAAA ADEEYEAEE- --EERDLREA FDVFDGNKDG ----LISAEE LGTVLESLGL -RQHGG---R PAVAECRDMI
    PhCML30  DLGIV-FSTF ---------D HDGDGFITAV ELEESLRRLG -----IAVS- --TDEAAAMV ARVDAN-SDG LIDIHEFREL Y--DSIPKKR KHQHPAAA-- LGAAREVPVE DAEEEEAEEE EEEERDLREA FDVFDGNKDG ----LISAEE LGTVLGSLGL -RRAGAG--R PAVAECRDMI
    SbCML30  DLGIV-FSTF ---------D HDGDGFITAV ELEESLRRLG -----IAVS- --ADEAAAMV ARVDAN-SDG LIDIHEFREL Y--DSIPKKR KHQHPAAD-F SGAAREVPVE GDDEEAEGEE EDEERDLREA FDVFDGNKDG ----LISAEE LGTVLGSLGL -RRQGNG--R TAVADCRDMI
    ZmCML30  DLGIV-FSTF ---------D HDGDGFITAV ELEESLRRLG -----IAVS- --ADEAAAMV TRVDAN-SDG LIDIHEFREL Y--DSIPKKR KHQHPAAGGF SGAAMEVPVE GDDEEAE--- --EERDLREA FDVFDGNKDG ----LISAEE LGTVLGSLGL -RRQGNA--R TAVADCRDMI
    SiCML30  DLGIV-FSTF ---------D HDGDGFITAV ELEESLRRLG -----IAVS- --ADEGAAMV ARVDAN-SDG LIDIHEFREL Y--DSIPKKR KHQHPAAASR EWVGNAFLTK SNIFIACDDE --EERDLREA FDVFDGNKDG ----LISAEE LGTVLGSLGM -RRAGSGAGR PAVAECRDMI
   AcCML3-2  ELKRV-FQMF ---------D HNGDGRITKK ELSDSLDKLG -----ICIP- --DKELVQMI DKIDVN-RDG CVDIEEFGAL Y--QTIMDE- ---------- -----RDEE- ---------- --E--DMKEA FNVFDQNGDG ----FITVEE LRSVLASLGL -KQGRT---- --VEDCRKMI
     CpCML5  ELKRV-FQMF ---------D KNGDGRITKK ELNDSLENLG -----IFIP- --DKELAQMI EKIDVN-GDG CVDIDEFGSL Y--KSIMDE- ---------- -----HDEE- ---------- --E--DMREA FNVFDQNGDG ----FITVDE LKSVLASLGL -KQGKT---- --VEDCKKMI
   MgCML3-2  ELRRV-FQMF ---------D RNGDGRITQK ELSDSLENMG -----IFIP- --DKELSQMI DKIDVN-GDG CVDIEEFGNL Y--QNIMDE- ---------- -----RDEE- ---------- --E--DMREA FNVFDQNGDG ----FITVDE LKAVLASLGL -KQGRA---- --VEDCKKMI
   PtCML3-1  ELKRV-FQMF ---------D RNGDGKITKK ELNDSLENLG -----IFIP- --DKELTQMI ETIDVN-GDG CVDIDEFGEL Y--QSLMDD- ---------- -----KDEE- ---------- --E--DMREA FKVFDQNGDG ----FITVDE LRSVLASLGL -KQGRT---- --LEDCKRMI
   PtCML3-2  ELKRV-FQMF ---------D RNGDGKITKK ELNDSLENLG -----IFIP- --DKELTQMI ETIDVD-GDG CVDIDEFGEL Y--QSLMDD- ---------- -----KDEE- ---------- --E--DMREA FKVFDQNGDG ----FITVDE LRSVLASLGL -KQGRT---- --LEDCKRMI
     PtCML5  ELKRV-FQMF ---------D RNGDGRITQK ELNDSLENIG -----IFIP- --DKELTQMI EKIDVN-GDG CVDIDEFGEL Y--QSLMDE- ---------- -----KDEE- ---------- --E--DMREA FNVFDQNGDG ----FITVDE LRSVLASLGL -KQGRT---- --FEDCKRMI
 PperCML5-1  ELKRV-FQMF ---------D RNGDGRITKQ ELNDSLENLG -----IFIP- --DKELFNMI QKIDVN-GDG CVDIDEFGEL Y--QSIMDE- ---------- -----RDED- ---------- --E--DMKEA FNVFDQNGDG ----FITVDE LRSVLSSLGL -KQGRT---- --IEDCKRMI
   StCML3-3  ELRRI-FQIF ---------D RNGDGRITKN ELNDSLENMG -----IFIP- --DPELIEMI EKIDVN-GDG CVDIDEFGSL Y--QTIMDE- ---------- -----RDEE- ---------- --E--DMREA FNVFDQNGDG ----FICVDE LKSVLASLGL -KQGRT---- --VEDCKQMI
   GrCML3-2  ELKRV-FQLF ---------D KNGDGTISKK ELNDSLENMG -----ICIP- --DPELTQMI EKIDVN-GDK CIDIDEFSEL Y--RSIMDN- ---------- -----KDEE- ---------- --E--DMKEA FNVFDQNGDG ----YISVEE LRSVLESLGI -KQGKG---- --IEDCKRMI
   TcCML5-2  ELKRV-FQMF ---------D KNGDGRITKK ELNDSLENLG -----IFIP- --DGELTHMI EKIDVN-GDN CVDIDEFGEL Y--HSIMDD- ---------- -----KDEE- ---------- --E--DMKEA FNVFDQNGDG ----YISVDE LRSVLVSLGL -KQGKT---- --IEDCKRMI
     CcCML3  ELDRV-FQMF ---------D HNGDGRISKK ELNDSLENLG -----IYIP- --DVELTQMI ERIDVN-GDG CVDIDEFGAL Y--KSIMEE- ---------- -----KDEE- ---------- --E--DMKEA FNVFDQNGDG ----FITFDE LKSVLGSLGL -KQGRT---- --VEDCKRMI
   CsCML3-1  ELDRV-FQMF ---------D HNGDGRISKK ELNDSLENLG -----IYIP- --DVELTQMI ERIDVN-GDG CVDIDEFGAL Y--KSIMEE- ---------- -----KDEE- ---------- --E--DMKEA FNVFDQNGDG ----FITFDE LKSVLGSLGL -KQGRT---- --VEDCKRMI
   EgCML5-3  ELKRV-FQMF ---------D RNGDGRITKK ELSDSLENLG -----IYIP- --DKELAEMI EKIDVN-GDG CVDIDEFGAL Y--RSIMEE- ---------- -----RDEE- ---------- --E--DMREA FNVFDQNGDG ----FITVDE LRSVLASLGL -KQGRT---- --LEDCKRMI
   GmCML5-3  ELKRV-FQMF ---------D RNGDGRITKK ELNDSLENLG -----IFIP- --DKELGQMI ERIDVN-GDG CVDIDEFGEL Y--QTIMDE- ---------- -----RDEE- ---------- --E--DMREA FNVFDQNADG ----FITVDE LRTVLSSLGL -KQGRT---- --VQDCKNMI
 PvulCML3-2  ELKRV-FQMF ---------D RNGDGRITKK ELNDSLENLG -----IFIP- --DKELTLMI ERIDVN-GDG CVDIDEFGEL Y--QHIMDD- ---------- -----RDED- ---------- --E--DMREA FNVFDQNGDG ----FITVEE LRTVLSSLGL -KQGRT---- --VEDCKKMI
     MtCML5  ELKRV-FQMF ---------D RNDDGRITKK ELNDSLENLG -----IFIP- --DKELSQMI EKIDVN-RDG CVDIEEFREL Y--ESIMSE- ---------- -----RDEE- ---------- --EEEDMREA FNVFDQNGDG ----FISVDE LRSVLVSLGL -KQGRT---- --VEDCKKMI
     FvCML7  ELKRV-FQVF ---------D RNGDGRISKE ELSHSLENLG -----IFIP- --DQELFNMI HKIDVD-GDG CVDIDEFGEL Y--QSIMDE- ---------- -----RDDE- ---------- --D--DMKEA FGVFDQNGDG ----FITVDE LRTVLSSLGL -KQGRS---- --MEDCTRMI
   BrCML4-1  DLKQV-FQMF ---------D KNGDGRITKE ELNDSLENLG -----IFMP- --DKDLIQMI HKMDAN-GDG CVDIHEFESL Y--GSIVVE- ---------- -----K---- ---------- --EEEDMRDA FNVFDQDGDG ----FISVEE LKSVMASLGL -KQGKT---- --LECCKEMI
     CrCML4  DLKRV-FQMF ---------D KNGDGRITKE ELNDSLENLG -----IFMP- --DKDLIQMI QKMDAN-GDG CVDINEFESL Y--GSIVEE- ---------- -----K---- ---------- --EEEDMRDA FNVFDQDGDG ----FISVEE LKSVMASLGL -KQGKT---- --LKCCKEMI
   BrCML4-2  DLKRV-FQMF ---------D KNGDGRITKE ELNDSLENLG -----IFMP- --DKDLIQMI RKMDAN-GDG CVDINEFESL Y--GSIVEE- ---------- -----K---- ---------- --EEEDMRDA FNVFDQDGDG ----FISVEE LKSVMASLGL -KQGKT---- --LKCCKAMI
     ThCML4  DLKRV-FQMF ---------D KNGDGRITKE ELNDSLENLG -----IFMP- --EKDLIQMI QKMDAN-GDG CVDIHEFESL Y--SSIVEE- ---------- -----K---- ---------- --VDEDMRDA FNVFDQDGDG ----YITVEE LKSVMASLGL -KQGKT---- --LECCKDMI
     AtCML4  DLKRV-FQMF ---------D KNGDGRITKE ELNDSLENLG -----IFMP- --DKDLIQMI QKMDAN-GDG CVDINEFESL Y--GSIVEE- ---------- -----K---- ---------- --EEGDMRDA FNVFDQDGDG ----FITVEE LNSVMTSLGL -KQGKT---- --LECCKEMI
   CrCML5-1  ELKRV-FQMF ---------D KNGDGRITKE ELNDSLENLG -----IYIP- --DQDLTQMI HKIDAN-GDG CVDIDEFESL Y--GSIVDE- ---------- -----HHNDG GT-------- --EEEDMKDA FNVFDQDGDG ----FITVEE LKSVMASLGL -KQGKT---- --LDGCKKMI
     AtCML5  ELKRV-FQMF ---------D KNGDGRITKE ELNDSLENLG -----IYIP- --DKDLTQMI HKIDAN-GDG CVDIDEFESL Y--SSIVDE- ---------- -----HHNDG ET-------- --EEEDMKDA FNVFDQDGDG ----FITVEE LKSVMASLGL -KQGKT---- --LDGCKKMI
   ThCML5-1  ELKRV-FQMF ---------D KNGDGRITKE ELNDSLENLG -----IYIP- --DKDLTQMI HKIDAN-GDG CVDIDEFESL Y--SSIVDE- ---------- -----HHNDG ET-------- --EEEDMKDA FNVFDQDGDG ----FITVEE LKSVMASLGL -KQGKT---- --LDGCKKMI
   BrCML5-1  ELKRV-FQTF ---------D KNGDGRITKT ELNDSLENLG -----IYIP- --DKELTQMI HNIDAN-GDG CVDIDEFESL Y--SSIVDE- ---------- -----HRKDG ET-------- --EEEDMKDA FNVFDQDGDG ----FITVEE LKSVMGSLGL -KQGKT---- --LEGCKKMI
   BrCML5-2  ELKRV-FQTF ---------D KNGDGRITKT ELNDSLENLG -----IYIP- --DKELTQMI HNIDAN-GDG CVDIDEFESL Y--SSIVDE- ---------- -----HRKDG ET-------- --EEEDMKDA FNVFDQDGDG ----FITVEE LKSVMGSLGL -KQGKT---- --LEGCKKMI
   BrCML3-1  ELCRI-FQMF ---------D KNGDGKITKQ ELNDSLKNLG -----IYIP- --EQELVQMI EKIDLN-GDG YVDSEEFGGL F--ETIMEE- ---------- -----RDE-- ---------- --EE-DMREA FNVFDQNRDG ----FITVEE LRSVLASLGL -KQGRT---- --LDECKRMI
   BrCML3-2  ELSRI-FQMF ---------D RNGDGKITKQ ELNDSLENVG -----IYMP- --DKDLVQMI EKIDLN-GDG YVDIEEFGGL Y--ETIMEE- ---------- -----RDE-- ---------- --EE-DMREA FNVFDQNRDG ----FITVEE LKSVLASLGL -KQGRT---- --LEDCKKMI
     CrCML3  ELCRI-FQMF ---------D RNGDGKITKQ ELNDSLENLG -----IYIP- --DKDLVQMI EKIDLN-GDG YVDIEEFGGL Y--QTIMEE- ---------- -----RDE-- ---------- --EE-DMIEA FNVFDQNRDG ----FITVEE LKSVLASLGL -KQGRT---- --LEDCKRMI
     ThCML3  ELCRI-FQMF ---------D RNGDGKITKQ ELNDSLENLG -----IYIP- --DKDLVQMI EKIDLN-GDG YVDIEEFGGL Y--QTIMEE- ---------- -----RDE-- ---------- --EE-DMREA FNVFDQNRDG ----FITVEE LRSVLASLGL -KQGRT---- --LEDCKRMI
     AtCML3  ELARI-FQMF ---------D RNGDGKITKQ ELNDSLENLG -----IYIP- --DKDLVQMI EKIDLN-GDG YVDIEEFGGL Y--QTIMEE- ---------- -----RDE-- ---------- --EE-DMREA FNVFDQNRDG ----FITVEE LRSVLASLGL -KQGRT---- --LEDCKRMI
   CpCML3-2  ELRRV-FQMF ---------D RNGDGKITKK ELNDSLENLG -----IYIP- --DKDLIQMI EKIDVN-GDG YVDIEEFGAL Y--QTIMDE- ---------- -----RDE-- ---------- --EE-DMKEA FNVFDQNGDG ----FITVEE LRSVLASLGL -KQGRT---- --IEDCKMMI
   PtCML3-5  ELRRV-FQMF ---------D KNGDGQITKK ELSDSLKNLG -----IYIP- --DKDLIQMI EKIDVN-GDG YVDIEEFGAL Y--QTIMDE- ---------- -----RDE-- ---------- --EE-DMREA FNVFDQNGDG ----FITVEE LKSVLSSLGL -KQGRT---- --LEDCKRMI
     EgCML3  ELRRV-FQMF ---------D RNGDGRITRK ELSDSLQNLG -----IHIS- --DKDLAQMI EKIDVN-GDG YVDIDEFGAL Y--QTIMDE- ---------- -----RDE-- ---------- --EE-DMKEA FNVFDQNGDG ----FITVEE LRSVLASLGL -KQGRT---- --LEDCKKMI
   GrCML3-1  ELRRM-FQMF ---------D KNGDGKITNK ELSESLENLG -----IFIP- --EKELTQMI EKIDVN-GDG YVDIDEFGAL Y--QTIMHE- ---------- -----RDE-- ---------- --EE-DMKEA FNVFDQNGDG ----FITVEE LRSVLSSLGL -KQGRT---- --IEDCKRMI
   CsatCML3  ELRRV-FQMF ---------D RNGDGRITKK ELSDSLENLG -----IFIP- --DKDLTQMI EKIDVN-GDG CVDIDEFGEL Y--QSIMDE- ---------- -----RDE-- ---------- --EE-DMREA FNVFDQNGDG ----FITVDE LRSVLASLGL -KQGRT---- --VEDCKKMI
   LuCML3-1  ELRRV-FQMF ---------D KNGDGQITKK ELSDSLQNLG -----IYIP- --EVDLTQMI EKIDVN-GDG FVDMDEFGEL Y--QTIMDE- ---------- -----RDE-- ---------- --EE-DMREA FNVFDQNGDG ----FITVDE LRSVLASLGL -KQGRT---- --VEDCRRMI
     FvCML3  ELRRV-FQMF ---------D RNGDGRITKQ ELSDSLENLG -----IFIP- --DKDLHQMI EKIDAN-GDG FVDIDEFGAL Y--QNLMDE- ---------- -----RDDRE DP-------- --EE-DMREA FNVFDQNGDG ----FITVEE LRSVLASLGL -KQGRT---- --LEDCKIMI
```

```
  MdCML3-2  EVRRV-FQMF ---------D QNGDGRITKK ELRDSLEKLG -----IYIP- --DKDLIQMI EKIDVN-GDG FVDIDEFGAL Y--QTIIGE- ---------- -----RDE-- ---------- --EE-DMREA FNVFDRNGDG ----FITVDE LQSVLSSLGL -KQGRT---- --LEDCKIMI
  CsCML3-2  ELRRV-FQMF ---------D RNGDGKITRK ELSDSLKNLG -----IYIP- --DNELVQMI EKIDVN-GDG YVDINEFGSL Y--QTIMDE- ---------- -----RDE-- ---------- --EE-DMKEA FNVFDQNGDG ----YITVEE LRSVLASLGL -KQGRT---- --VEDCKLMI
  SlCML3-1  ELKRV-FHMF ---------D RNGDGRITKK ELNDSLENMG -----IFMS- --DSDLAQMI NKIDVN-GDG YIDIDEFGAL Y--ETIMEE- ---------- -----RDE-- ---------- --EE-DIREA FNVFDQNGDG ----YITVDE LKSVLASLGL -KQGRT---- --VDDCKRMI
  StCML3-2  ELKRV-FHMF ---------D RNGDGRITKK ELNDSLENMG -----IFMT- --DSDLVQMI NKIDVN-GDG YIDIDEFGAL Y--ETIMEE- ---------- -----RDE-- ---------- --EE-DIREA FNVFDQNGDG ----YITVDE LKSVLASLGL -KQGRT---- --VDDCKRMI
  MeCML3-2  ELTRV-FQMF ---------D RNGDGRISKK ELNDSLENLG -----IFIP- --DPELTQMI QNIDVN-GDG CVDIDEFGAL Y--RSIMDE- ---------- -----RDEEE ---------- --EE-DMKEA FKVFDQNGDG ----FITVDE LRSVLAALGL -KQGRT---- --LEDCKTMI
    SlCML7  ELNRI-FQMF ---------D RNGDGTITKK ELNDSLQSLG -----IHIE- --DQELDHMI EKIDVN-GDG YIDVDEFGTL Y--KSIMDE- ---------- -----KDE-- ---------- --EE-DMREA FNVFDQNGDG ----YITVEE LRSVLGSLGL -KQGKT---- --LEDCKRMI
    StCML7  ELNRI-FQMF ---------D RNGDGTITKK ELNDSLQSLG -----IHIE- --EQELVHMI EKIDVN-GDG YIDIDEFGTL Y--KSIMDE- ---------- -----KDE-- ---------- --EE-DMREA FNVFDQNGDG ----YITVEE LRSVLGSLGL -KQGKT---- --LEDCKRMI
  SlCML3-2  EIRRV-FLMF ---------D RNGDGKITRK ELSDSLQNMG -----IYIP- --EMEFVQMI DKIDIN-RDG FVDMEEFGTL Y--QSIMDD- ---------- -----KDE-- ---------- --EE-DMREA FNVFDQNDDG ----FITVEE LRSVLSSLGL -KQGKT---- --LEDCKRMI
  GmCML3-1  ELKRV-FQMF ---------D RNGDGRISLK ELSDSLENLG -----ILIP- --DKDLAQMI ERIDVN-GDG CVDMDEFGDL Y--ESIMEE- ---------- -----RD--- ---------- --EEEDMREA FNVFDQNRDG ----FISVEE LRRVLASLGL -KQGGT---- --LDECKKMI
  GmCML3-2  ELKRV-FQLF ---------D RNGDGRISLK ELSDSLENLG -----ILIP- --DKDLAQMI ERIDMN-GDG CIDVDEFGDL Y--ESIMEE- ---------- -----PD--- ---------- --EEEDMREA FNVFDQNRDG ----FITVEE LGTVLASLGL -KQGRT---- --LDECKKMI
  GmCML3-3  ELKRV-FQMF ---------D RNGDGRITKK ELNDSLENLG -----IFIS- --DKDLSQMI QRIDVN-GDG CVDMDEFGEL Y--QTIMDE- ---------- -----RD--- ---------- --NEEDMREA FNVFDQNADG ----FITVDE LRTVLSSLGL -KQGRT---- --VQDCKAMI
PvulCML3-3  ELKRV-FEMF ---------D RNGDGRISVE ELRDSLVNMG -----IEIP- --EKELADMI QRIDVN-GDG CLDMEEFGEL Y--ESIMEE- ---------- -----RD--- ---------- --EEEDMLEA FNVFDQNRDG ----FISVDE LRTVLASLGL -HQGRS---- --LEECRKMI
  BrCML5-3  ELKRV-FQIF ---------D KDGDGRITKK ELNDSLKNLG -----IIIP- --EKELGQMI DKIDVN-GDG CVDIEEFGEL Y--KTIMVE- ---------- -----DEDE- -I-------- --GEEDMKEA FNVFDKNGDG ----FITVDE LKAVLSSLGL -KQGKT---- --LEECRKMI
  CrCML5-2  ELKRV-FQMF ---------D KDGDGRITTK ELNESLKNLG -----IIIP- --ESELTQII QKIDVN-GDG CVDIEEFGEL Y--KTIMVG- ---------- -----DEDE- -V-------- --GEEDMKEA FNVFDRNGDG ----FITVDE LKAVLSSLGL -KQGKT---- --LEDCRKMI
    AtCML6  ELNRV-FQMF ---------D KDGDGKITTK ELNESFKNLG -----IIIP- --EDELTQII QKIDVN-GDG CVDIEEFGEL Y--KTIMVE- ---------- -----DEDE- -V-------- --GEEDMKEA FNVFDRNGDG ----FITVDE LKAVLSSLGL -KQGKT---- --LEECRKMI
  ThCML5-2  ELKRV-FQMF ---------D KDGDGRITKK ELYESLKNLG -----IIIP- --ETELSKMI EKIDVN-GDG CVDIEEFGEL Y--KTIMVE- ---------- -----DEDE- -V-------- --GEEDLKEA FNVFDRNGDG ----FISVDE LKAVLSSLGL -KQGKT---- --LEECKQMI
    CrCML7  ELKRV-FQMF ---------D KNGDGKITGK ELSETLRSLG -----IYIP- --DKELTQMI EKIDVN-GDG CVDIEEFGEL Y--KTIMDG- ---------- -----EDEE- ---------- ---EEDMKEA FNVFDQNGDG ----FITVEE LKAVLSSLGL -KQGKT---- --LDDCKKMI
    AtCML7  ELKRV-FQMF ---------D KNGDGTITGK ELSETLRSLG -----IYIP- --DKELTQMI EKIDVN-GDG CVDIDEFGEL Y--KTIMDE- ---------- -----EDEE- ---------- ---EEDMKEA FNVFDQNGDG ----FITVDE LKAVLSSLGL -KQGKT---- --LDDCKKMI
  GrCML5-2  EFKKV-FQMF ---------D KNGDGRISKE ELNDSLKKLG -----IFIP- --DDELTQMI KKIDVN-GDN YVDIEEFREL Y--QSFMND- ---------- -----KD--- ---------- --EEEDILEA FKVFDQNGDG ----YISVDE LMSILASLGL -KQGKT---- --IEDCKKMI
    GrCML7  ELKRV-FQMF ---------D KNGDGKITKK DLIESLESLG -----IFIP- --EGDLIQMI EKYDVN-GDN CIDFDEFGEL Y--QSIMSD- ---------- -----KD--- ---------- --EEEDIKEA FKVFDQNGDG ----YISVDE LGSILVSLGL -KQGEK---- --AEDCKRMI
  BrCML2-1  ELSRV-FQIF ---------D KNGDGKIAKN ELREFFKSVG -----ILIP- --EKEINEMI AKMDVN-GDG FMDIDEFGSL Y--QEMMEE- ---------- -----NE--- ---------- --EEEDMREA FRVFDQNGDG ----FITGEE LRSVLASMGL -KQGRT---- --LEDCKKMI
  BrCML2-2  ELSRV-FKMF ---------D KNGDGKIAKN ELRDFFKSVG -----ILVP- --ENEIKEMI EKMDVN-GDG FMDIDEFGSL Y--QEMMEE- ---------- -----KE--- ---------- --EEEDMREA FRVFDQNGDG ----FITDEE LRSVLASMGL -KQGRT---- --LEGCRKMI
    CrCML2  ELSRV-FQMF ---------D KNGDGKIAKN ELKDFFKSVG -----IMVP- --ENEIKDMI EKMDVN-GDG FMDIDEFGSL Y--QEMVEE- ---------- -----KE--- ---------- --EEEDMREA FRVFDQNGDG ----FITDEE LRSVLASMGL -KQGRT---- --LEDCKKMI
    ThCML2  ELSRV-FQMF ---------D KNGDGKIAKN ELKDFFKSVG -----ILVP- --ENEIKEMI AKMDVN-GDG FMDIDEFGSL Y--QEMMEE- ---------- -----KE--- ---------- --EEEDMREA FRVFDQNGDG ----FITDEE LRSVLASMGL -KQGRT---- --LEDCKKMI
    AtCML2  ELSRV-FQMF ---------D KNGDGKIAKN ELKDFFKSVG -----IMVP- --ENEINEMI AKMDVN-GDG AMDIDEFGSL Y--QEMVEE- ---------- -----KE--- ---------- --EEEDMREA FRVFDQNGDG ----FITDEE LRSVLASMGL -KQGRT---- --LEDCKKMI
  GrCML3-3  ELRKV-FQMF ---------D KNGDGKITKK ELSDSLEEFS -----LFIS- --DKEINQII DSIDVN-GDG SVDIDEFGEL Y--QMMVNE- ---------- -----RN--- ---------- --EEEEMMEA FNVFDQNGDG ----FITFEE LRSVLSSLGL -KQGRT---- --IEDCKNMI
  GmCML3-4  ELARV-FQMF ---------D RNGDGRITRK ELSDSLKNLG -----ITIS- --EQDLTQMI EKIDVN-GDG FVDINEFGEL Y--QTIMDE- ---------- -----KD--- ---------- --EEEDMKEA FNVFDQNGDG ----FITGEE LSAVLCSLGL -KHGKT---- --IEDCESMI
  GmCML3-5  ELARV-FQMF ---------D RNGDGRITRK ELSDSLKNLG -----ITIL- --EQDLSLMI EKIDVN-GDG FVDMDEFGEL Y--QTIMDE- ---------- -----KD--- ---------- --EEEDMKEA FNVFDQNGDG ----FITGEE LSAVLCSLGL -KHGKT---- --IEDCKSMI
    MtCML3  ELARI-FQMF ---------D KNGDGRITKK ELSDSLQNLG -----ICIS- --EEDLVQMI EKIDVN-GDG YVDIDEFGEL Y--QTIMDE- ---------- -----KD--- ---------- --EEEDMKEA FNVFDQNGDG ----FISGEE LSAVLSSLGL -KHGKT---- --LEDCKNMI
PvulCML3-4  ELGRV-FQMF ---------D RNGDGRITRK ELSDSLKNLG -----ITIS- --EQDLTQMI EKIDVN-GDG YVDKDEFGEL Y--NAIMYE- ---------- -----KD--- ---------- --DEEDIREA FNVFDQNGDG ----FITGEE LSAVLCSLGL -KHGKS---- --VEYCMNMI
  MeCML3-3  ELQRV-FQMF ---------D KNGDGKITRK ELGESLQDLG -----IFIS- --DKELIQMI EKMDGN-RDG FVDFDEFGGL Y--ESISNE- ---------- -----KD--- ---------- --EDEDIREA FNVLDKNGDG ----FITWDE LRSVLASLGL -NQGRG---- --LEDCKRMI
   BdCML28  ELRKV-FQMF ---------D KNGDGQITKK ELGESFKNLG -----IYIP- --EDELDVTM EKIDTN-GDG CVDVEEFSSL Y--RSILAE- ---------- GE----GDDK GD-------- --EEDGLREA FDVFDRNGDG ----YITVEE LRSVLSSLGL -KQGRT---- --PEECRQMI
   PhCML28  ELRKV-FQMF ---------D KNGDGQITKK ELGESLRNLG -----IYIP- --DDELDAVM GKIDAN-GDG CVDVEEFGML Y--RSIVGE- ---------- GQ--GADGDK RD-------- --EEEDMREA FNVFDQNSDG ----YITVEE LRSVLASLGL -KQGRT---- --AEDCRKMI
   SiCML28  ELRRV-FQMF ---------D KNGDGQITKK ELGESLRNLG -----IYIP- --DDELDATM GKIDAN-GDG CVDVEEFGML Y--RSIVGE- ---------- GQ--GADGAK RD-------- --EEEDMREA FNVFDQNGDG ----YITVDE LRSVLASLGL -KQGRT---- --AEDCRKMI
 PvCML28-1  ELRKV-FQMF ---------D KNGDGQITKK ELGESLRNLG -----IYIP- --EDELDGIM GKIDAN-GDG CVDVEEFGML Y--RSIVGE- ---------- GQ--GGDGEK RD-------- --EEEDMREA FNVFDQNGDG ----YITVEE LRSVLASLGL -KQGRT---- --AEDCRKMI
 PvCML28-2  ELRKV-FQMF ---------D KNGDGQITKK ELGESLRNLG -----IYIP- --DDELDAIM GKIDAN-GDG CVDVEEFGKL Y--RSIVGE- ---------- GQ--GADDGD KR-------- --DEEDMREA FNVFDQNGDG ----YITVEE LRSVLASLGL -KQGRA---- --AEDCRKMI
   SbCML28  ELRKV-FQMF ---------D KNGDGQITKK ELGESLKNLG -----IFIA- --DEELDATM DKIDVN-GDG CVDVEEFGRL Y--RSIVED- ---------- GPVADADGDK HD-------- --EDEDMREA FNVFDQNGDG ----YITVDE LRSVLASLGL -KQGRT---- --AEDCRKMI
   ZmCML28  ELRKV-FQMF ---------D KNGDGQITKK ELGESLKNLG -----IHIS- --DDELDATM DKIDAN-GDG CVDVEEFGKL Y--RSIVGE- ---------- GQ-----VDK HD-------- --EEEDMREA FNVFDQNGDG ----FITVEE LRSVLSSLGL -KQGRT---- --AEDCRKMI
   OsCML28  ELRKV-FKMF ---------D KNGDGRITKK ELGESFKNFG -----IFIP- --DDELDATM DKIDAN-GDG CVDVEEFGLL Y--RSILGDD AAGRAPRTAA AAIGGEGGAP DD-------- --EDEGMREA FNVFDQNGDG ----FITVDE LRSVLSSLGL -KHGRT---- --ADDCRRMI
    MgCML3  DLHRV-FAMF ---------D RNGDGKISRK ELSESLEKLG -----IHIA- --EKELRQMI EKIDAN-GDG CVDAEEFGTL Y--AAVMEE- ---------- -----SERGG GD-------- --EEDDMREA FKVFDKNADG ----FITVEE LRSVLASLGL -KQGRT---- --LEDCKRMI
    MgCML7  ELRRV-FGMF ---------D RNGDGKISRR ELSESLEKLG -----IHIP- --EKELKQMI EKIDAN-GDG FVDVDEFGEL Y--AAIMED- ---------- -----E--GR AD-------- --EDEDMREA FNVFDQNGDG ----FITVEE LRAVLSSLGL -KQGRT---- --IEDCKRMI
   BdCML22  ELSRV-FQLL ---------D RNGDGRITRE ELEDCLGKLG -----IPVPG --D-ELAAMI ARIDAD-GDG CVDEEEFGEL Y--RAIMSTG G------GED EEKKGGDEGV EE-------- --DE-DMREA FRVFDANGDG ----YITVEE LGAVLASLGL -KQGRT---- --AEECRRMI
   PhCML17  ELARV-FELF ---------D KNGDGRITRE ELEDSLGKLG -----MSVPG --D-ELASMI ARIDAN-GDG CVDVEEFGEL Y--RAIMDG- -------GAG AGEEGGSAGE EA-------- --DD-DMREA FRVFDANGDG ----YITVDE LGAVLSSLGL -KQGRT---- --AEECRRMI
   SiCML22  ELARV-FELF ---------D KNGDGRITRE ELEESLGKLG -----MSVPG --D-ELASMI ARIDAN-GDG CVDVEEFGEL Y--RAIMAGD GGRAGGEGAG AGEEG-AGGE DA-------- --DE-DMREA FRVFDANGDG ----YITVDE LGAVLSSLGL -KQGRT---- --AEECRRMI
   SbCML22  ELARV-FELF ---------D KNGDGRITRE ELAESLRKLG -----MSVPG --D-ELASMI ARIDAN-GDG CVDVEEFGEL Y--RAIMAGD SS-----ANG AGKEGEAGGE EE-------- --DDEDMREA FRVFDANGDG ----YITVDE LAAVLSSLGL -KQGRT---- --AEECRRMI
   OsCML22  ELARV-FELF ---------D RNGDGRITRE ELEDSLGKLG -----IPVPA --D-ELAAVI ARIDAN-GDG CVDVEEFGEL Y--RSIMAG- -------GDD SKDGRAKEEE EE-------- --EDGDMREA FRVFDANGDG ----YITVDE LGAVLASLGL -KQGRT---- --AEECRRMI
   ZmCML17  ELARV-FELF ---------D KDGDGRITRE ELAESLRKLG -----MGVPG --D-ELASMM ARVDAN-GDG CVDAEEFGEL Y--RGIMDG- ---------- --AAEEEEEE DD-------- --DDDDMREA FRVFDANGDG ----YITADE LGAVLSSLGL -RQGRT---- --AEECRRMI
   OsCML17  ELRRV-FELF ---------D RDGDGRITRE ELTESLERLG -----MPVHR --E-ELAATI ARIDAN-GDG CVDMDEFTQL Y--ETVMR-- --------VD GGGGGGGGAC DV-------- --DEASMREA FDVFDRNGDG ----FITVDE LGAVLASLGI -KQGRT---- --AEDCGRMI
    SmCML5      M-FETF ---------D ENGDGKISCE ELGNTMKKLG -----FEMS- --RSELESMV VAVDND-GDG FVDFDEFLAL Y--SNIYYDD QHHR------ ----ARDGD- ---------- ---EQDLREA FSVFDKNKDG ----FITVVE LQAVLNSLGL -RDGGV---- -KLADCRRMI
    SmCML7  ELRRA-FDMF ---------D SNRDGMISRQ ELREIGDKLG -----MRWS- --DEETSSML ESVDEN-GDG LVDFGEFNAL Y--SQHIQGE EIQA------ ----AEEAR- ---------- --IKAELQEA FEVFDKNKDG ----FITALE LHSVLCSLGL -KHGS----- -DMVHVKNMI
   CcCML42  RLRRV-FDMF ---------D KNGDGMITVK ELHQALNLLG -----LETD- --LSELESTI ASHVKP-GND GLDFEDFVSL H--ESLDETF FPLNDLTSTT TTDADEGNKK VLSQ------ --EEADLSEA FKVFDEDGDG ----FISAHE LQVVLGKLGL -TEGNE---- --IARVQQMI
   MgCML42  RLRRI-FDLF ---------D KNKDSVITVD ELGEALSLLG -----LQTD- --ISQLESMV GAYVKQ-GNQ GLSYEDFESL H--RSLNDVF F------GSD VNNEIIGDHR SDDD------ --DESDLSEA FKVFDMDGDG ----YISARE LQSVLGKLGL HDEGRE---- --LDRVEMMI
   AtCML42  RLQRI-FDLF ---------D KNGDGFITVE ELSQALTRLG -----LNAD- --LSDLKSTV ESYIQP-GNT GLNFDDFSSL H--KTLDDSF FG-----GAC GGGENEDDPS SAAE------ --NESDLAEA FKVFDENGDG ----FISARE LQTVLKKLGL -PEGGE---- --MERVEKMI
   AtCML43  RLHRV-FDLF ---------D KNNDGFITVE ELSQALSRLG -----LDAD- --FSDLKSTV DSFIKP-DKT GLRFDDFAAL H--KTLDESF FG-----GE- -GSCCDGSP- ---------- ---ESDLEEA FNVFDEDGDG ----FISAVE LQKVLKKLGL -PEAGE---- --IEQVEKMI
   OsCML32  RLRRV-FDLF ---------D RNGDGEITVD ELAQALDALG -----LVAD- --RDGLAATV SAYVPE-GAA GLRFEDFDAL H--RALGDAL FG-----SLD GAAAAGEPGG GGGD------ --EEEEMREA FKVFDVDGDG ----FISASE LQEVLKKLGL -PEAGS---- --LATVREMI
 PaCML42-1  RLRKI-YDTV ---------D ENGDGKLTVE EISSFLNKLG -----MQMS- --DEDVESIV CPLYSS-EDG SLGFDQFISL Y--ESLCKDI ---------- ---GFSVDDE EGGE------ --SPDYLMEA FRVFDKNRDG ----YISSDE LQRILLNLGL -AEGTD---- --LGNCERMI
 PaCML42-2  KLRRI-FDAV ---------D ENGDGKLTIE EICGFLNKLG -----MQMS- --DEDLESIV RPLSSS-EDG SLKFEQFISL Y--ESLCQEI ---------- ---GLSTDHE EVGE------ --SPDDLMEA FRVFDKNGDG ----YISSEE LQQILHCLGW -GEGRD---- --LGNCEKMI
  PaCML5-2  GIRRL-YETV ---------D ENGDGKLTVT EMNRYLKRLR -----MDIS- --EEDLKYLI TPVSES-EDG SLTFDEFVSL C--QSILDN- ---------- ----SSEDEL HSDD------ --ECDDLMEA FKVYDMNNDG ----FISSTE LQQVLCNLGF -MQGEE---- --LANCQRMI
  PaCML5-1  EIRRL-YETI ---------D ENGDGRLTVT EMNNYLRRLG -----MEIS- --GEDLKYLA IPMSQR-EDG SLTFDEFVGM C--QSILDE- ---------- ----KSEDEL SNGD------ --GYEELMEA FKVYDMNNDG ----FISSPE LQRVLCNLGF -VEGEE---- --VANCQKMI
  PaCML5-3          MV ---------D ENGDGKLTVT EMNHYLKRLG -----LDIS- --EEDLKYLV IPMSES-EDG SLTFDEFVGL C--QSILDT- ---------- ----RSEDGL NDGN------ --GCEDLMGA FKVYDMNNDG ----FISSTE LQQVLCNLGF -MQGEE---- --LANCQKMI
    SmCML4  EFLRV-FQAI ---------D ENGDGLISKE EVGKLMAKLG -----HGMS- --DSDLELLM LTVDLN-GDG CVDFEEFQAL Y--ITSED-- ---------- ---------- ---------- --EEENLRDA FRVFDQNGDG ----FITAEE LHRVLSRLGF IQGARS---- --IAACKNMI
    SmCML3  ELWNV-FQEF ---------D SNRDGLICKG DIAQMMLRLD -----RSLS- --DRDVAATL EAIDED-GDG FVDFGEFCSI F--HGRRDIL DGEEAPDCEG EDQ------- ---------- --EEEDLMEA FRVFDRDNDG ----FITVEE LHTVLARLGF VEEHGG---- --RPSCSRMI
 AcCML25-1  ELKHV-FEKF ---------D VNGDGKISQF ELGSIMGSLG -----HS-AT --EEELQRMI EEVDSD-GDG FIDLNEFIEL N---TNGVD- ---------- -------SAK IL-------- ----DDLKNA FLIFDMDGNG ----SISPEE LQKVMKSL-- ---GES---- CSMDECRKMI
PvulCML25-  ELEYV-FRKF ---------D TNGDGRISSS ELGSMMKCLG -----QH-AT --EEEVSRMI QEIDCD-GDG HINLQEFIEL N---TKGVE- ---------- -------PDE VL-------- ----ENLKEA FSIFDIDGNG ----SITAEE LHMVMASL-- ---GDE---- CSLDECHRMI
   BrCML25  ELEAV-FKKF ---------D VNGDGKISSK ELGSIMASLG -----HE-VP --EEELQKAV AEIDKK-GDG YINLEEFVEL F---TKGMD- ---------- -------QND VL-------- ----ENLKDA FSVYDIDGNG ----SISAEE LHEVLRSL-- ---GDE---- CSIAECRKMI
   CrCML25  ELEAV-FKKF ---------D VNGDGKISSK ELGAIMKSLG -----HE-VP --EEELEKAI TEIDRK-GDG YINFEEFVEL N---TKGMD- ---------- -------QND VL-------- ----ENLKDA FSVYDIDGNG ----SISAEE LHEVLRSL-- ---GDE---- CSIAECKKMI
   AtCML25  ELEAV-FKKF ---------D VNGDGKISSK ELGAIMTSLG -----HE-VP --EEELEKAI TEIDRK-GDG YINFEEFVEL N---TKGMD- ---------- -------QND VL-------- ----ENLKDA FSVYDIDGNG ----SISAEE LHEVLRSL-- ---GDE---- CSIAECRKMI
 BrCML25-1  ELEAV-FKKF ---------D VNGDGKISSK ELGAIMTSLG -----HE-VP --EEVLQKAI TDIDRK-GDG YINFEEFVEL N---TKGMD- ---------- -------QNE VL-------- ----ENLKDA FSVYDIDGNG ----SISAEE LHEVLRSL-- ---GDE---- CSIAECRKMI
   ThCML25  ELEAV-FKKF ---------D VNGDGKISSK ELGAIMANLG -----HE-VP --EEELQKAI TEIDRK-GDG YINFEEFVEL N---TKGMD- ---------- -------QND VL-------- ----ENLKDA FSVYDIDGNG ----SISAEE LHEVLRSL-- ---GDD---- CSIAECRKMI
 CpCML25-1  ELEQV-FKKF ---------D ANGDGKISAA ELGAIMASLG -----HV-AT --EEELQKVV TEVDAD-GDG FIDFNEFVEL N---TKGMD- ---------- -------SDE VL-------- ----ENLKDA FSVYDIDGNG ----SITAEE LHEVLRSL-- ---GDE---- CSLADCRKMI
 EgCML25-1  ELEQV-FRKF ---------D ANGDGKISAS ELGSIMSSLG -----HP-ET --EEELAKMV REVDSD-GDG FIDFDEFVEL N---TKGVD- ---------- -------PAE AM-------- ----ENLRDA FSVYDIDGDG ----SITAEE LHKVMRSL-- ---GEE---- NSLAECRKMI
 MeCML25-1  ELEQV-FKKF ---------D VNGDGKISSS ELGSIMSNLG -----HQ-AT --EEELQKMI KEFDAD-GDG FIDFQEFVEL N---TQGMD- ---------- -------TDD VL-------- ----VNLKDA FSVYDIDGNG ----SISAEE LHKVMGSL-- ---GEH---- CSIAECRKMI
 MeCML25-2  ELEQV-FKKF ---------D VNGDGKISSS ELGSIMSSLG -----HQ-AN --EEELRKMI TEFDAD-GDG FIDFQEFVQL N---TQGVD- ---------- -------TDE VL-------- ----ENLKDA FSVYDIDGNG ----SISAEE LHKVMASL-- ---GED---- CSIAECKKMI
 PtCML25-1  ELEQV-FKKF ---------D VNGDGKISSA ELGSIMANLG -----HE-AT --EDELTQMI TEFDAD-GDG FIDLQEFVAL N---TQGVD- ---------- -------TNE VM-------- ----ENLKDA FSVYDVDGNG ----SISAEE LHKVMASL-- ---GEP---- CSMSECRKII
 PtCML25-2  ELEQV-FKKF ---------D VNGDGKISSA ELDSFWKKLG -----HE-AS --EEELQRMI TEFDAD-GDG FIDLQEFVAL N---TQGVD- ---------- -------TNE VM-------- ----ENLKDA FSVYDIDGNG ----SISAEE LHKVMASL-- ---GEP---- CSMAECRKMI
 GrCML25-3  EIEEV-FKKF ---------D ANGDGKISAS ELGSIMKSLG -----QQ-PS --DEELHNMI KEFDAD-GDG FINFNEFIEL N---TKGVD- ---------- -------SEE VL-------- ----ENLKDA FSVYDIDGNG ----WISAEE LHEVLKSL-- ---GEE---- CSIAECRKMI
 GrCML25-5  ELEHI-FKMF ---------D ANGDGKISSS ELASIMGSLG -----QQ-PS --DEEVQKMI KEFDAD-GDG FINFEEFVEL N---TKGVD- ---------- -------SKE VL-------- ----ENLKDA FSVYDLDGNG ----SISAEE LHKVLKSL-- ---GDD---- CSITECRKMI
 TcCML25-3  ELEEV-FKKF ---------D VNGDGKISSS ELGSIMGSLG -----HK-PS --EDELQKMI KEFDAD-GDG SINFKEFVEL N---TKGVD- ---------- -------SDE VL-------- ----ENLKDA FSVYDLDGNG ----SISAEE LHEVLKSL-- ---GDE---- CSIAECRKMI
 MdCML25-3  ELDQV-FKKF ---------D VNGDGKISSS ELGAIMSSLG -----QP-AS --DEELNGMI SEVDSD-GDG FINFKEFVEL N---TKGID- ---------- -------PDE AL-------- ----ENLKDA FSVYDIDGNG ----SISVEE LYKVLRSL-- ---GDE---- CSMDECRKMI
 MdCML25-4  ELDQV-FKKF ---------D VNGDGKISSS ELGAIMSSLG -----QP-SS --DEELNSMI SEVDSD-GDG FINFKEFVEL N---TKGVD- ---------- -------SAE AL-------- ----ENLKDA FSVYDIDGNG ----SISAEE LYEVLRSL-- ---GDE---- CSIDECRQMI
PperCML25-  ELDQV-FKKF ---------D VNGDGKISSS ELGAIMSSLG -----QP-AN --EEELSHMI LEVDSD-GDG FIDFKEFVEL N---TNGVD- ---------- -------QAE AL-------- ----ENLKDA FSVYDIDGNG ----SISAEE LYKVLRSL-- ---GDE---- CSIAECKKMI
 CsCML25-1  ELRQV-FNKF ---------D ANGDGKISAS ELGSILTSLG -----HA-AT --EEELQKMV REIDAD-GDG FVDFDEFVEL N---TKGVD- ---------- -------SAE VM-------- ----ENLKDA FSVYDIDGNG ----SITAEE LHQVLRSL-- ---GDD---- CTLAECRRMI
CsatCML25-  -LEQV-FNKF ---------D VNGDGKICSS ELGSIMGSLG -----QP-AT --EEELQNMI KEVDAD-GDG YIDLDEFIEL N---TKGVD- ---------- -------SDE VL-------- ----ENLKDA FSVYDIDGNG ----SITAEE LHEVLKSL-- ---GDD---- CSLADCRKMI
 MgCML25-1  ELEHV-FKKF ---------D VNGDGKICAA ELGSIMGSLG -----HP-AT --EEELRTMI QEVDSD-GDG FIDLREFIEL N---TNGIG- ---------- -------QDE VI-------- ----ENLRDA FEVFDMDKNG ----SISAEE LHNVLKSL-- ---GEE---- CTLAECRKMI
 MtCML25-4  DLEHV-FRKF ---------D VNGDGKISSS ELGSIMGSLG -----QP-AT --EEELDNMI REVDAD-GDG HINLEEFIEL N---TKDID- ---------- -------PNE IL-------- ----ENLKDA FSVFDIDKNG ----SISAEE LHNVMVSL-- ---GDQ---- CSLAECQKMI
 SlCML25-1  ELEQV-FKKF ---------D VNGDGKICSS ELGFIMASLG -----NR-AT --EEELVNMI REVDSD-GDG FIDLQEFIEL N---TKDID- ---------- -------SNE VL-------- ----ESLKDA FSVFDMDKNG ----SISAEE LLTVLQSL-- ---GED---- CNLAECRKMI
 StCML25-1  ELEQV-FKKF ---------D MNGDGKICSS ELGSIMASLG -----NP-TT --EEELVNMI REVDSD-GDG FIDLQEFIEL N---TKDID- ---------- -------SNE VL-------- ----ESLKDA FSVFDMDKNG ----SISAEE LLTVLRSL-- ---GED---- CTLAECRKMI
 MtCML25-3  ELEEV-FKKF ---------D VNGDGKISAS ELGSIMGSLG -----QQ-TS --EQELNNMI REVDGD-GDG CISLQEFIEL N---TKGVD- ---------- -------SDE IL-------- ----ENLKDA FAVFDMDGNG ----SITAEE LNTVMRSL-- ---GEE---- CSLAECRKMI
 PvulCML25  ELEQV-FKKF ---------D VNGDGKISAS ELGSIMRSLG -----QE-AT --EQEVDNMI REVDGD-GDG CISLQEFIEL N---TKGVD- ---------- -------SDE VL-------- ----ENLKDA FSVFDMDGNG ----LITAEE LNTVMRSL-- ---GEE---- CSLAECRRMI
 GrCML25-4  ELELV-FKKF ---------D INGDGKISSS ELGSVLGSLG -----LQ-HS --EDELLKII KEIDTD-GDG FIDFKEFVEL N---TKGVD- ---------- -------SNK VL-------- ----ENLKDA FLVYDIDGNG ----FITAEE LHQVFKSL-- ---GDE---- CSIMECRKMI
 SlCML25-2  ELQHV-FNKF ---------D INGDGKISFS ELGFIMASLG -----TA-VT --EEESIEMI NEVDGD-GDG FIDLREFIEL N---TKNID- ---------- -------SDE VM-------- ----ENLKDA FSVFDVDKNG ----SISAEE LQKVMKSI-- ---GEE---- CSLDECRKMI
 GrCML25-1  ELEQI-FNKL ---------D VNGDGKISLS ELGSIMGSLA -----QKHQS --KEELQKMM KEFNADYGDG FMNFDQFVEL N---TKGVE- ---------- -------SEE V--------- ------LKDA FSVYDRNGNG ----WISAEE LQEVLKSL-- ---GDE---- CSIEECRKMI
 LuCML25-2  ELDQV-FKKF ---------D VNGDGKISAS ELGMIMSSLG -----HR-AN --DDELQKMI TEFDAD-GDG FIDFDEFVAM N---TQGVG- ---------- -------SEE AM-------- ----VNLRHA FSVYDIDGNG ----SITAEE LHKVMISL-- ---GEE---- CSIAECRKMI
 LuCML25-3  ELDQV-FKKF ---------D VNGDGKISAS ELGMIMSSLG -----HR-AN --DDELQKMI TEFDAD-GDG FIDFDEFVAM N---TQGVG- ---------- -------SEE AM-------- ----VNLRHA FSVYDIDGNG ----SITAEE LHKVMISL-- ---GEE---- CSIAECRKMI
CsatCML25-  ELEEV-FKKF ---------D VNGDGKISSA ELGSIMRSLG -----HN-AT --EEELGKMI DEFDAD-GDG FINLHEFVEL N---TKEID- ---------- -------PEE LL-------- ----ENLREA FSVYDIDGNG ----SISAEE LHKVLQSL-- ---GDD---- CSVADCRQMI
 PaCML25-9  ELEDV-FKRF ---------D ANGDGKISSS ELGEILRSMG -----CR-VG --PRELGLMM KEADAD-GDG FISLEEFIDL N---TKGND- ---------- -------KAA CL-------- ----EDLKNA FKVFDLDRNG ----SISADE LYQVLKGM-- ---GDG---- SSREDCQNMI
   BdCML10  EMERV-FRKF ---------D ANGDGRISRP ELAALFESLG -----HA-AT --DDELARMM AEADAD-GDG FISLAEFAAL N--AAAAPG- ---------- -------DA- -E-------- ----EDLRLA FGVFDADGSG ----AISAAE LARVLHGL-- ---GEK---- ATVQQCRRMI
 PvCML10-1  EMARV-FRKF ---------D ANGDGRISRS ELAALFESLG -----HA-AS --DDELARMM AEADAD-GDG FISLDEFAAL N--ATVAGD- ---------- -------AAA VE-------- ----EDLRHA FRVFDADGNG ----TISAAE LARVLRGL-- ---GES---- ASVAQCRRMI
 SbCML10-1  EMARV-FRKF ---------D ANGDGRISRS ELAALFESLG -----HA-AS --DDEVARMM AEADAD-GDG FISLDEFAAL N--ATVAGD- ---------- -------AAA VE-------- ----EDLRHA FRVFDADGNG ----TISAAE LARVLRGL-- ---GES---- ASVAQCRRMI
 ZmCML10-1  EMARV-FRKF ---------D ANGDGRISRS ELAALFESLG -----HG-AS --DDEVARMM AEADAD-GDG FISLPEFAAL N--ATVAGD- ---------- -------AAA VE-------- ----EDLRHA FRVFDADGSG ----AISAAE LARVLRSL-- ---GEP---- ASVAQCRRMI
   OsCML10  EMERV-FRKF ---------D ANGDGRISRS ELGALFESLG -----HA-AT --DDELARMM AEADAD-GDG FISLDEFAAL N--ATASGD- ---------- -------AAA VE-------- ----EDLRHA FRVFDADGNG ----TISAAE LARVLHGL-- ---GEK---- ATVQQCRRMI
 PvCML10-2  EMARV-FRKF ---------D ANGDGRISRS ELAALFESLG -----HA-AS --DDELARMM AEADAD-GDG FISLDEFAAL N--ATVAGD- ---------- -------AAA VE-------- ----EDLRHA FRVFDADGNG ----TISAAE LARVLRGL-- ---GES---- ASVAQCRRMI
   BdCML15  EMERV-FRKF ---------D ANGDGRISRS ELAALFESVG -----HA-AT --DDEVARMM EEADAD-GDG YISLAEFAAI N--AAPD--- ---------- --------AA VE-------- ----EDLRHA FRVFDADGNG ----AISPAE LARVLRGL-- ---GEA---- ATVAQCRRMI
   OsCML15  ETERV-FRKF ---------D ANGDGRISRA ELAALFRSVG -----HA-VT --DDEVARMM QEADSD-GDG YISLGEFAAI S--APPPGD- ---------- -------AAA AE-------- ----EDLRHA FGVFDADGNG ----VITPAE LARVLRGI-- ---GEA---- ATVAQCRRMI
   PhCML10  EMERV-FRKF ---------D ANGDGRISRS ELAALFEGVG -----HA-AT --DDEVSRMM EEADAD-GDG AISLPEFAAL V--RSADAD- ---------- -------ADA VE-------- ----EDLRHA FMVFDADGNG ----LITPAE LARVLRGL-- ---GEA---- ATVAQCRRMI
   PvCML15  EMERV-FRKF ---------D ANGDGRISRS ELAALFEGVG -----HA-AT --DDEVSRMM EEADAD-GDG AISLPEFAAL V--RSADAD- ---------- -------AGA VE-------- ----EDLRHA FMVFDADGNG ----LITPAE LARVLRGL-- ---GEA---- ATVGQCRRMI
   SiCML10  EMERV-FRKF ---------D ADGDGQISRS ELAALFESVG -----HA-AT --DDEVSRMM EEADAD-GDG CISLPEFAAL V--RSADAD- ---------- -------AAA VE-------- ----EDLRHA FMVFDADGNG ----LITPAE LARVLRGI-- ---GEA---- ATVAQCRRMI
 SbCML10-2  ETERV-FRKF ---------D ANGDGQISRC ELAALFASVG -----HA-AT --DDEVSRMM EEADAD-GDG CISLTEFAAL M--DAASAD- ---------- -------AAA VE-------- ----EDLRHA FMVFDADGNG ----LITPAE LARVMRGL-- ---GES---- ATVAQCRRMI
   ZmCML10  ETERV-FRKF ---------D ANGDGQISRS ELAALFEGVG -----HA-VT --DDEVSRMM EEADAD-GDG CISLPEFAAL M--ESASAD- ---------- -------AAA VE-------- ----EDLRHA FMVFDADGNG ----LITPAE LARVLRGL-- ---GES---- ATVAQCRRMI
 ZmCML10-2  ETERV-FRKF ---------D ANGDGQISRS ELAALFESVG -----HA-AT --DDEVSRMM EEADAD-GDG YISLPEFAAL M--DSASGD- ---------- -------ADA VE-------- ----EDLRHA FSVFDADGNG ----LITPAE LARVLRGL-- ---GES---- ASVAQCRRMI
 PvCML10-3  GRDGARVRKF ---------D ANGDGRISRS ELAALFEGVG -----HA-AT --DDEVSRMM EEADAD-GDG AISLPEFAAL V--RSADAD- ---------- -------ADA VE-------- ----EDLRHA FMVFDADGNG ----LITPAE LARVLRGL-- ---GEA---- ATVAQCRRMI
 AcCML25-2  SVFEK----I ---------D ENGDGFISKY ELGSLMTTL- -----GHSVT --QEQLDKIF QEVDSN-KDG CIDFNEFLEL N--TIG-VD- ---------- -------SAK IS-------- ----DDLKGA FMMYDSDRNG ----FISPQE LLLVMRSL-- ---GDK---- CSITECIDII
 PaCML25-3  SVSEL-GAVI ---------N SHGDGKISVS ELGAVINSLF -----NYASS --EEELELIM KEIDSD-GDG LIDLQEFIHF N--TKG-EG- ---------- -------GFG SL-------- ----SELRDA FRIFDSDNDG ----FISAEE LRSVMASM-- ---GDR---- RSLEDCLRMI
 PaCML25-4  ELVEV-FEKF ---------D LDGDGKISQS ELGDFMRSL- -----GENPT --EEELRVVV SEADLD-GDG YIDLTDFVAL N--SEN-LD- ---------- -------TPR RL-------- ----QDLEEA FKMFDKDGDG ----FISPHE LLRVLTNL-- ---REP---- STLADCRAMI
 PaCML25-2  ELEEV-FKKF ---------D ANGDGRVSLL ELGDLMRSL- -----GGNPT --EEELGLIV EATDFD-GDE YIDLTEFVLL N--TEG-VD- ---------- -------SSR RL-------- ----EDLKAA FRMFDKDGNG ----TISPFE LLHVLTSL-- ---REP---- CTLGDCEAMI
 PaCML25-5  ELQVV-FRKF ---------D ANGDGKICWS ELGMLMTSL- -----GSSAT --DDELRLMV SVVDSD-GDG FIDFSDFVEI N--SFR-LD- ---------- -------DPR GL-------- ----QEMKSA FCIFDFDGNG ----LISPDE LLRVFENL-- ---GER---- CSLEDCRKMI
 PaCML25-1  ELEDV-FKKF ---------D ANGDGKISRS ELSELMKSL- -----GGNAT --EEEVGAMV SEADLD-GDG YIDLSEFVAL N--TDQTVS- ---------- -------SSR RL-------- ----QDLKDA FNVFDRDGNG ----SISPSE LHHVLTSL-- ---QEH---- CTIGDCHNMI
 PaCML25-7  ELEDV-FKKF ---------D ANDDGKISRS KLSELMKSL- -----GGNAT --KEEVGAMV SEADLD-GDG YIDLSEFVAL N--TDQTVS- ---------- -------RSR RL-------- ----QDLKDA FNMFDMDGNG ----SIFPPE LHHVLTSL-- ---QEH---- CTLGDCHNMI
 PaCML25-6  TCSAIGFQR- ---------D PLVLKIHNFE ELSSILSSLG -----YK-AT --EEEVQKII KEADRD-GDG FIDLQEFIEL N---TQGID- ---------- -------SAS SL-------- ----KDLRDA FEIFDLDRNG ----AISSEE LHVVLKNL-- ---GER---- STIEDCKLMI
 PaCML25-8  ELENV-FNAF ---------D EDGDGKISCR EVENIMSQFG -----FLNVT --EEQLQRMI NAMDSD-GDG FIDLQEFLEL N--TKDIMD- ---------- ------VMCE HM-------- ----DDLRSA FATLDLEQSG ----CITADE LHGVLNSL-- ---GEP---- TTIDDCRQII
   AcCML27  EVKKV-FNCF ---------D SNGDGKISST ELGDVLRALG ---SYTT--D ---EELQQMM EEIDKD-GDG FIDLDEFAEF H--RGGGSN- ---------- ------GENI NDDGV----- ----RELKDA FDMYDKDQNG ----LISATE LHMVLKSL-- ---GEK---- CSLQDCGRMI
   AcCML23  EVERI-FNRF ---------D SNGDGKISSS ELASVLEALG ---SDSS--Q ---EELKRMM SEIDLD-GDG FIDLKEFADF H--LHSGEE- ---------- ------TTAH NNNSH----- ----KDLKDA FDLYDKDKNG ----LISAAE LHLVFKNL-- ---GEK---- CTVRDCRKMI
 CcCML23-1  EVRKV-FNKF ---------D KNGDGKISAD ELKDVLRSLG ---SKTS--P ---EEVKRVM DEIDTD-GDG YIDFKEFSTF H--LAGGST- ---------- ---------- --DGT----- ----KELKDA FDLYDMDQNG ----LISANE LHAVLKKL-- ---GEK---- SSLKDCVNMI
 CsCML23-1  EVRKV-FNKF ---------D KNGDGKISAD ELKDVLRSLG ---SKTS--P ---EEVKRVM DEIDTD-GDG YIDFKEFSTF H--LAGGST- ---------- ---------- --DGT----- ----KELKDA FDLYDMDQNG ----LISANE LHAVLKKL-- ---GEK---- SSLKDCVNMI
   SlCML24  GVEKV-FRKF ---------D ANGDGKISLS ELGAILNALG ---SKTS--P ---DEVNRIM LEVDTD-GDG FIDLKEFAAF Y--CPRGAD- ---------- ---------- --GDN----- ----KELREA FDLYDKDKNG ----KITAAE LHSVMKSL-- ---GEK---- CSLKDCRRMI
 StCML23-2  EVEKV-FRKF ---------D ANGDGKISLS ELGAILNALG ---SKTS--P ---DEVKRIM LEVDTD-GDG FIDLKEFAAF Y--CPGGAD- ---------- ---------- --SDN----- ----KELREA FDLYDKDKNG ----KITAAE LHSVMKSL-- ---GEK---- CSLKDCRRMI
   SlCML23  EVEKV-FRKY ---------D TNGDGKISLS ELDGVLNALG ---TKTT--P ---DEAKRMM LEVDTD-GDG FIDLEEFAAF H--CPVEG-- ---------- ---------- --SNS----- ----KDLRDA FDLYDKDKNG ----KISASE LHSVMKGI-- ---GEK---- CSLKDCRRMI
 StCML23-3  EVEKV-FRKF ---------D TNGDGKISLL ELDEVLKALG ---TKTT--P ---DEAKRMM LEVDTD-GDG FIDLEEFAAF H--CPVEG-- ---------- ---------- --SNS----- ----KDLRDA FDLYDKDKNG ----KISAAE LHSVMKGI-- ---GEK---- CSLKDCRRMI
 MgCML27-2  EFEKV-FSKF ---------D ANGDGKISSA ELGAVLNGLG ---SKTS--D ---EEVARMM TELDTD-GDG FIDIVEFRKF N--RGGDA-- ---------- ---------- --DAD----- ----AELKEA FDMYDKDKNG ----QISASE LHAVLRTL-- ---GEN---- CSVKDCGRMI
 GmCML27-1  EVQQI-FNKF ---------D KNGDGKISMA ELKDMLSALG ---SKTT--D ---EELKRMI EELDQN-GDG FIDLKEFADF H--CNGGAG- ---------- ---------- -KDDS----- ----KELRDA FDLYDVDKNG ----LISAKE LHHVLRNL-- ---GEK---- CSLSDCRRMI
 GmCML27-2  EVRQI-FNKF ---------D KNGDGKISVT ELKDMLAALG ---SKTT--D ---EELKRMM EELDQN-GDG FIDLKEFADF H--CNGGAG- ---------- ---------- -KDDS----- ----KELRDA FDLYDVDKNG ----LISAKE LHDVLRNL-- ---GEK---- CSLSDCRRMI
PvulCML27-  EVRQI-FNKF ---------D KNGDGKISIT ELRDMLGTLG ---SKTT--K ---EELKRIM EELDQN-GDG YIDLKEFADF H--CND-AG- ---------- ---------- -NDES----- ----KELRDA FDLYDVDKNG ----LISAKE LHDVLSKL-- ---GEK---- CSLSDCRRMI
   MtCML24  EVRKI-FSKF ---------D KNGDGKISRS ELKEMLLTLG ---SETT--S ---EEVKRMM EELDQN-GDG FIDLKEFADF H--CTE-PG- ---------- ---------- -KDES----- ----SELRDA FDLYDLDKNG ----LISANE LHAVLMKL-- ---GEK---- CSLNDCKKMI
 GmCML27-4  EVRKI-FSKF ---------D KNGDGKISCA ELKEMMAALG ---SKTT--S ---DEVKRMM AELDRN-GDG YIDLKEFGEF H--CGG--G- ---------- ---------- -G-DG----- ----RELREA FELYDLDKNG ----LISAKE LHSVMRRL-- ---GEK---- CSLSDCRRMI
PvulCML27-  EVRKI-FNKF ---------D KNGDGKISCS ELRELMAALG ---SKTT--T ---EEVRRMM AELDRN-GDG YIDLKEFGEF H--CGG--G- ---------- ---------- -GGDG----- ----KELREA FELYDLDKNG ----LISAKE LHSVMRRL-- ---GEK---- CSLSDCRRMI
 CsatCML23  EIIRV-FNKF ---------D KNGDGKISVT ELAAALGELS ---GNIS--T ---DEIHRIM SEIDKD-GDG FIDLDEFTDF T--SSSTGG- ---------- ---------- ----N----- ----KDLQDA FDLYDIDKNG ----LISAKE LHSVLKRL-- ---GEK---- CSLKDCCRMI
   BrCML23  DIRAV-FQRF ---------D KNGDGKISID ELKDVIGALS ---PTAS--P ---EETTLMM KEFDLD-GNG YIDMDEFVAL F--QIE---- ---------- ---------- --DGGDI--- ----RDLKEA FDLYDLDGNG ----RISVNE LHSVMKNL-- ---GEK---- CSLEDCKRII
   CrCML23  DIKKV-FQRF ---------D KNNDGKISID EFKDVIGALS ---PNAS--Q ---EETKAMM KEFDLD-GNG YIDMDEFVAL F--QVKQ--- ---------- ---------- --DNDQI--- ----KDLKEA FDLYDLDRNG ----RISANE LHSVMKNL-- ---GEK---- CSIQDCKRMI
   AtCML23  DIKKV-FQRF ---------D KNNDGKISID ELKDVIGALS ---PNAS--Q ---EETKAMM KEFDLD-GNG FIDLDEFVAL F--QISDQS- ---------- ---------- -SNNSAI--- ----RDLKEA FDLYDLDRNG ----RISANE LHSVMKNL-- ---GEK---- CSIQDCQRMI
   BrCML24  DIRRV-FQRF ---------D KNGDGKISVD ELKEVIRALS ---PTAS--P ---EETVKMM KQFDLD-GNG FIDMDEFVSL F--QIGVNQ- ---------- ------GGG- -GGGNSK--- ----NDLKEA FELYDLDGNG ----RISAKE LHSVMKNL-- ---GEK---- CSVQDCRKMI
   ThCML24  DIRQV-FQRF ---------D KNGDGKISVD ELKEVIRALS ---PTAS--P ---EETVTMM KQFDLD-GNG FIDMDEFVAL F--QIGVNQ- ---------- ------GGG- -NNNNDF--- ----RDLKEA FEIYDLDGNG ----RISAKE LQSVMKNL-- ---GEK---- CSVQDCRKMI
   CrCML24  DIRKV-FQRF ---------D KNGDGKISVD ELKEVIRALS ---PSAS--P ---EETVTMM KQFDLD-GNG FIDLDEFVAL F--QIGIGG- ---------- ------GGGG GNNRNDV--- ----SDLKEA FELYDLDGNG ----RISAKE LHSVMKNL-- ---GEK---- CSVQDCKKMI
   AtCML24  DIKKV-FQRF ---------D KNGDGKISVD ELKEVIRALS ---PTAS--P ---EETVTMM KQFDLD-GNG FIDLDEFVAL F--QIGIGG- ---------- ------GG-- -NNRNDV--- ----SDLKEA FELYDLDGNG ----RISAKE LHSVMKNL-- ---GEK---- CSVQDCKKMI
   LuCLM24  QIRQI-FDKF ---------D KNGDGKISCS EVVDSLKELG ---TAVT--P ---AEVGCMI KEFDKD-GDG YIDPDEFVGF I--QSGGFE- ---------- ------DSES VDGSSRRGGG AAGRKELKDA FDLYDMDKNG ----LISANE LHAVMKML-- ---GLK---- CSMGECKKMI
 ThCML23-2  DIRKV-FQRF ---------D KNGDGKISID ELKDVIRALS ---PTAS--P ---EETKSMM KELDLD-GNG FIDMDEFVAL F--QIG---- ---------- ---------- -DGNSNGNDA ----QDLKAA FDLYDLDGNG ----WISAKE LQSVMNNL-- ---GEK---- CSIQDCKKMI
   MeCML23  EIQRI-FNKF ---------D KNGDGKISSS EVIDSLGELG ---TTIS--T ---EEVRCMM QEFDKD-GDG YIDLDEFVDF I--QRGGTD- ---------- ------CDAD GKTQSGDGN- ----KELKDA FDLYDIDKNG ----LISANE LHKVMRMI-- ---GLK---- CTLGDCTKMI
 PtCML23-1  DIRKI-FNKF ---------D KNGDGKISCS EVVDNLKELG ---TKIS--P ---AEVQSIM QEFDKD-GDG YIDLDEFVDF I--QNGGLD- ---------- ---------- --DGGGNDS- ----KELRDA FDLYDKNKNG ----LISVDE LHSVMKML-- ---GLK---- CSLSDCRKMI
   PtCML23  DIRKV-FNKF ---------D KNGDGKISCS EVVDNLSELG ---TKIS--P ---AEVELIM QEFDKD-GDG YIDLDEFVGF I--QNGGHG- ---------- ---------- --DSGGNDS- ----KELRDA FDLYDTNKNG ----LISVDE LHSVMKML-- ---GLK---- CSLSDCRKMI
   CpCML24  DIRKV-FNKF ---------D KNGDGKISGD ELREILLALG ---SGAT--V ---EEVARMM RELDKD-GDG YIDLDEFTAE F--QRAEVA- ---------- ------AGED GADQSVASN- ----KELKDA FDLYDIDGNG ----LISASE LHSVLKKL-- ---GES---- CSLRDCKKMI
 GrCML23-1  DMRAV-FNKF ---------D KNGDGKICRD ELKSTLSALG ---FAFS--S ---DEVDLIM LEMDKD-GNG YIDVEEFIA- F--HGIS--- ---------- --------GI DGDEKQCDN- ----KELKDA FDIYDLNKDG ----LISANE LHAVLKRL-- ---GEK---- YSLSDCQRMI
 GrCML23-2  EMKKV-FNKF ---------D KNGDGKISRD ELKSVLTALG ---SAPS--S ---GEVDRIM SEMDKD-GNG YVDLDEFVA- F--QQTNI-- ---------- --------ND DDGEAQCGN- ----KELKDA FDMYDLDKNG ----LISANE LHAVLKRL-- ---GEK---- CSLSDCRRMI
   TcCML23  EMKEV-FNKF ---------D KNGDGKISRD ELKSILSALG ---SPPS--S ---DEVDRIM SMMDKD-GNG YVDLDEFIA- F--HGTNI-- ---------- --------NG NGDDSQCGN- ----KELKDA FDMYDLDKNG ----LISANE LHAVLKRL-- ---GEK---- CSLSDCRRMI
 EgCML24-1  EVRLV-FNKY ---------D ANGDGKISCD ELRQALSALG ---PTAA--T --LEEARRAL SEIDMN-GDG FIDLNEFAEI F--TWGPG-- ---------- ---------- -GGRNEQ--- ----EELKGV FDIYDLDKNG ----RISAKE LHAVMKKL-- ---GEK---- CSLSDCRRMI
   FvCML23  ELKKI-FNKF ---------D KNGDGKICRD ELREIFRELG ---SETT--P ---EEVKTIM AEFDKD-GDG RIDLDEFAEI L--NGG-ST- ---------- ---------- ---------- ----KDLRDA FDLYDLDKNG ----RISASE LHEVLKRL-- ---GQK---- CSLKDCAKMI
 MdCML27-5  ELKKV-FDKF ---------D KNGDGRISCD ELRGVFGDLG ---SETA--S --LEEVQRIM AEFDKD-GDG HIDIEEFAEI I--NGG-ST- ---------- ---------- ---------- ----KELRDA FDLYDLDKNG ----LISTSE LHEVLKRL-- ---GQK---- CSLKDCAKMI
 MdCML27-6  ELKKI-FNKF ---------D KNGDGRISCD ELRGIFGELG ---SETA--S --LEEVQRIM AEFDKD-GDG HIDIAEFAEI I--NGG-ST- ---------- ---------- ---------- ----KELRDA FDLYDLDKNG ----LISKSE LHEVLKRL-- ---GQK---- CSLKECAKMI
PperCML27-  QLETI-FNKF ---------D KNGDGKISCD ELRGVFGELG ---SDTS--S --LEEVRSIM AEFDRD-GDG HIDILEFAEI M--NGG-SS- ---------- ---------- ---------- ----QELRDA FDLYDLDKNG ----LISVSE LHEVMKRL-- ---GLK---- CSLQDCVKMI
 FvCML27-1  EIKKV-FNKF ---------D ENSDGLISGD ELKSVFLALG ---WEMK--P ---EEVKTVM SEFDKD-GDG HIDLEEFTAI L--NGSPST- ---------- ---------- ---------- ----KDLRDA FNLYDLDKNG ----LISTSE LHEVLNRL-- ---GQK---- CSVLECAAMI
 BrCML27-2  ELKKV-FDQF ---------D SNGDGKISVT ELGGVFKAMG ----TSY--T --ETEINRVL EEVDTD-RDG FINVDEFSAL C--RSSS--- ---------- ---------- SA-------- ----SEIRDA FDLYDQDKNG ----LISAAE LHQVLNRL-- ---GMS---- CSVEDCAKMI
```

```
      AtCML27  ELKKV-FDQF ---------D SNGDGKISVL ELGGVFKAMG ----TSY--T --ETELNRVL EEVDTD-RDG YINLDEFSTL C--RSSS--- ---------- ---------- SA-------- ----AEIRDA FDLYDQDKNG ----LISASE LHQVLNRL-- ---GMS---- CSVEDCTRMI
    BrCML27-1  ELKKV-FDQF ---------D SNGDGKISVA ELGGVLKAMG ----TSY--S --ETELNRVL EDVDAD-RDG YINVDEFSTL C--RSSS--- ---------- ---------- TA-------- ----AEIRDA FDLYDQDKNG ----LISASE LHQVLNRL-- ---GMS---- CSAEECTRMI
        ThC27  ELKKV-FDQF ---------D SNGDGKISVA ELGGVFKAMG ----TSY--T --ETELNRVL EDVDTD-RDG FINVDEFSAL C--RSSS--- ---------- ---------- SA-------- ----SEIRDA FDLYDQDKNG ----LISASE LHQVLNRL-- ---GMS---- CSLEDCSRMI
      BrCML26  DLKKV-FNQF ---------D ANGDGKISVS ELGNVFKSMG ----TSY--T --EAELNQVL DDIDID-RDG FINMEEFAAI C--RSSS--- ---------- ---------- SA-------- ----TEVREA FDLYDQNKNG ----LISSSE IHKVLNRL-- ---GMT---- CSVEDCVRMI
      CrCML26  ELKKV-FDQF ---------D ANGDGKISVS ELGNVFKSMG ----TSY--T --EEELNRVL DDIDID-RDG YINQEEFATI C--RSSS--- ---------- ---------- SA-------- ----AEIREA FDLYDQDKNG ----LISSSE IHKVLNRL-- ---GMS---- CSVEDCVRMI
      AtCML26  ELKKV-FDKF ---------D ANGDGKISVS ELGNVFKSMG ----TSY--T --EEELNRVL DEIDID-CDG FINQEEFATI C--RSSS--- ---------- ---------- SA-------- ----VEIREA FDLYDQNKNG ----LISSSE IHKVLNRL-- ---GMT---- CSVEDCVRMI
      ThCML26  ELKKV-FDQF ---------D SNGDGKISVS ELGNVFKSMG ----TSY--T --ETELNRVL DDIDID-RDG YINLEEFATI C--RSSS--- ---------- ---------- TA-------- ----AEIREA FDLYDQDKNG ----LISSSE IHQVLNRL-- ---GMT---- CSVEDCVRMI
      CpCML27  ELQKV-FDRF ---------D ANGDGKISAT ELRDVLKAMG ----SDY--T --ENEVQRVM EEVDAD-RDG FINFQEFSDL C--RSNS--- ---------- ------C--D SS-------- ----AELRDA FDLYDQDHNG ----LISSSE LHLVLNRL-- ---GIK---- CSVEDCVRMI
      CcCML27  EMQKI-FNQF ---------D ANKDGKISLT ELATVLKSMG ----SSY--K --EEELVRVM EDLDTD-KDG FISLSEFETI C--RTSSGA- ---------- ------A--G EA-------- -----ELREA FDLYDQDKNG ----LISAEE LNLVLNRL-- ---GIK---- CSVDGCVAMI
      CsCML27  EMQKI-FNQF ---------D ANKDGKISLT ELATVLKSMG ----SSY--K --EEELVRVM EDLDTD-KDG FISLSEFETI C--RTSSGA- ---------- ------A--G EA-------- -----ELREA FDLYDQDKNG ----LISAEE LNLVLNRL-- ---GIK---- CSVDGCVAMI
    GrCML27-2  ELQKV-FNQF ---------D ANRDGKISMT ELRDVLKSMG ----SSI--T --EEELKRVL EDIDTD-KDG FINLSEFSSL F--RSSSDE- ---------- ------V--T AA-------- ----LELRDA FDLYDQDKNG ----LISTSE LHLVLNQL-- ---GMK---- CSVDDCARMI
      TcCML27  ELQKV-FNQF ---------D ANKDGKISVS ELGDVLKAMG ----SSY--S --KEELERVM EDIDTD-KDG FINLSEFSAF C--RSSSDA- ---------- ------V--S AA-------- ----SELRDA FDLYDQDKNG ----LISANE LHLVLNRL-- ---GLK---- CSVDDCVRMI
    GrCML27-3  ELKRV-FDQF ---------D ANKDGQISIT ELRDVLKAMG ----SNY--S --EEELKRVM EDIDSD-QDG YINFSEFSTF W--SASSDA- ---------- ------V--N AA-------- ----SELREA FDLYDQNKNG ----LISATE LHQVLNRL-- ---GMT---- CSADDCVGMI
    CsatCML27  ELRKV-FERF ---------D ANGDGKISIS ELDAVLTSLT --LKSAI--P --LEELRSVM DDLDSD-KDG YINIDEFAAF C--KKPMAS- ---------- ------DE-A GA-------- ----AELRDA FDLYDQDRNG ----LISQSE LHLVLNRL-- ---GIS---- CSKEDCQKMI
    GmCML27-5  ELRKV-FSRF ---------D ANGDGKISVS ELDNVLRSLG ----SGV--P --PEELQRVM EDLDTD-HDG FINLSEFAAF C--RSDT-A- ---------- ------D--G GD-------- ----TELHDA FNLYDQDKNG ----LISATE LCQVLNRL-- ---GMK---- CSVEECHNMI
    PvulCML27-  EMKRV-FSRF ---------D TNGDGKISVS ELDNVLRSLG ----SGV--P --PEELQNVM DDLDTD-HDG FINLSEFAAF C--RAET-A- ---------- ------D--G GD-------- ----TELQDA FNLYDQDKNG ----LISATE LCQVLNRL-- ---GMR---- CSIDECHSMI
      MtCML27  ELKTV-FTRF ---------D TNGDGKISVT ELDNILRSLG ----STV--P --KDELQRVM EDLDTD-RDG FINLAEFAAF C--RSGS-A- ---------- ------D--G DV-------- ----SELREA FDLYDKDKNG ----LISATE LCQVLNTL-- ---GMK---- CSVEECHTMI
    GmCML27-3  ELETV-FNRF ---------D ANGDGKISAD ELDSVLRSLG ----SGV--S --PEDLRRFM EDLDTD-RDG FISLTEFAAF C--RSDASA- ---------- ------D--G GS-------- ----GEFRDA FDLYDRDKNG ----LISAAE LHLALNRL-- ---GLK---- CSVDECRDMI
    PvulCML27-  ELERV-FNRF ---------D ANGDGKISTD ELDSVLKTLG ----SGV--S --PEDLRRVM EDLDTD-RDG FISLTEFAAF C--RSDA-A- ---------- ------D--G GD-------- ----GEFHDA FDLYDRDKNG ----LISAEE LHLALNRL-- ---GLK---- CSVDECSDMI
    LuCML27-1  ELRKV-FEKF ---------D TNGDGKISAS ELAGVLRSMG ----TEY--S --PPDLHRAM EEIDSD-KDG FINLEEFAQL C--RTSSSA- ---------- ------G--G AA-------- ----EELREA FDLYDENKDG ----LISEAE LHQVLNRI-- ---GMR---- CSVDECARMI
    LuCML27-2  ELKKV-FSKF ---------D ANGDGKISLA ELADVLRSMG ----SSY--S --PLDLQRAM DDIDTD-KDG FINLEEFAEL C--RSSSGA- ---------- ------S--A AA-------- ----E-LHEA FNLYDEDKDG ----LISSSE LHHVLNRL-- ---GMK---- CSMEECGRMI
    LuCML27-3  ELKKV-FSKF ---------D ANGDGKISLA ELADVLRSMG ----SSY--S --PLDLQRAM DDIDTD-KDG FINLDEFAEL C--RSSSGA- ---------- ------S--A AA-------- ----E-LHEA FNLYDEDKDG ----LISSSE LHHVLNRL-- ---GMK---- CSMEECGRMI
    PtCML27-2  ELRKV-FNQF ---------D TNGDGKISAS ELGEVLKSMG ----STY--T --MEELHRVM EDVDTD-KDG YIDLAEFAKL C--RSSSAA- ---------- ------A--A AS-------- ----E-LRDA FDLYDQNGDG ----MISAAE LHQVLNRL-- ---GMK---- CKVDECFQMI
    PtCML27-3  ELKVV-FDQF ---------D ANGDGKISTS ELGEVLKSTG ----STY--T --TEDLRRVM EDVDTN-KDG HIDLAEFAQL C--RSPSTA- ---------- ------S--A AS-------- ----E-LRDA FDLYDQNGDG ----LISTAE LHQVLSRL-- ---GMK---- CKVGECVKMI
    MeCML27-1  EIQKV-FNQF ---------D TNGDGRISVD ELGGVLQSMG ----SAC--T --SAEIERIM EDVDTD-KDG FISLEEFAQL F--KSSSGA- ---------- ------A--A DS-------- ----E-LKEA FDLYDQNKNG ----LISSAE LHQVLNRL-- ---GMS---- CSLDDCVRMI
    MeCML27-2  ELHKV-FNQF ---------D SNGDGKISVA ELGNVLKSMG ----SSY--T --TVELERVM DEVDTD-KDG FINLQEFAQL C--RSSSGA- ---------- ------A--A DS-------- ----E-LKEA FDLYDQNKNG ----QISSAE LHQVLNRL-- ---GMQ---- CSKEDCERMI
      EgCML26  ELRKV-FDQF ---------D ANGDGKISAS ELSQVLAAMG ----SAV--P --PEDLDRAM AEIDAD-GDG FISLPEFAGL C--RSGPSP- ---------- ------D--- ---------- ----ADLRDA FDLYDQDRNG ----LISAAE LHLVLNRL-- ---SMN---- CSVEDCARMI
    FvCML27-2  EVRRV-FKSF ---------D TNGDGRISVS ELGNVLKALG ----SNV--G --EDELQRVM VELDTD-RDG FICLDEFNAF W--VAGSPD- ---------- ---------G SS-------- ----AELKGA FDLYDQDRNG ----LISAEE LHLVLNRL-- ---QMK---- CSVEDCNRMI
    PperCML27-  EVQRV-FKSF ---------D ANGDGKISVS ELGNVLKALG ----SSV--S --ADELQRVM GDLDTD-RDG FICLDEFNAF W--VSGSKD- ---------- ---------G NA-------- ----AELRDA FDLYDQDRNG ----LISANE LHMVLNQL-- ---QMT---- CSLEDCNRMI
    MdCML27-1  EVQKV-FKSF ---------D ANGDGKISVN ELGNVLKALG ----SNV--S --GDELKRVM VDLDTD-NDG FICLDEFNAF W--VAGSKD- ---------- ---------G GA-------- ----AELKDA FDLYDQDRNG ----LISTNE LHLVLSRL-- ---NMA---- CTVEDCNRMI
    MdCML27-2  EVQKV-FKGF ---------D ASGDGKISVN ELGNDLKALG ----SNV--S --GDKLKRVM VDLDTD-NDG FICLDEFNAF W--VSXSKD- ---------- ---------G GA-------- ----AELKDA FDLYDQDRNG ----LISTNE LHLVLSRL-- ---NLA---- CTVEDCNRMI
    MdCML27-3  EVRKV-FKSF ---------D SNGDGKISVN ELGNVLTALG ----STV--S --EDELKRFM VDLDTD-NDG FICLDEFNAF W--VAGPKD- ---------- ---------G GV-------- ----TELKDA FDLYDQDRNG ----LISANE LHLVLNRL-- ---NMA---- CTIEDCHRMI
    GrCML27-1  PIQKV-FNQF ---------D KNKDGKISVS ELDEVLKALG ----CSS--P --EEEIKRVM EELDVD-KDG FINLSEFINL C--SSSFDT- ---------- ------A--N AE-------- ----TELHEA FDLYDQDKNG ----LISAEE LHLVLNRL-- ---GMT---- CSVEKAVKMV
    MgCML27-1  EVRKV-FQRF ---------D TNGDGKISSD ELGGVLNALG ----SGT--S --ADEVARMM EEIDTD-KDG HINLQEFAAF C--SGDSDP- ---------- -------Y-N AE-------- ----KELREA FQLYDQDHNG ----KISAAE LHQILTRL-- ---GEH---- CSEQDCAGMI
    SlCML27-2  EVQKV-FQRF ---------D TNGDGMISGE ELSGVISALG ----SGT--S --PEEVSRMM NEIDSD-RDG FISLQEFAEF C--KGDINS- ---------- -------IGD GV-------- ----RELREA FDLYDQDSNG ----LISAAE LHQILTRL-- ---GEN---- CSVQDCTKMI
    SlCML27-1  EVKKV-FARF ---------D ANGDGMISGD ELSGVLKALG ----SDT--S --PEEVARMM AEIDTD-KDG CINLDEFADF C--KNDESV- ---------- --------DG GA-------- ----KELKEA FDLYDQDHNG ----RISATE LHQILNRL-- ---GQN---- CTLQDCTRMI
      EgCML23  QVRQV-FAQY ---------D KNGDGKI--- ----SCDELR ---------- ---EEAQAAM AEIDKD-GDG FINLDEFAEL I--LGGGDG- ---------- ------GPEC GA-------- ----KELRDA FDLYDLDGNG ----VISVSE LHAVMKKL-- ---GQK---- CSLRDCRKMI
    EgCML24-2  QVRQV-FAQY ---------N ISCDELCEVL YVLGSSPTTR ---------- ---EEARAAM SEIDKD-GDG FINLDEFAEL I--LGGSDR- ---------- ------GPEC RA-------- ----KELRDA FDLYDLDGNG ----VILASE LPAVLKKL-- ---GQK---- CNRRYCRKMI
      BdCML16  EMKKL-FSRF ---------D ADGDGRISPS ELAAVSRAIA --PPPSESAG --GREVASMM DQLDAD-RDG FVDLGEFAAF H--SHTDGR- ---------- ------E--E EE-------- ---ERELRDA FAVYDIDGDG ----RISVAE LAKVLARI-- ---GEG---- CSTEECQRMI
      OsCML16  EIERV-FTRF ---------D ADGDGRISPS ELAAVTRAIA --PPPSESAG --GREVAAMM NELDTD-RDG FVDLGEFAAF H--GRGRG-- ---------- ------D--A EH-------- ----EAELRAA FDVYDVDGDG ----RITAAE LGKVLGRI-- ---GEG---- CSAEECERMI
      PhCML16  EMQKV-FSRF ---------D ADGDGRISPS ELAAVSRAIS --PPPSSSHG --GREVAAMM DELDAD-RDG YADLGEFAAF H--ARA-GR- ---------- ------G--D DG-------- -ELEAELRAA FDVYDVNGDG ----RITAAE LGKVLAQI-- ---GEG---- CGAEECERMI
      PvCML16  EMQKV-FSRF ---------D ADGDGRISPS ELAAVSRAIS --PPPSSSHG --GREVAAMM DELDAD-RDG YVDLGEFAAF H--ARA-AG- ---------- ------R--G DG-------- -ELEAELRAA FDVYDVDGDG ----RITAAE LGKVLAQI-- ---GEG---- CSAEECERMI
      SiCML16  EMQKV-FSRF ---------D ADGDGRISPS ELAAVNRAIS --PPPTSSHG --GREVAAMM DELDTD-RDG YVDLGEFAAF H--ARACAR- ---------- ------G--D DG-------- -ELEAELRAA FDVYDVNGDG ----RITAAE LGRVLAQI-- ---GEG---- CSAEECERMI
      SbCML16  EMQRV-FARI ---------D ADGDGRISPS ELAAVSRAIS --PPSSSSHG --RREVAAMM DELDTD-RDG FVDLGEFKAF H--ARARAG- ---------- ------G--G RGGDNGGSGG DELDAELRAA FDVYDVDGDG ----RITAAE LGKVLGRI-- ---GEG---- CSAEECQRMI
      ZmCML16  EMQRV-FSRI ---------D ADGDGRISPS ELAAVSRAIS --PPASSSHG --RREVAAMM DELDTD-RDG FVDLGEFRAF H--ARGVGG- ---------- ------G--G DDGD------ ---DAELRAA FAVYDADGDG ----RITAAE LGSVLARI-- ---GEG---- CSAEECRRMI
      PhCML18  EMERV-FRRY ---------D ANGDGKISAE ELASVLRALG --APP----G --PGEVRRMM DEMDAD-RDG FVDLAEFVAF H--C--GGA- ---------- ------G--S ARL-EGED-D DATEAELREA FRMYDADRNG ----LISARE LHRVLRQL-- ---GDK---- CSVADCSRMI
    PvCML18-2  EVEQV-FRRY ---------D ANGDGKISAE ELASVLRALG --APP----G --PGEVRRMM DEMDTD-RDG FVDLAEFVAF H--C--AAR- ---------- ------P--E G---EDED-G GAAEAELREA FRMYDADRNG ----LISARE LHRVLRQL-- ---GDK---- CSVADCSRMI
    PvCML18-1  EVEQV-FRRY ---------D ANGDGKISAE ELASVLRALG --APP----G --PGEVRRMM DEMDAD-RDG FVDLAEFVAF H--C--GAQ- ---------- ------P--- A---EGQD-D GAAEAELREA FRMYDADRNG ----LISARE LHRVLRQL-- ---GDK---- CSVADCSRMI
      SbCML18  EVEQV-FRRY ---------D ANGDGKISAE ELASVLRALG --APP----G --PGEVRRMM DEMDSD-RDG FVDLAEFIAF H--CSNGEE- ---------- ------E--E GAE-EGEGRE DATEADLREA FRMYDADRNG ----LISARE LHRVLRQL-- ---GDK---- CSVADCSRMI
      SiCML18  EVEQV-FRRY ---------D ANGDGKISAE ELASVLRALG --APP----G --PGEVRRMM DEMDAD-RDG FVDLAEFVAF H--C---GP- ---------- ------S--G GAG-AGEGQE DATEAELREA FRMYDADHNG ----LISARE LHRVLRQL-- ---GDK---- CSVADCSRMI
      OsCML18  ELEQV-FRRY ---------D ANGDGKISAE ELASVLRALG --APL----G --PGEVRRMM DEMDSD-RDG FVDLSEFAAF H--CGPTPA- ---------- ------H--G GKGGDAKDQE AASEAELREA FRMYDADSNG ----KISARE LHRVLRQL-- ---GDK---- CSVADCSRMI
      AcCML30  EMKKV-FDKF ---------D ADKDGKISKE EYLTVLRALG ---KQNLD-R ----EVNQVF EVADTN-GDG FINFKEFMDL H--EKGG-G- ---------- -------IKQ ---------- ----MDIRNA FRTFDLDGNG ----RISAEN LLEVMKRL-- ---GES---- CTLEACKKMV
    AcCML30-2  EMKRV-FDKF ---------D ANKDGKISKE EYRSVMRALG ---KQNLE-V ----EVTKAF EVTDLN-GDG FIDFKEFMDL H--EKGG-G- ---------- -------IKP ---------- ----MDIRNA FRTFDLDGNG ----KISAEE LLEVLRRL-- ---GER---- CNLQTCKKMV
      BrCML30  EMRRV-FNRF ---------D SDKDGKISQT EYKVVLRALG ---QERAI-Q ----DVPKIF KAADLD-GDG FIDFKEFIDA H--KRSG-G- ---------- -------IRS ---------- ----LDIRNA FWTFDLNGDG ----KISAEE VMSVLRKL-- ---GER---- CNLEDCKRMV
      ThCML30  EMRRV-FNRF ---------D WDKDGKISQT EYKVVLRALG ---QEKAI-E ----DVPKIF KAVDLD-GDG FIDFREFIEA H--KRSG-G- ---------- -------VRS ---------- ----SDIRNA FWTFDLNGDG ----KISAEE VMSVLRKL-- ---GER---- CSLEDCKRMV
      CrCML30  EMRRV-FNRF ---------D SDSDGKISQT EYKVVLRALG ---QERAI-E ----DVPKIF KAVDLD-GDG FIDFREFIDA Y--KRSG-G- ---------- -------IRS ---------- ----SDIRNA FWTFDLNGDG ----KISAEE VMSVLRKL-- ---GER---- CSLEDCKRMV
      AtCML30  EMRRV-FSRF ---------D LDKDGKISQT EYKVVLRALG ---QERAI-E ----DVPKIF KAVDLD-GDG FIDFREFIDA Y--KRSG-G- ---------- -------IRS ---------- ----SDIRNS FWTFDLNGDG ----KISAEE VMSVLWKL-- ---GER---- CSLEDCNRMV
    LuCML30-1  EMRQV-FNKF ---------D SNGDGKISRH EYKAILRALG ---QQSMV-A ----DVQHIF KVADLD-GDG FIDFKEFVEA Q--RKEGAG- ---------- -------VRT ---------- ----MDIQSA FRTFDVNGDG ----RISAEE LLQVLRQL-- ---GER---- CTLDDCRRMV
    LuCML30-2  EMRQV-FNKF ---------D SNGDGKISRH EYRAILRALG ---QQSMV-A ----DVQHIF KVADLD-GDG FIDFNEFVEA Q--KKEGAG- ---------- -------VRM ---------- ----MDIQSA FRTFDVNGDG ----RISAEE LLQVLRQL-- ---GER---- CTLDDCRKMV
    CcCML30-2  EMKRV-FDRF ---------D SNKDGKISQM EYKAILRAIG ---QASMI-S ----EVPKIF QVVDLD-GDG FIDFKEFMEA H--KKGG-G- ---------- -------IRT ---------- ----MDIQSA FRTFDKNDDG ----KISAEE ILEMLRRL-- ---GES---- CSLEDCRKMV
    CsCML30-1  EMKRV-FDRF ---------D SNKDGKISQM EYKAILRAIG ---QASMI-S ----EVPKIF QVVDLD-GDG FIDFKEFMEA H--KKGG-G- ---------- -------IRT ---------- ----MDIQSA FRTFDKNDDG ----KISAEE ILEMLRRL-- ---GES---- CSLEDCRKMV
    MeCML30-1  EMRQV-FNKF ---------D SNKDGKISQQ EYKATLRALG ---QENMI-G ----EVPKFF QVVDLD-GDG FIDFREFVEA Q--KKGG-G- ---------- -------IMM ---------- ----KDIQSA FQAFDMNGDG ----KITAEE VMEVLRRL-- ---GER---- CSLEDCRRMV
    PtCML30-1  EMRQV-FDKF ---------D SNKDGKISQQ EYKDTLRALG ---QGNML-G ----EVPKIF QVVDLD-GDG FIDFKEFVEA Q--KKGG-G- ---------- -------IRT ---------- ----TDIQTA FQTFDSNGDG ----KISAEE VMEVLRRL-- ---GER---- CSLEDCRRMV
    MdCML30-3  EMKKV-FEKF ---------D SNKDGKISQA EYKAILRALG ---KGNMI-G ----EVPKIF KFVDLD-EDG FINFKEFMEV H--KKGG--- ---------- -------IRT ---------- ----MDIQNA FRMFDLNGDG ----KISAEE VMEVLRGL-- ---EER---- CNLEDCRRMV
    PperCML30-  EMKRV-FDKF ---------D SNKDGKISQP EYKAILRALG ---KGNMV-G ----EVPKIF RIADLD-GDG FINFKEFMEV H--KKGG-G- ---------- -------IRT ---------- ----IDIQNA FRTFDLNGDG ----KISAEE VMEVLGRL-- ---GES---- CSLEDCQRMV
    MdCML30-4  EMKKV-FDKF ---------D SNKDGKISPA EYKAILRALG ---KGSKV-G ----EVQKIF KIVDLD-RDG FINFKEFLDV H--N--G-G- ---------- -------IRT ---------- ----IDIQNA FQMFDLNGDG ----KISAEE VMEVLTGL-- ---EES---- CNLDDCRRIV
       VvCM30  ELKKV-FDKF ---------D SNKDGKISEE EYKAVLGALV ---KEGVR-T ----EVEKIF QVADLD-GDG FIDFKEFVEV H--KKGG-G- ---------- -------VKT ---------- ----RDIQSA FRAFDLNRDG ----KINAEE LLEVLGRL-- ---GER---- CSLEECRRMV
    GmCML30-1  EMKQV-FDKF ---------D SNKDGKISQQ EYKATMKALG ---MGDSV-H ----EVPNIF RVVDLN-GDG FINFKEFMEA Q--SKGG-G- ---------- -------VRM ---------- ----MDIQSA FRTFDKNGDG ----RISAEE VKEMLGKL-- ---GER---- CSIEDSRRMV
    PvulCML30-  EMKRV-FDKF ---------D SNKDGKISQQ EYKVTMKALG ---LGDSV-H ----EVPDIF KVVDLD-GDG FINFKEFMEA Q--NKGG-G- ---------- -------VRA ---------- ----MDINSA FRTFDRNGDG ----KISAEE IREMLARL-- ---GER---- CSIDDCRRMV
    MeCML30-3  EMRQI-FNKF ---------D TNKDGKISQQ EYKETLRALG ---LEKMT-E ----EVPKIF QVVDSD-GDG FIDFTEFVEA Q--KKGG-S- ---------- -------INI ---------- ----TDIQSA FQAFDLNGDG ----KITAEE VMEVLRCL-- ---GER---- CSPEDCRRMV
    CsatCML30-  EIKQI-FNKF ---------D TNKDGRISKH EYRGILKALG ---RGNSM-E ----EVQKIF RAVDSD-GDG YINLNEFMEV H--RSGG-G- ---------- -------VQA ---------- ----KEVEFA FKTFDLNGDR ----KISAEE VMRVLKGL-- ---GEK---- CSIEDCRRMV
      MgCML30  ELKQV-FNKF ---------D SNKDGKISPE EYKAVLKALG ---KGNLLTK ----EVQNIF EVADLD-GDG FIDFNEFVEV Q--RKGG-G- ---------- -------VKA ---------- ----ADLQRA FQAFDKDNDG ----KITVEE VHELLKKL-- ---GER---- CSLRDCQKMV
    SlCML30-2  ELKKV-FDRF ---------D SNKDGKISPE EYKAILKAMG ---KKNLLTR ----EVQKIF DVADAD-GDG FIDFNEFVAV Q--KKEG-G- ---------- -------VKT ---------- ----TDLQSA FHTFDKDGDG ----RISVQE VYELQKGL-- ---GQR---- CSLQDCRKMV
    StCML30-1  DLKKV-FDRF ---------D SNKDGNISPE EYKAILKAMG ---KKNLLTR ----EVQKIF DVADAD-GDG FIDFNEFVEV Q--KKEG-G- ---------- -------VKT ---------- ----TDLQRA FHTFDKDGDG ----KISVQE VYELQKGL-- ---GQR---- CSLQDCRKMV
    EgCML30-1  EIRRV-FDKY ---------D ANGDGKITKE EYKLAIGALG ---QGISD-H ----EATEAF SVMDAD-KDG FVDFKEFLEV H--HGPGGG- ---------- -------IKS ---------- ----CDIEGT FRLFDLDGDG ----QISAEE LWEVMTSV-- ---G------ --LEDCRRMV
    EgCML30-2   MKRV-FDKF ---------D SNKDGKISQQ EYKAVLRAIS ---KENLI-E ----EVPQIF RVADLD-GDG FIDFKEFMEA Q--KKEG-G- ---------- -------IKT ---------- ----KDIQSA FYTFDLNGDG ----KITADE VLKVLGSL-- ---GER---- CSIEDCRKMV
    CcCML30-1  EMRQV-FDKF ---------D TNKDGKISQD EYNSALNVL- ---GKGI-SK --A-EMAKSF RFIDTD-KDG YIDFKEFIEM M--HDMGDN- ---------- ------RVKK ---------- ----NDIQGA FQLFDLNGDK ----KISAEE LMEVLRKM-- ---GEK---- YSLDSCRKMI
    CsCML30-2  EMRQV-FDKF ---------D TNKDGKISQD EYNSALSVL- ---GKGI-SK --A-EMAKSF RFIDTD-KDG YIDFKEFIEM M--HDMGDN- ---------- ------RVKK ---------- ----NDIQGA FQLFDLNGDK ----KISAEE LMEVLRKM-- ---GEK---- YSLDSCRKMI
    GrCML30-1  EMKWV-FDKF ---------D TNKDGKISKE EYKSALKVL- ---GKGM-TG --A-EVTKAF TAIDTD-GDG FIDYKEFMEM M--QNMGEG- ---------- -------INV ---------- ----NDIQSA FRVYDLDGNG ----KISAEE LMAVLKKM-- ---GER---- CNLETCRKMI
      TcCML30  EMKWV-FDKF ---------D TNRDGKISKE EYKSALKVL- ---GKGM-AE --T-EVAKAF SAIDTD-GDG FIDFKEFIEM M--RNMGEG- ---------- -------INS ---------- ----NDIQSA FRVYDLDGNG ----KISAEE LMEVLKKM-- ---GER---- CSLEACRKMI
    GrCML30-2  EMKWV-FNKF ---------D TNKDGKISKQ EYKSAMKMM- ---GKGI-TD --T-EMVKAF TTIDTD-GDG FIDYKEFIHM M--HNMEEG- ---------- -------FNN ---------- ----NDIKTA FRVYDLNGDG ----KISAEE LMAVMKNI-- ---GER---- CSLEACRKMI
    MeCML30-2  EMKWV-FSKF ---------D TNKDGKISRQ EYKSALRSL- ---GKGM-NE --E-EMAKSF EATDID-GDG FIDFEEFMVM M--NNMGDG- ---------- -------VKA ---------- ----SEIQSA FQAFDLDGNG ----KISAEE LMQVLKKM-- ---GER---- SSLDACRKMI
    PtCML30-2  EMKWV-FDKF ---------D LNKDGKISRQ EYKSALRAL- ---GKGL-EE --S-EMVKAF QATDID-GDG YIDFKEFMEM M--HNMGDG- ---------- -------VKS ---------- ----SDIESA FRVFDLDGNG ----KISAEE LMEVLKRL-- ---GER---- SSLDACRKMI
    GmCML30-2  EMKLV-FDKF ---------D TNKDGKITLE EYKAAMRTM- ---GWGI-EG --T-EADESF QVMDSD-GDG FIDFKEFMDM F---NVEE-- ---------- ------TVKE ---------- ----TEIKSA FQVFDLNGDG ----KISAEE LSQVLKSL-- ---GES---- CSLSACKKMV
    PvulCML30-  EMKWV-FDRF ---------D TNKDGKISLE EYKAAVRTM- ---GWGI-GG --S-EAVESF QVMDSD-GDG SIDFKEFMEM F---NVEE-- ---------- ------RVKE ---------- ----TEIKSA FQVFDLNGDG ----KISAEE LSHVLKSL-- ---GES---- CSLNACKKMV
    MtCML30-2  EMKWV-FEKF ---------D KNKDGKISLE EYKAAAKAL- ---DKGIICD --N-DAVKAF KAMDSD-KDG FIDFKEFMEM F---NGEGS- ---------- ------KIKE ---------- ----EDIKSA FQVFDINGDG ----KISAEE LSQIFKRL-- ---GES---- CSLSACKKMV
    MtCML30-3  EMKWV-FEKF ---------D KNKDGKISLE EYKAAAKAL- ---DKGIICD --N-DAVKAF KAMDSD-KDG FIDFKEFMEM F---NGEGS- ---------- ------KIKE ---------- ----EDIKSA FQVFDINGDG ----KISAEE LSQIFKRL-- ---GES---- CSLSACKKMV
    MtCML30-1  EMKWV-FEKF ---------D TNKDGKISLE EYKAAAKSL- ---DKGI-GD --P-DAVKAF NVMDSD-KDG FIDFKEFMEM F---NGENN- ---------- ------KIKE ---------- ----EEIKSA FQVFDINGDG ----KISAEE LSQIFKRL-- ---GES---- CSLSACKKMV
    PvulCML30-  EMKWV-FQKF ---------D TNKDGKISLE EYKAAARAL- ---DRGI-GE --A-EAVKAF RVMDSD-GDG FIDFREFVEM L---NGEG-- ---------- ------RIKE ---------- ----ADIKNA FQVFDLNGDG ----KISAQE LSQVLKRL-- ---GES---- CSLSACKKMV
    MdCML30-5  EVRYV-FDKF ---------D TNKDGRISRE EYKSALKTL- ---DKDI-AE --G-EIAKTF KALDSD-GDG FVGFTEFVEM F---NMGND- ---------- ------EARK ---------- ----ADIESA FRVFDLDGNG ----KISXEE LXLVLKKL-- ---GEN---- CSLGACRNMV
    PperCML30-  EIRYV-FDKF ---------D TNKDGRISRE EYKSALKTL- ---DKEI-AE --A-EIAKTF KALDSD-GDG FIGFKEFAEM F---NMGAD- ---------- ------QARE ---------- ----ADIESA FRLFDLDGNG ----KISAEE LSLVLKKL-- ---GEN---- CSLGACRNMV
    CsatCML30-  EIKWA-FEKF ---------D SNKDGKISFE EYKEAHRGLA ---GSKEITD --A-EAEKSF KLVDVD-GDG FVDLKEFVEL Y---TMSSG- ---------- ------EVKV ---------- ----GDIESA FKVYDSNGDG ----KISAEE VMGIMKIL-- ---GEN---- TTLKACKQMV
    SlCML30-1  EMKRV-FDKF ---------D TNKDGKISKE EYKSAMKMMS ---SGGGNTK --SYEVDDAF QAADTN-GDG FINFDEFMRV ----QSLEG- ---------- ------GVNS ---------- ----TDIKSA FKAFDLDGDG ----KISAEE LLQVQRML-- ---GEK---- CSLESCKKMV
    StCML30-2  EMKRV-FDKF ---------D TNKDGKISKE EYKLAMKMMS ---SGGGNTK --SYEVDDAF QAADTN-GDG FINFEEFMRV ----QSLEG- ---------- ------GVNS ---------- ----TDIKSA FKAFDLDGDG ----KISAEE LLQVQRML-- ---GEK---- CSLESCKKMV
      BdCML29  EMKKV-FNKI ---------A GEP-GRISRS DLRALLQRFE ---KTDPVG- ----DARRMV CAADNN-KDG YIDLEEFMEV H--RSG---- ---------- -------VQV ---------- ----RDIRRA FFVFDRDRDG ----KISAEE VMDVLQKL-- ---GER---- CSLEECRRMV
       PhCML6  EMKKV-FHTI ---------A RDPRG-INKK DLQLLLERLG ---KADAAG- ----EARRMM CVADHN-KDG YMDLEEFMEV H--RNG---- ---------- -------VQL ---------- ----GDIRRA FFVFDRNEDG ----RISAEE IMTVLRKL-- ---GES---- CSLEDCRKMV
       SbCML1  EMKKV-FSMI ---------A GQSHGKISKK DLQLLLERFG ---KADAAA- ----EARRMM CVADHN-KDG YMDLEEFMEV H--RNG---- ---------- -------VQL ---------- ----GDIRRA FFVFDRNGDG ----RISAEE VMTVLCNL-- ---GQS---- CSLDDCRKMV
      BdCML20  ALAGV-FRRY ---------D GDGDGQISSA EMRELCGC-- ---------T --DAEAEEMV AAADAD-GDG LVSIEELGAL M--VELETE- ---------- ------GGEP SS-------- ----DALQEA FAEYDEDGDG ----VITAEE LGRALRRVLV GQGEEE---- LTAEQCSRMV
      PhCML20  ELVRV-FRRF ---------D ADGDGRISAA EMRESCGC-- ---------T --AAEAEEMV AAADRD-GDG FISLEELVAL F-------E- ---------- ------DGD- RS-------- ----DALRAA FAEYDEDGDG ----GITAEE LRRALRRL-- GLCGEE---- MTAERCAEIV
      SbCML20  ELARV-FRHL ---------D ANGDGRISAA EMRESCGC-- ---------T --AAEAEDMV AAADRD-GDG FISLEELVAL L-------E- ---------- ------DGD- KA-------- ----DTLRAA FAEYDENGDG ----VITAEE LRRALRRL-- GIVGEE---- MSAERCAEIV
    ZmCML20-2  ELVRV-FRHF ---------D ADGDGRISVA EMRESCGC-- ---------T --AAEAEEMV AAADRD-GDG FISLDELGAL F-------E- ---------- ------DED- LS-------- ----DTLRAA FAEYDENGDG ----VITAEE LLRALRRL-- GIVGEE---- MTAERCAEII
    ZmCML20-1  ALVRV-FRHF ---------D ADGDGRISAD EMRELCGC-- ---------T --AVEADEMV AVADRD-GDG FISLEELEAL F-------E- ---------- ------DGD- RS-------- ----DTLRAA FAEYDENGDG ----VITAEE LRRALWRL-- GIVGEE---- MTAERCAEMI
      SiCML20  ELVSV-FRRF ---------D ADGDGRISAD EMRESCGC-- ---------T --AEEAEEMV AAADRD-GDG FISLEELGAL L-------D- ---------- ------GGD- QS-------- ----VDLRNA FAEYDEDGDG ----VITAEE LRRALRRL-- G---EE---- VTAERCAEMV
      OsCML20  ELIRV-FRCF ---------D TDGDGLISAA EMREFYGC-- ---------S --VDEAEEMV AAADRD-GDG FVSIEELRAV M-------E- ---------- ------GGG- L--------- ----DALRAA FDEYDEDGNG ----VITAEE LRRALRRL-- NLDGMD---- LTAEQCAEIV
    AcCML25-5  LSLMETFRAF ---------D ADNDGSISTA ELGGILSSLG ------HTVS --QEDLKVMM EQGDRN-GDG LLSIEDFLEM T--TDKIELG G--------- ---------- ---------- --LTDSLTIA FETLDANGDE ----AVTGED LYEILKN--- --TGIG---- LSKEACQDII
      CcCML25  LSLVETFRAF ---------D SDNDGSITGA ELSGIFNSVG ------YRAS --EEDVREMM RQGDAN-NDG LLSLEEFLEM N--TKDMELG S--------- ---------- ---------- --LGNLLKAA FESLNLDGDD ----ILTGAE LHEVIEN--- --LGVG---- LSLADCQEII
    CsCML25-2  LSLVETFRAF ---------D SDNDGSITGA ELSGIFNSVG ------YRAS --EEDVREMM RQGDTN-NDG LLSLEEFLEM N--TKDMELG S--------- ---------- ---------- --LGNLLKAA FESLNLDGDD ----ILTGAE LHEVIEN--- --LGVG---- LSLADCQEII
      GmCML25  LALVEAFRAF ---------D ADNDGRITQA ELGGILGSLG ------YNPS --EQEVRAMI EHGDKN-KDG LLSIHEFLEM N--TKDLEGG N--------- ---------- ---------- --LANTLSTA FEALDEDGNE ----ILTGEE LHEVMQN--- --LGLD---- LSLENCVHLV
    PvulCML25-  LSLVEAFRAF ---------D VDNDGRITKA ELGGIFGSLG ------YNAS --EQEVSAMM QEGDKN-KDG LLSIHEFLEM N--TRDLEGG S--------- ---------- ---------- --LASTLSTA FEALDEDGNE ----ILSGED LHGVMQN--- --LGLD---- LSLENCLHLV
    MtCML25-2  LSLIEAFKAF ---------D GDNDGSINEA ELGGIMGSLG ------YKAS --EQEVRAMM QQGDKN-KDG LLCISEFLEM N--TKGLETG N--------- ---------- ---------- --LANVLSAA FEALDEDGNE ----ILTGEE LHEAMGN--- --FGLA---- LSLEKCENVV
    MdCML25-2  LGLVETFRAF ---------D SDNDGKITAA ELGGILGSLG ------YNPS --EEDVRAXM QQGDTN-NDG FLSIEEFLEM N--TKNMEFG G--------- ---------- ---------- --LENVLKNA FEALEVDGDE ----VVTAEE LYEVMGD--- -DLGFE---- LTLEDCQGII
    PperCML25-  LGLVEAFKAF ---------D ADNDGKINAA ELGGILGSLG ------YQAS --EQDVRAMM QQGDTN-RDG FLSIEEFLGM N--TKNLEFG G--------- ---------- ---------- --LENVLKNA FEALDVDGDE ----VVTAEE LYEVVGD--- -DLGYE---- LSLEDCQGII
    TcCML25-2  LGLVEAFRAF ---------D ADNDGAINAA ELGGILGSLG ------YNAS --EQDVTAMM QQGDTN-KDG LLSIEEFLEM N--TKDMELG D--------- ---------- ---------- --LASFLRSA FQAFDVDGEE ----AVTATE LYQILGN--- --LGVE---- LSLEECQGVI
    PtCML25-3  LSLMEAFRAF ---------D SDNDGFITAA ELGGILGSLG ------YNAS --EQDVSAMM QQGDTN-KDG LLSMQEFLEM N--TKDMELG E--------- ---------- ---------- --LANLLQTA FDALDVDVDG ----IVTAEE LYEATVN--- -G-GLD---- LSLEDCQGII
    CsatCML25  FNLIEAFRAF ---------D ADNDGLISSA EVGGIMGSLG ------YNLS --EEDVNMMM EEGDAD-KDG LLSMGEFLEM N--AKNMDVG E--------- ---------- ---------- --LGSYLKIA LEALKADEDD ----LVSGEE LYDVFVN--- --LGLD---- VSLEDSMAIV
    AcCML25-4  NQLKQVFKII ---------D ANGDGKISSL ELGEVLLCLG --HE-KTTVA ---KEAEGMV SVVDYNGDG- FIDLDEFMEV V-------GG GNGE------ --NESNKD-- ---------- ----DGLLDA FHVFDSDKNG ----FISAKE LMNVFIS--- --LGYD---N CTLRECSLMI
    CpCML25-2  SQFEQVFKVF ---------D ANGDGKISAL ELTEVLLCLG --YE-KSTAD ---DEAKAMM REVDSNGDG- FIDLNEFMVA M----NPVNG NSVN------ --GNGNEY-- ---------- ----DHLMDA FMIFDADKNG ----LISAGE LRRVLLS--- --LGSK---N CSISECKKMI
    GrCML25-2  NQFKQVFKVM ---------D ANGDGKISSL ELRQVLLRLG --HE-KSTAS ---KAAEGMI REMDFNGDG- FINLDEFMHA V--VNTTSDG AISS------ --CSSGED-- ---------- ----DHLMDA FLIFDKDKNG ----FISAKE LRNVLIS--- --LGFN---K CSLKQCKRMI
    TcCML25-1  GQFRQVFKVI ---------D ANGDGKISSL ELREVLLCLG --HE-KSTAF ---KAAEGMI REMDFNGDG- FIDLDEFMHA V-----NTEG AI-------- --CSNSEE-- ---------- ----AYLMDA FHIFDTDKNG ----LISAKE LSKVLIS--- --LGFR---K CSLQECKRMI
    PtCML27-1  NQFKQVFKVI ---------D ANGDGKISCH ELSEVLLCLG --YE-KSKAA ---WEAERMV REMDCNGDG- FIDLDEFINA V-----NDDG NFG------- ---SGNKE-- ---------- ----DYLMDV FLIFDTDKNG ----LISARE LQTVLTS--- --LGCK---K CSLEDCRRMI
    MtCML25-1  NQFKQVFKLI ---------D TNGDGKISTS ELSELLSCLG --CK-DSIAA ---KEAEGMV NVLDSNGDG- FVDLEEFMVV M--DDKEGKF GCAN------ --DKEQDE-- ---------- -----YLMDA FHVFDTDKNG ----LISAKE LKRVLIN--- --LGFD---H CSIGECKRMI
    PvulCML25-  TQFHQVFKLI ---------D SNGDGKISTT ELSEVLACLG --YS-KCTAA ---KEAEGMV RVLDFNGDG- FVDLDEFMVV M--NGMEKEE EKEE------ --EEEEEDKI ---------- --DGYLMDA FLVFDTDKNG ----LISAKE LQRVLFN--- --LGCE---N CSLRECKRMI
      RcCML25  NQFKQVFKLI ---------D ANGDGKISSC ELSELLLCLG --FD-KSKAT ---SEAEGMV REMDCNGDG- FVDMDEFIYT L--NDDGKLR GVGG------ --DNKK---- ---------- ----DYLMDA FLIFDADKNG ----LISAEE LKKVLTN--- --LGCD---N CSLKKCRRMI
      FvCML25  NQFKQVFEVM ---------D ANGDGKISPV ELSEVLCCLG --YKNKSAAA ---KEAEGMV REMDCNGDG- FIDLDEFMTA V--SANDTKR SGND------ --GGDD---- ---------- -------LMDA FLIFDTDKNG ----KISAKE LRRVLVS--- --LGCE---R ISLKECKQMI
    PperCML25-  NQFKQVFEVM ---------D SNGDGKISPL ELSEVLSCLG --YK-KSIAT ---KEAEGIV REMDCNGDG- FIDLEEFMNA V--NI-DSGC GNEE------ --EDSE---- ---------- -------LMDA FLIFDTDKNG ----KISAKE LQRVLVS--- --LGCQ---R CSLRECRQMI
    MdCML25-1  NQFKQVFKVM ---------D ANGDGKISPL ELSDMLSCLG --YK-KSIAT ---MEAEGIS REMDFNGDG- FLDLDEFITA V--------- --KE------ --EECE---- ---------- -------LMDA FRIFDADKNR ----KISAEE LQKVLVS--- --LGCQ---K CSLKECRKMI
    CcCML23-2  SQFRQIFKVM ---------D SNGDGKLSSS ELGEVLICLG --CD-KSNAT ---KEAEGML KQMDYNGDG- FIDVDEFMDA V--HDDSGGK PKED------ ---------- ---------- -----YLMDA FLIFDINKNG ----LISAME LRRVLIN--- --LGCD---K CTLEDCRRMI
    CsCML23-2  SQFRQIFKVM ---------D SNGDGKLSSS ELGEVLICLG --CD-KSNAT ---KEAEGML KQMDYNGDG- FIDVDEFMDA V--HDDSGGK PKED------ ---------- ---------- -----YLMDA FLIFDINKNG ----LISAME LRRVLIN--- --LGCD---K CTLEDCRRMI
    MgCML25-2  TQLKQVFKII ---------D ANGDGKLSPF ELKKVLLIST --GHEKAEAA KLEAEAEGIV REMDFNGDG- YVDVDEFVIT V--MGMNGED LMGS------ --EI------ ---------- -----IMRSA FRVFDADNNG ----LISAEE LRSVIRR--- --VGFG---N SSLRECRKMI
    StCML25-2  NQLKLVFKLI ---------D TNGDGKISPL ELTEILLSVG --HHQELKAA --EELAEVMV KEMDCDGDG- FVDVNEF-LN V--MGVEKDE AKDI------ --DE------ ---------- -----IIRQV FLVFDVDNNG ----LISAKE LRRVLIS--- --LGCG---N SSVKECRRMI
    CsatCML36  NHFQLLFNLL ---------D YDGDGKISTK ELSQFLYRLG --YK-KLKAT ---MEAEEMV KEMDSDRDG- FIEMDEFLEV L--QKGEEEE EI-------- ---------- ---------- ------LREA FLIFDGDKNG ----LICAEK LKRTLSK--- --FGWQ---K CDLKQCKKMI
      PpCML23  KELTGIFKYF ---------D KNGDGRISAA ELGHVLRAL- --GIRSSD-- ---EELEAMV REVDCDNDG- FIDLDEFARL Y--KLTQEAT SDEE------ ----SEH--- ---------- ----KTLEAA FDVFDLNKDG ----FISATE LHRVLSD--- --LGEV---- LTEDDCRTMI
    PpCML25-3  QELARVFKVY ---------D ADHDGKISLV ELRAVLTTLG --GA-ISE-- ---EEGVQLM KDIDTNNDG- FISLAEFVAF H--VSIKGGI VGGD------ --ISSVD--- ---------- ----DPLRDA FQVFDKDGDK ----RISADD LQSVLVS--- --LGDK---G HSLEDCRQMI
    PpCML25-8  QEMEKAFKVY ---------D ADKDGRISLA ELSSVLTSLC --GA-ISE-- ---QEIVQIM EEVDTDNDG- FISLAEFVAF H--TSSKPGV LNGE------ --ISPDM--- ---------- ----DPMRDA FQMFDKDGDS ----RISANE LQSVLVS--- --LGDK---G HSIEECRQMI
    PpCML25-9  QEMEKAFKVY ---------D ADKDGRISLA ELSSVLTSLC --GA-ISE-- ---QEIVQIM EEVDTDNDG- FISLAEFVAF H--TSSKPGV LNGE------ --ISPDM--- ---------- ----DPMRDA FQMFDKDGDS ----RISANE LQSVLVS--- --LGDK---G HSIEECRQMI
    PpCML25-6  AELEAAFKVF ---------D ANGDGRICIT ELGKVLGSLS --GCEVTE-- ---QELQLIM NDVDKNQDG- FISLEQFKAA N--KTLTSHL ADNE------ ---TPKE--- ---------- ----DSIREA FATFDKDGNN ----LISADE LRAVMQS--- --LGDK---G YSLEDCRRMI
    PpCML25-1  RELSDAFDFF ---------D LNRDGKLSVE EIATVVRSFG --EE----VT --QEDIQMLI TRVDSDGDG- SLDLCEFIDL N--TRAMSTS TSLD------ -SQFSTENAE ---------- ---DDALLAT FNRFDINKDG ----FISADE LHRSLAA--- --FGED---K FSLEECGSMI
    PpCML25-2  KDLEEAFKLF ---------D LNGDGKISKA ELGTVLRSIG --DE----MS --DADLEQMI RDADTDGDG- EVDLQEFINL N--SDSVHIG KITLGVDLG- -GELPTSSSL ---------- ---TEALQSA FNVFDSDKDG ----FISAGE LQRVLSS--- --LGDD---K ISHDDCLYMI
   PpCML25-11  KDLEDVFKLF ---------D RNGDGKISKA ELGTVLHLLG --DT----LT --DAELDQMI RDVDVDGDG- AIDLQEFIKL N--VEFLDVK P-----SAG- -GE--NGSSD ---------- ---VEGLRSA FSVFDSDKDG ----FISAGE LHRVLSS--- --LGDD---K SSLDDCQYMI
   PpCML25-10  KDLEDVFKML ---------D RNGDGKISKT ELGAVLGSLG --EI----LT --DPELEQMI REVDVDGDG- GIDLQEFIKL N--AECVDAK RL----TAE- -GE--ADSHI ---------- ---EEALQSA FNVFDSDNDG ----FISAGE LHRVLSS--- --LGDD---N ISLDDCRYMI
    PpCML25-4  GEFSDSFKVY ---------D RNGDGKISRE ELGVVMRSLG --QD----VS --DTDLELLI KEVDASGDG- HIDLYEFIDL N--TRPIEIS QPTKTSTSDR LGKEDCGASN ---------- ---TALLSAA FTVFDADNSG ----FISAQD LQRAMVR--- --FGDA---T ASMEECQRMI
    PpCML25-5  QELTDSFKFF ---------D RNGDGKISKE ELGTVVQSLG --HK----VT --DADLDKLM KDVDKNGDG- FIDFQEFKDM N--TRAMIVE CPVDTDVNRN LPQPGSDDSL ---------- -------MSA FNVFDLDKNG ----FISSEE LHSVLVG--- --FGNE---K ISLDDCRFMI
    PpCML25-7  QELTDSFKFF ---------D RNGDGKISKE ELGTVVRSLG --QK----VN --DAELARLI SDVDSNGDG- YIDLQEFIDL N--ARA-IAE CPMGMDTNME IHQYGPGDSM ---------- ---HAAMVSA FNVFDVDRNG ----YISAEE LHRVLVG--- --FGDE---K VSLEDCRSMI
   PpCML25-12  QELTATFKVF ---------D KDSDGKISKS ELGTVLRSLG --DD----LT --DEELTEVI QNADGDGDG- FIDLQEFINF H--TRGDTAS GAGSPQTSS- --SENATSGE ---------- ---RLALQAA FNVFDVDRNG ----FISAEE LQRVMRS--- --LGDM---S TSLVECRHMI
      SmCML27   ELEAVFRLF ---------D ANGDGRISVS ELGAVLKCLA --MGNDPPPT --EEELHKMV EEVDADGDG- FISLDEFLHF H--AQS---- ---------- -------TAS ---------- ---VAELKAA FYVFDLDRNG ----FISADE LHRVLVG--- --LGEV---N LTMEDCGRMI
      SmCML26  QELEHAFRYF ---------D ANGDGKISVA ELGGVLKSLG --EN----PS --EEDLRTMV REVDADGDG- FVDFDEFVHL N--TEILG-- ---------- ----DALAAS ---------- ---VEELKAA FYVFDTDKNG ----YISAEE LYKVMFN--- --LGEK---G VTMEDCNRMI
      BrCML36  ---LQAFKLI ---------D RDNDGAVSRH DLESLLTRL- --GPDPLTE- ---EEIDVML KEVDCDGDG- TVRLEELASR C--VDD---D QSRG------ --------GS ---------- ----DELKET FEFFDADRDG ----KISAEE LLRVFSA--- --IGDE---R CTLEECERMI
      ThCML36  ---LHAFQLI ---------D RDNDGAVTRR DLSSLLSRL- --GPDPPSE- ---EEIDVML REVDCDGEG- TIRIEELASR V--VSS---D QSRD------ ---------S ---------- ----TELKET FEFFDADRDG ----FISAEE LLRVFSA--- --IGDE---R CTLEDCKRMI
    CrCML36-1  ---LQAFKLI ---------D RDNDGAVSRH DLESLLSRL- --GPDPLTE- ---DEISVMI QEVDCDGDG- AIRLEELASR L--VSS---D PSRD------ ---------S ---------- ----AELKET FEFFDADRDG ----LISAEE LLRVFST--- --IGDD---R CTLDDCKRMI
      AtCML36  ---LQAFKLI ---------D RDNDGAVSRH DLESLLSRL- --GPDPLTE- ---EEINVML KEVDCDGDG- TIRLEELASR V--VSL---D PARD------ ---------S ---------- ----TELKET FEFFDADRDG ----LISADE LLRVFST--- --IGDE---R CTLDDCKRMI
    CrCML36-2  ---LKAFTLI ---------D KDNDGAVSRH DLEALLTQL- --GPNPPNK- ---DEINVML REVDRDGHG- TISIDTLATR V--VSSVSSD SSHG------ --------SS ---------- ----TELKET FELFDSDRNG ----LISAEE LLRVFAA--- -AIGDD---R CTLEECSRMI
      BrCML35  ---VEAFKLI ---------D RDDDGVVSRK DLAALLSRL- --SPEPPSP- ---EEVSMML REVDADG--- CISLEELASR V--A-GTSSG EGCI------ --------ET ---------- ----EEMREV FEFFDADRDG ----RISAEE LHRVFGV--- --IGDE---R CTLEDCVRMI
      CrCML35  ---VQAFKLI ---------D RDHDGVVSRG DLAALLSRL- --SPEPPSQ- ---EEVSLML KEVDGDDDGD SIRLEELASR V--A-GTS-G EGSV------ --------ET ---------- ----EELREV FEFFDADRNG ----KISAEE LHRVFGV--- --IGDE---R CTLEECIRMI
      AtCML35  ---VQAFKLI ---------D RDDDGVVSRG DLAALISRL- --SHEPPSQ- ---EEVSLML REVDGGDGG- CISLEDLASR V--A-GTS-G EGSV------ --------ET ---------- ----EELREV FEIFDVDRNG ----KISAEE LHRVFGV--- --IGDE---R CTLEECMRMI
      CpCML35  ---AQAFKMI ---------D KDNDGMISRG ELEALLSRL- --GTKPPSR- ---EEVSLML SEVDGDGDG- SISIDALMSL V-----AGST SEFA------ --------CD ---------- ----AELRDT FDYFDTDHDG ----LISAEE LLRVFSA--- --IGDD---R CTLEDCRRMI
      MeCML36  ---VQAFKLI ---------D RDNDGIVSRT ELEALLSRL- --GAQPPSQ- ---EEVESML SEVDHDGDG- CISVEALINR I-----GSA CEPA------ --------AD ---------- ----EEMRVA FEFFDMDQDG ----KITAEE LLGVYKA--- --IGDE---K CTLDDCRRMI
      FvCML36  ---AQAFRLI ---------D RDNDGVVSRT ELEALLKRLL --GAEPPTQ- ---EEVAMML SEVDGDGDG- CIPLEALLHR F------GPG CEPA------ --------AD ---------- ----SELREA FEVFDTDHDG ----KISAEE LLSVFTA--- --IGDE---R CTLEECRRMI
    MdCML36-1  ---AQAFKXI ---------D RDNDGVVSRK ELEALLCRL- --GNDPQSR- ---EEVTLML SEVDRDGNG- SISLEAVLNR V------GPV SGPA------ --------AD ---------- ----SELRDA FEVFDTDNDG ----KISAEE LLSFFRA--- --IGDE---G CTLEECGRMI
     PperCML36  ---AQAFKLI ---------D RDNDGVVSRK ELEALLSRL- --GADPPSQ- ---EEVMLML SEVDREGNG- SISLEALLSR V------GPV CGPA------ --------AD ---------- ----SELRDA FEVFDSDHDG ----KISAEE LLNVFTA--- --IGDD---R CTLEDCRRMI
```

```
  MdCML36-2  ---AQAFKLI ---------D RDNDGVVSRK ELEAXLYQL- --GNDPPSQ- ---EEVTLML SEVDRDGNG- MIILEALLNQ V------XPV FGLD------ --------AD ---------- ----SELRDA FEVFDTDNDG ----KISAEE LLRFFTS--- --VGDE---G CTLEECRRMI
  MdCML36-3  ---AQAFKLI ---------D RDNDGVVSRK ELEALLYQL- --GNDPPSQ- ---EEVTLML SEVDRDGNG- MIILEALLNQ V------GPV FGLD------ --------AD ---------- ----SELRDA FEVFDTDNDG ----KISAEE LLRFFTS--- --VGDE---G CTLEECRRMI
  MdCML36-4  ---AKAFKLI ---------D RDNDGVVSRK ELEALLCQL- --GNNPPSQ- ---EEVTLML SEVDRDGSG- SITLEALLNQ V------GPV SGPP------ --------AD ---------- ----SELRDA FEVFDTDNDG ----KISAEE LLRVFTA--- --IGDE---R CTLEECRRMI
    MtCML36  ---AQAFRLI ---------D RDNDGVVSRE ELEAVLTRL- --GARPPTP- ---EEIALML SEVDSDGKG- CISVETIMNR V--GSGSSSG SDPN------ --------PE ---------- ----EELREA FEVFDTDRDG ----RISAEE LLRVFRA--- --IGDE---R CTLEECKRMI
    CcCML36  -ELVQACKLL ---------D RDNDGVVLRS ELEALLIRL- --GADPPTQ- ---EEVKSML SEVDREGDG- YIPLEALISR V--G---NSS CEPA------ --------CE ---------- ----PELKET FDFFDADHDG ----KITAEE LFGVFTK--- --LGDE---L CTLDDCRGMI
    CsCML36  -ELVQACKLL ---------D RDNDGVVLRS ELEALLIRL- --GADPPTQ- ---EEVKSML SEVDREGDG- YIPLEALISR V--G---NSS CEPA------ --------CE ---------- ----PELKET FDFFDADHDG ----KITAEE LFGVFTK--- --LGDE---L CTLDDCRGMI
  LuCML36-1  ---QSAFRVI ---------D RDNDGLVSKQ DLTALLNRF- ------GAE- ---EEVATML SEVDLHGDG- CVTVEALMDQ I------GSG WEPA------ --------GR ---------- ----EELKET FEFFDTDHDG ----KITAEE LLSGFVA--- --VGEE---K CTLEECRRML
  LuCML36-2  ---QSAFRVI ---------D RDNDGFVSKQ DLTALLSRL- ------GAE- ---EEVATML SEVDLHGDG- CVTVEALMDQ I------GSG WEPA------ --------GR ---------- ----EELKET FEFFDTDHDG ----KITAEE LLSGFVA--- --VGEE---K CTLEECRRML
  MgCML36-1  SELKQAFEMI ---------D RDGDGKIKKE ELEGLLSRLG --A-EPPSQ- ---EELKLMV SEVDRDGDG- CISLEEFYAI G--SAFAPPT CD-------- ---------- ---------- ----GELRST FDFFDSDRDG ----KITAEE LFRVFET--- --IGDA---R CTLEDCRRMI
  MgCML36-2  FDLKQAFEMI ---------D RDGDGKVTKA ELEDLLCGLG --A-DPPSR- ---EELRLML SEVDRDGDG- CITLEEFHSI G--SAFAPPT CD-------- ---------- ---------- ----TEMRET FDFFDSDRDG ----KITAEE LFGVFRT--- --IGDA---K CTLEDCRRMI
    SlCML36  SELVQAFQMI ---------D GDGDGKIHKE ELSAILTRVG --S-DPPTE- ---EELVQLL NEVDVNGDG- CISLQEFGAI S--SAFGPPS CD-------- ---------- ---------- ----SEMRDT FEFFDANHDG ----KITAEE LFNVFRT--- --IGDG---R CTLEECRRMI
  StCML36-2  SELVQAFQMI ---------D VDGDGKIHKE EISAILTRVG --S-DPPTE- ---EELVQLL NEVDVNGDG- CISLQEFGAI S--SAFGPPS CD-------- ---------- ---------- ----SEMRDT FEFFDANHDG ----KITAEE LFNVFRT--- --IGDG---R CTLEECRRMI
  StCML36-3  AELIHAFHMM ---------D TDGDGKIRKE ELEAILSRVG --T-EPPSK- ---EELMLLL EEVDVNGDG- CISLQEFGAI S--SAFGPPA CD-------- ---------- ---------- ----SELRDA FDFFDADHDG ----KITADE LFSVFRQ--- --IGDS---R CTLEECRRMI
    SlCML35  SDLVRAFSVI ---------D -NGDGKIKKE KLEAILSRVG --GKSPPSE- ---EELVLLL DELDENGDG- CISLENFETI T--SAFESTP TSAA------ --------GD ---------- ---AGEMRDA FDFFDEDHDG ----KITGEE LFNVFRM--- --IGDE---R CTLEECKRMI
  StCML36-1  SDLVQAFSVI ---------D -NGDGKIRKE QLEAILSRVG --GKSPPTE- ---EELVLLL DELDKNGDG- CISLENFETI T--SAFEPTP PSAA------ --------ED ---------- ---AGEMRDA FDFFDEDHDG ----KITGEE LFNVFRM--- --IGDE---R CTLEECKRMI
    VvCML36  ----DSSTNL ---------K TDGDGKITKR ELEALLSRVG --V-EPPSE- ---EEIMMML SEVDRDGDG- CISLEEFGAI S--SAFGP-A CD-------- ---------- ---------- ----TELRDA FCFFDTDRDG ----KITAEE LNQVFAA--- --IGDD---R CTLEDCQRMI
   OsCML1-1  ---AASSSGT ---------K KPAAAAVTRE DLEVALRRI- -----VSSK- ---EELAAML AEADYAGE-- -LVLEEIAAA A--------- ---------- --------AD ---------- ---EGELKET FAVFDADGDG ----RISAEE LRAVLAS--- --LGDE---L CSVDDCRRMI
   OsCML1-2  ---AASSSGT ---------K KPAAVAVTRE DLEVALRRI- -----VSSK- ---EELAAML AEAECAGE-- -LVLEEIAAA A--------- ---------- --------AD ---------- ---EGELKET FAVFDADGDG ----RISAEE LRAVLAS--- --LGDE---L CSVDDCRRMI
    AcCML38  --LERVFRFF ---------D EDKDGKISPA ELQSCVKTIG G--------E LSTTEAVTLV ESLDSD-GDG LLGLEDFAKL M--EANG--- ---------- ---EEEQN-- ---------- ----KDLREA FGMYENN--G --SGCITPTS LNQMLSRLG- -ESK-T---- --IEECQSII
    EgCML37  --LRRVFDYF ---------D ENGDGKISPA ELRSCVSSLG G--------D LSSEEAEMAV RASDLD-GDG MLGFEEFQRL V--ESGG--- ---------- ---EEEKR-- ---------- ----KDLEEA FGMYEME--G --SGCITAGS LRRMLSRLG- -EAR-S---- --VEDCKAMI
    TcCML37  --LQMVFDFI ---------D ANGDGKISAE ELQRCVRTAG G--------H LSMDEAELAV KSSDLD-GDG MLGFHEFQEL V--EGGGS-- ---------- --TEEERK-- ---------- ----KELREA FGMYVME--G --SECITPAS LRRMLRRLG- -ESK-S---- --IKDCKAMI
  VvCML39-2  --LQRVFRYF ---------D KNGDGKISPE ELQSCVRAVG G--------E LSAKEAEAAV KSSDMD-GDG MLGMEDFEML M--EANGE-- ---------- ---EEEKT-- ---------- ----KDLKEA FGMYEME--G --SGCITPKS LKRVLSRLG- -ESK-T---- --IEDCKVMI
  FvCML38-2  --LEKVFYYF ---------D ENGDGKISPA ELQTCVRTVG G--------E LSEEEAQAAV EASDMD-GDG MLGFEEFQKL M--EASNG-- ---------- --DEENKS-- ---------- ----KELRDA FGMYEME--G --SGCITPAS LKRMLGRLG- -ESR-S---- --TEDCKSMI
 PperCML38-  --MWKVFDFF ---------D ENGDGKISPA ELQTCVRSVG G--------E LSEEEAEAAV EASDLD-GDG LLGFEEFQKL M--EATN--- ---------- ---EDNNN-- ---------- ----QQLRDA FGMYEME--G --SGCITPAS LKRMLSRLG- -DSR-S---- --IDDCKAMI
    RcCML39  --LQRVFDYF ---------D ENRDGKISAA ELQRCVRAVG G--------E LSTEDAEAAV ISADTD-GDC LLGFEDFQRL M--EGSNS-- ---------- ---EEEKK-- ---------- ----EELRQA FGMYETE-PG --SGFISPAS LKRMLSRLG- -DSK-S---- --LTDCSQMI
    CcCML37  ELQKKVFNFF ---------D ENGDGRITAA ELQSCLMTVG GGGEL----S ISVADAEAAI QSSDLN-GDG VLDFDEFLKL M--EGNQE-- ---------- ---EEEKN-- ---------- ----CELRDA FALYVMD--G --SNSITPAS LKRMLTRLGL GESK-S---- --IDDCKAMI
    BrCML37  --LRTVFDYI ---------D ANSDGKISGT ELQNCVSLLG G--------A LSSREAEEVV KISDVD-GDG LMDFEEFLKL M--EGEDG-- ---------- --SDEERR-- ---------- ----KELREA FRMYVTE--G --EEFITAAS LRRTLSRLG- -ESC-T---- --VDACKVMI
    AtCML37  --LRTVFDYM ---------D ANSDGKISGE ELQSCVSLLG G--------A LSSREVEEVV KTSDVD-GDG FIDFEEFLKL M--EGEDG-- ---------- --SDEERR-- ---------- ----KELKEA FGMYVME--G --EEFITAAS LRRTLSRLG- -ESC-T---- --VDACKVMI
    ThCML39  --LRTVFDYI ---------D ANSDGKISCT ELQSCVSLLG G--------A LSLSEAEEVV KISDVD-GDG LMDFAEFMKL M--EGEDG-- ---------- --SDEERR-- ---------- ----RELKEA FGMYVME--G --EEFITAAS LQRTLSRLG- -ESC-T---- --VDACKVMI
    CrCML37  --LRTVFDYM ---------D TNSDGKISGE ELQSCFSLLG G--------A LSSREAEEVV KISDVD-GDG FIDFGEFLKL M--EGDDG-- ---------- --SDEEKR-- ---------- ----KELREA FGMYVME--G --EEFITAAS LRRTLSRLG- -ESC-T---- --VDACKGMI
    CpCML37  --LEVVFNYM ---------D ENGDGKISAA ELRNCVKMVG G--------E LSAEEAEAAV RFSDLD-GDG FLDLNEFHKL M--EGEGR-- ---------- --SVEDKK-- ---------- ----SELKEA FEMYVVE--G --NDCITATS LKRMLRRLG- -QSS-S---- --IDDCKAMI
  SlCML38-1  --LERVFTYF ---------D ENGDGKVSPA ELRKCVKAVG G--------E LTVEEAEMAV RLSDSD-GDG LLGIEDFTKL M--EGMEE-- ---------- ---ERNKE-- ---------- ----GELMGA FGMYETE--G ----YITPKS LKNMLSRLG- -EST-S---- --IDNCKAMI
  StCML38-3  --LERVFTYF ---------D ENGDGNVSPP ELRRCMKAVG G--------E LTMEEAEMAV RLSDSD-GDG LLGLEDFTKL M--EGMEE-- ---------- ---ERNKE-- ---------- ----SELMGA FGMYEME--G ----YITPKS LKMMLSRLG- -EST-S---- --IDKCKVMI
  SlCML39-2  --LERVFTYF ---------D ENGDGKVSPM ELRRCMKAVG G--------E ITVEEAEMVV RLSDSD-GDG LLGFEDFTKL M--EGMEE-- ---------- ---ERNKE-- ---------- ----SELMGA FGMYEME--G ----YITPKS LKMMLSRLG- -EST-S---- --IDKCKVMI
    StCML37  --LERVFTYF ---------D ENGDGKVSPT ELRRCMKAVG G--------E MTVEEAEMAV RLSDSD-GDG LLGLEDFTKL M--EGMEE-- ---------- ---ERNKE-- ---------- ----SELMGA FGMYEME--G ----YITPKS LKMMLSRLG- -EST-S---- --IDKCKVMI
  StCML38-2  --LERVFTYF ---------D ENGDGKVSPA ELRKCVKAVG G--------E LTVEEAEMAV RLSDSD-GDG LLGIEDFTKL M--EGMEE-- ---------- ---ERNKE-- ---------- ----SELIGG FGMYEME--G ----YITPKS LKNMLSRLG- -EST-S---- --IDNCKAMI
  SlCML38-2  --LERVFTYF ---------D EDGDGKVSPA ELRRCVKAVG G--------D LTTEEAEMAV RLSDSD-GDG MLGLEDFSKL M--E-LGE-- ---------- ---KDNKE-- ---------- ----SELRGA FEMYEME--G --SGQITPKS LKRMLSRLG- -ESK-S---- --IDNCKAMI
  StCML38-1  --LERVFTYF ---------D EDGDGKVSPA ELRRCVKAVG G--------D LTTEEAEMAV RLSDSD-GDG MLGLEDFSKL M--E-LGE-- ---------- ---KDNKE-- ---------- ----SELRGA FEMYEME--G --SGQITPKS LKRMLSRLG- -ESK-S---- --IDNCKAMI
  BrCML39-1  --LEAVFAYM ---------D ANRDGRISAE ELKKSFNTLG D--------Q LSDEEAEAAV KLSDID-GDG MLDFEEFSQL I--KGNDE-- ---------- -FTEEEQK-- ---------- ----RKMMEA FRMYIDE--G --EDCITPRS LKTMLMKLG- -ESR-T---- --SDDCVVMI
  ThCML23-1  --IEDVFAYM ---------D ANRDGRISAQ EVKKILKTLG E--------Q LSDEEAEAAV KLSDMD-GDG MLDFEEFAQL I--KGSDE-- ---------- -FTEEEKK-- ---------- ----IKIMEA FRMYIAE--G --EDCITPRS LKMMLMKLG- -EAR-T---- --IDDCILMI
  BrCML39-2  --LAAVFAYM ---------D ANRDGRISAE ELKKSFKTLG E--------Q LSDQEAEAAV KLSDLD-GDG MLDFEEFAQL L--KGGDE-- ---------- -FTEEEKK-- ---------- ----SKIMEA FRMYIAE--G --EDCITPRS LKMMLMKLG- -ESR-T---- --TDDCVVMI
    CrCML39  --LEAVFAYM ---------D ANRDGRISAE ELKKSFRTLG E--------P MSDEEAEAAV KLSDID-GDG MLDLHEFAQL I--RGNDE-- ---------- -FTEEEKK-- ---------- ----RKIMEA FRMYIAE--G --EDCITPGS LKMMLMKLG- -ESR-T---- --TDDCRVMI
    AtCML39  --LEAVFAYM ---------D ANRDGRISAE ELKKSFKTLG E--------Q MSDEEAEAAV KLSDID-GDG MLDINEFALL I--KGNDE-- ---------- -FTEEEKK-- ---------- ----RKIMEA FRMYIAD--G --EDCITPGS LKMMLMKLG- -ESR-T---- --TDDCKVMI
  BrCML38-2  --IEAVFAYM ---------D ANKDGRISPD ELQKSFMTLG E--------Q LSDEEAEAAV RLSDTD-GDG MLDFEEFAQL I--KGDDK-- ---------- -CSEEEKK-- ---------- ----MELKEA FKMYIAE--G --EECITPRS LKMMLKKLG- -ESR-T---- --TDDCRVMI
    ThCML38  --LEAVFAYM ---------D ANRDGRISAD ELQKSFTTLG E--------Q LSDEEAEAAV RLSDTD-GDG MLDYEEFAQL I--KGDDE-- ---------- -STEEDKT-- ---------- ----IELKEA FKMYIAE--G --EECITPRS LKMMLKKLG- -ESR-T---- --TDDCRVMI
  BrCML38-1  --LEAVFAYM ---------D ANRDGRISPH ELQKSFMTLG E--------Q LSDEEAEAAV RLSDTD-GDG MLDFQEFAQL I--KGDDD-- ---------- ---QEDKK-- ---------- ----TELKEA FRMYIAE--G --EECITPRS LKTMLKKLG- -ESR-T---- --TDDCRVMI
    AtCML38  --LEAVFSYM ---------D ANRDGRISPE ELQKSFMTLG E--------Q LSDEEAVAAV RLSDTD-GDG MLDFEEFSQL I--KVDDE-- ---------- ---EE-KK-- ---------- ----MELKGA FRLYIAE--G --EDCITPRS LKMMLKKLG- -ESR-T---- --TDDCRVMI
    CrCML38  --LEAVFAYM ---------D ANRDGRISAE ELKKSFRTLG E--------Q LSDEEAEAAV KLSDSD-GDG MLDFEEFAQL I--KRDDE-- ---------- -FTEEEKK-- ---------- ----MELKEA FRMYISE--G --EECITPRS LKVMLNKLG- -ESK-T---- --TDDCKVMI
  AcCML41-2  DELQQVFAHF ---------D SDGDGKISSL ELRAYFGSIG E--------Y MSHDEAQGVI NDLDVN-GDG LLDFHEFTQL M--MDRD--- ---------- -----GAD-- ---------- ----EDLKRA FEMFEAEK-G --SGCITPKS LQRMLNRLG- -DSK-S---- --FEDCEAMI
  AcCML41-1  DELQQVFAHF ---------D SDGDGKISSL ELRAYFGSIG E--------Y MSHDEAQGVI NDLDVN-GDG LLDFHEFTQF M--TDRD--- ---------- -----GAD-- ---------- ----EDLKRA FEMFEAEK-G --SGCITPKS LQRMLNRLG- -NSK-S---- --FEECEAMI
    AtCML41  EELRQVFSHF ---------D SDGDGKISAF ELRHYFGSVG E--------Y ISHEAAQEAI NEVDTD-ADG SLGFEDFVGL M--TRRDL-- ---------Y GDGEVDGD-- ---------- ----GELKTA FEMFEVEK-G --SGCITPKG LQKMLVKLG- -ESR-T---- --YGECEAMI
    EgCML41  QELQEVFCRF ---------D TDGDGKISAL ELRAYFGSVG E--------Y MSQEDAQSVI EELDTD-GDL LIDFSDFLKL M--KT----- ---------- -----DGD-- ---------- ----DDLRRA FEMFEDEK-G --SGSITPRG LQTMLRRLG- -DAK-S---- --YEECENMI
    CcCML41  DELKQVFRHF ---------D GDGDGKISAL ELRAYFGSIG E--------Y MSYEEARAAI DDFDTD-GDS LLDFQDFTRM L--LKDDR-- ---------G ----DGDD-- ---------- ----EDLKKA FEMFELEKK- ---GCITPKG LQRMLNRLG- -DSK-S---- --YDECVAMI
    CsCML41  DELKQVFRHF ---------D GDGDGKISAL ELRAYFGSIG E--------Y MSYEEARAAI DDFDTD-GDS LLDFQDFTRM L--LKDDR-- ---------G ----DGDD-- ---------- ----EDLKKA FEMFELEKK- ---GCITPKG LQRMLNRLG- -DSK-S---- --YDECVAMI
  MdCML41-2  DELREVFRYF ---------D GDGDGKISAS ELRAYFGSIG E--------Y MSHEEAEAAI NDLDAD-GDN LLDFEDFLQL M-----NS-- ---------G ----GGDD-- ---------- ----EDLKKA FEMFELEK-- ---GVITPKS LQRMLHRLG- -DTK-S---- --YDECVTMI
  MdCML41-1  DELREVFRYF ---------D GDGDGKISAS ELRAYFGSIG E--------Y MSHEEAEAAI NDLDAD-GDN LLDFEDFLQL M-----NS-- ---------G ----GGDD-- ---------- ----EDLKKA FEMFELEK-- ---GVITPKS LQRMLHRLG- -DTK-S---- --YDECVTMI
  MtCML41-1  KGLREVFKYF ---------D GDGDGKISAY ELRSYFGSIG E--------H MSHEEAERVI NYLDGD-GDN LLDFNDFIKL M--KGEGG-- ---------- ----RDDD-- ---------- ----KDLRKA FEMFVWEEKE ---GCITPKG LQRMLQRLG- -DDR-S---- --YEECVVMI
  PvulCML41  NGLTEAFHHL ---------D GDGDGKISAY DLRSYFGSIG E--------H MSHEEVEGVI HDLDSD-GDN MLDLQDFTKL M--KRGGG-- ---------- ----DGDE-- ---------- ----GDLRKA FEMFVWEKEG --CGCITPKG LQRMLHRLG- -DDR-S---- --YDECVAMI
    PtCML41  DELREVFRCF ---------D SDGDGRISAL ELRAYFGSIG E--------Y MSHEEAQLAI NDLDAD-QDN LLDFQDFLRL M--KREAN-- ---------- -----DNT-- ---------- ----DDLKMA FEMFEMEK-G --SGYITPKG LQRMLRRLG- -DAK-S---- --YDECVAMI
    RcCML41  DELKEVFRHF ---------D TDGDEKVSAL ELRSFFGSVG E--------F MSHEEAESVI NDLDSD-GDK LLDFNDFLKL M--KREGN-- ---------S NPNDQDHE-- ---------- ----DDLKKA FEMFEMEK-G --SGCITPKG LQRMLHRLG- -DSK-S---- --YDECVAMI
    SlCML41  DEFREVFRHF ---------D TDNDGKISAF ELRAYFGSIG E--------H MSHEDAQEIV DELDRD-GDN FIDFDDFKKL L--MQKEG-- ---------S ----SEED-- ---------- ----ESLKSA FEMYEVEK-G --CGRITPKS LQRVLSRLG- -DSK-S---- --YDECVTMI
    StCML41  DEFREVFRHF ---------D TDNDGKISAF ELRAYFGSIG E--------H MSHEDAQEIV DELDTD-GDN FINFEDFKKL L--MQKEG-- ---------S ----SEED-- ---------- ----ESIKSA FEMYELEK-G --CGRITPKS LQRVLSRLG- -DSK-S---- --YDECVTMI
    CpCML40  EQYLRVFRYF ---------D GNGDGKISAE ELEQCVKSIV GDQTNGGGLN LSLKEAEAVV AALDGD-GDG LLGVEEFMRL T--EGDG--- ---------- --GEEE---- ---------- ------LREA FKLY-EMEGS --G-CITPRS LKRMLSRLG- -ERK-T---- --MEECEVMI
  CcCML38-2  QQYERVFNHF ---------D SNGDRKISPS EIQQCVEAI- G------GE- LSLAEAEAAV EFLDAD-GDG LLGFDDFVRL V--E-GS--- ---------- --GEEEKM-- ---------- ----NDLKEA FKMY-EMDGC --G-CITPKS LKRMLSRLG- -QSK-S---- --DDECKSMI
  CsCML38-2  QQYERVFNHF ---------D SNGDRRISPS ELQQCVEAI- G------GE- LSLAEAEAAV EFLDAD-GDG LLGFDDFVRL V--E-GS--- ---------- --GEEEKM-- ---------- ----NDLKEA FKMY-EMDGC --G-CITPKS LKRMLSRLG- -QSK-S---- --DDECKSMI
  FvCML38-1  AQYERVFNQF ---------D GNGDGKISPM ELQQCVEAI- G------GE- LSSSEAEAAV EYLDSD-RDG LLGFDDFVKF V--E-GG--- ---------- --DEEEKV-- ---------- ----NDLREA FKMY-DTDGC --G-CITPKS LKRMLSRLG- -ESR-S---- --VDECKVMI
  MtCML41-2  TQYIRVFNHF ---------D ENGDGKISSS ELRQCVEAI- G------AK- MSNEEAEMAV ELLDSD-GDG LIGLDDFVKF V--E-GG--- ---------- --KEEKV--- ---------- ----NDLREA FKMY-EMEGC --G-CITPKS LKRMLGRLG- -ECR-S---- --VDECQAMI
 PvulCML38-  SQYVCVFNHF ---------D ENGDGKISAS ELRQCVEAI- G------GK- LSWKEAESLV EVLDSD-KDG LVGLDDFVRF V--E-EV--- ---------- --EEEKV--- ---------- ----EDLREA FKMY-EMEGC --G-CITPKS LKRMLGRLG- -ECK-S---- --IDECQGMI
 PvulCML38-  RQYERVFKQF ---------D ENGDGKISAW ELQQCVEAM- G------EE- LSAADAAATV TAMDAD-GDG MVGFDDFVRF V--E-GG--- ---------- --KEEKE--- ---------- ----NDLKEA FKMY-EMEES --G-CITPRS LKMMLSRLG- -EST-S---- --IDECEVMI
  EgCML40-2  EQYARVFSHF ---------D GDKDGKISPA ELRQCVAAV- G------GE- MSPAEAEAAV AALDGD-GDG HLGFEDFMAF V--E-GG--- ---------- --GAEERA-- ---------- ----ADLKEA FRMY-EVDGS --GGCITPKS LKRMLSRLG- -ESR-S---- --LDECKAMI
  MgCML38-2  EQYERVFKHF ---------D SNGDGRISPE ELRHCVASI- G------GV- MTADEAEAAV ALVDSD-GDG QMCLDDFVRI V--E-GA--- ---------- --GDEEKA-- ---------- ----SDLKWA FKLY-EMEGS --G-CITPES LKRMLSKLG- -QRS-T---- --VDQCRNMI
  MgCML38-1  EQYERVFNHF ---------D SNGDGRISPA ELRQCVCTI- G------GV- MTAEEAAAAV ALTDSD-GDG LMCLDDFVKV V--E-GA--- ---------- --GEEEKT-- ---------- ----SDLKAA FGMY-ELEGR --G-CITPES LKRMLSRLG- -QQR-S---- --VDECKNMI
  MgCML38-3  NNYERIFNRF ---------D SNGDGKICPV ELRRCVGSV- G------GE- MTEEEAAAAV VLMDGD-GDG LLGLEDFVRI V--E-GA--- ---------- --GDEEKA-- ---------- ----SDLKEA FKMY-EMEGS --G-CITPKS LKRMLSRLG- -ERR-D---- --VDECTGMI
 CsatCML38-  ALYEKVFKQV ---------D GDGDGKLSPP ELQRCILGV- G------GS- LTIEEAETVV EKLDSD-GDG LVGWDEFVEF V--E-GV--- ---------- --GEEEKV-- ---------- ----NDLKEA FRMY-EMDGC --G-FITTKS LKRMLSKLG- -ESR-S---- --IDDCKKMI
  CsatCML41  RCYKRVFEHF ---------D SDGDGKISPS ELRECIAVV- S------GEK LSVLEAQEAV AEFDSD-GDG QLKEEDFVRF V--D-GG--- ---------- --GEERV--- ---------- ----KELREA FKMY-EMKGS --G-LITAES LRRMLRKLG- -ETK-S---- --LGDCTAMI
  SlCML37-1  KQHQRLFDRF ---------D ENRDGKISAE ELQQCVHLI- G------KD- MSYEEAKAAV EHNDSD-NDG LLDFEDFVRL I--E-GG--- ---------- --SEEKA--- ---------- ----HELKEA FKMY-EMEGC --G-CITPES LNRMLSRLG- -EST-T---- --IDECRGMI
  StCML38-4  KQHQRLFDRF ---------D ENRDGKISAE ELQQCVHLI- G------KD- MSYEEAKAAV EHNDSD-NDG LLDFEDFVRL I--E-GG--- ---------- --SEEKA--- ---------- ----HELKEA FRMY-EMEGC --G-CITPES LNRMLARLG- -EST-T---- --IDECRGMI
  SlCML37-2  EGYKRLFEHI ---------D ENGDGKISVL ELQQCVDLII G------KDV LLFEEAEAAI VAHDSD-NDG LLDFDDFMRL V--EDGG--- ---------- --TKEEKE-- ---------- ----RELKEA FRMY-EMEGC --G-CITPES LKRMLHRLG- -EKK-S---- --VDECRGMI
  StCML23-1  EGYKRLFEHI ---------D ENGDGKISAL ELQQCVHLI- G------KDI LLFEEAEAAI VAHDSD-NDG LLDFDDFMRL V--EDGG--- ---------- --TEEEKE-- ---------- ----RELKEA FRMY-EMEGC --G-CITPES LKRMLHKLG- -EKR-T---- --VDECRGMI
  CcCML38-1  REYERVFVYF ---------D ENGDGKVSPS EIKNRMGMI- -----VGGGD VLLNEVEVAI ESLDKD-GDG FLDLEDLVGL I--EGAS--- ---------- ---AEEKL-- ---------- ----KDLREA FGLY-DFDNR --G-FISPND LKRMLAKLG- -ESK-S---- --IDECRMMI
  CsCML38-1  REYERVFVYF ---------D ENGDGKVSPS EIKNRMGMI- -----VGGGD VLLNEVEVAI ESLDKD-GDG FLDLEDLVGL I--EGAS--- ---------- ---AEEKL-- ---------- ----KDLREA FGLY-DFDNR --G-FISPND LKRMLAKLG- -ESK-S---- --IDECRMMI
  FvCML38-3  RGFERVFSYF ---------D ENGDGKISPS ELRNRLGLM- -------GGE MLQNEAEVAV GYLDSD-GDG LLGLDDLVEL M--EGGE--- ---------- ---EEEKM-- ---------- ----KGLREA FEVY-DLEGC --G-FITPKS LKRMLNKLG- -ESK-S---- --IEECKAMI
  MdCML39-2  KGYERVFRYF ---------D EDGDGKISPS ELRRRLGLM- -------GGE LLQNEAEVAV EMLDSD-GDG LLGLEDLVGL M--EGGK--- ---------- ---EEEKV-- ---------- ----KGLRXA FEVY-DVXRC --G-FITPSS LKRMLSKLG- -ESK-S---- --IDECKVMI
  MdCML39-1  KGYERVFRYF ---------D EDGDGKISPS ELRRRLGLM- -------GGE LLQNEAEVAV EMLDSD-GDG LLGLEDLVGL M--EGGK--- ---------- ---EEEKV-- ---------- ----KGLRXA FEVY-DVXRC --G-FITPSS LKRMLSKLG- -ESK-S---- --IDECKVMI
    MdCML38  KGYERVFRYF ---------D EDGDGKVSPA ELRRRLGLM- -------GGE LLQNEAEAAV EMLDSD-GDG LLGLEDLVGL M--EGGE--- ---------- ---EEDKV-- ---------- ----KGLREA FELY-DVEGC --G-FITPSS LKRMLSKLG- -ESK-S---- --VDECKVMI
 PperCML38-  RGFDRVLSYF ---------D EDGDGKISPS ELRRGLGFM- -------GGE LLQKEAEVAV ELLDSD-GDG LLGLEDLVCL M--EGGE--- ---------- ---EEEKV-- ---------- ----KGLRDA FQVY-DVEGC --G-FITPKS LKRMLSSLG- -ESR-S---- --IDECKVMI
    TcCML38  EEYERVLRYF ---------D EDGDGKISAS ELSRRLGQM- -------GGE LLLNEAEAAI EALDSN-GDG LLDLEDLIGL M--EEVG--- ---------- ---EEDKL-- ---------- ----KDLREA FGMY-DADGC --G-FITPKG LKKMLNRLG- -ESK-S---- --IDECKVMI
  MtCML38-1  AGFEHVLRYF ---------D EDGDGKVSPA ELRQRLRIM- -------GEE ILLKEAEMAI EAMDSD-GDG YLSLEELIAL M--EEGG--- ---------- ---EEQKL-- ---------- ----KDLREA FEMY-DSEKC --G-FITPKS LKRMLKKMG- -ESK-S---- --IDECKAMI
  MtCML38-2  AGFEHVLRYF ---------D EDGDGKVSPA ELRQRLRIM- -------GEE ILLKEAEMAI EAMDSD-GDG YLSLEELIAL M--EEGG--- ---------- ---EEQKL-- ---------- ----KDLREA FEMY-DSEKC --G-FITPKS LKRMLKKMG- -ESK-S---- --IDECKAMI
  MtCML38-3  MEFERVLSYF ---------D EDGDGKISPN ELRSRMAKI- -------SGE FQLKEVEIAI EALDSD-GDG LLSLEDLIAL M--ESGG--- ---------- ---EEEKL-- ---------- ----KDLREA FEMY-DDEGC --G-YITPKS LKRMLKKLG- -DSK-S---- --IEECKVMI
    PtCML37  GEFEVVFRCL ---------D EDGDGKVSPS ELSHRLSLI- -------GGD LLMKEAELAV EGLDSD-GDG LLGLEDLVRL M--EAGG--- ---------- ---EEEKL-- ---------- ----HDLREA FHLY-DIDNC --G-FIRAKD LKTMLGRLG- -ESR-S---- --IDECEVMI
 PvulCML38-  GEYERVLRYL ---------D RDGDGKISAS ELRNGIGML- -------GGE VGVREVEMAI EGLDSD-GDG FLCLEDLMKM M--EGVE--- ---------- ---EEEKV-- ---------- ----KDLREA FDLY-DTERC --G-FITPKA LKRMLKKLG- -DFT-S---- --MDQCRAMI
 CsatCML38-  AQYDRVLSYF ---------D EDGDGKISPS ELRNRLGLI- -------GGE LQQAEAEAAV ESLDSD-GDG LLCVGDIERL L--EVG---- ---------- ---EEEKL-- ---------- ----KDLKEA FALY-DSEGC --G-FITPKN LKKMLRKLG- -ERK-S---- --TEECKMMI
    EgCML40  EQYERVFRCF ---------D EDGDGKLSPS ELRRHVGLT- -------GRE LLLKEAEVAV ESLDSG-GDG LLCLEDFVGF M--EGGG--- ---------- ---EEEKM-- ---------- ----SDLREA FGMY-VTDGC --G-FITAQS LKRMLSRLG- -QSK-A---- --EDDCEAMI
  EgCML39-1  EQYERVFRCF ---------D EDGDGKLSPS ELRRHVGLT- -------GGE LLLKEVEVVV ESLDSG-GDG LLCLEDFVGF M--EGGG--- ---------- ---EEEKM-- ---------- ----GDLREA FGMY-VADGC --G-FITPKS LKRMLSRLG- -QSK-F---- --EDDCKAMI
    EgCML38  EQYERVFRCF ---------D EDGDGKLSPS ELRRHVGLT- -------GGE LLLKEAEVTV ESLDSD-GDG LLCLEDFVGF M--EGGG--- ---------- ---EEEKM-- ---------- ----GDLREA FGMY-VTDGC --G-FITPKS LKRMLSRLG- -ESK-A---- --EDDCKAMI
  EgCML39-2  EQYERVFRCF ---------D EDGDGKLSPS ELRRCVGLT- -------GGE VLLKEVEVAV ESLDSD-GDG LLCLEDFVGF M--EGGG--- ---------- ---EEERM-- ---------- ----GDLREA FGMY-VTDGC --G-CITPKS LKRMLSRLG- -QSK-A---- --EDDCEAMI
  EgCML40-1  -RYERVFRCF ---------D EDGDGKLSPL ELRRQVGLI- -------GGE LLLEEVEVAV ESLDSD-GDG LLCLEDFVGL M--EGGG--- ---------- ---EEEKM-- ---------- ----GDLREA FGMY-VTDGC --E-FITPKS LKRMLSRLG- -QSK-T---- --VDECEAMI
  VvCML39-1  VQFEQVFNQF ---------D EDHDGKLSPS ELTRCVGLI- -------GGE LPLKEAEAVV QQLDSD-GDG LLSLEDFIRL M--EGEGGEG ---------- ---EEEKM-- ---------- ----NELREA FGMY-DMDGC --G-FITPKS LKRMLSRLG- -QKK-S---- --VDECRVMI
 PvulCML38-  TDFERVLKCF ---------D EDGDGKISPW ELRKKLGMM- -------GGE LGMKEVELLM EELDCD-GDG FLSLEDLVKV M--EAAG--- ---------- ---EEEKL-- ---------- ----RDLGEA FEMYNDCEML --G-FITPKS LQMMLSRLG- -ESK-S---- --IHQCTAMI
 PvulCML38-  TDFERVLKHF ---------D EDGDGKISPW ELRKKLGMM- -------GGE LGLKEVELLM EELDCD-GDG FLSLEDLVKV M--EAAG--- ---------- ---EEEKL-- ---------- ----RDLGEA FEMYNDCEML --G-FITPKS LQMMLSRLG- -ESK-S---- --IHQCTAMI
  VvCML41-1  NVYERIFKRF ---------D EDGDGKLSPS ELRRCVGTI- -------GEE LLTEEAQEVV ESMDSD-GDG LLGLEEFVGW M--ERE---- ---------- --DEERKM-- ---------- ----EELREA FGMY-EMDGS --G-CITPKS LKRMLSRLG- -ESR-S---- --VEECSVMI
  VvCML41-2  DVYERIFKRF ---------D EDGDGKLSPS ELRCCLGTI- -------GEE QPMEEAQEVV ESMDSD-GDG LLGLEEFVGW M--ERE---- ---------- --GEERKM-- ---------- ----EDLREA FRMY-EMEGS --G-CITAKS LKRMLSRLG- -ESR-S---- --VEECSVMI
    VvCML38  SIYERIFKRF ---------D EDGDGKLSPS ELRRCVGTI- -------GEE LLMEEAQEVV ESMDSD-GDG LVGLEEFVGW M--KRE---- ---------- --GEERKM-- ---------- ----EELREA FRMY-EMEGS --G-CITPKS LKRMLSRLG- -ESR-S---- --VEECSVMI
  VvCML41-3  NVYERIFKRF ---------D EDGDGKLSPS ELRRCLGTI- -------GEE LLMEEAQELV ESMDSD-GDG LLGLEEFVGW M--EREDGLL GLEEFVGWME REGEERKM-- ---------- ----EDLREA FRMY-EMEGS --G-YITPKS LKRMLSRLG- -ESR-S---- --VEECSVMI
    LuCML38  EQYKRVFADF ---------D SDGDGKISCS ELRLCVG--- -------GDS LTEEEAEAAV RFSDSD-GDG MLDLEDFVRL V--EAGD--- ---------- ---EEEKV-- ---------- ----QDLREA FKMYAAAEN- --GKMITPKS LKRMLSRLG- -MRR-S---- --TAECGVMI
 CsatCML38-  PYHTLLFYNI ---------D TDSDGKVSPH DLLTRLPAL- ------TGQV LTEAEAASAV AAFDSD-GDG LLGMEEFGRL V--EGVVV-- ---------- --GGDDDD-- ---------- ----REVREA FQMY-EMEGL --G-VITPVS LRRMLSRLG- -ESR-S---- --VGDCKRMI
    AtCML40  DEYQRVFSCF ---------D KSHQGKVSVS TIERCVDAIK S-----GKRA VVDQEDTTNP NPEEST-DDK SLELEDFVKL V--EEGE--- ---------- ---EADKE-- ---------- ----KDLKEA FKLYEESEG- -----ITPKS LKRMLSLLG- -ESK-S---- --LKDCEVMI
    AcCML39  NEFECVFRHC ---------D VDGDGKVSPM ELQHCVKNVC GE-------- ISEQEVEDIV SSLDSD-GDG LLGLEDFIGL V--KGKGKQE EGIEE----- ------NI-- ---------- ----KDLKEA FRMY-AMEGS ---EGITPTS LKRMLNRLGE SK---T---- --IEECMVMI
    BdCML31  SELRTLFAAL ---------D KDADGRISAS ELRACMGATL GED------- VPAEEAEALV ASADAD-GDG MLCEEEFVRL A--QQASWAG EEEEEE---- ------RC-- ---------- ----RVLKEA FGMY-EMEGL ---GCITPAS LRRMLGRLGS DR---P---- --VGECRAMI
    OsCML31  CESSALFATF ---------D HDGDGRISAA ELRLCMKTTL GEE------- VSDEEAGQLV ASVDAD-GDG LLCEAEFVRL V--QAAEV-- EEEDER---- ------RG-- ---------- ----TGLREA FGMY-EMEGE ---GCITPTS LRRMLRRLGS DQ---D---- --IDDCRAMI
    PhCML31  SELGSLFAAF ---------D KDADGRISAA ELRLCMKATL GED------- VSAEDAEALV ASVDAD-GDG LLDGDEFARL V-----HA-- EVGEEE---- ------RH-- ---------- ----RGLRQA FGMY-EMEGQ ---GCITPTS LKRMLSRLGA HQ---E---- --IDDCRAMI
    SbCML31  SELGSLFAAF ---------D ADADGRISAS ELRECMRATL GAEAA----- VSAEEAEALV AEADAD-GDG LLDAAEFAGL V--ARLQQ-- DAGEED---- ------RH-- ---------- ----RGLRQA FGMYSETENN VEAGCITPAS LRRMLARLGE HQ---D---- --VDECRAMI
    PhCML19  SEFSRVFSAF ---------D RDDDGKISAA ELRLCMRAAL GED------- VSAEDADDLV ASADTD-GDG LLSQEEFLKL A---QLEAE- EEE------- ------RC-- ---------- ----RGLKEA FGMY-AMKGE ---GCITPLS LKRMLSKLGS HQ---E---- --IDECKAMI
    SiCML19  SEFSRVFSAF ---------D RDDDGKISAA ELRLCMKAAL GED------- VSAEDADELV ASADTD-GDG LLSQEEFLKL V---QLEAAA EEE------- ------RC-- ---------- ----RGLKEA FGMY-EMKGE ---GCITPLS LKRMLSKLGS HQ---E---- --IDECKAMI
    OsCML19  AEFRRVFSAF ---------D RDADGKISAA ELRLCMKAAL GED------- MSAEEAEALV SSADTD-DDG LLDEEEFTKL A--VQLEMGD EEE------- ------RC-- ---------- ----RGLMEA FRMY-EMEGE ---GRITPAS LKRMLSKLGS HQ---G---- --IEECQTMI
    SbCML19  SEFSRVFSAL ---------D RDGDGKLSAT ELRLFMKAAL GED------- VSSEEADRLV ASADAD-GDG LLSQEELLAL A--LAGTAAE EEEEEEEGG- -----ERR-- ---------- ----RELREA FGMY-AMEGQ ---GCITPLS LKQMLSRLGS HQ---D---- --IDECRAMI
    ZmCML19  SEFSRVFSAL ---------D RDGDGKLSAA ELRACMRAAL GED------- VSAEEADRLV ASADGD-GDG LLSQEELLAL A----GTTAA EEEEEEE--- ------RR-- ---------- ----RGLREA FRMY-AVEGQ ---GCITPLS LKRMLARLGS HQ---D---- --VAECTAMI
    BdCML23  ADFRSIFASF ---------D QDGDGKVSAA ELRLCVQAAL SGGADD---- MSAEEVQALM ASADTD-GDG LLDEEEFVRL V--QDHI--- ---HKEEG-- -----DRC-- ---------- ----RSLREA FGMY-EMEGK ---GCITSLS LKLMMSRLGL PL---D---- --VDECQAMI
    SiCML23  GELRRVFASF ---------D QDGDGKISAA ELRLCMKAAT G---ED---- MSAEDVRALM ASADAD-GDG LLDEEEFVRL A--GEVVEAD ADDDEEEE-- -----ERR-- ---------- ----RWLREA FGMY-EMEGR ---GCITALS LKLMLGKLGA HR---D---- --IADCQAMI
    OsCML23  DEFRRVFGSF ---------D QDGDGKISAT ELRLCVKASL G---ED---- MPDEEVQALM ALADTD-GDG LLDEEEFVRL V--TEMEADG DEEEDDDD-- -----ETC-- ---------- ----RCLREA FAMY-EMEGR ---GCITPLS LKLMLSKLGT HL---D---- --VAECQAMI
    OsCML25  SSASSVFAAF ---------D KDGDGKVSAS ELRGCMAAAL GEE------- VSEEEAAAIL ATADTD-GDG LLDHHEFMRL S--AAHQLQE PAEE------ ------SL-- ---------- ----RCLREA FDMYAEEEET ---AVITPAS LRRMLRRLGS EHQRLE---- --MEECRAMI
    OsCML26  SSASSVFAAF ---------D KDGDGKVSAS ELRGCMAAAL GEE------- VSEEEAAAIL ATADTD-GDG LLDHHEFMRL S--AAHQLQE PAEE------ ------SL-- ---------- ----RCLREA FDMYAEEEET ---AVITPAS LRRMLRRLGS EHQRLE---- --MEECRAMI
  SlCML39-1                      M ELRRCMKAVG G--------E ITVKEAEMVV RLSDSD-GDG LLGFEDFTKL M--EGMEE-- ---------- ---KRNKE-- ---------- ----SELMGA FGMYEME--- ---GYITPKS LKMMLSRLG- --ESTS---- --IDKCKVMI
    AcCML21  RNCKTIFEQF ---------D EDSNGAIDHE ELKHCFHK-- -------LEI NSTEEEISDL FEACDINENM GMKFNEFIVL LCLVHLLKDD PTVH---HA- ---------- -KTKMGLPNL EATFETLVGA FVLLDKNKDG ----YVSKSE MVQAIN---- ETTSGERSS- --GRIAMKRF
    MtCML21  RKCRAIFEQF ---------D EDSNGTIDQE ELKKCFSK-- -------LEI SFSEEETNDL FEACDINDDL GMKFSEFIVL LCLVYLLKDD PAAL---HA- ---------- -KSRIGMPNL EATFETLVDT FVFLDKNKDG ----YVSKNE MVQAIN---- ETTSGERSS- --GRIAMKRF
    VvCML21  RNCKAIFQQF ---------D EDSNGAIDHE ELKRCFLK-- -------LEI SFSEEEINDL FEACDINEDM GMKFNEFIVL LCLVYLLKED PAAL---QA- ---------- -KSRMGMPNL EATFETLVDA FVFLDKNKDG ----YVSKSE MVHAIN---- ETTAGERSS- --GRIAMKRF
    CpCML21  RNCKAIFEKF ---------D EDSNGAIDHE ELKKCFQK-- -------LEI SFTEEEIDDL FQACDINEDM GMKFNEFIVL LCLVYLLKDD PAAL---HS- ---------- -KSRMGMPKL EATFETLVDT FVFLDQNKDG ----YVSKNE MVQAIN---- E--SGERSS- --GRIALKRF
    TcCML21  RKCKAIFEQF ---------D EDSNGAIDHE ELKKCLNK-- -------LEV SFTEEEINDL FNACDINEDM GMKFNEFIVL LCLVHLFKDN ATSL---EA- ---------- -KSRMGMQNL EATFETLVDA FVFLDKNKDG ----YVSKNE MVQAIN---- E--SGERSS- --GRIAMKRF
    CcCML21  RNCKAIFEKF ---------D EDSNGTIDHE ELKKCFHK-- -------LEI KFTEEEINDL FEACDINKDM GMKFNEFIVL LCLVYLLKDD PTAL---RA- ---------- -KSRMGMPKL EATFETLVDA FVFLDKNKDG ----YVSRSE MTQAVT---- E--SGEGST- --GRIAIKRF
    CsCML21  RNCKAIFEKF ---------D EDSNGTIDHE ELKKCFHK-- -------LEI KFTEEEINDL FEACDINKDM GMKFNEFIVL LCLVYLLKDD PTAL---RA- ---------- -KSRMGMPKL EATFETLVDA FVFLDKNKDG ----YVSRSE MTQAVT---- E--SGEGST- --GRIAIKRF
    FvCML21  RNCKDIFQQF ---------D EDSNGAIDHD ELKRAFSK-- -------LEV SFTDEEIDDL FAACDINEDM GINFNEFIVL ICVVFLLKDD QNAG---EK- ---------- -KVDMGMPKL EATFETLVDA FVFLDKNKDG ----YVSKSE MVQAIN---- ETTTGERSS- --GRIAMKRF
   PperCML21  RKCKAIFEQF ---------D EDSNGAIDQE EMKKCFYK-- -------LEV SFTDEEISDL FEACDINEDM GIKFNEFIVL ICLVYLLKDD SNDV---HT- ---------- -KLHMEMPDL EATFETLVDA FVFLDKNKDG ----YVSKSE MVQAIN---- ETTTGERSS- --GRIAMKRF
    MgCML21  RKCKTTFDEY ---------D EDKNGTIDLQ ELKHCFRK-- -------LEI SFTEEEINDL FQACDINEDM TMKFNEFIVL ICLVYLLKED PSDK---DA- ---------- -KSQMGMPNL KATFETLVDT FVFLDKNKDG ----YVSKSE MVDAIN---- ETTSGERSS- --GRIAMKRF
    SlCML21  RKCKVIFQEF ---------D EDSNGAIDPQ ELKHAFNK-- -------LEI NFTDEEISDL FEACDINEDM GIEFSEFIVL LCLVYLLKDD PSAL---HA- ---------- -KSRMGLPNL EVTFETLVDA FVFLDKNKDG ----YVSRNE MVQAIN---- ETISGERSS- --GRIAMRRF
    StCML21  RKCKVIFQEF ---------D EDSNGAIDPQ ELKHAFNK-- -------LEI NFTDEEISDL FEACDINEDM GIEFSEFIVL LCLVYLLKDD PSAL---HA- ---------- -KSRMGLPNL EVTFETLVDA FVFLDKNKDG ----YVSRNE MVQAIN---- ETISGERSS- --GRIAMRRF
    CrCML21  RNCKAIFQEF ---------D EDSNGSIDHT ELKNCIRK-- -------LEI SFEEDEINDL FKACDINEDM GITFTEFIVL LCLVYLLKDD SSTL---QK- ---------- -KWTMGMPKL EPTFETLVDT FVFLDENKDG ----YVCREE MVRAID---- E--SGERSS- --GRIAMKRF
    AtCML21  RNCKAIFQEF ---------D EDSNGSIDHT ELKNCIRK-- -------LEI SFDEEEINDL FKACDINEDM GITFTEFIVL LCLVYLLKDD SSTL---QK- ---------- -KWTMGMPKL EPTFETLVDT FVFLDENKDG ----YVSREE MVRAID---- E--SGERSS- --GRIAMKRF
    ThCML21  RNCKAIFQEF ---------D EDSNGSIDHT ELKNCFRK-- -------LEI SFDEEEINDL FEACDINEDM GITFTEFIVL LCLVYLLKDD SSTL---QK- ---------- -KWRLGMPKL EPTFETLVDT FVFLDENKDG ----YVSREE MVRAID---- E--SGERSS- --GRIAMKRF
   CsatCML21  RNCKTIFQQF ---------D EDLNGIIDRR ELKKCFDG-- -------LEI LLTEEEIDDL FDACDISTAM GMKFNEFIVL LCLVYLLKDD PNAV---FS- ---------- -KSQFGMPKL EQTFESLVDA FVFLDKNKDG ----YVSKSE MVSAIN---- ETTSGERSS- --GRIAMRRF
    SiCML33  RNCRTIFQQF ---------D EDSNGEIDQQ ELKHCFQK-- -------LGI ESTDDEIKDL FEACDIYEHM GMKFNEFIVF LCLVYLL-ND PAVS---EA- ---------- -RKRMGLGSL EPTFETLVES FVFLDKNKDG ----YVSKNE MIQAIN---- ETTAGERSS- --GRIGVKRF
    SbCML33  RNCRTIFQQF ---------D EDSNGEIDQL ELKHCFQK-- -------LGI ESTDEEIKAL FQACDIYEHM GMKFNEFIVF LCLVYLL-ND PAVS---EA- ---------- -RKRMGLGNL EPTFETLVES FVFLDKNKDG ----YVSKSE MIQAIN---- ETIGGERSS- --GRIGVKRF
    OsCML33  RNCRIIFQQF ---------D EDSNGEIDQQ ELKHCFQK-- -------LDI SFTDEEIKDL FQACDIYENM GMKFNEFIVF LCLVYLL-ND PLVS---EA- ---------- -RKRMGLGSL EPTFETLVDS FVFLDKNKDG ----YVSKNE MIQAIN---- ETIGGERSS- --GRIGMKRF
    SiCML29  RKCKTIFEQF ---------D EDSNGEIDKE ELKHCFQK-- -------LEI SFTEEEISDL FEACDINEDM GMKFNEFIVF LCLVYLL-NE PAVS---EA- ---------- -KIKMGLGNL EATFETLVDA FVFLDKNKDG ----YVSKDE MIQAIT---- ETTTGERSS- --GRIAMKRF
    SbCML29  RKCKTIFEQF ---------D EDSNGEIDKE ELKHCFQK-- -------LEI SFTEEEISDL FEACDINEDM GMKFNEFIVF LCLVYLL-NE PAVS---EA- ---------- -KIKMGLGNL EATFETLVDA FVFLDKNKDG ----YVSKDE MIQAIN---- ETTTGERSS- --GRIAMKRF
    OsCML29  RKCKIIFEQF ---------D EDSNGEIDKQ ELKHCFQK-- -------LEI SFTEEEINDL FEACDINEDM GMKFNEFIVF LCLIYLL-NE PAVS---EA- ---------- -KIKMGLGNL EATFETLVDA FVFLDKNKDG ----YVSKEE MVQSMN---- ETATGERSS- --GRIAMKRF
    PaCML21  ENVRDVFQKF ---------D KDSSGTIDLE ELKECFRE-- -------LQV SFTDEEITEL HQICDMDESK GIEFKEFIVL LCLVYIL-WE PTDP---NS- ---------- -KSRIGLPHL EATFDSIIDA FRFFDKDGDG ----YVSKKE ILQALN---- QASPGGRAA- --DQIGIKRF
    PpCML21  EAVREVFNKA ---------D KNGDGSLDME ELKQCLQE-- -------LQV EYSDQEIEEF HHESDMDASH GIQFKEFIVV LALIYLL-GR PTHT---GV- ---------- -ESAIGLPDL EATFDEIAEA FVFFDRNKDG ----YVSKKE MIWAIN---- EASPSGRHG- --HRIGVQRF
    AcCML22  RKIKGVFERY ---------D EDSNGTIDRE ELKKCLET-- -------LQL HLTGKEIEDL FQSCDVDGEE GIQFNEFIVL LCLIYLLMEP SSP----HP- ---------- -TSKMGS-EL EATFNTIVEA FLFFDKNGDG ----KLNRKE MVKAMN---- EASPWERSP- --GHITKTRF
    CsCML22  KNIKSVFEQY ---------D EDSNGTIDRE ELKKCLEE-- -------LQL HLKEEEIEDL FKSCDIDGSE GIQFNEFIVL LCLIYLLTTP SSS-----S- ---------- -MSKMGSPQL KATFDAIVEV FLFLDKNGDG ----RLNKKD MVKTLN---- ESYPREKSP- --AHVTRTRF
    TcCML22  KNLRGVFEQY ---------D EDSNGTIDRE ELNKCLEK-- -------LQF HLTKEEVEDL FHSCDIDGSE GIQFNEFIVL LCLIYLLMKP SPSPDTVST- ---------- -TLKIDSPQL EATFDTIVEA FLFLDKNGDG ----KLNKKD MIKALN---- EASPWERSP- --AHVTRTRF
   PperCML12  KKIRDVFEQY ---------D EDSNGTIDLE ELKKCLQK-- -------LEL HLTAEEIEDL FHSCDMDGSE GIQFNEFIVL LCLIYLLKEP SSS----HN- ---------- -TSKIDSPQL EATFDTVIEV FSFLDKNGDG ----KLNKTD MVKALN---- EASPLEKSP- --AHVTRTRF
    FvCML22  KNIRNVFDLY ---------D EDSNGTIDRE ELKKCLQK-- -------LQL HLTAEEIEDL FHSCDMDGSE GIQFNEFIVL LCLIYLLKDP N-------T- ---------- -TSKIDCPQL EATFDTVIEV FVFLDKNGDG ----KLNKTE VVKALN---- EASPREKSP- --SHVSRNRF
   CsatCML22  QNIRGVFEQY ---------D EDSNGSIDRE ELKKCLQQ-- -------LQM HMTEEEVEDL FHSCDIDGSA GIQFNEFIVL LCLIYLLKDE HSL-----T- ---------- -KSKLGSPQL EATFDTIIQA FIFLDKNGDG ----KLNRKE MVKALN---- EASPYERSP- --ARITKTRF
    MgCML22  KEIRQVFEKY ---------D EDSNGTIDND ELKKCLHA-- -------LEF SVKDDEIDDL FLYCDMDENE GIQFKEFIVL LCLNYLLTDS STSSS--HA- ---------- -TLKMCSPKI EATFNTIVEV FLFLDKTGDG ----KLNRKD VVKALN---- EASPKEKSP- --SHITRTRF
    StCML22  KEIKGVFEQF ---------D EDSNGTIDRD ELKKCVKK-- -------LHF HVKDEEVNDL FDSCDVDEKE GIQFNEFIVL LCLLYLLIDS SSSS---EN- ---------- -TSKIGSPEL EATFNTIVEA FLFLDKNGDG ----KLHKKD VLKALN---- DECPCEKSP- --SHVTRTRF
    ThCML22  RNIRSVFESY ---------D KDGNGTIDME ELKKCLEE-- -------LKL SLSEEEVKGL YGWCDVDGSK GIEFNEFIVL LCLIYLLAKP SSESS--LE- ---------- -SREMGPKLV ESVFDPIVEV FLFLDKDGKG ----KLNKAD VIKTLN---N EDYPLERSP- --SHVTNMRF
    AtCML22  RNIRSVFESY ---------D NDTNGTIDIE ELKKCLEE-- -------LKL SLSDEEVKGL YSWCDVDGSK GIQFNEFIVL LCLIYLLAKP SSESS--TE- ---------- -SREMGPKLV ESIFDPIVEV FLFLDKDGKG ----KLNKAD VIKTLN---N EDYPLERSP- --SHVTNMRF
    BdCML32  RDIRDVFDHY ---------D VDSNGTIDNE ELRSCMSK-- -------LQV QMSEKEVDDV HRYCDVNSRK GIQFQEFVVL LCLMYLLFGP GVT----RQ- ---------- -VSEFESAKL NYVFDELIDA FLFFNKDGDG ----KMRRKD VTQRMN---- EASHQERTP- --SHITTQLF
```

```
   SbCML34  RDIKEIFDQY ---------D EDSNGTIDNE ELQSFLSK-- -------LQV KMSQQEIDNL HSYCDIDSRN GIQFQEFVVL LCLMYLLFGP DVT----RR- ---------- -VSEFESVKL NYVFDELIDA FIFFDKDGDG ----KMKKRD VTHRMN---- EASHQERTP- --SHITAQLF
   OsCML34  RNIRDVFDQY ---------D EDSNGTIDNE ELRNCLNK-- -------LQV QMSEEEIDNI HRYCDIDNRK GIQFPEFVVF LCLMYLLFGS DVT----YR- ---------- -VSEFESARL NYVFDELIDA FLFFDKDGNG ----KMKRKD VTQRMN---- EATHQERTP- --SHITSQLF
 SmCML21-1  DQVRDVFKRF ASVGWILVAD KHKRGTIDLE QLKACFRE-- -------LKV EFTEEEVQIF YEEGDVDHNR RISFKEFIVV LALAYLLGE- ---PLNSDG- ---------- -KSRIGLPDL EWSFETIEDA FVFFDKNGDG ----YVTKEE MIESIH---E SSHATNTQQ- --DSIGVERF
 SmCML21-2  EQVRGVFKKF ---------D KDDSGTIDLE ELKTCFRE-- -------LQV EFTDTEVKAF HEESDMDKSK GVDFKEFIIV LALVYLLGET GAKPVKQKEE DKGKGSQLKN LKSRIGLAEL ESTFETIVDT FRFFDKDGDG ----YVSRKE MITAIN---E ASPGQSSSG- --ESIGVKRF
   MpCML12  EALRGIFDRV ---------D KDANGFIEKH EFVTAVEE-- ----GFSRKV TVEREMIERI YDEADLKHDG RVNYKEFVLM IVLLYLVSWE TDLSVGKDTA VS-------- -GSAGEDTLV HNAVQKVLEA FLFFDANGDG ----YIRKDE VLKKLE---A HEGAHAKQKT EHGGLTAARF
 BrCML32-1           M ---------D TENSGAITFD ELKAGLRKYG ST-------- -LKDTKIHDL MEAADVDNSG TIDYSEFIAA TIHLNKLERE ---------- ---------- ---------- ----EHLVAA FQFFDKDGSG ----YITIDV LQQACVE--- ------HRMT DV-F-LEDII
   PvCML36  TGLKEMFKNI ---------D KDNSGTITLD ELKNGLAKHG TK-------- -LSDGEIQQL MEAADADGNG LIDYDEFVTA TVHMNKLDRE ---------- ---------- ---------- ----EHLYTA FQYFDKDNSG ----YITREE LEQALKE--- ------QGLY DADK-IKEVI
   PvCML29  AGLKEMFKMM ---------D TDNSGQINFE ELKAGLERVG AN-------- -MKESEIYQL MQAADIDNSG TIDYGEFIAA TLHLNKVEKE ---------- ---------- ---------- ----DHLFAA FQYFDKDGSG ----YITADE LQQACDE--- ------FGIE DV-R-LEDMI
 ZmCML36-1  AGLKEMFMAM ---------D TDNSGAITYD ELKEGLRKYG ST-------- -LKDTEIRDL MEAADIDNSG TIDYIEFIAA TLHLNKLERE ---------- ---------- ---------- ----EHLVAA FSYFDKDGSG ----YITVDE LQQACKE--- ------HNMP AA-F-LDDVI
   MeCML32  EDIKEMFRKM ---------D TDNDGIVSIE GLKAGLRNFN SQ-------- -LAESEVQML IEAVDTNGKG TLDYGEFVAV SLHLQRMAND ---------- ---------- ---------- ----EHLRKA FSYFDKDGNG ----YIEPDE LRDALME--- ------DGAD DCSNVANDIF
 ZmCML36-3  EAIRELFQLL ---------D TNKDGHLTIE ELRKGMRLIG HN-------- -VHDTDVDML MEAADIDGNG TLDCKEFVTV SIHLKKIRSE ---------- ---------- ---------- ----DHLPKV FSYFDKNGSG ----YIEIEE LKEALSP--- ------RGDQ ---KAIEDII
 ZmCML36-2       MFKAM ---------D NGNNVVITLS ELKEGLSKCG SV-------- -FRNIEISDI VEADDYDGNT NINWEEFIAV TVPLNKTEHK ---------- ---------- ---------- ----EHLMAA LTYFDKDGGD ----YITVDE LQKASVE--- ------HEMK NA--FLEDTI
   MpCML11  DALRDIFAQF ---------D RDQSGNITHD ELKHVVKNLD GTGFISPSEL RYLRENIDDI IADIDKDGDG EINYGEFVDM MIHLKEQFKQ ---------- ---------- ---------- ----QDMREL FRTFDKDNSG ----SITAAE LRKAVSQLDG IGLMSVMEQR EMMENLDAMI
CreinCML20  AWAEMDLDGD GKVSAADLRR YFGRVQPGLL AFLPSIFRAI ESRSFASGRS ASARPGSRGA GGSGGAGGGG GGGGGAITFQ QFLKALYPEA SLSDLRTLQA MA-ADRKSR- AVAAAEAVAN DKLLAEIQGV FVVFDDDQSG ----ELDEDE FVSAME---- -LTGHTAAEA KGIFQQIDAD
CreinCML20  AVAAYNANHN ----LPALLR NIHKLQPALL SAVCTQL--- ------SGGA GAASPFPLES LGALIAMAAP GVTIPSLEVE RLRQLLVLDT GVHTMQPADL VTQVGLCAD- AIKRAAAISR MVLEAKSQNI PVQLDTDLGG ----AEIVLQ RMSDML---- -VEGNGQQIA EA-FKRFDKD
   AtCML48  YAPSAPELPE SFV-QQQHDG ESRYTYAYPS YQPTQQFSS- ----YSGMFS PETHPEIVRS FESADRNRSG FLEESELRQA LSLSGYDGIS NRTIRLLLFI YKIPVDSLL- RLGPKEYVEL WNCLAQWRAI FNRYDRDRSG ----KMNSTQ LRDAFY---- -NLGCVLPTS VHQLIVSQFD
   AtCML49  PQQSGHG--G GYGGAPQQ-- -SGHGGGYGA PPPQASYGSP FASLVPSAFP PGTDPNIVAC FQAADRDNSG FIDDKELQGA LS-SYNQSFS IRTVHLLMYL FTN--SNVR- KIGPKEFTSL FFSLQNWRSI FERFDKDRSG ----RIDTNE LRDALM---- -SLGFSVSPV ILDLLVSKFD
   AtCML50  TPPHGVSDYG SYGGAPPRPA SSGHGGGYGG YPPQASYGSP FASLIPSGFA PGTDPNIVAC FQAADQDGSG FIDDKELQGA LS-SYQQRFS MRTVHLLMYL FTN--SNAM- KIGPKEFTAL FYSLQNWRSI FERSDKDRSG ----RIDVNE LRDALL---- -SLGFSVSPV VLDLLVSKFD
CreinCML32  PKAPAAAPVV EVPAAEPVAA PAAAAPEEPV VDEGPSLESL KLAAISAVAG ATDSPGVRDA LTTLVKSLQT ASTAAAIASW ETPAGAAAAS GSAAAATGAA ESPYAASLA- ALTAALKLRG THGIISIGKK FRSMDDSGDH ----KLTYDE FKKALV---- -EMKLGLSET D----MSRLF
   RcCML23  SKAPTKRCLM VKQRLSRLKE YGVRTDTKTK YTAGIMLLSL IPVILVELAS AFESRPWSHI VTLVVVGAAL VSYFLFLSRR QW-IQERSLE YSREQLLLAG FLDHLQKFA- KRRLVNK-EG KVDVSCVKRT FRNIDKNNDN ----HISQKE LKDFLK---- -HMKSGDLEF DDAFAVQELM
    SbCML6  PGMEAAPVPE DLT------- DGEADADEDY INDAARFN-- VTHRVEALFP KIDVDPADGA VTGAELAAWN LANARREVLH RTARELELHD RDHDGRVAFG EYER-PSWA- WRFDDHNSTN DGVGWWKEEH FNAADMDGDG ----FLNLTE FNDFL----- -HPADTTNPK LINWLCKEEV
   SbCML15  PDIDTFRDKL DRLLPDWSRF DGELGPMHRY FGLDGPLD-- VRQRLAYLFV ILDRSPRDGG VGVDELEAWL RRQAVARLDA VTRREMARHD RDRNGAVTLR EF-------- --FADWSNMG HGKMARWMDK FASADRNGDG ----SLNAVE FNDFL----- -HPEDTSQES VMLWLLKDKL
    VcCML5                                                              MGAGCSTES SAETEQPRFP PTQSVSIERT KSGKPSEEEA APAPRPKLSG PPVEVDLKE- VERDHGDRDQ AARLDALRRC FEALDKDKSG ----SIDAKE LKQYL----- --FKTGVNVS HISDKAEELM
   AcCML18
   MeCML18
   RcCML18
   SlCML18
 StCML18-1
   CpCML18
   MgCML18
 PtCML18-1
 PtCML18-2
   CcCML18
   CsCML17
 FvCML18-2
 PperCML18
   MdCML18
   TcCML18
   EgCML17
 StCML18-2
 GrCML18-2
   GmCML17
 PvulCML18
   MtCML18
 CsatCML18
 GrCML18-1
   BdCML11
   PhCML11
   SbCML11
   ZmCML11
   OsCML11
   GmCML18
   BrCML18
   ThCML18
   CrCML18
   AtCML18
 LuCML18-1
   BrCML17
   CrCML17
   AtCML17
   ThCML17
   SlCML17
 FvCML18-1
 LuCML18-2
   EgCML18
 MdCML17-3
 MdCML17-2
 PpCML17-1
 PpCML17-2
   AcCML16
   FvCML15                                                                                                                      MATTSQTLP NYQPAGESLS TNPIPEAVPI SSSGSIGPFF ---AVISVLT ILAFLSCVLG RILTRDQVVL TPLEGIRDQR
   BdCML12
   PhCML12
   PvCML12
   SiCML12
   SbCML12
 ZmCML14-1
   OsCML12
   BdCML14
   PhCML14
 PvCML14-1
 PvCML14-2
   SiCML14
   SbCML14
 ZmCML14-2
   OsCML14
 BrCML16-1
 BrCML16-2
   ThCML16
   AtCML16
   CrCML16
   CpCML16
 MeCML16-1
   PtCML16
   RcCML16
   MgCML26
 PperCML16
 VvCML16-2
   CcCML16
   CsCML16
 LuCML15-2
 MeCML16-2
   SlCML16
   GmCML16
 PvulCML16
 EgCML15-2
   CpCML15
   CcCML15
   CsCML15
 CsatCML15
 MdCML15-2
 PperCML15
 MdCML15-1
 GrCML15-2
 GrCML15-3
   TcCML15
   MeCML15
   RcCML15
   PtCML15
 VvCML16-1
   SlCML15
   StCML15
   BrCML15
   CrCML15
   AtCML15
   ThCML15
 CsatCML16
   GrCML16
 EgCML15-1
 GmCML15-2
 GmCML15-3
 PvulCML15
 GmCML15-1
   MtCML15
 GrCML15-1
   FvCML16
   LuCML15
   AcCML20
 GrCML20-2
   FvCML20
 MdCML17-1
   MdCML20
 PperCML20
   CcCML20
   CsCML20
   CpCML20
   PtCML20
 MeCML20-1
   CrCML20
   AtCML20
   ThCML20
 CsatCML20
   SlCML20
 GrCML20-1
   EgCML20
   StCML20
   GmCML20
 PvulCML20
   MtCML20
```

```
  VvCML20-1
  VvCML20-2
     BdCML8
     SbCML8
   ZmCML8-2
   PhCML8-1
   PvCML8-2
    ThCML19
    AtCML19
  MeCML20-2
  MpCML20-2
    SmCML20
    OlCML20
  VcCML20-1
  VcCML20-2
   PhCML8-2
   PvCML8-1
   PvCML8-3
   ZmCML8-1
     OsCML8
    SmCML19
  MpCML20-1
    AcCML11
  GmCML11-3
  GmCML11-4
  MeCML11-2
  MeCML11-1
PperCML11-
  GrCML11-3
  BrCML11-2
  BrCML11-3
    CrCML11
    AtCML11
    ThCML11
  BrCML11-1
    FvCML11
PperCML11-
  GrCML11-2
  GmCML11-1
  GmCML11-2
  PvulCML11
  PtCML11-1
     BdCML4
     OsCML4
     PhCML4
     PvCML4
     ZmCML4
     PhCML5
   PvCML5-2
   PvCML5-1
     SiCML5
     ZmCML5
     SbCML5
     OsCML5
    EgCML11
     BdCML1
     BdCML3
     OsCML2
     OsCML3
  CsatCML50
     SiCML2
     ZmCML2
  GrCML11-1
  PtCML11-2
  PtCML11-3
PperCML11-
     BdCML5
     OsCML6
    MdCML11
    AtCML13
    AtCML14
  BrCML34-2
  BrCML34-1
    CrCML34
    AtCML34
    ThCML34
  BrCML32-2
    CrCML32
  CrCML32-2
  CrCML32-1
    AtCML32
    AtCML31
    ThCML32
    ThCML33
    AtCML33
  EgCML25-2
    SmCML25
  LuCML25-1
  AcCML25-3
     BdCML2                                                                                                          MDELS QEQLDEFRAA FSLFDRDGDG ---NLYTSSS VPSSSSSSVP WMKGLIWAGT ITLEELGTVM
    CrCML12
    AtCML12                                                                                                         MADKLT DDQITEYRES FRLFDKNGDG ---S------ ---------- ---------- ITKKELGTMM
  CsubCML24                                                                                                                MKNSDF FKQFDTDGDG ---Y------ ---------- ---------- ISFTEYLMIV
      PpCML7                                                                                                                                                                     MGCYGSK
  MpCML21-1
     MpCML4
    MpCML30
    MpCML15
    MtCML46
    AtCML45
    AtCML46
    AtCML47
     VcCML4
   OsCML1-3
    AtCML44
    OlCML11
     AtCML1
 CsubCML20
    SiCML36
    SbCML50
    MtCML11                                                                                                                                                                        MCPSD
  Consensus  ......f..f          d ...dg.i... el........ . ........ ....e..... ...d....dg .....ef... .  ....... ....    ..    ........ .....        .......a f...d....g     .i...e l...l..l..  .....    . .....c..mi

             361                                                                                                                                                                         540
   AcCML3-1  KKVDMDGDGM VNFDEFKHMM KTGGKLISVS
     CcCML7  RKVDVDGDGM VNFDEFRRMM KAGGVLLTAF
     CsCML7  RKVDVDGDGM VNFDEFRRMM KAGGVLLTAF
   GrCML5-1  RKVDIDGDGM VNFDEFKTMM KNGGRLVSAF
   GrCML5-3  RKVDIDGDGM VNFDEFKKMM RSGGGLVSVS AF
   TcCML5-1  RKVDMDGDGM VSFDEFKRMM KSGGGLVSVS AW
   MeCML3-1  RKVDMDGDGM VNFDEFKRMM MMSGSTKLTP LC
     MeCML5  RKVDMDGDGM VNFDEFKRMM R-NGTSNLIS VC
   PtCML3-3  RKVDMDGDGM VNFDEFKRMM MRGGSKLVSV F
   PtCML3-4  RKVDMDGDGM VNFDEFKRMM TRGGSKLVSV F
     RcCML3  KKVDMDGDGM VNFDEFKKMM -RSRTLHAHA FTDF
     LuCML5  RRVDVDGDGM VNFDEFKTMM RNGGGNKLLL PGLC
   LuCML3-3  RRVDVDGDGM VNFDEFKMMM RSGGG-KLLL PVF
   LuCML3-2  RRVDVDGDGM VNFDEFKMMM RSGGG-KLLL PVF
   GmCML5-1  KKVDMDGDGM VNFNEFKRMM MNGGKFFFNA
   GmCML5-2  KKVDMDGDGM VNFNEFKRMM MNGGKLAFNA
 PvulCML3-1  KKVDMDGDGM VNFKEFKRMM MKGPKLNA
     MtCML7  RKVDMDGDGN VNFNEFKRMM KNGGANLFAT
   CpCML3-1  RKVDVDGDGM VNFDEFKKMM KRGVAGGRLP SVSN
   CsatCML5  RKVDLDGDGM VNFDEFKKMM RNGVTILTSS
     SlCML5  RNVDVDGDGM VNFDEFKKMM RNGGRLIPIP
     StCML5  RNVDVDGDGM VNFDEFKKMM KNGGRLIPIL RGNSIMLQD
     FvCML5  KKVDKDGDGM VNFDEFKMMM KGG---RLLT AF
   MdCML3-1  RKVDRDGDGM VNFDEFKMMM KGG---GLLL AH
   MdCML3-4  RKVDRDGDGM VNFDEFKMMM KGG---GLLL AH
 PperCML5-2  KKVDRDGDGM VNFDEFKRMM KGGAGGRLLL AH
   EgCML5-1  RKVDADGDGM VNFDEFKRMM KG
   EgCML5-4  RKVDVDGDGM VNFDEFKRMM RGGQALLVSA
     MgCML4  RRVDVDGDGM VDFDEFRNMM KSGFARLVSV S
  LuCML30-3  RRVDVDGDGM VNFDEFKTMM SRNNDFGSRS EMEYVLTLAS
   StCML3-1  KKVDVDGDGM VNFDEFKKMM KACGTLVPFS
    BdCML30  RLVDSDGDGM VNFEEFKRMM TVVKA
    OsCML30  RLVDSDGDGM VSFEEFKRMM TVVKA
    PhCML30  RLVDSDGDGM VSFEEFKRMM TVVKA
    SbCML30  RLVDSDGDGM VSFEEFKRMM TVVNVKA
    ZmCML30  RLVDSDGDGM VSFEEFKRMM AVVKA
    SiCML30  RLVDSDGDGM VSFEEFKRMM TVVKA
   AcCML3-2  MKVDVDGDGM VNFKEFKQMM RGGGFAALT
     CpCML5  MQVDEDGDGM VNYKEFRQMM KGGGFSALS
   MgCML3-2  MRVDADGDGM VNFTEFKQMM RGGGFAALGN
   PtCML3-1  MKVDVDGDGM VDYKEFKKMM KGGGFSALG
   PtCML3-2  MKVDVDGDGM VDYKEFKKMM KGGGLVLWG
     PtCML5  MKVDVDGDGM VDYREFKKMM KGGGFSAVG
 PperCML5-1  MKVDVDGDGR VNYKEFKQMM KGGGFSALS
   StCML3-3  NKVDIDGDGM VNFAEFKQMM RGGGFAALS
```

```
   GrCML3-2  TKVDVDGDGR VNFMEFKQMM KGGGFTAMA
   TcCML5-2  MKVDVDGDGR VNFKEFKQMM KGGGFSALT
     CcCML3  MKVDVDGDGM VDYKEFKQMM KGGGFSALT
   CsCML3-1  MKVDVDGDGM VDYKEFKQMM KGGGFSALT
   EgCML5-3  MKVDVDGDGM VDFKEFKQMM KGGGFSALS
   GmCML5-3  SKVDVDGDGM VDFKEFKQMM KGGGFSALT
 PvulCML3-2  MKVDVDGDGM VDYKEFKQMM KGGGFSALT
     MtCML5  GTVDVDGNGL VDYKEFKQMM KGGGFTALS
     FvCML7  MKVDVDGDGM VNYNEFRQMM KGGGFGALT
   BrCML4-1  MQVDEDGDGR VNYKEFLQMM KTGGFSNTSS SN
     CrCML4  MQVDEDGDGR VDYKEFLQMM KSGGFSNRA
   BrCML4-2  TQVDEDGDGR VNYKEFLQMM KSGGFSNRSS
     ThCML4  TQVDEDGDGR VNYKEFLQMM KSGGFSNNRS SSN
     AtCML4  MQVDEDGDGR VNYKEFLQMM KSGDFSNRS
   CrCML5-1  MQVDADGDGR VNYKEFLQMM KGGGFSSSSN
     AtCML5  MQVDADGDGR VNYKEFLQMM KGGGFSSSN
   ThCML5-1  MQVDADGDGR VNYKEFLQMM KGGGFSSSN
   BrCML5-1  MQVDGDGDGR VNYKEFLQMM KGGGFSCSN
   BrCML5-2  MQVDGDGDGR VNYKEFLQMM KGGGFSCSN
   BrCML3-1  GKVDVDGDGM VDFKEFKKMM KAGGFAAL
   BrCML3-2  GKVDADGDGM VNYKEFKQMM KGGGFAALES NL
     CrCML3  SKVDVDGDGM VNFKEFKQMM KGGGFAALGS NL
     ThCML3  SKVDVDGDGM VNFKEFKQMM KGGGFAALGS NL
     AtCML3  SKVDVDGDGM VNFKEFKQMM KGGGFAALGS NL
   CpCML3-2  TKVDVDGDGM VNFKEFKQMM KGGGFAALGS S
   PtCML3-5  KKVDVDGDGM VNFREFKQMM KGGGFAALGS S
     EgCML3  NKVDVDGDGM VNYMEFKQMM KGGGFAALGS S
   GrCML3-1  KKVDADGDGM VNFKEFKQMM KGGGFAALSS S
   CsatCML3  MKVDVDGDGM VNYKEFKQMM KGGGFSALG
   LuCML3-1  MKVDVDGDGM VNFKEFKQMM KGGGFAALSS S
     FvCML3  KKVDVDGDGR VNFKEFKQMM KGGGFAALTS N
   MdCML3-2  KKVDGDGDGR VNFKEFKQMM KGGGFAALSS N
   CsCML3-2  KKVDVDGDGM VNYNEFKQMM KGGGFAALR
   SlCML3-1  KKVDADGDGM VNFIEFKQMM KSGGFAALS
   StCML3-2  KKVDADGDGM VNFIEFKQMM KSGGFAALS
   MeCML3-2  MKVDVDGDGM VNFKEFKQMM KGCGFSALG
     SlCML7  MKVDVDGDGM VDFKEFRQMM KGGGFSLT
     StCML7  MKVDVDGDGM VDFKEFRQMM KGGGFSLT
   SlCML3-2  IKVDVDGDGM VDYKEFRQMM KRGGFSSLS
   GmCML3-1  TKVDVDGDGM VNYKEFRQMM KGGGFTALS
   GmCML3-2  MKVDVDGDGM VNYKEFRQMM KGGGFTVLS
   GmCML3-3  SKVDVDGDGM VDYKEFKQMM KGGGFTALT
 PvulCML3-3  VKVDIDGDGM VNYKEFRQMM KSGGFSGLG
   BrCML5-3  IQVDVDGDGR VDYTEFRQMM KKGRFFSSLS
   CrCML5-2  MQVDVDGDGR VNYMEFRQMM KKGRFFSSSS
     AtCML6  MQVDVDGDGR VNYMEFRQMM KKGRFFSSLS
   ThCML5-2  LQVDVDGDGR VNYKEFRQMM KKGRFFSSTS
     CrCML7  KQVDADGDGR VNYQEFRLMM KGGGFCALS
     AtCML7  KKVDVDGDGR VNYKEFRQMM KGGGFNSL
   GrCML5-2  MKVDVDGDGR VNFLEFKQMM KGGGFSAFS
     GrCML7  MQVDADGDGR VNFSEFKQMM KGGGLSALT
   BrCML2-1  SKVDVDGDGM VNFKEFKQMM RGGGFAALSS S
   BrCML2-2  SKVDVDGDGM VNFKEFKQMM RGGGFAALSS N
     CrCML2  SKVDVDGDGM VNFKEFKQMM RGGGFAALSS N
     ThCML2  SKVDVDGDGM VNFKEFKQMM RGGGFAALSS N
     AtCML2  SKVDVDGDGM VNFKEFKQMM RGGGFAALSS N
   GrCML3-3  TKVDADGDGR VNFKEFQQMM KGGGFAALSS S
   GmCML3-4  KKVDVDGDGM VDYKEFKQMM KAGGFAAASL S
   GmCML3-5  KKVDVDGDGM VNYREFKQMM KAGGFAAASL S
     MtCML3  KKVDVDGDGM VNFKEFQQMM KAGAFAADSL S
 PvulCML3-4  KKVDVDGDGM VNFNEFKQMM KDGGFVVHG
   MeCML3-3  KKLDVDGNGM VDFKEFKQMT KGGGFAALDS I
    BdCML28  SKVDADGDGR VDFKEFKQMM RGGGFSAIRG
    PhCML28  SKVDADGDGR VDFTEFKQMM RGGGFAALGR
    SiCML28  SKVDGDGDGR VDFTEFKQMM RGGGFAALGR
  PvCML28-1  SKVDADGDGR VDFMEFKQMM RGGGFAALGR
  PvCML28-2  SKVDADGDGR VDFTEFKQMM RGGGFAALGR
    SbCML28  SKVDADGDGR VDFTEFKQMM RGGGFAALGR
    ZmCML28  SKVDADGDGR VDFTEFKQMM RGGGFDALGT
    OsCML28  SMVDADGDGR VDFKEFKQMM RGGGFAALGG
     MgCML3  SKVDVDGDGR VNFDEFRQMM KGGGFAALS
     MgCML7  KKVDFDGDGR VNYDEFRQMM KGGGFAALSS
    BdCML22  GQVDRDGDGR VDFHEFLQMM RGGGFAALG
    PhCML17  GRVDRDGDGR VDFHEFRQMM RAGGLATLG
    SiCML22  GRVDRDGDGR VDFHEFRQMM RAGGLATLG
    SbCML22  GHVDRDGDGR VDFHEFRQMM RAGGLASLG
    OsCML22  GQVDRDGDGR VDFHEFLQMM RGGGFAALG
    ZmCML17  GRVDRDGDGR VDFREFRQMM RAGGLAALG
    OsCML17  GQVDRDGDGR VDFLEFKQMM RGGAFATLR
     SmCML5  KAVDADGDGQ VNFDEFKRMM ASNLLEK
     SmCML7  SSVDADGDHK VNFKEFRTMM SKALAM
    CcCML42  GSVDRNHDGR VDFFEFKNMM QSVLVRSS
    MgCML42  SSVDQNHDGR VDFFEFKHMM RTVIVPSS
    AtCML42  VSVDRNQDGR VDFFEFKNMM RTVVIPSS
    AtCML43  VSVDSNHDGR VDFFEFKNMM QTVVVPS
    OsCML32  CNVDRNSDGR VDFGEFKSMM QGITVWGP
  PaCML42-1  CNFDLDSNGL LDFFEFKVMM SRN
  PaCML42-2  CKFDLDSNGL LDFYEFKIMM KYKD
   PaCML5-2  CAYDSDSNGL LDFSEFKTMM TSNIPALPAD LQEP
   PaCML5-1  CRYDSDSNGL LDFSEFKN
   PaCML5-3  GTYDSDSNGL LDFSEFKIMM TSNIPGVSVS SPP
     SmCML4  RGVDSNGDGL VDFLEFKNMM
     SmCML3  RMVDSNGDGL VDFLEFKRMM
  AcCML25-1  SGVDVDGDGM INFEEFKVMM MGPQQEA
 PvulCML25-  AGVDGDGNGM INFEEFKIMM TGNK
    BrCML25  SGVDKDGDGT IDFEEFKVMM TMGSRRDNVM GGGAQW
    CrCML25  GGVDKDGDGT IDFEEFKIMM TMGSRRDNVM GGGAQR
    AtCML25  GGVDKDGDGT IDFEEFKIMM TMGSRRDNVM GGGPR
  BrCML25-1  GGVDKDGDGT IDFEEFKIMM TLGSRRDNVM GGGQR
    ThCML25  GGVDKDGDGT IDFEEFKVMM TMGSRRDNVM GGQR
  CpCML25-1  SGVDKDGDGT IDFEEFKVMM MMGSRYDAMG SHSQRSVSIK ID
  EgCML25-1  GGVDSDGDGM ISFEEFKVMM MVGSRYDSTS SGNTHNN
  MeCML25-1  SGVDSDGDGM IDFEEFKVMM TVGIKWDAIN GLG
  MeCML25-2  SGVDSDGNGM IDFEEFKVMM TVGVKWESNN GLS
  PtCML25-1  SGVDSDGDGM IDFEEFKVMM MMGARWDSMD ALKGIGR
  PtCML25-2  SGVDRDGDGM IDFEEFKVMM MMGARWDSMD TLRGTRG
  GrCML25-3  SGVDNDGNGM IDFEEFKVMM MAGATFVSMD SKRDVAV
  GrCML25-5  SGVDNDGNGM IDFEEFKVMM LAGPRYDSMD S
  TcCML25-3  SGVDSDGDGM IDFEEFKVMM MAGARYDSIE S
  MdCML25-3  GGVDSDGDGM INFEEFKVMM MTGSRYTDPV SG
  MdCML25-4  GGVDSDGDGM ISFEEFKVMM MTGSRYIDPA SG
 PperCML25-  SAVDSDGDGM INFEEFKVMM MMGSRYNDSA AV
  CsCML25-1  RGVDCDGDGT IDFEEFKVMM TAGSRYEFAD PVQGQVAV
 CsatCML25-  TGVDKNGDGM ISFDEFKVMM MSGSRSQ--G FNG
  MgCML25-1  SGVDADGNGT ICFDEFKVMM IKGSRFD--D VVKVD
  MtCML25-4  GGVDSDGDGM IDFEEFKKMM MGSNFGSMEG TNQVDSET
  SlCML25-1  SGVDCDGDGM INFEEFKIMM AKGSRFDVKG C
  StCML25-1  SGVDCDGDGM INFEEFKIMM AKGSRFDVTD ARIV
  MtCML25-3  GGVDSDGDGT IDFEEFRMMM MMGSRHDTTD RVKPEPMPTE
  PvulCML25  GGVDRDGDGT IDFEEFRVMM MMGSRHDTTD RVKPLPETS
  GrCML25-4  SGVDKDGNGM IDFEEFKVMM MAGARS
  SlCML25-2  GGVDCDGDGM IDFEEFKVMM IGKRK
  GrCML25-1  SGVDNDGNGM IDFQEFKVMM VAAHSIQPLN GLNL
  LuCML25-2  SGVDRDGNGT IDFEEFKMMM TMGSRWGSSE Q
  LuCML25-3  SGVDRDGNGT IDFDEFKMMM TVGSRWGSSE HDAVKQ
 CsatCML25-  SGVDSNGDGM ISFEEFKVMM STGLSVAGS
  PaCML25-9  TGVDRNGDGL INFEEFKTMM TTSSFSSSSK
    BdCML10  EGVDKNGDGL ISFEEFKVMM D-GGGG-FAK IA
  PvCML10-1  EGVDQNGDGL ISFEEFKVMM A-GGGC-FAK IA
  SbCML10-1  EGVDQNGDGL ISFEEFKVMM A-GGGC-FAK IA
  ZmCML10-1  EGVDQNGDGL ISFDEFKVMM ARGGAC-FAK IA
    OsCML10  EGVDQNGDGL ISFEEFKVMM A-GGGS-FAK IA
  PvCML10-2  EGVDQNGDGL ISFEEFKVMM A-GGGC-FAK IA
    BdCML15  EGVDRNGDGL VSFDEFKLMM AGGGG--FGK IGA
    OsCML15  DGVDRNGDGL INFEEFKLMM AAGAG--FGR IAS
    PhCML10  QGVDRNGDGL VSFDEFKLMM AAGGG--FGR MASSS
    PvCML15  QGVDRNGDGL VSFDEFKLMM AAGGG--FGR VSSSSFS
    SiCML10  QGVDKNGDGL VSFDEFKLMM AAGGS--FGR IAYS
  SbCML10-2  QGVDRNGDGL VSFDEFKLMM AAGGGG-FGR IGAA
    ZmCML10  QGVDRNGDGL VSFDEFKLMM AAGGGGGFGR IGA
  ZmCML10-2  QGVDRNGDGL VSFDEFKLMM AGG----FGR IGA
  PvCML10-3  QGVDRNGDGL VSFDEFKLMM AAGGG--FGR MASSSS
  AcCML25-2  RAVDSDGDGL IDYEDFKVMM MASKQ
  PaCML25-3  RVADSDGDGF VDFEEFKKMM TVSS
  PaCML25-4  RNFDRNGDGF ISLDDFIAML NSP
  PaCML25-2  RTFDSNGDGL IDFDEFTAMM NAS
  PaCML25-5  AGVDSDGDGY VSFDEFLIMM TP
  PaCML25-1  KGVDSNGDGQ VSFDEFMAMM TNTSHNSWTT GLN
  PaCML25-7  KGVDSNGDGQ VNFNEFMAMM TNTSNNSWIT GLH
  PaCML25-6  KGVDCNGNAQ VDFEEFKNMK SGST
  PaCML25-8  ACIDVNGDGV VTLEEFYAHV SNDAV
    AcCML27  SSVDADGDGY VNFEEFKKMM KNGLQ
    AcCML23  RSVDVDGDGC VNFEEFKNMM GTNTTKPKSS RY
  CcCML23-1  KKVDADGDGH VNFEEFKKMM TRS
  CsCML23-1  KKVDADGDGH VNFEEFKKMM TRS
    SlCML24  SKVDVDGDGC VNFEEFKKMM SRT
  StCML23-2  SKVDVDGDGC VNFEEFKKMM SRT
    SlCML23  SSVDDDGDGS VNFEEFKKMM TRA
  StCML23-3  SSVDVDGDGS VNFEEFKKMM TKA
```

```
   MgCML27-2  ESFDVDGDGS INFDEFKKMM TGGG
   GmCML27-1  SNVDGDGDGN VNFEEFKKMM TRS
   GmCML27-2  SNVDADGDGN VNFEEFKKMM TRS
  PvulCML27-  SNVDADGDGS VNFEEFKKMM ARS
     MtCML24  SNVDVDGDGN VNFEEFKKMM AH
   GmCML27-4  GNVDADGDGN VNFEEFKKMM SRS
  PvulCML27-  GNVDADGDGN VNFEEFKKMM TRS
   CsatCML23  SSVDVDGDGH VNFEEFKKMM TRS
     BrCML23  SKVDADGDGC VNFEEFKKMM MNGRATA
     CrCML23  SKVDSDGDGC VDFDEFKKMM SHGGA
     AtCML23  NKVDSDGDGC VDFEEFKKMM MINGSA
     BrCML24  SKVDTDGDGC VNFEEFKLMM NNGGA
     ThCML24  SKVDSDGDGC VNFEEFKKMM NNGGA
     CrCML24  SKVDTDGDGC VNFDEFKKMM SHGGA
     AtCML24  SKVDIDGDGC VNFDEFKKMM SNGGGA
     LuCLM24  RQVDQDGDGS VNFEEFRKMM STGNRAPAAC SCGHQLVERL ELHPKYLGDY SHWRVASCKL ARCSKSVSLF EDLARGRFPA RKSLFDRLRT TANCSTTGVA PCA------R ISPEKVVFVA PHDPPGSLSP VRLA
   ThCML23-2  SSVDVDGDGC VNFEEFKKMM SKMSRKQTCF KKFNQSCKPL NNSQNRNRLL PQTPALATAT VAVLPVKPLG VSLLCQRSRP IIITLSRAKQ MVRERKQALT RFW------V IAEIENVETE IVTIKSLIIL LKT
     MeCML23  RQVDQDGDGN VNFEEFKKMM TNGLL
   PtCML23-1  REVDQDGDGN VNFEEFKKMM TRGLA
     PtCML23  REVDEDGDGN VNFEEFKKMM TKGLA
     CpCML24  SQVDKDGDGH VNFEEFKKMM SNSP
   GrCML23-1  SQVDKDGDGS VDFEEFKKMM MNNS
   GrCML23-2  SQVDKDGDGN VNFDEFKKMM TNASN
     TcCML23  SQVDKDGDGN VNFEEFKKMM TNA
   EgCML24-1  GSVDRDGDGQ VDFDEFKAMM TKSSSSDS
     FvCML23  GSVDADGDGH VNFEEFKKMM KK
   MdCML27-5  SSVDVDGDGH VNFAEFKKMM TRK
   MdCML27-6  GSVDADGDGH VNFAEFKKMM TRN
  PperCML27-  RNVDADGDGH VNFDEFKKMM TPR
   FvCML27-1  TNFDVDGDGF INFQEFKKMM NRS
   BrCML27-2  GPVDADGDGN VNFEEFQKMM TSSSLANSNN GSSA
     AtCML27  GPVDADGDGN VNFEEFQKMM TSSSLLNSNG SAAPPST
   BrCML27-1  GPVDADGDGN VNFEEFQKMM TSSSLTNSNN GSTAAANSSS T
       ThC27  GPVDADGDGN VNFEEFQKMM TSSSLINSNG SANGASA
     BrCML26  GRVDTDGDGN VNFEEFKKMM SSPELLNKQS A
     CrCML26  GHVDADGDGN VNFEEFQKMM SSPELVKGSS AN
     AtCML26  GHVDTDGDGN VNFEEFQKMM SSPELVKGTV ANS
     ThCML26  GHVDSDGDGN VNFEEFQKMM ASPELLNKQG SSA
     CpCML27  GPVDSDGDGN VNFEEFQKMM TTSLAPAKE
     CcCML27  KPVDADGDGN VNFEEFRSMM TTSLKPGPAP
     CsCML27  KPVDADGDGN VNFEEFRSMM TTSLKPGPAP
   GrCML27-2  KSVDSDGDGN VNFEEFQKMM SAS-LAANGK GSKP
     TcCML27  TSVDSDGDGN VNFEEFEKMM SAS-APRIDN GSKP
   GrCML27-3  KSVDSDGDGH VNFEEFRKMM NASSIPSNGD GSKP
   CsatCML27  NSVDSDGDGN VNFEEFRKMM TDNSKSKAAQ QNGTAAAAP
   GmCML27-5  KSVDSDGDGN VNFPEFKRMM SNNRENASNA EEKTD
  PvulCML27-  KSVDSDGDGD VNFEEFKRMM CN-RENTC
     MtCML27  KSVDSDGDGN VNFEEFKKMM NNNQANSN
   GmCML27-3  KSVDADGDGC VNFEEFKTMM TTSKNRGGAT NGSVH
  PvulCML27-  KSVDADGDGF VNFEEFKTMM MTSKNTNGSV HQS
   LuCML27-1  KGVDSDGDGR VNYKEFEKMM ASSNGGGGPA KN
   LuCML27-2  QGVDSDGDGC VNFAEFEKMM AANSEATAPA VN
   LuCML27-3  QGVDSDGDGC VNFEEFEKMM AANGEATAPA VN
   PtCML27-2  KNVDSDGDGC VNFEEFQKMM AANINNGSAV VAP
   PtCML27-3  KNVDSDGDGS VNFEEFQKMM AANMGS
   MeCML27-1  SNVDADGDGS VNFQEFQKMM AATVNNGANA TDSGN
   MeCML27-2  GSVDSDGDGC VNFEEFQKMM ATTINNETAT ARAADGVQND A
     EgCML26  KSVDGDGDGN VNFEEFRKMM ASKGPGN
   FvCML27-2  QTVDADGDGN VNFEEFKKMM SNNSASNAGT VASNGA
  PperCML27-  KSVDADGDGN VNFEEFKKMM GNNASTVSNT NA
   MdCML27-1  KSVDTDGDGN VNFEEFKKMM GHSVSTN
   MdCML27-2  KSVDADGDGN VNFEEFKKMM GHSVSTNX
   MdCML27-3  KTVDADGDGN VNFEEFKKMM GHKASSDGTN AANPAA
   GrCML27-1  ASVDADHDGN INFEEFRKMM SDSKAGNEE
   MgCML27-1  RTVDSDGDGF VSFDEFRKMM TKTDGTANGG NAN
   SlCML27-2  NSVDSDGDGY VSFEEFKKMM TTSK
   SlCML27-1  TSVDANGDGY VCFEEFKKMM GNK
     EgCML23  SSVDRDGDGN VNFEEFKKMM TAASNGASS
   EgCML24-2  SLVDRDGDSN VNFKEFKKMM TAGC
     BdCML16  ASVDVDGDGC VGFEEFKKMM SRDAGAAHAD ADAG-----V PDKPKKE
     OsCML16  ASVDVDGDGC VGFEEFKKMM CRDAAAT--- ---G-----G ADKAKTE
     PhCML16  ASVDVDGDGC VGFEEFKKMM APQ--GSAA- -AAPGG--DV PDKAKKE
     PvCML16  ASVDGDGDGC VGFEEFKKMM APQPQGSAAD AAAPGG--GL PDKAKKE
     SiCML16  AGVDVDGDGC VGFEEFKKMM APKGGAEPAA VPPADA--AG PDKAKKE
     SbCML16  ASVDTDGDGC VGFEEFKKMM CPQQPQAGAG ADAGA---AG PDKAKKE
     ZmCML16  AGVDADGDGC VGFEEFKIMM RGPQPQPQPQ PQAGAGVTAV PDKAKKE
     PhCML18  RSVDADGDGS VNFDEFKKMM GAGAGARR
   PvCML18-2  RSVDADGDGS VNFDEFKKMM GAGTGARR
   PvCML18-1  RSVDADGDGS VNFDEFKKMM GAGAGARR
     SbCML18  RSVDADGDGS VNFDEFKKMM GAGAGGRR
     SiCML18  RSVDADGDGS VNFDEFKKMM GAGARR
     OsCML18  RSVDADGDGC VNFDEFKKMM GGGGGRR
     AcCML30  RAIDADGDDL IDMKEFMTMM TETMKMC
   AcCML30-2  RAVDVDGDGL IDMDEFMKMM TQTMKRF
     BrCML30  RAVDADGDGL VNMEEFMRMM TQSMNIV
     ThCML30  RAVDADGDGL VNMEEFMRMM TQTINIV
     CrCML30  RAVDADGDGL VNMEEFMRMM SSNIV
     AtCML30  RAVDADGDGL VNMEEFIKMM SSNNV
   LuCML30-1  RAVDNDGDGL VNMDEFTTMM TRTLVHN
   LuCML30-2  RAVDNDGDGL VNMDEFTTMM TRTLVHN
   CcCML30-2  RAVDTDGDGM VNMDEFMTMM TRSMKLG
   CsCML30-1  RAVDTDGDGM VNMDEFMTMM TRSMKLG
   MeCML30-1  RAVDADGDGM VNMDEFTTMM TQTLVNL
   PtCML30-1  NAVDIDGDGM VNMDEFMTMM TRSMTSG
   MdCML30-3  RAVDTDGDGM VDLDEFMTMM TRSMRPA
  PperCML30-  RAVDTDGDGV VDIDEFMTMM TRSMKTCLEN
   MdCML30-4  RAVDTDGDGM VDLNDFMTMM TSSMRRA
      VvCM30  RGVDTDGDGA VDIDEFTTMM TRSMVLMLRI PLIRAS---L LRIALTQTRV SSWRIILILM VRATRVLRLR VRWSLAIPLI RVLMLRITLT LIRVSPIGIT -LI------L IPIIHMLRVA LTLGLSQIPK FYHTTNIYSA KLG
   GmCML30-1  RAVDTDGDGM VDMDEFTTMM TQSLRHV
  PvulCML30-  RAVDTDGDGM VDMDEFTTMM THNLRHV
   MeCML30-3  RAVDGDGDGM INMDEFKTMM TQT
  CsatCML30-  RAVDSDGDGM VDINEFMTMM TRSAK
     MgCML30  RAVDANGDGV IDSDEFITMM TRTMTTY
   SlCML30-2  KAVDANGDGV IDLDEFVTMM TRTMTLC
   StCML30-1  KAVDANGDGV IDLDEFVTMM TRTMTLC
   EgCML30-1  RAVDTDGDGS VNMDEFTNMM TRTMKQC
   EgCML30-2  RAVDRDGDGV VDMDEFMTMM TRTLKSN
   CcCML30-1  RGVDADGDGL IDMDEFMTMM TRNVKVA
   CsCML30-2  RGVDADGDGL IDMDEFMTMM TRNVKVA
   GrCML30-1  RGVDADGDGL IDITDFTTMM TRTMKVCQR
     TcCML30  RGVDADGDGL IDINEFMTMM TRTMKPSQ
   GrCML30-2  KGVDVDGDGL IDINEFMIMM TRTMKPFH
   MeCML30-2  KGVDKDGDGL VDIDEFMNMM TCTMKSS
   PtCML30-2  RAVDGDGDGL IDMNEFMGMM TRTMKMC
   GmCML30-2  MGVDGNGDGF IDLNEFMRML MSGKKLA
  PvulCML30-  MGVDGNGDGF IDLNEFMRMM MSGKKLT
   MtCML30-2  KGVDSDGDGL IDLNEFTRMM MNGKKCA
   MtCML30-3  KGVDSDGDGL IDLNEFTRMM MNGKKCA
   MtCML30-1  KGVDGDGDGL IDLNEFTRMM MNGKKCA
  PvulCML30-  KGVDGNGDGF IDLNEFTRMM MSGKKLG
   MdCML30-5  KGVDSDGDGL IDMNEFTNMM GGFKTPTA
  PperCML30-  KGVDTNGDGL IDMNEFSKMM GNFKKPTA
  CsatCML30-  KGVDMDGDGF IDVQEFSKLM GKYSSKFQN
   SlCML30-1  RGVDANGDGL IDMDEFVTMM TRTMKLV
   StCML30-2  RSVDANGDGL IDMDEFVTMM TRTMKLV
     BdCML29  KEIDRNQDGF VDMDDFMDMM TGPRKKP
      PhCML6  REIDRNGDGF VDMDDFMAMM TRPRRKL
      SbCML1  REVDRNGDGF VDMDEFMVMM TRPRRKP
     BdCML20  AAVDADGDGV ISFDEFKAMM AS
     PhCML20  AAVDRNGDGV ISFDEFKAMM ATEPAA
     SbCML20  AAVDRDDDGV ISFDEFKAMM AIKPPPA
   ZmCML20-2  AAVDRDGDGV ISFDEFKAMM ATEPAA
   ZmCML20-1  AVVDIDGDGV VCFDEFKAMM DTAAAS
     SiCML20  AAFDRNGDGV ISFDEFKAML NTEPAA
     OsCML20  AAVDSDGDGV ISFDEFKAMM SKQA
   AcCML25-5  ASMDEDGDGA VSFEDFKLIV NSLS
     CcCML25  ASMDGDGDGA VSFEDLQLIV NSLL
   CsCML25-2  ASMDGDGDGA VSFEDLQLIV NSLL
     GmCML25  TSLDADGDGA VSLDEFRLIV DSLI
  PvulCML25-  ASLDADGDGA VSLDEFRLIV DSLL
   MtCML25-2  ASLDMDGDGA VSLDDFRLIV ESLI
   MdCML25-2  ASIDIDGDGA VSYDDFKLIV NSL
  PperCML25-  ASIDIDGDGA VSFEDFKLIV NSL
   TcCML25-2  ASMDADGDGA VSFEDFKLIV NSLL
   PtCML25-3  ASIDADGDGA VSCNDFKLIV NSLQILENSF LM
   CsatCML25  ASIDGDGDGA MFLHDFKLIL ALWLFILFSG RSNILL
   AcCML25-4  RGVDKDGDGL VDFEEFRSMM TGRLD
   CpCML25-2  KGVDRDGDGF VDFEEFRSMM RARK
   GrCML25-2  RGVDKDGDGF VDFKEFRLMM SAAERSS
   TcCML25-1  RGVDKDGDGF VDFKEFRSMM AAGTT
   PtCML27-1  KGVDKDGDGF VDFHEFRSMM TTSASWLED
   MtCML25-1  KGVDKNGDGF VDYEEFRSMM KSGQD
  PvulCML25-  KGVDKNGDGF VDFQEFRSMM QSGLAS
     RcCML25  KGVDKDGDGS VNFEEFRSMM TNTSRLANTM K
     FvCML25  KGVDRNGDGV VDFEEFRLMM TRNV
  PperCML25-  KGVDKNGDGA VDFEEFRLMM TRNYVS
   MdCML25-1  KGVDRKGDGA VDFEEFRLMM TKNYVS
   CcCML23-2  KGVDKDGDGF VDFEEFRSML SH
   CsCML23-2  KGVDKDGDGF VDFEEFRSML SH
```

```
     MgCML25-2  EGVDIDGDGF VSFDEFKFML CGGGLS
     StCML25-2  KGVDKDGDGF VNFEEFKQMM AAGCNF
    CsatCML36  EGVDHDGDGY VNFQDFRLMM TQKR
      PpCML23  SSVDRNGDQL VDFSEFKYLM QDARVY
    PpCML25-3  NNVDKDGDGY VDFEEFQELM VGS
    PpCML25-8  NSVDKDGDGH VDFQEFLELM GCS
    PpCML25-9  NSVDKDGDGH VDFQEFLELM GCS
    PpCML25-6  SNVDQDGDGF VDFKEFQSLL TAKS
    PpCML25-1  KCVDENGDHL ISFAEFQSLM SDQPEMRGAA LQSVLAAASP SPS
    PpCML25-2  SCVDIDGDGL VNFKEFEVLM TGHLAQ
   PpCML25-11  SCVDADGDQL VDFNEFQRLM SGHLAQ
   PpCML25-10  SCVDADGDQL VDFKEFRKLM NGHVTQ
    PpCML25-4  QSVDRDGDRL VSFAEFETLM SGICID
    PpCML25-5  QCVDEDGDHM VSYTEFEALM SGIRSS
    PpCML25-7  ECVDEDGDQM VNFREFEALM GGTSTCVY
   PpCML25-12  NSVDQDGDNM VNFAEFQCLM SSAFVC
      SmCML27  RGVDSNGDGR VDFEEFKLMM ASA
      SmCML26  GGVDSDGDGF VNFEEFQRMM LSSSSSSAAP STA
      BrCML36  AAVDDDGNGF VCFTEFSRMM DLQR
      ThCML36  AAVDDDGNGF VCFSEFSRMM DLER
    CrCML36-1  ADVDEDGDGF VCFTEFSRMM DLQR
      AtCML36  ADVDEDGDGF VCFTEFSRMM DLQR
    CrCML36-2  AAVDEDGDGF VSFNEFSRMM DLY
      BrCML35  ATVDRNGDGF VRFDDFRRMM ELQAPATNDH H
      CrCML35  ATVDRNGDGF VCFEDFCRMM ELQS--SDE
      AtCML35  ATVDGNGDGF VCFDDFCRMM VPAM--NDHH H
      CpCML35  AAVDKNDDGF VCFEDFSRMM EPQR
      MeCML36  AEVDKNGDGF VCFEDFSRMM ELQR
      FvCML36  AEVDKNGDGF VCFQDFARMM DLQR
    MdCML36-1  AGADKNGDGF VCFEEFAHMM ERQR
    PperCML36  AGVDKNGDGF VCFEDFARMM ELQR
    MdCML36-2  DGVDKDGDXF VCFEEFVHMM EPQR
    MdCML36-3  DGVDKDGDGF VCFEEFVHMM EPQR
    MdCML36-4  AEVDKNGDGF VCFEEFVHMM ELQR
      MtCML36  AGVDKNGDGF VCFQEFSLMM DLQR
      CcCML36  ALVDKNGDGF VCFEDFSRMM ELQR
      CsCML36  ALVDKNGDGF VCFEDFSRMM ELQR
    LuCML36-1  AEVDKNGDGF VCLDDFCRMM DLQR
    LuCML36-2  AEVDKNGDGF VCLEDFCRMM DLQSAF
    MgCML36-1  KGVDRNGDGF VCFEDFSLMM DQHCSTTKDK MI
    MgCML36-2  RGVDRNGDGF VCFEDFSLMM DHHQQR
      SlCML36  RGVDKNGDGF VCFEDFSLMM EQQR
    StCML36-2  RGVDKNGDGF VCFEDFSLMM EQQR
    StCML36-3  RGVDKNGDGF VCFEDFCVMM EQQQRC
      SlCML35  TSVDKNGDGF VCFDDFCLMM EQQR
    StCML36-1  TSVDKNGDGF VCFEDFCLMM EQQR
      VvCML36  AGVDKNGDGF VCFEDFSRMM EQQT
     OsCML1-1  GGVDTDGDGF VCFDEFARMM MCGRA
     OsCML1-2  GGVDTDGDGF VCFDEFARMM MYGCA
      AcCML38  QNFDLNGDGV LNFEEFRVMM A
      EgCML37  RAFDLNGDGV LSFDEFSVMM H
      TcCML37  RAFDLNGDGV LSFDEFSVMM R
    VvCML39-2  HMFDINGDGV LSFEEFSAMM R
    FvCML38-2  RMFDLNGDGV LNFDEFRIMM A
   PperCML38-  RAFDLNGDGA LSFNEFTIMM R
      RcCML39  RTFDINGDGL LSFHEFSLMM R
      CcCML37  RPFDINGDGV LSFEEFSLMM H
      BrCML37  RGFDQNGDGV LSFDEFLLMM R
      AtCML37  RGFDQNDDGV LSFDEFVLMM R
      ThCML39  RGFDQNGDGV LSYDEFVLMM R
      CrCML37  RGFDQNGDGV LSFDEFVLMM R
      CpCML37  RAFDLNGDGV LSFDEFALMM H
    SlCML38-1  RRFDLNGDGV LSFDEFKVMM TT
    StCML38-3  RRFDINGDGV LSFDEFKIMM TS
    SlCML39-2  RRFDTNGDGV LSFDEFKVMM TS
      StCML37  RRFDINGDGV LSFDEFKVMM TT
    StCML38-2  RRFDLNGDGV LSFDEFKVMM TT
    SlCML38-2  KRFDLDGDGV LSFDEFKVMM NGS
    StCML38-1  KKFDLDGDGV LSFDEFKVMM NSS
    BrCML39-1  KAFDLNDDGV LSFDEFALMM MR
    ThCML23-1  KAFDTNADGV LSFDEFVLMM R
    BrCML39-2  KAFDLNADGV LSFDEFALMM MH
      CrCML39  QAFDLNADGV LSFDEFALMM L
      AtCML39  QAFDLNADGV LSFDEFALMM R
    BrCML38-2  RAFDLNDDGV LSFDEFALMM R
      ThCML38  SAFDLNADGV LSFDEFVLMM R
    BrCML38-1  RAFDLNADGV LSFDEFALMM R
      AtCML38  SAFDLNADGV LSFDEFALMM R
      CrCML38  SAFDLNADGV LSFDEFALMM R
    AcCML41-2  QVYDIDDNGI LDFHEFHCMM T
    AcCML41-1  QVYDIDDNGI LDFHEFHRMM T
      AtCML41  KFYDIDGNGI LDFHEFRQMM TV
      EgCML41  RVYDTDGNGV LDFQEFNQMM A
      CcCML41  QVFDTDGNGV LDYHEFSQMM A
      CsCML41  QVFDTDGNGV LDYHEFSQMM A
    MdCML41-2  QVFDTDGNGV LDFNEFHRMM A
    MdCML41-1  QVFDTDGNGV LDFNEFHRMM A
    MtCML41-1  DAFDIDHNGV LDFNEFHQMM A
    PvulCML41  GAFDIDHNGV LDFDEFYQMM A
      PtCML41  QVFDIDGNGV LDFYEFNQMM A
      RcCML41  HVFDIDGNGV LDFHEFYQMM A
      SlCML41  KVYDIDGNGE LDYHEFRQMM TT
      StCML41  QVYDIDGNGE LDYHEFRQMM TT
      CpCML40  AQFDLNGDGV INFDEFRVMM L
    CcCML38-2  AYFDLNGDGV LNFDEFRIMM S
    CsCML38-2  AYFDLNGDGV LNFDEFRIMM S
    FvCML38-1  AKFDLNGDGV LNFDEFKVMM F
    MtCML41-2  SQFDIDGDGV LSFDEFRTMM L
   PvulCML38-  ARFDLNGDGV LTFDEFRVMM L
   PvulCML38-  ARFDLDGDGV LTFDEFKVMM L
    EgCML40-2  ARFDLNGDGV INFDEFRVMM S
    MgCML38-2  ARFDINGDGV LNFDEFKVMM SAC
    MgCML38-1  ARFDLNGDGV LNFDEFKVMM SST
    MgCML38-3  ARFDLNGDGV LNFDEFKEMM A
   CsatCML38-  AKFDLDSDGV LNFDEFKFMM S
    CsatCML41  AKFDIDGDGV LSFDEFRVMM S
    SlCML37-1  CRYDIDGDGL LNFHEFEIMM RC
    StCML38-4  CRYDIDGDGL LNFHEFEIMM RC
    SlCML37-2  GRYDINGDGL LNFEEFVIMM CC
    StCML23-1  ARYDINGDGL LNFEEFVIMM RC
    CcCML38-1  DRFDLNGDGV LSFEEFRIMM Q
    CsCML38-1  DRFDLNGDGV LSFEEFRIMM Q
    FvCML38-3  SRFDLNGDGL ISFEEFIVMM Q
    MdCML39-2  QRYDLNGDGL ISFEEFRIMM Q
    MdCML39-1  QRYDLNGDGL ISFEEFRIMM Q
      MdCML38  QRFDLNGDGV ICFEEFRIMM Q
   PperCML38-  NQFDLNGDGV ISFEEFRIMM Q
      TcCML38  KQFDLNGDGV LSFEEFRVMM Q
    MtCML38-1  KHFDLDGDGL LSFDEFITMM Q
    MtCML38-2  KHFDLDGDGL LSFDEFITMM Q
    MtCML38-3  KRFDLDGDGV LSFEEFRIMM E
      PtCML37  NKFDLNGDGV LSFEEFMVMM K
   PvulCML38-  ARFDLNGDGM LSFEEFRVMM N
   CsatCML38-  RRFDLNGDGL ISFEEFQIMM A
      EgCML40  RHFDLDGDGV ISFDEFKVMM M
    EgCML39-1  RHFDLDGDGV ISFDEFKVMM M
      EgCML38  RHFDLDGDGV ISFDEFKVMM M
    EgCML39-2  RHFDLDGDGV ISFDEFKVMM M
    EgCML40-1  CHVDLDGDGV ISFDEFKVMM M
    VvCML39-1  NQFDLNGDGV LSFDEFKVMM L
   PvulCML38-  HHFDLNGDGL LNFHEFTLMM NQ
   PvulCML38-  HHFDLNGDGL LNFHEFALMM NQ
    VvCML41-1  RQFDVNGDGV LGFDEFKLMM LMLLIPKIIF DCACQ
    VvCML41-2  GQFDVNGDGV LSFDEFKPHD ALILFDTFHM NFIFFLDL
      VvCML38  RQFDVNGDGV LSFDENLWGG WRERVKTER
    VvCML41-3  AQFDVNGDGV LSFDEFKLML L
      LuCML38  AEFDVNGDGV LDFDEFKVMM S
   CsatCML38-  AKFDLDGDGV LCFDEFRGMM LL
      AtCML40  SQFDINRDGI INFDEFRAMM Q
      AcCML39  DHFDLDGDGV LSFNEFIVMM Q
      BdCML31  CRFDLDGDGV LSFDEFKIMM S
      OsCML31  CRFDLNGDGV LSFDEFKIMM NA
      PhCML31  CRFDLDGDGV LSFDEFKIMM NA
      SbCML31  CRFDLDGDGV LSFDEFKIMM NAA
      PhCML19  CRFDLDGDGV LSFEEFKIMM DA
      SiCML19  CRFDLDGDGV LSFEEFKVMM DA
      OsCML19  CRFDLDGDGV ISFEEFKIMM DA
      SbCML19  CRFDLNGDGV LSFEEFKVMM DA
      ZmCML19  CRFDLDGDGV LSFEEFRVMM DA
      BdCML23  CRFDLNGDGV LTFDEFKTMM TME
      SiCML23  CRFDLDGDGV LSFEEFRTMM MG
      OsCML23  CRFDMNGDGV LTFDEFKTMM MA
      OsCML25  CRFDLNGDGV LSFDEFRVMM LMA
      OsCML26  CRFDLNGDGV LSFDEFRVMM LMA
    SlCML39-1  RRFDINGDGV LSFDEFKIMM TC
      AcCML21  EEMDWDKNGM VTFKEFLFAF TKWIGMDDNE -EEELGDENV
      MtCML21  EEMDWDKNGM VNFKEFLFAF TRWVGIDEFE DEENA
      VvCML21  EEMDWDKNGM VNFKEFLFAF TRWVGIDDIE DEEEEGEEKV
      CpCML21  EEMDWDKNGM VNFKEFLFAF TGWVGIEDEE EGEATSEEVI PVVPVYDTDK RSLECSN
```

```
TcCML21 EEMDWDKNGS VNFKEFLFAF TRWVGIDDME DEEEEEEGRV
CcCML21 EEMDWDKNGM VNFKEFLFAF TRWCGVGENE DEEEGEEKN
CsCML21 EEMDWDKNGM VNFKEFLFAF TRWCGVGENE DEEEGEEKN
FvCML21 EEMDWDRNGM VNFKEFLFAF TRWIGIDDLD DEDEEE
PperCML21 EEMDWDKNGM VNFKEFLFAF TRWIGIDDLE DEDED
MgCML21 EEMDWDRNGM VNFKEFLFAF THWVGIEDID DEDDDENV
SlCML21 EEMDWDKNGM VNFKEFLFAF THWVGIEDNE DEEGEE
StCML21 EEMDWDKNGM VNFKEFLFAF THWVGIEDNE DEEGEE
CrCML21 EEMDWDKNGM VNFKEFLFAF TQWVGIDENE EEEEEEEDNK EKA
AtCML21 EEMDWDKNGM VNFKEFLFAF TQWVGIDENE EEEEE--DNN EKA
ThCML21 EEMDWDKNGM VNFKEFLFAF TQWVGIDENE EDDDNN
CsatCML21 EEMDWDKNGM VNFKEFLFAF TRWVGIDENE DGEEEEEE
SiCML33 EEMDWDKNGT VTFKEFLFAF TRWVGIDNED DDEEDNE
SbCML33 EEMDWDKNGT VTFKEFLFAF TRWVGIDNED DDEDDEEAA
OsCML33 EEMDWDKNGT VTFKEFLFAF TRWVGIDDNE DDDDEEGV
SiCML29 EEMDWDKNGM VTFKEFLFAF TRWVGIDENE DDDE
SbCML29 EEMDWDKNGM VTFKEFLFAF TRWVGIDENE DDDE
OsCML29 EEMDWDKNGM VTFKEFLFAF TRWVGIDENE DDNE
PaCML21 EEMDFDKNGM ISFKEFLFAF THWVGVEVDD DDE
PpCML21 EEMDWDKDGM ITFKEFLFAF TDWVGLENDE EETGHNNVSL PF
AcCML22 KELDSNKCGQ VSFKEFLFAL INWVGFETDN EEELSI
CsCML22 EEMDWDRNGK VSFREFLFAL ISWIGIDNDE EIAEME
TcCML22 KEMDWDKNGK VSFREFLFAF INWVGIESDE ELPD
PperCML12 KEMDWSKRGK VSFREFLFAF IDWVGIDTNE EMSPVRSSSK
FvCML22 KEMDWDKNGK VTFRQFLFAF IDWVGIETDE EISQEQS
CsatCML22 KEMDWNNSGK VNFREFLFGF INWVGVDTDD DLSPT
MgCML22 KEMDWDKNGK VSFKEFLFSF ISWVGVDCDE DEGI
StCML22 REMDWNRSGK VSFRQFLFAF LDWVGIDTDD ENHTTKRETK CS---NNTST KLVDSAV
ThCML22 EEMDWGRKGK VGFREFLFAF IGWVGLDDAD DCI
AtCML22 EEMDWGRKGK VGFREFLFAF MSWVGLDDAD DDFMSS
BdCML32 KEMDLNKNGS VNLKEFLFSM IRWAGIETED EGS-NETSP
SbCML34 KEMDLNRNGK VNLKEFLYSM IRWAGLETED DGS-NEASP
OsCML34 KEMDLNRNGH VNLKEFLLSI IRWAGVETDD DEARNETSP
SmCML21-1 EEMDWDRDGR VTFKEFLLAF TGWVGIEDDE DQED
SmCML21-2 EEMDWDKNGM ITFKEFLFAF TDWVGIEEEE DADEDEEE
MpCML12 RELDWDKSGR VDFKQFLFAV EGWCGMDDDL DGSVEGGNNA GGGAS
BrCML32-1 KEVDQNNDGQ IDYGEFVEMM QKGN--TGVG RRTMRNSLNI SMRDA
PvCML36 SDADSDNDGR IDYSEFVAMM RKGT--AGAE PLNNKKRRDI VL
PvCML29 GEVDQDNDGR IDYNEFVAMM QKST--GGFG KKGHQYNLSI GFRDALKQAH S
ZmCML36-1 KEADQDNDGR IDYGEFVAMM TKGN--MGVG RRTMRNSLNI SMRDTPAGAL
MeCML32 QEVDTDKDGK ISYDEFVAMM KTGTDWRKAS RHYSRGRFNS LSMKLMKDGS LNLGNE
ZmCML36-3 LDVDIDRDGK ISYEEFELMM KAGVDWRNTS RQYSRAVYNT LSRKMFKDAS LKLGSNNGPL AAAAVKEQQD VD
ZmCML36-2 YQVDQNNDGQ TNYGEFVTMM QSDS--SGLG WQTMESSMNV PLREAPEVY
MpCML11 NEADTDGDGT VDYEEFVAMM TKMNEAKAAA
CreinCML20 GSGAVSWDEF RKWYTSSYAR YMEESQRRML -------DDE AEAPFGYDDA
CreinCML20 GSGYLDGPEF CGALRQAQAM ITMDEVRTML AYIHMDADRD ANGHISLDEI QAILRPFQ
AtCML48 DGTGKTVDLC FDSFLECGMI VKGLTEKFRE N----DPGYT GYATLSYDVF MLMVIPFIAT YD
AtCML49 KSGGRNRAIE YDNFIECCLT VKGLTEKFKE K----DTALS GSAIFNYENF MLTVLPFLVA
AtCML50 KSGGKNRAIE YDNFIECCLT VKGLTEKFKE K----DTAYS GSATFNYESF MLTVLPFLIA
CreinCML32 RHFDRDASGF VTFDELLLGL R---GQLNDR RLDMVKRCYK VLDKTGDGVV TLADLENRYD ASRHPDVVAG IKTPRQVLLE FLSVFEEQGG GVKGDGQVDF EE-------F KTYYAMISAN IDGGEHGDDY FELMMRNVWH VSGGEGWCAN TTCKRVLVVF EDDRQQVVEV TDDFDVDT--
RcCML23 TQFDEDSNRS ITEDEFVSGC HKIIGKAKQM VADDNDSSRK YLPQLHKMVQ PLIERKKAKL AEIEQQLSQI LNTAQNQQLA FLVTDGKPDV DKIRSLFAEF DKDDNKKMTA RELKGMIKSK FGSAKLDHDD VVKKMMKVFD VDKDKEIHVE EFTDGMKKRL SGDFQLIDEC IESLISESCI
SbCML6 RERDKDNDGK LNFQEFFSGL FYSIRHYDDE GITD-DTGGS DAPAKKSFSH LDLDNDGLLS ADELKPIIDN LHPSEHFYAK QQADYVISQA DTNKDGQLNM NEMIEN---P YVFYNALFTE DDYG------ FHDELR
SbCML15 SEMDHDGDGR INLEEFVA-- ---------- -----QSDLE RAEAEEKFRE LDADMDNYLT VEEARSVLQS LITGEFSYAT SHAKFLM-KA DVNQDGKLSL EEMLDD---Y VSFYSTVYTD DHYSNEVDSD SHDEL
VcCML5 RKMDVNDDQL ISLAEFSDMM ---------E ALLTDKSDED LDVWVAEVVF TASSRNVVGF GNTLGEVLQN LEFKEEQAKK IKTLFK--EL DRDKSGFVEL HE-------- ---LRLLLGK SGMDMEQLSE EVLAIMAKLD KNGDNKVSTQ EFYV-TLSAI RKSLRSDEEF MALLD-----
AcCML18 MSKEA VELDDEQL-- AELREIFRSF --DRNND---
MeCML18 MSGKDP VKLDDDQL-- AELREIFRSF --DRNKD---
RcCML18 MQ ESSKSLERDQ ETKMSRGKDP VKLDDEQL-- AELREIFRSF --DRNKD---
SlCML18 MSGDAE PKLDDDQI-- AELREIFRSF --DRNND---
StCML18-1 MSGDAE PKLDDDQI-- AELREIFRSF --DRNND---
CpCML18 MDDKEPA VKLDDEQI-- AELREIFRSF --DRNND---
MgCML18 MSNNKEPA VKLDDDQL-- AELREIFRSF --DRNND---
PtCML18-1 MSNKQPA VKLDDEQI-- SELREIFRSF --DRNND---
PtCML18-2 MSNKKPA VKLDDEQI-- AELREIFRSF --DRNND---
CcCML18 MSNKQP- MNLDDDQI-- AELREIFRSF --DRNND---
CsCML17 MSNKQP- MNLDDDQI-- AELREIFRSF --DRNND---
FvCML18-2 MSNKQP- VKLDDDQI-- ADLREIFRSF --DRNND---
PperCML18 MSNKQP- VKLDDEQI-- ADLREIFRSF --DRNND---
MdCML18 MSNKQP- VKLDDDQI-- ADLREIFRSF --DRNND---
TcCML18 MSDKQP- AKLNDEQI-- AELRDIFRSF --DRNND---
EgCML17 MSNKES- VKLDDEQI-- AELREIFRSF --DRNKD---
StCML18-2 MSNKAP- VKLDDEQL-- AELREIFRSF --DRNDD---
GrCML18-2 MSDKEEP TKLNDEQI-- GELREIFRSF --DRNND---
GmCML17 MSKKQT VKLDEEQI-- AELREIFRSF --DRNND---
PvulCML18 MSKKHV VKLDEEQI-- AELREIFRTF --DRNND---
MtCML18 MNKKQHQ VKLDDEQI-- SELREIFRSF --DRNND---
CsatCML18 LDDEQI-- AELREIFRSF --DRNND---
GrCML18-1 MKMGDKQP- TKLDEEQM-- AELREIFSSF --DRNND---
BdCML11 MSEAPAITV GEDPPPETPT ATDQQQQRRR EQAPPPQSSL EEEDADQL-- SELRQIFRSF --DRNKD---
PhCML11 MS VPFCVAQEDL LLGATSPPRS AFSGRSTQFV VLPVSLPASR SRHERHHHGP RRRRSRRPRR RRRRAKATGR PVARGHGGHP AGRQLRGHHH QPGGGAQQQQ SRLDDDQL-- EELREIFRSF --DRNAD---
SbCML11 MGWPAD FQGRREDPSS PAAPPNSSSS RPPPSSRSRD LNMSDATTAP TADAAAHSKQ APSSAPSS-L ETTMASIRRG GGSSVAATMS QQASQQQQQP SRLDDDQL-- AELREIFRSF --DRNAD---
ZmCML11 MSDATTAP ATGAGDHVVD AAAHKPPAAP SPDMASIRR- GGSSVTETVT Q---ATQQQQ SRLDDDQL-- AELREIFRSF --DRNAD---
OsCML11 MSEPATTTP TPTPAGDHDA AATACKPAET TTALITCR-- ---SSSCSAQ QQQQQQQQQE EPLGDDQL-- GELREIFRSF --DRNGD---
GmCML18 MSKKQM VKLDEEQI-- AELREIFRSF --DRNND---
BrCML18 MSRD DGDKPSPAPG KKLGDEQL-- AELREIFRSY --DQNND---
ThCML18 MSG DGDKPSPAPG K-LGDEQL-- AELREIFRSF --DQNKD---
CrCML18 MSG DGEKPAPA-- -KLGDEQL-- AELREIFRSF --DQNKD---
AtCML18 MSC DGGKPAPA-- -KLGDEQL-- AELREIFRSF --DQNKD---
LuCML18-1 MS SEKEPAP--- KKLDDEQL-- GELHEIFRSF --DRNKD---
BrCML17 MSHKVS KKLDEEQI-- NELREIFRSF --DRNKD---
CrCML17 MSHKVS KKLDEEQI-- NELREIFRSF --DRNKD---
AtCML17 MSHKVS KKLDEEQI-- NELREIFRSF --DRNKD---
ThCML17 MNHKIS KKLDEEQI-- NELREVFRSF --DRNKD---
SlCML17 M RGLDEEQI-- NELREIFRSF --DRNKD---
FvCML18-1 MSTKRKQSAA PKLDDEQI-- TELREIFRSF --DGDND---
LuCML18-2 MEGSQI-- ADLRQIFRSF --DRDSD---
EgCML18 SF --DRNND---
MdCML17-3 MSNKQP VKLDYEKI-- ADLREIFRPF --DRNNY---
MdCML17-2 MEEEQM-- AQLRKIFRSF --DGNKD---
PpCML17-1 MESN RLERELSSRK GGRASAVEVE EAGAMVVMAA AVEKEYVVGG VSLCKEQL-- AELREIFSRF --DRDQD---
PpCML17-2 MVVMTTGTVE RPEAEYVVGG VTLCKEQL-- AELKEIFARF --DRDQD---
AcCML16 MSPTR FFYSVSITNK NRAPHIYNQP TSHENQKTFS PLSLSFFFLL SSFFFLHFSS LFLLTIIIFM AALQTEQL-- QQLKDIFCRF --DMDSD---
FvCML15 SCFAWLKRKC RFRCLSGDHC PHPDVEVGST KVKGFGQRGN DVAKVKQGDK APPQEEIMKQ LDDAMLTPNE QEIGQPSNLT IIHHQNNNPH FTKANQTTTT LFSSSSSSSS SAATHHHRNQ QSHCVNHNLL FSYCAPSLFV SLLFIVLVLM PALQVEQL-- NQLRAIFARF --DMDSD---
BdCML12 MQSHRLAPE- DRVQEGSRSA RAHAPIQGQG QAVAPAAATR RAQRSTSMSL PPE------- --SGT----- ---PPPPPSG A-----GGGR SRLRDEQL-- RQLRELFLRF --DLDGD---
PhCML12 MQSFRPAPE- DRAREGRAAA QQH---DAMS A------AGP RPKRSASMPF HGA------- --GAG----- -KGRDPSPAP A-----AGGR ARLRDEQL-- RQLRELFLRF --DLDRD---
PvCML12 MQSFR----- -RAREGSAAA QQR---DAMP A------AGP RPKRSASMPF HGA------- --GAA----- -KGRDPSPAP A-----AGGR ARLRDEQL-- RQLRQLFLRF --DLDRD---
SiCML12 MQSSRPATE- DRVREGSATA AAS---PAQP ATMSTTAAAA RTKRSASMPH PPA------- --GGG----- -KARDPSSAP A-----GGGR ARLRDEQL-- RQLRELFLRF --DLDRD---
SbCML12 MQAQCPRPE- DRVPGGSATA AAP---PVQP ATATMSTTNA RTKRSASMPL HGT------- --GASASAGA GKGRDPSPTP G-----PGGR ARLRGEQL-- RQLQELFLRF --DLDGD---
ZmCML14-1 MQSPRPEE DRVRDGSATA P-P---PVLP ATATTSTTTA RTKRSASMPL SGA------- --SAGTGRDR DPSPAPAPAP A-----PGGR SRLRDEQL-- SQLRELFIRF --DLDGD---
OsCML12 MQSQRERPRE DRVHEETRGA DHAHPSVPHA AAAASATATE TATRTMSLHA GGVVVVDGKE KGKKEEGEGK RKGKAPATAE A-----VRGR ARLRGEQL-- RQLHEIFLRF --DLDGD---
BdCML14 M TTTTKTTLTP ----APAANK GKG------- APLRGSQL-- KQLRELFTRF --DMDGD---
PhCML14 M TRSAPAAAA- --S-VPAPTK KHQ------H APLRGSQL-- KQLREIFRRF --DMDGD---
PvCML14-1 M TRSAPSAAAA A-S-VPAPTK KQQ------H AALRGSQL-- KQLREIFRRF --DMDGD---
PvCML14-2 M TRSAPAAAAA AAS-VPAPTK KQQ------H AALRGSQL-- KQLREIFMRF --DMDGD---
SiCML14 M TRSAPAAAAA --S-VPATKQ QQQPPH---H AVLRGSQL-- KQLREIFRRF --DMDGD---
SbCML14 M ARSAPAAAAA ASSPAPKPQP QPQPHP---H PQLRGSQL-- KQLREIFQRF --DMDGD---
ZmCML14-2 M TRSAPPASP- -----PAPKP ---------- -VLRGSQL-- EQLREIFRRF --DMDGD---
OsCML14 M TTMAARRSEA ----APAPQQ ---------- --LRGSQL-- KQLRELFRRF --DMNGD---
BrCML16-1 MA SSKPTDQI-- KQLKDIFARF --DMDGD---
BrCML16-2 MA SAKPTDQI-- KQLKDIFTRF --DMDGD---
ThCML16 MA SAKPTDQL-- KQLKDIFARF --DMDGD---
AtCML16 MA STKPTDQI-- KQLKDIFARF --DMDKD---
CrCML16 MA SAKPTDQI-- KQLKDIFTRF --DMDND---
CpCML16 M AAIQSDQL-- AQLKHIFTRF --DMDSD---
MeCML16-1 MM ATIQSDQV-- KQLKDIFMRF --DMDSD---
PtCML16 M ATLQTDQL-- KQLKDIFIRF --DMDSD---
RcCML16 MM ATLQPDQL-- KQLKDIFMRF --DMDSD---
MgCML26 MM AKMQSEQI-- KQLKDIFKRF --DMDHD---
PperCML16 MM AALGSDQL-- KQLKDIFMRF --DMDSD---
VvCML16-2 MVM DTLKSDQL-- TQLKDIFKRF --DMDSD---
CcCML16 MTVM ETVQSEQL-- KQLKDIFMRF --DMDSD---
CsCML16 MTVM ETVQSEQL-- KQLKDIFMRF --DMDSD---
LuCML15-2 MTTAAA AAVRADQI-- QQLRDIFMRF --DMDSD---
MeCML16-2 M LTLESNQL-- QQLKEIFMHF --DMDGD---
SlCML16 MV SIQSDEQL-- KQLKEIFGRF --DLDHD---
GmCML16 M SMLETDQI-- KQLNDIFKRF --DMDQD---
PvulCML16 M SMQQTDQI-- KQLNDIFNRF --DMDSD---
EgCML15-2 MAAAS AALQSEQL-- KQLRDIFARF --DMDGD---
CpCML15 MG EFEEQDQL-- SQLRDIFDRF --DMDSD---
CcCML15 M GGLHIDQL-- NQLRDIFARF --DMDSD---
CsCML15 M GGLHIDQL-- NQLRDIFARF --DMDSD---
CsatCML15 M AALAVEQL-- NQLRDIFARF --DMDSD---
MdCML15-2 M PALRVDQL-- NQLRDIFARF --DMDSD---
PperCML15 M PALAVDQL-- NQLREIFARF --DMDSD---
MdCML15-1 M PALRVDQI-- NQLRDIFARF --DMDSD---
GrCML15-2 M VALRVEQL-- KQLKDIFARF --DMDSD---
GrCML15-3 M VAMHVEQL-- NQLKDIFTRF --DMDSD---
TcCML15 M VSLRVDQI-- NQLKDIFARF --DMDAD---
MeCML15 MPT AASCVEEL-- KQLKDIFTRF --DMDSD---
RcCML15 MAAT AAMRIEQY-- NQLKDIFARF --DMDSD---
PtCML15 MTE AALHVEQL-- NQLKEIFGRF --DMDSD---
VvCML16-1 M AELEVNQI-- KQLRSIFSRF --DMDSD---
SlCML15 M TTKKRGKQIM SRLHVDQL-- DQLRNIFSRF --DMDDD---
StCML15 M SRLHVDQL-- DQLRNIFSRF --DMDND---
BrCML15 MEGQL-- RQLQDIFNRF --DMDGD---
CrCML15 MEGQI-- RQLKDIFDRF --DMDAD---
AtCML15 MEDQI-- RQLKDIFDRF --DMDAD---
ThCML15 MEEQL-- RQLKDIFERF --DMDGD---
CsatCML16 L-- KQLHDIFRRF --DMNSD---
GrCML16 MAMMSSCGNI SVQSADQL-- KQLKDIFMRF --DMDSD---
```

```
EgCML15-1      MAASL-- SQLRDIFARF --DMDAD---
GmCML15-2      M SKLQVNKL-- NQLREICATF --YMDSD---
GmCML15-3      M SKLQVNQL-- NQLREIFAKF --DMDSD---
PvulCML15      MYCTSLLHC CFVDYLENNM SKLKVNQL-- RQLRDIFEKF --DTDSD---
GmCML15-1      MS SKLQVQQL-- NQLREIFGRF --DMDSD---
MtCML15        M SKVKVDQL-- NQLREIFRRF --DMDSD---
GrCML15-1      M EPLGEDDL-- SQLKEIFARF --DMDSD---
FvCML16        M AKLETDQL-- KQLKDIFMRF --DMDSD---
LuCML15        MTTLRM-- SQLCDIFDRF --DMDSD---
AcCML20        MASLYRGSS RIDKPR-GRP HGLTQQKR-- QEIKEAFELF --DTDGS---
GrCML20-2      MASLYRGAS RKDKQK-GRH HGLSQQKR-- QEIKEAFELF --DTDGS---
FvCML20        MAGRKK KKEKSEAGVR EKDRFRATAK GGGVEGR-EG GRLVEGLSRV VFEGGSEGAV VEEGEDLERK IDGGDDVTMV GGGDDGGRRG IQVGEIEVED EKATLYRGPS RKEKPRAGRH HGLNQQKR-- QEIKEAFELF --DTDGS---
MdCML17-1      AFSELLG EERSNSSRGH FLRPSYLTRR SMASLYRGPS RKEKPR-GRH HGLTQQKR-- QEIKEAFELF --DTDGS---
MdCML20        MASLYRGAS RXEKPR-GRH HGLTQQKR-- QEIKEAFELF --DTDGS---
PperCML20      MASLYRGVS RKEKPR-GRH HGLPTQKK-- QEIKEAFELF --DTDGS---
CcCML20        MASLYKGVS RKDKIR-GRH HGLSQQKK-- QEIKEAFELF --DTDGS---
CsCML20        MASLYKGVS RKDKIR-GRH HGLSQQKK-- QEIKEAFELF --DTDGS---
CpCML20        MASLYRGQS RKEKPR-GRH HGLTQQKR-- QEIKEAFDLF --DTDQS---
PtCML20        MASFYKGQN RKDKPR-GRH HGLTQQKR-- QEIKEAFDLF --DTDGS---
MeCML20-1      MESLHRAQS RKDKPR-GRP HGLSQQKR-- QEIREAFELF --DTDGS---
CrCML20        MSSLYRGVS RKEKPR--RH HGLTTQKR-- QEIKEAFELF --DTDGS---
AtCML20        MSSIYRTVS RKEKPR--RH HGLTTQKK-- QEIKEAFELF --DTDGS---
ThCML20        MSSLYRGVS RKEKPR--RH HGLNQQKR-- QEIKEAFELF --DTDGS---
CsatCML20      MASLYRGVS RKEKPK--GR HGLTPQKK-- QEIKEAFELF --DTDGS---
SlCML20        MTI AEGSLYRGQS RREKPR-GRN QGLTQQKR-- QEIREAFELF --DTDNS---
GrCML20-1      MKSSIYKDTP GKDKHK-GRH HGLSQQKK-- QEIKEAFELF --DTDGS---
EgCML20        MGLQRGPS RKDKNR-GRH HNLTQQKR-- QEIREAFELF --DTDGS---
StCML20        MASVSRKIS RKETRN--RH PGLTQQKR-- QEIKEAFELF --DTDGS---
GmCML20        MSSLYRGES RRYNNKPRGR HNLTTQKK-- QEIKEAFELF --DTDGS---
PvulCML20      MSSLYRGES RRYNSKPKGR HHLTTQKK-- QEIREAFELF --DTDGS---
MtCML20        MSSSILRGDS RRFNNKQRGK HHLTQQKR-- QEIKEAFELF --DTDGS---
VvCML20-1      MN RQKSMYGEKS RKDKPK--GR HGLPQRKR-- QEIKEAFDLF --DTDGS---
VvCML20-2      MYGGAS RREKPR--GR HGLTTQKK-- QEIKEAFDLF --DIDES---
BdCML8         MSTVKGQT RRERPR-ARA HGLTQQKR-- QEIKEAFDLF --DTDNS---
SbCML8         MS FNQSTVKGQT RRERPR-ARP HGLTQQKR-- QEIKEAFDLF --DTDNS---
ZmCML8-2       MS FNQSTVKGQA RRERPR-ARP HGLTQQRR-- QEIKEAFDLF --DTDNS---
PhCML8-1       MSYAGSA PGERPR-TRP HGLTQQKR-- QEIKEAFDLF --DTDNS---
PvCML8-2       MAYTGSA PGERPR-TRP HGLTQQKR-- QEIKEAFDLF --DTDNS---
ThCML19        MSEAVQLR RGLKPR-GKT YGLTNQNR-- REIREIFDLF --DIDGS---
AtCML19        MA NYMSEAAQLR RGLKPK-GKT YGLTNQKR-- REIREIFDLF --DIDGS---
MeCML20-2      P ICKGFFSISV EYFSLNFRNL IASAG----N PMASLYSGKS RKEKPR-GRH QALSQQKR-- QEIKEAFELF --DTDGS---
MpCML20-2      MAVGSA RRDKGK-GRS TGLTEEQK-- QEIREAFDLF --DTDGS---
SmCML20        M SYR-KGIAAA KRDKNK-GRT HGLTEEQK-- QEIREAFDLF --DTDGS---
OlCML20        MWAG PARKGK-GRS SGLSEEQK-- QEIREAFDLF --DTDGS---
VcCML20-1      M SYR-KNVAAA RRDKKG-GR- TGLTEEQK-- AEIREAFDLF --DTDGS---
VcCML20-2      M SYKAKTVVSA RRDQKK-GRG AGLTEEQK-- QEIREAFDLF --DTDGS---
PhCML8-2       MASAYKG YE--PPSNSS G--------- -YRKDK-VRR KKLTAQKR-- KEIKEAFDLF --DIDGS---
PvCML8-1       MASPYKA YE--PPSNSS G--------- -YRTDK-VRR KKLTAQKR-- KEIKEAFDLF --DIDGS---
PvCML8-3       MESPYKA YE--PPSNSS S--------- -YRKDK-VRR KKLTAQKR-- KEIKEAFDLF --DIDGS---
ZmCML8-1       MTGTYEA SDRPPPPNSA G--------- -YRTDK-VRR KRLTAQKR-- KEIKEAFDLF --DIDGS---
OsCML8         MASKYRG YYHDEASSAA GGGGGGGGGD GYRREKQVRK KRLTAQKR-- KEIKEAFDLF --DTDGS---
SmCML19        M QGITDEQR-- SELREAFDMF --DTDSS---
MpCML20-1      MLK KQQQAVGGFV HAAPKKKKVL KELTPEED-- AELREAFNLF --DADGS---
AcCML11        MA DVLTEEQI-- IEFEEAFHLF --DKDGD---
GmCML11-3      MA DILSEEQI-- VDFKEAFGLF --DKDGD---
GmCML11-4      MA DILSEEQI-- VDFKEAFGLF --DKDGD---
MeCML11-2      MA EILTEEQI-- VEFKEAFCLF --DKDGD---
MeCML11-1      MA EILTEEQI-- VEFKEAFCLF --DKDGD---
PperCML11-     MA DVLSEEQI-- VEFKEAFCLF --DKDGD---
GrCML11-3      MG DILTQEQI-- VEFKEAFNFF --DKDGD---
BrCML11-2      MGETKQQ LELTQEQI-- MEFKEAFCLF --DKDGD---
BrCML11-3      MDKTQLQQ QELTQEQI-- MEFKEAFCLF --DKDGD---
CrCML11        MEETQQQQK QELTKEQI-- MEFKEAFCLF --DKDGD---
AtCML11        MEEIQ QQQQQQQQQQ QQQQQQQQQQ QELTQEQI-- MEFKEAFCLF --DKDGD---
ThCML11        MDETQ QELTQEQI-- MEFKEAFCLF --DKDGD---
BrCML11-1      MDQTQQ QELTQEQI-- MELNEAFCLF --DKDGD---
FvCML11        MG EVLSEEQI-- AEFQEAFCLF --DKDGD---
PperCML11-     MG EVLTEQQI-- AEFQEAFCLL --DMDGD---
GrCML11-2      MG EIMSEELI-- AEFQEAFCLF --DKDGD---
GmCML11-1      MK DALREEQI-- GEFLEAFCLF --DKDGD---
GmCML11-2      MK EALREEQI-- GEFLEAFCLF --DKDGD---
PvulCML11      MK ETLREEQI-- GEFLEAFCLF --DKDGD---
PtCML11-1      MV DVFTEKQI-- AEFQEAFCLS --DKDGD---
BdCML4         M EGLTGEQM-- VAFKEAFSLF --DKNGD---
OsCML4         M EGLTSEQM-- VAFQEAFLLF --DKNGD---
PhCML4         MWASQSL LSGCNSCICM EGLTGEQR-- LAFQEAFSLF --DKNGD---
PvCML4         M EGLTGEQR-- LAFQEAFSLF --DKNGD---
ZmCML4         M DGLTDEQR-- LAFQEAFSLF --DKNGD---
PhCML5         M DLLSPEQV-- SEFREAFAFF --DKDGD---
PvCML5-2       M DLLSPEQI-- SEFREAFAFF --DKDGD---
PvCML5-1       M DALSPEQI-- SEFREAFAFF --DKDGD---
SiCML5         MAASL PCHFSDDAPS LCTSAEASSS FSKPALFCSA HCRCVDRALV LVETRTSRSM DLLNPEQI-- SEFREAFAFF --DKDGD---
ZmCML5         M DLLNPEQI-- SEFREAFAFF --DKDGD---
SbCML5         M DLLNAEQI-- SEFREAFAFF --DKDGD---
OsCML5         MAEVE VRVRQEQV-- AEFRETFAFF --DKDGD---
EgCML11        MG GELTKEQI-- LEFKEAFCLS --EKDGD---
BdCML1         MA DQLSDEQI-- GEFKEAFSLF --DKDGD---
BdCML3         M DDLSKEQI-- AEFREAFSLF --DKDGD---
OsCML2         M DHLTKEQI-- AEFREAFNLF --DKDGD---
OsCML3         M DHLTKEQI-- AEFREAFNLF --DKDGD---
CsatCML50      MTTFL --FHFFI---
SiCML2         MDEVE QQLSKQQI-- EEFREAFSLF --DKDGD---
ZmCML2         MEEVE QQLTKKQI-- EEFREAFSLF --DKDGD---
GrCML11-1      MA DTLTEDQI-- SEFREAFCLI --DKDSD---
PtCML11-2      MA DALAGEQVNV AELREIFSLI SIDKVAD---
PtCML11-3      MA DSLTEEQV-- AEFREAFCLI --DKDAD---
PperCML11-     MA EALTEAQI-- SEFQEAFCLI --DKDSD---
BdCML5         MASA AAMSAEQV-- REFRSAFAFF --DKDGD---
OsCML6         ME SHLNEQQI-- SDFRDAFSLF --DKNND---
MdCML11        MA DVLSEEQI-- VEFKEAFCXF DKDGDGEFPT
AtCML13        MGK DGLSDDQV-- SSMKEAFMLF --DTDGD---
AtCML14        MSK DGLSNDQV-- SSMKEAFMLF --DTDGD---
BrCML34-2      MSAKQIFEKF --DKNKN---
BrCML34-1      MSAKQILQKF --DKNND---
CrCML34        MSAKRVFDKF --DKNKD---
AtCML34        MSAKRVFEKF --DKNKD---
ThCML34        MSARQIFEKF --DKNKD---
BrCML32-2      MSYVKVFESI --DKNKD---
CrCML32        MSVAQIFERI --DKNRD---
CrCML32-2      MSVAQIFERV --DKNRD---
CrCML32-1      MSVAQIFERV --DKNKD---
AtCML32        MSVAEIFERV --DKNKD---
AtCML31        MAEIFESV --DKNKD---
ThCML32        M SSVAEMFERI --DKNKD---
ThCML33        MKS TKKHMNVNMN LSTSDIFEKF --DKNKD---
AtCML33        MNN MSLSDIFERF --DTSKD---
EgCML25-2      SEVS AQLRQVFELI --DADGD---
SmCML25        KELEQVFRSI --DTDGD---
LuCML25-1      MSSLGQKASD EELQKMITEF --DADGD---
AcCML25-3
BdCML2         RSLGQRPTEA ELRDMIAEVD ADGNGAVDFA EFLALVDRKL L--------- ---------- ---DAEDDLR DAFRVFDADG NGFISLDELR RVMLDLGERL SDEELAQMLL E-ADGDGDGQ INYSEFAKLM MSKLELAGSK PMVPPLFKNG IRVCRYL--- LAFR-ILQVG --RNVVR---
CrCML12        MP QVRAQIDSHT LHLSIFLFSH HRKKKTMGAQ TTYDQVTELC EAFSAFDVDG DGCITAKELG TVMRSLGQNP TKAELQDLVN E-MDADGNGT IDFLEFLCAM IDT---DSKE KFIKAISVVS QLTDEQL--- SEFKETFSAF --DKDGD---
AtCML12        RSIGEKPTKA DLQDLMNEAD LDGDGTIDFP EFLCVMAKNQ GHDQ----AP RHTKKTMADK LTDDQITEYR ESFRLFDKNG DGSITKKELR TVMFSLGKNR TKADLQDMMN E-VDLDGDGT IDFPEFLYLM AKNQGHDQAP RHTKKTMVDY QLTDDQI--- LEFREAFRVF --DKNGD---
CsubCML24      TFLAIPLEDV EI--IFSMFD DDDSGAISLQ EFRRVTTALR LRLR----RV SHLQRTGLQT DTGDETQGLL VTF--FGKKG QDQLDLKHFA AFCTELHAEL VRLEF----L H-YDIKGQGY ISGTSFAHSL VSHCNMRNVD HFLSRI---- DNLPDKI--- KALKVTWAEF --QQFAQ---
PpCML7         SLEHNSHTLA DPEAVARNTA FSVSEVEALY ELFKNISSVG VYDGVV--NK EEFQLALFKQ SNKGESLFSD RVFNLFDEKQ KGYLEFSEFA RALSVFHPNA NVEDKIDFAF RLYDLQHQGF IERAEVKRMV VATLAESG-- -----LNLTD DVIEDII--- NKTFAEADTK --MDGKI---
MpCML21-1      MTVKQ FQKKLVGWMS ARGSGLVKLR AMFKYLDTDG NGKVSHAEFA KGLHRMNIHP RHEDMVKILK H-YDANEDGE IDFEGFFKTL IPEQDNAGFV DGNWQWDAYH DPKPANI--- VPTSHMLYGN --GLEKM---
MpCML4         MLDR VRTEIVEKGG IHG--VHTLR RIFRIMDDGG NNRIEPAEFK FALKDMGISI TKDELEKAMT L-FDKNKDGG LDVNEFTNAI RGKM-NPRRI DLVRAAFQVL DREGRGV--- VTMDDLV--Q --IFDPS---
MpCML30        MQG GRLVPFERSQ EDRFIQGLDG YLKTQQKRLK DVFDTFDRDK QGSLNRAEVT NILRAILPDA SAGDVQYFRM L-LDLNGDGE VSFAEFCDGM -----SA--- ----VLKTIK QKLAIGG--- VNLSVAFGKH --DKGRT---
MpCML15        YLKTEKRRLR DVFDAFDLDK DGALDRRELS RAIAKILPSA SNHDMHYFRV M-LDVDGDGV LTFDELLGTI RDCRAAG--- ----ALRRLS DFMRHNA--- ADVRAVFDAC --DLDGS---
MtCML46        MALPQIS QSNSISNSTS PLFGLIDLFL --YLTFFNKI HNFFSSIWFF LLCQIHSGSS EVREEKKVSE SKCSSQENES NIGRDNGDM- --IERDEVKM VMEKMGFFCS SE-SEELEEK YGSKELCEVF EENEPSL--- EELKQAFDVF --DENKD---
AtCML45        MESKSSSSSL PLFALFNFFL ISFCRWVSST RIFLSRFVPL LQHHQRVFDK KNNKDQQETL TKQEDDDDDD DDDDDDDDDD IDISREEAEM VMRSLGLFYN DD-Q--LQEQ YSAKEVSSLF EEKEASL--- EEVKQAFDVF --DENKD---
AtCML46        MTENQLY SFITMKSSLS KCKQSSSLSF PLFGLINFFL IGFFRWVSFA QLFFSRFWPL VQHQQCVSEK KSKDLEFQTS IKHEEYRDDD DDG------- --LCREDVGM VMKSLGLSTD QE-NEGLQKQ YSSKEVSNLF EEKEPSL--- EEVKQAFDVF --DENRD---
AtCML47        M EDSSLLSPIS LLTIVIFLFI LNLMMIIQDF SSSFPFRFHL FFSNAYILFT SIRNNKQNTE LPIIKKVVVP N--------- ----RADIKT SVEEVKAIID DS-EALYECL IEEGEEYLLE KNEMMGK--- EIVKEAFRLF --DENQD---
VcCML4         MALQRL- RGMGVGRTSA SAHRRAVSCN VINHRTLQHQ TQNSQEPRSS VASQLLAAGL ALGISLAGSP VVAAPQYGSL AD-------- --IVRSSFEF VDENKDGVVT KE-----ELL RTSKAVAEDV EFMLPDE--- SQLEFAMKLF --DLNQD---
OsCML1-3       MKLSIQSFA RKLSLPSPKR TWSSGGGSSK RDGGMSKNGS GVKRAISRSE ASSFASASSE SESSSDDALM ARSTPRSVLP AE-------- --ISRRELEA VLRRLGHGEP DD-----EEL DAVAAIAAEA EAGGGED--- ELME-AFKVF --DADGD---
AtCML44        MDCSFI TTNDLRRMFK TLDKNQDGLV TLDELLWILD KLGWAE---- --HTPDELEL IVGKQSLDLD EFLRFYYDAV LDSKGSKKNI DVVADND--- EAIARAFNVF --DVNGD---
OlCML11        LKAIYKA FLESDKDGSG RVDVNEFVR- ---------- --MLRVDRTP FVERLFSMFD TDRTGLIDVK EFIVGMANVG GEARDNK--- --IQFAFSVY --DLDGS---
AtCML1         MPI FQWLKRCLCG DTNITIDQAI ALVDEEIAN- ---------- --LRKLERGY QTNIRNAENA RDQTNVQIER DSFNDTIRLL -QAECNL--- --INLEIPTL --NEEKF---
CsubCML20      MGDA QLRQWFNAID VDRSGQLDAE ELQR--ALAL GGLHFSLNLA NKMIRIHDRD NSGAVSVEEF KTLHQFLLDT QNRFQQADTR RHGRIDKQTV ERALQAQG-- --YKLDGPAF HTLFNAYNPE RNGTMDLTEF IAMTLFLQSA NATFSAF--- DQQRMGRVTL --DYNQF---
SiCML36        MESAV VLREWFDRVD AARTGNITAP QLQS--ALAV GNLDFPLSVV QQMIRMYDFD RNGTMSFEEF LALNKFLQKV QSVFSTLE-R GRGFLSLEEV YEALIKLG-- --FSLDSPAF YTVCESFDKS KKGMVHLDEF ISLCIFVQSA RNMFNSF--- DTSKQGRVSL --DFNQF---
SbCML50        MENAV VLREWFDRVD AGRTGNITAA QLQG--ALAV GNLNFPISVV QQMIRMYDFD RNGTMSFEEF LALNKFLQKV QSVFSTLE-R GRGFLSLEEV YEALIKLG-- --FSLDSPAF YTVCESFDKS KKGMIQLDEF ISLCIFVQSA RNLFNSF--- DTSKQGRVTL --DFNQF---
MtCML11        RNLRPQPTAT DFRPAFDILD TDCDGKISRD DLRSFYTTVT GGVNGGDDAI RAMMSVADTN KDGFVEYEEF ERVVSG---- ----NNGEKR PLGCGAMEDV FKVMDRDG-- -----DGKLS HGDLKNYMAW AGFAASDEEI NAMIKLGGGD QNGGVSF--- DGLIR---LL --ALDHF---
Consensus      ...d.dgdg. ..f.ef..mm .......... ......   ...... .......... ........ .......... .....

               541                                                                                                                                                                                    720
AcCML3-1
CcCML7
CsCML7
GrCML5-1
GrCML5-3
TcCML5-1
MeCML3-1
MeCML5
PtCML3-3
PtCML3-4
RcCML3
LuCML5
LuCML3-3
LuCML3-2
```

GmCML5-1
GmCML5-2
PvulCML3-1
MtCML7
CpCML3-1
CsatCML5
SlCML5
StCML5
FvCML5
MdCML3-1
MdCML3-4
PperCML5-2
EgCML5-1
EgCML5-4
MgCML4
LuCML30-3
StCML3-1
BdCML30
OsCML30
PhCML30
SbCML30
ZmCML30
SiCML30
AcCML3-2
CpCML5
MgCML3-2
PtCML3-1
PtCML3-2
PtCML5
PperCML5-1
StCML3-3
GrCML3-2
TcCML5-2
CcCML3
CsCML3-1
EgCML5-3
GmCML5-3
PvulCML3-2
MtCML5
FvCML7
BrCML4-1
CrCML4
BrCML4-2
ThCML4
AtCML4
CrCML5-1
AtCML5
ThCML5-1
BrCML5-1
BrCML5-2
BrCML3-1
BrCML3-2
CrCML3
ThCML3
AtCML3
CpCML3-2
PtCML3-5
EgCML3
GrCML3-1
CsatCML3
LuCML3-1
FvCML3
MdCML3-2
CsCML3-2
SlCML3-1
StCML3-2
MeCML3-2
SlCML7
StCML7
SlCML3-2
GmCML3-1
GmCML3-2
GmCML3-3
PvulCML3-3
BrCML5-3
CrCML5-2
AtCML6
ThCML5-2
CrCML7
AtCML7
GrCML5-2
GrCML7
BrCML2-1
BrCML2-2
CrCML2
ThCML2
AtCML2
GrCML3-3
GmCML3-4
GmCML3-5
MtCML3
PvulCML3-4
MeCML3-3
BdCML28
PhCML28
SiCML28
PvCML28-1
PvCML28-2
SbCML28
ZmCML28
OsCML28
MgCML3
MgCML7
BdCML22
PhCML17
SiCML22
SbCML22
OsCML22
ZmCML17
OsCML17
SmCML5
SmCML7
CcCML42
MgCML42
AtCML42
AtCML43
OsCML32
PaCML42-1
PaCML42-2
PaCML5-2
PaCML5-1
PaCML5-3
SmCML4
SmCML3
AcCML25-1
PvulCML25-
BrCML25
CrCML25
AtCML25
BrCML25-1
ThCML25
CpCML25-1
EgCML25-1
MeCML25-1
MeCML25-2
PtCML25-1
PtCML25-2
GrCML25-3
GrCML25-5
TcCML25-3
MdCML25-3
MdCML25-4
PperCML25-
CsCML25-1
CsatCML25-
MgCML25-1
MtCML25-4
SlCML25-1
StCML25-1
MtCML25-3
PvulCML25
GrCML25-4
SlCML25-2
GrCML25-1
LuCML25-2
LuCML25-3
CsatCML25-
PaCML25-9
BdCML10

```
PvCML10-1
SbCML10-1
ZmCML10-1
OsCML10
PvCML10-2
BdCML15
OsCML15
PhCML10
PvCML15
SiCML10
SbCML10-2
ZmCML10
ZmCML10-2
PvCML10-3
AcCML25-2
PaCML25-3
PaCML25-4
PaCML25-2
PaCML25-5
PaCML25-1
PaCML25-7
PaCML25-6
PaCML25-8
AcCML27
AcCML23
CcCML23-1
CsCML23-1
SlCML24
StCML23-2
SlCML23
StCML23-3
MgCML27-2
GmCML27-1
GmCML27-2
PvulCML27-
MtCML24
GmCML27-4
PvulCML27-
CsatCML23
BrCML23
CrCML23
AtCML23
BrCML24
ThCML24
CrCML24
AtCML24
LuCLM24
ThCML23-2
MeCML23
PtCML23-1
PtCML23
CpCML24
GrCML23-1
GrCML23-2
TcCML23
EgCML24-1
FvCML23
MdCML27-5
MdCML27-6
PperCML27-
FvCML27-1
BrCML27-2
AtCML27
BrCML27-1
ThC27
BrCML26
CrCML26
AtCML26
ThCML26
CpCML27
CcCML27
CsCML27
GrCML27-2
TcCML27
GrCML27-3
CsatCML27
GmCML27-5
PvulCML27-
MtCML27
GmCML27-3
PvulCML27-
LuCML27-1
LuCML27-2
LuCML27-3
PtCML27-2
PtCML27-3
MeCML27-1
MeCML27-2
EgCML26
FvCML27-2
PperCML27-
MdCML27-1
MdCML27-2
MdCML27-3
GrCML27-1
MgCML27-1
SlCML27-2
SlCML27-1
EgCML23
EgCML24-2
BdCML16
OsCML16
PhCML16
PvCML16
SiCML16
SbCML16
ZmCML16
PhCML18
PvCML18-2
PvCML18-1
SbCML18
SiCML18
OsCML18
AcCML30
AcCML30-2
BrCML30
ThCML30
CrCML30
AtCML30
LuCML30-1
LuCML30-2
CcCML30-2
CsCML30-1
MeCML30-1
PtCML30-1
MdCML30-3
PperCML30-
MdCML30-4
VvCM30
GmCML30-1
PvulCML30-
MeCML30-3
CsatCML30-
MgCML30
SlCML30-2
StCML30-1
EgCML30-1
EgCML30-2
CcCML30-1
CsCML30-2
GrCML30-1
TcCML30
GrCML30-2
MeCML30-2
PtCML30-2
GmCML30-2
PvulCML30-
MtCML30-2
MtCML30-3
MtCML30-1
PvulCML30-
MdCML30-5
PperCML30-
CsatCML30-
SlCML30-1
StCML30-2
BdCML29
PhCML6
SbCML1
```

BdCML20
PhCML20
SbCML20
ZmCML20-2
ZmCML20-1
SiCML20
OsCML20
AcCML25-5
CcCML25
CsCML25-2
GmCML25
PvulCML25-
MtCML25-2
MdCML25-2
PperCML25-
TcCML25-2
PtCML25-3
CsatCML25
AcCML25-4
CpCML25-2
GrCML25-2
TcCML25-1
PtCML27-1
MtCML25-1
PvulCML25-
RcCML25
FvCML25
PperCML25-
MdCML25-1
CcCML23-2
CsCML23-2
MgCML25-2
StCML25-2
CsatCML36
PpCML23
PpCML25-3
PpCML25-8
PpCML25-9
PpCML25-6
PpCML25-1
PpCML25-2
PpCML25-11
PpCML25-10
PpCML25-4
PpCML25-5
PpCML25-7
PpCML25-12
SmCML27
SmCML26
BrCML36
ThCML36
CrCML36-1
AtCML36
CrCML36-2
BrCML35
CrCML35
AtCML35
CpCML35
MeCML36
FvCML36
MdCML36-1
PperCML36
MdCML36-2
MdCML36-3
MdCML36-4
MtCML36
CcCML36
CsCML36
LuCML36-1
LuCML36-2
MgCML36-1
MgCML36-2
SlCML36
StCML36-2
StCML36-3
SlCML35
StCML36-1
VvCML36
OsCML1-1
OsCML1-2
AcCML38
EgCML37
TcCML37
VvCML39-2
FvCML38-2
PperCML38-
RcCML39
CcCML37
BrCML37
AtCML37
ThCML39
CrCML37
CpCML37
SlCML38-1
StCML38-3
SlCML39-2
StCML37
StCML38-2
SlCML38-2
StCML38-1
BrCML39-1
ThCML23-1
BrCML39-2
CrCML39
AtCML39
BrCML38-2
ThCML38
BrCML38-1
AtCML38
CrCML38
AcCML41-2
AcCML41-1
AtCML41
EgCML41
CcCML41
CsCML41
MdCML41-2
MdCML41-1
MtCML41-1
PvulCML41
PtCML41
RcCML41
SlCML41
StCML41
CpCML40
CcCML38-2
CsCML38-2
FvCML38-1
MtCML41-2
PvulCML38-
PvulCML38-
EgCML40-2
MgCML38-2
MgCML38-1
MgCML38-3
CsatCML38-
CsatCML41
SlCML37-1
StCML38-4
SlCML37-2
StCML23-1
CcCML38-1
CsCML38-1
FvCML38-3
MdCML39-2
MdCML39-1
MdCML38
PperCML38-
TcCML38
MtCML38-1
MtCML38-2
MtCML38-3
PtCML37
PvulCML38-
CsatCML38-
EgCML40
EgCML39-1
EgCML38
EgCML39-2

```
      EgCML40-1
      VvCML39-1
     PvulCML38-
     PvulCML38-
      VvCML41-1
      VvCML41-2
        VvCML38
      VvCML41-3
        LuCML38
     CsatCML38-
        AtCML40
        AcCML39
        BdCML31
        OsCML31
        PhCML31
        SbCML31
        PhCML19
        SiCML19
        OsCML19
        SbCML19
        ZmCML19
        BdCML23
        SiCML23
        OsCML23
        OsCML25
        OsCML26
      SlCML39-1
        AcCML21
        MtCML21
        VvCML21
        CpCML21
        TcCML21
        CcCML21
        CsCML21
        FvCML21
      PperCML21
        MgCML21
        SlCML21
        StCML21
        CrCML21
        AtCML21
        ThCML21
      CsatCML21
        SiCML33
        SbCML33
        OsCML33
        SiCML29
        SbCML29
        OsCML29
        PaCML21
        PpCML21
        AcCML22
        CsCML22
        TcCML22
      PperCML12
        FvCML22
      CsatCML22
        MgCML22
        StCML22
        ThCML22
        AtCML22
        BdCML32
        SbCML34
        OsCML34
      SmCML21-1
      SmCML21-2
        MpCML12
      BrCML32-1
        PvCML36
        PvCML29
      ZmCML36-1
        MeCML32
      ZmCML36-3
      ZmCML36-2
        MpCML11
     CreinCML20
     CreinCML20
        AtCML48
        AtCML49
        AtCML50
     CreinCML32  ------KDLA AVKAKLTEQG ITGIKSVALY GDMSSTESGS GPSTSAPPRT PGAPPRSPAG TAAA------ ------GAPA SSPEPSMRPG TGRRGANPDH NRSSIIFG
        RcCML23  SSLKLQKEKS IKKMSLRALT KSGILVVLGV AIVSSLGMPL INNTQLLSER IGISSFYISF VVLP------ ------FAVN FKTAMATIFP ASQKKEEASS IMFSEIYGAV FMNNVSGLLT LLALIWARGF TWDYSAEVIV LLVVSAIIGA IAFLRRIYPL WTCLLAFSFY PLSLVLFYVI
         SbCML6
        SbCML15
         VcCML5  ------NVLA VAKAPEAATE KPALAEVLLA EDF
        AcCML18  ------GSLT QLELGSLLRS LGL--KPS-A DQLDALIQKA DI-NSNG-LV EFSEFVALVA PDLL------ PA----KSPY TEDQLKKLFK MFDRDGNGYI TAAELAHSMA KLGHALTAKE LTGMIKEADT DGDGCINFAE FSQAITSAAF -DNSWA
        MeCML18  ------GSLT QLELGSLLRS LGL--KPS-E DQLEALTQKA DK-NNNG-LI EFSEFVALVE PDLL------ QT----KCPY TEDQLKKIFS MFDRDGNGYI TPAELAHSMA KLGHALTAEE LTGMIKEADT DGDGCINFQE FAQAITSAAF -DNSWC
        RcCML18  ------GSLT QLELGSLLRS LGL--KPS-E DQLEALIQKA DK-NSNG-LI EFSEFVALVE PDLV------ QA----KSPY TEDQLKKIFT MFDRDGNGYI TPAELAHSMA KLGHALTAEE LTGMIKEADT DGDGCISFQE FTQAITSAAF -DNSWC
        SlCML18  ------GSLT QLELGSLLRS LGL--KPS-N DQLEDLIQKA DR-NSNG-LI EFSEFVALVA PELI------ SA----KCPY SEEQLKKIFQ MFDRDGNGVI TAAELAHSMA KLGHALTQEE LTGMIKEADR DGDGCISFEE FAQAMTSAAF -DNSWT
      StCML18-1  ------GSLT QLELGSLLRS LGL--KPS-N DQLEDLIQKA DR-NSNG-LI EFSEFVALVA PELI------ AA----KCPY SEEQLKKIFR MFDRDGNGVI TAAELAHSMA KLGHALTQEE LTGMIKEADR DGDGCISFEE FAQAMTSAAF -DNSWT
        CpCML18  ------GSLT QLELGSLLRA LGL--KPS-A DQIETLIQKA DT-NSNG-LV EFSEFVALVS PELL------ SA----KSPY SEEQLRQLFR MFDRDGNGFI TAAELAHSMA KLGHALTAEE LTGMIREADT DGDGRISFQE FSQAITSAAF -DNSWA
        MgCML18  ------GSLT ELELGSLLRS LGL--KPS-P DQLDALIQRA DT-NSNG-LV EFSEFVALVA PELL------ PA----KSPY TEEQLKHLFK MFDRDGNGYI TAAELAHSMA NLGHALTAEE LTGMIREADT DGDGRISFQE FSQAITSAAF -DNSWA
      PtCML18-1  ------GSLT QLELGSLLRS LGL--KPS-P DQLETLIHKA DT-NSNG-LI EFSEFVALVA PELL------ PE----KSPY SEEQLKHLFK MFDRDGNGFI TAAELAHSMA KLGHALTAEE LTGMIKEADT DGDGRISFQE FSQAITSAAF -DNSWV
      PtCML18-2  ------GSLT QLELGSLLRS LGL--KPR-P DQLETLIQKA DT-NSNG-LI EFSEFVALVA PELL------ PE----KSPY SEEQLKHLFK MFDRDGNGFI TAAELAHSMA KLGHALTAEE LTGMIKEADT DGDGRISFEE FSQAITSAAF -DNSWV
        CcCML18  ------GSLT QLELGSLLRS LGL--KPS-P DQLETLIQKA DT-NSNG-LV EFSEFVALVA PELL------ SS----KSPY SEDQLRQLFR MFDRDGNGFI TAAELAHSMA KLGHALTAEE LTGMIREADT DGDGRISFQE FAQAITSAAF -DNSWA
        CsCML17  ------GSLT QLELGSLLRS LGL--KPS-P DQLETLIQKA DT-NSNG-LV EFSEFVALVA PELL------ SS----KSPY SEEQLRQLFR MFDRDGNGFI TAAELAHSMA KLGHELTAEE LTGMIREADT DGDGRISFQE FAQAITSAAF -DNSWA
      FvCML18-2  ------GSLT QLELGSLLRS LGL--KPG-P DQLDTLIQKA DT-NSNG-LV EFSEFVALVA PELV------ SA----KSPY TEDQLRQLFR MFDRDGNGFI TAAELAHSMA KLGHALTAEE LTGMIREADT DGDGRINFQE FAHAITSAAF -DNSWA
      PperCML18  ------GSLT QLELGSLLRS LGL--KPG-P DQLDTLIQKA DT-NSNG-LV EFSEFVALVA PELV------ SA----KSPY TEDQLRQLFR MFDRDGNGFI TAAELAHSMA KLGHALTAEE LTGMIREADT DGDGRIDFQE FAHAITSAAF -DNSWS
        MdCML18  ------GSLT QLELGSLLRS LGL--KPG-P EQLDALIQKA DT-NSNG-LV EFSEFVGLVA PELL------ SA----KSPY TEDQLRQLFR MFDRDGNGFI TAAELAHSMA KLGHALTAEE LTGMIKEADT DGDGRINFQE FAHAITSAAF -DNSWS
        TcCML18  ------GSLT QLELGSLLRS LGL--KPS-P DQVEALIQKA DT-NSNG-LV EFSEFVALVA PELL------ SE----KSPY SEEQLKQLFK MFDRDGNGFI TAAELAHSMA KLGHALTVEE LTGMIKEADT DGDGRISFEE FAQAITSAAF -DNSWA
        EgCML17  ------GSLT QLELGSLLRS LGL--KPS-P EQLEALIQKA DT-NSNG-LV EFSEFVALVS PQLL------ PA----KSPY TEEQLKQLFQ MFDRDGNGFI TAAELAHSMA RLGHALTPEE LTGMIKEADT DGDGRISFRE FSQAITSAAF -DNSWA
      StCML18-2  ------GSLT QLELGALLRS LGL--KPS-P DQLETLIQKA DK-NDNG-LV EFSEFVSLVA PELL------ PA----KSPY TEEQLKQLFK MFDRDGNGYI TAAELAHSMA KLGHALTAEE LTGMIREADT DGDGRISYQE FTQAISSAAF -DNSWA
      GrCML18-2  ------GSLT QLELGSLLRS LGL--NPS-S DQVEALIHKA DT-NNNG-LV EFSEFVSLMA PELL------ SE----KSPY SEEQLKQLFK MFDRDGNGYI TAAELAHSMA KLGHALTAEE LTGMIKEADT DGDGMISFEE FSNAITSAAF -DNSWG
        GmCML17  ------GSLT QLELSSLLRS LGL--KPS-A DQLEGFIQRA DT-NSNG-LV EFSEFVALVA PELL------ PA----KSPY TEEQLKQLFR MFDRDGNGLI TAAELAHSMA RLGHALTAEE LTGMIKEADT DGDGMINYQE FAHAITSAAF -DNCWP
      PvulCML18  ------GSLT QLELSSLLRS LGL--KPS-A DQLEVFIQRA DT-NNNG-LV EFSEFVALVA PELL------ PA----KSPY TDEQLRQLFR MFDRDGNGFI TAAELAHSMA RLGHALTAEE LTGMIKEADT DGDGMINFQE FCHAITSAAF -DNSWP
        MtCML18  ------GTLT QLELNSLLRS LGL--KPS-A EQLEGFIQRA DT-NNNG-LI EFSEFVALVA PELL------ PA----KSPY TEEQLRQLFR MFDRDGNGFI TAAELAHSMA KLGHALTAEE LTGMIKEADM DGDGMISFQE FAQAITSAAF -DNSWV
      CsatCML18  ------GSLT QLELGSLLRS LGL--KPS-P DQLESLIMKA DT-NSNG-LV EFSEFVALVE PELV------ SA----KCPY TEEQLKQLFR MFDRDGNGFI TAAELAHSMA KLGHALTAEE LTGMIREADT DGDGRINFQE FSQAITSAAF -DNSWA
      GrCML18-1  ------GSLT QLELGSLLRS LGL--KPS-L DQVEALIQKA DS-NNNG-LI EFSEFVAMLA PELQ------ PE----KSPY SEEQLKQLFK MFDRDGNGFI TAAELAQTMA KLGHALTVEE LTGMIKEADA DGDGRISFDE FSQAITSAAF HNNSWA
        BdCML11  ------GSLT QLELGSLLRS LGL--KPS-A DELDALIQRA DL-NSNG-LV EFSEFVALVA PELL------ DD----RSRY SEDQLRRLFE IFDRDGNGFI TAAELAHSMA RLGHALTAKE LTGMIEEADT DGDGRIDFHE FSRAITAAAF -DNVFS
        PhCML11  ------GSLT QLELGSLLRS LGL--KPS-T DQLDALISRA DT-NSNG-LV EFSEFVALVA PDLL------ VD----RSPY SEDQLRKLFS IFDRDGNGFI TAAELAHSMA KLGHALTVKE LTGMIKEADT DGDGRINFQE FSRAITAAAF -DNIFS
        SbCML11  ------GSLT QLELGSLLRS LGL--TPS-A DQLDALITRA DT-NSNG-LV EFSEFVALVA PDLL------ AD----RSPY SEDQLRKLFA IFDRDGNGFI TAAELAHSMA RLGHALTVKE LTGMIKEADT DGDGRINFQE FSRAITAAAF -DNILY
        ZmCML11  ------GSLT QLELGSLLRS LGL--TPS-A DQLDALITRA DT-NSNG-LI EFSEFVALVA PDLL------ QD----RSPY SEDQLRKLFA IFDRDGNGFI TAAELAHSMA KLGHALTVKE LTGMIKEADT DGDGRINFQE FSRAITAAAF -DNIFS
        OsCML11  ------GSLT QLELGSLLRS LGL--KPS-T DELDSLIQRA DT-NSNG-LI EFSEFVALVA PELL------ YD----RAPY SEDQIRRLFN IFDRDGNGFI TAAELAHSMA KLGHALTVKE LTGMIKEADT DGDGRISFQE FSRAITAAAF -DNIFS
        GmCML18  ------GSLT QLELSSLLRS LGL--KPS-A EQLEGFIQRA DT-NNNG-MV EFSEFVALVA PDLL------ PA----KSHY TEDQLRHLFR MFDRDGNGLI TAAELAHSMA RLGHALTVEE LTGMIKEADT DGDGMINFQE FAHAITSAAW P
        BrCML18  ------GSLT ELELAALLRS LGL--KPS-Q DQLDTLIHKA DR-NNNG-II EFSEFVALVE PDLV------ K------CPY TEDQLKAIFK MFDRDGNGYI TAAELAHSMA KLGHALTAEE LTGMIREADR DGDGCIDFQE FVQAITSAAF -DNAWG
        ThCML18  ------GSLT ELELASLLRS LGL--KPS-Q DQLDTLIHKA DR-NNNG-MI EFSEFVALVE PDLV------ K------CPY TDEQLRAIFK MFDRDGNGYI TAAELAHSMA KLGHALTAEE LTGMIKEADR DGDGCIDFQE FVQAITSASF -DNAWG
        CrCML18  ------GSLT ELELGSLLRS LGL--KPS-Q DQLDTLIHKA DR-NNNG-LV EFSEFVALVE PDLV------ K------CPY TDEQLKAIFK MFDRDGNGYI TAAELAHSMA KLGHALTAEE LTGMIKEADR DGDGCIDFQE FVQAITSAAF -DNAWG
        AtCML18  ------GSLT ELELGSLLRS LGL--KPS-Q DQLDTLIQKA DR-NNNG-LV EFSEFVALVE PDLV------ K------CPY TDDQLKAIFR MFDRDGNGYI TAAELAHSMA KLGHALTAEE LTGMIKEADR DGDGCIDFQE FVQAITSAAF -DNAWG
      LuCML18-1  ------GSLT QLELGSLLRS LGL--KPS-E DQLDRMIHKA DT-NNNG-LI EFSEFVALVE PDLV------ KS----KCPY TEEQLRQMFT LFDRDGNGFI TAAELAHSMA KLGHALTAAE LTSMIREADG DGDGCISFQE FAKAVTSASF -DNFWCGHTG R
        BrCML17  ------GSLT QLELGSLLRA LGL--KPS-P DQFETLIDKA DT-KSNG-LV EFPEFVSLVS PELL------ TEEKT-RTPY TEEQLLRLFR IFDTDGNGFI TAKELAHSMA RLGHALTVAE LTGMIKEADS DGDGRINFQE FSKAINSAAF -DDLWG
        CrCML17  ------GSLT QLELGSLLRA LGI--KPS-P DQFETLIDKA DT-KSNG-LV EFPEFMALVS PELL------ SSTKR-TTPY TEEQLLRLFR IFDTDGNGFI TAAELAHSMA KLGHALTVAE LTGMIKEADS DGDGRINFQE FAKAINSAAF -DDLWG
        AtCML17  ------GSLT QLELGSLLRA LGV--KPS-P DQFETLIDKA DT-KSNG-LV EFPEFVALVS PELL------ SPAKR-TTPY TEEQLLRLFR IFDTDGNGFI TAAELAHSMA KLGHALTVAE LTGMIKEADS DGDGRINFQE FAKAINSAAF -DDIWG
        ThCML17  ------GSLT QLELGSLLRA LGL--KPS-P DQFETLIDNA DT-KSNG-LV EFSEFVALVS PEIL------ AAAKT-LTPY TEEQLLRLFR IFDTDGNGFI TAAELAHSMA KLGHALTVAE LTGMIKEADS DGDGRINFQE FSKAINSAAF -DDLWG
        SlCML17  ------GSLT QLELGSLLRA LGV--KPS-P DQFETLIDKA DT-KSNG-LV EFPEFVALVS PELL------ SPAKR-TTPY TEEQLLRLFR IFDTDGNGFI TAAELAHSMA KLGHALTVAE LTGMIKEADS DGDGRINFQE FAKAINSAAF -DDIWG
      FvCML18-1  ------GSLT PLELRSLLRS LGL--EAS-H EQVEALMQKA DR-NDNG-LV EFSEFVVLVE PDLL------ QLQGG-KCPY SEEMLKSMFD MFDRDGNGYI SAAELAHSMA KLGHALTAEE LIGMMEEADT DGDGQIDFQE FVRAITSAAF -HNRWT
      LuCML18-2  ------GSLT QLELGSLLRS LGL--SPS-P DQLDALIKTA DT-NSNG-TV EFSEFLALVA PDLV---RQH PAEEE-KSPY SEEKLKQLFR MFDRDGNGYI TAAELAHSMA KLGHALTAEE LTGMITEADT DGDGRISFPE FSRAISSAAF QNSSWG
        EgCML18  ------GSLT QLELGSLLRS LGL--KPS-P DQLEALIHKA DR-NSNG-LV EFSEFVALVE PDLI------ PA----KCPY TEDQLKKIFH MFDRDGNGYI TAAELAHSMA KLGHALTAEE LTGMIKEADT DGDGCINFQE FVQAITSAAF -DNSWG
      MdCML17-3  ------GSLT QLELGSLLCS L--------- --VDALIQKA DT-NNNG-LV KFLEFIGLVA PELL------ ---SA-KSPY TDDQLRQLFQ MFNRHGNGFI TVTELAHSMV KLDHALTTEE LTGIIKEADT DEDGRINFHE FSHAITSVAF -DSSWS
      MdCML17-2  ------GSLT EPELGSLLRS LGL--KPS-H EQLQALIRKS DT-NHNG-LV GFSEFVALMR PDLN---GLL QDDARCPCSY SEDQLREMFS MFDRDANGYI SPAELAHSMA KLGHPLTVQE LFGMMNEADT DRDGLIDFHE FSRAITSAAF LNINA
      PpCML17-1  ------GSIT ELELGLMLRS LGL--KPE-G YQLDSLLRRA DT-NSNG-MI EFAEFVALMG PELV---K-- ------TVAY NDKELLTVFR AFDRDGNGFI TAAELAHSMA KLGQTLSVKE LWTMIREADI DGDGRISFPE FAAAMTTASS LDIPV
      PpCML17-2  ------GSIT ELELGLMLRS LGL--KPE-G HQLEALLQRA DT-NSNG-MI EFAEFVSLMG PELV---K-- ------AVVY SDKELLTVFR AFDRDGNGYI TAAELAHSMA KLGQTLSVTE LRTMIREADS DGDGRISFSE FSAAMTCASR LDVHV
        AcCML16  ------GSLT HLELAALLRS LGL--KPT-G DQLHVMLSNM DA-NGNG-AV EFEELVNAIL P--------- --DMNEEILI NQEQLLEVFQ SFDRDGNGYI TPAELAGSMA KMGHPLSYRE LTEMMREADM DGDGVISFGE FATIMARSAA DFLGIAVS
        FvCML15  ------GSLT ILELGALLRS LGL--RPS-G DQMHVLLSNI DA-NGNG-SV EFDELVKAIL P--------- --DMNEEIFI NQQQLMEVFQ SFDRDGNGYI TAAELAKSMA KMGQPLSYKE LTEMIKEADT DGDGVISFNE FATIMAKSAS GYLGLGLS
        BdCML12  ------GSLT KLELVALLRS LGL--RPAAG DEIHALVATM DA-DGNG-TV EFDELTSSLA QLLL------ -GPCRPSVAV DHEQLAEAFR AFDRDGNGYI SAAELARSMA QMGHPICYAE LTDMMREADT DGDGSISFEE FTAIMAKSAV DFLGLAAL
        PhCML12  ------GSLT MLELAALLRS LGL--RPAAG DEIHALIAAM DA-DGNG-TV EFDELASSLA PLLL------ -GPCRPAVAV DQAQLAEAFR AFDRDGNGFI SAAELARSMA RLGHPICYAE LTDMMREADT DGDGVISFQE FTAIMAKSAV DFLGLAAL
        PvCML12  ------GSLT LLELAALLRS LGL--RPAAG DEIHALIAAM DA-DGNG-TV EFDELASSLA PLLL------ -GPCRPAVAV DQAQLAEAFR AFDRDGNGFI SAAELARSMA RLGHPICYAE LTDMMREADT DGDGVISFQE FTAIMAKWLL RVLVLGLLR
        SiCML12  ------GSLT MLELAALLRS LGL--RPAAG DEIHALIAAM DA-DGNG-TV EFDELASSLA PLLL------ -GPCRPAVAV DQAQLAEAFR AFDRDGNGFI SAAELARSMA RMGHPICYDE LTDMMREADT DGDGVISFQE FTAIMAKSAV DFLGLAAL
        SbCML12  ------GSLT KLELAALLRS LGL--RPAAG DEIHALIAAM DA-DGNG-TV EFDELASSLA PLLL------ -GPCRPAVAV DHAQLAEAFR AFDRDGNGFI SAAELARSMA LMGHPICYAE LTDMMKEADT DGDGVISFQE FTAIMAKSAV DFLGLAAL
      ZmCML14-1  ------GSLT KLELAALLRS LGL--RPAAG DEIHTLIAAM DA-DGNG-TV EFDELSSSLA PLLL------ -GPCRPAVAV DHAQLAEAFR AFDRDGNGFI SAAELARSMA LMGHPICYAE LTDMMKEADT DGDGVISFQE FTAIMAKSAV DFLGLAAL
        OsCML12  ------GSLT KLELAALLRS LGL--RPAAG DEIHALIAAI DA-DGNG-TV EFDELASSLA DLIL------ -GPCRPSVAV DQAELAEAFR AFDRDGNGFI SAAELARSMA RMGHPICYAE LTDMMREADT DGDGLISFEE FTAIMAKSAL DFLGLAAL
        BdCML14  ------GSLT QLELAALLRS LGL--RPT-G DDARALLAGM DA-DGNG-AV EFEELASAIA PLLL------ -SPSAAGL-V DRDQLLEVFR AFDRDGNGYI SAAELARSMA RIGQPLTFQE LTATMREADA DGDGVISFQE FAAVMAKSAL DFLGVPT
        PhCML14  ------GSLT QLELAALLRS LGL--RPT-G EEVRALLVGM DA-DGNG-AV EFDELAAAIA PLLT------ -TQTH--L-V DQAQLLEVFR AFDRDGNGFI SAAELARSMA RLGQPLTFEE LTRMMRDADA DGDGVISFKE FAAVMAKSAL DFLGIA
      PvCML14-1  ------GSLT QLELAALLRS LGL--RPT-G EEVRALLAGM DA-DGNG-AV EFDELADAIA PLLT------ -TQTH--L-V DQAQLLEVFR AFDRDGNGFI SAAELARSMA RLGQPLTFEE LTRMMRDADA DGDGVISFQE FAAVMAKSAL DFLGVA
      PvCML14-2  ------GSLT QLELAALLRS LGL--RPT-G EEVRALLAGM DA-DGNG-AV EFDELAAAIA PLLT------ -TQTH--L-V DQAQLLEVFR AFDRDGNGFI SAAELARSMA RLGQPLTFEE LTRMMRDADA DGDGVISFQE FAAVMAKSAL DFLGVA
        SiCML14  ------GSLT QLELAALLRS LGL--RPT-G EEVRALLAGM DA-DGNG-AV EFEELAAAIA PLLT------ -TQTH--L-V DQDQLLEVFR AFDRDGNGYI SAAELARSMA RLGQPLTFEE LTRMMRDADA DGDGVISFQE FAAVMAKSAL DFLGVA
        SbCML14  ------GSLT QLELAALLRS LGL--RPT-G EEARALLAAM DS-DGNG-LV EFGELAAAIA PLLT------ -TQTH--L-V DQAQLLEVFR AFDRDGNGFI SAAELARSMA RLGQPLTFEE LTRMMRDADA DGDGVISFQE FAAVMAKSAL DFLGVA
      ZmCML14-2  ------GSLT QLELGALLRS LGL--RPT-G EEARALLAAM DS-NGNG-AV EFGELAAAIA PLLT------ -TQTH--L-V DQAQLLEVFR AFDRDGNGYI SAAELARSMA RIGQPLTFEE LTRMMRDADA DGDGVISFNE FAAVMAKSAL DFLGVA
        OsCML14  ------GSLT QLELAALLRS LGL--RPT-G DEVHALLAGM DA-NGNG-SV EFDELAAAIA PVLT------ -TQTH--L-V DQAQLLEVFR AFDRDGNGFI SAAELARSMA RLGQPLTFEE LTRMMRDADT DGDGVISFKE FAAVMAKSAL DFLGVA
      BrCML16-1  ------GSLT HLELAALLRS LGI--KPR-G DQISLLLNQI DR-NGNG-SV EFDELVVAIM PDL------- -NEE---VLI NQEQLMEVFR SFDRDGNGQI TAAELAGSMA KMGHPLTYRE LTEMMTEADS NGDGVISFNE FAHIMAKSAA DFLGLTAAA
      BrCML16-2  ------GSLT QLELAALLRS LGI--KPR-G DQISLLLNQI DR-NGNG-SV EFDELVVAIL PDL------- -NEE---VLI NQEQLMEVFR SFDRDGNGQI TAAELAGSMA KMGHPLTYRE LTEMMTEADS NGDGVISFNE FAHIMAKSAA DFLGLTAA
        ThCML16  ------GSLT QLELAALLRS LGI--KPR-G DQISLLLNQI DR-NGNG-SV EFDELVVAIL PDI------- -NEE---VLI NQEQLMEVFR SFDRDGNGQI TAAELAGSMA KMGHPLTYRE LTEMMTEADS NGDGVISFNE FAHIMAKSAA DFLGLTAAS
        AtCML16  ------GSLT QLELAALLRS LGI--KPR-G DQISLLLNQI DR-NGNG-SV EFDELVVAIL PDI------- -NEE---VLI NQEQLMEVFR SFDRDGNGSI TAAELAGSMA KMGHPLTYRE LTEMMTEADS NGDGVISFNE FSHIMAKSAA DFLGLTAS
        CrCML16  ------GSLT QLELAALLRS LGI--KPR-G DQISLLLNQI DR-NGNG-SV EFEELVVAIL PDI------- -NEE---VLI NQEQLMEVFR SFDRDGNGSI TAAELAGSMA KMGHPLTYRE LTEMMREADS NGDGVISFNE FAHIMAKSAS DFLGLAV
        CpCML16  ------GSLT QLELGALLRS LGI--KPT-G DQLHLLLANI DS-NGNG-SV EFDELISAIL PDM------- -NEQ---VLI NQEQLMEVFR SFDRDGNGYI TASELAGSMA KMGHPLTYRE LADMMREADT NGDGVISFHE FANVMAKSAA DFLGLTVA
      MeCML16-1  ------GSLT QLELAALLRS LGL--KPS-G DQIHVLLSNM DS-NGNG-YV EFDELVSAIL PDM------- -NEQ---VLI NQDQLMQVFQ SFDRDGNGYI TASELAGSMA KMGQPLSYQE LAEMMKEADT NGDGVISFNE FANIMAKSAA EFLGLAMQQ
        PtCML16  ------GSLT QLELAALLRS LGL--KPT-G DQLHVLLSNM DA-NGNG-YV EFDELVSAIL PDM------- -NEE---VLI NQEQLLEVFR SFDRDGNGFI TAAELAGSMA KMGHPLTYRE LSDMMREADT NGDGVLSFNE FANVMAKSAA DFLGIKVP
        RcCML16  ------GSLT QLELAALLRS LGL--KPT-G DQLQILLSNM DA-NGNG-YV EFDELVNAIL PDM------- -NED---VLI NQEQLLEVFR SFDRDGNGYI TAAELAGSMA KMGHPLTYKE LSEMMREADT NGDGVISFNE FANIMAKSAS DFLGI
        MgCML26  ------GSLT QLELAALLRS LGL--KPN-G DQIHVLLANM DA-NGNG-SI EFDELVEAIL PDM------- -NEQ---VLI NQDQMMEVFR SFDRDGNGYI TAAELAGSMA KMGHPLTYRE LSEMMREADT NGDGVISFNE FANILGKSAV DYLGLSVS
      PperCML16  ------GSLT QLELAALLRS LGV--KPT-G DQLHVLLANM DA-NGNG-TV EFDELVTAIL PDM------- -NAE---ILI NQEQLTEVFR SFDRDGNGYI TAAELAGSMA KMGHPLTYRE LSDMMQEADT NGDGVISFSE FATIMSRSAA DFLGV
```

```
 VvCML16-2  ------GSLT QLELAALLRS LGL--KPT-G DQLQVLLTNM DA-NGNG-SI EFDELVEAIL PDL------- -NEQ---ILV NQEQLTEVFR SFDRDGNGYI TASELAGSMA KMGSPLSYRE LSDMMREADI NGDGVISFNE FATIMAKSAA DFLGLAVF
   CcCML16  ------GSLT QLELAALLRA LGL--KPT-G DQLHILLADM DS-NRNG-LV EFDELVALIL PDI------- -SEQ---VLV NQEQLMEVFR SFDRDGNGQI TAAELAGSMA KMGHPLTYGE LSEMMREADT NGDGVISFNE FATIMAKSAA DFLGLKLA
   CsCML16  ------GSLT QLELAALLRA LGL--KPT-G DQLHILLADM DS-NGNG-LV EFDELVALIL PDI------- -SEQ---VLI NQEQLMEVFR SFDRDGNGHI TAAELAGSMA KMGHPLTYGE LSEMMREADT NGDGVISFNE FATIMAKSAA DFLGLKLA
 LuCML15-2  ------GSLT HLELAALLRS LGL--KPT-G DQLHSLLSNI DS-NGNG-YV EFDELVSAIL PDL------- -NEE---VLI NQEQLLEVFQ SFDRDGNGYI TAAELAGSMA KMGHPLSYKE LSEMMRQADT NGDGVISLHE FANILAKSAA DFLGVAVRRP
 MeCML16-2  ------GSLT QLELAALLRS LGL--KPS-G DQIHVLLSNM DA-NGNG-YV EFDELVTAIL PDM------- -NEE---VLI NQEQLLEVFQ SFDRDGNGYI SAAELAGSMA KLGHPLTYQE LSQMMNEADT NGDGVLSLHE FAHIMARSAA DFLGLALQA
   SlCML16  ------GSLT QLELAALLRA LGL--KPT-G DQIHTLLANM DN-NGNG-FI EFDELVNAIM PDI------- -NAE---ILI NQEQLMEVFR SFDRDGNGYI TAAELAGSMA KMGRPLTYKE LSDMMQEADT NGDGVISFNE FANIMGKSAA DILGLTIS
   GmCML16  ------GSLT HLELAALLRS LGI--KPT-G DEIYALLSNM DE-NGNG-YI EFDELVHAIM PDL------- -TES---VLI NQEQLLEVFR SFDRDGNGYI TASELAGSMA KMGQPLTYRE LASMMAEADS NGDGVISFNE FAALMAKSAA EFLGVKVA
 PvulCML16  ------GSLT HLELAALLRS LGI--KPT-G DEIYALLSNM DE-NGNG-YI EFDELLHAIM PDL------- -NQD---VLI NQEQLLEVFR SFDRDGNGYI TASELAGSMA KMGHPLTYHE LASMMAEADS NGDGVISFNE FAALMAKSAA DFLGLNVA
 EgCML15-2  ------GSLT HLELAALLRS LGL--KPT-G DQIHAMLAEM DA-NSNG-SV EFDELVGALL PDM------- -TEQ---VFI NQEQLAEVFQ LFDRDGNGYI TPAELAGAMA KMGHPLTYRE LSEMMREADT NGDGVISFQE FATIMARSAV DFLGLR
   CpCML15  ------GSLT ILELAALLRS IGI--KPS-G DQIHVLLANM DS-NGNG-AV EFDELVTAIL PDM------- -NEE---VLI KQEQLMEVFR SFDRDSNGYI SAAELAGAMA KMGQPLTYKE LREMITEADT DGDGVISFNE FASVMAKSAY EFFGFHFS
   CcCML15  ------GSLT ILELAALLRS LGL--KPS-G DQIHVLLANM DA-NGNG-AV EFDELVNAIL PDM------- -DET---ILI NQEQLLEIFR AFDRDGNGYI TAAELAGSMA KMGQPLTYKE LTEMIKEADT NGDGVISFNE FAAVMAKSTL DFFGLGLS
   CsCML15  ------GSLT ILELAALLRS LGL--KPS-G DQIHVLLANM DA-NGNG-AV EFDELVNAIL PDM------- -DET---ILI NQEQLLEIFR AFDRDGNGYI TAAELAGSMA KMGQPLTYKE LTEMIKEADT NGDGVISFNE FAAVMAKSTL DFFGLGLS
 CsatCML15  ------GSLT ILELAALLRS LGL--KPS-G DQIHVLLANM DS-NGNG-SV EFDELVTAIM PDF------- -NEE---VMV NQTQLLEVFR SFDRDGNGYI TAAELAGSMA KMGQPLTYRE LTEMMKEADT DGDGVISFNE FASVMAKSAA DFLGLAIS
 MdCML15-2  ------GSLT ILELAALLRS LGL--KPS-G DQIHVLLANM DA-NGNG-SV EFDELVSAIL PDM------- -NEE---ILV NQEQLLEVFR SFDRDGNGYI TAAELAGSMA KMGQPLTYKE LTEMIKEADT DGDGVISFNE FSTIMAKSAS SFLGLAPS
 PperCML15  ------GSLT ILELAALLRS LGL--KPS-G DQIHVLLANM DS-NGNG-SV EFDELVSAIL PDM------- -NEE---ILG NQEQLLEVFR SFDRDGNGYI TAAELAGSMA KMGQPLTYKE LTEIIKEADT DGDGVISFNE FATIMAKSAS SFLGLAPS
 MdCML15-1  ------GSLT ILELTTLLRS LGL--KSS-G DQVHVLLANM DA-NGNG-SV EFDGLVSAIL PDM------- -DEE---IMV NQKQLLEVFR SFEHDGNGYI TAAELAWSMA KMGQPLTYKE VTETIKEADT DGDGVISFNE FATIMAKSAS SFLGLAAS
 GrCML15-2  ------GSLT VLELAALLRS LGL--KPS-G DQIHALLAKM DS-NGNG-AV EFDELANTIL PTM------- -KDE---ILV NQEQLTEVFR LFDRDGNGYI TAAELAGCMA KMGHPLTYKE LTDMIKEADA DGDGVISFNE FSSVMAKSAL EFLGISLSSS
 GrCML15-3  ------GSLT ILELAALLRS LGL--KPT-G DQIHVLLANM DS-NGNG-AV EFEELANAIV PHM------- -NEQ---VLV NQEQLMEVFH LFDRDGNGYI TPAELAGCMA KMGQPLSYRE LTEMIREADA DGDGVISFNE FSTVMARSAL DFLGVRLSM
   TcCML15  ------GSLT ILELAALLRS LGL--KPS-G DQIHVLLANM DS-NGNG-AV EFDELVNAIL PDM------- -NEQ---ILV NQEQLREVFH LFDRDGNGYI TAAELAGCMA KMGQSLTYRE LTEMIKEADT DGDGVISFNE FSSVMAKSAL DFLGITLSC
   MeCML15  ------GSLT ILELGALFRS LGL--KPS-G DQIHVLLSNM DS-NGNG-FI EFDELVSAIL PDM------- -NEQ---LVI NQEQLLYVFQ LFDRDGNGYI TAAELAGSMA KMGQPLTYTE LKEMIEEADT DGDGVISFNE FASVMAQSAM EFLGLATSSL SDGML
   RcCML15  ------GSLT ILELAALLRS LGL--KPS-G DQIHVLLANM DS-NGNG-SV EFDELASAIL PDM------- -NEE---TFV NQEQLLDVFQ LFDRDGNGYI SAAELAGSMA KMGQPLTYKE LREMIKEADT DGDGVISFSE FTSVMARSTM EFLGFTLS
   PtCML15  ------GSLT ILELAALLRS LGL--KPS-G DQIHVLLAGM DS-NGNG-SI EFEELVHAIL PDV------- -NEE---VLV NQEKLLEVFH IFDRNGNGYI SAAELAGSMA KMGQPLTYKE LTEMIEEADT DGDGVISFNE FATVMAKSAM EFLGIT
 VvCML16-1  ------GSLT LLELAALLRS LGL--KPS-G DQIHSLLANI DS-NGNG-SV EFDELVSAIM PDM------- -NEE---ILI NQQQLLEVFR SFDRDRNGYI TAVELAGAMA KMGQPLTYRE LTDMIREADT NGDGVISFNE FSSIMAKSAG DFLGLTLL
   SlCML15  ------GSLT HLELAALLRS LGL--KPN-G DQIHVLLAKM DS-NGNG-SV EFDELVNAIT PDLI------ -NEQ---VLV SQGQLLEVFQ SFDRDGNGYI TLAELAGSMA KMGQPLSYRE LTEMIKEADV DGDGVISFNE FATVMARSAA DFLGVPIL
   StCML15  ------GSLT QLEFAALLRS LGL--KPN-G DQIHVLFAKM DS-NGNG-SV EFDELVNAIT PDLI------ -NGE---VLV NKGQLLEVFR SFDRDGNGYI TLAELAGSMA KMGQPLSYRE LTEMIKEADV DGDGVISFNE FATVMARSAA EFLGVPIL
   BrCML15  ------GSLT ILELAALLRS LGL--KPT-G DQIHLLLASM DA-NGNG-FV EFDELVGAIL PDL------- -NEE---VLI NSEQLMNIFK SFDRDGNGFI SAAELAGAMA KMGQPLTYRE LTEMIQEADT NGDGVISFGE FASIMAKSAV EYFGLKINS
   CrCML15  ------GSLT ILELAALLRS LGL--KPS-G DQIHVLLASM DA-NGNG-FV EFDELVGTIL PDL------- -NEE---ILI NSEQLLDIFK SFDRDGNGFI SAAELAGAMA KMGQPLTYKE LTEMIKEADT NGDGVISFGE FASIMAKSAV DYFGLKINS
   AtCML15  ------GSLT ILELAALLRS LGL--KPS-G DQIHVLLASM DS-NGNG-FV EFDELVGTIL PDL------- -NEE---VLI NSEQLLEIFK SFDRDGNGFI SAAELAGAMA KMGQPLTYKE LTEMIKEADT NGDGVISFGE FASIMAKSAV DYFGLKINS
   ThCML15  ------GSLT ILELAALLRS LGL--KPS-G DQIHVLLASM DS-NGNG-FV EFEELVGAIL PDL------- -NEE---ILI NSQQLLDIFK SFDRDGNGFI SAAELAGAMA KMGQPLTYKE LTEMIKEADT NGDGVISFGE FASIMAKSAV DYFGLKINS
 CsatCML16  ------GSLT QLELGALLRS LGI--KPS-G DQLHSLLSNM DS-NGNG-SI EFDELVNAIL PDM------- -NDD---ILV NQEQLMEVFR SFDRDGNGYI TAAELAGSMA KMGHPLTYRE LSDMMRQADT DGDGVISFNE FTTVMAKSAA DFLGLTFA
   GrCML16  ------GSLT QLELAALLRS LGL--KPS-G DQIHLLLGDI DA-NGNG-LV EFEELESAIL PDI------- -THQ---ILI NEAQLLEVFQ LFDRDGNGFI TPAELAGSMA KMGHPLTFRE LSDMMKEADS NGDGVVSFLE FTSIMANSAA DFLGIKLNHS VK
 EgCML15-1  ------GSLT ILELAALLRS LGL--KPS-G DQVHVLLGNM DA-NDNG-LV EFDELASVIL PDM------- -NTD---TLI NQEQLLEVFR SFDRDGNGYI TAAELAGAMA KMGQPLTYKE LRDMIKEADA DGDGVISFNE FATVMAKSAS DIFGKSFS
 GmCML15-2  ------GSLT ILELAALLRS IGL--NPS-G DEIHALLANM DS-NGNG-FV EFDELVDAIL HDI------- -SAE---ILL KQEMLFGVFK CFDRDGNGYI TAAELAGAMA KMGQPVTYRE LTEMITEADT DGDGVISFNE FVTVMGRSAT HFLGLALL
 GmCML15-3  ------GSLT ILELAALLRS LGL--NPS-G DEIHALLANM DS-NGNG-FV EFDELVEAIL HDI------- -SAE---ILL NQEMLFGVFK CFDRDGNGYI TAAELAGAMA KMGQPLTYRE LTEMITEADT DGDGVISFNE FASVMGRSAS DFLGLALL
 PvulCML15  ------GSLT NMELAALLGS LGL--KPS-S EQIHVLLTNM DS-NGNG-FV EFDELVDAIL HNI------- -SAK---ILL NREMLLGVFK YFDRDGNGFI TAAELAGAMA KMGQPLTYRE LREMITEADT DGDGVISFNE FATVMGRSAS DFLDFALL
 GmCML15-1  ------GSLT MLELAALLRS LGL--KPS-G DQVQALLANM DS-NANG-KV EFDELIRAIL PDI------- -NAQ---VLL NQEQLLGVFK CFDRDGNGYI SAAELAGAMA KMGQPLTYRE LTEMIKEADT DGDGVISFTE FATIMARSAS DFLGLSFC
   MtCML15  ------GSLT MLELAALIRS LGV--RPS-G DEVQILLTKM DS-NGNG-SV EFDELVEAIM PNM------- -NAE---VLV NQEQLIGVFK CFDRDGNGFI SAAELAGAMA KMGQPLTYKE LIEMIREADM DGDGVISFSE FATIMARSAS DLLGVFEYRN KTHSFQS
 GrCML15-1  ------GSLT ILELAALLRS IGI--KPS-G DQIHVLLANM DA-NGNG-SV EFDELAS-IL PEL------- -TGE---ILN NQERLTEVFQ LFDRDGNGYI TAAELAGCMA KMGYPLTYSE LTEIIKEADS DGDGVISFTE FSSIMGKSAL EFLGISLSS
   FvCML16  ------GSLT HLELAALLRS LGV--K---- --LHVLLNNM DA-NGNG-AV EFEELVNAIM PNM------- -NDE---ILV NQEQLMEVFR SFDRDGNGYI TAAELAGSMA KMGHPLTYRE LSDMMREADT NGDGVISFNE FATIMSRSAA NFLGI
   LuCML15  ------RSLT VRELAGLLRS LGL--SAS-G NEIHDLLTNM DS-NGNG-FV EFEELVEAIS NQID------ -EQT----LV NQEQLLEIFQ LFDRDGNGVI TKAELAGGLA KMGQPLTWRE LTEMMIQADT NGDGVISFTE FSTVMARSTL ESLGIVTPV
   AcCML20  ------GTID AKELNVAMRA L-G--FEMTE EQITQMIADV DK-DGSG-AI DFDEFVHMMT AKIG------ -------ERD TKEELMKAFH IIDQDNNGKI SAVDIQRIAK DLGESFSDRD IQEMIEEADR DHDGEVNADE FIRMMRRTTY GY
 GrCML20-2  ------GTID AKELNVAMRA L-G--FEMTE EQINQMIADV DK-DGSG-AI DFDEFVHMMT AKIG------ -------ERD TKEELMKAFQ IIDQDNNGKI SADDVKRIAK ELGEHFSEKD IQDMIEEADR DHDGEVSIEE FMRMMKRTTY GY
   FvCML20  ------GTID AKELNVAMRA L-G--FEMTE EQINQMIADV DK-DGSG-AI DFDEFAHMMT AKIG------ -------ERD TKEELMKAFH ILDQDNNGKI STADIKKIAK ELGETFTDRE IQEMVEEADR DRDGEVSADE FIRMMKRTTY GY
 MdCML17-1  ------GTID AKELNVAMRA L-G--FEMTE EQIHQMIADV DK-DGSG-AI DFDEFAHMMT AKIG------ -------ERD TKEELMKAFQ LIDLDRNGKI SAADIKSIAK DLGENFTDSE IQEMIEEADR DRDGEVNADE FIRMMKRTAY GYX
   MdCML20  ------GTID AKELNVAMRA L-G--FEMTE EQINQMIADV DK-DGSG-AI DFDEFAHMMT AKIG------ -------ERD TKEELMKAFQ LIDLDRNGKI SAADIKNIAK DLGENFTDRE IQEMIEEADR DRDGEVNADE FIRMMKRTAY GY
 PperCML20  ------GTID AKELNVAMRA L-G--FEMTE EQITQMIADV DK-DGSG-AI DFDEFVHMMT AKIG------ -------ERD TKEELMKAFH LIDQDNNGKI SAADIKNIAK DLGENLSDRE IQEMIEEADR DRDGEVNAEE FIRMMKRTAY GY
   CcCML20  ------GTID AKELNVAMRA L-G--FEMTE EQINQMIADV DK-DGSG-AI DLDEFEHMMT AKIG------ -------ERD SKEELMKAFH IIDQDNNGKI SAGDIKRIAR ELGVNFTDRE IHEMVEEADR DHDGEVNADE FIRMMKRTTF GY
   CsCML20  ------GTID AKELNVAMRA L-G--FEMTE EQINQMIADV DK-DGSG-AI DLDEFEHMMT AKIG------ -------ERD SKEELMKAFH IIDQDNNGKI SAGDIKRIAR ELGVNFTDRE IHEMVEEADR DHDGEVNADE FIRMMKRTTF GY
   CpCML20  ------GTID AKELNVAMRA L-G--FEMTE EQINQMIADV DK-DGSG-AI DFDEFVHMMT AKIG------ -------ERN TNDELTKAFQ IIDQDKNGKI SADDIKCVAK ELGENFTDRE INDMIDEADR DNDGEVNIDE FMRMMKRTTF GY
   PtCML20  ------GTID AKELNVAMRA L-G--FEMNE EQIDQMIADV DK-DGSG-AI DFDEFVHMMT AKIG------ -------ERD TKEELSKAFR IIDHDKNGKI SVGDIKQIAK ELGESFTERE IQEMVEEADQ DRDGEVGVDD FMRIMRRTTY GY
 MeCML20-1  ------GTID AKELNVAMRA L-G--YEMTE EQINLMIADV DK-DGSG-AI DYDEFEHMMT AQIG------ -------ERD TKEELMKAFR IIDQDNNGKI SVDDIKRIAK ELGENFTDRD IREMIDEADR DHDGEVGMDE FFRTMKRTTY RY
   CrCML20  ------GTID AKELNVAMRA L-G--FEMTE EQINQMIADV DK-DGSG-AI DFDEFVHMMT AKIG------ -------ERD TKEELTKAFQ IIDLDKNGKI SPDDIKRMAK DLGENFTDAE IREMVEEADR DHDGEVNMDE FMRMMRRTTY GGN
   AtCML20  ------GTID AKELNVAMRA L-G--FEMTE EQINKMIADV DK-DGSG-AI DFDEFVHMMT AKIG------ -------ERD TKEELTKAFQ IIDLDKNGKI SPDDIKRMAK DLGENFTDAE IREMVEEADR DRDGEVNMDE FMRMMRRTAY GGN
   ThCML20  ------GTID AKELNVAMRA L-G--FEMTE EQINKMIADV DK-DGSG-AI DYDEFYYMMT AKIG------ -------ERD TKEELTKAFQ IIDLDKNGKI SADDIKRMAK DLGENFTDAE IREMVEEADR DRDGEVNMEE FMRMMKRTAA YEY
 CsatCML20  ------GTID AKELNVAMRA L-G--FEMTE EQIKQMIADV DK-DGSG-AI DYDEFEYMMT AKIG------ -------ERD TKEELTKAFD IIDYDKNGKI SGNDIKRIAK ELGEVFTDKD IQEMIDEADR DRDGEVNVDD FFRMMRRTTY GS
   SlCML20  ------GTID AKELNVAMRA L-G--FEATE EEINQMIAEV DK-DGSG-AI DFDEFVHMMT AKFG------ -------ERD TKEELMKAFH IIDQDKNGKI SFADIQRIAD ELGERFTDRE IQEMIEEADR DRDGEVNVED YMRMMRRTNF GH
 GrCML20-1  ------GTID AKELNVAMRA L-G--FEMTE EQIEQMIADV DK-DGSG-AI DFDEFVYMMT AKIG------ -------ERD TKEELTKAFQ IIDQDNNGKI SAQDIERIAN ELGVNLTKKE IQDMIEEADK DNDGEVSMDE FMRMMKRTTY GY
   EgCML20  ------GTID ARELNVAMRA L-G--FEMTE EQIGKMIADV DK-DGSG-SI DYDEFEHMMT AKIG------ -------ERD TKEELTKAFH LIDQDNNGKI SFEDIRRISK ELGEHFTDRD IQEMIGEADQ DRDGEVDMNE FIQMMGRTRL GF
   StCML20  ------GTID ASELNVAMRA L-G--FEMTE EQVEQMIADV DK-DGSG-AI DFDEFVHMMT AKIG------ -------ERD TKEDLAKAFN ILDQDKNGKI SAADIQQIAK ELGENFSAKE IQEMIEAADR DRDGEVSAEE FMRMMKKTSF AN
   GmCML20  ------GTID AKELNVAMRA L-G--FEMTE EQINQMIADV DK-DGSG-AI DYEEFEYMMT AKIG------ -------ERD TKEELMKAFH IIDHDKNGKI SALDIKRIAK ELGQNFTDRE IQEMVEEADQ DNDREVSAEE FITMMNRTRF HH
 PvulCML20  ------GTID AKELNVAMRA L-G--FEMTE EQINQMIADV DK-DGSG-AI DYDEFEYMMT AKIG------ -------ERD TKEELMKAFH IIDHDKNGKI SASDIKRIAK ELGQNFTDRE IQEMVDEADQ DNDREVSPEE FITMMTRTRF HH
   MtCML20  ------GTID AKELNVAMRA L-G--FEMTE EQIEQMIADV DK-DGSG-AI DYDEFEHMMT AKIG------ -------ERD TKEELMKAFH IIDKDKNGKI SASDIKRIAK ELGQNFTDRE IQEMVDEADQ NNDREVDPEE FIMMMNTTGY RH
 VvCML20-1  ------GTID ARELNVAMRA L-G--FEMTE EQITQMIADV DK-NGSG-AI DFDEFAHMMA AKIG------ -------ERD SKEELMKAFH IIDQDQNGKI SSMDIKRITE ELGEKFSTRE IEEMIREADQ DSDGEVSAEE FMRMMKRTTY GY
 VvCML20-2  ------GTID AKELSLAMRA L-G--FEMTD AQIEQMIKDV DK-DGSG-AI DFDEFVYMMT TKIG------ -------ERD TKEELMKAFH IIDLDKNGKI SATDIQRVAK ELGESFTANE IEEMIREADQ DSDGEVNLEE FMKMMKRTTY GY
    BdCML8  ------GTID AKELNVAMRA L-G--FEMTE EQINQMIADV DK-DGSG-SI DYEEFEHMMT AKIG------ -------ERD TKEELTKAFR IIDQDKNGKI SDVDIQRIAK ELGENFTLQE IQEMVHEADQ NGDGEIDFGE FARMMKRTSY GY
    SbCML8  ------GTID AKELNVAMRA L-G--FEMTE EQIRQMIADV DK-DGSG-AI DYEEFEHMMT AKIG------ -------ERD SKEELTKAFR IIDQDGNGKI SNIDIQRIAK ELGVNLTLDE IQDMVQEADR NGDGEIDFDE FIRMMRRTSY GY
  ZmCML8-2  ------GTID AKELNVAMRA L-G--FEMTE EQIRQMIADV DK-DGSG-AI DYEEFEHMMT AKIG------ -------ERD SKEELSKAFR IIDQDGNGKI SNIDIQRIAK ELGVNLTLDE IQDMVQEADR NGDGEIDFDE FIRMMRRTSF GY
  PhCML8-1  ------GTID AKELNVAMRA L-G--FEMTG EQIDQMIADV DK-DGSG-AI DYEEFEHMMT AKIG------ -------ERD SKEELTKAFR IIDQDRNGKI SNIDIQRIAK ELGVNLTLEE IQDMVQEADR NGDGEIDFDE FTRMMRRTSY GVRRR
  PvCML8-2  ------GTID AKELNVAMRA L-G--FEMTE EQINQMIADV DK-DGSG-AI DYEEFEHMMT AKIG------ -------ERD SKEELTKAFR IIDQDRNGKI SNIDIQRIAK ELGVNLTLEE IQDMVQEADR NGDGEIDFDE FTRMMRRTSY GVRGR
   ThCML19  ------GSID PRELNVAMRS L-G--FEMNN EQINELMAEV DK-NQSG-AI DFEEFVHMMT TKFG------ -------ERD STAELTKAFK IIDQDNNGKI SQRDIKLIAK ELGENFTDND IEEMIEEADR DKDGEVNLEE FMKMMMRTSY AYQK
   AtCML19  ------GSID ASELNVAMRS L-G--FEMNN QQINELMAEV DK-NQSG-AI DFDEFVHMMT TKFG------ -------ERD SIDELSKAFK IIDHDNNGKI SPRDIKMIAK ELGENFTDND IEEMIEEADR DKDGEVNLEE FMKMMKRTSY G
 MeCML20-2  ------GTID AKELNVAMRA L-G--FEMTE EQINQMIADV DK-DGSG-AI DFDEFVHMMT AKIG------ -------ERD TKEELMKAFR IIDQDNNGKI SVDDIKRIAK ELGENFTDRE IQDMVDEADR D
 MpCML20-2  ------GTID AKELKVAMRA L-G--FEPKK EEIKKMIADI DK-DGSG-TI DFEEFLQMMT AKMG------ -------ERD SREEIMKAFR LFDDDETGKI SFKNLKRVAK ELGENMTDEE LQEMIDEADR DGDGEVNEEE FFRIMKKTSL F
   SmCML20  ------GTID AKELKVAMRA L-G--FEPKK EEIKKMIADV DK-DGSG-TI DFEEFLQMMT AKMG------ -------ERD SKEEIMKAFR LFDDDETGKI SFKNLKRVAK ELGENMTDEE LQEMIDEADR DGDGEISEEE FYRILKKTSL F
   OlCML20  ------GTID AKELKVAMRA L-G--FEPNK DEIKKMIADI DK-DGNG-TI DFEEFLAMMT AKMG------ -------ERD SREEIMKAFR LFDDDETGKI SFKNLKRVAK ELGENMTDEE LQEMIDEADR DGDGEVNEEE FFRIMKKTSL F
 VcCML20-1  ------GTID AKELKVAMRA L-G--FEPKK EEIKKMISDI DK-DGSG-TI DFEEFLNLMT AKMG------ -------ERD SREEILKAFK LFDDDGSGTI TFKDLKRVAK ELGENLTDEE LQEMIDEADR DGDGEVNEEE FIRIMKKTAL F
 VcCML20-2  ------GTID AKELKVAMRA L-G--FEPKK EEIKKMISDI DK-DGSG-TI DFEEFLQMMT AKMG------ -------ERD SREEILKAFR LFDDDGSGTI TLKDLRRVAK ELGENLTDEE LQEMIDEADR DGDGEINEDE FIRIMKKTSL F
  PhCML8-2  ------GTID ARELNVAMRA L-G--FEMTP EQINQMIAEV DK-DGSG-TI DFDEFVHMMT DKMG------ -------ERD ARDELYKAFR IIDKDGNGKI SDIDIQRLAI ETGEHFTLDE VREMIEAADE NGDGEIDLEE FMKMMRWTNL GSGF
  PvCML8-1  ------GTID ARELNVAMRA L-G--FEMTP EQINQMIAEV DK-DGSG-TI DFDEFVHMMT DKMG------ -------ERD ARDELYKAFR IIDKDGNGKI SDIDIQRLAI ETGEHFTLDE VREMIEAADE NGDGEIDLEE FMKMMRRTNL GSGF
  PvCML8-3  ------GTID ARELNVAMRA L-G--FEMTP EQINQMIAEV DK-DGSG-TI DFDEFVHMMT DKMG------ -------ERD ARDELYKAFR IIDKDGNGKI SDIDIQRLAI ETGEHFTLDE VREMIEAADE NGDGEIDLEE FMKMMRRTNL GSGF
  ZmCML8-1  ------GTID ARELNVAMRA L-G--FEMTP EQIGQMIAEV DK-DGSG-TI DFDEFVHMMT DKMG------ -------ERD ARDELHKAFR IIDQDANGKI SDMDIQRLAI ETGEHFTLDE VREMIEAADE DGDGEIDLEE FMKMMKRTDF GSGF
    OsCML8  ------GTID PKELNVAMRA L-G--FEMTP EQIHQMIAEV DK-DGSG-TI DFDEFVEMMT KKMG------ -------ERD AREELNKAFK IIDKDNNGKI SDVDIQRLAI ETGEPFTLDE VREMIEAADE NGDGEVDHEE FLKMMKRIGF GAGFF
   SmCML19  ------GTID AKELRVAMKA L-G--FETSA QEIRDMMASV DV-DQSG-TL DFEEFVEMMT KKMG------ -------ERE AKQELVRVFA ILDEQGRGML SFRDLRRLAV ELELPFSDKE VEDMIRVADS DGDGEVTEAD FVRIMSAAAS KSV
 MpCML20-1  ------GTID MRELKVAMRA L-G--FAVSS EDVKAIMTEY DA-DGSG-EI EYPEFRNVMR DKMA------ -------SRN PEDELAKAFK VFDDDSSGKI TLKNLRRIAK ELGEDVDDDE LRAMIDEFDA RGAGYIDERT FLDIMHKGAD AE
   AcCML11  ------GCIT IDELATVIRS L-D--QNPTE EELLDMINEV DV-DGNG-TI EFEEFLNLMA KKMK------ -------ETD ADEELKEAFK VFDKDQNGYI SATELRHVMI NLGEKLTDEE VEQMIKEADL DGDGQVNYEE FVRMMMAV
 GmCML11-3  ------GCIT VEELATVIRS L-D--QNPTE EELQDMISEV DA-DGNG-TI EFDEFLSLMA KKVK------ -------DTD AEEELKEAFK VFDKDQNGYI SASELRHVMI NLGEKLTDEE VEQMIKEADL DGDGQVNYDE FVKMMMTVR
 GmCML11-4  ------GCIT VEELATVIRS L-D--QNPTE EELQDMISEV DA-DGNG-TI EFDEFLSLMA KKVK------ -------DTD AEEELKEAFK VFDKDQNGYI SASELRHVMI NLGEKLTDEE VEQMIKEADL DGDGQVNYEE FVKMMMTVR
 MeCML11-2  ------GCIT VDELATVIKS L-D--QNPTE EELQDMINEV DA-DGNG-TI EFAEFLNLMA KKIK------ -------ETD AEEELKEAFK VFDKDQNGYI SATELRHVMI NLGEKLTDEE VEQMIKEADL DGDGQVNYDE FVKMMSTVG
 MeCML11-1  ------GCIT AEELATVIRS L-D--QNPTE EELQDMISEV DA-DGNG-TI EFAEFLNLMA KKMK------ -------ETD AEEELKEAFK VFDKDQNGYI SANELRHVMI NLGEKLTDEE VEQMIKEADL DGDGQVNYDE FVKMMMTVG
PperCML11-  ------GCIT VDELATVIRS L-D--QNPTE EELQDMISEV DV-DGNG-TI EFAEFLSLMA NKMK------ -------ETD AEEELKEAFK VFDKDQNGYI SATELRHVMI NLGEKLTDEE VEQMIKEADL DGDGQVNYDE FVKMMMTIG
 GrCML11-3  ------GCIT VEELATVIKS L-D--QNPSE EELQDMINEV DA-DGNG-TI EFSEFLNLMA KKMQ------ -------ETD AEEELKEAFR VFDKDLNGYI SASELRNVMM NLGEKLSDEE VEQMIKEADL DGDGQVNYEE FVKMMTTVG
 BrCML11-2  ------GCIT ADELATVIRS L-D--QNPTE QELQDMINEI DS-DGNG-TI EFSEFLNLMG SKIQ------ -------ETD ADEELKEAFK VFDKDQNGYI SASELRHVMI NLGEKLTDEE VDQMIKEADL DGDSQVNYDE FVRMMMTSG
 BrCML11-3  ------GCIT ADELATVIRS L-D--QNPTE QELQDMINEI DS-DGNG-TI EFSEFLNLMA NKIQ------ -------ETD ADEELKEAFK VFDKDQNGYI SASELRHVMI NLGEKLTDEE VAQMIKEADL DGDGQVNYDE FVRMMMTSG
   CrCML11  ------GCIT ADELATVIRS L-D--QNPTE QELQDMITEI DS-DGNG-TI EFSEFLNLMA NQIQ------ -------ETD ADEELKEAFK VFDKDQNGYI SASELRHVMI NLGEKLTDEE VNQMIKEADL DGDGQVNYDE FVRMMMTNG
   AtCML11  ------GCIT ADELATVIRS L-D--QNPTE QELQDMITEI DS-DGNG-TI EFSEFLNLMA NQLQ------ -------ETD ADEELKEAFK VFDKDQNGYI SASELRHVMI NLGEKLTDEE VDQMIKEADL DGDGQVNYDE FVRMMMING
   ThCML11  ------GCIT ADELATVIRS L-D--QNPTE QELQDMINEI DS-DGNG-TI EFSEFLNLMA NKIQ------ -------ETD VDEELKEAFK VFDKDQNGYI SASELRHVMI NLGEKLTDEE VDQMIKEADL DGDGQVNYDE FVRMMMNSG
 BrCML11-1  ------GCIT ADELATVIRS L-D--QNPTE QELQDMINEI DS-DGNG-TI EFSEFLNLMA IKIQ------ -------ETD ADEELKEAFK VFDKDQNGYI SASELRHVMI SLGEKLTDEE VDQMIKEADL DGDGQVNYDE FVRMMMASG
   FvCML11  ------GCIT IEELATAIKS V-D--QNPTA EELQSMINEV DI-DGNG-TI EFGEFLNVMV RKMK------ -------END ADEELKEAFK VFDKDQDGYI SPNELRNVMI NLGERLTDEE AEQMIREADL DGDGLVNYEE FVTMMLLAF
PperCML11-  ------GCIT IEELGTALKS L-D--QHPTE EELQNMISEI DA-DGNG-TI EFGEFLHLMA RKIQ------ -------ENE VDAELREAFK VFDKDQDGYI SPNELRHVMI NLGERLTDEE VEQMIREADL DGDGLVNYDE FVRMMLAF
 GrCML11-2  ------GCIT MEELAIAIKS L-D--QNPTQ EELQNMINEV DS-NGNG-TI EFGEFLNLMA TKMK------ -------EAE AEDELKEAFR VFDKDQDGYI SPYELRLVMM NIGEKLTDEE LEQMIREADL DGDGRVNYEE FVRMMLAAAS
 GmCML11-1  ------GCIT IEELSTAIRS L-D--ENPTV EELQIMMNEV DM-DGNG-TI EFGEFLNLMA RKMK------ -------ETE AEEELKEAFR VFDKDHDGYI SPSELRSVMR TIGEKVTDEE VEQMVKEADL DGDGLIDYEE FVRMMLAD
 GmCML11-2  ------GCIT IEELSTAIRS L-D--ENPTV EELQIMMNEV DM-DGNG-TI EFGEFLNLMA RKMK------ -------ETE AEEELKEAFR VFDKDHDGYI SPSELRSVMR TIGEKVTDEE VEQMVKEADL DGDGLVDYEE FVRMMLAV
 PvulCML11  ------GCIN IEELGTAIRS L-D--ENPTL EELQIMMSEV DT-DCNG-TI EFGEFLNLMA RKMK------ -------ESE AEEELKEAFR VFDKDQDGYI SASELRSVMR TIGEKVTEEE VAQMVKEADL DGDGLVDYEE FVRMMLAA
 PtCML11-1  ------GRIT FEELATVIKS L-D--HGATE EELRHMIREV DV-DGNG-TI EFGEFWNLMA RKIK------ -------END ADDELKEAFK VFDKDQDGYI SPNELRHVMI NLGEQVTDKE LELMIQVADL DGDGQVNYEE FMRMMLAI
    BdCML4  ------GCIS LEELAAVTRS L-G--LEPTE QELSDMMREV DT-DGNG-TI DFQEFLSLIA RKMK------ -------DGD GDEELKEAFE VLDKDQNGFI SPVELRTVMI NLGEKMTDEE VEQMIREADT DGDGLVNYDE FVLMMKNAER KISG
    OsCML4  ------GCIT LEELAAVTRS L-G--LEPTD QELNDMMREV DT-DGNG-II DFQEFLSLIA RKMK------ -------DGD GDEELKEAFE VLDKDQNGFI SPTELRTVMT NLGEKMTDEE VEQMIREADT DGDGQVNYDE FVIMMKNAER KISG
    PhCML4  ------GCIT MEELAAVTRS L-G--LDPSD QELNDMMSEV DT-DGNG-II DFQEFQSLIA RKMK------ -------DGD GDEELKEAFE VLDKDQNGFI SPNELRTVMI NLGEKMTDQE VEQMIREADT DGDGLVNYDE FVLMMKNAER KISG
    PvCML4  ------GCIT MEELATVTRS L-G--LDPSD QELNDMMSEV DT-DGNG-II DFQEFQSLIA RKMK------ -------DGD GDEELKEAFE VLDKDQNGFI SPNELRIVMT NLGEKMTDQE VEQMIREADT DGDGLVNYDE FVLMMKNAER KISG
    ZmCML4  ------GCIT MEELAAVTRS L-G--LDPSD QELNDMMSEV DT-DGNG-II DFQEFLSLIA RKMK------ -------DGD GDEELKEAFE VLDKDQNGFI SPVELRTVMT SLGEKMTDEE VEQMIREADT DGDGQVNYDE FVLMMKNAER KISG
    PhCML5  ------GCIT VEELATVMGS LQG--QRPGA EELREMIRDA DA-DGDG-AI DFAEFLGLMA RKAAAG---- GAGAGDAAAD PDEELREAFK VFDKDQNGYI SAAELRHVMI NLGEKLTDEE VEQMIREADL DGDGQVNYDE FVRMMMLSDG HHQ
  PvCML5-2  ------GCIT AEELATVMGS LQG--QRPGA EELREMIRDA DA-DGDG-AI DFAEFLGLMA RKTAAG---- AGGDGDGAAD PDEELREAFK VFDKDQNGYI SATELRHVMI NLGEKLTDEE VEQMIREADL DGDGQVDYDE FVRMMMLSDG HHHQ
  PvCML5-1  ------GCIT LEELATVMGS LQG--QRPGA EELRAMIRDA DA-DGNG-TI DFAEFLGLMA RKTTGA---- GAAGGDGA-- -DEELREAFK VFDKDQNGYI SATELRHVMI NLGEKLTDEE VEQMIREADL DGDGQVDYDE FVRMMMLSDG HHQ
    SiCML5  ------GCIT VEELATVMGS LQG--TRPSA EELREMIRDA DA-DGNG-TI DFAEFLGLMA RKTA------ GAGGDGDGAD PDEELREAFK VFDKDQNGYI SATELRHVMI NLGEKLTDEE VEQMIREADL DGDGQVNYDE FVRMMMLSDG HGGGHAGAAA VTQHQ
    ZmCML5  ------GCIT VEELATVMGS LQG--QRPSA EELREMIRDA DA-DGNG-AI DFAEFLGLMA RKTA------ G-GGAGGGAD PDEELREAFK VFDKDQNGYI SATELRHVMI NLGEKLTDEE VEQMIREADL DGDGQVNYDE FVRMMMLSDG AG---AGAAA TGHHQ
    SbCML5  ------GCIT VEELATVMGS LQG--HRPSE DELGEMIRDA DA-DGNG-TI DFPEFLALMA RKTAASSAAS GAGGGDGDYD PDEELREAFK VFDKDQNGYI SATELRHVMI NLGEKLTDEE VEQMIREADL DGDGQVNYDE FVRMMMLSDG AG--HAGAGA -GHHQ
    OsCML5  ------GCIT LEELDTVVRS L-G--QTPTR EELAEMIRDV DV-DGNG-TI EFAEFLALMA RKASRGGE-N GGGGDDSGDA ADEELREAFK VFDKDQDGLI SAAELRHVMI SLGEKLTDEE VEQMISEADL DGDGQVNFDE FVRMMMLSDQ
   EgCML11  ------ACMT LEEFAAIIQS L-D--HSPSK KELQDMIDKA AAADGNG-VI EFAKFLGLMA YNIK------ ------EAAD EEEELKEAFK VFDKDQDGYI SPSELRHVMI NLGEKLTEEE VQQMISEADL DGDGQVGFEE FVKLMSTIG
    BdCML1  ------GSIT TKELGTVMRS L-G--QNPTE AELQDMINEV DA-DGNG-TI DFSEFLNLMA RKMK------ -------DTD SEEELKEAFR VFDKDQNGFI SASELRQVMT NLGEKLSEEE VEEMVREADV DGDGQINYDE FVKVMMAKRR NKRIQERKA- -SGKKSTAVP GPSDG-KRGQ
    BdCML3  ------GTIT TAELGTVMKS L-G--QHPTE AELRDMVEEV DA-DGSG-AI DFEEFLSLVA RQMR------ ----GEGDAD AEEELREAFR VFDKDSSGAI SLDELRSVMK NLGEKLSEDE LNEMLHEADV DGDGQINYKE FAKVMMAKRR QNMEEERGG- -DHHGSDHSH G-GGC----P
    OsCML2  ------GTIT SKELGTVMGS L-G--QSPTE AELKKMVEEV DA-DGSG-SI EFEEFLGLLA RKLR------ -------DTG AEDDIREAFR VFDKDQNGFI TPDELRHVMA NLGDPLSDDE LADMLHEADS DGDGQINYNE FLKVMMAKRR QNMMEGHGS- -GGHRSSNSH KKSGCCGPNS
    OsCML3  ------GTIT SKELGTVMGS L-G--QSPTE AELKKMVEEV DA-DGSG-SI EFEEFLGLLA RKLR------ -------DTG AEDDIRDAFR VFDKDQNGFI TPDELRHVMA NLSDPLSDDE LADMLHEADS DGDGQINYNE FLKVMMAKRR QNMMEGHGS- -GGHRSSNSH KKSGCCGPNS
 CsatCML50  ------GCIT TKELGTVMRS L-G--QNPTE AELQDMINEV DA-DGNG-TI DFPEFLNLMA RKMK------ -------DTD SEEELKEAFR VFDKDQNGFI SAAELRHVMT NLGEKLTDEE VDEMIREADV DGDGQINYEE FVKVMMAKRM EAMREERKKR KSLKQSIDKK KRGFLNRRIH
    SiCML2  ------GTIT SKELGTVMRS L-G--QSPSE EELQEMVKEV DA-DGSG-AI DFQEFLTLLA RQMR------ ----EANGAD -EEELREAFR VFDQDQNGFI SRDELRHVLQ NLGERLSDDE LAEMLREADV DGDGQINYTE FAKVMLA
    ZmCML2  ------GTIT TKELGTVMRS L-G--QSPTE EELQEMVDEV DA-DGSG-AI DLQEFLTLLA RQMR------ ----EASGAD -EDELREAFH VFDQDQNGFI SRDELRHVLQ NLGERLSEEE LAEMLREADA DGDGQINYSE FAKTKEPGAG RAWE
 GrCML11-1  ------GFIT MGELAAVIRT L-D--VNPTK D-VQGMFSEA NV-EGNGKTI DFKDFLNIMA SKMK------ --------EN LMDELQEAFK VFDRDEDGFI SANELRQVMM NLGERLTMEE AEQMIREADV DGDGVVSYEE FARMMMMAF
 PtCML11-2  ------GFIT LEELATIVQS L-D--RRPTI EEIRDMICEV YI-DGNG-TL DFEEFLNVMG RKQK------ --------EN VTEELKEAFK VFDRNQDGYI SSSELRQVMM NLGERLTEEE AEQMIREADL DGDGLVSYEE FSRMMAMAF
 PtCML11-3  ------GFIT MEELATIVQS L-D--RRPTK EEIQDMICDV DL-DGNG-TL DFEEFLNIMG RKQK------ --------EN VTEELKEAFK VFDRNQDGYI SANELRQVMI NLGERLTEEE AEQMIREADV DGDGLVSYEE FARMMMVAF
PperCML11-  ------GLIS LEELAAVIQS L-D--EHPTK EEIQDMINEV GA-EGNG-TI DCEEFLNIMA RKMK------ --------EN VAEELKEAFK VFDRDQDGYI SANELRQVMI NLGEKLSDEE AEQMIREADV DGDGLVSYEE FARMMMLS
    BdCML5  ------GRIT ADELSTVIRT SLG--QSPTP SELRDMVSEV DA-DGDG-TI EFAEFLALMA RNRC------ ------KDGD GEEELREAFG VFDRNQDGLI SREELRHVMV SLGEKMSEEE VDGMIFEADV DGDGFVDFRE FVRMMMLADD GQK
    OsCML6  ------GCIS REELATVLTR L-G--MAPSQ EDLQDMIVAV DE-DGNG-TI EFDEFLAIMK KKLY------ ----ENDKGD DEEELRKAFR IFDKDDNGFI SRNELSMVMA SLGEEMTEDE IDDMMKAADS NNDGQVDYEE FKRVMMSTWN ITEIFKPHVT IWRYKP
   MdCML11  IMSSVLGCIT VDELXTVIRS L-D--QNPTE XELQDMISXV DV-DGNG-TI EFAEFLSLMA NKMK------ -------ETD AEEELKEAFK VFDKDQNGYI SATEATKFRI SDGFIAGIAS MDSDKDATVL NTAWNVETFV SISVCPKPTL QLIFFDYVLT LKRLSVLLRD EHNHID
   AtCML13  ------GKIA PSELGILMRS L-G--GNPTQ AQLKSIIAS- ---ENLSSPF DFNRFLDLMA KHLK------ -------TEP FDRQLRDAFK VLDKEGTGFV AVADLRHILT SIGEKLEPNE FDEWIKEVDV GSDGKIRYED FIARMVAK
   AtCML14  ------GKIA PSELGILMRS L-G--GNPTE SQLKSIITT- ---ENLSSPF DFNRFLDLMA KHLK------ -------TEP FDRQLRDAFK VLDKEGTGFV AVADLRHILT SIGEKLQPSE FDEWIKEVDV GSDGKIRYED FIARMVAK
 BrCML34-2  ------GKLS LDEFREAVLA FS---LNISE KEVTQMFKDI DV-DGDG-EL SSDEFASSV- ---------- ---------- -DKMLKEAFA LCDADGDGKI TSSEFHAAMI GLGKECTEEK CAEMVKTVDA DGNGYLSFEE FMVMIIGEF
 BrCML34-1  ------GKLS LEEFREAALA FS---KNIPD EEITNMFKEF DV-DGDG-EL DADEFTLCI- ---------- ---------- -NNMLKEAFD FCDTDGAGKV TASEFHAAMT GLGEDFTEEK CAEMVQAVDA DGDGYVSFEE FMAMIIGEFK K
   CrCML34  ------GKLS LDEFREAALA FS---PNFSQ EDIAKFFKEI DV-DGNG-EL NADEFTSCI- ---------- ---------- -EKMLKEVFD FCDVDGDGKI PASESYVTMT SIGMTCTEES CAEKVRAVDA DGDGYLNFEE FMALVIGDI
   AtCML34  ------GKLS LDEFREVALA FS---PYFTQ EDIVKFFEEI DV-DGNG-EL NADEFTSCI- ---------- ---------- -EKMLKEVFV FCDVDGDGKI PASESYVTMT SLGKKFTEET SAEKVRAADV DGDGYLNFDE FMALVIGDI
   ThCML34  ------GKLS LDEFHEAALA FF---NNFSQ EDISSMFKEM DV-DGDG-EL DADEFTSCI- ---------- ---------- -EKMLKDVFD FCDVDGDGKI PADELYVSMT KLGKECTEES CAEKVRLADA DSDGYLSFEE FTAMVLSDF
 BrCML32-2  ------GKIS WDEFSQWIRA FS---PLMTS EEIDEMFTEL DA-DGDR-QI DIVEFAKCYV V--------- ----GEKEEE EEAVLKEAFD LYDLNRDGKI SPGELHVVLK RLGRNKSMEQ CASMVGAVDA DQDGHVSFEE FKTMMNSDNK TLL
   CrCML32  ------GKIS WNEFAEEIRV FS---RSITS EELDKMFRVL DV-DGDV-QI DVVEFASCLV IDGG------ ----GEQN-D EETVLKGAFD LYDMDGDGKI SASEIHVMLK RLGEKHTMEE CVVMVQAVDA DEDGFVNFEK IKTMMSSNHK KSL
 CrCML32-2  ------EKIS WNEFAEAIRV FS---PSITS EELDKMFRVL DV-DGDG-QI DAVKFASCLV IDGR------ ----GEQD-D EETVLKEAFD LYDMDGDGKI SASEIHVVLK RLGEKHTMEE CVVMVQAVDA DGDGFVNFEE FKTMMSSNHK KSL
 CrCML32-1  ------GKIS WDEFAEAIRV FS---PKITS EEIDKMFREL DI-DGDN-EI DAVEFASCLM LNN------- ----GEKE-D EEVVMKEAFD LYDIDRDGKI SASEIHVVLK RLGEKHTMDE CVMMVRAVDK DGDGFVDFEE FKTMMTSTKK SL
   AtCML32  ------GKIS WDEFAEAIRA FS---PSITS EEIDNMFREI DV-DGDN-QI DVAEYASCLM LGGE------ ----GNKE-D EDIVMKEAFD LYDIDGDGKI SASEIHVVLK RLGEKQTIAE CIAMVRAVDA DGDGFVSFEE FKTMMSCNNK KLQ
   AtCML31  ------GKIL WDEFAEAIRV FS---PQITS EEIDKMFIVL DV-DGDG-QI DDVEFASCLM VNGG------ ----GEKDTE EEVVMKEAFD LYDMDGDGKI SASEIHVVLK RLGEKHTMED CVVMVQTVDK DSDGFVNFEE FKIMMNSNKE SH
   ThCML32  ------GKIS WDEFAEGIRV FS---PSTTS EEIDKMFREL DT-DGDG-HI DVNEFAKCMI SG-------- ------KEDD EEAVMKTTFD LYDIDGDGKI SANEIHVVLK RLGENISMEE CVKMVRAVDA DGDGFVSFEE FKTMMNSNNK KAL
   ThCML33  ------GKIS WEEFRDAMNA LS---PTIPS EKLVEMFIQL DT-NGDG-QV DATEFASCMD QTAR------ ----SSGC-G VEKELKDAFR LYDIDSDGKI SANELYEVMT RLGEKCSLEI CVEMVRAVDV DGDGYISFEK FKSMMGF
   AtCML33  ------GKIS WEEFRDAIHA LS---PSIPS EKLVEMFIQL DT-NGDG-QV DAAKFASCMD QTAQ------ ----SSGG-D VEKELKDAFK LYDINCDGKI SANELHVVMT RLGEKCTVES CVGMVQAIDV DGDGYIRFVG V
 EgCML25-2  ------GKIS PTELSRVLSG LGHVGAAAAD EEARVMVKEI DR-DGDG-RV DLDEFLVAVL SGD------- ---------- --DDLMDAFL IFDANKDGLI SAKELQRVLQ GVRCEASIED CRRMIRGVDT DGDGFVNFEE FRSMMRGCFS
   SmCML25  ------GRIC LEELRAMLRL IGN--ANPDD TELLGLLRAI DS-DGDG-FI SLEEFLRAND EGG------- ---------S SADDLRAAFQ VFDIDGNGFI SADELHCVLQ KMGDKITKSE CRRMIKGVDS DGNGLVDFEE FRIMMA
 LuCML25-1  ------GFID FKEFVEL--N TQGVGTEEAM VNLRDAFSVY DI-DGDG-SI TAEELHKGVG TEE------- ---------A MVN-LRDAFS VYDIDGDGSI TAEELHKVMA SLGEDCSIGE CRKMISGVDN DGDGTIDFEE FKMMMLRL
 AcCML25-3                 MTT LG---HSVTQ EQLDKIFQEV DS-NKDG-CI DFNEFLELNT IGVD------ -------SAK ISDDLKGAFM MYDSDRNGFI SPQELLLVMR SLGDKCSITE CIDIIRAVDS DGDGLIDYED FKVMMMASKQ
    BdCML2  ------RRYR SGPMDWMSGP I-LKVMSGPF EKVLTRPTEK ERSGPLE-EE KEKEKSGPL- ---------- --EEKSAGGD HRRRCGTSCT IL
   CrCML12  ------GCIT AKEFVTMLRS L-GEDNTEAE VQDMINEVDL DGNGTID-FF EFLSIMARS- ---------- ---VVNQGQ- VTRKRDRTVS ILKTIGNSLS TFSIVVDIIQ LLF
   AtCML12  ------GYIT VNELRTTMRS L-GETQTKAE LQDMINEADA DGDGTIS-FS EFVCVMTGKM IDTQS--KKE TYRVVNQGQG QVQRHTRNDR AGGTNWERDI AVGVASNIIA SPISDFMKDR FKDLFEALLS
 CsubCML24  ------LRQN VHKLAVALDF F-YSTTDRIG RADFQRAVAK ILGDTLA-ED LVDVIFAIY- ----D--PKE QDNLAYKELL YALEKREGNM IYAKQMLDLN SDTEVQSVFS RLRSSFMPQ
    PpCML7  ------DKEE WHTLVQQHPS L-LKNMTLPY LRDITTTFPS FIFHSRV-ED N
 MpCML21-1  ------LMEK IEQLMDGKGS M-IKVFREL- --DWDRSGTV DPREFRA-WL NRLNFH-PD- -DETF--E-- --RTWKSFDP --EGKGH--- --LVYQDFVS RVVPKTKFGG LE
    MpCML4  ------LHPD VKEGKITPEQ C-VRDFGKQW -DTLERDGVI TFDEFLE-YY RDISAAIPM- -DDYF--ELM ICNAWRMAPP GADSSAHATR VLVTFENGRS EMVEIEDDAG LDTHWFPYDR VRVVNADP
   MpCML30  ------GYIS HHEALSMLRS L-VPSLTTSN LRQLVHHLRA ADAENVG-RV TLPE
```

```
   MpCML15  ------GYLE YPELWRMVGD L-VPSATTLE RRSLLAGLWS ADVDGDG-RV SYDELLR
   MtCML46  ------GFID AKELQRVLVI L--GLKQGSE FENCQKMITI FDENQDG-RI DFIEFVNIM- -KNHF--CYL RVAKSSCYFL GFKQGSEFEK CQKIIKNFDE NQDERIESLN LLILLFKGNH FC
   AtCML45  ------GFID AIELQRVLTI L--GFKQGSY LDNCLVMIRS LDGNKDG-KI DFNEFVKFM- -ETSF--Y
   AtCML46  ------GFID PIDLQRVLTI L--GLKQGSN LENCRRMIRS FDGSKDG-RI DFYGFVKFM- -ENNF--C
   AtCML47  ------GFID ENELKHVLSL L--GYDECTK ME-CRKMVKV YDENRDG-KI DFYEFVKLI- -EKSF--S
    VcCML4  ------GTLA TDELLA-SIA LDGAVGDDAI DADVVKVF-- -DQDGDG-FV SLREFKSGV- -PALG--PNG EAAK------ ---EYIFSRV DQMVDGDNRL DTQEFANALT LMRTAVLGY
  OsCML1-3  ------GRIT AEELRGVMVA ILGGDGDGCS LDDCRRMIGG VDADGDG-FV GFQDFARMM- -MAAT--ATA TATA------ ---DGPRSW
   AtCML44  ------GYIS AEELRDVLER L--GFEEEAK AWDCGRMIRV HDKNLDG-FV DFEEFKNMI- -LHV
   OlCML11  ------GYID SSELRKIICA T-NMSTDKQI ERKVEWLMRQ CDTDGDG-NI SFEEF
    AtCML1  ------AITR LKSAKHFFGA V-SSLKEGKA LECCKEMIKQ VDEDGHG-RV DYKEFLQMM- -KTGD--FSN R
 CsubCML20  ------IYAA ANVV
   SiCML36  ------VYCT ANCRI
   SbCML50  ------VYCT ANCRI
   MtCML11  ------VPAN
 Consensus        .... .......... ...  ..... .......... .. .... .. .......... ....        ...  .... .......... .......... .......... .......... .......... .......... .......... ......

            721
  AcCML3-1
    CcCML7
    CsCML7
  GrCML5-1
  GrCML5-3
  TcCML5-1
  MeCML3-1
    MeCML5
  PtCML3-3
  PtCML3-4
    RcCML3
    LuCML5
  LuCML3-3
  LuCML3-2
  GmCML5-1
  GmCML5-2
PvulCML3-1
    MtCML7
  CpCML3-1
  CsatCML5
    SlCML5
    StCML5
    FvCML5
  MdCML3-1
  MdCML3-4
PperCML5-2
  EgCML5-1
  EgCML5-4
    MgCML4
 LuCML30-3
  StCML3-1
   BdCML30
   OsCML30
   PhCML30
   SbCML30
   ZmCML30
   SiCML30
  AcCML3-2
    CpCML5
  MgCML3-2
  PtCML3-1
  PtCML3-2
    PtCML5
PperCML5-1
  StCML3-3
  GrCML3-2
  TcCML5-2
    CcCML3
  CsCML3-1
  EgCML5-3
  GmCML5-3
PvulCML3-2
    MtCML5
    FvCML7
  BrCML4-1
    CrCML4
  BrCML4-2
    ThCML4
    AtCML4
  CrCML5-1
    AtCML5
  ThCML5-1
  BrCML5-1
  BrCML5-2
  BrCML3-1
  BrCML3-2
    CrCML3
    ThCML3
    AtCML3
  CpCML3-2
  PtCML3-5
    EgCML3
  GrCML3-1
  CsatCML3
  LuCML3-1
    FvCML3
  MdCML3-2
  CsCML3-2
  SlCML3-1
  StCML3-2
  MeCML3-2
    SlCML7
    StCML7
  SlCML3-2
  GmCML3-1
  GmCML3-2
  GmCML3-3
PvulCML3-3
  BrCML5-3
  CrCML5-2
    AtCML6
  ThCML5-2
    CrCML7
    AtCML7
  GrCML5-2
    GrCML7
  BrCML2-1
  BrCML2-2
    CrCML2
    ThCML2
    AtCML2
  GrCML3-3
  GmCML3-4
  GmCML3-5
    MtCML3
PvulCML3-4
  MeCML3-3
   BdCML28
   PhCML28
   SiCML28
 PvCML28-1
 PvCML28-2
   SbCML28
   ZmCML28
   OsCML28
    MgCML3
    MgCML7
   BdCML22
   PhCML17
   SiCML22
   SbCML22
   OsCML22
   ZmCML17
   OsCML17
    SmCML5
    SmCML7
   CcCML42
   MgCML42
   AtCML42
   AtCML43
   OsCML32
 PaCML42-1
 PaCML42-2
  PaCML5-2
  PaCML5-1
  PaCML5-3
    SmCML4
    SmCML3
 AcCML25-1
PvulCML25-
   BrCML25
   CrCML25
```

```
AtCML25
BrCML25-1
ThCML25
CpCML25-1
EgCML25-1
MeCML25-1
MeCML25-2
PtCML25-1
PtCML25-2
GrCML25-3
GrCML25-5
TcCML25-3
MdCML25-3
MdCML25-4
PperCML25-
CsCML25-1
CsatCML25-
MgCML25-1
MtCML25-4
SlCML25-1
StCML25-1
MtCML25-3
PvulCML25
GrCML25-4
SlCML25-2
GrCML25-1
LuCML25-2
LuCML25-3
CsatCML25-
PaCML25-9
BdCML10
PvCML10-1
SbCML10-1
ZmCML10-1
OsCML10
PvCML10-2
BdCML15
OsCML15
PhCML10
PvCML15
SiCML10
SbCML10-2
ZmCML10
ZmCML10-2
PvCML10-3
AcCML25-2
PaCML25-3
PaCML25-4
PaCML25-2
PaCML25-5
PaCML25-1
PaCML25-7
PaCML25-6
PaCML25-8
AcCML27
AcCML23
CcCML23-1
CsCML23-1
SlCML24
StCML23-2
SlCML23
StCML23-3
MgCML27-2
GmCML27-1
GmCML27-2
PvulCML27-
MtCML24
GmCML27-4
PvulCML27-
CsatCML23
BrCML23
CrCML23
AtCML23
BrCML24
ThCML24
CrCML24
AtCML24
LuCLM24
ThCML23-2
MeCML23
PtCML23-1
PtCML23
CpCML24
GrCML23-1
GrCML23-2
TcCML23
EgCML24-1
FvCML23
MdCML27-5
MdCML27-6
PperCML27-
FvCML27-1
BrCML27-2
AtCML27
BrCML27-1
ThC27
BrCML26
CrCML26
AtCML26
ThCML26
CpCML27
CcCML27
CsCML27
GrCML27-2
TcCML27
GrCML27-3
CsatCML27
GmCML27-5
PvulCML27-
MtCML27
GmCML27-3
PvulCML27-
LuCML27-1
LuCML27-2
LuCML27-3
PtCML27-2
PtCML27-3
MeCML27-1
MeCML27-2
EgCML26
FvCML27-2
PperCML27-
MdCML27-1
MdCML27-2
MdCML27-3
GrCML27-1
MgCML27-1
SlCML27-2
SlCML27-1
EgCML23
EgCML24-2
BdCML16
OsCML16
PhCML16
PvCML16
SiCML16
SbCML16
ZmCML16
PhCML18
PvCML18-2
PvCML18-1
SbCML18
SiCML18
OsCML18
AcCML30
AcCML30-2
BrCML30
ThCML30
CrCML30
AtCML30
LuCML30-1
LuCML30-2
CcCML30-2
CsCML30-1
MeCML30-1
PtCML30-1
MdCML30-3
PperCML30-
MdCML30-4
```

VvCM30
GmCML30-1
PvulCML30-
MeCML30-3
CsatCML30-
MgCML30
SlCML30-2
StCML30-1
EgCML30-1
EgCML30-2
CcCML30-1
CsCML30-2
GrCML30-1
TcCML30
GrCML30-2
MeCML30-2
PtCML30-2
GmCML30-2
PvulCML30-
MtCML30-2
MtCML30-3
MtCML30-1
PvulCML30-
MdCML30-5
PperCML30-
CsatCML30-
SlCML30-1
StCML30-2
BdCML29
PhCML6
SbCML1
BdCML20
PhCML20
SbCML20
ZmCML20-2
ZmCML20-1
SiCML20
OsCML20
AcCML25-5
CcCML25
CsCML25-2
GmCML25
PvulCML25-
MtCML25-2
MdCML25-2
PperCML25-
TcCML25-2
PtCML25-3
CsatCML25
AcCML25-4
CpCML25-2
GrCML25-2
TcCML25-1
PtCML27-1
MtCML25-1
PvulCML25-
RcCML25
FvCML25
PperCML25-
MdCML25-1
CcCML23-2
CsCML23-2
MgCML25-2
StCML25-2
CsatCML36
PpCML23
PpCML25-3
PpCML25-8
PpCML25-9
PpCML25-6
PpCML25-1
PpCML25-2
PpCML25-11
PpCML25-10
PpCML25-4
PpCML25-5
PpCML25-7
PpCML25-12
SmCML27
SmCML26
BrCML36
ThCML36
CrCML36-1
AtCML36
CrCML36-2
BrCML35
CrCML35
AtCML35
CpCML35
MeCML36
FvCML36
MdCML36-1
PperCML36
MdCML36-2
MdCML36-3
MdCML36-4
MtCML36
CcCML36
CsCML36
LuCML36-1
LuCML36-2
MgCML36-1
MgCML36-2
SlCML36
StCML36-2
StCML36-3
SlCML35
StCML36-1
VvCML36
OsCML1-1
OsCML1-2
AcCML38
EgCML37
TcCML37
VvCML39-2
FvCML38-2
PperCML38-
RcCML39
CcCML37
BrCML37
AtCML37
ThCML39
CrCML37
CpCML37
SlCML38-1
StCML38-3
SlCML39-2
StCML37
StCML38-2
SlCML38-2
StCML38-1
BrCML39-1
ThCML23-1
BrCML39-2
CrCML39
AtCML39
BrCML38-2
ThCML38
BrCML38-1
AtCML38
CrCML38
AcCML41-2
AcCML41-1
AtCML41
EgCML41
CcCML41
CsCML41
MdCML41-2
MdCML41-1
MtCML41-1
PvulCML41
PtCML41
RcCML41
SlCML41
StCML41
CpCML40
CcCML38-2
CsCML38-2
FvCML38-1

```
  MtCML41-2
 PvulCML38-
 PvulCML38-
  EgCML40-2
  MgCML38-2
  MgCML38-1
  MgCML38-3
 CsatCML38-
  CsatCML41
  SlCML37-1
  StCML38-4
  SlCML37-2
  StCML23-1
  CcCML38-1
  CsCML38-1
  FvCML38-3
  MdCML39-2
  MdCML39-1
    MdCML38
 PperCML38-
    TcCML38
  MtCML38-1
  MtCML38-2
  MtCML38-3
    PtCML37
 PvulCML38-
 CsatCML38-
    EgCML40
  EgCML39-1
    EgCML38
  EgCML39-2
  EgCML40-1
  VvCML39-1
 PvulCML38-
 PvulCML38-
  VvCML41-1
  VvCML41-2
    VvCML38
  VvCML41-3
    LuCML38
 CsatCML38-
    AtCML40
    AcCML39
    BdCML31
    OsCML31
    PhCML31
    SbCML31
    PhCML19
    SiCML19
    OsCML19
    SbCML19
    ZmCML19
    BdCML23
    SiCML23
    OsCML23
    OsCML25
    OsCML26
  SlCML39-1
    AcCML21
    MtCML21
    VvCML21
    CpCML21
    TcCML21
    CcCML21
    CsCML21
    FvCML21
  PperCML21
    MgCML21
    SlCML21
    StCML21
    CrCML21
    AtCML21
    ThCML21
  CsatCML21
    SiCML33
    SbCML33
    OsCML33
    SiCML29
    SbCML29
    OsCML29
    PaCML21
    PpCML21
    AcCML22
    CsCML22
    TcCML22
  PperCML12
    FvCML22
  CsatCML22
    MgCML22
    StCML22
    ThCML22
    AtCML22
    BdCML32
    SbCML34
    OsCML34
  SmCML21-1
  SmCML21-2
    MpCML12
  BrCML32-1
    PvCML36
    PvCML29
  ZmCML36-1
    MeCML32
  ZmCML36-3
  ZmCML36-2
    MpCML11
 CreinCML20
 CreinCML20
    AtCML48
    AtCML49
    AtCML50
 CreinCML32
    RcCML23  RFVLGWK
     SbCML6
    SbCML15
     VcCML5
    AcCML18
    MeCML18
    RcCML18
    SlCML18
  StCML18-1
    CpCML18
    MgCML18
  PtCML18-1
  PtCML18-2
    CcCML18
    CsCML17
  FvCML18-2
  PperCML18
    MdCML18
    TcCML18
    EgCML17
  StCML18-2
  GrCML18-2
    GmCML17
  PvulCML18
    MtCML18
  CsatCML18
  GrCML18-1
    BdCML11
    PhCML11
    SbCML11
    ZmCML11
    OsCML11
    GmCML18
    BrCML18
    ThCML18
    CrCML18
    AtCML18
  LuCML18-1
    BrCML17
    CrCML17
    AtCML17
    ThCML17
    SlCML17
  FvCML18-1
  LuCML18-2
    EgCML18
  MdCML17-3
```

```
  MdCML17-2
  PpCML17-1
  PpCML17-2
    AcCML16
    FvCML15
    BdCML12
    PhCML12
    PvCML12
    SiCML12
    SbCML12
  ZmCML14-1
    OsCML12
    BdCML14
    PhCML14
  PvCML14-1
  PvCML14-2
    SiCML14
    SbCML14
  ZmCML14-2
    OsCML14
  BrCML16-1
  BrCML16-2
    ThCML16
    AtCML16
    CrCML16
    CpCML16
  MeCML16-1
    PtCML16
    RcCML16
    MgCML26
  PperCML16
  VvCML16-2
    CcCML16
    CsCML16
  LuCML15-2
  MeCML16-2
    SlCML16
    GmCML16
  PvulCML16
  EgCML15-2
    CpCML15
    CcCML15
    CsCML15
  CsatCML15
  MdCML15-2
  PperCML15
  MdCML15-1
  GrCML15-2
  GrCML15-3
    TcCML15
    MeCML15
    RcCML15
    PtCML15
  VvCML16-1
    SlCML15
    StCML15
    BrCML15
    CrCML15
    AtCML15
    ThCML15
  CsatCML16
    GrCML16
  EgCML15-1
  GmCML15-2
  GmCML15-3
  PvulCML15
  GmCML15-1
    MtCML15
  GrCML15-1
    FvCML16
    LuCML15
    AcCML20
  GrCML20-2
    FvCML20
  MdCML17-1
    MdCML20
  PperCML20
    CcCML20
    CsCML20
    CpCML20
    PtCML20
  MeCML20-1
    CrCML20
    AtCML20
    ThCML20
  CsatCML20
    SlCML20
  GrCML20-1
    EgCML20
    StCML20
    GmCML20
  PvulCML20
    MtCML20
  VvCML20-1
  VvCML20-2
     BdCML8
     SbCML8
   ZmCML8-2
   PhCML8-1
   PvCML8-2
    ThCML19
    AtCML19
  MeCML20-2
  MpCML20-2
    SmCML20
    OlCML20
  VcCML20-1
  VcCML20-2
   PhCML8-2
   PvCML8-1
   PvCML8-3
   ZmCML8-1
     OsCML8
    SmCML19
  MpCML20-1
    AcCML11
  GmCML11-3
  GmCML11-4
  MeCML11-2
  MeCML11-1
 PperCML11-
  GrCML11-3
  BrCML11-2
  BrCML11-3
    CrCML11
    AtCML11
    ThCML11
  BrCML11-1
    FvCML11
 PperCML11-
  GrCML11-2
  GmCML11-1
  GmCML11-2
  PvulCML11
  PtCML11-1
     BdCML4
     OsCML4
     PhCML4
     PvCML4
     ZmCML4
     PhCML5
   PvCML5-2
   PvCML5-1
     SiCML5
     ZmCML5
     SbCML5
     OsCML5
    EgCML11
     BdCML1  KCVIL
     BdCML3  SCTIL
     OsCML2  SCTIL
     OsCML3  SCTIL
  CsatCML50  QCCVL
     SiCML2
     ZmCML2
  GrCML11-1
  PtCML11-2
  PtCML11-3
 PperCML11-
```

```
BdCML5
OsCML6
MdCML11
AtCML13
AtCML14
BrCML34-2
BrCML34-1
CrCML34
AtCML34
ThCML34
BrCML32-2
CrCML32
CrCML32-2
CrCML32-1
AtCML32
AtCML31
ThCML32
ThCML33
AtCML33
EgCML25-2
SmCML25
LuCML25-1
AcCML25-3
BdCML2
CrCML12
AtCML12
CsubCML24
PpCML7
MpCML21-1
MpCML4
MpCML30
MpCML15
MtCML46
AtCML45
AtCML46
AtCML47
VcCML4
OsCML1-3
AtCML44
OlCML11
AtCML1
CsubCML20
SiCML36
SbCML50
MtCML11
Consensus
```
